# Virome Characterization of a Collection of *S. sclerotiorum* from Australia

**DOI:** 10.3389/fmicb.2017.02540

**Published:** 2018-01-11

**Authors:** Fan Mu, Jiatao Xie, Shufen Cheng, Ming Pei You, Martin J. Barbetti, Jichun Jia, Qianqian Wang, Jiasen Cheng, Yanping Fu, Tao Chen, Daohong Jiang

**Affiliations:** ^1^State Key Laboratory of Agricultural Microbiology, Huazhong Agricultural University, Wuhan, China; ^2^The Provincial Key Lab of Plant Pathology of Hubei Province, Huazhong Agricultural University, Wuhan, China; ^3^Faculty of Science, UWA School of Agriculture and Environment and The UWA Institute of Agriculture, The University of Western Australia, Crawley, WA, Australia

**Keywords:** *Sclerotinia sclerotiorum*, RNA_sequencing, mycovirus, virome, Australia, virus diversity, biocontrol

## Abstract

*Sclerotinia sclerotiorum* is a devastating plant pathogen that attacks numerous economically important broad acre and vegetable crops worldwide. Mycoviruses are widespread viruses that infect fungi, including *S. sclerotiorum*. As there were no previous reports of the presence of mycoviruses in this pathogen in Australia, studies were undertaken using RNA_Seq analysis to determine the diversity of mycoviruses in 84 Australian *S. sclerotiorum* isolates collected from various hosts. After RNA sequences were subjected to BLASTp analysis using NCBI database, 285 contigs representing partial or complete genomes of 57 mycoviruses were obtained, and 34 of these (59.6%) were novel viruses. These 57 viruses were grouped into 10 distinct lineages, namely *Endornaviridae* (four novel mycoviruses), *Genomoviridae* (isolate of SsHADV-1), *Hypoviridae* (two novel mycoviruses), *Mononegavirales* (four novel mycovirusess), *Narnaviridae* (10 novel mycoviruses), *Partitiviridae* (two novel mycoviruses), *Ourmiavirus* (two novel mycovirus), *Tombusviridae* (two novel mycoviruses), *Totiviridae* (one novel mycovirus), *Tymovirales* (five novel mycoviruses), and two non-classified mycoviruses lineages (one *Botrytis porri* RNA virus 1, one distantly related to *Aspergillus fumigatus* tetramycovirus-1). Twenty-five mitoviruses were determined and mitoviruses were dominant in the isolates tested. This is not only the first study to show existence of mycoviruses in *S. sclerotiorum* in Australia, but highlights how they are widespread and that many novel mycoviruses occur there. Further characterization of these mycoviruses is warranted, both in terms of exploring these novel mycoviruses for innovative biocontrol of Sclerotinia diseases and in enhancing our overall knowledge on viral diversity, taxonomy, ecology, and evolution.

## Introduction

Mycoviruses are viruses that replicate in fungi and are found in all major fungal kingdom groups (Ghabrial et al., 2015). While mycoviruses can be grouped into 14 families, as released by the International Committee on Taxonomy of Viruses (ICTV) in 2016, many remain unclassified. Mycoviruses that represent novel types of viruses are continually being discovered (Kanhayuwa et al., 2015; Lin et al., 2015; Ong et al., 2016; Zhang et al., 2016; Jia et al., 2017). While many mycoviruses are associated with latent infection that does not cause any obvious impact on their fungal hosts, some do cause significant effects on host growth, development, and reproduction. Hypovirulence-associated mycoviruses have a potential to control fungal plant pathogens and a classic example is using a hypovirus to control chestnut blight (Zhang and Nuss, 2016; Rigling and Prospero, 2017). A hypovirulence-associated mycovirus in *Rosellinia necatrix* has been found have potential to control white root rot of fruit trees (Chiba et al., 2009; Kondo et al., 2013); A fungal DNA virus was recently found to have potential to control Sclerotinia diseases (Yu et al., 2013; Liu et al., 2016). Such discoveries provide stimulus for the investigation of mycoviruses in and across many different fungal pathogens.

*Sclerotinia sclerotiorum* is an ascomycetous plant-pathogenic fungus with worldwide distribution and ability to attack over 400 species of plants including numerous weeds and important crops, such as sunflower, soybean, oilseed rape, and various vegetables (Bolton et al., 2006). In Australia, *S. sclerotiorum* is a serious disease of many cruciferous crops and frequently poses a threat to their sustainable and profitable production (Barbetti et al., 2014; Uloth et al., 2014; You et al., 2016), particularly canola (*Brassica napus*) and it can also be serious on other non-cruciferous crops such as sunflower (Ekins et al., 2007) and legumes (Bretag et al., 1987). Diseases caused by *S. sclerotiorum* are very difficult to control and currently, cultural and chemical controls provide only partial control and can be cost prohibitive, particularly for broad acre oilseed *Brassica* crops in countries such as Australia, India, and China (Barbetti et al., 2014). Utilizing mycoviruses as biocontrol agents is an alternative way to reduce the ecomonical damage of crops caused by *S. sclerotiorum*.

Mycoviruses that infect *S. sclerotiorum* have been investigated by several research groups in China, USA, and New Zealand, and they found that *S. sclerotiorum* hosts various mycoviruses, including double stranded RNA (dsRNA) viruses, positive-sense single-stranded RNA (+)ssRNA viruses, DNA virus and negative-sense single-stranded RNA (−)ssRNA viruses (Yu et al., 2010; Liu, 2014; Xie and Jiang, 2014; Marzano et al., 2015). Some novel mycoviruses were reported to transmit even amongst pathogen strains that are vegetative incompatible *via* various strategies. For example, fungal DNA virus, *Sclerotinia gemycircular* virus 1, originally called *Sclerotinia sclerotiorum* hypovirulence-associated DNA virus 1 (SsHADV-1), could infect a mycophagous insect and derive it as a transmission vector, and its particles are able to directly invade the hyphae of *S. sclerotiorum* (Yu et al., 2013; Liu et al., 2016); the mycoreovirus *Sclerotinia sclerotiorum* mycoreovirus 4(SsMYRV4) could suppress host's vegetative incompatibility reaction resulting in facilitation of its transmission as well as the transmission of other viruses in populations of *S. sclerotiorum* (Wu et al., 2017); while a hypovirulence-associated partitivirus, *Sclerotinia sclerotiorum* partitivirus 1(SsPV1), could spread through donor and receptor hyphal fusion and also confer hypovirulence to *Botrytis cinerea* (Xiao et al., 2014). These successful discoveries of novel viruses which seemingly overcome the transmission barriers open the prospect for utilization of mycoviruses for biological control of Sclerotinia diseases.

Deep sequencing techniques have been used to efficiently discover novel viruses in fungi. Examples include: Marzano et al. (2016) who collected RNA samples of *Colletotrichum truncatum, Macrophomina phaseolina, Diaporthe longicolla, Rhizoctonia solani*, and *S. sclerotiorum*, and used RNA_Seq to obtain virus contigs from fungal RNA samples. Their research led to the discovery of novel mycoviruses, never previously found in fungi, that belonged to the families *Benyviridae, Ophioviridae*, and *Virgaviridae* (Marzano et al., 2016); and Khalifa et al. (2016) who discovered 10 viruses in five *S. sclerotiorum* strains using Illumina sequencing (Khalifa et al., 2016), and Osaki et al. (2016), using deep sequencing, found that 17 mycoviruses co-infected a strain of *Fusarium poae*. Mycoviruses were found on the soybean phyllosphere and in the roots colonized by arbuscular mycorrhizal fungi (Ezawa et al., 2015; Marzano and Domier, 2016). Australia and New Zealand are geographically embraced by oceans as a natural barrier to spread of pathogens, unlike in New Zealand, whether viruses that infect *S. sclerotiorum* are present in Australia has never been determined. The population structure of *S. sclerotiorum* in Australia is known to be uniquely diverse, in terms of sub-specific (i.e., pathogen pathotypes) (Ge et al., 2012) and genetic (i.e., pathogen haplotypes) (Clarkson et al., 2017) variation, making the Australian populations of this pathogen a particularly interesting target. Further, with control of *S. sclerotiorum* such a challenge worldwide, that mycoviruses could potentially be utilized to manage *S. sclerotiorum* brings additional urgency in determining the presence and nature of mycoviruses in this important pathogen in Australia. In this study, we utilized RNA_Seq analysis and RT-PCR amplification to determine the mycoviruses present in 84 isolates of *S. sclerotiorum* collected from various crops and across several agricultural regions of Australia.

## Materials and methods

### *S. sclerotiorum* isolates and growth conditions

*S. sclerotiorum* isolates were recovered from sclerotia collected from diseased *Apium graveolens, B. napus, B. oleracea* var. *botrytis, B. oleracea* var. *capitata, Cichorium intybus* “Witlof,” *Daucus carota, Lactuca sativa, Lupinus angustifolius, Phaseolus vulgaris*, and *Solanum tuberosum* (see Table S5 for background information on isolates). All isolates were cultured on laboratory-produced potato dextrose agar (PDA) (200 g of peeled potato, 20 g of dextrose, and 20 g of agar in 1,000 ml distilled water) at 20°C. Isolates were maintained on PDA slants at 4^o^C throughout these studies.

### Total RNA extraction and purification

To extract RNA samples conveniently, the 84 *S. sclerotiorum* isolates were divided into 17 groups of five isolates each, except Group 17 which had only four strains. To extract total RNA, each of the 84 isolates was cultured on a cellophane membrane overlaying a PDA plate for 3–5 days. One-gram mycelial mass was collected from each strain and mycelial mass of each group was mixed and ground in liquid nitrogen with a mortar and pestle to fine powder. Total RNA was prepared by using a Trizol RNA extraction kit (Takara Bio, Inc. Japan) according to the manufacturer's instructions and treated with DNase I. The total RNA was stored at −80°C. Each group provided about 1,500 ng for RNA-Seq analysis, the total RNA of 17 groups were mixed, and then the mixed sample used for RNA_Seq analysis.

### RNA sequencing and sequence analysis

83.4 μg total RNA was used for RNA-Seq. Sequencing was performed on the Illumina MiSeq 2000/2500 by the Shanghai Biotechnology Corporation. Sequencing libraries were prepared from mixed rRNA-depleted total RNA samples extracted from the 84 isolates. And the TruSeq™ RNA Sample Prep Kit (Illumina, RS-122-2001) was used for library construction. The unqualified reads were filtered out, contained paired-end reads shorter than 100 bp, low quality scores (<20) in the raw data, the RNA and genome sequence of the *S. sclerotinia* and the linker sequence. Then clean reads were assembled by a metagenomic *de novo* assembly for the CLC Genomics Workbench (version: 6.0.4). Primary UniGenes were obtained and then CAP3 EST used to splice primary UniGenes to construct the first_contig (contiguous sequence) and the second_contig. The contigs obtained were then subjected to BLAST against GenBank using BLASTn and BLASTp (the nucleotide sequences of the contigs were converted into amino acid sequences then a BLASTp search was run).

### Confirmation of putative mycoviruses

To verify the presence of putative mycovirus in the strains, cDNAs were synthesized by using Moloney murine leukemia virus (M-MLV) transcriptase (Takara Bio, Inc., Japan). Then, assembled contigs (58 contigs) that matched viral sequences were used to design detection primers (see Table S2 for details). Viral sequences were detected using RT-PCR (Polymerase Chain Reaction).

### Phylogenetic analysis

The nucleotide sequences and translated amino acid sequences of contigs with high similarity to known viral nucleic acids and proteins in GenBank were used for phylogenetic analysis. Alignments were performed by Clustal W in the MEGA7 and phylogenetic trees were constructed by the Maximum likelihood method with a bootstrap value of 1,000 replicates through MEGA 7.0.18 (http://www.megasoftware.net/megamacBeta.php). For some contigs that were incomplete and could not be used for phylogenetic analysis, the relationships were judged based on the result of the BLASTn. Viruses and accession numbers of viral gene(s) which were selected to perform phylogenetic analysis are listed in Table S3.

## Results and discussion

### Metatranscriptomic identification of mycoviruses infecting tested *S. sclerotiorum* isolates

**Figure 1:**
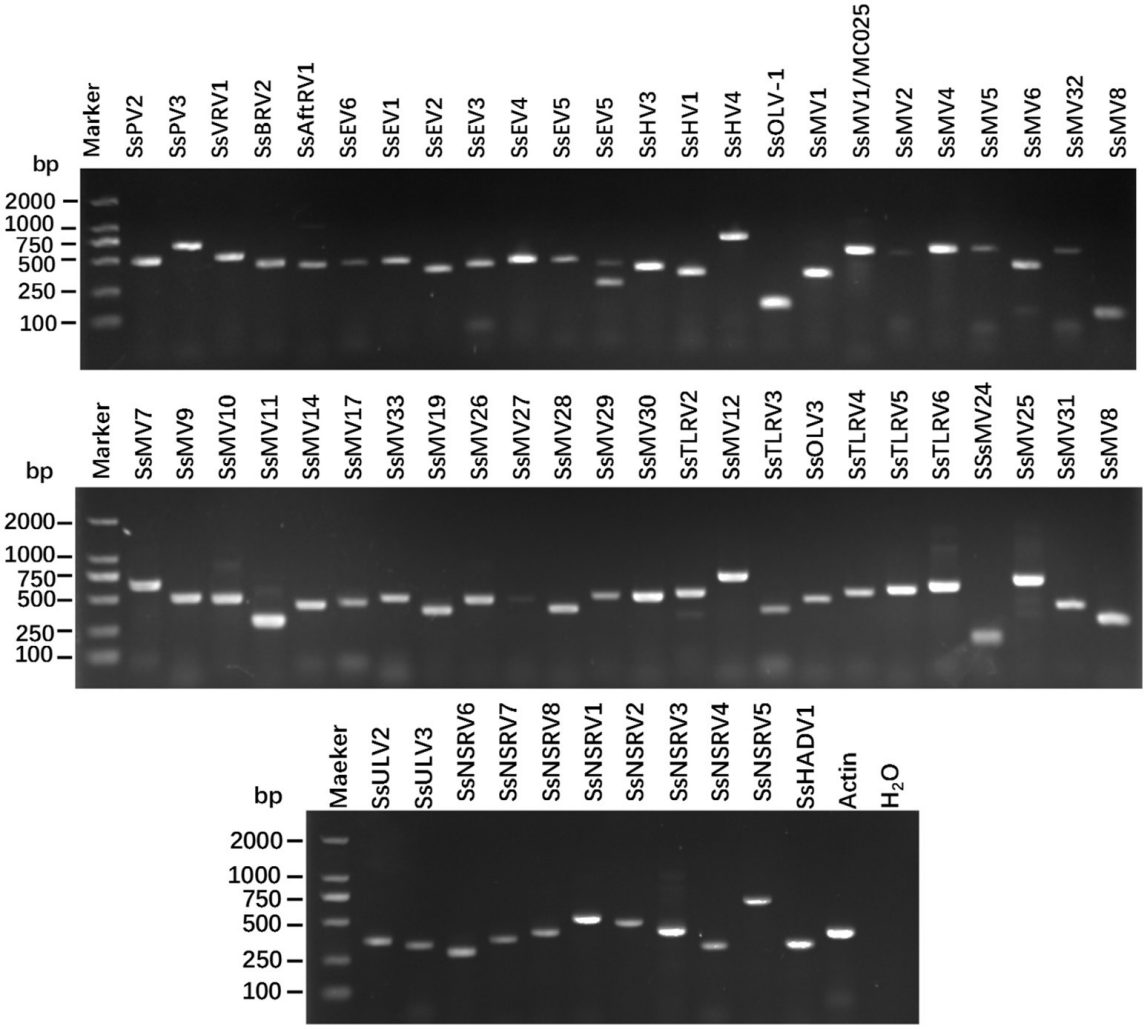
RT-PCR confirmation of mycovirus contigs. RT-PCR confirmation of *de novo* assembled mycovirus contigs from *Sclerotinia sclerotiorum* generated by Illumina sequencing. The presences of viral sequences identified by BLAST analysis were confirmed by RT-PCR products generated from total RNA samples. The primers were designed according to the contigs' sequences (genomic sequences of putative mycoviruses). Primers pairs used and predicted sizes of amplicons are listed in Table S3. Lane M, DNA marker, 2,000 bp DNA Ladder (Takara Bio Inc., Japan); Lane 1 to 58, abbreviates of viruses (see Table 1 for detail), Lane H_2_O, ddH_2_O was instead of RT products as control.

After removal of any low-quality reads, 7.3^*^10^7^ reads with lengths >20-nt (paired-end) were obtained. These reads were *de novo* assembled into large contigs. Consequently, 19,539 contigs were achieved. All contigs were subjected to BLAST analysis. As a result of the analysis, 285 contigs which represented partial or complete genome segments of 57 mycoviruses were obtained (Table 1 and Table S1). Provisional names and the most closely related viruses are listed in Table 1. PCR amplification further demonstrated that these putative viruses existed within these isolates (Figure 1). The majority of the putative viruses were predicted with (+)ssRNA genomes accounting for 75.44% of the total viruses; then dsRNA viruses accounting for 8.77% of the total viruses and (−)ssRNA genomes accounting for 14.04% of the total viruses. Further, a contig whose predicted DNA sequence was most similar to the genome of SsHADV-1 was identified (Yu et al., 2010). In (+)ssRNA viruses, 59.09% viruses were mitoviruses, with a total of 25 mitoviruses identified from the 84 isolates. The remainder of the (+)ssRNA genomes were related to viruses in *Hypoviridae, Endornaviridae, Tombusviridae, Tymoviridae*, and *Gammaflexiviridae*. The dsRNA virus genomes were most similar to viruses in *Partitiviridae, Totiviridae*, and *Megabirnaviridae*, and there were some additional unclassified mycoviruses. The sequences of 285 contigs were listed in Table S2.

**Table 1:** Assembled sequences with similarity to previously described viruses.

**Number**	**Contig number**	**GenBank accession numbers**	**Contig length**	**Name of putative viruses**	**Best match**	**aa identity (%)**	**Genome type**	**Family/Genus**	**Reference**
1	Contig 9550	MF444211	649	Sclerotinia sclerotiorum victorivirus 1 (SsVV1)	Sclerotinia nivalis victorivirus 1 (YP_009259368.1)	81	dsRNA	*Totiviridae*	Wu et al., 2016
2	Contig 661	MF444213	1,547	Sclerotinia sclerotiorum partitivirus 2 (SsPV2)	Pseudogymnoascus destructans partitivirus-pa (YP_009259751.1)	46	dsRNA	*Partitiviridae*	Ren et al., 2016
3	Contig 904	MF444214	1,769	Sclerotinia sclerotiorum partitivirus 3 (SsPV3)	Verticillium albo-atrum partitivirus-1 (AIE47664.1)	67	dsRNA	*Partitiviridae*	Cañizares et al., 2014
4	Contig 823	MF444216	5,417	Sclerotinia sclerotiorum botybirnavirus 3 (SsBRV3)	Botrytis porri RNA virus 1 (YP_006390637.1)	99	dsRNA	Unclassified	Wu et al., 2012
5	Contig 6913	MF444217	2,612	Sclerotinia sclerotiorum tetramycovirus-1 (SstRV1)	Aspergillus fumigatus tetramycovirus-1 (CDP74618.1)	45	dsRNA	Unclassified	Kanhayuwa et al., 2015
6	Contig 259	MF444220	10,205	Sclerotinia sclerotiorum hypovirus 1-A (SsHV1)	Sclerotinia sclerotiorum hypovirus 1 (YP_004782527.1)	99	+ssRNA	*Hypoviridae*	Xie et al., 2011
7	Contig 1373	MF444222	4,624	Sclerotinia sclerotiorum hypovirus 3 (SsHV3)	Sclerotinia sclerotiorum hypovirus 1 (YP_004782527.1)	76	+ssRNA	*Hypoviridae*	Xie et al., 2011
8	Contig 1333	MF444225	1,665	Sclerotinia sclerotiorum hypovirus 4 (SsHV4)	Cryphonectria hypovirus 3 (NP_051710.1)	61	+ssRNA	*Hypoviridae*	Smart et al., 1999
9	Contig 7442	MF444226	2,509	Sclerotinia sclerotiorum endornavirus1-A (SsEV1)	Sclerotinia sclerotiorum endornavirus-1 (YP_008169851.1)	98	+ssRNA	Endornaviridae	Marzano et al., 2016
10	Contig 3829	MF444227	7,517	Sclerotinia sclerotiorum endornavirus2-A (SsEV2)	Sclerotinia sclerotiorum endornavirus-2 (AND83000.1)	95	+ssRNA	*Endornaviridae*	Khalifa and Pearson, 2014a
11	Contig 1364	MF444228	1,617	Sclerotinia sclerotiorum endornavirus 3 (SsEV3)	Rhizoctonia cerealis endornavirus 1 (YP_008719905.1)	49	+ssRNA	*Endornaviridae*	Li et al., 2014
12	Contig 282	MF444229	11,034	Sclerotinia sclerotiorum endornavirus 4 (SsEV4)	Vicia faba endornavirus (YP_438201.1)	24	+ssRNA	*Endornaviridae*	Pfeiffer, 1998
13	Contig 6278	MF444230	2,757	Sclerotinia sclerotiorum endornavirus 5 (SsEV5)	Rhizoctonia solani endornavirus—RS002 (AHL25280.1)	31	+ssRNA	*Endornaviridae*	Das et al., 2014
14	Contig 14516	MF444232	551	Sclerotinia sclerotiorum endornavirus 6 (SsEV6)	Discula destructiva virus 3 (AF375469.1)	49	+ssRNA	*Endornaviridae*	Rong et al., 2002
15	Contig 192	MF444233	2,493	Sclerotinia sclerotiorum mitovirus 1-A (SsMV1)	Sclerotinia sclerotiorum mitovirus 1 (AEX91878.1)	83	+ssRNA	*Narnaviridae*	Xie and Ghabrial, 2012
16	Contig 67	MF444235	2,477	Sclerotinia sclerotiorum mitovirus 1 HC025-A (SsMV1/HC025)	Sclerotinia sclerotiorum mitovirus 1 HC025 (YP_009121785.1)	90	+ssRNA	*Narnaviridae*	Xu et al., 2015
17	Contig 5	MF444236	1,897	Sclerotinia sclerotiorum mitovirus 2-A (SsMV2)	Sclerotinia sclerotiorum mitovirus 2 (AHX84129.1)	84	+ssRNA	*Narnaviridae*	Khalifa and Pearson, 2014b
18	Contig 238	MF444237	2,737	Sclerotinia sclerotiorum mitovirus 4-A (SsMV4)	Sclerotinia sclerotiorum mitovirus 4 (AGC24233.1)	89	+ssRNA	*Narnaviridae*	Pfeiffer, 1998
19	Contig 188	MF444238	1,524	Sclerotinia sclerotiorum mitovirus 5-A (SsMV5)	Sclerotinia sclerotiorum mitovirus 5 (AHX84130.1)	86	+ssRNA	*Narnaviridae*	Khalifa and Pearson, 2014b
20	Contig 308	MF444239	2,510	Sclerotinia sclerotiorum mitovirus 6-A (SsMV6)	Sclerotinia sclerotiorum mitovirus 6 (AHF48622.1)	79	+ssRNA	*Narnaviridae*	Khalifa and Pearson, 2014b
21	Contig 69	MF444241	2,602	Sclerotinia sclerotiorum mitovirus 7-A (SsM7)	Sclerotinia sclerotiorum mitovirus 7 (AHF48623.1)	82	+ssRNA	*Narnaviridae*	Khalifa and Pearson, 2014b
22	Contig 42	MF444243	1,489	Sclerotinia sclerotiorum mitovirus 8-A (SsMV8)	Sclerotinia sclerotiorum mitovirus 8 (AHF48624.1)	87	+ssRNA	*Narnaviridae*	Marzano et al., 2016
23	Contig 202	MF444244	2,510	Sclerotinia sclerotiorum mitovirus 9-A (SsMV9)	Sclerotinia sclerotiorum mitovirus 9 (AHF48625.1)	96	+ssRNA	*Narnaviridae*	Marzano et al., 2016
24	Contig 226	MF444245	808	Sclerotinia sclerotiorum mitovirus 10-A (SsMV10)	Sclerotinia sclerotiorum mitovirus 10 (AHF48626.1)	86	+ssRNA	*Narnaviridae*	Marzano et al., 2016
25	Contig 3	MF444246	772	Sclerotinia sclerotiorum mitovirus 11-A (SsMV11)	Sclerotinia sclerotiorum mitovirus 11 (AHF48627.1)	83	+ssRNA	*Narnaviridae*	Marzano et al., 2016
26	Contig 26	MF444247	1,176	Sclerotinia sclerotiorum mitovirus 12-A (SsMV12)	Sclerotinia sclerotiorum mitovirus 12 (AHF48628.1)	80	+ssRNA	*Narnaviridae*	Marzano et al., 2016
27	Contig 126	MF444249	2,557	Sclerotinia sclerotiorum mitovirus 14 -A (SsMV14)	Sclerotinia sclerotiorum mitovirus 14 (AHF48630.1)	82	+ssRNA	*Narnaviridae*	Marzano et al., 2016
28	Contig 73	MF444250	1,559	Sclerotinia sclerotiorum mitovirus 17 –A (SsMV17)	Sclerotinia sclerotiorum mitovirus 17 (ALD89134.1)	95	+ssRNA	*Narnaviridae*	Marzano et al., 2016
29	Contig 569	MF444253	1,448	Sclerotinia sclerotiorum mitovirus 19-A (SsMV19)	Sclerotinia sclerotiorum mitovirus 19 (ALD89136.1)	88	+ssRNA	*Narnaviridae*	Marzano et al., 2016
30	Contig 24	MF444254	1,243	Sclerotinia sclerotiorum mitovirus 24 (SsMV24)	Sclerotinia sclerotiorum mitovirus 7 (AHF48623.1)	62	+ssRNA	*Narnaviridae*	Marzano et al., 2016
31	Contig 15	MF444255	1,447	Sclerotinia sclerotiorum mitovirus 25 (SsMV25)	Sclerotinia sclerotiorum mitovirus 5 (AHX84130.1)	62	+ssRNA	*Narnaviridae*	Marzano et al., 2016
32	Contig 634	MF444256	1,992	Sclerotinia sclerotiorum mitovirus 26 (SsMV26)	Rhizoctonia solani mitovirus 10 (ALD89102.1)	51	+ssRNA	*Narnaviridae*	Marzano et al., 2016
33	Contig 958	MF444257	2,917	Sclerotinia sclerotiorum mitovirus 27 (SsMV27)	Ophiostoma mitovirus 1a (CAJ32466.1)	51	+ssRNA	*Narnaviridae*	Marzano et al., 2016
34	Contig 867	MF444258	2,721	Sclerotinia sclerotiorum mitovirus 28 (SsMV28)	Botrytis cinerea mitovirus 3 (YP_009182161.1)	37	+ssRNA	*Narnaviridae*	Unpublished
35	Contig 536	MF444259	3,262	Sclerotinia sclerotiorum mitovirus 29 (SsMV29)	Botrytis cinerea mitovirus 3 (YP_009182161.1)	38	+ssRNA	*Narnaviridae*	Unpublished
36	Contig 59	MF444260	2,536	Sclerotinia sclerotiorum mitovirus 30 (SsMV30)	Macrophomina phaseolina mitovirus 1 (ALD89100.1)	83	+ssRNA	*Narnaviridae*	Marzano et al., 2016
37	Contig 131	MF444261	1,696	Sclerotinia sclerotiorum mitovirus 31 (SsMV31)	Sclerotinia sclerotiorum mitovirus 2 (AHX84129.1)	60	+ssRNA	*Narnaviridae*	Marzano et al., 2016
38	Contig 1865	MF444262	2,473	Sclerotinia sclerotiorum mitovirus 32 (SsMV32)	Sclerotinia sclerotiorum mitovirus 6 (AHF48622.1)	70	+ssRNA	*Narnaviridae*	Marzano et al., 2016
39	Contig 90	MF444263	2,661	Sclerotinia sclerotiorum mitovirus 33 (SsMV33)	Sclerotinia sclerotiorum mitovirus 19 (ALD89136.1)	45	+ssRNA	*Narnaviridae*	Marzano et al., 2016
40	Contig 758	MF444275	1,470	Sclerotinia sclerotiorum ourmia-like virus 1-A (SsOLV-1-A)	Sclerotinia sclerotiorum ourmia-like virus 1 RNA 1 (ALD89138.1)	76	+ssRNA	*Ourmiavirus*	Marzano et al., 2016
41	Contig 30	MF444276	2,442	Sclerotinia sclerotiorum ourmia-like virus 3 (SsOLV-3)	Sclerotinia sclerotiorum ourmia-like virus 2 (ALD89139.1)	37	+ssRNA	*Ourmiavirus*	Marzano et al., 2016
42	Contig 224	MF444273	4,170	Sclerotinia sclerotiorum umbra-like virus 2 (SsULV2)	Sclerotinia sclerotiorum umbra-like virus 1 (YP_009253998.1)	84	+ssRNA	*Tombusviridae*	Marzano et al., 2016
43	Contig 534	MF444274	3,925	Sclerotinia sclerotiorum umbra-like virus 3 (SsULV3)	Magnaporthe oryzae RNA virus (YP_009115495.1)	61	+ssRNA	*Tombusviridae*	Marzano et al., 2016
44	Contig 3545	MF444265	1,575	Sclerotinia sclerotiorum tymo-like RNA virus 2 (SsTLRV2)	Nectarine virus M (YP_009222597.1)	37	+ssRNA	*Tombusviridae*	Villamor et al., 2016
45	Contig 10849	MF444266	964	Sclerotinia sclerotiorum tymo-like RNA virus 3 (SsTLRV3)	Turnip yellow mosaic virus (AJF99750.1)	25	+ssRNA	*Tymoviridae*	Morch et al., 1982
46	Contig 11935	MF444267	2,926	Sclerotinia sclerotiorum tymo-like RNA virus 4 (SsTLRV4)	Poinsettia mosaic virus (BAJ14669.1)	40	+ssRNA	*Tymoviridae*	Bsm et al., 2007
47	Contig 14854	MF444268	796	Sclerotinia sclerotiorum tymo-like RNA virus 5 (SsTLRV5)	Fusarium graminearum mycotymovirus 1 (AMN92730.1)	27	+ssRNA	*Tymoviridae*	Li et al., 2015
48	Contig 5512	MF444270	2,307	Sclerotinia sclerotiorum tymo-like RNA virus 6 (SsTLRV6)	Fusarium graminearum deltaflexivirus 1 (YP_009268710.1)	39	+ssRNA	*Gammaflexiviridae*	Marzano and Domier, 2016
49	Contig 88	MF444277	7,735	Sclerotinia sclerotiorum negative-stranded RNA virus 1-A (SsNSRV1)	Sclerotinia sclerotiorum negative-stranded RNA virus 1 (YP_009094317.1)	91	−ssRNA	Mymonaviridae	Liu, 2014
50	Contig 100	MF444278	9,614	Sclerotinia sclerotiorum negative-stranded RNA virus 2-A (SsNSRV2)	Sclerotinia sclerotiorum negative-stranded RNA virus 2 (ALD89145.1)	93	−ssRNA	*Mymonaviridae*	Marzano et al., 2016
51	Contig 29	MF444280	7,104	Sclerotinia sclerotiorum negative-stranded RNA virus 3-A (SsNSRV3)	Sclerotinia sclerotiorum negative-stranded RNA virus 3 (AJT39503.1)	90	−ssRNA	*Mymonaviridae*	Marzano et al., 2016
52	Contig 512	MF444281	9,564	Sclerotinia sclerotiorum negative-stranded RNA virus 4-A (SsNSRV4)	Sclerotinia sclerotiorum negative-stranded RNA virus 4 (ALD89140.1)	94	−ssRNA	*Mymonaviridae*	Marzano et al., 2016
53	Contig 4241	MF444283	4,421	Sclerotinia sclerotiorum negative-stranded RNA virus 5 (SsNSRV5)	Sclerotinia sclerotiorum negative-stranded RNA virus 4 (ALD89140.1)	32	−ssRNA	*Mymonaviridae*	Marzano et al., 2016
54	Contig 89	MF444284	5,076	Sclerotinia sclerotiorum negative-stranded RNA virus 6 (SsNSRV6)	Soybean leaf-associated negative-stranded RNA virus 1 (ALM62220.1)	28	−ssRNA	*Mymonaviridae*	Marzano and Domier, 2016
55	Contig 579	MF444285	7,819	Sclerotinia sclerotiorum negative-stranded RNA virus 7 (SsNSRV7)	Soybean leaf-associated negative-stranded RNA virus 2 (ALM62227.1)	37	−ssRNA	*Mymonaviridae*	Marzano and Domier, 2016
56	Contig 1114	MF444286	1,219	Sclerotinia sclerotiorum negative-stranded RNA virus 8 (SsNSRV8)	Soybean leaf-associated negative-stranded RNA virus 3 (ALM62228.1)	28	−ssRNA	*Mymonaviridae*	Marzano and Domier, 2016
57	Contig 2147	MF444288	2,166	Sclerotinia sclerotiorum hypovirulence associated DNA virus 1 (SsHADV-1)	Sclerotinia sclerotiorum hypovirulence associated DNA virus 1 (AJD07457.1)	99	DNA	*Genomoviridae*	Yu et al., 2010

### One predicted novel virus in family *Totiviridae*

**Figure 2:**
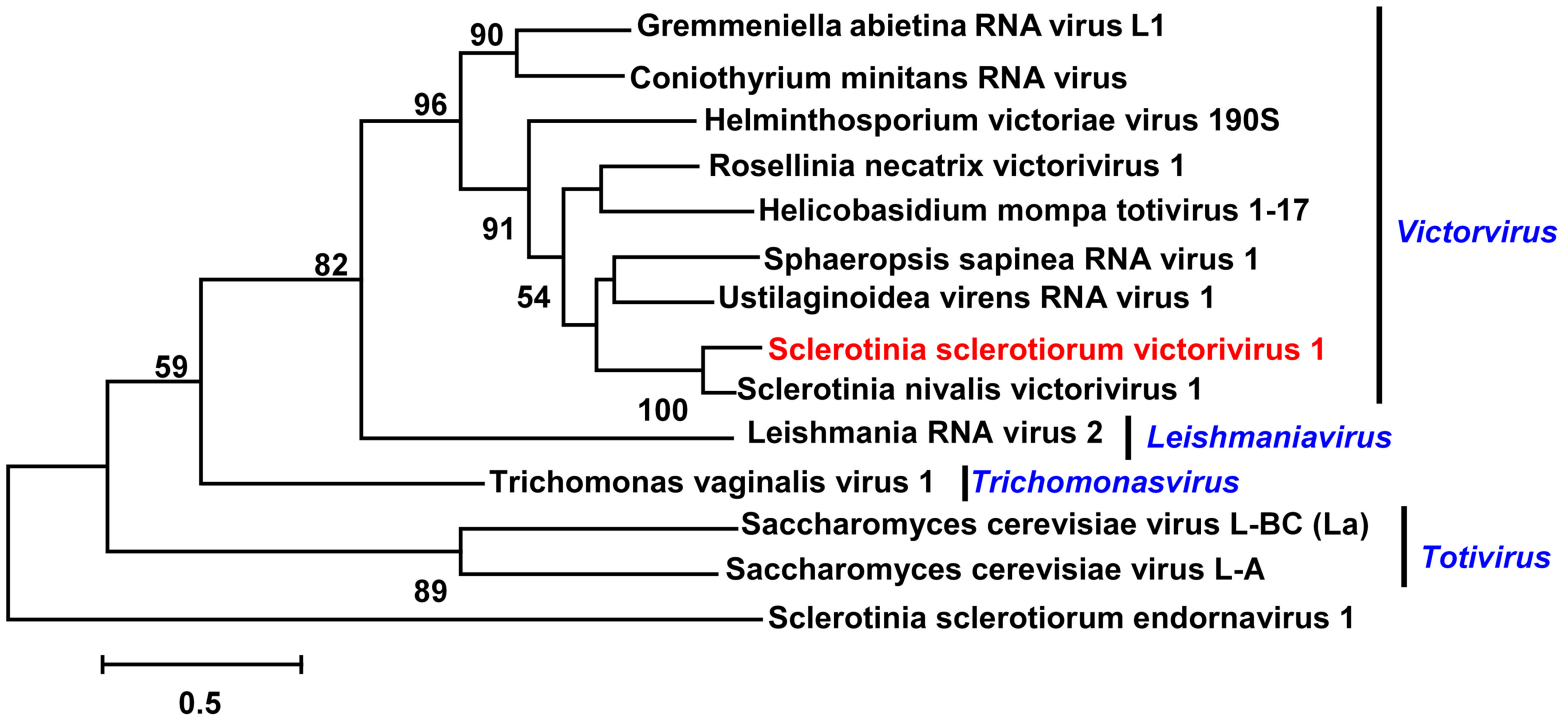
Phylogenetic analysis of the putative virus in Family *Totiviridae*. Phylogenetic analysis of the putative Sclerotinia sclerotiorum victorivirus 1 with other selected viruses in Family *Totiviridae* based on viral RdRp amino acid sequences. Branch lengths are scaled to the expected underlying number of amino acid substitutions per site. If not specifically addressed, the alignments of RdRp amino acid sequences were performed by ClustalW, and phylogenetic tree was constructed using MEGA7.0.18, and selected viruses are listed in Table S4. Viruses marked with red color are found in Australian isolates of *Sclerotinia sclerotiorum*. Sclerotinia sclerotiorum endornavirus 1 was as the outgroup.

The family *Totiviridae* encompasses a broad range of viruses characterized by isometric virions, about 40 nm in diameter, each containing a non-segmented dsRNA genome coding in most cases for only a capsid protein (CP) and an RNA-dependent RNA polymerase (RdRp) (Ghabrial and Nibert, 2009). There were four contigs, namely contig 15405, contig 18615, contig 17728, and contig 9550, where sequences likely represent the viral genome of a novel Totivirus. The gap between contig 15405 and contig 18615 was filled by RT-PCR, and was assembled as RNA sequence 1 of a single viral sequence; the gap between contig 17728 and contig 9550 also was filled and was assembled as RNA2. Both of these RNA sequences had a large Open Reading Frame (ORF). The ORF of RNA 1 encoded a putative protein with 346 aa; while the ORF of RNA sequence 2 encoded a putative protein with 291 aa. The results of Blastp analysis revealed that the putative protein encoded by RNA 1 was similar to the RdRp of Sclerotinia nivalis victorivirus (Wu et al., 2016) with 64% identity match (Table 1), and the putative protein of ORF of RNA 2 was similar to the coat protein (CP) of this victorivirus at 78% identity match. These results suggest that the RNAs of contig 15405, contig 18615, contig 17728, and contig 9550 were likely to be either dsRNA genome or transcripts of a victorivirus that belongs to the Family *Totiviridae*. We have named this new virus Sclerotinia sclerotiorum victorivirus 1 (SsVRV1). Phylogenic analysis with the RdRp of SsVRV1 and other selected viruses showed that SsVRV 1 clustered with members of the genus *Victorivirus* (Figure 2).

### Two predicted novel viruses in the family *Partitiviridae*

Viruses in the family *Partitiviridae* have bi-segmented genomes, about 1,400–2,400 bp in length and encompass one large ORF per segment (Ghabrial et al., 2015). Generally, the larger segment (dsRNA1) encodes the RdRp and the smaller segment (dsRNA2) encodes the CP. These two genome segments are packaged into separate virus particles. Previously, some partitiviruses have been discovered in *S. sclerotiorum*, namely Sclerotinia sclerotiorum partitivirus 1 (Xiao et al., 2014), and Sclerotinia sclerotiorum partitivirus S (Liu et al., 2012).

Two putative viruses that belonged to the Family *Partitiviridae* were identified in this work. The contig 661 was 1,547 nt, with one incomplete ORF which encoded 432 aa. The Blastp analysis result showed this putative protein to be similar to the CP of Botryotinia fuckeliana partitivirus 1 with 44% identity (Table 1). Thus, the contig 661 represented a partial sequence of a partitivirus, and we named this virus as Sclerotinia sclerotiorum partitivirus 2 (SsPV2).

The RNA sequence of contig 904 was 1,769 nt, with one complete ORF which encoded a 538 aa protein. The Blastp analysis result showed that this putative protein had 87% identity to the RdRp of Verticillium albo-atrum partitivirus 1, a member of the *Gammapartitivirus* genus (Table 1). Thus, the contig 904 represented a new partitivirus of *S. sclerotinia*, and we named Sclerotinia sclerotiorum partitivirus 3 (SsPV3); and it is likely that SsPV3 is a strain of *V. albo-atrum* partitivirus 1.

**Figure 3:**
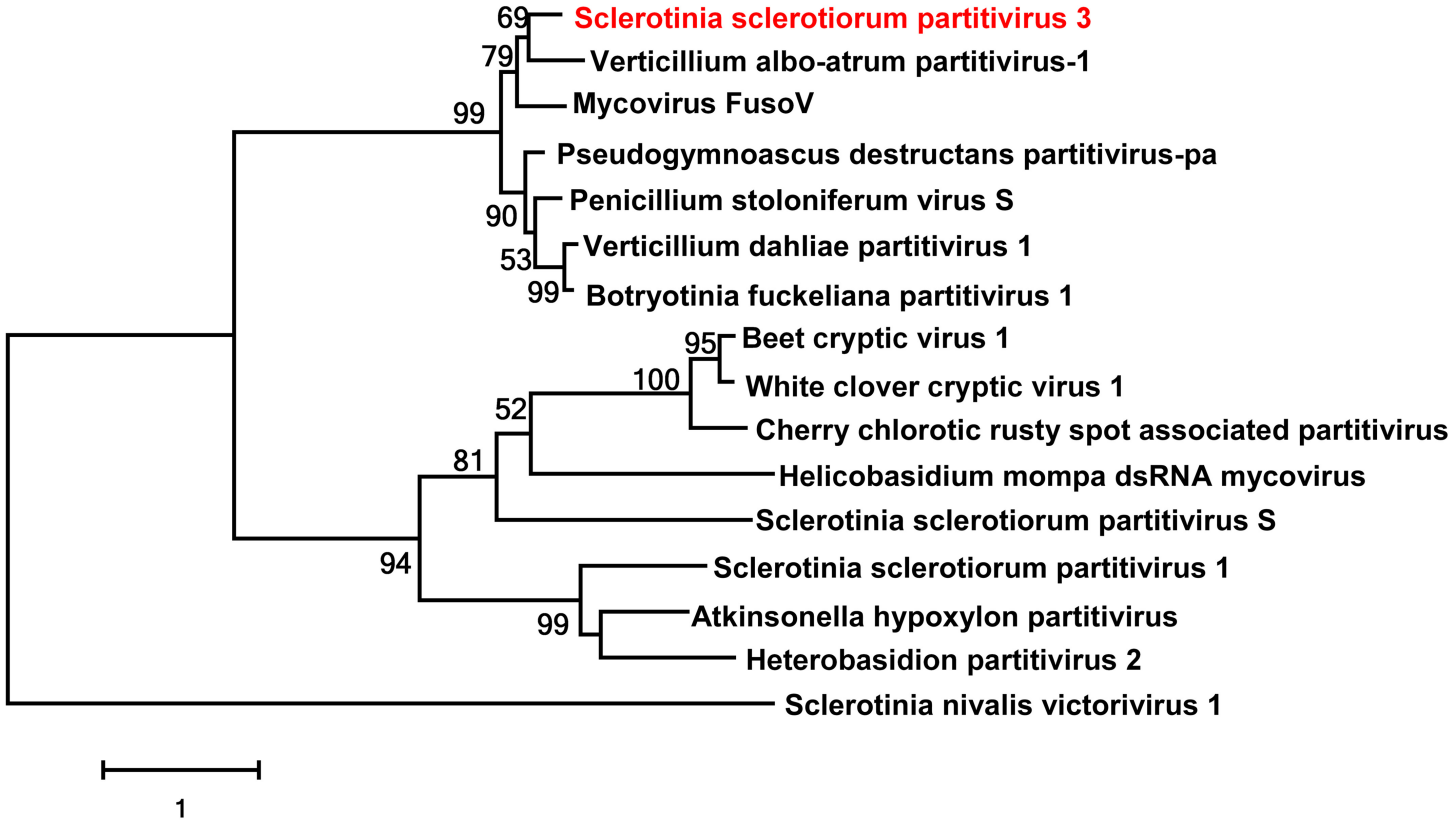
Phylogenetic analysis of Sclerotinia sclerotiorum partitivirus. Phylogenetic analysis of *Sclerotinia sclerotiorum* partitivirus 3 and other selected viruses based on the RdRp amino acid sequences. Viruses marked with red color are found in Australian isolates of *Sclerotinia sclerotiorum*. Sclerotinia nivalis victorivirus 1 was as the outgroup.

A phylogenetic analysis for the conserved RdRp domain among SsPV3 and other selected partitiviruses, such as SsPV1, SsPVS, Botryosphaeria dothidea virus 1, Verticillium albo-atrum partitivirus 1, and Pseudogymnoascus destructans virus, was conducted and a phylogenetic tree constructed (Figure 3). This tree showed that *S. sclerotiorum* could host various partitiviruses.

### An isolate of *Botrytis porri* RNA virus 1 determined

*Botrytis porri* RNA virus 1 (BpRV1) is a hypovirulence-associated mycovirus that infects the fungal pathogen *B. porri*, and it represents a novel type of dsRNA virus tentatively assigned to the Family *Botybirnaviridae* by Wu et al. (2012). Previously, two botybirnaviruses, Sclerotinia sclerotiorum botybirnavirus 1 (SsBRV1) and Sclerotinia sclerotiorum botybirnavirus 2 (SsBRV2), have been discovered in *S. sclerotiorum*, but unlike BpRV1, the previously reported *S. sclerotiorum* botybirnaviruses have only limited impact on host's virulence (Liu et al., 2015; Ran et al., 2016).

In this study, three contigs related to BpRV1 were identified. Two RNA sequences, namely contig 1941 and contig 1940, were identified to be most similar to the cap-pol fusion protein of BpRV1; and multi-kmer and multi-library assemblies showed that they were in fact part of the same genome, resulting in a final contig length of 5,779 nt. It had an incomplete ORF that encoded 1,832 aa and Blastp searching showed that this putative protein was almost identical with the cap-pol fusion protein of BpRV1 with 98% identity (Table 1). The RNA sequence of contig 823 was 5,417 nt and it had a complete ORF that encoded 1,725 aa; Blastp searching showed that this putative protein was almost identical to hypothetical protein of reported BpRV1 with 98% identity (Table 1).

These results suggested that BpRV1 also could infect *S. sclerotiorum* naturally. BpRV1 was originally isolated from *B. porri*, and it confers hypovirulence to *B. porri* as well as *B. cinerea* (Wu et al., 2012). Thus, *S. sclerotiorum* is also a natural host of BpRV1, and it is likely that BpRV1 would spread extensively in the fungal host. Furthermore, BpRV1 may confer hypovirulence to *S. sclerotiorum* since the tested strains were abnormal strains of *S. sclerotiorum*. The potential for use of BpRV1 to control *Sclerotinia* diseases is need to be a future research priority.

### Predicted novel dsRNA virus related to *Aspergillus fumigatus* tetramycovirus-1

A tetra-segment dsRNA virus (*Aspergillus fumigatus* tetramycovirus-1, AftV1) has been isolated from *A. fumigatus*, a virus that represents a new type of the dsRNA viruses which are different from chrysoviruses and quadripartite dsRNA viruses. AftV1 is considered as an intermediate between dsRNA and ssRNA viruses as well as between capsidless and encapsidated viruses (Kanhayuwa et al., 2015). Recent research showed that *B. dothidea* RNA virus 1 (BdRV1) that infects *B. dothidea* and *Colletotrichum camelliae* filamentous virus 1 that infects *C. camelliae* were phylogentically related to AftV1 but have five dsRNA segments and eight dsRNA segments, respectively (Zhai et al., 2016; Jia et al., 2017).

In the current study, three contigs were associated with AftV1. A contig 889 with 2,091 nt contained an incomplete ORF which encoded a putative protein with 614 aa. Blastp search showed this putative protein was most similar to methyltransferase of AftV1 with 30% identity (Table 1). Contig 6913 with 2,612 nt contained a complete ORF encoding a putative protein with 799 aa; Blastp search showed this putative protein was most similar to the RdRp of AftV1 with 45% identity (Table 1). The RNA sequence of contig 1672 was 2,282 nt and had one complete ORF which encoded 713 aa. Blastp analysis result showed this putative protein to be similar to the hypothetical protein of AftV1 with 28% identity (Table 1). In addition, these putative proteins were somewhat related to Beauveria bassiana polymycovirus 1 (BbPmV-1) (Kotta-Loizou and Coutts, 2017), suggesting that an AftRV1-like virus could infect *S. sclerotiorum*, and we named it as Sclerotinia sclerotiorum tetramycovirus 1 (SstRV1).

**Figure 4:**
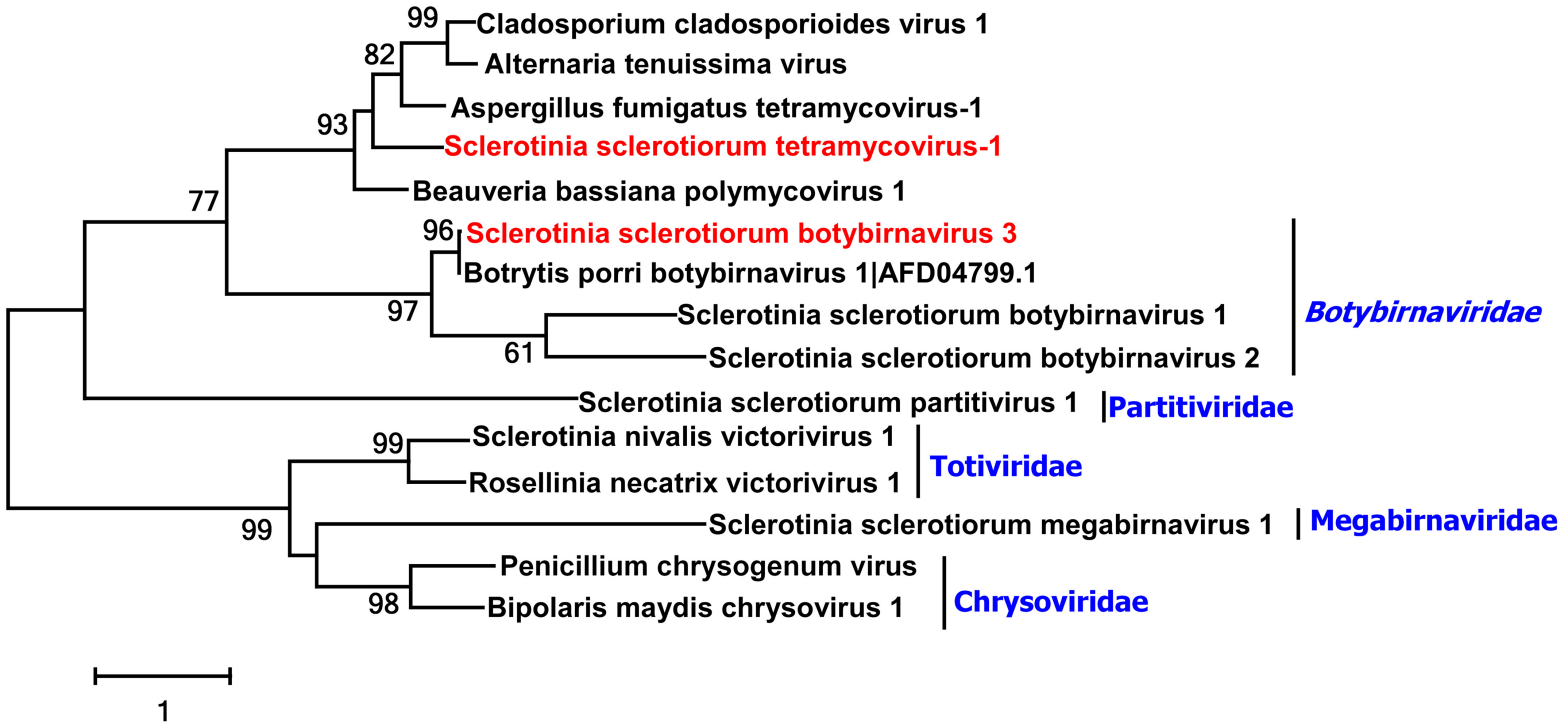
Phylogenetic analysis of two unclassified viruses. Phylogenetic analysis of Sclerotinia sclerotiorum tetramycovirus-1 and Sclerotinia sclerotiorum botybirnavirus 3 with other selected viruses based on the RdRp protein amino acid sequences. Viruses marked with red color are found in Australian isolates of *Sclerotinia sclerotiorum*.

A phylogenetic analysis for the putative RdRp of SstRV1, AftV1, BbPmV-1, and other selected viruses was conducted and a phylogenetic tree constructed (Figure 4). From this tree, we found that SstRV1 was closely related to AftRV 1. These results suggest that terymyco-like viruses are likely widespred in fungi.

### Two predicted novel viruses plus SsHV1 in the family *Hypoviridae*

Viruses in family *Hypoviridae* typically have (+) ssRNA genomes of 9 to 13 kb with one or two ORFs (Ghabrial et al., 2015). These include some hypoviruses discovered in *S. sclerotiorum*, namely Sclerotinia sclerotiorum hypovirus 1 (SsHV1) (Xie et al., 2011), and SsHV2 (Hu et al., 2014; Khalifa et al., 2016). Recently, recombinant strain of Sclerotinia sclerotiorum hypovirus 2 was found in North America, viz. Sclerotinia sclerotiorum hypovirus 2 Lactuca (SsHV2L) (Marzano et al., 2016).

Three RNA sequences showed similarity to members of the family *Hypoviridae*. These included contig 259 that was 10,205 nt, and contained one large ORF encoding a putative polyprotein with 2,942 aa, and was similar to members of the family *Hypoviridae*. This putative polyprotein contained RdRp, peptidase and helicase domains. The amino acid sequence of the predicted protein was most similar to the RdRp gene of SsHV1 with 99% identity. Thus, there is at least one strain of SsHV1 present in Australia.

The contig 1373 was 4,624 nt, contained one incomplete ORF encoding putative polyprotein with 1,243 aa, and the putative polyprotein contained RdRp and helicase domains. Blastp analysis showed that the putative protein was most similar to that of SsHV1 with 76% identity. Thus, the contig 1373 represented a novel SsHV1-related hypovirus, and we named this new virus Sclerotinia sclerotiorum hypovirus 3 (SsHV3).

**Figure 5:**
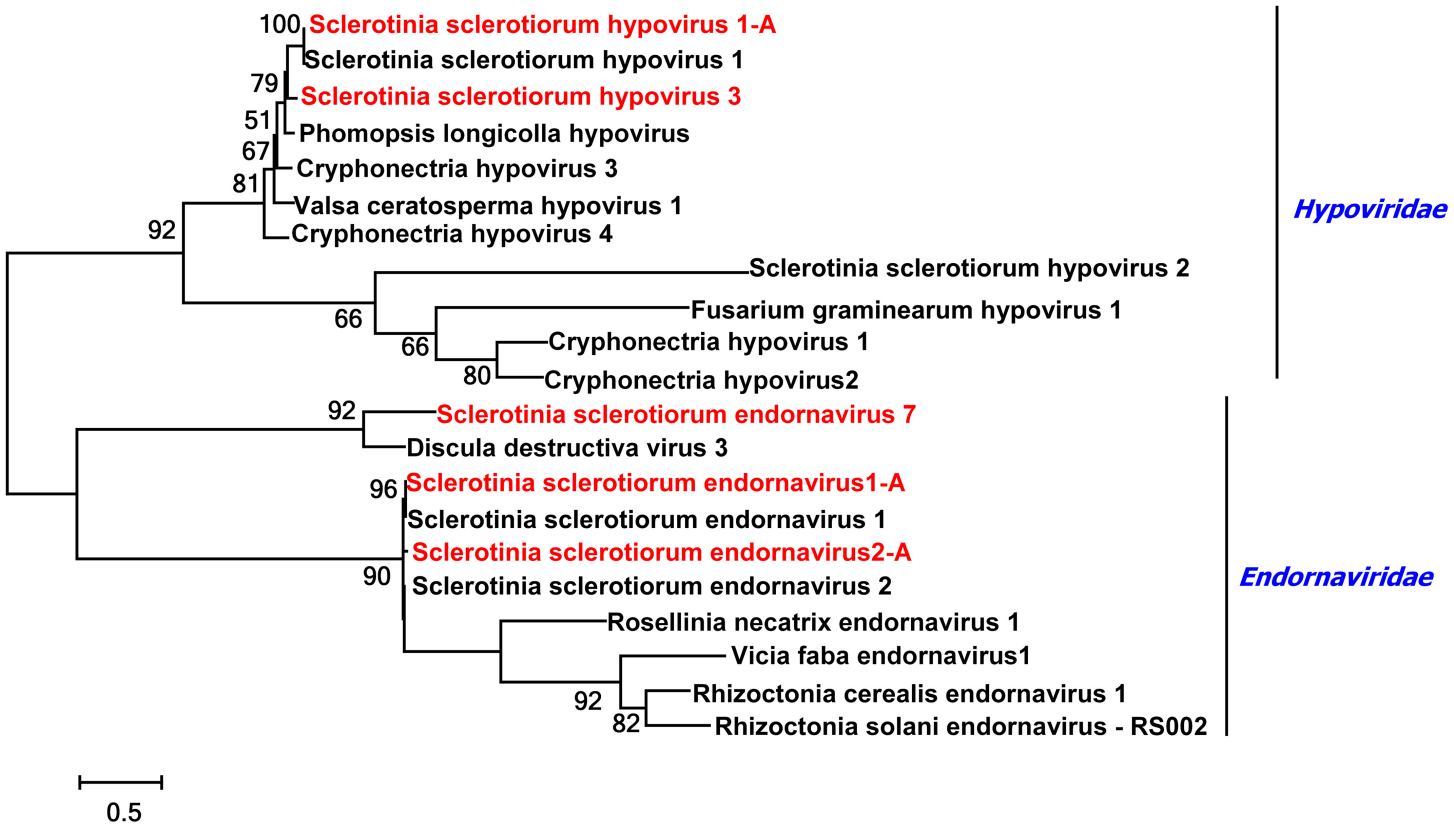
Phylogenetic analysis of viruses in the Family *Endornaviridae* and *Hypoviridae*. Phylogenetic analysis of viruses in the Family *Endornaviridae* and viruses in the Family *Hypoviridae* based on multiple alignments of full-length RdRp amino acid sequences. Viruses marked with red color are found in Australian isolates of *Scleorotinia sclerotiorum*.

Contig 1333 and contig 1332 overlapped and had almost identical sequence as analyzed by multi-kmer and multi-library assemblies. Contig 1333 was 1,665 nt, contained one incomplete ORF encoding putative polyproteins with 432 aa, and the putative polyprotein contained a RdRp domain. The putative protein was the most similar to Cryphonectria hypovirus 3 with 61% identity. Clearly, contig 1333 and contig 1332 represented a novel virus, and we named it Sclerotinia sclerotiorum hypovirus 4 (SsHV4) (Table 1). The phylogenetic tree of the SsHV1 and SsHV3 was constructed with family *Hypoviridae* (Figure 5).

### Four predicted novel viruses plus two characterized viruses in the family *Endornaviridae*

Viruses in the family *Endornaviridae* have linear ssRNA genomes that range in length from about 10 kb to more than 17 kb, and contain a single large ORF. Each characterized genome encodes a single long polyprotein that includes conserved domains of typical viral RNA helicases and RdRps (Ghabrial et al., 2015). Some endornaviruses have been discovered in *S. sclerotiorum*, namely Sclerotinia sclerotiorum betaendornavirus 1 (SsEV1) (Khalifa and Pearson, 2014a). Further, recently representative strains of Sclerotinia sclerotiorum endornaviruses were found in New Zealand, Sclerotinia sclerotiorum betaendornavirus 1 Lactuca (SsEV1L), and Sclerotinia sclerotiorum betaendornavirus 1 2 (SsEV2-IL) (Marzano et al., 2016).

Seven RNA sequences showed similarity to members of the family *Endornaviridae*. These included contig 7442 that was 2,509 nt, had one large incomplete ORF encoding a putative polyprotein with 790 aa, and this putative protein had RdRp and helicase domains. The predicted RdRp was similar to that of SsEV 1 with 98% identity. Contig 3829 was 7,517 nt, had one large incomplete ORF encoding a putative polyprotein with 2,438 aa, and this putative protein had RdRp and helicase domains. The predicted RdRp was similar to that of SsEV2 with 95% identity. Thus, these two contigs represented strains of SsEV1 and SsEV2, respectively.

Contig 1364 was 1,617 nt, contained one incomplete ORF encoding putative polyprotein with 565 aa. Blastp analysis showed that this putative protein was most similar to the RdRp of Rhizoctonia cerealis endornavirus 1 (RcEV1) with 49% identity. Thus, contig 1364 represented a novel endornavirus that we named Sclerotinia sclerotiorum endornavirus 3 (SsEV3).

Contig 282 was 11,034 nt and contained one complete ORF. It encoded a putative polyprotein with 2,867 aa. The predicted amino acid sequence of this protein was similar to the protein ORFA+B of Vicia faba endornavirus with 24% identity. This suggests that contig 282 represents a novel endornavirus, and we have named it Sclerotinia sclerotiorum endornavirus 4 (SsEV4).

There are two contigs, contig 6278 was 617 nt and contig 15215 was 302 nt, both their encoded proteins were similar to the polyproteins of viruses in the *Endornaviridae*. In order to confirm they were representing two viruses or one virus, the primers pairs were designed according to these two sequences of the contigs and amplification and sequencing analysis. The results indicated that these two contigs could be linked together. Thus, these two contigs represented one virus. The combined sequence length was 2,757 nt and contained one incomplete ORF encoding a putative polyprotein with 904 aa (Table S6). The predicted amino acid sequence of this protein was similar to the polyprotein of *R. solani* endornavirus—RS002 (RsEV-RS002) with 39% identity. This suggests that the sequence represents a novel endornavirus, and we named it Sclerotinia sclerotiorum endornavirus 5 (SsEV5).

Contig 14516 was 551 nt and the predicted amino acid of this contig was the most similar to the RdRp of *Discula destructiva* virus 3 with 49% identity. Thus, contig 14516 likely represents another novel endornavirus, and we named it Sclerotinia sclerotiorum endornavirus 6 (SsEV6).

A phylogenetic analysis for the conserved RdRp domain among SsEV1, SsEV2, SsEV3, SsEV5, and other selected endornaviruses, such as RcEV1, RsEV-RS002 and Helicobasidium mompa endornavirus 1 was conducted and a phylogenetic tree constructed (Figure 5). This tree highlighted that *S. sclerotiorum* hosts various endornaviruses.

### One novel plus one characterized ourmiaviruses

The genomes of ourmiaviruses are linear; contain three RNA segments with 2.8, 1.0, and 0.9 kb size. The largest RNA segment encodes the viral replicase (Donaire et al., 2016; Turina et al., 2017) Two ourmiaviruses have previously been discovered in *S. sclerotiorum*, viz. Sclerotinia sclerotiorum ourmia-like virus 1(SsOLV1) and Sclerotinia sclerotiorum ourmia-like virus 2(SsOLV2) (Marzano et al., 2016). Recently there was a report of an ourmiavirus in *Botrytis* (Donaire et al., 2016). In our dataset there are some contigs that are related to the RdRp amino acid sequence of SsOLV1. We predicted there was an ourmiavirus in tested isolates of *S. sclerotiorum*, and it is likely a new strain of SsOLV1 (SsOLV1-A).

Four contigs, contig 30, contig 1694, contig 4006, and contig 5114, were related to the RdRp amino acid sequence of Sclerotinia sclerotiorum ourmia-like virus 2. These contigs could be assembled into one sequence which was 2,442 nt with an incomplete ORF encoding a putative protein with 602 aa. Blastp analysis of this putative protein revealed that it was similar to the RdRp protein of Sclerotinia sclerotiorum ourmia-like virus 2 with 37% identity, namely Sclerotinia sclerotiorum ourmia-like virus 3(SsOLV3).

### Ten predicted novel mitoviruses plus 15 characterized mitoviruses in the family *Narnaviridae*

Mitoviruses typically have a monopartite and linear genome, the size of genome approximating 2.5–2.9 kb and containing a single ORF encoding only the RdRp. Mitoviruses do not have true virion or structural proteins (Hillman and Cai, 2013). Many mitoviruses have been discovered in *S. sclerotiorum*, and characterized mitoviruses include Sclerotinia sclerotiorum mitovirus 1,−2,−3,−4,−5,−6, and−7 (SsMV1,−2,−3,−4, −5,−6,−7) (Xie and Ghabrial, 2012; Khalifa and Pearson, 2014b; Xu et al., 2015; Ran et al., 2016). Many novel mitoviruses have been found in *S. sclerotiorum* and as follows: SsMV7-IL1, SsMV8, SsMV9, SsMV10, SsMV11, SsMV12, SsMV13, SsMV14, SsMV15, SsMV16, SsMV17, SsMV18, SsMV22, and SsMV23 (Marzano et al., 2016).

In the current study, a total of 116 contigs showed similarity to mitoviruses in the family *Narnaviridae*. Some sequences showed high similarity to previously reported Sclerotinia sclerotiorum mitoviruses. Virus sequences of SsMV 1, SsMV 1/HC025,−2,−4,−5,−6,−7,−8,−9,−10,−11,−12,−14,−17, and −19 were determined, indicating that these 15 mitoviruses or strains of these viruses were present in the tested *S. sclerotiorum* isolates (Table 1 and Table S4).

The current study demonstrated the presence of 10 novel mitoviruses, as follows:

Contig 24 was 293 nt, with a predicted amino acid sequence most similar to the RdRp amino acid sequence of SsMV7 with 62% identity. With a siRNA sequencing dataset derived from the same RNA samples (data not showed) and RT-PCR amplification, we extended the viral RNA sequence to 1,243 nt (Table S6). The sequence contained an incompleted ORF encoding a putative protein for which amino acid sequence was most similar to the RdRp amino acid sequence of SsMV7 with 75% identity. We named the novel mitovirus Sclerotinia sclerotiorum mitovirus 24 (SsMV24).

Contig 15 was 1,447 nt, with a predicted amino acid sequence most similar to the RdRp amino acid sequence of SsMV5 with 62% identity. We named it Sclerotinia sclerotiorum mitovirus 25 (SsMV25).

There were eight contigs, contigs 634, 1286, 635, 896, 649, 1377, 2035, and 87, similar to the sequence of Rhizoctonia solani mitovirus 10 (RsMV10), that could be assembled into a single sequence, the length of the sequence was 1,469 nt. The assembled contig was further extended with RT-PCR amplification, and the length of extended contig was 1,992 nt (Table S6), The predicted amino acid sequence is most similar to the RdRp amino acid sequence of RsMV10 with 49% identity, we named it Sclerotinia sclerotiorum mitovirus 26 (SsMV26).

Contig 958 was 2917 nt, with a predicted amino acid sequence most similar to the RdRp amino acid sequence of Ophiostoma mitovirus 1a with 47% identity. We named it Sclerotinia sclerotiorum mitovirus 27 (SsMV27).

There were two contigs similar to *B. cinerea* mitovirus 3, and these contigs could not be assembled into a single sequence. Contig 867 was 2,721 nt, with the predicted amino acid sequence most similar to the RdRp amino acid of Botrytis cinerea mitovirus 3 at 37% identity. We named it Sclerotinia sclerotiorum mitovirus 28 (SsMV28). Contig 536 was 3,262nt, with the predicted amino acid sequence most similar to the RdRp amino acid of Botrytis cinerea mitovirus 3 at 38% identity. We named it Sclerotinia sclerotiorum mitovirus 29 (SsMV29).

Seven contigs (contigs 22, 28, 39, 49, 50, 59, and 119) were similar to Macrophomina phaseolina mitovirus 1 (MpMV1). Contig 59 was 2,536 nt in length and encoded a putative protein most similar to the RdRP of MpMV1 at 83% identity. We named it Sclerotinia sclerotiorum mitovirus 30 (SsMV30), and it was likely to be a strain of MpMV1. Other contigs were shorter and were similar to the RdRP of MpMV1, and these likely represent different isolates of SsMV30.

Two contigs were similar to the sequence of SsMV2, contigs 31 and 24, and these contigs could be assemblyed into a single sequence. The length of the sequence was 1,696 nt, the putative protein of the sequence was most similar to the RdRp amino acid sequence of SsMV2 with 60% identity. We named it Sclerotinia sclerotiorum mitovirus 31(SsMV31).

Contig 1865 was 2,473 nt, and had a predicted amino acid sequence most similar to the RdRp amino acid sequence of SsMV6 with 70% identity. We named it Sclerotinia sclerotiorum mitovirus 32 (SsMV32). Contig 90 was 2,661 nt, and had a predicted amino acid sequence most similar to the RdRp amino acid sequence of Sclerotinia sclerotiorum mitovirus 19 with 45% identity. Thus, we named it Sclerotinia sclerotiorum mitovirus 33 (SsMV33).

**Figure 6:**
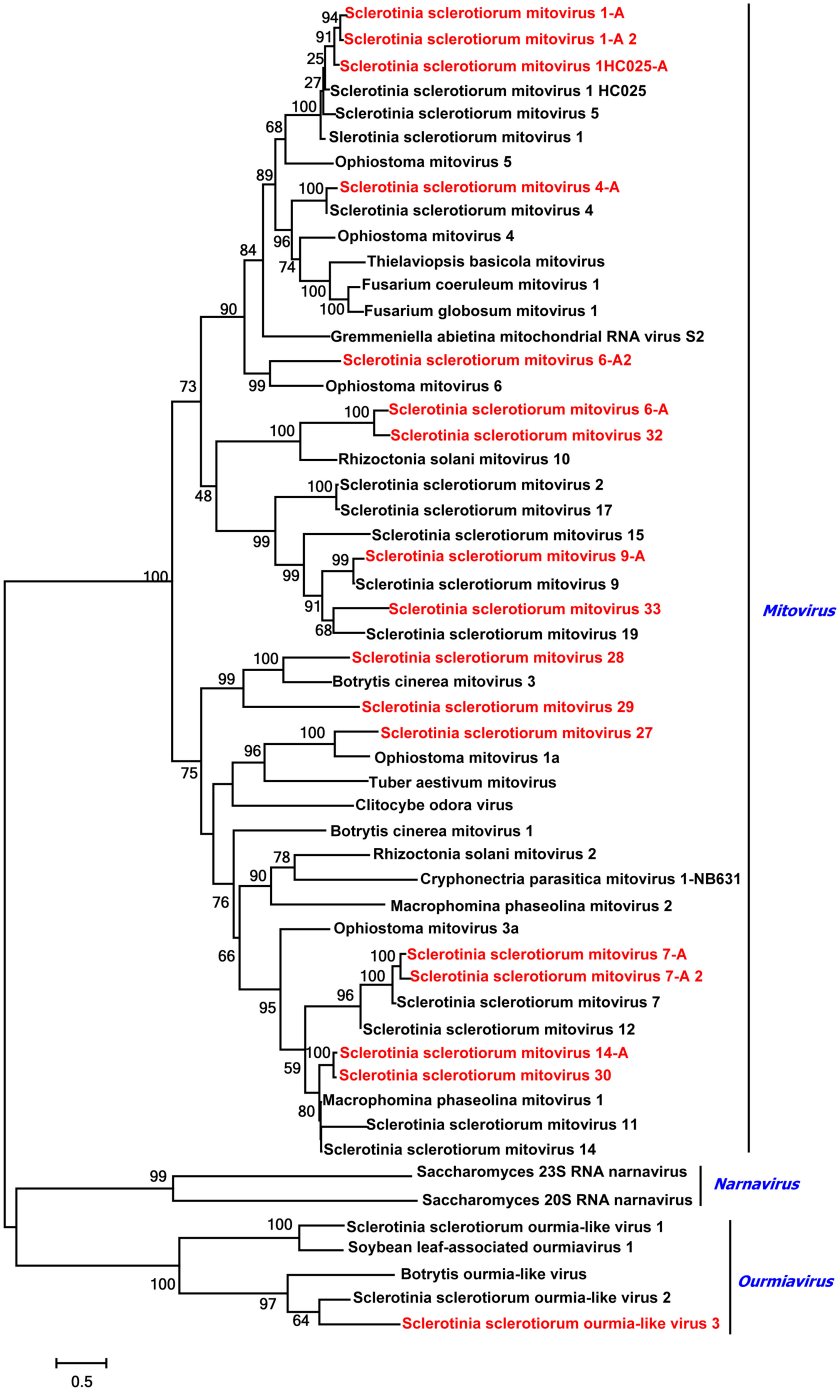
Phylogenetic analysis of *Mitoviruses* and *Ourmiavirus*. Phylogenetic relationships of mitoviruses and ourmiavirus that infect *Sclerotinia sclerotiorum* and other selected viruses. Multi alignments are based on the virus's full-length RdRp amino acid sequences. Viruses marked with red color are found in Australian isolates of *Sclerotinia sclerotiorum*.

A phylogenetic analysis based on multiple alignments of full-length RdRp amino acid sequences of SsMV25, SsMV26, SsMV27, SsMV28, SsMV29, SsMV30, SsMV32, SsMV33, SsOLV3, and other selected viruses was conducted, and results grouped the sequences into two well-supported distinct clades where sequences were most similar to previously identified mitoviruses from the same fungal host (Figure 6). Previously, several mitoviruses were found to infect phylogenetically distant fungi (Melzer et al., 2005; Wu et al., 2010), thus the isolation of mitoviruses that infect phylogentically distantly related fungi was not unexpected.

### Two novel umbra-like viruses

**Figure 7:**
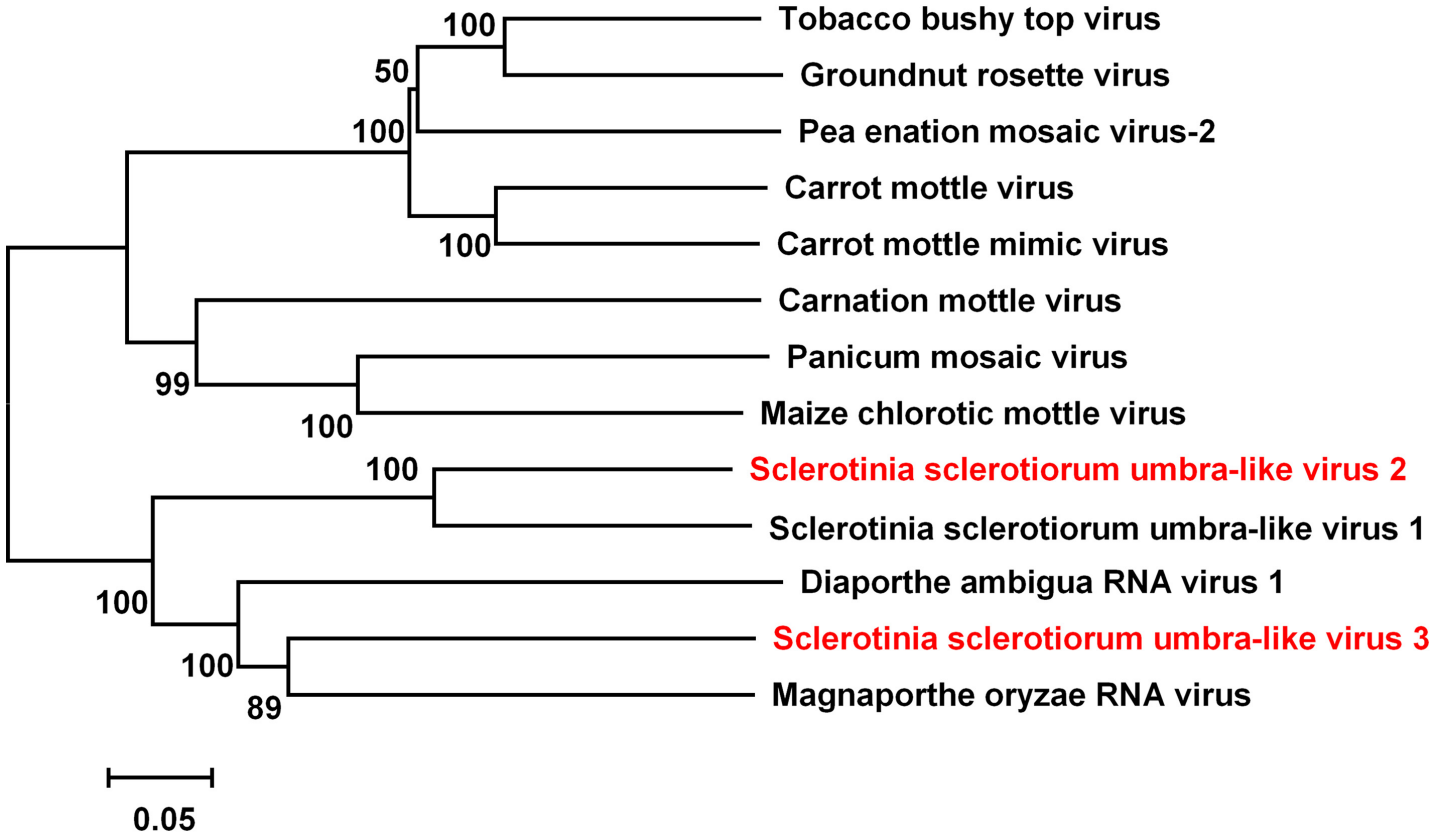
Phylogenetic analysis of virus in the Family *Tombusviridae*. Phylogenetic relationships of putative new virus genomes of *Tombusviridae* virus. Viruses marked with red color are found in Australian isolates of *Sclerotinia sclerotiorum*.

Members in the Family *Tombusviridae* have genomes composed of one or two of 3.7–4.8 kb ssRNA segments with up to six ORFs. A mycovirus in the family *Tombusviridae* had been reported earlier, Sclerotinia sclerotiorum umbra-like virus 1 (SsULV1) (Marzano et al., 2016). The sequence of contig 224 was 4,170 nt with an incomplete ORF encoding a putative protein with 1331 aa. Blastp analysis of this putative protein revealed that it was similar to the RdRp protein of SsULV1 with 49% identity. Contig 534 sequence was 3,925 nt with an incomplete ORF encoding putative proteins with 489 aa. Blastp analysis showed that this protein was most similar to the RdRp of Magnaporthe oryzae RNA virus at 37% identity. These results indicated that two umbra-like viruses, different from SsULV1, are present in tested isolates of *S. sclerotiorum*. We named these as Sclerotinia sclerotiorum umbra-like virus 2 (SsULV2) and Sclerotinia sclerotiorum umbra-like virus 3 (SsULV3). When a phylogenetic tree was constructed based on multiple alignments of full-length RdRp amino acid sequences (Figure 7), SsULV2 was in the same clade with SsULV1, while SsULV3 was distant from both SsULV1 and SsULV2.

### Five predicted novel viruses in the order *Tymovirales*

The order *Tymovirales* is composed of four approved families (*Alpha-, Beta-, Gamma-flexiviridae*, and *Tymoviridae*), and all members contain a linear genome from 5.9–9.0 kb in length (Martelli et al., 2007). Members in the order *Tymovirales* have diverse host ranges, which is also a characteristic feature of individual genera. Members of *Betaflexiviridae* and *Tymoviridae* have been shown to infect plants, and a single member of *Gammaflexiviridae* only infects a filamentous fungus, whereas members of *Alphaflexiviridae* infect both plants and fungi in nature (Martelli et al., 2007). Some viruses in *Tymovirales* that infect *S. sclerotiorum* have been reported, such as Sclerotinia sclerotiorum debilitation-associated RNA virus (SsDRV) (Xie et al., 2006) and Sclerotinia sclerotiorum deltaflexivirus 1 (SsDFV1) (Li et al., 2016). Here we report some contigs related to *Tymoviridae* (Table 1).

Two contigs, contigs 3545 and 13690, were similar to the sequence of Nectarine Virus M. These contigs could be assembled into a single sequence. The length of the sequence was 1,575 nt, and contained one incomplete ORF encoding putative proteins with 510 aa. The predicted amino acid sequence was most similar to Nectarine Virus M with 32% identity. Nectarine virus M belongs to unclassified *Tymoviridae*, and we named this novel virus as Sclerotinia sclerotiorum tymo-like RNA virus 2 (SsTLRV 2).

Contig 10849 was 964 nt, with a putative incomplete ORF encoding a protein most similar to Turnip yellow mosaic virus with 41% identity. We named this new mycovirus as Sclerotinia sclerotiorum tymo-like RNA virus 3 (SsTLRV 3).

Contig 11935 was 2,926 nt, contained one complete ORF encoding putative proteins with 825 aa, and had a predicted amino acid sequence most similar to Poinsettia mosaic virus with 27% identity. *Poinsettia mosaic virus* is an unassigned species in the family *Tymoviridae*. We named this new mycovirus as Sclerotinia sclerotiorum tymo-like RNA virus 4 (SsTLRV4).

Contig 14854 was 796 nt, with a predicted amino acid sequence most similar to *Fusarium graminearum* mycotymovirus 1 (FgMTV1)with 33% identity. FgMTV1 belongs to unclassified *Tymoviridae*. We named this new mycovirus as Sclerotinia sclerotiorum tymo-like RNA virus 5 (SsTLRV5).

There were five contigs that were related to *F. graminearum* deltaflexivirus 1(FgDFV1). Three contigs, 9241, 5512, and 5513, could be assembled into a single sequence. The length of the sequence was 2,307 nt and contained one incomplete ORF encoding putative proteins with 708 aa. The predicted amino acid sequence was most similar to FgDFV1 with 43% identity. The other two contigs, 13466 and 15254, also could be assembled into a single sequence. The length of the sequence was 1,948 nt and contained one incomplete ORF encoding putative proteins with 592 aa. The predicted amino acid sequence was most similar to FgDFV1 with 42% identity. In addition, both sequences were similar to the hypothetical protein A4X09_g7752 of the fungus *Tilletia walkeri*. We named these contigs as Sclerotinia sclerotiorum tymo-like RNA virus 6 (SsTLRV6), another new virus, and the two sequences represented different strains of this virus.

### Four novel viruses plus four characterized viruses related to members of the *Mononegavirales*

The *Mononegavirales* includes five families: *Bornaviridae, Nyamiviridae, Rhabdoviridae, Ophioviridae*, and *Paramyxoviridae*. Recently, the ICTV has agreed to a new family, *Mymonaviridae*, within the *mononegavirales*, and viruses within this most recent family of the *mononegavirales* are (−)ssRNA viruses known to infect fungi (Afonso et al., 2016). Some viruses in the *Mymonaviridae* have been reported in *S. sclerotiorum*, including Sclerotinia sclerotiorum negative-stranded RNA virus 1 (SsNSRV1) (Liu, 2014), Sclerotinia sclerotiorum negative-stranded RNA virus 2 (SsNSRV2), Sclerotinia sclerotiorum negative-stranded RNA virus 3 (SsNSRV3), and Sclerotinia sclerotiorum negative-stranded RNA virus 4 (SsNSRV4) (Marzano et al., 2016). We found eight sequences that showed similarity to negative-strand RNA viruses in the order *Mononegavirales*. Four viruses, represented by four different contigs (contigs 88, 100, 29, and 512), were different strains of known SsNSRV1,−2,−3,−4. Seven contigs, contigs 88, 83, 89, 398, 3470, 4115, and 195 could be assembled into one long sequence which was 7,735 nt, namely Sclerotinia sclerotiorum negative-stranded RNA viruse 1-A (SsNsRV1-A), and it encoded a protein which shared 100% amino acid identity with the amino acid sequence of the RdRp of SsNSRV1. Contig 100 was 9,614 nt, namely Sclerotinia sclerotiorum negative-stranded RNA viruse2-A (SsNsRV2-A), and from this sequence two ORFs were predicted. Blastp analysis showed that the putative RdRp protein shared 93% identity to that of SsNSRV2. The sequence of contig 29 was 7,104 nt, namely Sclerotinia sclerotiorum negative-stranded RNA viruse 3-A (SsNsRV3-A), with two ORFs, RdRp and GP3. The predicted amino acid sequence of RdRp was most similar to SsNsRV 3 at 98% identity. The contig 512 was 9,564 nt, namely Sclerotinia sclerotiorum negative-stranded RNA viruse 4-A (SsNsRV4-A) and had one putative ORF. Blastp analysis showed that the protein was similar to that of SsNsRV4 with 94% identity.

Four contigs represented novel viruses not previously characterized in *S. sclerotiorum*. The sequence of contigs 4241, 89, 579, and 1114 were 4,421, 5,076, 7,819, and 1,219 nt, respectively. These four contigs had incomplete ORFs encoding putative RdRps which shared 32, 28, 37, and 28% identity to those of SsNsRV4, Soybean leaf-associated negative-stranded RNA virus 1, Soybean leaf-associated negative-stranded RNA virus 2, and Soybean leaf-associated negative-stranded RNA virus 3, respectively. Thus, we have demonstrated four additional new negative-stranded RNA viruses in the *S. sclerotiorum* isolates tested, and we named these as Sclerotinia sclerotiorum negative-stranded RNA virus 5,−6,−7,−8.

**Figure 8:**
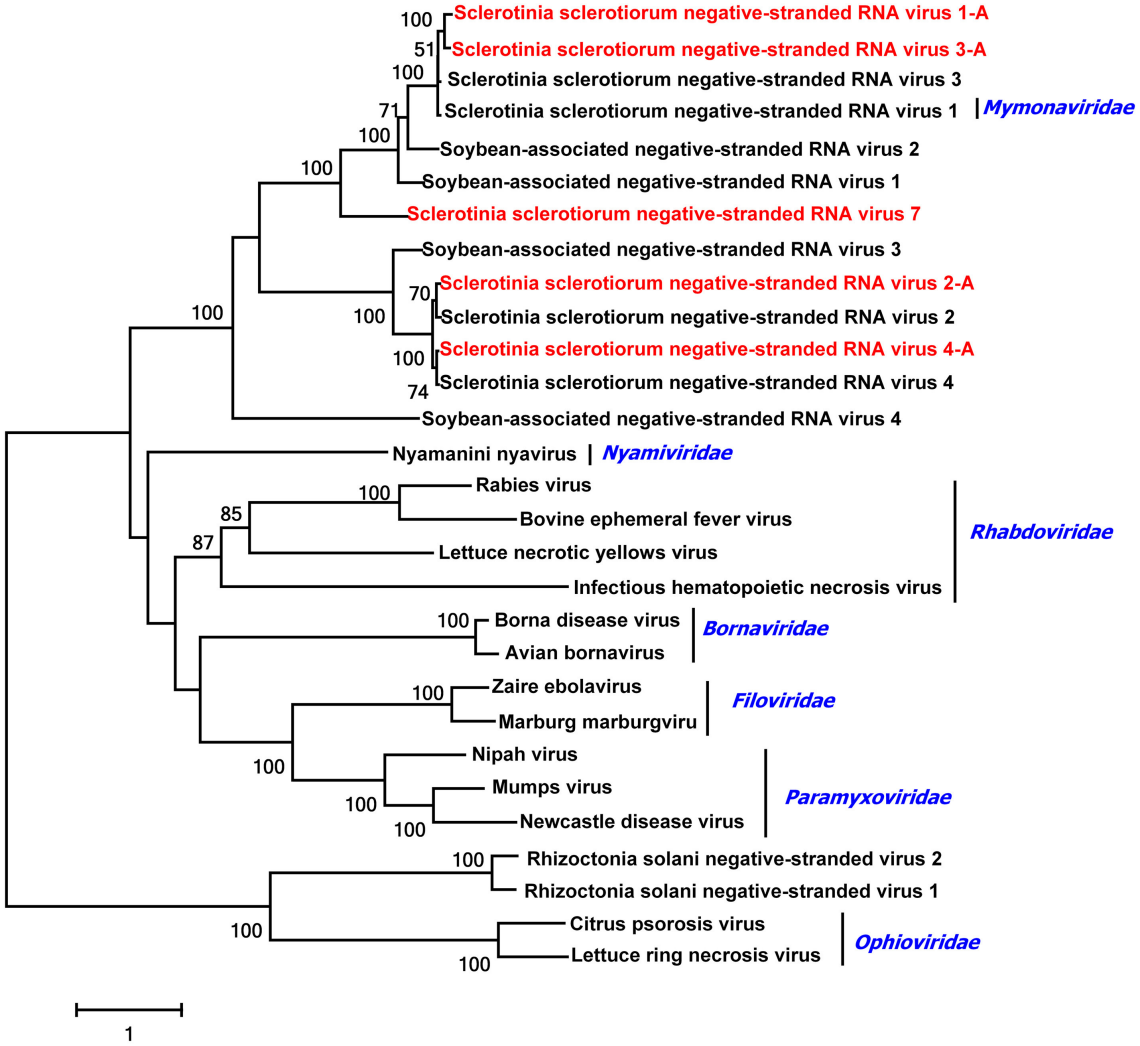
Phylogenetic relationships of putative negative-stranded RNA virus genomes. For the neighbor joining tree, predicted RdRp amino acid sequences were aligned, and phylogenetic trees were constructed. Viruses marked with red color are found in Australian isolates of *Sclerotinia sclerotiorum*.

A phylogenetic analysis based on multiple alignments of full-length RdRp amino acid sequences of these eight viruses and other members of the *Mymonaviridae* was conducted, and the result showed that SsNsRV1-A, SsNsRV2-A, SsNsRV3-A, SsNsRV4-A were novel strains of these known mycoviruses, while SsNsRV 5,−6,−7,−8 were novel negative-stranded viruses (Figure 8).

### *Sclerotinia gemycircular* virus 1 determined in test *S. sclerotiorum* isolates

*Genomoviridae* contains a single genus, *Gemycircularvirus*, which currently has one recognized virus species, *Sclerotinia gemycircular virus 1*, and it is currently the sole representative isolate of the family (Yu et al., 2010, 2013). Contig 2147 was 2,166 nt and had two ORFs encoding putative coat protein with 312 aa and a putative replication-associated protein with 274 aa. The predicted amino acid sequence of replication-associated protein was almost identical to that of SsHADV-1 with 99% identity. This result confirms that SsHADV 1 occurs in Australia, and SsHADV-1 DNA has also been confirmed in Urban River Sediments of Heathcote and Styx Rivers in New Zealand (Kraberger et al., 2013).

## Conclusion

In conclusion, this is the first study to show existence of various mycoviruses in *S. sclerotiorum* in Australia. *S. sclerotiorum* isolates collected from various crops in Australia contained 57 mycoviruses either with RNA or DNA genomes. These 57 mycoviruses could be grouped in ten distinct lineages, namely *Endornaviridae* (four novel mycoviruses), *Genomoviridae, Hypoviridae* (two novel mycoviruses), *Mononegavirales* (four novel mycoviruses), *Narnaviridae* (10 novel mycoviruses), *Partitiviridae* (two novel mycoviruses), *Ourmiavirus* (two novel mycovirus), *Tombusviridae* (two novel mycoviruses), *Totiviridae* (one novel mycovirus), and *Tymovirales* (five novel mycoviruses); and two non-classified mycoviruses lineages (one *B. porri* RNA virus 1, one distantly related to *A. fumigatus* tetramycovirus-1). Twenty-five mitoviruses were determined and mitoviruses were dominant across the isolates tested. These results confirm that mycoviruses that infect *S. sclerotiorum* are widespread in Australia, that many novel mycoviruses occur in Australia, and that some of them are highly likely to offer significant potential for innovative biocontrol of Sclerotinia diseases of crops and vegetable crops both there and elsewhere. Further characterization of these mycoviruses is warranted, both in terms of exploring these novel mycoviruses for biocontrol of Sclerotinia diseases and in enhancing our overall knowledge on viral diversity, taxonomy, ecology, and evolution.

## Author contributions

FM, JX, YF, DJ, and MB: Designed the research and wrote the paper; FM, SC, and JX: Executed the experiments; FM, JJ, JC, QW, and TC: Performed the data and bioinformatics analyses; MB and MY: Supplied materials. All authors read and approved the final manuscript.

### Conflict of interest statement

The authors declare that the research was conducted in the absence of any commercial or financial relationships that could be construed as a potential conflict of interest.
