# Supplementary material for: Virome Characterization of a Collection of S. sclerotiorum from Australia
Source: Front Microbiol. 2018 Jan 11;8:2540. doi: 10.3389/fmicb.2017.02540 (PMC5768646; doi:10.3389/fmicb.2017.02540)
Supplement: Supplementary file 1 [file Table1.DOC]

**Table S1.** Details regarding the non-assembled contigs obtained by RNA_Seq analysis from 84 strains of *Sclerotinia sclerotiorum*

|  | Name of putative virus | unigene_ID | unigene_length(bp) | protein_description | Ident | Genome  Type | *Family/Genus* | E-value |
| --- | --- | --- | --- | --- | --- | --- | --- | --- |
| 1 | Sclerotinia sclerotiorum victorivirus 1 | Ss-AA_clean.1_(paired)_contig_18615 | 432 | coat protein [Sclerotinia nivalis victorivirus 1]1-119 | 64% | dsRNA | *Totiviridae* | 1E-39 |
| 2 |  | Ss-AA_clean.1_(paired)_contig_15405 | 1275 | coat protein [Sclerotinia nivalis victorivirus 1]205-574 | 70% | dsRNA | *Totiviridae* | 0 |
| 3 |  | Ss-AA_clean.1_(paired)_contig_17728 | 549 | RNA dependent RNA polymerase [Sclerotinia nivalis victorivirus 1]155-241 | 49% | dsRNA | *Totiviridae* | 1E-13 |
| 4 |  | Ss-AA_clean.1_(paired)_contig_9550 | 649 | RNA-dependent RNA polymerase [Sclerotinia nivalis victorivirus 1]281-446 | 64% | dsRNA | *Totiviridae* | 3E-88 |
| 5 |  | Ss-AA_clean.1_(paired)_contig_5680 | 815 | RNA dependent RNA polymerase [Sclerotinia nivalis victorivirus 1]669-832 | 74% | dsRNA | *Totiviridae* | 1E-76 |
| 6 | Sclerotinia sclerotiorum partitivirus 2 | Ss-AA_clean.1_(paired)_contig_661 | 1547 | coat protein [Botryotinia fuckeliana partitivirus 1]1-433 | 44% | dsRNA | *Partiviridae* | 2E-92 |
| 7 | Sclerotinia sclerotiorum partitivirus 3 | Ss-AA_clean.1_(paired)_contig_904 | 1769 | RNA-dependent RNA polymerase [Verticillium albo-atrum partitivirus-1]22-539 | 62% | dsRNA | *Partiviridae* | 0 |
| 8 | Sclerotinia sclerotiorum botybirnavirus 2 | Ss-AA_clean.1_(paired)_contig_1941 | 5690 | cap-pol fusion protein [Botrytis porri RNA virus 1]69-1902 | 98% | dsRNA | *unclassfied* | 0 |
| 9 |  | Ss-AA_clean.1_(paired)_contig_1940 | 5779 | cap-pol fusion protein [Botrytis porri RNA virus 1]69-1902 | 97% | dsRNA | *unclassfied* | 0 |
| 10 |  | Ss-AA_clean.1_(paired)_contig_823 | 5417 | hypothetical protein [Botrytis porri RNA virus 1]63-1788 | 99% | dsRNA | *unclassfied* | 0 |
| 11 | Sclerotinia sclerotiorum Aspergillus fumigatus tetramycovirus-1 | Ss-AA_clean.1_(paired)_contig_6913 | 2612 | RNA dependent RNA polymerase [Aspergillus fumigatus tetramycovirus-1]84-761 | 45% | dsRNA | *unclassfied* | 3E-157 |
| 12 |  | Ss-AA_clean.1_(paired)_contig_1672 | 2282 | hypothetical protein [Aspergillus fumigatus tetramycovirus-1]1-694 | 28% | dsRNA | *unclassfied* | 6E-64 |
| 13 |  | Ss-AA_clean.1_(paired)_contig_889 | 2091 | Methyl transferase [Aspergillus fumigatus tetramycovirus-1]10-614 | 30% | dsRNA | *unclassfied* | 9E-48 |
| 14 | Sclerotinia sclerotiorum hypovirus 1-A | Ss-AA_clean.1_(paired)_contig_259 | 10205 | unnamed protein product [Sclerotinia sclerotiorum hypovirus 1] 1-2948 | 99% | +ssRNA | *Hypoviridae* | 0 |
| 15 |  | Ss-AA_clean.1_(paired)_contig_1288 | 2462 | hypothetical protein [Sclerotinia sclerotiorum hypovirus 1] 1-639 | 97% | +ssRNA | *Hypoviridae* | 0 |
| 16 | Sclerotinia sclerotiorum hypovirus 3 | First_Contig182 | 405 | unnamed protein product [Sclerotinia sclerotiorum hypovirus 1]891-965 | 36% | +ssRNA | *Hypoviridae* | 3.00E-06 |
| 17 |  | Ss-AA_clean.1_(paired)_contig_5231 | 573 | unnamed protein product [Sclerotinia sclerotiorum hypovirus 1]1034-1148 | 58% | +ssRNA | *Hypoviridae* | 2E-40 |
| 18 |  | First_Contig146 | 2186 | unnamed protein product [Sclerotinia sclerotiorum hypovirus 1]188-512 | 65% | +ssRNA | *Hypoviridae* | 3.00E-86 |
| 19 |  | Ss-AA_clean.1_(paired)_contig_1373 | 4624 | unnamed protein product [Sclerotinia sclerotiorum hypovirus 1]1703-2945 | 76% | +ssRNA | *Hypoviridae* | 0 |
| 20 | Sclerotinia sclerotiorum hypovirus 4 | Ss-AA_clean.1_(paired)_contig_1333(1332) | 1665 | polyprotein[Cryphonectria hypovirus 3]1077-1630 | 61% | +ssRNA | *Hypoviridae* | 0 |
| 21 | Sclerotinia sclerotiorum endornavirus-1-A | Ss-AA_clean.1_(paired)_contig_6470 | 595 | polyprotein[Sclerotinia sclerotiorum endornavirus-1]1434-1568 | 30% | +ssRNA | *Endornaviridae* | 7E-15 |
| 22 |  | Ss-AA_clean.1_(paired)_contig_13321 | 202 | polyprotein [Sclerotinia sclerotiorum endornavirus-1]475-1265 | 94% | +ssRNA | *Endornaviridae* | 0 |
| 23 |  | Ss-AA_clean.1_(paired)_contig_13322 | 317 |  |  | +ssRNA | *Endornaviridae* |  |
| 24 |  | Ss-AA_clean.1_(paired)_contig_7442 | 2509 |  |  | +ssRNA | *Endornaviridae* |  |
| 25 |  | Ss-AA_clean.1_(paired)_contig_13409 | 845 |  |  | +ssRNA | *Endornaviridae* |  |
| 26 |  | Ss-AA_clean.1_(paired)_contig_7441 | 1160 |  |  | +ssRNA | *Endornaviridae* |  |
| 27 |  | Ss-AA_clean.1_(paired)_contig_4912 | 1377 |  |  | +ssRNA | *Endornaviridae* |  |
| 28 |  | Ss-AA_clean.1_(paired)_contig_7361 | 312 |  |  | +ssRNA | *Endornaviridae* |  |
| 29 |  | Ss-AA_clean.1_(paired)_contig_5232 | 1134 | putative polyprotein [Sclerotinia sclerotiorum endornavirus-1] | 91% | +ssRNA | *Endornaviridae* | 0 |
| 30 |  | Ss-AA_clean.1_(paired)_contig_16357 | 844 |  |  | +ssRNA | *Endornaviridae* |  |
| 31 |  | Ss-AA_clean.1_(paired)_contig_18898 | 256 |  |  | +ssRNA | *Endornaviridae* |  |
| 32 |  | Ss-AA_clean.1_(paired)_contig_16358 | 209 |  |  | +ssRNA | *Endornaviridae* |  |
| 33 |  | Ss-AA_clean.1_(paired)_contig_9741 | 326 |  |  | +ssRNA | *Endornaviridae* |  |
| 34 |  | Ss-AA_clean.1_(paired)_contig_9289 | 255 | putative polyprotein [Sclerotinia sclerotiorum endornavirus-1]1391-1649 | 96% | +ssRNA | *Endornaviridae* | 4E-171 |
| 35 |  | Ss-AA_clean.1_(paired)_contig_15674 | 794 |  |  | +ssRNA | *Endornaviridae* |  |
| 36 |  | Ss-AA_clean.1_(paired)_contig_10459 | 527 | polyprotein [Sclerotinia sclerotiorum endornavirus-1]2614-2992 | 99% | +ssRNA | *Endornaviridae* | 0 |
| 37 |  | Ss-AA_clean.1_(paired)_contig_9333 | 440 |  |  | +ssRNA | *Endornaviridae* |  |
| 38 |  | Ss-AA_clean.1_(paired)_contig_17844 | 228 |  |  | +ssRNA | *Endornaviridae* |  |
| 39 |  | Ss-AA_clean.1_(paired)_contig_14467 | 216 |  |  | +ssRNA | *Endornaviridae* |  |
| 40 |  | First_Contig428 | 888 | polyprotein [Sclerotinia sclerotiorum endornavirus-1] | 100% | +ssRNA | *Endornaviridae* | 1E-36 |
| 41 |  | Ss-AA_clean.1_(paired)_contig_10245 | 571 | polyprotein[Sclerotinia sclerotiorum endornavirus-1]3110-3260 | 100% | +ssRNA | *Endornaviridae* | 1E-97 |
| 42 |  | Ss-AA_clean.1_(paired)_contig_14869 | 447 | polyprotein [Sclerotinia sclerotiorum endornavirus-1] | 100% | +ssRNA | *Endornaviridae* | 1E-29 |
| 43 |  | Ss-AA_clean.1_(paired)_contig_14987 | 321 |  |  | +ssRNA | *Endornaviridae* |  |
| 44 |  | Ss-AA_clean.1_(paired)_contig_2947 | 652 | putative polyprotein [Sclerotinia sclerotiorum endornavirus-1] | 99% | +ssRNA | *Endornaviridae* | 8E-143 |
| 45 | Sclerotinia sclerotiorum endornavirus-2-A | Ss-AA_clean.1_(paired)_contig_3829 | 7517 | polyprotein [Sclerotinia sclerotiorum endornavirus 2]963-3458 | 95% | +ssRNA | *Endornaviridae* | 0 |
| 46 |  | First_Contig294 | 293 | polyprotein [Sclerotinia sclerotiorum endornavirus 2]867-961 | 46% | +ssRNA | *Endornaviridae* | 1E-08 |
| 47 | Sclerotinia sclerotiorum endornavirus 3 | Ss-AA_clean.1_(paired)_contig_1364 | 3284 | polyprotein [Rhizoctonia cerealis endornavirus 1]5289-5745 | 49% | +ssRNA | *Endornaviridae* | 9E-124 |
| 48 | Sclerotinia sclerotiorum endornavirus 4 | Ss-AA_clean.1_(paired)_contig_282 | 11056 | ORFA+B [Vicia faba endornavirus]1040-2246 | 24% | +ssRNA | *Endornaviridae* | 2E-53 |
| 49 | Sclerotinia sclerotiorum endornavirus 5 | Ss-AA_clean.1_(paired)_contig_6278 | 617 | polyprotein, partial [Rhizoctonia solani endornavirus - RS002]1416-1614 | 31% | +ssRNA | *Endornaviridae* | 6E-17 |
| 50 | Sclerotinia sclerotiorum endornavirus 5 | Ss-AA_clean.1_(paired)_contig_15215 | 302 | polyprotein [Helicobasidium mompa endornavirus 1]1213-1310 | 38% | +ssRNA | *Endornaviridae* | 1E-16 |
| 51 | Sclerotinia sclerotiorum endornavirus 6 | Ss-AA_clean.1_(paired)_contig_14516 | 551 | RNA-dependent RNA polymerase [Discula destructiva virus 3]8-171 | 49% | +ssRNA | *Endornaviridae* | 2E-30 |
| 52 | Sclerotinia sclerotiorum mitovirus 1-A | Ss-AA_clean.1_(paired)_contig_192 | 2493 | RNA-dependent RNA polymerases [Sclerotinia sclerotiorum mitovirus 1]1-689 | 83% | +ssRNA | *Narnaviridae* | 0 |
| 53 | Sclerotinia sclerotiorum mitovirus 1-A2 | Ss-AA_clean.1_(paired)_contig_187 | 2086 | RNA-dependent RNA polymerases [Sclerotinia sclerotiorum mitovirus 1]1-689 | 80% | +ssRNA | *Narnaviridae* | 0 |
| 54 | Sclerotinia sclerotiorum mitovirus 1-A | First_Contig50 | 1250 | RNA-dependent RNA polymerase [Sclerotinia sclerotiorum mitovirus 1 ]299-691 | 75% | +ssRNA | *Narnaviridae* | 0 |
| 55 |  | Ss-AA_clean.1_(paired)_contig_430 | 214 |  |  | +ssRNA | *Narnaviridae* |  |
| 56 |  | Ss-AA_clean.1_(paired)_contig_3529 | 219 |  |  | +ssRNA | *Narnaviridae* |  |
| 57 |  | Ss-AA_clean.1_(paired)_contig_429 | 338 | RNA-dependent RNA polymerases [Sclerotinia sclerotiorum mitovirus 1]7-118 | 85% | +ssRNA | *Narnaviridae* | 2.00E-57 |
| 58 |  | Ss-AA_clean.1_(paired)_contig_641 | 285 | RNA-dependent RNA polymerases [Sclerotinia sclerotiorum mitovirus 1] | 91% | +ssRNA | *Narnaviridae* | 1E-52 |
| 59 |  | Ss-AA_clean.1_(paired)_contig_624 | 276 |  |  | +ssRNA | *Narnaviridae* |  |
| 60 |  | Ss-AA_clean.1_(paired)_contig_198 | 292 | RNA-dependent RNA polymerase [Sclerotinia sclerotiorum mitovirus 1]632-722 | 96% |  |  | 1.00E-50 |
| 61 | Sclerotinia sclerotiorum mitovirus 1 HC025 | Ss-AA_clean.1_(paired)_contig_67 | 2477 | RNA-dependent RNA polymerase [Sclerotinia sclerotiorum mitovirus 1 HC025]1-723 | 90% | +ssRNA | *Narnaviridae* | 4.00E-32 |
| 62 | Sclerotinia sclerotiorum mitovirus 2 | First_Contig5 | 1929 | RNA-dependent RNA polymerase [Sclerotinia sclerotiorum mitovirus 2]1-629 | 84% | +ssRNA | *Narnaviridae* | 0 |
| 63 | Sclerotinia sclerotiorum mitovirus 2 | Ss-AA_clean.1_(paired)_contig_2607 | 264 | RNA-dependent RNA polymerase [Sclerotinia sclerotiorum mitovirus 2]1-45 | 78% | +ssRNA | *Narnaviridae* | 9E-16 |
| 64 |  | Ss-AA_clean.1_(paired)_contig_283 | 1001 | RNA-dependent RNA polymerase [Sclerotinia sclerotiorum mitovirus 2]10-335 | 87% | +ssRNA | *Narnaviridae* | 0 |
| 65 |  | Ss-AA_clean.1_(paired)_contig_284 | 1104 | RNA-dependent RNA polymerase [Sclerotinia sclerotiorum mitovirus 2]84-392 | 91% | +ssRNA | *Narnaviridae* | 0 |
| 66 |  | Ss-AA_clean.1_(paired)_contig_1263 | 219 | RNA-dependent RNA polymerase [Sclerotinia sclerotiorum mitovirus 2]311-381 | 90% | +ssRNA | *Narnaviridae* | 9E-39 |
| 67 |  | Ss-AA_clean.1_(paired)_contig_2610 | 203 | RNA-dependent RNA polymerase [Sclerotinia sclerotiorum mitovirus 2]514-544 | 81% | +ssRNA | *Narnaviridae* | 3E-09 |
| 68 | Sclerotinia sclerotiorum mitovirus 4 | Ss-AA_clean.1_(paired)_contig_238 | 2737 | RNA-dependent RNA polymerase [Sclerotinia sclerotiorum mitovirus 4]17-731 | 89% | +ssRNA | *Narnaviridae* | 0 |
| 69 | Sclerotinia sclerotiorum mitovirus 5-A | Ss-AA_clean.1_(paired)_contig_188 | 1524 | RNA-dependent RNA polymerase [Sclerotinia sclerotiorum mitovirus 5]40-301 | 86% | +ssRNA | *Narnaviridae* | 4E-163 |
| 70 | Sclerotinia sclerotiorum mitovirus 5-A | Ss-AA_clean.1_(paired)_contig_789 | 441 | RNA-dependent RNA polymerase [Sclerotinia sclerotiorum mitovirus 5]215-329 | 77% | +ssRNA | *Narnaviridae* | 4E-58 |
| 71 |  | Ss-AA_clean.1_(paired)_contig_1716 | 408 | RNA-dependent RNA polymerase [Sclerotinia sclerotiorum mitovirus 5]1-40 | 88% | +ssRNA | *Narnaviridae* | 2E-08 |
| 72 |  | Ss-AA_clean.1_(paired)_contig_781 | 279 | RNA-dependent RNA polymerase [Sclerotinia sclerotiorum mitovirus 5]28-116 | 84% | +ssRNA | *Narnaviridae* | 2E-44 |
| 73 |  | Ss-AA_clean.1_(paired)_contig_312 | 439 | RNA-dependent RNA polymerase [Sclerotinia sclerotiorum mitovirus 5]4-53 | 82% | +ssRNA | *Narnaviridae* | 2E-12 |
| 74 |  | Ss-AA_clean.1_(paired)_contig_189 | 641 | RNA-dependent RNA polymerase [Sclerotinia sclerotiorum mitovirus 5]53-257 | 87% | +ssRNA | *Narnaviridae* | 8E-117 |
| 75 |  | Ss-AA_clean.1_(paired)_contig_190 |  |  |  | +ssRNA | *Narnaviridae* |  |
| 76 |  | Ss-AA_clean.1_(paired)_contig_790 |  |  |  | +ssRNA | *Narnaviridae* |  |
| 77 |  | Ss-AA_clean.1_(paired)_contig_132 | 489 | RNA-dependent RNA polymerase [Sclerotinia sclerotiorum mitovirus 5]108-267 | 91% | +ssRNA | *Narnaviridae* | 1E-100 |
| 78 |  | Ss-AA_clean.1_(paired)_contig_66 | 1220 | RNA-dependent RNA polymerase [Sclerotinia sclerotiorum mitovirus 5]297-603 | 77% | +ssRNA | *Narnaviridae* | 6E-103 |
| 79 |  | Ss-AA_clean.1_(paired)_contig_1018 | 1013 | RNA-dependent RNA polymerase [Sclerotinia sclerotiorum mitovirus 5]360-677 | 82% | +ssRNA | *Narnaviridae* | 0 |
| 80 |  | Ss-AA_clean.1_(paired)_contig_46 |  |  |  | +ssRNA | *Narnaviridae* |  |
| 81 |  | Ss-AA_clean.1_(paired)_contig_37 |  |  |  | +ssRNA | *Narnaviridae* |  |
| 82 |  | First_Contig27 | 677 | RNA-dependent RNA polymerase [Sclerotinia sclerotiorum mitovirus 5]517-622 | 48% | +ssRNA | *Narnaviridae* | 5.00E-22 |
| 83 | Sclerotinia sclerotiorum mitovirus 6-A | Ss-AA_clean.1_(paired)_contig_308 | 2510 | RNA-dependent RNA polymerase [Sclerotinia sclerotiorum mitovirus 6]1-703 | 79% | +ssRNA | *Narnaviridae* | 0 |
| 84 | Sclerotinia sclerotiorum mitovirus 6-A2 | First_Contig17 | 2474 | Pol [Sclerotinia sclerotiorum mitovirus 6]1-698 | 88% | +ssRNA | *Narnaviridae* | 0 |
| 85 | Sclerotinia sclerotiorum mitovirus 6 | Ss-AA_clean.1_(paired)_contig_572 | 1365 | RNA-dependent RNA polymerase [Sclerotinia sclerotiorum mitovirus 6]52-350 | 92% | +ssRNA | *Narnaviridae* | 0 |
| 86 |  | Ss-AA_clean.1_(paired)_contig_8104 |  |  |  | +ssRNA | *Narnaviridae* |  |
| 87 |  | Ss-AA_clean.1_(paired)_contig_123 |  |  |  | +ssRNA | *Narnaviridae* |  |
| 88 |  | Ss-AA_clean.1_(paired)_contig_74 |  |  |  | +ssRNA | *Narnaviridae* |  |
| 89 |  | Ss-AA_clean.1_(paired)_contig_1060 |  |  |  | +ssRNA | *Narnaviridae* |  |
| 90 |  | Ss-AA_clean.1_(paired)_contig_593 | 411 | RNA-dependent RNA polymerase [Sclerotinia sclerotiorum mitovirus 6]294-384 | 80% | +ssRNA | *Narnaviridae* | 1E-42 |
| 91 |  | Ss-AA_clean.1_(paired)_contig_35 | 2049 | RNA-dependent RNA polymerase [Sclerotinia sclerotiorum mitovirus 6]356-529 | 89% | +ssRNA | *Narnaviridae* | 0 |
| 92 |  | Ss-AA_clean.1_(paired)_contig_23 | 490 | RNA-dependent RNA polymerase [Sclerotinia sclerotiorum mitovirus 6]514-612 | 92% | +ssRNA | *Narnaviridae* | 1E-56 |
| 93 |  | Ss-AA_clean.1_(paired)_contig_22 | 304 | RNA-dependent RNA polymerase [Sclerotinia sclerotiorum mitovirus 6]614-697 | 88% | +ssRNA | *Narnaviridae* | 5E-44 |
| 94 |  | First_Contig6 | 2039 | RNA-dependent RNA polymerase [Sclerotinia sclerotiorum mitovirus 6]52-672 | 75% | +ssRNA | *Narnaviridae* | 0 |
| 95 |  | First_Contig2 | 1261 | RNA-dependent RNA polymerase [Sclerotinia sclerotiorum mitovirus 6]344-703 | 83% | +ssRNA | *Narnaviridae* | 0 |
| 96 |  | First_Contig38 | 1536 | RNA-dependent RNA polymerase [Sclerotinia sclerotiorum mitovirus 6]1-357 | 83% | +ssRNA | *Narnaviridae* | 0.00E+00 |
| 97 |  | First_Contig28 | 349 | RNA-dependent RNA polymerase [Sclerotinia sclerotiorum mitovirus 6]48-160 | 43% | +ssRNA | *Narnaviridae* | 7.00E-11 |
| 98 |  | First_Contig62 | 521 | RNA-dependent RNA polymerase [Sclerotinia sclerotiorum mitovirus 6]607-739 | 38% | +ssRNA | *Narnaviridae* | 2.00E-09 |
| 99 |  | First_Contig19 | 312 | RNA-dependent RNA polymerase [Sclerotinia sclerotiorum mitovirus 6]357-436 | 49% | +ssRNA | *Narnaviridae* | 2.00E-08 |
| 100 | Sclerotinia sclerotiorum mitovirus 7-A | Ss-AA_clean.1_(paired)_contig_69 | 2602 | RNA-dependent RNA polymerase [Sclerotinia sclerotiorum mitovirus 7]1-703 | 82% | +ssRNA | *Narnaviridae* | 0 |
| 101 | Sclerotinia sclerotiorum mitovirus 7-A2 | Ss-AA_clean.1_(paired)_contig_148 | 2582 | RNA-dependent RNA polymerase [Sclerotinia sclerotiorum mitovirus 7]1-703 | 76% | +ssRNA | *Narnaviridae* | 0 |
| 102 | Sclerotinia sclerotiorum mitovirus 7 | Ss-AA_clean.1_(paired)_contig_99 | 583 | RNA-dependent RNA polymerase [Sclerotinia sclerotiorum mitovirus 7]1-32 | 84% | +ssRNA | *Narnaviridae* | 4E-11 |
| 103 |  | Ss-AA_clean.1_(paired)_contig_154 | 442 | RNA-dependent RNA polymerase [Sclerotinia sclerotiorum mitovirus 7]35-169 | 83% | +ssRNA | *Narnaviridae* | 3E-72 |
| 104 |  | Ss-AA_clean.1_(paired)_contig_469 | 465 | RNA-dependent RNA polymerase [Sclerotinia sclerotiorum mitovirus 7]1-39 | 92% | +ssRNA | *Narnaviridae* | 1.00E-15 |
| 105 |  | Ss-AA_clean.1_(paired)_contig_1539 | 491 | RNA-dependent RNA polymerase [Sclerotinia sclerotiorum mitovirus 7]36-181 | 93% | +ssRNA | *Narnaviridae* | 3.00E-87 |
| 106 |  | Ss-AA_clean.1_(paired)_contig_174 | 387 | RNA-dependent RNA polymerase [Sclerotinia sclerotiorum mitovirus 7]222-312 | 98% | +ssRNA | *Narnaviridae* | 5E-54 |
| 107 |  | Ss-AA_clean.1_(paired)_contig_175 |  |  |  | +ssRNA | *Narnaviridae* |  |
| 108 |  | Ss-AA_clean.1_(paired)_contig_1126 |  |  |  | +ssRNA | *Narnaviridae* |  |
| 109 |  | Ss-AA_clean.1_(paired)_contig_6259 | 289 | RNA-dependent RNA polymerase [Sclerotinia sclerotiorum mitovirus 7]278-361 | 85% | +ssRNA | *Narnaviridae* | 2E-43 |
| 110 |  | Ss-AA_clean.1_(paired)_contig_176 | 480 | RNA-dependent RNA polymerase [Sclerotinia sclerotiorum mitovirus 7]301-384 | 92% | +ssRNA | *Narnaviridae* | 1E-47 |
| 111 |  | Ss-AA_clean.1_(paired)_contig_397 | 425 | RNA-dependent RNA polymerase [Sclerotinia sclerotiorum mitovirus 7]375-515 | 83% | +ssRNA | *Narnaviridae* | 7E-80 |
| 112 |  | Ss-AA_clean.1_(paired)_contig_718 | 335 | RNA-dependent RNA polymerase [Sclerotinia sclerotiorum mitovirus 7]375-485 | 86% | +ssRNA | *Narnaviridae* | 8E-62 |
| 113 |  | Ss-AA_clean.1_(paired)_contig_11577 | 227 | RNA-dependent RNA polymerase [Sclerotinia sclerotiorum mitovirus 7]388-433 | 83% | +ssRNA | *Narnaviridae* | 3E-17 |
| 114 |  | Ss-AA_clean.1_(paired)_contig_64 | 332 | RNA-dependent RNA polymerase [Sclerotinia sclerotiorum mitovirus 7] 660-703 | 77% | +ssRNA | *Narnaviridae* | 2E-14 |
| 115 |  | Ss-AA_clean.1_(paired)_contig_331 | 460 | Pol, partial [Sclerotinia sclerotiorum mitovirus 7]215-340 | 74% | +ssRNA | *Narnaviridae* | 3E-57 |
| 116 |  | Ss-AA_clean.1_(paired)_contig_597 | 302 | Pol, partial [Sclerotinia sclerotiorum mitovirus 7]70-169 | 68% | +ssRNA | *Narnaviridae* | 6.00E-36 |
| 117 |  | First_Contig18 | 1604 | Pol, partial [Sclerotinia sclerotiorum mitovirus 7]66-244 | 36% | +ssRNA | *Narnaviridae* | 2.00E-13 |
| 118 |  | Ss-AA_clean.1_(paired)_contig_1170 | 328 | Pol, partial [Sclerotinia sclerotiorum mitovirus 7]612-689 | 87% | +ssRNA | *Narnaviridae* | 3E-39 |
| 119 | Sclerotinia sclerotiorum mitovirus 8 | Ss-AA_clean.1_(paired)_contig_153 | 642 | Pol, partial [Sclerotinia sclerotiorum mitovirus 8]152-320 | 85% | +ssRNA | *Narnaviridae* | 2E-95 |
| 120 |  | Ss-AA_clean.1_(paired)_contig_15 | 273 | Pol, partial [Sclerotinia sclerotiorum mitovirus 8]472-522 | 75% | +ssRNA | *Narnaviridae* | 7E-18 |
| 121 |  | First_Contig42 | 765 | Pol, partial [Sclerotinia sclerotiorum mitovirus 8]279-467 | 87% | +ssRNA | *Narnaviridae* | 3E-109 |
| 122 |  | First_Contig43 | 529 | Pol, partial [Sclerotinia sclerotiorum mitovirus 8]176-203 | 68% | +ssRNA | *Narnaviridae* | 0.017 |
| 123 | Sclerotinia sclerotiorum mitovirus 9 | Ss-AA_clean.1_(paired)_contig_202 | 2510 | RNA-dependent RNA polymerase [Sclerotinia sclerotiorum mitovirus 9]190-325 | 96% | +ssRNA | *Narnaviridae* | 0 |
| 124 | Sclerotinia sclerotiorum mitovirus 10 | Ss-AA_clean.1_(paired)_contig_226 | 808 | RNA-dependent RNA polymerase [Sclerotinia sclerotiorum mitovirus 10]216-353 | 86% | +ssRNA | *Narnaviridae* | 8E-66 |
| 125 |  | Ss-AA_clean.1_(paired)_contig_737 |  |  |  | +ssRNA | *Narnaviridae* |  |
| 126 |  | Ss-AA_clean.1_(paired)_contig_227 |  |  |  | +ssRNA | *Narnaviridae* |  |
| 127 |  | Ss-AA_clean.1_(paired)_contig_916 |  |  |  | +ssRNA | *Narnaviridae* |  |
| 128 | Sclerotinia sclerotiorum mitovirus 11 | Ss-AA_clean.1_(paired)_contig_140 | 734 | RNA-dependent RNA polymerase [Sclerotinia sclerotiorum mitovirus 11]519-683 | 76% | +ssRNA | *Narnaviridae* | 2E-110 |
| 129 | Sclerotinia sclerotiorum mitovirus 11 | First_Contig3 | 748 | RNA-dependent RNA polymerase [Sclerotinia sclerotiorum mitovirus 11]513-982 | 83% | +ssRNA | *Narnaviridae* | 3E-94 |
| 130 |  | First_Contig221 | 720 | RNA-dependent RNA polymerase [Sclerotinia sclerotiorum mitovirus 11]555-682 | 41% | +ssRNA | *Narnaviridae* | 4E-10 |
| 131 | Sclerotinia sclerotiorum mitovirus 12 | Ss-AA_clean.1_(paired)_contig_85 | 327 | >AHF48628.1 RNA-dependent RNA polymerase [Sclerotinia sclerotiorum mitovirus 12]643-693 | 84% | +ssRNA | *Narnaviridae* | 1.00E-18 |
| 132 |  | First_Contig26 | 1176 | RNA-dependent RNA polymerase [Sclerotinia sclerotiorum mitovirus 12]41-275 | 80% | +ssRNA | *Narnaviridae* | 2.00E-135 |
| 133 |  | Second_Contig2 | 1071 | RNA-dependent RNA polymerase [Sclerotinia sclerotiorum mitovirus 12] 378-682 | 68% | +ssRNA | *Narnaviridae* | 7.00E-24 |
| 134 | Sclerotinia sclerotiorum mitovirus 14 | Ss-AA_clean.1_(paired)_contig_126 | 2557 | RNA-dependent RNA polymerase [Sclerotinia sclerotiorum mitovirus 14] 1-714 | 82% | +ssRNA | *Narnaviridae* | 0 |
| 135 |  | Ss-AA_clean.1_(paired)_contig_12119 |  |  |  | +ssRNA | *Narnaviridae* |  |
| 136 | Sclerotinia sclerotiorum mitovirus 17 | Ss-AA_clean.1_(paired)_contig_76 | 1559 | RNA-dependent RNA polymerase [Sclerotinia sclerotiorum mitovirus 17]1-58 | 98% | +ssRNA | *Narnaviridae* | 6E-28 |
| 137 |  | Ss-AA_clean.1_(paired)_contig_73 |  | RNA-dependent RNA polymerase [Sclerotinia sclerotiorum mitovirus 17]64-163 | 95% | +ssRNA | *Narnaviridae* | 0 |
| 138 |  | Ss-AA_clean.1_(paired)_contig_2 | 687 | RNA-dependent RNA polymerase [Sclerotinia sclerotiorum mitovirus 17]514-578 | 96% | +ssRNA | *Narnaviridae* | 2.00E-106 |
| 139 |  | Ss-AA_clean.1_(paired)_contig_207 |  |  |  | +ssRNA | *Narnaviridae* |  |
| 140 | Sclerotinia sclerotiorum mitovirus 19 | Ss-AA_clean.1_(paired)_contig_143 | 953 | RNA-dependent RNA polymerase [Sclerotinia sclerotiorum mitovirus 19]168-455 | 88% | +ssRNA | *Narnaviridae* | 0 |
| 141 |  | Ss-AA_clean.1_(paired)_contig_144 |  |  |  | +ssRNA | *Narnaviridae* |  |
| 142 |  | Ss-AA_clean.1_(paired)_contig_569 | 1448 | RNA-dependent RNA polymerase [Sclerotinia sclerotiorum mitovirus 19]493-579 | 81% | +ssRNA | *Narnaviridae* | 0 |
| 143 | Sclerotinia sclerotiorum mitovirus 24 | Ss-AA_clean.1_(paired)_contig_24 | 293 | RNA-dependent RNA polymerase [Sclerotinia sclerotiorum mitovirus 7] | 62% | +ssRNA | *Narnaviridae* | 2E-24 |
| 144 | Sclerotinia sclerotiorum mitovirus 25 | First_Contig15 | 1447 | RNA-dependent RNA polymerase [Sclerotinia sclerotiorum mitovirus 5]298-575 | 62% | +ssRNA | *Narnaviridae* | 4E-103 |
| 145 | Sclerotinia sclerotiorum mitovirus 26 | Ss-AA_clean.1_(paired)_contig_634 | 1469 | RNA-dependent RNA polymerase [Rhizoctonia solani mitovirus 10]262-728 | 51% | +ssRNA | *Narnaviridae* | 9.00E-154 |
| 146 |  | Ss-AA_clean.1_(paired)_contig_1286 |  |  |  | +ssRNA | *Narnaviridae* |  |
| 147 |  | Ss-AA_clean.1_(paired)_contig_635 |  |  |  | +ssRNA | *Narnaviridae* |  |
| 148 |  | Ss-AA_clean.1_(paired)_contig_896 |  |  |  | +ssRNA | *Narnaviridae* |  |
| 149 |  | Ss-AA_clean.1_(paired)_contig_649 |  |  |  | +ssRNA | *Narnaviridae* |  |
| 150 |  | Ss-AA_clean.1_(paired)_contig_2035 |  |  |  | +ssRNA | *Narnaviridae* |  |
| 151 |  | Ss-AA_clean.1_(paired)_contig_1377 |  |  |  | +ssRNA | *Narnaviridae* |  |
| 152 |  | First_Contig87 | 1350 | RNA-dependent RNA polymerase, partial [Rhizoctonia solani mitovirus 10]73-395 | 25% | +ssRNA | *Narnaviridae* | 0.0000007 |
| 153 |  | Ss-AA_clean.1_(paired)_contig_3142 | 354 | RNA-dependent RNA polymerase, partial [Rhizoctonia solani mitovirus 10]75-160 | 55% | +ssRNA | *Narnaviridae* | 2e-18 |
| 154 | Sclerotinia sclerotiorum mitovirus 27 | Ss-AA_clean.1_(paired)_contig_958 | 2917 | RNA-dependent RNA polymerase [Ophiostoma mitovirus 1a]355-720 | 51% | +ssRNA | *Narnaviridae* | 0 |
| 155 | Sclerotinia sclerotiorum mitovirus 28 | Ss-AA_clean.1_(paired)_contig_867 | 2765 | RNA dependent RNA polymerase [Botrytis cinerea mitovirus 3]428-664 | 37% | +ssRNA | *Narnaviridae* | 2E-125 |
| 156 | Sclerotinia sclerotiorum mitovirus 29 | Ss-AA_clean.1_(paired)_contig_536 | 3262 | RNA dependent RNA polymerase [Botrytis cinerea mitovirus 3]54-542 | 38% | +ssRNA | *Narnaviridae* | 3E-97 |
| 157 | Sclerotinia sclerotiorum mitovirus 30 | Ss-AA_clean.1_(paired)_contig_59 | 2536 | RNA-dependent RNA polymerase [Macrophomina phaseolina mitovirus 1]1-714 | 83% | +ssRNA | *Narnaviridae* | 0 |
| 158 | Sclerotinia sclerotiorum mitovirus 30 | Ss-AA_clean.1_(paired)_contig_39 | 940 | RNA-dependent RNA polymerase [Macrophomina phaseolina mitovirus 1]1-320 | 86% | +ssRNA | *Narnaviridae* | 0 |
| 159 |  | First_Contig49 | 1412 | RNA-dependent RNA polymerase [Macrophomina phaseolina mitovirus 1]1-432 | 89% | +ssRNA | *Narnaviridae* | 0 |
| 160 |  | Ss-AA_clean.1_(paired)_contig_50 | 638 | RNA-dependent RNA polymerase [Macrophomina phaseolina mitovirus 1]116-281 | 80% | +ssRNA | *Narnaviridae* | 5E-88 |
| 161 |  | Ss-AA_clean.1_(paired)_contig_28 | 1429 | RNA-dependent RNA polymerase [Macrophomina phaseolina mitovirus 1]41-506 | 75% | +ssRNA | *Narnaviridae* | 0 |
| 162 |  | Ss-AA_clean.1_(paired)_contig_119 | 1389 | RNA-dependent RNA polymerase [Macrophomina phaseolina mitovirus 1]27-507 | 83% | +ssRNA | *Narnaviridae* | 0 |
| 163 |  | First_Contig22 | 1699 | RNA-dependent RNA polymerase [Macrophomina phaseolina mitovirus 1]208-595 | 84% | +ssRNA | *Narnaviridae* | 0 |
| 164 | Sclerotinia sclerotiorum mitovirus 31 | First_Contig31 | 1695 | RNA-dependent RNA polymerase [Sclerotinia sclerotiorum mitovirus 2]1-414 | 60% | +ssRNA | *Narnaviridae* | 2E-32 |
| 165 |  | First_Contig24 |  |  |  | +ssRNA | *Narnaviridae* | 7E-68 |
| 166 | Sclerotinia sclerotiorum mitovirus 32 | Ss-AA_clean.1_(paired)_contig_1865 | 2473 | RNA-dependent RNA polymerase [Sclerotinia sclerotiorum mitovirus 6]1-703 | 70% | +ssRNA | *Narnaviridae* | 0 |
| 167 | Sclerotinia sclerotiorum mitovirus 33 | Ss-AA_clean.1_(paired)_contig_90 | 2661 | RNA-dependent RNA polymerase [Sclerotinia sclerotiorum mitovirus 19]2-687 | 45% | +ssRNA | *Narnaviridae* | 0 |
| 168 | Sclerotinia sclerotiorum tymo-like RNA virus 2 | Ss-AA_clean.1_(paired)_contig_3545 | 1575 | polyprotein [Nectarine virus M]81-364 | 31% | +ssRNA | *Tymoviridae* | 4.00E-26 |
| 169 |  | Ss-AA_clean.1_(paired)_contig_13690 |  |  |  | +ssRNA | *Tymoviridae* |  |
| 170 | Sclerotinia sclerotiorum tymo-like RNA virus 3 | Ss-AA_clean.1_(paired)_contig_10849 | 964 | replicase [Turnip yellow mosaic virus]18-262 | 41% | +ssRNA | *Tymoviridae* | 7E-51 |
| 171 | Sclerotinia sclerotiorum tymo-like RNA virus 4 | Ss-AA_clean.1_(paired)_contig_11935 | 2926 | replicase-associated protein [Poinsettia mosaic virus]864-1520 | 27% | +ssRNA | *Tymoviridae* | 8E-66 |
| 172 | Sclerotinia sclerotiorum tymo-like RNA virus 5 | Ss-AA_clean.1_(paired)_contig_14854 | 796 | replication-associated polyprotein [Fusarium graminearum mycotymovirus 1] | 32% | +ssRNA | *Tymovirales* | 5E-25 |
| 173 |  | Ss-AA_clean.1_(paired)_contig_18967 | 636 | replication-associated polyprotein [Fusarium graminearum mycotymovirus 1]2035-2159 | 30% | +ssRNA | *Tymovirales* | 3.00E-11 |
| 174 |  | Ss-AA_clean.1_(paired)_contig_4648 |  |  |  | +ssRNA | *Tymoviridae* |  |
| 175 | Sclerotinia sclerotiorum tymo-like RNA virus 6 | Ss-AA_clean.1_(paired)_contig_9241 | 2307 | hypothetical protein A4X09_g7752, partial [Tilletia walkeri] | 51% |  |  | 0 |
| 176 |  | Ss-AA_clean.1_(paired)_contig_5513 |  |  |  |  |  |  |
| 177 |  | Ss-AA_clean.1_(paired)_contig_5512 |  | replication-associated polyprotein [Fusarium graminearum deltaflexivirus 1]1310-2010 | 43% | +ssRNA | *Tymovirales* | 4.00E-154 |
| 178 |  | Ss-AA_clean.1_(paired)_contig_13466 | 756 | hypothetical protein A4X06_g9439, partial [Tilletia controversa]132-303 | 48% |  |  | 0 |
| 179 |  | Ss-AA_clean.1_(paired)_contig_15254 |  | replication-associated polyprotein [Fusarium graminearum deltaflexivirus 1]251-422 | 38% | +ssRNA | *Tymovirales* | 2.00E-37 |
| 180 | Sclerotinia sclerotiorum umbra-like virus 2 | Ss-AA_clean.1_(paired)_contig_82 | 1177 | RNA-dependent RNA polymerase [Sclerotinia sclerotiorum umbra-like virus 1] | 48% | +ssRNA | *Tombusviridae* | 5E-36 |
| 181 |  | First_Contig1 | 1700 |  |  | +ssRNA | *Tombusviridae* |  |
| 182 |  | First_Contig57 | 2113 |  |  | +ssRNA | *Tombusviridae* |  |
| 183 |  | Ss-AA_clean.1_(paired)_contig_224 | 4170 | RNA-dependent RNA polymerase [Sclerotinia sclerotiorum umbra-like virus 1] | 61% | +ssRNA | *Tombusviridae* | 0 |
| 184 |  | First_Contig60 | 1224 | >YP_009253999.1 replication-associated protein [Sclerotinia sclerotiorum umbra-like virus 1]56-411 | 39% | +ssRNA | *Tombusviridae* | 3E-43 |
| 185 | Sclerotinia sclerotiorum umbra-like virus 1-A | Ss-AA_clean.1_(paired)_contig_29 | 604 | RNA-dependent RNA polymerase [Sclerotinia sclerotiorum umbra-like virus 1] | 99% | +ssRNA | *Tombusviridae* | 3E-136 |
| 186 |  | Ss-AA_clean.1_(paired)_contig_381 | 325 | >YP_009253999.1 replication-associated protein [Sclerotinia sclerotiorum umbra-like virus 1]164-245 | 95% | +ssRNA | *Tombusviridae* | 3e-34 |
| 187 | Sclerotinia sclerotiorum umbra-like virus 3 | Ss-AA_clean.1_(paired)_contig_534 | 3925 | RNA-dependent RNA polymerase [Magnaporthe oryzae RNA virus] | 53% | +ssRNA | *Tombusviridae* | 7E-153 |
| 188 | Sclerotinia sclerotiorum ourmia-like virus 1-A | Ss-AA_clean.1_(paired)_contig_195 | 589 | RNA-dependent RNA polymerase [Sclerotinia sclerotiorum ourmia-like virus 1 RNA 1]1-111 | 84% | +ssRNA | *Ourmiavirus* | 9E-79 |
| 189 |  | Ss-AA_clean.1_(paired)_contig_1976 |  |  |  | +ssRNA | *Ourmiavirus* | 9E-28 |
| 190 |  | Ss-AA_clean.1_(paired)_contig_2128 |  |  |  | +ssRNA | *Ourmiavirus* | 2E-34 |
| 191 |  | Ss-AA_clean.1_(paired)_contig_628 |  |  |  | +ssRNA | *Ourmiavirus* | 4E-33 |
| 192 |  | Ss-AA_clean.1_(paired)_contig_872 |  |  |  | +ssRNA | *Ourmiavirus* | 3E-57 |
| 193 |  | Ss-AA_clean.1_(paired)_contig_20 |  |  |  | +ssRNA | *Ourmiavirus* | 6E-34 |
| 194 |  | Ss-AA_clean.1_(paired)_contig_277 |  |  |  | +ssRNA | *Ourmiavirus* | 6E-16 |
| 195 |  | First_Contig126 |  |  |  | +ssRNA | *Ourmiavirus* | 1E-13 |
| 196 |  | Ss-AA_clean.1_(paired)_contig_278 | 1467 | RNA-dependent RNA polymerase [Sclerotinia sclerotiorum ourmia-like virus 1 RNA 1]1-321 | 84% | +ssRNA | *Ourmiavirus* | 0 |
| 197 |  | Ss-AA_clean.1_(paired)_contig_40 |  |  |  | +ssRNA | *Ourmiavirus* |  |
| 198 |  | First_Contig56 |  |  |  | +ssRNA | *Ourmiavirus* |  |
| 199 |  | Ss-AA_clean.1_(paired)_contig_4328 |  |  |  | +ssRNA | *Ourmiavirus* |  |
| 200 |  | Ss-AA_clean.1_(paired)_contig_131 |  |  |  | +ssRNA | *Ourmiavirus* |  |
| 201 |  | Ss-AA_clean.1_(paired)_contig_937 |  |  |  | +ssRNA | *Ourmiavirus* |  |
| 202 |  | Ss-AA_clean.1_(paired)_contig_783 |  |  |  | +ssRNA | *Ourmiavirus* |  |
| 203 |  | First_Contig39 |  |  |  | +ssRNA | *Ourmiavirus* |  |
| 204 |  | Ss-AA_clean.1_(paired)_contig_1492 |  |  |  | +ssRNA | *Ourmiavirus* |  |
| 205 |  | Ss-AA_clean.1_(paired)_contig_7164 |  |  |  | +ssRNA | *Ourmiavirus* |  |
| 206 |  | Ss-AA_clean.1_(paired)_contig_758 | 366 | RNA-dependent RNA polymerase [Sclerotinia sclerotiorum ourmia-like virus 1 RNA 1]322-428 | 84% | +ssRNA | *Ourmiavirus* | 5E-68 |
| 207 |  | Ss-AA_clean.1_(paired)_contig_359 |  | RNA-dependent RNA polymerase [Sclerotinia sclerotiorum ourmia-like virus 1 RNA 1]327-422 | 87% | +ssRNA | *Ourmiavirus* |  |
| 208 |  | First_Contig21 |  | RNA-dependent RNA polymerase [Sclerotinia sclerotiorum ourmia-like virus 1 RNA 1]321-546 | 85% | +ssRNA | *Ourmiavirus* |  |
| 209 |  | Ss-AA_clean.1_(paired)_contig_6355 |  | RNA-dependent RNA polymerase [Sclerotinia sclerotiorum ourmia-like virus 1 RNA 1]318-400 | 81% | +ssRNA | *Ourmiavirus* |  |
| 210 |  | Ss-AA_clean.1_(paired)_contig_630 |  | RNA-dependent RNA polymerase [Sclerotinia sclerotiorum ourmia-like virus 1 RNA 1]354-468 | 77% | +ssRNA | *Ourmiavirus* |  |
| 211 |  | Ss-AA_clean.1_(paired)_contig_418 | 318 | RNA-dependent RNA polymerase [Sclerotinia sclerotiorum ourmia-like virus 1 RNA 1]424-528 | 91% | +ssRNA | *Ourmiavirus* | 4E-62 |
| 212 |  | Ss-AA_clean.1_(paired)_contig_1160 | 680 | RNA-dependent RNA polymerase [Sclerotinia sclerotiorum ourmia-like virus 1 RNA 1]576-686 | 91% | +ssRNA | *Ourmiavirus* | 8E-58 |
| 213 |  | Ss-AA_clean.1_(paired)_contig_150 |  |  |  | +ssRNA | *Ourmiavirus* |  |
| 214 |  | Ss-AA_clean.1_(paired)_contig_94 |  |  |  | +ssRNA | *Ourmiavirus* |  |
| 215 |  | Ss-AA_clean.1_(paired)_contig_149 |  |  |  | +ssRNA | *Ourmiavirus* |  |
| 216 |  | Ss-AA_clean.1_(paired)_contig_280 | 569 | RNA-dependent RNA polymerase [Sclerotinia sclerotiorum ourmia-like virus 1 RNA 1]591-670 | 74% | +ssRNA | *Ourmiavirus* | 8E-34 |
| 217 |  | First_Contig12 | 368 | RNA-dependent RNA polymerase [Sclerotinia sclerotiorum ourmia-like virus 1 RNA 1]524-603 | 87% | +ssRNA | *Ourmiavirus* | 9E-11 |
| 218 | Sclerotinia sclerotiorum ourmia-like virus 3 | First_Contig30 | 2442 | RNA-dependent RNA polymerase, partial [Sclerotinia sclerotiorum ourmia-like virus 2 RNA 1]4-606 | 40% | +ssRNA | *Ourmiavirus* | 4.00E-137 |
| 219 |  | Ss-AA_clean.1_(paired)_contig_1694 |  |  |  | +ssRNA | *Ourmiavirus* |  |
| 220 |  | Ss-AA_clean.1_(paired)_contig_4006 |  |  |  | +ssRNA | *Ourmiavirus* |  |
| 221 |  | Ss-AA_clean.1_(paired)_contig_5114 |  |  |  | +ssRNA | *Ourmiavirus* |  |
| 222 | Sclerotinia sclerotiorum negative-stranded RNA virus 1 | First_Contig88 | 7739 | large polymerase [Sclerotinia sclerotiorum negative-stranded RNA virus 1]1-1561 | 97% | -ssRNA | *Mymonaviridae* | 0 |
| 223 |  | First_Contig83 |  |  |  | -ssRNA | *Mymonaviridae* | 0 |
| 224 |  | First_Contig89 |  |  |  | -ssRNA | *Mymonaviridae* |  |
| 225 |  | Ss-AA_clean.1_(paired)_contig_398 |  |  |  | -ssRNA | *Mymonaviridae* |  |
| 226 |  | Ss-AA_clean.1_(paired)_contig_3470 |  |  |  | -ssRNA | *Mymonaviridae* |  |
| 227 |  | Ss-AA_clean.1_(paired)_contig_4115 |  |  |  | -ssRNA | *Mymonaviridae* |  |
| 228 |  | First_Contig195 |  |  |  | -ssRNA | *Mymonaviridae* |  |
| 229 |  | Ss-AA_clean.1_(paired)_contig_1094 | 1320 | nucleoprotein [Sclerotinia sclerotiorum negative-stranded RNA virus 1]1-297 | 99% | -ssRNA | *Mymonaviridae* | 0 |
| 230 |  | Ss-AA_clean.1_(paired)_contig_2273 |  |  |  | -ssRNA | *Mymonaviridae* |  |
| 231 |  | Ss-AA_clean.1_(paired)_contig_1824 |  |  |  | -ssRNA | *Mymonaviridae* |  |
| 232 |  | Ss-AA_clean.1_(paired)_contig_4907 | 767 | hypothetical protein [Sclerotinia sclerotiorum negative-stranded  RNA virus 1]1-203 | 99% | -ssRNA | *Mymonaviridae* | 6.00E-145 |
| 233 |  | Ss-AA_clean.1_(paired)_contig_2381 | 256 | large polymerase [Sclerotinia sclerotiorum negative-stranded RNA virus 1]1138-1221 | 99% | -ssRNA | *Mymonaviridae* | 2.00E-48 |
| 234 |  | Ss-AA_clean.1_(paired)_contig_1368 | 292 | large polymerase [Sclerotinia sclerotiorum negative-stranded RNA virus 1]491-586 | 99% | -ssRNA | *Mymonaviridae* | 1.00E-58 |
| 235 | Sclerotinia sclerotiorum negative-stranded RNA virus 2 | Ss-AA_clean.1_(paired)_contig_100 | 9616 | RDRP [Sclerotinia sclerotiorum negative-stranded RNA virus 2]1-1414 | 93% | -ssRNA | *Mymonaviridae* | 8E-127 |
| 236 |  | First_Contig7 | 9452 | RNA-dependent RNA polymerase [Sclerotinia sclerotiorum negative-stranded RNA virus 2]235-1956 | 90% | -ssRNA | *Mymonaviridae* | 0 |
| 237 |  | First_Contig206 |  |  | 62% | -ssRNA | *Mymonaviridae* | 5E-14 |
| 238 |  | Ss-AA_clean.1_(paired)_contig_2473 |  |  | 94% | -ssRNA | *Mymonaviridae* | 3e-88 |
| 239 |  | Ss-AA_clean.1_(paired)_contig_1376 | 2801 | RNA-dependent RNA polymerase [Sclerotinia sclerotiorum negative-stranded RNA virus 2]1680-1823 | 30% | -ssRNA | *Mymonaviridae* | 0.000003 |
| 240 |  | Ss-AA_clean.1_(paired)_contig_1375 |  |  | 30% | -ssRNA | *Mymonaviridae* | 0.000002 |
| 241 | Sclerotinia sclerotiorum negative-stranded RNA virus 3 | Ss-AA_clean.1_(paired)_contig_6637 | 1462 | gp1 [Sclerotinia sclerotiorum negative-stranded RNA virus 3]38-289 | 97% | -ssRNA | *Mymonaviridae* | 5.00E-173 |
| 242 |  | Ss-AA_clean.1_(paired)_contig_651 |  |  |  | -ssRNA | *Mymonaviridae* |  |
| 243 |  | Ss-AA_clean.1_(paired)_contig_650 |  |  |  | -ssRNA | *Mymonaviridae* |  |
| 244 |  | Ss-AA_clean.1_(paired)_contig_1093 |  |  |  | -ssRNA | *Mymonaviridae* |  |
| 245 |  | Ss-AA_clean.1_(paired)_contig_925 | 1664 | gp2 [Sclerotinia sclerotiorum negative-stranded RNA virus 3]1-375 | 99% | -ssRNA | *Mymonaviridae* | 0 |
| 246 |  | Ss-AA_clean.1_(paired)_contig_3817 |  |  |  | -ssRNA | *Mymonaviridae* |  |
| 247 |  | Ss-AA_clean.1_(paired)_contig_952 |  |  |  | -ssRNA | *Mymonaviridae* |  |
| 248 |  | First_Contig67 |  |  |  | -ssRNA | *Mymonaviridae* |  |
| 249 |  | First_Contig68 |  |  |  | -ssRNA | *Mymonaviridae* |  |
| 250 |  | Ss-AA_clean.1_(paired)_contig_951 |  |  |  | -ssRNA | *Mymonaviridae* |  |
| 251 |  | Ss-AA_clean.1_(paired)_contig_2258 | 321 | gp3 [Sclerotinia sclerotiorum negative-stranded RNA virus 3]319-486 | 95% | -ssRNA | *Mymonaviridae* | 5.00E-68 |
| 252 |  | Ss-AA_clean.1_(paired)_contig_2349 | 283 | gp3 [Sclerotinia sclerotiorum negative-stranded RNA virus 3]1-269 | 98% | -ssRNA | *Mymonaviridae* | 0.00E+00 |
| 253 |  | First_Contig95 | 1267 |  |  | -ssRNA | *Mymonaviridae* |  |
| 254 |  | Ss-AA_clean.1_(paired)_contig_4783 | 427 |  |  | -ssRNA | *Mymonaviridae* |  |
| 255 |  | First_Contig29 | 8489 | gp5 and gp6 [Sclerotinia sclerotiorum negative-stranded RNA virus 3]1-1832,1-163 | 90% | -ssRNA | *Mymonaviridae* | 0 |
| 256 |  | First_Contig47 |  |  | 87% | -ssRNA | *Mymonaviridae* | 0 |
| 257 |  | Ss-AA_clean.1_(paired)_contig_558 |  |  | 95% | -ssRNA | *Mymonaviridae* | 2.00E-105 |
| 258 |  | Ss-AA_clean.1_(paired)_contig_977 |  |  | 99% | -ssRNA | *Mymonaviridae* | 1.00E-128 |
| 259 |  | First_Contig125 |  |  | 46% | -ssRNA | *Mymonaviridae* | 3.00E-14 |
| 260 |  | Ss-AA_clean.1_(paired)_contig_557 |  |  | 98% | -ssRNA | *Mymonaviridae* | 1E-130 |
| 261 |  | Ss-AA_clean.1_(paired)_contig_981 |  |  | 87% | -ssRNA | *Mymonaviridae* | 0 |
| 262 |  | Ss-AA_clean.1_(paired)_contig_1357 |  |  | 99% | -ssRNA | *Mymonaviridae* | 2.00E-151 |
| 263 |  | Ss-AA_clean.1_(paired)_contig_1426 |  |  | 97% | -ssRNA | *Mymonaviridae* | 0 |
| 264 |  | Ss-AA_clean.1_(paired)_contig_1219 |  |  | 100% | -ssRNA | *Mymonaviridae* | 9.00E-50 |
| 265 |  | Ss-AA_clean.1_(paired)_contig_344 |  |  | 90% | -ssRNA | *Mymonaviridae* | 0.00E+00 |
| 266 |  | Ss-AA_clean.1_(paired)_contig_1352 |  |  | 99% | -ssRNA | *Mymonaviridae* | 3.00E-85 |
| 267 |  | Ss-AA_clean.1_(paired)_contig_1912 |  |  | 90% | -ssRNA | *Mymonaviridae* | 4.00E-69 |
| 268 |  | Ss-AA_clean.1_(paired)_contig_2096 |  |  | 96% | -ssRNA | *Mymonaviridae* | 3.00E-84 |
| 269 |  | Ss-AA_clean.1_(paired)_contig_2204 |  |  | 96% | -ssRNA | *Mymonaviridae* | 2.00E-127 |
| 270 |  | Ss-AA_clean.1_(paired)_contig_3112 |  |  | 99% | -ssRNA | *Mymonaviridae* | 1.00E-108 |
| 271 |  | Ss-AA_clean.1_(paired)_contig_4528 |  |  | 96% | -ssRNA | *Mymonaviridae* | 7.00E-40 |
| 272 |  | First_Contig247 |  |  | 79% | -ssRNA | *Mymonaviridae* | 0 |
| 273 |  | Ss-AA_clean.1_(paired)_contig_927 |  |  | 96% | -ssRNA | *Mymonaviridae* | 0 |
| 274 |  | Ss-AA_clean.1_(paired)_contig_1525 |  |  | 78% | -ssRNA | *Mymonaviridae* | 5.00E-93 |
| 275 |  | Ss-AA_clean.1_(paired)_contig_1881 |  |  | 98% | -ssRNA | *Mymonaviridae* | 2E-26 |
| 276 |  | Ss-AA_clean.1_(paired)_contig_1075 |  |  | 100% | -ssRNA | *Mymonaviridae* | 3E-39 |
| 277 |  | Ss-AA_clean.1_(paired)_contig_405 |  |  | 95% | -ssRNA | *Mymonaviridae* | 0 |
| 278 | Sclerotinia sclerotiorum negative-stranded RNA virus 4 | Ss-AA_clean.1_(paired)_contig_512 | 9564 | RDRP [Sclerotinia sclerotiorum negative-stranded RNA virus 4]1-2010 | 96% | -ssRNA | *Mymonaviridae* | 9E-123 |
| 279 |  | Ss-AA_clean.1_(paired)_contig_513 | 9554 | RNA-dependent RNA polymerase [Sclerotinia sclerotiorum negative-stranded RNA virus 4]1-854 | 95% | -ssRNA | *Mymonaviridae* | 0 |
| 280 | Sclerotinia sclerotiorum negative-stranded RNA virus 5 | Ss-AA_clean.1_(paired)_contig_4241 | 4421 | RDRP[Sclerotinia sclerotiorum negative-stranded RNA virus 4]189-625 | 32% | -ssRNA | *Mymonaviridae* | 5E-44 |
| 281 | Sclerotinia sclerotiorum negative-stranded RNA virus 6 | Ss-AA_clean.1_(paired)_contig_89 | 5076 | hypothetical protein [Soybean leaf-associated negative-stranded RNA virus 1] | 28% | -ssRNA | *Mymonaviridae* | 8E-17 |
| 282 | Sclerotinia sclerotiorum negative-stranded RNA virus 7 | Ss-AA_clean.1_(paired)_contig_579 | 7819 | RNA-dependent RNA polymerase [Soybean leaf-associated negative-stranded RNA virus 2 ]5-1791 | 37% | -ssRNA | *Mymonaviridae* | 0 |
| 283 | Sclerotinia sclerotiorum negative-stranded RNA virus 8 | Ss-AA_clean.1_(paired)_contig_1114 | 1219 | RNA-dependent RNA polymerase, partial [Soybean leaf-associated negative-stranded RNA virus 3]884-1252 | 28% | -ssRNA | *Mymonaviridae* | 5E-46 |
| 284 |  | Ss-AA_clean.1_(paired)_contig_6890 | 519 | RNA-dependent RNA polymerase, partial [Soybean leaf-associated negative-stranded RNA virus 3]650-811 | 44% | -ssRNA | *Mymonaviridae* | 4.00E-30 |
| 285 | Sclerotinia sclerotiorum hypovirulence associated DNA virus 1 | Ss-AA_clean.1_(paired)_contig_2147 | 2166 | coat protein [Sclerotinia sclerotiorum hypovirulence associated DNA virus 1] | 99% | DNA | *Genomoviridae* | 0 |
