# Supplementary material for: Virome Characterization of a Collection of S. sclerotiorum from Australia
Source: Front Microbiol. 2018 Jan 11;8:2540. doi: 10.3389/fmicb.2017.02540 (PMC5768646; doi:10.3389/fmicb.2017.02540)
Supplement: Supplementary file 2 [file Table2.DOC]

Table S2 The sequences of 285 primary cotigs assembled from RNA_Seq data of 84 strains of *Sclerotinia sclerotiorum*

| >Ss-AA_clean.1_(paired)_contig_18615  GUCCACGAGCAAGGCGUUCAACGAUGGCGGCGUGAGAGGAGAAUUCGAAGGUGGCGGAGAAGUUCGUGAAUUUCUUUGAGAGACCGAUAAAGUCCUCCGCGAGGGCAUUGGCUGUAGGGUACCCAGCCUCGAUAAGUAUAGCAUUUUCGGCAGGGCGAGCGAGGGCACGGCCUUUGGUAUUAACUGACCUGCCCACUUCGUACGCAAUUUGUACUAGGCGGGAGUCCUCGCUGCCACCGAUAGUGGCGGAUGUGCGCACGUUAGAUUUGUAACGCCGAUAUUCGGCGUCAGCACCAAGGUAGGAGCCACGUGGCGAAGCUACUACCGAUGCAAGGAAACUGUUUCUAAUGACUGUUGUCAUCGUAGCGUUUAGUGGAAAUUGGAGGUUUGGUUUUGAAGUUAAAAGCGAUAUUUCGUCAAUCUUGUUUGGAG  >Ss-AA_clean.1_(paired)_contig_15405  GACGCAGGGACAUGCUCAACAUUGGGGAUACCAUCGUCGUCAAAUGUGAGGUGUUUAACGAAGAAUCCGACGGUCCCAGAAAUGUUGAGCAUCUCGCCAGGAGCAGGAAAAGGUGAUUGACCACGAGUCCAAAGGUAGUCGUUCCAUGGGAGAGCGUUUUCCACGCGAUCGCGCACAUCUUGGUGGGCAGCACAGGAACCGGGAUGGAUAAUGGCGUUCGGGUCGAGCUGGCGGACAGAAACACAUGACAGGCCAUUAGUAGGAUUAUUCAACCAAUGGAGGAAAAACCAGCAGGCCCGGGCGCCACGGAAGAGGGCGUGGUAACCGGAGAAAGCAACAUCAGACACCCCCGCAGACUGUAAAUCUUCCCAAGCGCUGCGAGUACGGGUGGAGUCGCGACCACCGUAUGAAGCGAAACCCUCAUGUUCAGAUAUGUUGCCAGUGAAAUCAUGAGGGAUCAGAGAAGUGGGUUCGAUCCAGAACCAAGGCGAGACAGAGGCAUAGCGCAGGUGACGCGGGUCCCCGGACAGGAGCGAUGCCGCACCACAAAAGAAAGAGGAUGCGACCGCAUUACUCCCCUCGGCAGCAAAGAGUUUCCCAAGCGCACUAGUGUACAAGUUGACAAACUUAGGAAGGCCGGAAAGGAGUUGAGCCCGGUUACGUGCAGACAUAGCGGCAGUACCAGCGAUGUUGUCACCGGGGCGUGUAGUCGGGUCAUCUGCCGAGGUACCAGAAUAGAAAGUCGGUAACCACCUACCGUCAUAGUACUCACCUGGGUCACAAUGAGCGACCAGAGCAGCGGUGGUGAGAGCGAUGGAAUCGACGUAGGCAGCCUUACCUGACCCGGAGUUUGUUGAGAGAGCAGGGAUGCCGGCGUAUGGCUCGAGACCAUAAUGGAUGCCACCGAAAGGUACAGAGAAAGAACCGGUUCGCAAGAGGUCACGCGUUAUCCCACCUUCAUCAGUAUGGCCGACAACAGACAGGACUUUGUGUAGACCACGAGUGAAGGCAAGCGCGAACAAGGGCCCCUGGUCGGAGGCGAUCAUGUUAGCCCCGAGUAUACGGAGAGCAUCAACGAUAGCGCGUGAGAGGCCGGCACUGUCGACGACUGGUAUGAUGGGUUGACGAGUGAGGGCGUCUAGCUCGAUGAUGUCGGUAGCGACUUCACCACCCUCCCCAGCAACGGCAUUUACAAGAACAGAAAAAACGUCACCAGUGAUGGCUGUGUUGACGAGACGAGGGAUGAAGACAGAAUGUUAGCCCCGAGUAUACGGA  >Ss-AA_clean.1_(paired)_contig_17728  CGGGUUUCGAAAGGCGGCCCUGCAGAGUAUCAGCCUCGACGAGCAAAGCCCCCUCGCGUACGGUGUUUGCCCCGAGUGCCUUGAGAGUAACGGAGACAUUUUUAGCUAUGUCAGGGUUUAGCACGUAGUGCGCAGCGAUAUCCCAGCCAGAUGAAGAAAAACUAUAGGAUAAGGAAUGACCGUAUAUGAGAGCAUUAAUGAAAGCAUCUUCGUGCAUACGUCCAGCGCAACGGAUAAUCAUCCCGAAAAGCAUGCGCCCAUUACCCAUACGGACAAUGGAGUUAGCAAGAUCAGGAAGUGAUACGUUGACUUUGGUAUGGGCAGCGGCGUGACGUUUGACAGGAAAACAAUAAGAAAGGAAAUCGGCGCGAGUGUACACAACAGCUAAAUCAGAAGUUCGAUAUGGUGAUUCAAGGAUAAAAGUAGAAUCGGAUCGAGUAGCGGAAAGAAUGUAAUUGAAUGGGACGCCAGCAGCGCGUUUACCAAUGGUUAAUAUUUGCGAUAACGAGAGUGGGACCUGCAGCGGGAAAGGAAGGGCGAGCAAAGAGA  >Ss-AA_clean.1_(paired)_contig_9550  CGGGUUUCGAAAGGCGGCCCUGACAUAGCUCAUCAAACACGCACUGUUGGACUUGGGUUGAGUGGUGCGAGUUGAAGUCAUCAUAAUCGAGCAUGAGAUUAACCCCGCCGCCGCGCUGAGCACCCUGGACACGUUUUGCGAUACCAAGGUGACCACCGACACCGGGGUUGAGUACGACACGUUCCCCACGCCAAACCUUCUCAGCAGCAGAAAGGAUCCAGGAGAAAGCGAAGUAAGAGUUGGUAUCACAAGCGAAAAUGGCACGUUCUUUACCGUUCUCGAGUUUGAUGCUCUUGGACACGUUAGUUCUGGAAUCCCAGGCAGUUAGUGGUUCACGUUCGAGGGCCUCAGAUGCCAUACGGCGGUAGUCUUGGUCAUGGGUCACAGCAUUACGCUUCGGGUCUAUACCCAUAGCGCGUGAAGAUGCAGUAGUCUGAGAUCCAUUUACACACCAAAGCCAGCGAGCGGACCACCAUUUCUGGAGGUCGGGCAGGGUCUUAUCGCGGUCGCGCAGCUCCAUAUGUAGGAUAGCACGGAUAUGAGAGCGUAGCUCAUCGGAAUAUGGAAUGACUUUCUUGGCAACAAGGUCAGGGUCACACCGGUACGAUGCCUCGAGGUCAAGGUUGACGGGUUUCGAAAGGCGGCCCUG  >Ss-AA_clean.1_(paired)_contig_5680  GGGGGUGUAUGUAAAAUACACAUGUAAUGUAAGUUGAUCAGGAGAAUACAUUAUACAGAGUGAAGAUGUUACCAGCGGUUGUGCGUUUAGAGAGAGACGCAGCGUCACCGAAAGGGAGGUGACCGAUGAUAUUCUUCGAGAGAGAAGUCCGACCGAACGCUUCCUCACGUAAGUCGCGGGCAGCAGGGUUUCCACCAGCGAGAGUGAUGAGUUCGCGUAAGGAAGCGGGGUCGAGACGAUUCUCAAAGAGGCGGAUGAUAGGAUAUUGGGUGAGGCACCCCCUUUCUAGCGUUUGGUCAAGUAGUGAUGUCGCACUUACGAAACCGCGAGCGAGAAAUACAGCGGACCGCUUAAUAGUAGGUCGAGUUAAUGCUUCCUUAAAUUUCGAGUUUAACCCCUUAGUAUAACUAGAAGUAACAAGCAAAUUCAAAGCAUCAACACCAACCAGACUAAUAGCAGUUGCUUCUAUUUCCGAAAGGUGGGAGGUGAGGUAGUCAACAGACGCAUGCCUCGCCCAGGAGGAGGGGACAGGUAUUGAAUCCGGAGUCGCGCGCUGGAUGGAAUAAUUCUUGAUCCUGUUGUCGGUGUUAUAGACAGGAGAGCCUUCCAGGGCAGCGGCACCGGAGAGUAAUGAACGGACGGUUUUGUGUGAAAAACCACGCGCGUAACGAAUAGCACGGCAAAGGAGUGUGGGGAGCGAUUGACAGCCAGAGCGGUUGAUCAGGGUUCGCGUACCGGAAAUGGCGGAAACAAGCGCAUCGUCCGGGGAAAGAAUAUCCUGUGAUACCCAAUUACCCGAGACGAAGCCGGCAACA  >Ss-AA_clean.1_(paired)_contig_661  GGUUAUUAUAAAACCGACUGGAUAGACCAGCGGAACCAUUUUGAAGUUGAGGAAACGAUUGAAAAUGGGAACCACCGAGUGGUGGAUUUGAUAAAAGGAUAAACCAGUUUAUUUUAACGCCUUCAUUGGCGCAGCGUCGAAGAAACUAAUCUCUUGAACUCACAACCAAUCGAGUUGGGUGAAUUCCGUAGCUCUCUGAGAUACGUUAAGCGAAGUGGUUAACACCACAUUCAGCUUAAUCGGUCCGAAGACACCAGAAAGCGGGAAACAAGCAAGAAGUGAGUACUCGGGAGCAGCCUGAGCAAGUAAACUCUUGACGAUGGUAACGCCAGAGACAGAAGAAACGGUUGACAAUUGGAUUGACGAGCCAAUCGCUUGAGAGCGAUUCGCAACACCAGAGCCACACGAGAAAAACUUUGCAUAGGUUGCUCGUUUCCGAGCAAGUUGAUCAGUCAUAGCUGAGAAUUUGUCUUUAACAUUGAUUGACCAGGAUAGGUCAUCCAAUGCUGGGGUUGGCCAUUCAAGACCGAUCUCAGCUAAGAUCGAGGAGCGGGACGUGUCACCUACGGCGGUGAGGAAUGAUUGCGGCGUUGAGUAGCGCGCAAACAGAAAAUCAAACCGAUCACGAGUAUCGGGUUUUCCAUCACCAGGUGGAUCGCCGAGCAGAUUUUUGAUGCCCUCCCAGACAUCACUGCUACCAUGAAAAAUGGAUUCAGCGAGGCGUUCUACGUCUAAAGAAACACCAGAAGAAGAGACAAACGAGGCAAGAGCAGAAGCAACAAUGAAGCGCGAAUUCUUACAAUCCGCAGAAGUUGGUAACCAGAAGGUAGAACCUAUCCGGUCAGGAGUGCUGCUUGUGGCGGCGGAAGCGGCGCGAACCAAUGCUUUCACCGUCUCUGCAUAACCUGCAAGCAGGUAACGGGAGCCGAGAGUGUCAGACGAGAAUUCACCAAAAUGAGCGAGGAUAGACCUUACAGCUGCAAAAUUGGUUACGUCAGAAGAGAAAACCGGAGAGAAGUCACCUUGGGGUAAACCCAUAUUCACGUGAGCGUGAACGGUUUGCUGAGCCAAGCCAAGAAAGAAAGCAGAAGUGAUUUCAGUUUCGAAGUCACCACGAGAAAGUUCAGUGUGUGCUGAAAACUCAGCAAAUUUCGAGUUGGAUUCGUAUCGCUCGGGUAGACCAGAAGCGAUACGGCCAAGGUUAUCUCCAGAGUAUGCGAAGAACGCAUCUCUGGUAGGUUCACCAGCACCAGACGGGAAAACGACAGGGAACUUACCGGGUUGAGGGGUAGGCAUGGAUGGGACAGCGGAAGAAAAGCGGGAGGCAGCAGAGACAGAAGAGGGAGCAGUUUGAGCGGGUUGACCGCCAGGCAAGCCUUUGGCCUGUUGUCUUUGCAUCUUGCCAGGACGCUUCUUCUUUCCGGAAGAGGAGGCGGACUCGGAUGGAGUCACAGUUGACAUGUUGUAAGUUUGUAAAAUUUUAAAGUUUUUGAACUUGAGCAAUUUGUAAUGAAGCAGGAAAUAAACUGGAUUAACCAGAUCGGAAC  >Ss-AA_clean.1_(paired)_contig_904  UAUUGUUAUUAACUUCUAACUGGAUAAACCAGUGAGUAGGACACUCUAGAUGGAUAAACCAUGAUUGUGUUUGUUGUCGCUUAAGACGAAGAAAAGCUAGUAUUAUUAGAUCGUGUAAAAUAUCGAUCUCCAGAAUAAGGACUUCUUCGAUGUCCAGCCUCUGGGGGCGCUGCCGCCGUGGAUGAUUUCGAGCCAUCUUCUUUGAUCCUUUGUGAAAGUGCCAUCCGUCGGACACUCGUAGCAGGAUUGAUAAAAUGUCAUGAAUCUACAGAAUUCAUGGCUCCACAUCGCACCACCUAUCCAUAAACCAACCAGCCGAGAUAAACUCGUGCCUAUGUCUGGAGGUGGAUUUUCAGGAUAAAGCAAAAGCUUGAACCAAUCAUCGUCCUCGCGGUAUGCGCGUUGGUUACGAUAAGUUGUUCCAAGCAGCUUGAACUCGGUCGGAUCUUCAGUCACAUCGCACUUCUUGACAUUUAAGUUCAUGCCUAGUACGUCUGCAUCUUUUUGUGACUGUUCGAGAUCAAGUGCUUUACCUGACCUGAAAGCUGAGUCGUCACCUAAAACUUUCAGGUUUUUGGCCUCCACUUGUUGACACUUCGUUAAAUAUUCGACGAUAAUAUAGUUGACUACUGAGUCAACUAACGAAGUGAACCAGGUUCCAGAUGGUACACCGCGACGUUUUCGAAACAUCCGGCCAUCUGGCAUCAAGAUGGGUGUGUUGAUAAAGUACCAAACCAUACCAUCCCAUACGUUGCGCCACUUUUGGCGAGAUCGUUUUGUUGUGGGUUUACCACGCCAAUUCAACCAAUCAACGUUUUGGUGCAGUAUGUCAAAUGCAGUGCGGAUUAACCACUGUGGUACACUUGAAUCAAAUGCUGAGAAGUCCAAGCCGUGCAACUUCUCCCCUUCUCGGUAAUUCACAAUCCACUCGGUAUACAAGCGUUGUGAACUCUUCCCGCAUAGCAUCGGCCCAUUGGGCAAGCUCAUGAACUGAUGGUACAUCACUGGGGCCCAAAGGCCUUCAAUGACUAACAUCUCUGCGGGGUAGAUCCACGUAAGCCGUGUCUUCGGAUCAUCUCUCGGAGACAGGUGUCCCCGCUGUGCAGCCAAACACGGUGGGAACCGAACCUUACGUGGAUCGAAUCGCUCUUUACCUCCUUGCUUCAUACGGUGUCCAAGCCAACGUGCCUCAGUGUAGAUCUGAGCAGCCACCUCUCCUUUCUUCUUGCCUGGGAAGGUCACUCCUGCUGAAGUGUCGUGGCGCAUAUACUGGCCAACUUCUUCCCACUCAAGAGGUUCGUGCUUUACCGGUAAACGGAAAGCCGAGCGAGCGGAUUUAAUUGCUGAGCGCAUACAAUUGCGCUGAUCAAAAGAUAAACUCUUGAAUUCGUUAACUUGUUCGCCGAAGCGGAACAGUGCUUUGUACAUGCCCUCGAGACCGGCUGGUCGUCGGGUGAAACCGUAAAUGCGAUCAUAUCUUUCACGGUCAUGUAUUUUUAGUGCCAAGCGCACCCAAGGAUCUGUGUUACCAGGAGCUGAGUAAGUGGAGUAACCACCAUACCUAGCUAUCUCCUUUAGGCCGGGCUGUUCUAUAAAGUCGGUGUUAGCGGUAACUCUUGAACGGGAUGAAGAACUAGCAUCCCAGAGUAGAAACGUUUCUUUGUUGUCACCAAGAAACGGUUCAACCGCAUCGACAGGGUCAUCGUAAAUUACAUUUUUAUUCAUGUAUUGUUUUCGAAAAUGAAGAAGGAAAUAAACUGGAAAAACCA  >Ss-AA_clean.1_(paired)_contig_1941  CCAACUGCAUGUUGUAAGCUCUCUCACACAACGUUUUGCACCCCUAGUACCGUAGUACUGCUGUGUAGUCUGCUAUCCAUCUAGGGAUGGACAUCUGCUGACUAUCGUUGUUGUUCACAGUCAACCACUGUGUGUAUAUACCUUCCAAUUCCGGUGCCAUCACCGGCUUAGGUAUAUAAUCUAUCUGCUCUCCUCUCAGCACGCUUGCCGACUUAAACGAGUGUUUCACCGCUGUCACGCUCACAUCUAGAUCGGCCGCUAUCUUCAUCUGGACUGCUUCGACACUCUCCGCGCUCAUCAAGUGUGCCAGCUUCGCCACUUUCGUCACCGUACUUAGUAACUUAUCCUCACUGACCCGUAUCUUCCUGUACUGUUUCUCAAUGUUCUCAGGCCCUAUCAGCGGACCAUUGUUCCACCUGUCCCAGUUCAUCGCUUCUAACCACUUCGCCUUCUCUAAUUGCGCUCCCAGCACUCGCAUCUUGAGCACCUCGUGCUCUGCCCCGACAUUCGUUAUUCCUUCUACUAAGUUAGAAGCUGCGACUUGCGCCGUAGCCUUGAUUUUCCCAUUGUGCUUCCAUACCACACCGUAUUCUCUUUCUAACGUGCCGACGUAGUCGCUGGAAGCUUUAAACUUGUUAGCUACUCUUCCUUCCACUAAGCUGUCUGCCAGUAUCUGCGCUACGUCCACUUCUUCUGCUGCUUCUUCUGCUUCUACAGGCAGCUUUGCUACUCUAUCAGUAGCGGUGUUUAAGGGCAUUAACCCUAAUCCGCCACUCCUCUUGCUCCCGUACAAGUACUUCAGGCACUGCCCGCGAGACACCUGGGUUGUGUACCCUAUCUCGUAUAUUGCCAGGUCUUCGAACACCUUCUGCCACUUCGCUGGAUCCAAGCCUCGCCUCACCAGCAUACAAGCUUGUGAGUACAUCUCGGUUAUCCGCUGCUGGCCCACUGAUGCUUGCUUGUUCUCCACGUUGCCAUGGACAAAACUCGCUAGCGAUCGCACGGGGCAUCCUCUCACUCGACCAUCCACGUCGUAUAUCAAUCUAAGGUACUCACCUUUCCCCUGUCCAAUGAGCUGUUUGAUGGCGUUGAUCUCCAUCCCUCCCAUUCUAGCUGCUACUACGUACAUCUCUGCUUCUUCCUUCGUGCUGAACUCUACCCAACCAUCAUCACCCAGCACAUACGUCUGCAAACACUCGACCUUCUGACCGAGGUUGUAGCAAUUCUUCAUCGCUAUGUGGUGGUACGUGAGGUUUAGGAUGGUAUUGAUCAGCGUCGUCUGCCUUACUCCCGAAAACAUACCGGACACUGCUCGCACCCACUGUUUGUUGUCGGGGCGUUGAAACCACUGGUUAGUGAAACUAUCAGCGUACCACUCUGAUAUUCGGUUGAACUCCUCGGUAGGCUGGAAACCCAUCAUCUUCGCCCACUUGUACAGCGUGCGUAUCACGCUGCGCUGUGCCAUACCUGAGUGUUGUGCAUUGAAAUCGGCGUAGUCUAGGCAUACAAUCCAACGUCCAAAUCUACACGAUAUCGCUCUUUGCAUGGUUUCAGCUAUCAGCCUACUAUUGUCCUUGUUCAUGCUAGCGCUUGCCAACGUCACCGUGUCUUCCAAGUAUGUGCUCCAGAAACUGCCAAGCACGUAGUGCGCAUACAACGAACCGUAGAUAGCUCUCACUUUCCCGUGUUCGUUACCUUUCGUGUGCGCUUUAGCCAGGUGUAUGGCCAUGUCGUCUAACAGUGCUAGUACUGCUUGGGCUGACACGUACUCUGCGACAUCCGUUUUUGUCGCUCGCUCUCUCAGCUCUACGCCCAAUCCUUUGACUUGACCAACGAUAUCCUUGUAUUCUGAGAUCAACAUCCUUUCGCCAGACGCUGACCCCUUAAUCAUCCAGUUCCCUCUGCUGCUGUAGAACUGCUCAAACGUUUUCGGCUUCUUAACUCUGAUACUACCCUCUUUGAGCAGCAUCUGGAGUAUCUCUUCUUCCAGCUGACUUUCGUCUGCCGGCACCAUCUUUCCGUUUCUUGCUAUUUUGAUUUCUCCUACGUUCCGCGUUCUCUUCUUGACCUCAUCGUUCCAAUCACACUCCCGGUGCGGUCUUCCUACCAUGUUUGCGAUGUACAAACAUUGUACAAGAUCAUCAGGUGUGUGUUUGAACUCACCUUUUGCCCACUUAUUGCGGAUGAUGUCAUUGUACGAUGCCCAUUUGCUGUUCCAGUCUGAGUAUUCAGUGCUCAAGAUCCCUUCUUUGAUUAGAUCUUCGAAACAGCUGUCGGUCAUGGUAUUCGUCAUCACCCACAAUCCAAGGGUUACCAUCCAAGCGUUUUUCCCGUAAGCGCCAUUUAGCAUCGCUGUUUUGAGGUGUGCAAUGCGCGUCUUGUUCUUCGCUAUGAUCUGCCUUUUCGACCACAUCACUCCAGCUGUGUAGGGGGCGCGCUUAAGCGCCGCGUCCAUACCUCUGUCGUAGUAGCGAUCGAUUUCCUUCCUGUCCUCAGCAGAAGUUUGCUCUUUCUCUAGCAAGAUCCUGAGUCGUCCUAAGCUUAAUGGCCUGGUGUCGAAACUCUUCCUCGCUUCAGCGCCUUUCUUCAGCGCAGCCUCCUCUUCUUCGCGACUCGCAACUCUAAGGUGCCACGGAGUUGGUUUUUUAUAACCUCCGCUCAACGUGCGCCUCACCUUAGGUGUGCUGUUUGCAACUUUCAUCGGUGACUUCCCCAUUGGCAUGGUCGUCAUCCUUGUCACUUGCACUGAUGCUGGACGUGCUCCGCCUGUCACCGGUAGGAACCCUUCUCUGUCAGGCUUUAGCGUUUUCUUAACUGCUAGGACUGGCACCUGCUGUCUCACAGGCUUUGUCCUCUUCACAGUAGUAGGUCUGACGUUGACCACUUCUUCUCUAUUCUGUUGGCUUUUCUCAGGUUGCGGUGCCCAGCCUUUCUCUUCUCUGUGUGAUGCAAUGCCAAGCGCCUCUUCAUCUUCUCGGAUCAUCGCCUCGCUGUUCACCUGCAUCCUCUUUUCAUACAGCUGUGCAAAUAGCCCUGUUGGUGGACCUAGCUCGUUGCUUUCGAGCUGGAACUCCUUCACUCGCUGUGGCGAGUGCUUGUAGGCCACUGCUGUUGUGGACGCCCCCCACAAGACUGGGGCUCCGCGCAGGUAGUCUAACCUGUUCUUACCCAACCACCCCCUCACGACAGGAGUGUCUGAGACAGACUCGGAUGGUAGUAUCUCGUGCUGGUAGUAAGUUACUGCCCCUACGUUCUCCUGUGCUACUUUCUGCGUCAGCCAGAGACAGGUUAGCUGUUCUCGCUCAGUGCAGUAAUCUUGUUGGAGGAUCUGUUUCCUCCUGCUCUCUGAAAAGACAACUUGCCACCCUUCACCUUCCGGCGUUUCAUCCAUGCACAAGUAGCUGCUAGCCCUGAACUUUUUACGGUCCAGGUCGCGACCAGUUGUGCAGUGCUCGAGUGCUAACACCGAGUCAGCCAAGUAUUGCGUGAGCUCGGGAAAAGUAUCCCCGAGCAACUUCUCGACAAUUGGCAUUAUCAGGUGAGCAUACCCGUGGUACAAAUGCCUACCCAGCUCUUGUUCAACGGUGCCAGUGUACGCUGGAUUGGUAUACUCGAUACCGGCCUCUACAACGGUAGACACAAUAGAAUCCGUUAGCACUUCUUCUGCCAUACGCGCCGCUGUUACCACAGACGCGUGUAGGGAGGUUUGGUGGUCACGUUCCCCAAGUAUCACACCCCUGUGCUCAAUUGAAGCUGGGUUCGGGAACAAGUUCAAUUGUAAAUCAGCUGUGUGCAUUGGUGCUGGGUACUGCAUGUUAACGGACGUCGCAGGGUGAGCUAGUAGUGCCCACGCGAAUGCUCGCAUUACUGGGAACUGCUGCCACAAAUUGUGUGCGGUCAGGUACCUGCUCACCAGCGACCUCGCUCUGUAUGCAUCCACCUUAAAUGCACCAUCGUCAUCGCUAUCUCGCUCUUCUAUAAGCUCGCUGCCGUUCAGUGGGCACAAUGUGAAGCGCUGCUCUGGUACGUACAAAUCGAUUGUCGAGUGCACGGCCAAGUCUGGUGCUCCAGGGCCAGCUGUAACGUAGGUAGUAAUUCUAGAAUUACCAGCAAGGUAGUACAUCAGCACAGCAUCACUAUCCGCUGAUUGAUCAGGUAGCGUGAUCAUGUUGUAAGGCUGGCGUGCCACAACGGAUCUCGCCACUUUACGGAUUGGUGCUUCAUCUCCAUCCUCAACAGGCAGAAGGAAUCGGUUGAAACCCUCUGCUGUCAAGUCGAUGUACGUCGCUGCUCCUUGCUCAGCCAGCAUCUCCAAUGCCAUCAACCUCAUGUACAUCGUGCAUCCUAGCGCGAUGUGAUUGUUUCCUUUGCUUGACGAGGUUAGGAUCAAGCUGUUGUAUGCCGAUGUGAGCGCUUUGUCUUCCACAGCCGACAAAAUGUCGGACAGCGGUGUUACGCUGGCAGUGGAAUUGAAAGGCUGGGCGCGGUUGUACACGCUCAUGCCAAAUGGCACGUUGUACGCGAUUGUGUCGCGCGCGAACGGGAUCAUGACAGUUCUCCUGUCGGUAGUGCACUGCACGACCUCGUAACUAUCGAUAUACCCAGCUUUCGUGAUCGUGGCGUCAGGUGUGCCCUCACCGUAACCCCAGGUCACGUGUGGAGACAAGCUCAUAGCCACUGUUUUGUGGUGUGCUUUGACAUCUCUCAUUAUAUCACCGCCUAUCGUGAAAACGUUGUUCCAGUGCCCUUGUAGCGCUGGUUCCCACGCUUUGUGUCUAGCACUACGCAUGAUAGUACCUGCGUACCUGUCAAACAUAGUAUCGUCUUCGUCGUUUAACCACGACAACCACCCAGCAUGCUCUGGUUCUUCGACAGGCUGCCCAGAUCUCUCGUUACUAAGCACAUUCUCGUACUUAUUCCCAGAGUUUUCUUGUUCAGCCGCAGCCUCAUCACCGGGGUUAACUGGGGUGGGAGCAGCUACUGGGAGAUCUGGUCCAGUAACUUGGUCCGGGACAGUGUCAUCUCCCACAGUCUCUCUGUCCAGCACCGGCCCAGAAGGUGGUGGUGGUACAGGGUCAGAGCCUGGUUCGUCAGCACCCUUAGGUCCUAACACGGUCAGCUCUCUUGAGAAAAAGAGCGCCUUAAGGCAGAGAUCUUCUUCUAAUACCACCUUCUCAGCGCAGUAUCUCAGGUAAUCUCUCCAGUCGGAUAGAUCCCUAUCUUCUCUAUCUUUCUCCUCCAUCUCUGACUCCGUUGGGUUUGGUCCGUACCUGCCCUUCGUCUGCCGAGCAUAAGUGGCCAACAAAUCCUGCCUUAACGCCUCUUCUCUGUCUUGGGUUGUGACCCACGACAUAGAGCCGCUCUCGCAGCGACACGACCUCGUGCCGUUGUAGGCACAGCAGAUGAAACUCCCAACCUCAUCCUUCUUUACUACGAGGUGUCCUACCGUCGACCUCAUGAAUGCCUUGCAACCGCAGGGCACUGCGUACACUCUUCGAGCAGCAGGACACGGGUACUUGUACUCGUCAGCUGCUCUCGACAUCAUGGCAACCACCAAGGGGUGGGAGCUCACACCGACGCACUUCGACACGACAGCGCAGUCGUACGACUGCAAACUGUUCGACACCAAGGCCCACAAUUCAGCAGGCAAGUUUAUGUUUUGUGGAGCUGAUUUAGCAACAUUCUCCACU  >Ss-AA_clean.1_(paired)_contig_1940  CCAACUGCAUGUUGUAAGCUCUUCCACACAACGUUUUGCACAUCUAGUACCGUAAUACUGCUGUGUAGUCUGCUAUCCAUCUAGGGAUGGACAUUUGCUGACUAUCGUUGUUGUUCACAGUCAACCACUGUGUGUAUAUACCCUCCAGUUCCGGUGCCAUCACCGGCUUUGGUAUAUAAUCAAUCUGCUCUCCUCUUAGCACGCUCGCCGACUUGAACGAGUGUUUUACUGCUGUCACACUCACGUCUAGGUCAGCCGCUAUCUUCAUCUGAACUGCCUCAACACUCUCCGCGCUCAUCAGGUGGGCCAGCUUUGCCACUUUUGUCACCGUACUUAGCAACUUAUCCUCGCUGACCCUUAUCUUCCUGUACUGUUUCUCGAUGUUCUCAGGCCCUAUCAGCGGACCAUUGUUCCACCUGUCCCAGUUCAUCGCUUCUAACCACUUCGCUUUCUCCAACUGUGCUCCUAGCACUCGCAUUUUAAGCACCUCGUGCUCUGCGCCAACGUUCGUUAUCCCUUCUACCAGAUUAGAUGCUGCGACUUGCGCCGUAGCUUUGAUCUUCCCGUUGUGCUUCCAAAUCACACCGUAUUCUUUCUCUAAUGUGCCGACGUAGUCGCUGGAGGCUUUGAACUUGUUAGCCACUCUUCCCUCCACCAAGCUGUCUGCCAGUAUCUGUGCUACAUCCACUUCCUCAGCGGCUUCUUCCGCUUCCACAGGUAGUUUCGCUACUCUAUCAGUAGCGGUGUUCAAAGGCAUUAACCCUAGCCCGCCGCUCCUUUUGCUCCCAUACAGGUACUUCAGGCAUUGUCCUCGAGAUACCUGGGUCGUGUACCCUAUCUCGUAUAUCGCCAGAUCUUCGAACACUUUCUGCCAUUUCGCUGGAUCCAACCCUCUUCUCACCAGCAUACAAGCUUGCGAGUACAUCUCGGUUAUCCUCUGCUGACCCACUGAAGCUUGUUUAUUCUCCACGUUGCCAUGGACAAAACUCGCUAGCGAUCGCACAGGACACCCUCUCACUCUACCAUCUACGUCGUAGAUCAGUCUAAGGUACUCACCUUUCCCUUGCCCAAUGAGCUGUUUGAUGGCGUUGAUUUCCAUCCCUCCCAUUCUGGCUGCUACUACGUACAUCUCUGCCUCUUCCUUCGUGCUGAACUCUACCCAUCCGUCAUCACCCAGUACGUACGUCUGCAAGCACUCGACUUUCUGCCCGAGGUUGUAGCAAUUUUUCAUUGCUAUGUGGUGGUACGUGAGGUUUAGUAUCGUGUUGAUCAGCGUCGUCUGUCGUACUCCCGAAAACAUACCAGAUACUGCUCGCACCCAUUGUUUGUUGUCGGGACGUUGAAACCACUGGUUGGUGAAACUGUCAGCAUACCACUCUGAUAUUCGGUUGAACUCCUCGGUAGGCUGGAAACCCAUCAUCUUCGCCCACUUGUACAGCGUGCGUAUCACGCUGCGCUGUGCCAUACCUGAGUGCUGCGCAUUGAAAUCGGCAUAGUCUAGGCAGACUAUCCACCGUCCUGACCGGCACGAUAUCGCUCUUUGCAUGGUUUCAGCUAUCAGCUUACUAUUGUCCUUGUUCAUGCUAGCGCUUGCUAACGUUACCGUAUCUUCCAAGUAUGUGCUCCAGAAACUGCCGAGCACGUAGUGCGCAUACAACGAACCGUAGAUAGCUCUCACUUUCCCGUGCUCGUUACCUUUUGUGUGCGCUUUGGCCAAGUGUAUGGCCAUGUCGUCUAGCAGUGCCAACACUGCUUGGGCUGACACGUACUCUGCGACAUCCGUCUUUGUCGCUCGCUCCCUCAGCUCUACGCCUAGUCCUUUGACUUGGCCGACAAUCUCCUUAUAUUCUGAUAUCAGCAUCCUCUCACCUGAUGCUGAACCCUUGAUCAUCCAGUUCCCUCUGCUGCUGUAAAACUGCUCAAACGUCUUUGGUUUUUUUACUCUGAUGCUUCCCUCUUUGAGCAGCAUCUGAAGUAUUUCUUCCUCCAGCUGACUCUCAUCUGCCGGCACCAUCUUCCCGUUUCUCGCUAUUUUGAUCUCUCCCACGUUUCUCGUUCUCUUCUUGACCUCGUCGUCCCAAUCACACUCCCGAUGCGGUCUCCCUACCAUGUUUGCGAUAUACAGGCAUUGUACCAGGUCGUCGGGUGUGUGUUGAAACUCACCUUUCGCCCACUUGUUGCGGAUAAUGUCGUUGUAUGAUGCCCACUUGCUGUUCCAAUCUGAGUAUUCGGUCUUCAAGAUCCCUUCUUUGAUUAGAUCUUCGAAACAGCUGUCGGUCAUGGUGUUCGUCAUAAUCCACAAUCCAAGGGUCACCAUCCAAGCGUUCUUCCCGUAUGCGCCAUUCAGCAUCGCUGUCUUAAGGUGUGUGAUACGUGUCUUGUUCUUUGCGAUGAUCUGCCUUUUCGACCACAUAACUCCUGCAGUGUAGGGAGCGCGUUUGAGCGCCGCGUCCAUACCUCUGUCGUAGUAGCGAUCGAUUUCUUUCCUAUCUUCAGCCGAGGUUUGCUCUUUCUCUAGCAAGAUUCUGAGCCGCCCUAAGCUUAAUGGCCUGGACUCGAAACUCUUCCUUGCUUCAGCGCCUUUCUUGAGCGCAGCUUCCUCUUCUUCGCGGCUCGCGGCUCUGAGAUGCCAUGGAGUUGGCUUCUUAUAGCCUCCGCUCAACGUGCGCCUCACUUUUGGUGUGCUGUUCGCAACUUUCAUUGGUGAUUUGCCCAUCGGCAUGGUCGUCAUCCUCGACACUUGCACUGAUGUGGGACGGGCUCCACCUGUUACCGGUAGGAACCCUUCUUUGUCAGGCUUUGGUACUGCUUUUACCGUUAGGACUGGCACCUGCUGUCUUGCAGGCUUCGUCUUCUUCGCAGCAGUAGGUCUGACUUUGACCACCUCCUCUUUCUUCUCUUCUAUUCUAGGCUCGGGUUUGGGUGUCCAGCCUUUCUCUUCUCUAGGUGAUGCAGUACCCAGUGCUUCUUCAUCCUCUCGGAUCAUUGCUUCGCUGUUCACCUGCAUCCUCUUUUCGUACAACUGUGCAAAUAGCCCUGUUGGUGGACCUAGCUCAUUGCUUUCGAGCUGGAACUCCUUCACUCGUUGCGGUGAGUGUUUGUAUGCCACUGCUGUUGUGGACGUUCCCCACAAGAUUGGGGCUCCGCGUAGGUAAUCUAACCUGUUCUUGCCCAACCACCCUCUCACAACAGGAGUGUCUGAGACGGACUCGGAUGGUAGUAUUUCGUGUUGAUAGUAAGUCACAGCCCCAACGUUCUCCUGCGCUACUUUCUGCGUCAACCAGAGACAGGUCAACUGUUCACGCUCAGUGCAGUACUCUUGUUGGAGGAUCUGCUUCCUCCUGCUCUCUGAGAAAACAACUUGCCACCCUUCACCUUCCGGUGUUUCAUCCAUACACAAGUAACUGCUAGCUCGGAACUUUUUACGGUCCAGAUCGCGGCCAGUUGUGCAGUGCUCAAGUGCUAGUACCGAGUCAGCCAAAUAUUGUGUGAGCUCGGGGAAAGUGUCUCCGAGCAGCUUCUCGACAAUCGGCAUUAUCAGGUGAGCGUAUCCAUGGUACAAGUGCCUGCCCAGCUCUUGUUCUACAGUGCCGGUGUACGCUGGAUUAGUGUACUCGAUACCUGCCUCUACAACGGUAGACACGAUGGAAUCCGUUAGCACUUCCUCUGCCAUCCGCGCUGCUGUUACCACAGACGCGUGAAGGGAAGUUUGGUGAUCUCGCUCUCCAAGUAUCACCCCCCUGUGCUCAAUUGAGGCUGGGUUUGGGAACAGGUUCAGCUGCAAAUCAGCUGUGUGCAUCGGUGCCGGAUACUGCAUGUUAACAGAUGUCGCAGGGUGGGCUAGCAAUGCCCAUGCGAAUGCUCGCAUAACUGGGAACUGCUGCCACAAAUUGUGUGCGGUUAAGUACCUGCUCACCAGCGACCUCGCUCUGUAGGCAUCUACCUUAAAUGCUCCGUCAUCAUCGCUAUCUCGCUCUUCUAUAAGCUCACUGCCGUUCAGCGGGCACAAUGUGAAACGCUGCUCUGGUACGUACAAAUCAAUUGUCGAGUGCACGGCCAAAUCUGGCGCUUCAGGGCCAGCUGUAACAUAAGUAGUGAUCCUAGAAUUACCAGCAAGAUAGUACAUUAAUAUCGCAUCGCUGUCUGCUGAUUGGUCAGGCAGCGUGAUCAUGUUGUAAGGCUGGUGUGCCACUACUGAUCUCGCCACUUUGCGGAUUGGUGCGUCAUCUCCAUCCUCGACAGGUAGGAGGAAUCGGUUGAAUCCUUCUGCCGUCAAGUCAAUAUAUGUCGCUGCUCCUUGUUCAGCUAGCAUCUCCAAUGCCAUCAACCUCAUAUACAUCGUGCAUCCAAGCGCGAUGUGAUUGUUCCCUUUGCUUGAUGAGGUCAAUAUCAAGCUGUUGUAUGCCGACGUCAGCGCCUUAUCUUCUACAGCUGACAAAAUGUCAGACAGCGGUGUUACGCUGGCUGUAGAAUUGAAAGGUUGAGCGCGGUUGUACACGCUCAUGCCAAACGGCACGUUGUACGCGAUUGUGUCGCGCGCAAACGGGAUCAUGACAGUUCUCCUGUCGGUGGUGCACUGCACGACUUCGUAGGUGUCGAUGUACCCGGCUUUUGUGAUCGUAGCAUCAGGAGUACCCUCACCGUAACCCCAGGUCACGUGUGGAGAUACACUCAUUGCUACUGUCUUGUGGUGCGCCUUAACAUCUCUCAUUAUAUCACCACCUAUCGUAAAAACGUUGUUCCAGUGCCCUUGCAACGCUGGUUCCCACGCUUUAUGUCUGGCACUGCGCAUGAUGGUACCUGCGUACCUGUCAAACAUAGUGUCAUCUUCGUCGUUAAGCCACGACAACCACCCAGCGUGCUCUGGUUCAUCUACAGGCUGUCCAGACCUCUCAUUGCUAAGCACGUCCUCGUACUUAUUCCCUGAGUUCUCUUGUUCAGCCGCGGCCUCAUCACCAGGGUUGAUUGGGGUGGGUGCUGCAAUCGGGAGGUCCGGCCCAGUCACUUGAUCUGGCACAGUAUCAUCUCCCACGGUCUCCCUGUCCAGCACCGGCCCGGAGGGUGGUGGUGGUACAGGCUCGGAGCCUGGCUCGUCAGCACCUUUAGGUCCUAACACAACCAGCUCUCUUGAGAAGAAGAGCGCCUUAAGGCAGAGGUCCUCUUCUAAUACCACGUUGUCAGCGCAGUAUCUCAGAUAUUCUCUCCAAUCGGCCAGUUCUCUAUCUUCUCUAUCUUUCUCCUCCAUCUCUGACUCCGUUGGGUCAGGUCCGUACCGGCCUCUCGUCUGACGAGCGUAGGUGGUCAGCAAGUCCUGCCUCAGCGCUUCCUCUCUAUCUUGGAUCGUGACCCAAGACAUAGAGUCGCUUCGGCAGCGACAGGAUCUCGUGCCGUUGUAGGCACAACAGAUGAAACUCCCAGUCUCAUUCUUCUUAACUACGAGGUGUCCUACUGUCGACCUCAUGGAUGCCUUGCAACCGCAGGGCACUGUGUACACCUUACGAGUAGCAGGGCACGGGUACUUGUACUCGUCAGCUGCUCUCGACAUCAUGGCAGCCACCAAGGGGUGGGAGCUCACUCCGAUGCACUUCGACACAACAGCACAGUCGUACGACUGCAAGCUGGUCGACACCAAAGCCCACAAUUCUGUAGGCAAAUUUAAAGAUUGUGGAGCUGAUUUAGCAACAUUCUCCACUCUGUUGUCCUCUUGCGCCGAGGAGGCGGUUUGUGUGUUGUAAGGCAUUUUGAAUUUCAUUCUCCCACUUUACCCUAGAGAGCCUGG  >Ss-AA_clean.1_(paired)_contig_823  CGCUCUGUAAUUACUCCUUCCGUUAUAAUAUGGGAUUAGCCCCUGGUUAACUCAUCCAUUACUACUCACUGCAUGUGAUAGUAUUCGUACGUUUCGAACUUCGUGUGUUCUAGUUCAUUUUUGAGAGCAUUGCCCUCGUCCAGCUUCGUACUGGUGCUGCGUUGUCCGGCCCAGCCGGCGUAACACCUAGCGGAUCACCACCUAGUGGUACCGCCAUCGAAGUAUACUGUGAUAGCCGAUCUGGCAUCACUUCUCCUUCAUCGUGGUUUGCACUCGACAGACUCCAUCCGUCGUCCCACACGCUCUUUUCUUCUACCGCGAGCAGCGGUUGUUCGAACUCAGCAUCGGUGGCUGUAUUCUGCCCUCUGACCAGCUCGUUCACUGCUGCGCCUAUCGACUCUGCUUUCACCCGCUUCCGAUACACCGUCCACACUGACAUAGCUAUUAUCGCGCCAACAGCUAUUCCUCCCAAUGCUAUUGCAGCUACUGAUCCAACUGCUAACGAUGUUUUCACCCUAGCGUCUGGUAUCGUCGGCAGCUCACUCGGCGGUUCUGGUAUGCUCCCAUGUUCGUCUUUAAGCCUUUUGCAGUGCGCAUUAAAACGUGAUAUAGACGUCAGUUUAGCCGCGUUGGUGCCCAUACUGCACGGGUAGAUUGUCACACCGUCCCUUGCUAAGUACACCGUUGCGGGUAUCACAUUCUGGGCAGUGUUGGCUGCCCACUCGGUGAUCUGUCUCUGACUGCUAUUUAUAGACCACAGCUUGAUACCUUUCGCAUUAGCCAGCAUCGCCAUUACUCUGAGGUUGAUCCAGGAUGGAUCAUCUGGGAAGUACCCAAGCUGCUCUUUCAUCCAAUCCUUUGCAUUGUAGAGCAUCCACCUAGCUUCUCUUCUGUACCCGCCGAAGAGCAGAGCACCGGCCAACCGUUCCAGGUUGGACGUUAGCUUGUCCUUCUGAUCCUCUCCUUCUCCUGUGCCUUUCUUGAAAAUCAUGUCCAUCUCUGCUACCGAGCAGACUGACUUGUAGGCUGAAGGUUCGAUUCCCAUUUCCGCUAGCUUGUUAUCCACAGCGGCAAACCACUCCUCUCUACUCUUCGUGCUCUGCGCAGCAGCGUAGGAGCUAUCCGCGUACCUUGUCCGCACAUCCGCCAGAUACGCUGCUAUUCCCUCCUUCCUGGUUAUAGCGUUCGGAUCGCUGAAUUCUCCCAUUCUCAGGUAGAACAAAUUGUUUCCGUACAACAAGAAAGUUGGUUGGUAUCUGCUGUGGGCAGUAUGUUGAGGCACGUACUUGUGCAGCCUGACUAGCCUCAUCACCUCGGAACUGCGGUCGACCUCGAUCACGGCAAACGGUAGUUCUGCGAAGCCUGCUAGUGCUUUAAGCACUGGUACACGCGGAUGCGAGUUCCUAAGAUGAUCUCUAUCCUCGGGUUUUAUCAUGUAGUGAUCGCAGAACCUAUUGAGCGUGUCUUCGUAAUAGUGGUCAUCCGCCCCUUGUGCUAGGUGCUUGAUCAUCCGCUCAGUGGUUAGGCUCAGCUCCACGCCUAAGUGCAGUGUUGAUGGCGAGAUUGCUGUUCCGUCUAACUCCGCUGUGCUGUAUUCAGAAAUCUUGUUCGAUAUCCCUCCGUGCACAGCUGCAACUUCUUCCACGUACUGGUCCCAUGUGCGAGCCCCGUACCUGCUCACGCCGAUCGCCCCGUUUUGGAAUUCCGUCAUCCCUGUUCUGUGCGGGUGUCCGACUACAGCAACACGCGCCUUAUUCUCCCACUUGUAGUGGUCUGCAGCGCUGUGCUGCUUGGACUUAGGAGUAUUUUUUUCUAUCGCAGCUAUGAUCUCAUCAUCUGCUGCAGACAGAGGUGUCGACCUGAAAGGUUUCUGUGCCUUCGGUGUCUUUGCGGGGAAGAGAACUUUGUCUUUCUCACUUCCGCUCGCUCCUUUCUCAGAAGAAUAAGCACCUGGCAUCGCUCUUUCACCGAACUCGGCCAAGUAUUCGUGAAUGGUUUCUCGCAAUGUCUCCUCGUCUGCGUAAUCGAUCUCAGCGUCCCACUUCAUUGCUGUCCAGUGCGUAGGCUGUGACAGAAUCUCUAAGUACCUGUUGUGACCUGUUGCGAUCACUCGCACCUUGUCGAUCGUGGGGUCCUCAUCAUUGGAUUCUUGGUACAUGACCAGACUGUAGCCGUAUUGGCGAGCUACUAGAGCUAGCACUUCAUCGGUUGCCCAAAUCUGCUUGGCUACUCUCCUCUCCUCCAGCCCGCAUACUUGCGCUUCCACUUCGUACACUUCGGUCAGCGAAAUCAUAUCUCCUUUUGCUACCAUCUGUGCGUGUAAAGCCCUAACACCGCACCUCCCAUCGCCAAUCGUUGGCACCACUCUGAUGUGAUCAUACAGUCCUCUUUGAUCUUCCUCAGCUACAACAACUUCGUACUCUUCAAAGUCGGCAACUGCGUACAUCUGGUUGACGUGCUUGUUGAUUCGUGCCGGGUUCGCCGUUAUAAUUUCUGACGCUUUAUUCUCCUCUGGCUCGCUCAUCGUGUCAAUUGCAGCCUGUGCUCUUGCCGAUGCCUCUGAUGCCAGUUCCUCUAAUGUAUCGAACACCCCGUUGUGGUAAGCAUCGUAUGCUGAUCCUGGGUGCAUUGUCCUCGUAUACAUGGGUGCUUGGGCGUAUACCAUUUGCUCGACGUAGAUGUCCUUCCUGGGUGGGGUCACUGGUUGCAAAUCUUUACUGCGCCCUUCUACCAUACGCCCGGCUGUAGCUAAAGCAUCCUUUGCGAAUUCCACCCACGAGUUCUUUCUGGUGCGUUGUCCAGUCGGUCUCUUGAUUUGGCUUACGCUUACAGGCUCAUCCUUGUGCAUCUUGGUGUUUCCACUUGGCCCAGCCUUGAUUGGCAAAUCGGUCUGCACAGUGUUAGCUGCUGCAUCUUGGUCAAAGAAAGGCUCGUAAUGUGCAGCCAACUGUGGUCCGGUUCCCGUGUGGGCGAGCCUUUGUGCCAUGGUGUUGUGAGCUGGAACGGUGCCGAUGUAGAGCAUCUUUGGUGCUCCUUUAAUCGUACCAACGCGUCGCAAUCCAGUAGACGAGACUGUCAUCCUUUGAUCUCUGUUCUUAGUACCGAAGUCUGUGAAGUUGAACAUGAAUAACCCUUCUGUGUCAAAGAUGUUCUUCUUUUCAGAGUACAGCAUUGAGAGCAUUCUAGCCACAGGUUUGGUUACAAACACAGAAGUCGCUUCCUUUACCCUUGCGGCCUCAUCCCAGAUUAAGCUCAUUGCUCCAGUCUCAAUAGUAGUACCUGCCAGCAUGCACGAUGUUGGUCGUAAUCUCCCAGUUGUGAAAGUUUCUUCCAGAUAUCUCGUGAGCAUAACAGCGUCUGUAUGCAGCAACUUCUCUUGCUCAGCUGAUACGUAUCCGAACAUGUGUGAUCCCCAAGCCGACAUCAGUUGAGAAGCUCCUGAUCCGAGCACUUCUGACAGGAAAAUACCAGCUUCUCUUUCUCCGGCGAUCGAGAUCAUUGGUAUGCCCUUGCUCUGCACUCGCUCGAUCAUCUUAGCAGCCACAACGUCGAUGAGUACUCCGCGUUGGAUUUGCAGUAAUGCAAACAAUAGAGCGAACGACUCCCCUUGCAUCAGGGUAAGAGUGGUACCUCUACCUUGAGCCCGCGUAGACAGCCACAGUUCGUACUCUUGCACGUGAUUUGGCUUUGGAAUGCCACCCAUCAGGUUUGAGUCUGAGUUGGAGAUAUCUGAGACAAUCAAUGAGCAAGCAAUCAGUUGCGCGUUGUGCAGUUGAUCUUGCAAACCCAUCCUUCGUACAUAGUCGAUAAUGAUGCACUCCAGACCACCAGAAGUCAUCAGGUACUCGUUCUCUGACAAGUCCACCAAGCUCAAUGCUUUAAGUGAAUUGCACAACGGUAUCCUUAAGUACUCGCGGAUAUCGGUCUGAUAUGUGUAGUACGUCGGUACGAUGCUCAGAUCUGUCUUGUUGUAUCUGAAUUUUGUCGUAUCCUGUAACAUACCACACAAGAACAUCAUAUGUAUAAUGUCAAUUGGGUUCGCUGAAUCUCCUGGUAGCACUAGGUCGAAAGGUUUAGCUUUGAUAGCCCAUUCCCAAGCGUGUGCUACUGCUUCCAUGAUCUUCACUUCAGUUGUCUCGUUCGUGUUCUUUGCUGGCACAACGUUGAGUGUGUACUUCUCAGCUGCACUGCUGCUCGCUCUGACGUGGCACGUCGAGCCUUGUUCCUCAGCUUGUCUUACUGACCACAGCAUGAUAAGCCAUUGCACUAGCAUCCCGAUGUGGUUGUCUCCGAAUUGAGCCUUUACGUUACCGGCCUUGUACCACUCCGAGGGUACCUUCUUUCGCAUAUCACCGAGCACGAUGUAAUCCUUGGCAUCCACAAGCUUAUCAGCAUUGUCGGUUACCCCGGCGAUAUGCUGAUUGGCACUAACGCGGGGCGAUGUGUAUCCGAUGCCAGUGUUGACAGCCUUGAUCCUAGAUUGUGCCGAAGUAGGUCUGUAGCUCCACAAGUUGACGGAGUGCUUAUCAGUGUAAGCAGUACCGACACGCAAUGUGGGUAGCUUGUCCAGGUUGUAGCCCCAACACAGUGCUACGUCGACAGGAUCUAAGGUUGGCUUCAGCUGGGAGAAAGUGUCGGUGAACUUGAACUUACCCAUCUUUGCCAUGAGCGCCCCGGUCAUCGAGAUGAGCCUUGUGGCUGAUGGUUGGAUUAUAACCCGACCAUAAGCUUCGGACUGAGCGAGUCUGUCAUUGACCUUCCAAUUUGUAAUAGCCUCCCAAAGUGUUGCUGAGUUGUCCAAUGUUUGUACGACGGGUGCCAUUCCGCCAGCAUCUUUGUAGAUGCCAGCAGCUGCCGCUCCCGCUCUCUCUGCCUUUGUGGGCAUCUUGUUUGGUUUGAAGCCCUUAGGCCCCAAAGUCGCCAGUCUGCGCUCGUUACUCUCUUUGUCGCUAGUAGCACCGCGCGCGAUAGCGAUGCACACGUAUGUGCACAACAUCAGCGCGUACGCUACAAUGUAAGCUGCCAAACUGGUCCCGCUUACACCACCACCGACCACAACGUUGUCUGCCAUUGAGUACAAUACUUCAGCCGGGAACAAUGUUCCUGGCACGAUAUGGUACGCUACAACGGCAUCUACGUCUUCAGGCGCUAGCAAAAGCAGCGCGGACACUCUCUCUGUUGAGAGCGAGAUCUUGUUCUCCUCCACGUACUUCUUGAUAGCACCAGCUUUUGGCCAUGCUAGCAGAUUGCUAGCUGCCGCCGCUCGAUCCUUCUCCUCAAAGAAAGCCAUGUGGCAAUAUCCAUCAAAGUGUGACUUCGAUGCUGCCAAGGCCCUCUUCGCUUUCUUCCCUGCCUUCACCUUCUUGCUCUUAGGUUGAGCGGUCUUGCGUGGAGAUGAUUUAGCAACAUUCUCCACU  >Ss-AA_clean.1_(paired)_contig_6913  GCAUAAUUAACGACGUUUAUACGCGAUACCGGUUAAGACCCGUUAAUUACUGUUCGAUAUGUCCGAUAAACUUGAGAACCUCCAACUCAUGGGUGAGUUGUCCUGGGGCCGCCGUGGUGCCGCCGAGACCAAGCCCCCAAUGAUCGAGCCAGAGGAUGUCGUCAGCACCGCGUGGGGUGUCGGCGAGGCCGGCGCCGUUGAAAUCGCCGAUCUCGUUGCCACCUGCGUGGGGUUCCUCACCACUGUCGCGCCGCGACAACGUCAGCGCCAGCUGCCCUUUGCACUAUCCAAUGUGGAGGGGGCACCUGUCGUUGACGUUGAGUUUCAGGCGGCCGCCAGCCAUGUCGCCAUGCCGGAGAUGCCAACCACACGCGCCCGCUAUUUCAGAGUGCCAGGGAAAACCCCGUCUGAAAUUUGGGAGUCCCUCCGUGCCCGCGACGAACGCAACGCCGGUGCGAAGCGCAAGUCUGCCGGUGGUCCUCUUGUCCGAGACGCCAAUCUUUUGGGACCCGCGAUGGUCAAGCGUUCUGCCAUUGAGGAUGCUGGCGGUAAGCCAUCAUUCGACAUCGCAUGGAUGCAGAACACCGUUCCGUUUGGCUUUGGUGCCCGCCCCGCCGUGCCUGAGCGUGAUUUGCGUCCGGUGGUCGAGACCGCGAUAGAGCGCGCAUCUCGAUUACCGUACGCACCGCCUGGGUAUCGUGCCCGCCGCCGUGAAGCAGCCCACCUCGGUGACUUCACCACUCACGACCCUGUCACACUGUCCCCCCGUUUCCUGGAGUACGUGAAGCCCCGUGUUGAGAUCGCCCCGCCACGUACCGACGUUGCCCUGCGCUUGGCAUCCGACAUCAUGAUCAAGGUGUGGCAGGCUGCCGGCAUUGCACCUCGUGCCCGUUCCGUGUUUGACGUCGACCCUGAAUACCUGGCCGACAUAAUCAAGGACGGCAAUGCUGGAGAAUACCGCACGGCUGGCAUUUCAUCACGCCGUGAUCCGCGCAUGAUGCACAUGCUCUCCGACCACAUUACGGGCUUUGUGCAUGCCGGUCGUGAAUUGAUGGCUGGGCGCACCGUGCCAUCGUACCUCGGCACCCUCCAGCACCAGACACUCUCCUUUGGCAAAGAGGAAGCCAAGGCAGCGAAGCCCGUGCCUGCUCACCCUGGUGACGACGGUGCUUAUCUGCGUCCCGACGGAACAUGGGCCACACGCGUCGCACCAAUCCCGCGUUUCAUUUUCUCCCCGUCACCGUGCAACUAUGCCGUUGCCGCAUUCCUCCAUCAUGACGUUUCUAAGAGCAUGAUGGACCUCGACCCCACACACGGACCGGGUUUUGGGCCUGGCCGUGGCCGCUCCCACAAGUUCACCGAUCUCGUGACACGGUCGUUUGGAGACAAGAACGUGAUGGUUGAUGAUGAGAUGGUGAUGUCCGACAUCGAGAAGUGGGAUGCCAGCGCGACGGAGGCACUGCUGGGUUGUGGUAUGGAUACGAUGGAACGCGCCGUCGGUAAGGAACAUCUUACCGAUCUCGACCGCAGUACGCGGUUGGCAAUGUACAAGUACGCCAAGCGUACGUUGCUGACCAAGAUCGUCGAGCAUCCGUCCGGCUACAACCUCGUUCUCCAUGGAACCAUGCCAUCCGGUUCGUAUUACACAUCACUCGUCAAUACGGUGUGUAAUGACUUGCUCGCCAUUGGGCUGCUCGUCCGAACCAUGCUCGAUGCCGGCCAUACUGUCGAUGUUGAUGAGUGUGCCGCCACGGUGUCCAGAUGGUUGCUCUCAUACGGUGACAACCAGCUUUUUUCCACGGCGAUGUUCCGUGAGAUGGGGGUGUCAUAUGAUAAGGAAAAACACGCCGCGUUCCUCGCAACCCUCGGCAUGAAGUUGAAGCUGGAUGAGACCGAGAUCACCCAGCAGCUGGGCAGGGUACGUUUCUGCUCACGUGCCGUUGUGCGCACACCGCAUGGGUUGGCCGUUACAAGAUCACACGACCCGCUCAUCUCCAAGCUCGCAGGACGACCCACUGCAUCACCACUGGCGGACAAGCUGUACGUGCGGGCGCUCAUGAUGGACUAUCUGGGCACCGACCCCAUCGCGUACCAAAUGCUGGAGUUCGUGGACCGCCAGAUUGUGCUGCGGCCCGGCGACGCCGAUACAAUUCCCAAGCAGCUGAAACGCGAGGUUGAGGAGACUGCAAUGCACGUGUUUGGGUCUGCCGAUGCAACGGCAAUUGGUGCAGUCGUCGCCAUGGUCACGGCGACCGAUGUGCCGCGAGCCGACCUGCUGCUCCUGCACCUACCGCGCGUACCAGGUGCCGCGUUUGGUGACGUCUUCGGAAUGGGAGUGCGUGGAGGUCGUGUGUUCCGCGGUGCCGGGACGGAAAUGACGCGUUGGUUGGCCGAGCAGACACCCGACCAGUGGUUUGAGUUUUUGACACGGUCGGGCCAGCUGGGGGUGUUGGAACCGUAAAUAUCAAGUUUUAGGAUGCCGGGCAAGGGCGUUGUGCCACCGCCGGAAUGGGAUUUUCCCUAUCCGUUAGUGGUGCAUCAGUACUCAAUGCCGGAUGAGAGCGCGCACUCUUUUCCGGCCUUGAGCUGUCGCCCCCCACCGGCGGGGGGGG  >Ss-AA_clean.1_(paired)_contig_1672  AUGCACGCGAGUUGAAUGUUAAGUAAAAACACAUUCAACGACGUUUGUACUCGAAACCGGAUAAGAGAACACUACCUCAUCGUCAUACGCAGAAUGUCAGACGUCACCCGUCUUACCACGAUACUCGCACGCGCGAAUGCGCACGUUGUUCUCGCUGCCGGUAUCGCGUCAUCUGCCAAUGCCGACUUGGAUAAGAUCUCCGACACGGCUUCGCUGCAGACUGCGUUGCGGUGGUUUGCCGAGUGCCCCUACCCUCACGUCGUCGCGCGCCUCACGCCGUGCAGCAUUGACAACCUCAAAGCUGCACUGCCGGAGAUGACCCUCGCCGACUGGGAGGAUACGCUGUACCUUUUGAGGGCCUUUGUGCCUGCCGAUCAAAGGUAUACGCGGACCGCCCGACGAGAGGUGAUAGACGCACUCUUUUCUCACAAGGAGGUAUCCGGCCUCCACGAAGCACGGCCGUGUCCGGCCGAGUUUGCAGCACUUGAUGCUGCGACCUCGUCCGGAUACUAUGAGUUGCCCAAUACCACGAACCGCAUCAAAAUGUACUGUGAUAUUGGGAUCAUUCGUGAUCUGCGCGCGCACCGUACGCAGAUGCACGGGUCGCUCAUCUCUUUUUCCUGCGACGUGGAUGUCGAUGGUCGUUGCAGUACGAUCAUCACCCCUUAUUGUUUAUCGCGGGAGUCCGCAAUGGUGUAUUUGUCCGUCGUGUUGACAGAUUACCUCCCCGCGAUAAACCGCGCCGUCCAAGCCCGUCGCGUCACCACCGCCACAUCCCGUCUCUCGCGGAUGACCCUCACACAGGUGCGUGACGCCCUCGACAUGGCGCGCACGAUUCUCAAGACGUACAAAUUCAACCCCCAGCGUCUUGAAUUCGUGGAUGAUGGAGGAUCGACCAUCGUGUCCGCCCGCUCCCGUGGCGUUGCGCCGGCUCUCGCGCUGUCGUAUCGUCUCGGUGGCCGUACUCCCGAAGGACCGCUCCGUGGGAGCGUCGACGUUCUUCUCCGCAUGGCACUUGCCAGGACGGAGGCGUUGACGCGUGCCCACGUGGCCCUUCCACCGGAGUACGCGGUGAGAAAAUCGCUGCCUGACGCGUUGCUGCGCCAGGUCCGAUCGCUAUGGCAUGACAGGCAUGUGCGGGAGGCGCUCCCCACCGGCCCGGAGACCAACAAGCGACCUGGCCUCGGCUUAGUUGCGCGUGGUAGUGGGACAAGUGACCGCAACACUGAUGCGGCUUUGUCCCGGUUGGAGGAAGUCGUUGCUUCCCGGCGCGAUGAUGUGAGAGGCAUGCUCCUAGUCCUUGAGUGGGCUGGCAUGUAUGAUCCAUCAACCGUCGUUGCAGCGGCCUUACUCCACGGUGUCGAUAUCGCCGUCGAUGCCGGUGUUCCUAGUGAAGGGGAGCGUAUGGGGGGCGUGAACGUCCCAGCCGGGUAUGGGCGAUACAUGUGUUUCGCAACUACCCGGCUGGGGGAUUUGCCCGUGUCCAGCGAGGUCAUUUAUCCCGCUGGCACACCCCUCACCGCCACUUUGUCCCUGUUGGCCUCACAGUUCCAGCACACGCAUACCCACCUUGGUAUCGUGCGCGGGGGCAUUGCCGCCAACCGUGACAUCACCCCUCUCGCUAGCAUCACCGACACCGCUGGCGCAUACCGUGCCACCGCGGAUGCACAAAUGGCUUUGCCAGUCCGCUUUUUCUCGUCCGAGAUACUUCUGCCUGUGCCCACCUGCCCUCACGCUGGUGCGCCGUUUGACAGUUUUGUUCGCGCCAAAGGCGCUCUCGUGUCGGGGUGUGCAUCCUGUACCGGAGUCAAGUCGGCCAUCACCAUGGUCUACUCCCUCCUCAAGACCACCGGCCGCCUGGUUAAGCAGCGUGCCGCAUAUGGGCAUAACACCCAUUAUACCGUUGAGGUCUUUGAGGGAAUGGAUCCUGGUGAUCGCCUUGCCCAAUCAGUUGCCGCCAUUGAUGCAGCUGUCCGCGUGUCGCAUGCGCGGAAUACGUUUACUGAUGUCGAUGCCGCAUCACCAGAUCUGCAGUCGCCCGCCUUCGGUGACCUGAUGAUGCGCGUCCGCCAGAGUGCAUAUGAGGCACUCAUCGGCACCCAGAACGUAUUGCCGCCCAUUGCGGACGGAGAGCUGGAGUCAAUUGCGGCCAGUAUAUAAGAAAUGCACGCGAGUUGAAUGUUAAAGUUUGGGUUUCCGGCAUCGAGGUUACAUGUCCGUGCCGCCCCGGGGUGUUCCCCCGAGGCGGUGCGGACAUGUUUCCCCCCUGCCGGCUGGGGGGG  >Ss-AA_clean.1_(paired)_contig_889  CCCCGCGCCGGUGGGGGCCAUGGUACCAUAGCCGGCAGGCAGAAAACUGCCAUGCCGGCUAUGGUGCCCUCACCCCCGCCCGGUUUUUGCAAUCAUGUAAAAGCCGGAAAGGGGUGGGACGCGUAAUACGAGGUGGUGUUUGUGCCCACCACCCACGUACACGCGAUGACCCACCACCGGGAGGGCCACGCUCAGGCUCUCACGAACUCGUGAGAAAGCGGAUUAGACGCUCGUAGGUGUUGCCAUACACCCGCCACGCAGAAUGCGCAGCGGCGAGGUCCACCAUGAGCUUGCCCCCUCUGGCGCCACCCUGCUUAUCUGGACGGUGUUUCGAAAACCGCCAGCGUAAACACGCAGCAUCAGAGGGGACAAACUCUGCAACAUCGGCCUCCUCGGACCACGACAUCACCAGUUGAAUUGUCCCUCCAUUGUAGCCCAACCAGGUGCGCGCGAGCGUGAUAUCGUCGAUACCAGUUUCGGAGUGGACAUCGACAGACCACUCGGAACCCAACUUAUAAAAGUUGCCGAGCUGGUCGCCGAUGGCGCAGUUGAGCCCAUCAACGGGCAAGAGGAGUUCUCGCUUGUCGGCCGUGGGAUAGCGCACGACGCCGCAGGUAUAGUCGAACAGGUCCAGCAUAUGGGAACCAUUAACAACUAUACCGGCUGCGUCGAGCCCACCCAGGACACGACGACAAAGAGCGAGACCGGAAAGGGACUGGGGAUUCGUGUGUCGAUCCCCGAGCCCAAGCAAUCCCGUCACAACAACAACCUGGCUCUUACCGGUGACGUCACCACGGACAAGUUCAGCCGACGCGUGAAUUGCAGUCGGAUAAGUCUUGGGUAGACGGAGGGCGAAACGGUAAGUGCCGGAAAUAAACACGGCACCGGGUCCCUGCACGGUCAGCACGCCACCCGUCCCAUCAAACUUGGGAAGGAUUGCGGCCGAGGAAAACUUUCCACGGUCAGAGGGAACCGGUACGCGUUUGGUACCGCGGGUUGAAGGGGGUACAAGCGCAUCACAGUCGGAGUCAAAUCCUGUGGGCUGUACCGAAAAGGGGACUACUGGGCGCACCGAACCACGGUCAAGGGUGGAGAGCUCAGGGCGCAUACCGCCAACCUUGCGAUACACGAACUGCUUCCACCCUUGGACGGGGUUCGAGUGCGGUGACCAAGAGAGUACUGGGUCGAACUCGGGGUGAUGAAUGUCCAUCGAGUAGGCAACCUCGUCUGCGUAGCGACCACAGACAGUUGCUCGAUCGUAGUCACUCGGGGCGGCGAGAUCUCCAACCUUACCGGACAUGCCCGCGUAUGCGAUGUGGUGGUCAACGACCAACACCCCAUCAUAUGCGAGGGCAUGUGCGGCAAGAUCAAAAAACUCGCCGACGGAGCGACCAUUCAUCAACACGAGACCGGCGCAUUUGACCAUUACGAUCACACUAGCAGGGGGUGCACCAGAAGCAAGAUACUGGAAUGCACCCAUACACGAGAAUUCUGCCGUGCAAAGGUCGUCAGGACGCUCCGCCGCAAUGAUCGAUUGCAAACGCGCCAACACGACUUGGUCGGGGUCAACGAAGGUCACGCGCGAGGGCCCAUAUACGAAUAGGCUGAGAAGAUGCCGCGAAGCACCGGAUCCCAGGACUACGAUAUGGGCGUCCCUUAGUUGCGUGACACGUGCGAGGACGCGUCGUGUCUGGCGAGAGUACAAGCGCUCACUGCGGAGGAGUUCUUGGCCCUCGGGGGAGGAAAGGAAAAUGUGGGCGCGAUCGCGUUGACGGUGGACCGCAUCCUUCGAGAGCAUGCCAGAGAGGGGUGGAAGGGAGUAGUCCACUCUGGCGAUGGGGAAGGAGGAUGCGGCCACAACGUCAACGUCGGUUCUAGCCGACAUUGCUCGUCGGGAGGGUAAGGACGCGAGGGUUGACGGGGGCCCUCUGCGCUCCACGCCGGGGGAGGACGAAAAAUCCUCCAACGGCGUGGCAACAUCAUCAUCAUCGCUGGACGACUCCCAGUCGGAGUCGUCCAGCCUAAGGCGUGGCACCUCAAGAGACAUCUAAAUUAUUCUACGCCGGUAUUCAGUACAAACGUGAGAUCGGA  >Ss-AA_clean.1_(paired)_contig_259  CCCCUGUCUAACAGUGCUGCCCUACACAAAAAUGAGUGUCUCCACUAACAUUCCAGCCCGUUAUUACUAUCUGGUCAUUUUAUCACAAGGUAAGAUCUCUGUCUUCCCCACGGAUGUUAGUAUCAUUGAAAAUCCUCUUUUGUCCACCUGCUUAAGUCUAUAAUAUGUGUCUUAAGAACAACUGUGAACAUCGAAAAGAAUUGGAUGGCUGCUAUUGUCUCUUUAUCCUCUUUUAAGGGUUGAAAGAGCUUUGCGAUAUCCCCAAUUAGCGCCCUGGUCUUUAAUAUGUGUCCAGAGAACAAACGUAAUUUUCGUCUAUCUUUAGUAGUUAUCCUCAUAUCGAGUUACAUCUAUUUACAGCCGAUGUGGCCUCAGAGUAGGGUUGGCCGCCCUAUGCGUCAAUAUCGUUGUGAGAUUUCCCCUGUUUACAGGCGGGGACCGUCAGAACGAGAUCUUGGCUCUCGUGCCACCAUAUUCUAUUGAUUCCAUCCACGUGAUUUGUAUCGUCACGCUCAGUGUUGCAGAUGUUGCGAUCUGCUUAUUAGGAACCCUUCGCACAGAGUUGCGAAGGUAGUUUACCGACAUUGCGGUCAUGAAUAUGUUUGUGGUUGCCCACAUUUAGGAGUUUAUCCACUUCUCGGUGUUUUAUAGUUUUCAAUCGUUUCAGAUCAAUGUCUAUCUUCCAGAAUAUGGCAGAUAGUGUUGCAUUUGGUGUUUGAUUUUGAUUGCCUCGUAGGAUUGGCCGUAGCUUCUACGGUGGUUGGAACUCACCUGUUCCGUUGCCCUCAUUCGUAUAAGGUUUGGGCCCACAAUUGACAAAGUUUUGUUGCCCCUAAAAAGGGUUUCUCUUUCCAGUUUUCUUCUGGUUAAGAUAUUUUAAGCUUGAUCAUAUUUUUACGGGGUUAUUUCGCCCUAGCGCUGCAACACGUCCAUAAGUAUUUUCGACUUUUGAAGUUUAUCCUCAUUACGGAGUUAGUAGUUUUCCGGCCGUUGCGGGCAAGCAUCUAAUCCUCCCACCCGUCGACUAAGUGGCUAGGCACAUCAGUGCCAAAGUCAAUUUGUACCACUUGUGAAAGGUUCCAGUUGUAGAAAUUCCCAUCGCCCUGAGGCAACUUGUGUCUACGCAAUUUGUCUUCUACGACAAAGAGAUCGUCUAAAGCUUGAUUCUUGAUGUGGGGUGCAAGAUGUUGAAAUUCCUUACGUUCGGGGAAUUUUCGCAAAUCUUGAUACCUCGCGCGUGCCUUGUUUAUAUCUAACCUCGUUUCGUACAUAAACAUCGCAUAGAGAGCUGGUGACCAAUCGCCUUCGAGCAUCAUCUUGUCAUAACCCCCAGGUAUGCCUUCUAUUAAACCAUUUGUUCUACACAAUCCCCUAUGAAAUGUCGGAUCCCAUUUGGAUGCCGUGUCCCAAGAAUUGCAUAAGAAUGGUGUUGAAAAGUCAAAAUUAGUAUCAUCAUAUCUAUCAGUUAAUCUGAUGUAUGUGCCAUUACACGUUCUACCAGUCCUACCCUUUCUCUGAACAGAAAUGUUCUUGGAAGAAGGUCGUUUGGUAAACUUGCCCUUAGAGUAUCCAAGGGAGUAGCCCAUAUCAAUGACCACACCUACAUGAGGUAUGGUUAUUCCAGCAUCGACUAUAGAGGUAGCAAAAUACCAAUCUGCUUCCAACACUGUGUUUGUGCCUCUAUAUAGUUUGCAUAUCCUUUUACCUUUAGCGAGUUGCUGAGCAUGCCUAACACAUGUCUCAACAUCAUUUACUGUGGGUAGAAUGACAAGAACCCUUUCAUUUGACUCGUGAUGAAGCAUCAAUUCAUCCCACGCGUCGGUUAGCUUCGGUACUCCCUUCCGAAUCUCAUCAUGCACAGUCCAUCUUGAAUUUCUCCCUUUUGACAGACGAACCUCAACAAACCUGUCAGCACCCGUGAAAGCAGGUGUUGCUGUUAUUGUCAAUGCUUGUCCUCUAUAACGGUCGAGUAACCAAAGUGUGUCCUCGUCCAUCUCAUGGAAUUCAUCAAGACAUAUUACCGUGCUUGGUGGCAAAUCUGCUAGGAUCCUCCUCAAGUAGCCAGCUGUUCCGAAAUUUAUCAUCCCUCUUGUCAUGUUGUCUUCACACCCUGCAUACAGGCGUGUUUGGGCGACAGGGUUGUUUUGGACCAAUAUAACUCUAGGGCAAGCCACAAUCACAGUUUCGAACUCUUUCUUGAGUGAAAGAAUAAAAUCUGUUGAUUUGCCUGCUCCUGGAGGACUGGUUAUCAGUGGUGUUGCAUUUCUUUCGAAAACAGAAAGCACUUGCAUGACUGCAUCACCAUGAUCUAAACCAGCCCAAUUGGUGUUCGUUCCAGGUUUAUUUGCUUCUCCAGUGCCUGGCACAGGGAGAAGAGAAGAGAUAUCACCCUCAAGAAAUAUUUGAUGUAUAUCCUGGGAGAGUUUGAUUAUAGAGUCAGCCCAAUCUUGCGCGCCAUCUAUCCCACCAUUGAAAUUGGCAAAUUCCAAUGGUGUUAUAGUGCACCAUAGCCUGUGUGCAAUAACCUUCAGUGUCAUGAACCUAUCUUUAGGCAUCAUUGCUGAGAUGACAGCUGAUGAGUCCCCAAACAUAGCAUAGUAAAGUGAAUUGAGUUUAGAAUAAACUCGUUCCGACAAUAUUUUUGCUGUGCCCAGGAAUCUAUACAGGGGGCCGACCACCGGUACCGUCAUUAAUGCCUGCUCCAGCCAGGCAACCAAAGUGUAAAUUCCCAUCAAAAGCGUUACUUUUACUCUAAGUUCAUUUGCCGUUUCCAGAGACGUGUCAUAGUAAUCACGUCUUUGCCAGAAACCUGGAAUGUCCAUGAAGAUUGAGAAAGGUGUUUUAGAUGCUAGCAUCUGUAACUUUUCAAAACUUUCAGGGCAGCCAUUUAGCAAGUACAAAUGAUGCUCAAGUGUGAAUUCUGUGUCAUGGUAGAGUUCACCAGGUCCUGUCAUCCAGAGCCCUGGGUCUUCAAUACCAAGUCCACCGACUUCGAGUAGUCCCGCGAAUUGACUUAAGAUACGGCCAUAGGUAUUAGCAGUUGAAUCGAAGGCCAAGAUGUGGGUGCGAGUUGUGUCCCACCAAUCUUCAAGUUUCUCAACGAAACCUGGUUCAUGGGUGGUGAUAAUCAAGUCCCGUCUUGUCUUAUUGUCUGGCAAGUACAUCAUCCUCAUCACGUCGGGGUACGACAUACGAGGAUGCUGUCUUAAGAAUUUCUUGCCCGAUCUCCGUUUCAAAAGCAGCUUUUGUAUCUCGUCAAGGUACACAUCCACUUUAGCAUGCACUUCCGGAUAGUGUGCACAAUUUAGUUGGAGUGCUGCGAACUUUUCAAAUCUAUACCGCAAGGUAUUCUUUUUCUUAUAAUCUGAAAAUUUUUGCAGCAGUCUUUCGAUAUCAUGGACGAUGGCCACUUUCGGUUGAGCACCAGUUAUAUCCUUAACCUCCUGUAGAUGUUUUUCAUCUAAAGAAAAUUUCUUCGAUAGGAAGGAUAAAUCUGCUAGGUUGUCGGAACAACCCUCAACACGAACUUGGACGCCCUUUGACAACCAGAAAUCUGAAACCAGUUUACCAGAGAAAACAUUUGGUGGUAGAUUAGUUGACCAAAAAUUGUCAUCUGAAAAAGAACUGAACUUUACCGUGUCGUAAAACUCAGCAUAUGGCCUUCCAGUUAUCUCCUUCCAGGCAUGCAACAUUAAAACUCUCAUGUACUGUGUGUUAGAAGGGGUCGUUGUUGCGUGACCAGUAGAGAGUCCCUGUUUCUUCAGGCGUGCUCUUCCAGUGUGUAUGUCAAUAAUCCAGGAUGCAAUAAGAUUGUCAUAAGUUGCAUCCACAGCAGUUUCAAUCUCGGCACGUUGGGGAUGGUUCUUGUAUCCCUUCUUGUUCAGUUUUUUAAUAACUCCGACAGCGUCUAUGGCAGCUGUUGAGUCCAUUGCAGUUACAUCCAUUGCUGUGUGAUGCUCAUAUGCCAAAUGUUUGGCAGCCAGAGCUGACAUUUGACCAUGUGAGGGGGAUAUACCUACUGCUGAAAAGGAGGAAGGAUCCAAUCUCUUGGCUCCAUCACCUUGCACCGCCAUUGCCAGGAAAUAAUUCAACGGAUCCUGUGCAAUGAUUGUUCUGAUCUUUUCCCUUUCAAUGUAGCUUGCAGGGAGAACUUCAUCCUUUAUAAACGCGUGACUAACAGUGGGAAAUGCUUCAGGUGAUUCGAUAUAAUCUCUAACACACUGAAGGAAUUUUGCCUUCCCACCAACAGCGUCAACAAGUGCUUGUCUCUGUGCUCGCCCAUUUUCAUUAAACCGGAAAGGGAAACCGGCGGAAUAGUUGCGAUGCCAUUUUUUCCAAACUUCUUCGGGACUUAUCAAUUGCAUGUCCGCGAAAAGGUGUUCUUCAUUUUGGAAAAUGGCCUCAGCUAUUGCCUCUUGGUCUUCUUCUGAGAUGAGCUGACCAGCCUCAAAGGAACCCUCAAAGUAUCUAUCGGUAACUUUUUGGGCCAUUUCUACCGUGCCAAAGCGUAGAGAGAACACCCCCAGUUCAAACGGGUGCAUCUCUUGAAGUUCUUGGCACAAAGAUUGGGUUAAACUAUCCAUUUUUGGUAGGUCCUUACGGAUGUUAACCUUUACUCCAAUCUCAUAUUCACCAGGCUUGAUCCACUUGAAACCAAUCUUAGGAAACUCCACUCCAGGGAAGUAAUGUUCAUACUUCUUAGCUAUUUGUGGGAGGUCAAGAUUGACUUUAUACUUUUUCUGCAUUGUCAAGUUUUCAGGCAUGUGAUUUGCUUCAACCAACCGAUUACCAGAUCGAAACCAGUGCCUAGGCACUGAUAUAUUUACCAGCGGCGUCCAACAUACUUUCUUUCCAUCUCUCAGUGCUCCACGCAAGUUCACUGUUGAAGUGGCGACCCCAAUUAAAUCUUGGCCUAUCCUUGUUCUCCCAGCGGAACGCAUCAAAGACAGCAUGUGAUGUAAAAACUUCCAUUUUCUUUGCGAAGUUUUGAUCUUCCUGUUCCUACGCACAAGUAUGGAUCUUGCCACGAUUGAGCCAUUUAUCCCGAAACGGAUGACCGCAAACCUGAUAAAAUCCAUCAUGUGCUCUUUCUCUUGAGGAAGAUGUACAUCUGGGUCGUCACUAUAAGCCUCUAGAGCAGCUUCAAGCCUUUGCACAGUUGACAAGCCGUAAUCUUUAAUGACGGUUUUGGGACCUGUCCAACUCUUGAUAGAGUGGAUUAUAUUGCCAUCGUAGGUCAAACCAGCAUCCUUGGUAUCUUUCACCUGGUAACGUUCUUCAUCCUCAGAUAAGUAUAUCCAAGAUGAUGCCCAUUGACCAAAUGCACUAGUUAUACUCUUUGGUACAUCAGCACCAAAAGCUCCAAAAUGCCUCCAAGGCACAACAGUGCCUGUCAGCAUGUAAACAAGUAGAUUAACAAAUACCAAGCAAUUAUCCGCCAAUACCUUGUAACCGAGACCAUCAAAAUCUCUAGAUAAAGCUCGUUUAUCCAGCUGCCCUAUUAUAGGCGAAUUCACGAUGAUAGUCUUGACCAAAGGUCUCUGUUUUGUGCAUUCUGGGGGAUAUAGAGUCCUGUUAAUAAAACAUUUCUCACCUUUUAUCUGUUGUAAUUCCCAGAACCAGCCAUCAUACUCCAUAACAGCGUGAAACAAACCGAAAGCAGAGCCUAAUAUAGGUAGUUUCAAAGAGUACAAGUGAAUGCGUAACCCACUUGAUCCCUUUGGCAAAUUGUUUAACCACAUAUAUCCAGCAACCUUUGUCUUUAUGGGUUUUGCUAAAGCGAACGAAUACGGUAACAACACUUUGAUAUCAGCAUUCUCUAUCGCAGGAAAGGUAUUUAGAGCAAAGUCGGGUAUAGCGCUCAUAUCUAACCUACGUAAGAAAUCCUCAUAGUGUCUCACGGCGUCUAUUAGGACGUGCCAUUUCUUCCAUGUCAAAGACCAUCCCGCCGGUGUCAUUAAAUUGAAAUUUAACACCCGCGUGGCCUUUGCCCACAAAGUAUUUAUUGACAACACGUCUCCUAAAGCUUUAACCACAGAAUUGCCGAUCUCCUUAACCCCAACGUCUAAGUAUCUUGCUUCUUCUCUACGAAUACGCAUAUCAAGAUGUAUUUCGAGAGUUUUAUACUUGUAAUGCGGAGGUUUGGAAUUGGUGUAUUUCCAGAAAGCUUCACAAGCGGAAACAUCGUCUAAUGAGGUGAUGCGGUCCAAAAGACCACGUCCCACUUGCUGAUGUGCUCCAUUCUUGAUGAAUCUAGAAUCUAUACAGUAGUACCGUUUCCGUGAUUGAACCCAAAAGGCUGAAGGCUCCUUUUGGAAAGUUUUCAUCUUAGAGACAGGUUUGAGCCUCGGUUUGAAACCCUUGUAAACGGUGUUCACAAGCGUCUCAACAUCCUCGACUUUCAACCUGCCAUUAUCCAGUUGUGAUAGCAAACAGCGUUCACACCUACCACUCAUAUUGUACCCCUUUACACCACACCUACAUGUGAUGUUUCGCGGUUUCAUAUAAAUGUAGGGAUUGAACUUUGACCUGGAUGUCAAAGCUUUGUUGCUGUAAGCUUUUAAGGUUUCUUCACUAUUAUUGUCAAGAUAGAGCGCAUUGAAUGACUUCAUAUGUGCCCCGUCAUUGUGUCUCCCCUUUGAAUAUGCCCAGCCAUGGGCAUGUUCAGGGUAAGUUUGAGUAAGUGAAAAACCAGACCAACCAUACACCGGGUCUCCCCGGGCCGAGCAGAUUUUCCGUAUGAAUUUCAGUUCAUGUUUAUUGGGCUGUAACUUGCUUUCCCAACCACUAUUGAAGAUACCAAACCAGUAGUCCUUAUUUUGACCGACUAUGUAGUUUGGUGGUGGACCCCACCCAUAACUGUUCCAGUCAAUGCAGCAAUGCAAAGUCAAAUGAAAUGGUUCUAUGGGUGUUGCGUUAUUGUCUAUCAAGGCUUCUAACCAAUCGUCGGUGCUAGUAAGUAUUUCUUCGCACCAAGUGGUUGCCUCUUUCGCAGGCAGACGAGUCUUAAAAAGUCUUGAACGCAAGAUGUCUUCCGUCACGAGUGGUUUUAUUGUGAACUGCUCCAGGGCAUCUCUCCAGACGAACUGAUCACCAACUUGGGGGAGUAUCGUUUGCGGAACACCAAUCGCCAAACACGUGUUGGUUAUCCCAGAACCGCCAUGAUGCACGACCCAAUCAAAAUGAUGGAGGUAUGUUGUGUGUGCAAUAUACGGUCGUGUGGAAAACGUUGUACCCUCAAAUAAGUAAGUCCAUCUUUCAUCAACUUCCCAGAUGACGUUUAACCCUUUUAACCAAUCUAUCAGCUCCUGGGUCUCUCGCGUGAUAGAUUCGCAUGAACCUAGAGAAAAAUAAGCAUGUAUACUCUCAUUCUCUAUGAAAUCCUCUUUCCUAACGAAAUCACUAUACGGCUCAAAACCAACGUUUGGCGAAGAAAUUUGCCAUUUGUCAGCAGCCCAUGGUGAUAUUGAAUGAAAUUUGGGUGGUUCCGUAUAUUUGUACGUCCCCUCCCAUUCUAUCCCUUGUAAGAACUUAGGAGCUGUCAGAUACUGUGCAUAUAUCCUUGAUAAAGAAAAACCAAUGCUUGGUGUGGCAAAUGGGUUAUCAAAGUAGUGAUGCAAGUAGAAUUCUGGGCAGACUCCAUUAUCCUCUCGCGGAACAGGGCAAAUUUCAUAAACUUGCUUUUCAGGAAAAAUGGAUGCCAUAAAGUAAUAAAAACAAGUACCAGAAACAAGGUAUAUGAGGUCUGACUGUUGACAGACAUCCAUACUUGCUUUGACAUUCUCGUACAAUGCUUCAUGCAUAGCUUUCAGAUCUUUGCCACCAAAAAUACCCUUCUCAAGAAUUGAUUGCCCUAAAGCAAUGAACUCAUGUGAAGUCUUAGCUAGUGGUCUCAUAUCCCAACCUUUAUCGGCAAAUCUUGCCAUGUGGUCUGGAUGAGUUAUGAGAACAAAAUCAUCUGGCAUAACACGCUUAUGUUCUCCAAUCAUUCUGUUCGGAACAUUUAGAUACUCUGAUUCGUCGCAAUGUUCCCUAACGAACGAACCAGGCAUGUGCAACGGCACAGGUAGUAACUCUCCACCCCAUUCACUACAUAUGCUAUUCCACAAGACAGGGGAAACUUUCAACUCUCCUUUAGCUGAACCGUACUGUUCAGAGAAACGUUUUGCAAAAUUGUCAUUGACCUUGAAGAAGUCAGUUGCCGUCCACCCGGCUCCUGUCUCUAAAAAAGAGAAUUCACCGUCGAAGUCUUCGUCAACAGGUUCAAUCUUAUCCAUAGAUGCGGCAAAAUAAUUUGUUGCCACCACAUCCAAAGAUGGCUUAACGUCUCCAAUAGUACCAGCUACAACCAGUGUAGUUAGUUCCACAAACUUGCAAAGUUGGCUAGGAUCUUUAAAACAAAUGUCAAAUAUGUGCUCGUUCAGAGUCUUCAUAUCCCAUUGUGCUUCAUUGACCCUACCCAUUGUGGCAUUAGCUACAUGGUACUUGUCACCAUGGCGAGAAAAUUCAAUAUGAUCCCAAUCAUCAUGUGGAAUAAAGGCACGAGCCCCUAUCAAACUUGACAGAUUGUAAAGCAUCAUAUAUCCUAGAUCGUCCCAGGCAGGAAACAGUCCCUUCCAGCAAAGCGGGCCACUCAAAGUGGCAUCAUAUUCUUUCUGAAAGUCUCUAACUGUUUCAAAAGCUGUCAUUCUCAAGAUUGGCUCUCCCACAGGAAAAUCAACUUCGUCUCUAAUGAGGGAAUCACGCAGCGCGUCCACUAAAUUGCCAACUAAGCUGUCUCUUUCAACAGGAGGGCCGGAGAAAAAUUCUUCAUCAUCACUAUCAUUAGCCAGGUACGUAAACUGAGUGUGAGACAAAGAUGCAAAACUAGUUCCACCUUUAUUAUCAAAUACCUCAUAAGUUUCUUCUUCUAACUGCUGAGUCGUGACAUCCAUCCUUUUAAGGUCAGGGGGUGCUUCACCUUCGCCUAAAUGGCAAAUGGCGAUAUUAUUAUAUAACACCUCAAUCCCUUGUUGUGCUUUGUCUGGUAUGAGGGAGGAGUAACCCUCAAACCAGUGUAAGAUGUAACCCUUCUUCGGACAGGCCCAGAACAUCUUUAACUGGUGAACCCAGUAUAAGAAGCGGUCCGCUGAAACUACACCCAACAAUCCUUGAACAUGUAGCACUCGCUCAAGUGCUAAGAAGUUAUCAGGCAGCCAUUUCCUUCCGAAAUGUUCUGCCACAUGCUGCACUGGAGUUCCCAGUGCACGAGCAAAUUUGCAUUUCAGCUGCCAAAGUUUCUUCUCAAGACAUUUCUGCUGCCAUCGCAAGGCAGAAAAGUUCUUUUUACAGUUGUAAGCUUUCGUUCUAACGCGGGUCUGAACACCAACCCACGCCGGGCCAUCAUACACUUUCUCGUGCACAUCAGAUAAUGGAGCAUAAAAGCCACCAUCCAUACUGACAUGGUAUUCCUCGAGAACGUGAAUCCGUUGAGAUUUGACAGGUUGCUUGACAAUAGGUUCAGGUUUAGGGGCCGGAAUGCCCCAGAACUUUUCACCUGUGCAGAAGUCAACACAUUCGACGAGUAAGCUGUUAGACAAUCUAACUUUCUUUUCAUACUCGGAUGGUUCCAUGACUUCAGCCUUCACCUGUGCCUUUUCCGGUUGACAGAAUUUAAGAUGCUGAUCUGGGUAGUAUUCACCUGUGAUCCAAUCUCUGCCUGUGCCAUUAUAGUCCACUCCUCUGGAAACAGCGACCCGAGUGGGUGGAGUUGCUGCCUUAUUUUUGGAAUAUUUCCGAUCUUUCAAAGGUCCAGAUCGAUUCCGCUUGACAAAAAGGUUUUGUUUUAAAGAAUUCAUGAUUGUUUCGUCCCGUAUUCGGAGGGAAGCCCUACUCAAUCAAGAGAUGUGCCGGGCAUCACGGUUGCCAGUGUGGGAGCACGGUCCGACACUCUUCCACUAAACCAGUAGGCGGUUUCACAUUUGCCGUAAGUGAGACGUAAGAUAUAUGUUUUAUAUAUUUUGCGUACAGCCGCCGCCAUUUCGCUCUAAGCGUGUUUCGCAGUAAGAUGCAAGCUAAAUCUAAAUUUCAUUUUUAGUUUAAGGUUUUAGAUAAUUUUAUGUUAGUUUGAAUUAAAGUUUUAGUCUGAAUAUAAAUUUAAAUUUAAGUUUAAGUUAGAAUUUAAGUUUGACAGUGACUUGUCAGGCUUGAAACAACACGAAAGAAAG  >Ss-AA_clean.1_(paired)_contig_1288  UCGACUUUACGGUCUUUUCUUUUCAUACCAGUUAUCCUCUUGGGAGCCUCACCACCUUUUGAGAGUUUAUCCUCUUCUAGAGUUAUAUUGGCUUUCCAGCCCAAAACCAUUCCAGUAGCCAAACCAAAAGUGUGCAAGAUGAAUUUUGUUAUAAUAUUAAGAAACGUGGUAACACGUCACUUGCAAUCCUGGUUCUUGUAAGCCGAAACUUACAGAACAGAAGCACCCUCGCCACAUGCGUGGUGUCGGGUACGACAUGUCCCAAUGUUUCACAAGACCAUACCGUUUGACCCAAGAUGGGAAAACAGCGGUCUGUAAAACGAACCAGAAGUAGCUUGCCAUAACAUUUUCUUCAGAAACGAAGAUAAAAACACCAUCCCUGCCAGCUUGAAUGACACAAGUGUAGGUCGGUUCCAUAAGGAACCUGCAGCUUGUGUCAUUGUAUGGUACACGUAUAACGACCUUCUUGCCAUAACUUCGUUGUUUAGGUGACGGGCUAACCCGUAUAACCUUUCCCUCGAAGUCUUUUGCGAGCUCAGAUUUACCUGGGGUCCAAUAAUGAAAGUUAGUUAUAACAAUCACAAAUUCCCCUUUCAAGGUUUCUGAAGCAUCCAAGAAUGUUUUUACCUCACCAAUGUUUAUGCGACCGGGUACAAUAUGCAAAGAGUUUUCUACCUUGUGCCAGCCAGGGUGUAUCCGCAUGGAAGCAUCAAAACCAUCCGCUGCAACAACGAUAGUUUUGAAAACCUCAGUCAAACCAUUAACAAAAGCGACGAAACCAGUAGAGCUUUGACUAGCUAUAACCGUUGGGUGAUAACCAGUAGCAAGGGCAGUGUAACAAUCGGCGACACGUCGUGGGACCAGAGCAGAAGAAUCACAACUGUGGUUAGGUUGCCUUGAAGUCACCUCAGCGAUAGCAGUGAACACAGCGUCAGCUGAUUCGACCACAUCGUCAACCACUUCAUUCACAACCUGGUUAAUCUGAGCUUCAAGUGGGGACUCUUCCUUGGUUGAUCCAACGCCAACAUCCAUAGCAGCUCCCAUGGAGCAUGCCACUCUGUUGUCCGUCGGUAACCCCCAGGUGGUUGUUGAGUUGGGUUCAAACCAAUCUUCCUCCUCAACAUAAUCAUCAGGUAGCUCGUCAUAAAAACCUUCAUUAUAAGCGUCCAAUGCGUCAGCAGCCGCAGCACCAAAAUCUUGUAUUCUUUGUUCGAUCAUCUCACUAGGAAAAUGCAUUGUAUUAGGAACAAAGAAAGGAUCUUGAGCCAUUGCUUGCCACGUAUUUCGAAACGCUGGAGACCACAUAGAUUCGGCAGAAGAACCAGAAGCCGGGCUUGGUGACGGUGUUUCAGUUGGUGGGAUAACACGUAAGCCAGGGUAAUUUCUUUGCAGAUCACGGUAGUGAUCCCAAUCGCCUGCAUGUGUAACAUCUUCACCUUCAUCCUCGUCUUCCCACCCUUCGUCAUCAUCUUCUUCGUAUCCAGAAGAGCCAACUCGAACAGUAGCAUAAGCCUCAUUGUCUAUCAGGGUUCUGGGAGUUAUACCCGUAUGGUUGUGAACCCUCUCAACAUGUAAGCUGACAGGCUUAAGUGGGCCCAUCUUGCUUAGUCCGAUAUUGGCUGUAAUCUUGAUCUGGAUAUUCUUGAGAGAAUACUUAGGUCUUGGACCGCCAGCAACAGCACGCAACUCUGCUAGCGUAGGAUCAGCUUUCAAUUCCUCCAUGACAUUGUGCCUAUGCUCACGUUCAAACAUCACGGCAUAACAGUAACCAGGUCUGGGAGCCUUCUGGUGACUUUCUGUGCUCGAGCCGCGAUGCCUGCUGCUCUCUCGUGAUCUUUCAAUCGGCCGAGAAGGCAAAUUUAAAGGAGCAGGCCUAUGUGCAAUGGUUAUUUCCUCACCUCUAGAGGAUGAAGGCCGUGAGGGUGGCUUUUCAUCCCUACCAGAACGUCUGGCGUGUUUAUUAUCAUGUUCUCCACCGCUGGAUAUAAGAGAUCGUGGAGAUGAUUCUCUUACCUUGUGUUCGACAUUGUGAACCCUUGCACCACCCCCGGUGGGUGCAACAGGUUUAUGUCCCUUGUCAGAAGAAGAAGGAGGAUUGUUCAUAUUUUGGUUCGUCCCGUUUUCGGAGGGAAGCCCUAUUCCAAAAGUGGAAAACCGGGCAUCACGGUUGCCAAUGCGGGCACACGGGCCGGCAUUCUUCUACUAAAUCAGUAGGCGAUUUCACAUUUGCCGUAAGUGAGACGUAAUCCUUGUUGGGCCAAGAUAACCUGCAUGAGCAUAUUUCGCAGUAAGAUGCUAGCUAAAUAUGAAUAUAAAUUUAAUGUUUUAUUUUAAGUUUUAAAAGUUUAUGUAAAUAUAAUUUAAAGUUUUAAUUAAAGUGUUAAUAGUUUUAAUGACAGUUUGAAUUUAAAUUUUAAGAGGCAGCAAUUUGCCAGAUUUGCAACAAUAUGAAACAACAAAAG  >FirsU_Contig182  AGAAGGACCAUUGAUAAUCUGACCACACCAUCCCUCAACGGGUUCCGCAGGGGACCUCUGNAAGGAAAAGGAGUGCACGAAGGACAGAAGUAUCAAUGUGUAGAGGAACCAUGUGAUUAACNCAGUUCGCGGCGCCAUAUAAAUUGAUCACCUAUCUGUGGCAAUAUUGUCUGAGGUACUCCNAACAGCAAGGCAGGUGUUAGUCACACCUGACCCACCAUGAUGUACGACCCAGUCGAACUUNGCACAGGUAAGUGGAAUGAUUGGUGAAAGGAGAUAUGACAUAUUCAGAACCUUCAAAUAANAUGAGUCCAACGUUUAUCAACCUCCCACAAAACGGGUAAGCUUUUCAACCAAUUUAUAGCNCGUUUUAGUUUCGGGCGUUAUACUUUCACAUGACCCAAN  >Ss-AA_clean.1_(paired)_contig_5231  CUACGGACAUCAUAAACCCCACCUUCCAGAGUAGCUAACACACAGCGUUCACAUUUGCCUCCCAUAUCAUAACCAUAGCCUCCACAGGCGCAUGGUGCGUUAUUGGGUUUGACGAACAUGUAUUUAUUGAACUUUGACCUGGAUGUCAAAGCUUGCUCACUGUAUGCCUUUAAGGUUUCCUCACUGUUUGCACUUAUGUAUUUAGCAUUCAGGGCCUUAACAUGCCUUGAAUCAUUUAUAAGUUCUGGAGACGUUAUCCAACCAUGUGCAUGGUCAGGGUAAGUCUGAGUGAGGGUAAAACCAGUCCAACCCACCACAGUGCUUUUACACUGGGUGGUACAGAUUUUCCUAACGAAUUUUAACUCUAUGUUGACCUCAUUGGUUUCCCAUGUGCUAUUGUACAAGCCAAACCACCAUUCCUCAUUCUCUCCAAUAAUAUGGACUGGAGGGGCCCCCCACCCGUACGCACUCCAAUCGUGACAACAAUUGAAAGACAGGUGGAAGGGUUUUACAGGUGUGGCGUUGUUGGCUUGUAAUGCAGGCAACCAAAAAGAAGGACCAUUGAUAAUCUGA  >FirsU_Contig146  NGGGUUUUAUGCUCCCCUUUCAUUCGUCAAAGANGGAUGAAUAUGCUGGUCCUGGAUGGGAUGGGUUUGCAGCCCCAUCCAGGAAAAGAGGUUUNUAAUUGCCAAAAGAACUUUUCUGCCGUGCGUUGGCAACAGAAAUGUCUAGAGAAGAAAAUNGUGGCAGUUGAAAUGCAAGGUUGCCCGUGCAAUAGGGCAACCUAUUCAACGCGUGGCAGANGUACUUCGGAAGGAAGUCUCUGCCAAACAAUCAAGUAGNCACUUGAAAGAGUGCUAUCUGCNUCAAGGACUGUUAGGUAUGGUUUCUGGUGAACGAUUCCNUCUAUUGGAUCCACCAGUUGAANACGCUUGUGGGCUUGUCCCAAAAAAGGCUAUAUCUUGCNACUGGUUUGAGGGGUUUUCCUCNCCUCACACCAGAUAAGAAACAAAGCGGAAUUGAAGUGCNUUCACAACAAUAUCACCAUCUGNCCAUCUUGGCCUUGGUGAUACACUUCGAGAGAUGCUUANGCGAAGGAACAUCAACAGCUGUCUGGGCCAGAAUUUGAUGAUGAAUACGAGAUCUUUGAUANACCACGGUGGCACGCAAUUUGCNACCACUACCCCCUCCAGGUGGUUUCUAUGCAGUAUACANACGACAGCGAUGAUGAAGAUAANUUUCCUUUCUGGACCACCCAUAGAGGGAGGUAGCCUCGNUCGGCUCUCUCGUUGACUCUCUNCAAGAGUUCAAUGAUUAGGGACGAAGUUGACUUUCCGGNUGGGUGAGCCUAUCUUGAGGAUNGACAGCGUUUGACACGGUGAAGGAUUUCCAAAAAGCGUNAUGAUGCCACACUAAGUGGCCCNACUCUGCUGGAAAGGCUUGUUUCCUGUCUGGGAUGAUCNUAGGAUAUAUGAUGUUAUGCAANUCUAUCAAGUUUGAUCGGUGCUCGUACAUUCAUACCGCNAUGAUGACUGGGAUCACAUAGANGUUUUCUAGACACGGAGACAAGUAUCACGUAGCAAAUGNCUACAAUGGGUAGGACCACCGANAGAACAAUGGGAUGUUGCUGCACUAAAUGAGCACAUCCNUCGACAUAUGUUUUAAGGAUCCNAACCCAACUAUGCAAAUUUGUGGAGUUAACAACAGCUGNUCGUCAUGGGGACAAUAGGUGAUCCUUGAACCCACCCUUGACACAGUUGCGACAAACUACUNUUGCUUCACAAAUGGACCAAAUNUGAGGUUGUUGCCCCAACCGAAACUCAGUACACCUUCCNAGGAGACUGGAGCUGGCUGGACCUGCCACAGACUUCUUCAAAAAACAAUGUGAUGGUUUUGNCCAAAGAUUUUUCUGACAAACANUGGCUCAGCCAAAGGUACACUAUCUGUAUCACCUGAGCUUUGGAACCUCAUAUGUCACGUNAUGGGGUGGCCAGAAAGUGUAUUUUGAAGGAGGGACAAUGCCCGGUUCGUUUGUUGUGGANACUUACAGACGACACUGAAUAUCUAAAAGUUCCUGAUAGAGAGGCUGGACCCCAUGCCCGNGGUUAUGCCUUCAGAUUUUGUGUUGGUUACACAUCCAGAUCAUAUUGAAAAGAUGGCACCNCGAGAUAAAAGCAGGCAACUGGGAAACAGUUGCGUUGCCUAUGGAUAGUAAGAAAUUCAUNAGCACUUGGACAAUCCAUCUUGGAGAAAGGAAUCUGGGCAGCACAAGAUAUGAAAGCCAUNGCACAACACGUUGUACGAACACAUUAAGGUUGCUAUGCCUAAAUGUGAGAUGAGUGAUCUNUAUAUACCUCGUCUCUGGAACAACGUUUUACUAUUUCAUGGCAUCUGUUUUCCCUGAUAANACAGGUUUUUGAAGUGUGUCCGGUGCCAAGAGAGGACAACGGCGUUUGCCCUGAGUUCUANCUUGGCACACUACUUCGAAGACCUGUCUCUCGAUCCCCAUGUUGGUUUCGCUGUUGGGAGNGAUGUAUUCUAAAUGGCUGACUGCCCCAAAAUAUCUGAAUAGGCUUGAGUGGGAGGGUACNUUACCAGUACCGUGAACCCCCAAAGUACCAUUCCAUUUCACCUUGGGCAGCAGACAGAUGNGAAGAUAUCUUCUCCAAAUAUUGGCUUUAAACCAUAUGAUGAUUUUGUAAGGAAAACAGANCUUUAUUGAAAAACCUAUCAUCCUUGGUUACUUCUCUCUUGGGUCAUGUGAAAGUAUAAC  >Ss-AA_clean.1_(paired)_contig_1373  CAGCCCGUUAUUACUAUCUGGUUAUUUUGUACAAGGUAGGAUCUAGGUCCUCCCCACGGAUGUUAGCUUCAUUGACAGCUUUUCUCGAUACGCAAGCCCAGUUCUAAUGUUACUGGGAACAAGUGUCGUAUAGCGAAAGAAAAUGCUUUUACGUUGACUCUUUAUCCUCUUCUAAGGGUGAAAGAGCUGUGUUGCUUCAUAUGUCACGCCUGGUUCUCAAGCUACCAAGAUCAAAUGGACAUAUUUCUGCUUUGAUAGUUUAUCCACAUAUUGGUGUUACACCCAUUUACAGCCGGUGUGGCCUCAGAGACAGGAUGGCCGUCCUGUUAAAGGUUCAUUUCGGUGUGAGUUUCCCCUGUUUACAGGCGGGGACCAUCAGAAUGGGAUCUUGACUCCCAAGCCGUCUAACUGGUGUUUCCAACCAGGUGUUGUGUAUCAACACCCUCAAGUGUUGUGGAUGUUGCGAUCCACUUAAAAGGUUAGUGCCCUAGCUCCUAGUAGCGUUGGGCUGAUUUAUCGUCUCACGACCGGAUUGUCUAUCGUCAGCAGUGUGGCAGAUAGUCUUAACAUUUGGUGUUUGAUUUUGAUUGCCUCGCAGGAUUGCCCGUAGGUUCUGCGGUGGUUGAGUCUCACCUGACUCGUUGCCCUCAUUCGUAUAAGGUUUAGGCCCACAAUUGACAAAGUUUAGUUGCUCCUAAAAAGGAUUUCUCUAUCUAGUUUUCUCUAGCUUAGAUCAUUUAAGCUUGACAUAGUUUUACGGGGUGUUUCGCCCUGGCUCUGUUAAGACGUCCACGCUUAUUUUGGACUUUAGGAGUUUAUCGACUUCUCGGUCUUAUUGUAGUUUACGGUCCUUGCAGACACUGGCUUACUCCGCAUCCAAGAGAUGUGAAGGUAUUGGUGUGUCGAAAUCAAUAAAUUUGGUUUCAGACAAAUUCCACGUCCAGAAAUUUGCCAAACCAUCACCUAAUUUAUGGCGACGUAGUUUGUCUUCGACAACAAACAAAUCGUCAAAUGCAUAGCCUUCACGUUGAGCAGUUAAGUGGCGGAAUUCCUUGCGCUCAGGGAAUUUCCGUAGAGCUUGGUAAGAAGCUCUAGCUUUAUUAACGUCGAGUCGAUUCUCAUACAUGAAUAUUGCAUAAAUUAGCGGGGACCAAUCAGCGUUGGCAAGAAAAUCUUCGUAGCCUCCAGGUAGCCCGUCAAGGCACCCAUCUUUUCUGCAGAGUCCACGGGUGAACGUUGGAUCCCAUUUUCUUGCCGUAUACCAAGAAUUACAUAAGAAAGGAGUUGAGAAAUCCCAGUUUGUGUCAUCGUACUUCUCACUAAGACGAAUGUAUGUGCCAUUGCAAGUUCUACCAGUUCUUCCUCUUCUCUGCAAAGAGAUAUUCCGAGAGGAAGGCCUAGGUUUAAACUUGCCUUUGUGGUACCCCAUGGAUACCCCAAGAUCUAUAACCACAUUAACGUUCGGUAUGGUUAUUCCAGCAUCUACAAUCGCAGUGGCAAAGUACCAGUCAGCCUCAACAACAGUUGAGUGUCCUCUAUAAAUUUUUGCACACCUCUUGUUUGUUACUAAUUGUUCAGCAUGCCGCAUGCAUGUCUCAACAUUAGCUACAGUCGGCAUGAUGAAAAGGACACGGUCAUUCGUUUUUGAGAAUGUCAUGAGAGUCUCCCAACCUACAUCAAGCGUGUUUGAGCCUUUACGUAUAUCAUCUGUUAUCGUCCAUCGAGAGUUCCUUCCCUUGCUAAGCCUUACUUCGGUGAACUUGUUAGCACCGUAAAAGUCAGGUGUUGCAGUUAUAGGAAAUGACUGUUCAUGAUAACGGUCCAAGAGCCAUAAAGCAUCUUCGUCCAUUUCGUGAAACUCAUCGAGGCAGAUGAUAGUGUUUUGAGGCAAAUCGGCUAAAACACGCCUGAGAUAUCCAGCUGUUCCAAAAUUGAUUAAUCCUUGUGUUAAAGUGUCUUCACAACCAGCAUACAGUCUGGUUUGCGCCACAGGAUUAUUUUGCACUAAGAUUUGUCUAGGACAUGCUACUAUCACAGUGUCAUAGACUUGCUUUAGCGAAAGGAUGAAAUCAGUGGAUUUCCCUGCGCCAGGUGGACUGGUGAUCAUGGGUAUUUUACCUUCAGAGAGUAAGGUCUGAACCUUAUCUACUGAAUCUGCAUGAUCGAGGCCGACCCAAUUGGUGUUAACACCAGAUUGAGCCCUUUCACCAGUCCCCGGGGCGGGGAGAAGAGCGGACAAAUCCCCAUCAAGAACUAUUUGGUGUAUGUCUUGUGUGAAUUUUAUAAGAGCAUCGGCCCAUGCUUGAGCACCGUGGAUACCACCCUUUAGACCACCAAAGUCUAAAGGGGUCAUCGUACACCACAGUCUAUGUGCUACCACUUUCAAGCUGUAAUAUCUGUCCUUAGGCAUUAAGGAAGAUAUGACGGCAGAGGAGUCUCCAAAGACAGCAUAAUACAAGGAGUUUAGUCUAGAAUAGACCUUCUCAGACAUGUACUUUGCUGUUCCAAAGAGACGGUAGAGUGGACCUACCACAGGGACGGACAUUAAGGCUUGUUCAAGCCAGGCUACAAGGGUGUAAACAGCUAAAAGGAGAGUUACUUUUACUCGUAAACUAUUUGCCAUUUCCUCGGAGAGGUCAUACACUUCACGUCUUGCCCAAAAACCGGCUAUAUCCAUGAACCCUGAGAAAGGCGUCUUAGACGCUAAGAUUUGCAUUUUCUCAUACGAUUCAGGACAGUUGUUUAAUAGGUAAAGGUGAUGUUCAAGUGUAUACUCCGGAUCAUGGUAAAGUUCUCCAGGUCCUGUGAGGAAUAAACCUGGAUCCUCUAUGUUUAGGCCACCAAUUUCCAACAGACCAGCGACCUGACUAAGUAUUCGACCAUAUGUGUUCGCAGUUGAGUCGAAUGUCAUGAUAUGACUUCUGGUUGUGUCCCACCAAUCUUCAAUUUUUUCAAAGAGACCUGGUUCGUGAGUGCUCACUAUCAAGUCUCUCCUUGUCUUGUCAGUUGGUAGAUACAUUAACCUCAUCACAUCAUGGUAUGAUGUCCGGGGGUGCUGUUGCAGGAAACGUUUGCCUGACUUCCUUUUCAAUAACAUUUUCUCCAUCGUGUCCAAGUAUUUGUCAACUGUUGCGUGUAUUUCAGGGUAAUGGGCACAGUUUAGUUGGAGUGCAGCAUACUUUUCCCAUCUAUAUCUUAAUGUGUUCUUCUUCUUAAAGUCAGAGAACUUUUGCAAUAGACGGGACAUAUCAUGCACAAUAGCAACUUUAGCGUGCGCGCCAGUUAUCGUUCUAACCUCUUCUAAGUGUUUCUCAUCUAAUGAGAAUUUUUUAGAGAGGAAAGAGAUAUCUGCGAGGUUGUCAGAACAACCCUCGACACGCACCUGCACCUUAUUCUUUAGCCAGAAUUCAGAUACUUUUUCGCCAGAGAAAACGGAAGGGGGAAGAUUAGUAGACCAGAAAUUGUCAUCUGAAAAAGAACUAAAUUUUACACAAUCGUAAAAUUCAGAGUACGGCCGGUCUGUUACUUUCUUCCAUGCAUACAACAUCAAAACACGCAUAUACUCUGUGUUUGAUGGUGUUGUUGUCGCAUGGCCAGUAGAAAGACCUUGUCUUUUUAAUCGUGCUCGACCAGUGUGUAUAUCAAUAAUCCAGGAUGCAACUAGAUUGUCAUAGGUUGCAUCAACAGCAGUUUCAAUGGCUUCCCUUUGGGGAUGGUCUUUGAAACCUUUUUUCCUGAGUUUUUUAAUCACACCUAUGGCGUCUACAGCAGCGGUCGAAUCCAUUGCUGUAACGUCCAUAGCGGUGUGAUGUUCAUAUGCCAAAUGUUUGCUCGCCAGAGCAGCCAUUUGACCAUGAGAGGGAGAUAUACCAACAGCUGAGAACGAUGAGGGGUCCAACCUUUUUGCACUAUCACCUUGAAUUGCCAUAGAAAGGUAGUAGUUCAAAGGGUCUUGGGCUAUAAUCGUUCGUAUUUUUUCCCGUUCAAUGUAAGAUUUGGGAAGAACUUCAUCUUUGAUAAACGCAUGACUGACGGUUGGGAAUGCUUCAGGUGACUCUAUGUAGUCCCGAACGCAUUUGAGGAAUUUUUCCUUGCCACCAACCUGGUCAAUUAAGGUUUGUCUUGAAGCCCUUCCUUGGGCAUUGAACCGGAAAGGGAACCCAGCAGAAUAAUUGCGGUGCCACUUUUUCCAGACUUCUUCGGGCGAUAUCAAUUGGGUGUCACCGAACAAGUGUUUCUCAUUCUGGAAAAUUGCCUCAGCAAUUUCUUCCUGAUCAGCUUCGGGUAUAAGCUGACCAGGGUCAAAGGAGCCAGUAAAGUAUCUAUCGGUAACUUUCUCGGCCAUUUCUACCGUGCCAAAGCGAAGAGAAAAAACUCCUAUUUCAAAGGGGUUUAUUUCUUGAAGCUCUUUGACGAGAGAAUGGGUGAGAACAUCCAUUUUUGGUAGGUCCUUUCGGAUGUUCACCUUAACCCCAAUCUCAUAUUCACCAGGUUUGACAAACUUGAAACCCAUCUUUGGAAACUCCGCAUCAGGGAAAAAUUGUGCAUAUUUCCUUGCUAUUUGCGGGAGGUCUAGACGGGUGAUUGUUUUCUUUUGCAUUUUCAAGUUUUCGGGCAGGUGAUU  >Ss-AA_clean.1_(paired)_contig_1333 UGGGGUUUAUGAUGUCCGUAGCCUGGAUGGUUUUGUUAAAACUCUAUAUGGUGGUCUUCCCCGUAGAAAGAAACCAGCCAGCAAGGAUAUAACUUUCAGGAAAGAGCCAGGCACUUUUGCCGUGCAAUCCAGAAAGAGAUAUUACCAGUUGGACUCGCGUUGUAUACCCACCGCAAAAUCACCUGCCGUUGCGCGUGCUUUGCUAGAUCAGAUAAGCUCGUUGGAAGAUGUGGCUAAUGUGGAGGCCUUUUGGAAGUAUACACUAACAAAGCCACCACAUUAUCAUUACAAAUCCAAUUCGGGCUUCUUAAAGCACAAGAUAGAAGAGCAAGAACGCAAGCAUCUUGACGUGACUCUCAGUGGUAUGGGGACAGAGGUUAUGUUCGCCCUUGGUGGUGUCCUUUCUGUAGGAGCCCCAAAGGACUUGGCACACCGCCUCUUCAAUAGACAUGUCUUGACAUCAAGUGGUCGCAGUUUUCUAUUUCGCCGUUGGUGGGUUCUGGUAGAUGCUCUCAGGCAUUUCGAUGAAUUCGUUCGUGAACUAGGAAUGAAAGCGAUGUAUGAUGUUGUGAGAGAUUUCUUCCCACCUAUACCUGAAGUGGAGUUCAAAAGUGUGCUACCAUACUCUUUUGCCUUGCACCAUCCUUUGAGGACACGUGCAGCUGGCAAAUUGUGGAUGCAAACACUGCCAAAUACAGAUGCUCGUCUGCGUGUACAUCUCUUCUCAUUACGCGCACACGGACUGGGUAACGCUUUUGGUCUUUUUCACGCUGUUAUUGAGCAUGAAGGAAACUACUGGGAACUACAACAGAUAGCAGGUGAACGUUGUCACAUCAACGUGUCAAAAUUUCCACCUGAACCGACAGCCGAUAGGCCGUUGGUCAAAACUAUAAUUGUUAGUUCUCAGAUAGUAGGUUCACUUGAUAGGAGAGCAAUAUGUCGUGAAUUUGACAAUCUACAAUACAAGGUCUUAGGUGACAACUGCCUUGUAUUUGCAAAUAUGCUAGUCUUUCUACUCACUGGAAAGGUGAUUCCAUGGAAACAUUUCGGUGCUUUUGGCACUGACAUAUCACUUGAUGCUUUCAAACUACUUGGAAAGUGGGCAUCUUCAUGGGUUUUCCUUAGCGAAGGGGAAGAAAGACUACAGAUUAGAGACAAUCGAUCAGCUUGCCUGACAUACGAUGGCCCGAUAGUGCAUUCCUUGAAAUCUUGGACUGGUCCGAAGAGGUUCACGAAGGAUUAUGGAAUACACGCCGUCCAACGUAUAGAAGCAUGUUUGGAAGCUUUCUCAGACGAUCCGGAUGUUGAAUGCCCUCAAGAGAGGGAUCAUUUACUUCAAUUUAUGUCUUUCGCAACCUCUAAAUUUGGCGUGACUGGGGCUACUGUAGCUCGUGCCAUCAUGACAAGAAGGGUUAGAAAGAUACCAACAUCUGGUAGACAAUGGAAAUUCCUCCACCACUUAGCCGUGCUGUUUAGACAAGCCAGAGACACCCGUCUGGGUGGCGACGUAAUUGGCUUAAUGACAGCAACGGUCAAUCUUCGUGGAAGCAUGCGAAAUGGAAAGAAAGUGAGCUGGACCCCUCUAGUGAACGUUUCAGUGCCUAGGCACUGGUUCCGCUCUGGAGACCGAUUGGUUGAAGUGAAUCACCUGCCCGAAAACUUGA  >Ss-AA_clean.1_(paired)_contig_6470  ACCGCGUCAGUGAAAGCUAUCUUAUUUUUUAAGUGCAGAUAUAACUCACUCACAGUUUCAAUCCAUGAGUCAAGCACAUCCCAUUGUUCAUUUUGAAAAUCAUAACCACGUUGCACUGCAACAUCGCGCUCACGCUUAAUAGCGUCAAAAUCCACCUUGUUCGUUUUUAAGUUAUCAUACAGAUCUUUACAUCGUGUAUUUGCUGGAAUCCCAACCAUCAAGCUGUAUAGUAACUCGACGUGAACAGCUGGCAUGUUAGCUGCUGCUGGCAGUGACAUGGCUGCGUAUCCUACGCUCGUCCUACCUGCGCGCCCCCUUGACUGAGUUAACUGCGUCAUAUCAUACUGGUGUCGCUUAUAGUUAAACAACCGCAUCUUGCUAAUGCCCUUAAUCUCGGCUUUAUAUGGCGGGAAUAUAAGCUCAGGGGUAAUCCUAUAUCCUAGGUCAUGCAUGUUCUCUAAGCCGGGUAGGGUUGAACCAGUAGUGAUACAGUUCGUACAUAUAGCUGAAUUUAUGGUCGCCAGUUCCAGAUUGGCUAACGUCUUAGAUGUGAUGCUAGCAAACUUUACACCACCAUUCACUGCUUGAGUGACAC  >Ss-AA_clean.1_(paired)_contig_13321  CCCUGGACAAAAUUUGUUGUCACAUCAAAUUUGCAGUUAUAAUCUAGCUUAAUACCAGGCAUAGUAGCUGAGCAGACGUAAGUCUUGUUUUUUGGUAGAACAGUCAAUAAUCUGGCAUAGUGAGGGCUAAUCUCAUGAACUUCAUCACAUAUGAUGGCACGUGCUGACACUAAUUGAUGAUAAAUAUUCUUCUUGUCAUGUC  >Ss-AA_clean.1_(paired)_contig_13322  UAAAUAUUCUUCUUGUCAUGUCCAAUAAAUAUACCUGAAUAUAAUGUCUCCAUAGUCAUUAUUACUAGGUCUGAUUUAUUUAUAUCUUUCAUGUCAAUAUUAUCAGGUAUCCAAGCGCCUUGAGCUCUAGCAACCACUUUCACUUUAUUGCGUAGAUAAUCAAAGCUAUUAUUUACUGCUGAACGCAGUGGUGUAAUUAGUAGUACUUUACCUUCAAUAUGUUGGAAUGCGGUUGUAGUUUUACCUGAUCCAGUUGGUCCAACAAUCAUCUUCACACUAUCUGACUUAUCAGAUAUUUGACACCUUGAAUUGCCACC  >Ss-AA_clean.1_(paired)_contig_7442  AAGUACAAUGAAGGCCAUUGGUAUAAAAAAAAUGAUGAUUGGACAAUUGAAUUUAAUGGAGAAAGUUUAGCAUACAGCCAAAGUAUUGCCCUCACUGAUACUUAUUUAUCUGUGCCUCUGCAUAAUAUUGGUGAGAUGGUUGUUUAUUGCAAGGUAUCAGGUUAUAAGGGCGCUCAUCUAAUAAUAGAACACUAUUUAAUUGAUCGAGCACAACUCGAUCAAUUGUAUUUAAAGCAUGUUAUGUGGUUUAAUACUAAUAUGGACGAAUUAUUUGUAAUGGUGCCACGUAUUAAUAUGGAUCAAUCAACCACAGUCAUGGGCAGAAUUCCAUACACUAUGGAGCAAGUUGUUAUUAACGUUCGAUUCUAUGAACGUCUUUUAAAUAGAUUGAUGCAGUCAUACACUUGGGAUAGUGUUUUGUCCUAUGCUGCUGGUCUUAUAGGUCGUGUUUAUGCAACAUCAAGCGGUUUACAUAUGAAGUGGAAUUUAACUAAUUCACAGGUGCGUGAUCAUUGCUUGAUCGCCUACUGGACUACUAAUCGUAUCAAUGAAUCGAUCAAGCCACUGCUUGCCCAGGCAGAACGUGCAUCCCGAGAUCCUGAUUUCUUAACUAGCUUGUGGAAUUCACUCAAAAACUGGCUUAAAGAUUUUGGCUCUCAAUUUGACCCCACUGGCAACACCACAGUCCAAAAAUUUAUAGCUGAUAAUAAAGGUGACACCCUUAGGCUUAUUCAAAUGUUCAAUAGUACCGUACGUAGUGUUGAAUCACUUAAUGUAGCAACACAGAUGGUACACUCAAGAAGUGUCGUAGACUGGGUUGCAUCCUCCACUGGAGUUAGCUACAGGCGUCUAGAUGUUGACAUUGACACUUGGCGAAUGAAACAAGAUAUGAGUGGCAAUAAAAUGACAGUUUAUGAUCAUAUUGAAUUUAAAGAUGCAAAGCCAGCAGGGGUUAAUAUUGGUUGUUUAACCACAAAGACAUGCCCACAUGACCACAAACCAUUCCAUCUACAUUUAUCUGAUCUCAACAGACGUAUGGGCAACUGUGCAUGUUGUGGUGUGAUCAGUAAUCUGAACAUAACAAAGCUAUGUACUAUUUGUUCCGAUAUACUACCUUGCCACGGAAAAAACUUACGAUGCACCCACACUCAUGAACAAUUAAAUGAUGAAUGUUGUUCAUUGGCUAAGUGUACUUGUAAAAAAGAUAAACAUUGUUCAUGCUGUGGUUUACCAUCUGUUAAUAUGUACUGUAAAGUAUGCCAAUUUUUACCUGACAAAUCGGUGAAACCGCAGCCAACCACAGCGGUAGAUGGCAGUAACAUCACCAUCGCACCUGUCACUAUAAAACCAACAACCGGGAAAAAAGAAGCACCUGUGCGUUUCACAUUCGAAAAACCACAGGCUACCAGCACAAAUGAUGAUGUGAAUGACAGCACAACAAUCAAUCAUCCACUUUCUAUUAACAGUAAUUUCCAAUUUGAUACUUUAAAGCAACAGGAAGUGGAUAAAAUUAUCACAUAUGAUGAUGAAACCACUAUCAAAGAAGAUAAUAUGGAAAUACAUAGUGUUGAUGCUGAGUCAGUUGGUGCAGUUGAUACCAAUAUUGAUGCUGGCCGAUCCAUUAAUAACGACGUUGGACCAUUAUUACCUGAAGAAGAUGUUGCUGGCAUUGAUCAAAUCAAUACGCUAAUGAGAGCAGUUGUUAAUGUUGAGGAUAUUAAUCCAGGGCAUGCUGAUACUAUACUAACGAGGCUACCACCACACUAUCAAGGAGGUCUGGCAGUUCGUGUUGUUGGAUACACCGACGUGCCAGGCGAUGGCACAUGUGGUGCCCACGCCCUAAGUGUUGCUCUUGGAUUGAAUGUAGAUGAUGUAAAACAUUGGUUAACCAUUGCAACUGGCCAUGAUGACUGGCAUAAUUCUGAGGAGUUAGCUGCUUGCGCUUUAGCAUAUGGCCGAAAUUUAAUUUGUGUUGAUAUUGGUGACACUACUAUAUACAGAGGUGGUGACCAAGACUAUGCUGGUUCUAUCAUACAUGGUACUGUCGUCGGUUGUGGUGCACACUGGUUGGCAGGAAAUUGUAAUAUCCUUAGUUACUCUUCACUCUUCACUAAUAAGUUAAUAACUGACUACACCAAUCUACUACGUGCAGCUCAUCUCGCAUUAAAUGGCAAUGACCAAACAGCCCUUGCAUACACACACAGCGAUUUUGAUAUUGAUAGUAUCAUAGACAUGGGUGGCAACAUUGUCAUUGGCCAUGACGGAAUUUCUAUAGAACAUGAAGGUGGCAAUUCAAAGUGUCAAAUAUCUGACAAGUCAGAUAGUGUCAAGAUGAUUGUUGGACCAACUGGUUCAGGCAAAACUACAACUGCAUUCCAACAUAUUGAGGGCAAAGUACUAUUAAUCACACCACUGCGUUCGGCAGUAAAUAAUAGCUUUGAUUAUCUACGCAACAAAGUGAAGGUGGUUGCCCGUGCCCAAGGUGCCUGGAUACCUGAUAAUAUUGACA  >Ss-AA_clean.1_(paired)_contig_13409  GUCUACAUUAAGUCCAAGCGCAACACUAAGGGCGUGAGCACCACACGUGCCAUCUCCCGGCACGUCGGUAUAUCCAACAACGCGGACUGCGAGCCCUCCUUGGUAGUGCGGUGGUAGCCUGGUCAGUAUAGUAUCAGCAUGCCAUGGAUUAACAUCCUCAAUAUUAACAACUGCCCUCAUUAAUGUACUGAUUUGAUCAAUGCCAGCAACAUCUUCUUCAGGUAAUAAUGGUCCAACGUCAUUAUUAAUAGAUUGAUCAGCGUCAAUAUUGGUAUCAAUUAUGUCAAUUGAUUUAGUACCAACACUAGGUGUUUCUAUUCCAUCUUCUUUAAUAGCAAUUUCAUCAUCAUACGUAAUAAUUUUAUCCACUUCUUGUUGUUUCAAAGCAUCAAAUUUAAAGUUAGUACUAGUAGAAAGUGGAUGAUUGACUGUUGUACUGUCCUUCACAUCAUCAUUUGUGAUAGUAGCUUGUGGCUUUUCAAAUGUGAAACGUAGAGGUGCUUCUUUUUUGGUGGCUGUUGGUUUUACAGCAACUGGUGCGAUGGUGAUGUUACCACCAUCAACUGUUGUAGUUGGCUGUGGCUUCAUUGAUUUGUCAGGUAAAAAUUGACAUACUUUGCAGUACAUAUUAACAGAUGGUAAACCACAGCAUGAACAAUGUUUAUCUUUUUUACAAGUACACUUAGCCAAUGAACAACAUUCGUCAUUUAAUUGUUCAUGAAUAUGGGUGCAUCGUAAGUUUUUCCCAUGGCAAGGUAAUCUAUCGGAACAUAUGGUACAGAGUUUUGUUAUGUCUAAAUUACUAACUACCCCGCAGCAAGCGCAAUUACCCAUACGUCGAUUGA  >Ss-AA_clean.1_(paired)_contig_7441  GCAACAAUAAUGUUAUCUUUGUUAGUGGCAGUGACUGGUUGUACUUUAAUCGAUUUAUCUGGUAGGAAUUGACAUACUUUACAAUAUGUGUCAACAGAUGGUAAACCGCAGCAAGAACAGUUUUUAUUUUUCUUGCAAAAACAUUUGGCCAAUAAACAACAUUCAUCAUUUAGUUGCUCAUGCUCGUGGGUGCAUCGUAAGUUUUUCCCAUGGCAAGGUAAUCUAUCGGAACAUAUAGUACAGAGUUUUGUUAUGUUUAAAUUACUAACUACCCCGCAGCAAGCGCAAUUACCCAUACGUCGAUUGAGAUCUGAAAGAUGUAGAUGGAAUGGUUUAUGGUCAUGUGGACAUGUUUUGUUAGUCACGCAACCAAUGUUUACCCCUGCUGGUUUAGCAUCUUUAAAUUCAAUAUGAUCAUAUACUGUCAUUCUAUUCCCACUUGCAUCUUGAUUCAAUCGCCAGGUGUCGAUGUCUACAUCAAGACGCCUGUAGCUGAUACCGGUAGAUGAUGCAACCCAAUCAACAACACUUCUUGAAUGUACCAUUUGUGUUGCGACGUUAAGUGAUUCAACACUACGCACUGUACUGUUGAACAUUUGGAUAAGCCUAAGAGUAUCACCUUUGUUAUCAGCAAUAAAUUUCUGAACAGUGGCGUUUCCAGUUGGGUCAAACUGAGAGCCAAAGUCUUUUAGCCAAUUCUUUAGUGAAUUCCAUAAGCUAGUCAGGAAAUCUGGAUCUCGAGAUGCUCGUUCUGCCUGAGCGAGCAAUGGUUUAAUUGAUUCAUUAAUGCGGUUAGUGGUCCAGUAGGCUAUCAAGCAAUGAUCACGCACUUGUGAAUUGGUUAGAUUCCAUUUCAUGUGUAAACCACUUGAUGUUGCGUAAACACGGCCUAUCAAACCAGCGGCAUAAGAUAAGACACUGUCCCAAGUGUAUGAUUGCAUUAAUCUGUUCAAGAGACGUUCAUAGAAACGGACAUUGAUCACGACCUGCUCCAUAGUGUAUGGAAUUCUGCCCAUGACAGUGGAUGAUUCAUCCAUAUUAAUACGUGGCACCAUGACAAAUAGUUCAUCCAUAUUGGUAUUAAACCACAUAACGUGUUUAAGGUAUAAUUGGUCAAGUUGUGCCCGAUCGAUCAAGUAAUGUUCUAUGAUUAAAUGAGC  >Ss-AA_clean.1_(paired)_contig_4912  GGCUAUAUGCUAAGCUCUCACCAUUGAAUUCGAUCGUCCAGUCAUCAUUCUUUUUAUACCAAUGGCCUUCAUUGUACUUUAGUGCACCAUUUCUGGCGUAUGAGUAAUUGUUUGGCAAUGUUAUAGCAUGCACUGUAUGGAAAACUUUGUUCCUUAUAUAAAAGUCAAACAAAUCAUUAACUGGUAAAUGAAACAAAGUGUCAAUGGAGAUACCAAAAGAGCCUUGCUUGCUGGAGUGAACACAUUUAUUGAUUCCAUCUGAGCACCAAAGGGCACUAUUGGGCUUGAGUAUUGAAUUAGCCACUCCAUUAACAACAGAAUGAUUACUGUCAUUAUUCAUGUUGCGACGUGCCCACGCCACACAUUGCUUUGACCAUUUGAUAUGUCGUGCAUUAUCAGCAGCCUGGUUUUUACUAUAGACACUGUGCACUGUAAAGCGACCAGCGUUAAGAUGGCUAGCUGGGUUACCACCAAUAUCAUAUAUAAGCGCAUCUCUACCAUGCCAACCACACAGUAAAUUUGUGACUGCUUUCCUACUAGCAUUAUGGUAAAUAUGUGGGUGGUGCUCUUUAUUACCACGAAUAAAGGUUGCAUUGCCAAACUCAUUAUUCAAAUCAUUAACAAUUUCUUCAGGGCAACUAGUUGGCAGAAUAAUUGCCUGGUCAGUAAAUUUAGUAACCGCUCUUGUGCCGCGGCUGUUAACGGAGUCAGAAAUGGCUUUCUUGAGCUGUUGUGUGUCAACAGAAUUAACAUCUACACCUAUGUCAUUAGCUAGUUUCUCUGUGAUAGUACGUGGGGUAAAACCAACCAUAGUACCCUCAUCAUACAAAUCCACAAGGUCAAUCAAUUUAAUACACCCGUCAAUUGGAUGUUCGACAAAUUUUCUCUGAGCAGCAAAUAGUUCAGGUGUCAUCUCUCCUGUGGGCAAUCCAGGCGGGUUUGCAAUAUGCAGCAUGCCGUUAGCAUAACUGGCUUGAAUUUGUAUAUGUGCUGCGUCUUCAGGCUGACACAUAAUAGUCAUUUUAAUCAUUUCAAGCGAGAUGGCAGUGUUUAUACCUAAAUCAUAACCAAAAAUGCACUGCCAACAGUUAUAACUAGCCAUAACUUGGGUUUCACCGCCAAGUUCCACUUUGCAAACUGCAUCUCCAGACGUAGACAGUUUUUUCCAUCGCGGGUUAGGCGUAAGUUUGGGCCAAUGUUUCUGUUGUUUAGGUGGUUCAACAACAUCAAACCAAGUUAAACCACGCUGAUGGUUUUUGUUUGGGUAAUCUUUUGAGAGGGACCCAAAAGCCCUCUUAUGUGUAAUUAUUUGUGCAUCGGUUAUUGUUGUAGUCGUGGAGGUUGUGUUUGUAAUUGUUUGCGACAUUUUGAUUGUGG  >Ss-AA_clean.1_(paired)_contig_14748  UGUCUGCAGCUUGGUUUUUGCUAUAAACGCUAUGUACAGUAAACCGGCCUGCGUUAAGAUGGCUAGCUGGGUUGCCACCAAUAUCGUAUAUUAGAGCGUCCCUGCCAUGCCAACCACACAAUAAGUUUGUGACAGCUUUGCGACUGGCAUUGUGAUAAAUGUGUGGGUGAUGUUCUUUAUUGCCACGAAUAAAUGUUGCAUUUCCGAACUCAUUAUUCAGAUCAUUAACAAUCUCUUCAGGGCAACUUGUUGGCAAUAUGAUUGCCUGGUCAGUGAAUUUAGUGACAGCUCUUGUGCCACGGCUAUUAACAGAAUCGGAUAUUGCUUUUUUGAGUUGUUGUGUAUCAACAGAGCUAACAUCAACACCUAUAUCAUUAGCCAACUUCUCUG  >Ss-AA_clean.1_(paired)_contig_7361  CUAAAUCAUAACCAAAAAUGCAUUGCCAACAAUUGUAGCUUGCCAUAACUUGGGUCUCACCACCAAGCUCAACUUUGCCAACAGCAUCUUUUGAUGUAGAUAAUUUUUUCCAUCUAGGGUUAGGCGUAAGUUUGGGCCAAUAUUUUUGCUGUUGGGGUGGAUCAACAACAUCAAACCACGUUGUACCACGUUGAUGGUUUUUGUUUGGGUAAUCUUUAAAGAGGGACCCAAAAGCCCUCUUAUGUGUAUUUGUUUGUGCAUCGGUUAUUGUUUUAGUAGUAAUAGUUGUGUGAGUAAUUGAUUGAGACAUGG  >Ss-AA_clean.1_(paired)_contig_5232  GAUGCAAUAAUCUGUGCCACUAACCAUCUAGCAAGAAUGUUCAGGAAAAUUACACAAAUCCCAGUAUAUAAUAUUGCUAAAUGUCAGGGUAUCGACACACCUAAUACAUUAGUUGUUUUACAGGGCGGGCAAAUUGAUAUGCUAGCCAACAAUCAGUUGUAUGUAGCUCUAACUAGACAUAGCCAAUGUGUCUCUGUAGUUGUUGACCCACCACAUGCAGCACUAUUAAUGCAAUUAUGUAUCAAAACAGAGAAUAUGAUAUCAUAUACUGAUGCGCUUGGUGGUGCAAGUAAUAUUACACAACCGAUUCUUAACCAUAUUGUAGAGUCGAACAAGAACAAUCUAUUGAAAGAUGUUCAAGUGAUGAAUUAUUUAACAACUAUCUGUGCUCUGCAGUCUGGAAAAAUUACACCAAACACAAUGAUUGUGAUCAUGGAUCAGAUGAAUUUCCAUUACAAGAAUUGCCCAUUUAGGUUUGUUAGAUUAAGAGAAAUUAAAGAUAUUAAACAAUUAGAGUGUGUUGUUAGCAUAUAUGGCAUCCAGUGCGUGGUGUAUGAUGUUUCCCUAACUAAUGAACGUGAAUUGCUCAGUUGUUUACUGAGCCAAGUAACAACAAAUAAACAUAUCAAAGAAUAUCUACGUAAUCUACCAAGGAAAUUUGUAACAAUAGGAAAGAAAAUUGGUUACAACUUAUAUCUCUGGCUUAGUAAAUUUAUGAAAGAUAAUAUUACUAUACUUAGUGGUUACUUCAAUACUAAUGAGGAUAUUAACAUUGGUGGCAGUACAGACCUAAGCCACAUAAUAUGGCUAGCGAUCAAUGGUGUCCAUAGUGCAUUUAUGACAUUAGUGCACAUAUUAGAUGAUGUUGGGAGUAAGUUGUCCAAGUAUACAGAUGGAAGUUACGCAGCUGAACAAUUCAAAGAACUGUUAAUCGAAACAUGGAAAAAGAGCAAUCCAUUUAGUUUCGGUGAUCUGAAGAUAAGUAAUAGCCUCAUGUCACUUUACAAAUUAUUCCGAGACCACCUCAGCAAAGGUGUUAACACAUUAUGGGAUUUCAUAAAAUAUGUUGCUAAUACACUGCGUGAAUGGUGGCAUAAACAAUUCAAUGUCAGUAAACCAACAUACUAUGAACUU  >Ss-AA_clean.1_(paired)_contig_16357  UAGUGGCACAGAUUAUUGCAUCUGGUUCAGAAGCAAUUAAUACUUUACUAUAUGAUUUAUUAUCAUUUAUUGCGCACACGUAACCUUUAAUUUCUUCAACUUUGCUCAUAUUGGUGGGAUUAAUCUCAAACCCUAACUUGGCAACUAAAUUACAUGUUUGUGGACCAAAACGGUGGGUUUUAUCAAGAUAAAUUUUGUUUGCAUACUUUGAAAACAAGUCAAUGCCACUGUCAAACCCAGUAUAAUAGGCAUUUGUUUCAUUGGCUGUAUGCACUACUUGACUGAUAUCACCAGUACCAGUUAAUUUGUUAGAUCGACUUGCAAGUGACAAUAUGACGGAAACAUCUAAUAACCCAACUUCAUCAAUAUUGAUAUUUUCUUGGCUAUGGGGUAUCAACGAUGGGGGCAACCAUGUACCAUGGUUUUUUAACUGUCCAGAGCUGAUAGUCGUCGUAUAUGAUGCUUUAUUCUCACUAACCAUCAUAGUUGUUUUACCGGUCCCAGGUGGUCCCACGUAUAACGUUGAGUUGCGUAGUGUGUCAAUCAAUGGUGUUUGGCGAUCGCGAAUUAGACCUAUAUCAGUUAACUGGCCCAUAUAAUGAUGUGUCUUGUAUGUUGGUACUAUAACGUGACCUACCCAUUUAUUAGAUUGCUGGUAGUGCGUAAUGCGCAUACUUGAUUUUUUAAUAUCAACUAGCCCGAUAAUGUCGUAUUCUGAUAAGUUAGUGCCUGAUAGUGCUUGCGCAUCUUCUAAACUAUCAUAUAAUAUGUAUUGGCCAUGUUGAUCACUCCAUUUAUUCGUGUUAAUAUAAAUCUGACCACGUGCCAUUAACCCUUUGAUAGC  >Ss-AA_clean.1_(paired)_contig_18898  UAGUGGCACAGAUUAUUGCAUCCGGUUCAGAAGCAAUUAAUAUUUUACUAUAUGAUUUGUUAUCAUUUAUUGCGCACACAUAACCUUUAAUUUCUUCGACCUUGUCCAUAUUAGUAGGAUUAAUUUCAAACCCUAAUUUGGCGACUAAAUUACAUGUUUGUGGACCAAAACGGUGGGUUUUGUCAAGAUAAAUCUUGUUUGCAUACUUUGAAAACAGGUCAAUGCCACUGUCAAACCCAGUAUAAUAGGCAUUUGU  >Ss-AA_clean.1_(paired)_contig_16358  ACACACCAUUAUAUGGGCCAAUUAACUGAUAUAGGUCUAAUUCGCGAUCGCCAAGCACCAUUGGUUGACACACUUCGCAACUCAACGUUAUAUGUGGGACCACCUGGGAGUGGCAAAACAACCAUGAUGAUUAGUGAGAAUAAAUCAUCAUAUACCACGACUAUCAGCUCAGGACAACUAAAGAACCAUGGUACAUGGUUGCCCCCAUC  >Ss-AA_clean.1_(paired)_contig_9741  AUAUCAGUAUGCUCUUCCAUAUAUUGCAUUAAUAGUUCAUGCAUUUUAUUAUGAUUGUCAACAUAUUUACCAACCCUGGUCAUACCUGACUUUAGUUUAUUACCAGUCAUUAUUACGGGUAGUUGUAUAAUUAUCUCUUCUGAAACUUGCUGCUUAGCAUUAAUUAUAUCAUUUAUUAUAUUAAACACUUGUAUAUCAUUUAUUUCAUUAUAUAGAGCAUUAGCUGCGACUCUUUGUGACAUUGUUGUUCCUAGACUUGGGAAAGACACAAACCCACUGUAUUCCAUUGCUUUCUUAGUUUGAGUUGAAUACCCAACCAUCAUUAA  >Ss-AA_clean.1_(paired)_contig_9289  GUUGAAAAAUCUAAUGUUUUUUUCAACGUCAGUUAAAUAUGGUUUGAUUACAACAUCGCUUGUUAUUCUGACUCCUAAAUCGAUGAUACUUGUAAUCUGUGGCAUGGUUAUACUUGUUGUCACUAUAUUAGUAGCUAUACAUCUAUCAACUUCAUUGAUAGAUACAUUAUUAAUCGUGGACGAAUUAAUGGCUUUAAUAUUUGCUGGCAUCUCAGCAAAUGUACAUUCAUUCUUUGUUCCAACUAUAUAACAUGU  >Ss-AA_clean.1_(paired)_contig_15674  GAAAUUGUGAGCUAUCAGGAACAGAUAUCACCAAGCUUUCAAAAUCAAUAUUGUCUGUUGCCUCAUACAAGUGCUUAGGUAGCACAUUAUCCCAAUCAAUAGUGCAACUAUAACGUAAAGCGCCAACUCUAUGUUUAGCAUGUUUCAAACUUGUUGAAAUUGGGUUAGUGCCAAUGUAGUUAGGUAUUAAAGCCAAGAUAUCCUCUUUGUUUAUGUACUCAUCAACAAACACUGGUUGUUGAUUGGAAUUUUUACACUGCAAUAACUCCGCAACUGAAUCCUGCCAUGAUUGAAACUCUUGCCACCAUGUGGGUUCGUUAUCAAAUGUAUUGCCAAGUGUGGUGAAAUAAUUGACUACUUCAUCAUAAUUUUUCUGAUUAACCAUUUGCCAUUCAUGUAGCAAACCACUGUAACAUGGAACACGUGUAUAACAACUAUUCAAUAAUGCAUCAAUAUUUGAUUGGCGCGUUGUCUUGUCAGGGCAUGGUCCAUAGAACAUACCAUUGGAGACACGACCAACUCUACCCCUAGCUUGAGUCAUCUCUGCCCAACUAUAUAAUCGUUUACUAUAGUUGAAAAAUCUAAUGUUUUUUCCAACGUCAGUUAGAUAUGGUUUGAUUAUAACAUCACUUGUUAUUCUAACUCCUAAAUCAAUGAUAUUUGUAACUCGCGGCAUGGUUAUACUUGUUGUCACAAUAUUAGUAGCUAUACAUUUGUCAACUUCAUUGAUGGAUACAUUGUUGAUCGUAGAAGAAUUGAUAGCUUUAAUAUCUGCUGGCAUCUCAGCAAAUGUA  >Ss-AA_clean.1_(paired)_contig_10459  AUAGAUAUCAUCAUAUCCAGGAUUAUUCCUCAUAAAUGAAUCUCCAAGUAUACUAUCAAUAUUAUCAACAAUUGUAGUUCCAUCCUCUAAAGUGAUAUCACCAGUUAAUUUUUUGUUUAAAUAAUUAAUAGUUACAUUAUACACAUCAUCAUAAUUCUGGGUGUGGACUAUAUUAUUCAUACUCUCAAGAUGAUUGACAUUUUUAUCAGACCAGAUUCUAUCUAAUGGGACAUCAGCACCAUCAUUUAUGAUUAUUAUGACUUUGUAACCAGCAGGCGCACAGAUAGGCGAAUGUAUAAAUACUGCAUCUGGUGGUCUUGUACUAUUAGCAACAUACCUUUCUAAUUUCUCUUUAUAAUUUAGCAUUUUGUCCUCAAACCCUAAGUGCAAUAUUGUUGCUUGUUCUUGUACUUCAUCAACAUCAAGGACUGUAAAUUCUUGGUUGCUUCUACGUAUUCUAUUAACUGUUGUUGUUUUACCAUGGCCACUGGGUAUACAAAAUGCAUACUUAAUGUCUAAUGGCUUAU  >Ss-AA_clean.1_(paired)_contig_9333  ACACGUGAGUGUAUUGGUGGUACAGUAUUGUCAACUAUAGAGCUAGUACCGAAUAAUGCUGCCAAUCGCAGUGAAAUUUUCAUAGCAAUAUACAACGUAAAUGGUGAUACACAAAUACUCAUUUUUACAAAUGAUAAUAAUUAUUCAAAUUAUAUCACACGUCGCCUAAUGACCCCAAUUGAACAUGGCGAUUUCGCUGGUGACAGAUCAGCAAUUAGUGCAUUGCUUAGACUAGGUAUUGAUAUUAAAUCAAUUAUGACACAAGAUAACGACCUAACCAUUAACGACAUUAAGAAGUGGCUACUAGCUAACCCACUCACGUCUAUGUUUGAUGUCACUGACCAACGGACUUACAAUAAUGCCAAAGAACACUCAAUUGUUGAAAAACAUUUAUUUACUGACAUCCAUAAUAUAUUGGAUAAGCCAUUAGACAUUAAGUA  >Ss-AA_clean.1_(paired)_contig_17844  UCAAUAAUGGCAUGACUUUGUUGGUUGAUUCACUUAAGAUCACUCGUAAAUUUAUUAAAAAAUUGAUUAGGUUGACACAUUACAAAUUGAAAUAUUUAAUAGGUAGAAUCAAUUUGCGCCAUCUUACAAGUAAUAGGUCAAACUUAGAAGUCUAUCGUACAAUAUGCCACAGAUUGCCUACCUCAUGUAGUAACAGUAAAUUCAGUACACGUGAGUGUAUUGGUGGUA  >Ss-AA_clean.1_(paired)_contig_14467  GUUGGUCAGUGACAUCAAACAUUGACGUGAGUGGGUUGGCUAGUAGCCAUUUCUUAAUGUCGUUAAUUGUUAGAUCAUUAUCCUGUGUCAUAAUUGAUUUGAUAUCAAUACCUAGCCUGAGUAAUGCACUAAUUGCUGACCUAUCACCAGCGAAGUCGCCAUGUUCAAUUGGGGUCAUUAGGCGACGUGUGAUGUAAUUUGAAUAAUUAUUAUCAU  >FirsU_Contig428  UUUCCUUUAAACGCACAAAAUUAUGACUUAAAUAGUAUGUUCCAUCUUCAUUUCGCGCAANCUAUCAUUUGUAAAAACAGUGCACAUUUUGUGCUAGCAACAGCAGUGUUCUGUACAUUAUNGUCGGCUUGCACAAAUACGUUUAUACAACUCUAAAUCAGGUUUUGUAUUCAUUAAAACAANGACUAUCAUCACCCAGAGUGUAUAUUGCCACAUAAUUUUCACCAAUACAAUCUGAUAGUANACAAUAGAUUACGUAUCUCAUUGCCCAUACUGGUCAUAGCACCACCACUAAAAUGUAUAGNCCGGUAGUUUAGCGGUCUCAUGGCUUGUACCAUUGAUUCGGAACGUUGGUAUAAAUGAAGNUCAUGAACUCAAUCAAUAUAUCAGGUACACCUAACAUACCCAUCAAUUUCCAUUCAUAAANUUAAUAUUUGUGCAUCUGUUUGUCUAUCUUGUUUUGAUAAAUCCAUCUCAAGCAACCAUCNUGCUUCUGUACUGACCAAUACUUUUAUUCAUAUCAGCAACUGUCAUACCGUCAGUGUAAANUAACAUUGGGUUUUAAUAAUGAUUUGAAACGUGCCUUGGCUUCAUUAAUGACUGGGCAUGNCAUACAUAUUAAUAUUUUGGUUAUUCCAUACAAUAACUCUACCAACUUGAUCAAGUAUAUNCAUUGACCUGCUCUUUCAUUAGAUUCUCAACUUUAUAAUGCACAUUAAUGUUAGCUGGAUNCAGUAGCAAACUCAUCAUCUUUAACAGAUCGCAACAUAUUGUCUAAUAGUUGUAUUUUAUNUACCUUUGGCUACUAACCAGGUGAGUAUAUCUUCAUAAUUCAAAUUAAUUGGGUCAUCUGNUAUAACUAGCAAGUUUAGAUUCAUAACCACUAAN  >Ss-AA_clean.1_(paired)_contig_10245  UUAGUGGUUAUGAAUCUAAACUCGCUAGUUAUACGGACGACCCAAUUAAUCUGAAUUAUGAAGAUAUACUUACCUGGCUAGUAGCCAAAGGUAAUAAAAUACAAUUAUUGGACAAUAUGUUGCGAUCAGUUAAGGAUGAUGAGUUUGCUACUGAUCCGGCUAACAUUAAUGUGCAUUAUAAAGUUGAGAAUUUAAUGAAAGAGCAAGUCAAUGAUAUACUUGAUCAAGUUGGUAGAGUUAUCGUAUGGAAUAAUCAAAACAUUAAUAUGUAUGCAUGUCCGGUCAUUAAUGAAGCCAAAGCACGUUUCAAAUCAUUAUUAAAACCGAAUGUCAUUUACACUGAUGGUAUGACAGUUGCUGAUAUGAAUAAAAGUAUUGGUCAAUAUAGAAGUAGAUGGUUACUUGAGAUGGAUUUAUCAAAACAAGAUAGACAGACAGAUGCACAAAUAUUAAUUUAUGAGUGGAAGUUAAUGGGUAUGUUAGGUGUACCUGAUAUAUUAAUUGAGUUCAUGACUUCAUUUAUACCAACGUUCCGAAUCAAUGGUACAAGCCAUGAGACCGCUAAACUACC  >Ss-AA_clean.1_(paired)_contig_14869  UAUUUGAUUAUGAAGAAGAAGCUAUUAAUUAUAACCCAACAGUUUGUAGAGAGCACCCUGAACCCACUAUAGAUGAAAUUGGCCAGACGCAAAGAUUUAGCUCUAGUGAACCUAUUGAAAAGAAAGAUGAUACGUCCUCAAUUGAUAUUGAGUAUUCAAAGCCAACUUUAGAUGAAUCAAUUCAGAUGGAUGAAAACAAUCCAAGUGUACUAAAUAAGAGGAAUGAGGAAAGUAAGCAAAAACUAUGCUUAUGCAUGGAAACAUGUNNUAGCUGAUAACAUAGUCACUAAUCUUGAUCUGAAUACUUAUAAACCAAACUUGAUGGGUAGGAUGGUUAUUGAGAAAAUACCAGAUGGUAUCAAAAUAAACUUAAAAUUACCAGGUGUUGGCAAAUUUAAUGCUAUCACUAAAAAAUGCUGUGGAGUUAAUACACAUUUGAUCUAUGUG  >Ss-AA_clean.1_(paired)_contig_14987  ACAUGUUUCCAUGCAUAAGCAUUGGUUUUGCUUACUUUUAUUAAUCCCCUUAUCUAGUUCAAUUGGAUUGUUUUCUUCUAUCUGAGUUGAUUUGUCUAUUGUUGGUUCUGUAUGCUCUAUACCAAUUGAGGACAUAUUAUCUUUUAUUUCUACAGGCUCACUAGAGUUAAAUNNGCUUCUUCUUCAUAAUCAAAUAGAACUUCAAUCUCAUCCAAGACUUCCUGUACAUCAUCAAGACAGUCUUCACAUGUUGGAGAAGGAGUAGCUUUCUUCUCCCAACCUAUUAUUGAAUUGUCAAUUAAAUUGUCUAGUUGUGUAAUA  >Ss-AA_clean.1_(paired)_contig_2947  GGUAUUUUAUUAACUGUUGGACGCAUCACUUCUGGAUGUGGUCCCGUUUUAAAAAAUCUUUGUUCACCUAAACCAAUAUUUCCUGUGCUCAUAUUUAAGAUAUCUUGUUUUCCGGUUGGUAAUCUAUAGAUAUCAUCAUAUCCAGGAUUAUUCCUCAUAAAUGAAUCUCCAAGUAUACUAUCAAUAUUAUCAACAAUUGUAGUUCCGUCCUCUAAAGUGAUAUCACCAGUUAAUUUUUUGUUUAAGUAGUUAAUAGUUACAUUAUAUACAUCAUCAUAAUUUCGGGUGUGGAUUAUAUUAUUCAUACUCUCAAGAUGGUUGACGUUUUUACCUGACCAGAUUCUAUCCAAUGGGACAUCAGCACCAUCAUUUAUGAUUAUUAUGACUUUGUAACCAGCAGGUGCACAGAUAGGCGAAUGUAUGAAUACUGCAUCUGGUGGUUUUGUACUAUUCGCGAUGUACUUUUCUAAUUUUCCUUUAUAAUUUAACAUUUUAUCCUCAAACCCUAAGUGCAAUAUUGUUGCUUGUUCUUGUACUUCAUCAACAUCAAGGACUGUGAACUCUUGGUUGCUUCUCCGUAUUCUAUUAACUGUUGUUGUUUUACCAUGGCCACUGGGUAUACAAAAUGCGUACUUAAUGUCUAAUGGCUUAU  >Ss-AA_clean.1_(paired)_contig_3829  GGGGGGGCACAGAAUAAAUACACAUAAUUUGUUGCUGUUGUGCAUUUUGGCUAUUCAUGGCUUGAGUAUUUACUUGAUUAAGGGUAGUACUCUGAGUAGUAUUACCCUAAGUGUCAGUAUGCUCUUCCAUAUAUUGCAUUAACAGUUCAUGCAUCUUAUUAUGGUUGUCAACAUACUUACCAACUUUGCUCAUACCAGACUUUAGUUUGUUGCCAGUCAUAAUUACAGGUAAUUGUAUAAUUAUUUCCUCUGAUACCUGUUGUUUAGCGUUAAUUAUAUCAUUGAUUAUAUUAAACACUUGAAUAUCACUAAUUUUGUUAUAUAAGGCAUUUGCUGCAACUCUUUGUGACAUUGUUGUUCCCAGGCUUGGAAAUGAUACAAAUCCACUGUACUCCAUAGCUAUUUUGGUUUGGGUUGAAUAUCCUAUCAUCAUUAAGUAUGAUGCGUACUUCAUUUUCCAAUCAGAGCUAGUGUUUGGGUACGUUGAGUAUACCAAUUUUUCUUUAAGACGCACAAAAUUAUGACUUAAAUAGUAUGUCCCAUCUUCAUUUCGGGCAACUAUCAUCUGUAAGAACAAAGCACACUCUGUGCUGGCGACAGCUGUGUUUGCUACAUUAUGUCGGCUAGCACAAAUGCGUUUAUAUAACUCCAGAUCAGGCUUCAUAUUCAUUAGAACAAGACUAUCAUCACCCAAUGUAUAUAUUGCCACGUAAUUACUACCAAUGCAGUCAGAUAGUAAUAAUAGAUUACGUAUUUCAUUGCCCAUACUAGUCAUGGCACCACCACUAAAAUGAAUAGCUGGUAAUUUUGCUGUUUCAUGGCUAGUGCCAUUGAUUCGGAAUGUUGGGAUAAAUGAUGUCAUAAAUUCUAUUAAUAUAUUUGGUACACCUAACAUACCCAUUAAUUCCCACUCAUAUAUCAAUAUUUGUGCAUCGGUUUGCCUAUCUUGCUUUGAUAAGUCCAUUUCAAGUAACCACUUGCUUUUAUACUGGCCAAUACUCUUAUUCAUAUCAGCGACUGUCAUGCCAUCAGUAUAAAUGACAUUUGGCUUUAAUAACGACUUAAACCGUGCUUUGGCUUCAUUUAUGACAGGGCAAGCAUACAUAUUAAUGUUUUGAUUAUUCCAUACAAUCACUCUGCCAACUUGAUCAAGUAUAUCAUUAACUUGUUCCUUCAUUAGGUUUUCAACCUUAUAAUGUACAUUUAUAUUUGCUGGAUCAGUGGCAAACUCAUCAUCUUUGACAGAUCGUAACAUGUUAUCUAAUAAUUGUAUCUUAUUACCUUUAGCAAUUAGCCAGUUGAGUAUGUCUUCAUAAUUCAAAUUGAUUGGAUCAUCAGUGUAACUAGCAAGCUUUGUUUCAUAACCUUUAAUAAAGAAUUUAGUCAUUAAUUUAUGGUAAUCCUUAAUGGCUAAGUCAACCGUCCUAUACUUUACACGACCCAUCAGCCUGGUCAUAAUUGCAUUUGGUAAAGCUGAAGGUAUUUUAUUAACUGUUGGACGCAUUACUUCUGGGUGUGGUCCAGUUUUGAAAAACCGUUGUUCUCCAAGACCAAUGUUUCCUGUAUUCAUAUUUAGAACAUCUUGUUUUCCAGUUGGUAGUCUAUAGAUAUCAUCAUACCCAGGAUUAUUCCUCAUGAAUGCAUCUCCCAGUAUACUAUCUAUGUUAUCUACAAUUGUAGUUCCAUUCUCUAAAGUGAUGUCACCAGUUAGUUUUUUGUUUAAAUAGUUUAUUGUUACGUUGUAUACAUCAUCAUAAUUUUGAGUGUGUAUGAUAUUGUUCAUACUUUCAAGAUGGCAGACGUUUUUAUCUGACCAGAUUCGAUCCAUCGGAACAUCUGCACCAUCGUUUAGAAUUACUAUAACUUUAUAACCAGCAGGCGCGCAAAUAGGUGAGUGUAUGAAUACCGCAUCAGGUGGUUUUGUGCUAUUGAUAAUGUAUUUCUCCAAUUUUGCUUUAUAAUUUAACAUUUUAGCUUCAAAAUCUAAGUGCAAUAUCGUUGCUUGUUCUUGUACUUCAUCAAUAUCUAGAACUGUGUAUUCUUGAUUACUUCUUCGUAUUCUAUUAACUGUUGUUGUUUUACCGUGACCACUAGGUAUGCAGAAUGCAUACUUGAUGUCUAACGGUUUAUCUAACACAUUAUGGAUAUCCGAGAACAAAUGUUUUUCAACAAUUGAAUGCUCUUUUGCGUUAUUGUAAGUCCGUUGAUCAGUAACAUCAAACAUUGAUGUCAAUGGGUUGGCUAGCAACCAUUUUUUGAUAUCAUUAAUGGUUAGAUCAUUAUCCUGCGUCAUAAUUGACUUAAUAUCAAUACCCAAUCUGAGCAGUGUACUGAUUGCUGAUCUAUCACCAGCAAAGUUACCAUGUUCAACUGGAGUCAUCAAGCGUCGUGUGAUAUAAUUAGAAUAGUUAUUAUCAUUGGUAAAAAUGAGUAUCUGUGUGUCGCCACUUACAUUGUAUAUAGCAAUGAAAAUUUCACUUCGGUUAGCAGCAUUAUUUGGAAUUAAUUCAAUAGUUGACAGAACUGUGCCACCAAUACACUCACGUGUGCUGAAUUUGCUGUUGCUAUAUGAAGUCGGCAAUCUAUGGCAUAUUGUACGGUAAACUUCUAAAUUUGACCUGUUACUCGUGAGAUGGCGCAAGUUGAUUGUACCCAUUAAAUACCUUAAUUUAUAGUGUGUUAACCUAAUUAGUUUCUUGAUGAAUUUCCGGGUAAUCUUGAGUGAAUCAACCAAUAAAGUCAUACCAUUAUUGAGUUUGCCACCUCCUAAUUUACUAAGCAUUUUAAUAGCUUGUUCAAGCUGUGAAAAGUUAACAUCAACAACAUUAGGCAUACAAUAUAAUGCAUAGCCAGAAACUGUUUUUUCAAUUUUCAAUGUCAAUUGCUCACCAUUAACUGUCACAUAUAUUAGAUGAGUAUUAUCACCACAGCAUUUCUUGGUAACAGCAUUGAAUUUUCCAACACCUGGCAAUUUCAAAUUUAUUUUAAUCCCAUCAGACAUCUUCUCAAUUACCAUCCUACCCAUUAAAUUGGGCUUAUAAGUGUUUAAAUCAAGGCUAGUAACUAUAUUGUCAGCUAAUUGCCAAUCAUCACAUGCUUCCAUACAUAGGCAUUUAUCUCGCUUGUUAUCUUUUUUAUCUUCUAACUCACUGGGGUUGGAUUCUUUUAUGUGAUGUGGUUCAUCUAUGGCUGGUUUGGAACUCGUACUAUUGGCGAACGAGUUAUGUUCAUUGCUGGUUUCAUCAUCAUAAUCAAACAGUACGUCAAUUUCAUCUAAUACUUCCUGAGUAUCAUCUACACAAUCUUCACAUGUUGGUGAAGGGAUGGCCUUCUUCUCCCAACCUGUUGUAGAAUUAUCAACCAAGCUAUCCAGGGGUGAAAUGUAUUGUGUGUUAGUGAAUAUAGGAGAGCCAUCAAUCAGCGAAUGAUCACCAUCCAAGCUAUUUCUCAUCAUGCUAAUGCAUGGCGAAUCGGUAGCAAUUAAUGACAUACUAGUGCCUUCAAGUUCAUAGUAUGUUGGUUUAUUUACAUUAAAUUGGUUUUGCCACCAAUUUCGUAUGGCACUCACUAUGUAUUGGACGAAGUCCCAUAAUGUAUUGAUGCCAUUCCUGAGAUGAUCACGAAACAACUUAUAGAGUGAAAUAAGACUGUUGCUAAUUUCCAAAUCACCCAAACUCAGUGGAUUGCUUUUCUUCCAUGUAUCAAUUAGCAGCUCUUUAAAUUGUUCAGCAGCAUAACUCCCAUCUGUAUAUCUGGAUAAUUUGCUUCCAACAUCAUCUAGUAUGUGUACCAGUGUCAUAAAUGCACUGCGAACACCGUUCAUAGCCAACCAUAAUAUAUGGCUCAAGUCAAGUCCGCCACCAAUAUCAAUAACCUCAUUAGUAUUAAUGUAGUUGCUUAGUAUAGCGGCAUUGUCUUUCAUAAACUUACUGAGCCAAAGGUACAAGUUAUAACAUAUUUUCUUUCCUGUUGUCAUAAACUUACUUGGUAAACUCUUAAGAUACUCUUUAAUAUGUUUGUUUGUCGUCACUUGGCUCAAUAAGCAACUAAGUAAUUCCCGUUCUCCAGUCAAAGAGACGUUAUACACCGCGCACUGGAAACCGUAUAUGCUAACAAUGCACUCCAACUGUUUAAUAUCUUUAAUCUCUCGUAAUCUAAUAAAUUUGAAUGGACAAUUCUUAUAAUGCAGAUUCAUUUGAUCCAUGAUCACAAUUAAAGUAUUGGGUGUGAUUUCCCCUGAUUGUAACGCACAGAUUGUCGUUAAGUAAUUCAUUACUUGAGCAUCAUUCAGUAAAUUAUCUCUAUUGGAUUCAACAAUAUAAUUAAGAAUUGGUUGUGUAAUACUACUUGCACCACCUAGUGCGUCGGCGUAUGAUAUCAUAUUUUCUGUUUUGACACAUAGCUGAGUUAAUAGAGCUGCGUGCGGUGGAUCAACAAUUAUGGAAAGGCAUUGGCUAUGUCUAGUUAAAGCAACAUAUAACUGAUUGUUGGCCAACAUAUCAAUUUGUCCACCUUGCAGUACAACUAAUGUGUUUGGUGUGUCAAUGCCCUGACAUUUAGCAAUGUUAUAUACAGGGAUUUGUGUGAUUCUCCUAAACAUUCUCGCUAAGUGAUUAGUGGCACAAAUUAUUGCAUCAGGCUCAGAAGCAAUCAGCACUUUACUGUACGAUUUGUUAUCAUUUGUCGCACAAAUGUAACCUUUGAUUUCUUCAAUUUUAUUAGUAUCGACGGGAUUAAUUUCAAACCCUAAUUUUGCAACUAAGUUGCAGGUCAGUGGUCCAAAACGAUGGGUUUUGUCUAAGUAAAUUUUAUUGGCAUACUUUGAGAACAAUUCAAUUCCACUGUCAAAACCAGUAUAGUAUGCAUUAGUUUCAUUAGCAAUAUGCACAACUUGACUGAUGUCACCUGUGCCAGUUAACCUGUUUGAUCGGCUAGCAAGUGAUAGUAUAACUGAGACAUCUAAUAACCCAACCUCGUCAAUGUUAAUAUUUUCUUGGCUAUGUGGUAUUAAUGAUGGGGGUAACCAUGUCCCAUGAUUUUUCAACUGUCCAGAGCUAAUUGUUGUGGUAUAUGAAGAUUUAUUUUCAUCAAUCAUCAUAGUUGUCUUACCACUUCCUGGUGGCCCCACAUACAAUGUUGAGUUACGCAAUGUGUCAACUAAUGGUGCUUGGCGAUCACGUAUUAAACCAAUAUCUGUUAAUUGGCCCAUAUAAUGGUGUGUCUUGUACGUUGGUACUAUGACAUGACCUGUCCACUUAUUUGAUUGUUGGUAAUGCGUAAUGCGCAUACUUGAUUUUUUAAUAUCAACUAAUCCAAUUAUAUCAUACUCUGAUAAAUUGAUGCCCGAUAAUGCUUGAGCAUCCUCUAUACUAUCAUAUAAUAUGUACUGGCCAUGUUGAUCGCUCCAUUUGUUAGUAUUGAUAUAAAUUUGUCCACGUGCCAUUAACCCUUUGAUAGCAUUUAAUAUACUUGCUAUAGCUAAGAGCCGUAAUUUGGAUGCUAUAUCUUCAUUUGAAUCUGUUUCAAUAUUGAGAAAUUGUGAGCUAUCAGGAACUGAUAUUACUAAGCUUUCGAAGUCAAUAUUAUCGGUUGCUUCAUAUAGAUGUUUAGGUAAAACAUUAUCCCAAUCAACUGCACAACUAUAACGUAAUGCACCAACUCUGUGUUUAGUGUGUUUUAUACUCGUUGAUAUUGGGUUUGUUCCAAUGUAAUUAGGUAUUAAUGCCAAAAUAUCUUCUUUAUCUAUAUACUCAUCAACGAAUACUGGUUGUAAAUUGGAAUUUUUGCACUGCAAUAGCUCUGCAACCGAAUUUUGCCAUGAUUGGAAUUCAUCCCACCAAAUUGGUUUAGUAUCAAAUGAGUUGCCGAGCUUGUUGAAGUAACUAACCGUCUCAUCAUAGCUUUUCUGGGUGACCAUUUGCCAUUCAUGCAGCAAACCACUAUAGCAUGGAGCACGCGUAUAACAACUGUUCAAAAUCGCAUCAAUAUUUGAUUGGCGCGUUGUUUUGUCAGGACAUGGACCAUAAAAUUGCCCAUUGGAGACACGGCCAACUCUACCCCUUGCCUGAGUCAUCUCCGCCCAACUAUACAAGCGUUUGCUGUAAUUGAAAAAUCUAAUAUUUUUCUCAACAUCGGUUAAAUACGGCUUGAUGAUAACGUCACUGGUAAUUCUAACCCCCAAGUCAAUAAUGUUUGCUACUUGUGGCAUAGUGAUACUUGUUGUCACAAUAUUAGUCGCAAUACAUUUGUCAACUUUGUUAAUAUCAACAUUAUUGAUAGUAGUUGAAUUAAUUGCUUUAAUAUCAGUUGGCAUUUCAGCAAAUGUGCACUCACUCUUUGUUCCAACUAUNNNNNNCACGUCAAAUUUACAAUUGUAAUUUAGUUUAAUGCCAGGCAUAGUGGCUGAACAAAUAUAUGUUUUGUUCUUUGGUAGAACAGUCAGUAGCCUAGCAUAAUGAGGACUGAUCUCAUGUACUUCAUCACAAAUGAUAGCACGUGCUGAUACUAGUUGAUGAUAAAUAUUUUUACCAUCCUGGCCAAUGAAUAUACCUGAAUAUAGUGUUUCCAUUGUCAUUAUUACAAGGUCAGAUUUGUUGAUAUCUUUUGUAUUUAUAUUGUCAGGUAUCCAUACUCCUUGGGCGCGGGCAACCACUUUAACUUUGUCAAUUAAAUAAUUGUAACUAUUGGUUACAGCUGAACGAAGCGGUGUGAUUAGUAGUACUUUACCUUCGAUAUGUUGGAAAGCGGUAGUAGUUUUACCAGAUCCUGUUGGUCCAACAAUCAGUUUGACAUUAUCAGACUUAUCAGAUAUUUGGCAUCUUGAAUUGCCACCUUUAUGUUCUAUGGAGAUCCCUUCAUUGCUUAUAACAAUACUACCAUUCAUGUCUAUAAUACUGUCAAUGUCUAAGUCACUAUGUGAGUAAGCUAGUGCAGUUUGAUCACUACCAUUCAGGGUAAGAUGAGCUGCUCUUAGUAGAUUGGUGUAAUCAGUCAUCAAUUUGUUUGUGAAGAGCGAGGAAUAACUGAGAAUGUUGCAGUUACCGGCUAACCAAUGCGCACCACAACCAACCACAGUACCAUGUAUGAUAGAGCCAGCAUAAUCUUGAUCACCACCUCUAUAAAUAGUGGUAUCGCCAACAUCAACACAGAUUAAAUUACGGCCAUAUGCUAGAGCACAAGCGGCUAAUUCUUCAGAAUUAUGCCAAUCGUCGUGACCAGUUGCGACAGUUAACCAAUGUUUCACAUCAUCCACAUUAAGUCCAAGUGCUACACUUAAAGCAUGUGCUCCACACGUGCCGUCACCUGGCACAUCAGUGUAACCAACAACGCGAACGGCUACACCUCCUUGAUAAUGUGGUGGUAGUCUUGUUAAUAUUGUGUCAGCAUGUCCAGGGUUAACAUCCUCAACACUAACAAUUGCCCUCAUUAAUGUGCUAAGUUGCUCGGUGCCAGCAACCUCCUCUUCAGGUAGAAGUGGGCCAAUGUCAUUACUACUAGGUUGGAUAUCAUCAUUAUUA  >FirsU_Contig294  UGUCAACUGUUGCAAUAGAGUCAGCAUCAACACUAUGUAUCUCCGUACUAUCAUUAUUAANUGAUUGUUUCGCUAUCAAAUGUAAUAAUCUCAUCAACCUGCUGUUUUCUUAGUAUAUCAAAACUGAGAAUUUGUGCUAUCAGCAAGUGGAUGGUUAGUUGGUGUAUUAUCUUUAAUACCAACAAUCAGGGUUAAUAACUUGCGGCUUUUCAAAUGUAAAUCUAGCUGAUGCUUCUUUUUUAACCUGUGUUUGGUUGUAACGGAAUGGGCGCAACAAUAAUGUUAUCUUUGUN  >Ss-AA_clean.1_(paired)_contig_1364  TTGCAGTCCACATTATCCCCAGCAAACAAAACGTTTCTTCCTTTTACACCTAGAATTGGCACAGACGTAGAGATACCCGGATGTTTGCAACCATTAGTTACCCACACAGTGATGGACTTATTTGATGACACCGACTTAACGCATTGGAACACAATGATAGGTCCAACCACTGGGGCCATCAAAACTACTGAATCCATAAATGAGTTTGTCAAAACGAGTAAAACACTATTGACGGATTATCCTGAGATGTCTAGGCCTGTGTTGACTAAGGGTCCAAACCAGACATTTAACGCTGTAAGCACAAGGCTACATTCTGTCGTAACAATGCGCAGGTATGACATAAATCCAAAAGAAGAGTTCTTGGGCATAGCCACTGCCTTCTTCAGGAGAGATTGGCAAACACAAGTGAACACCTGGAGGCACAACAACCCCCTTAACTTTGACGGAGAGGAGATACGATCTTGGCTCAGGGAACGACCAGGTAGTCTGAAAATTGACCAAGAATTAGAGTCCATACTATCAGAAGGGCTGACAATCCACAGACTCAATGACTTGAAAGTGCACAACAAGCTAGAGTCGTTACTCAAAGAGGACGTACAAGAACAACCATTCACACAACAGAAAATAAGAATCATTGTATGGCAAATGAAAGGCATGTGCGCCATGTTTGCGCCGGTTTTCCTAGAAGCAAAAAAACGACTCAAGGCGCTATTGAATGAAACCACTATCTATGCGGATGGCTTGACCCCTGACGAACTGGCACAGAGACTCAAGTCTGTCACAGGCGCTGAGTGGTTTTTCGAAGATGATGGAAAGAAACAAGACAGACAGACCGACGGACCTGATCTGGAATGCGAATTTTACATATATGAGCAACTCGGCGTCCACCCCAGGGTGCTGTCGCTATGGAGACAAGTGCACAGACACTGGAAATATAAAGGAACGAACGTGTCTGGCGTACTAGACTTCATGCGAATGACCGGCCAAGCAACCACTGCTCTGGGAAACGTCATCATTAACATGCTGATTCACTGGAGGATTGTCAAGGAGAACGCAGGTCACATTAAAATTAACTTGCTACTAGGTGATGACAACTTGATTGCCAGTGCAAAGAAGATAGACTCTAGCCAGCAGAAGAAAATCGGCAAAGACTATTACAACATGGCCTGCGAGAGCTTTCATAGGAAGACCTATGGTACATTCTTGCAAATGGTGGCTTATCACCGGGAAGATGGCACGCTAGGCTTAGGTCCAGACGTCCACAGACTGCGTCAACGATTTGAAGTAACTAATGGGGTGTCCAAACTAAATGATGACAACATGATAGCGCGTGCGATGAGCTACGCCATGATGCTTGGTCAAAGTTCTGCCATAGATAACATGGTAGCGATCAAGAACTGGCCCATTAAACCCCTGTTATGGCATGATTTAACAGCATCCATACAGGCCAGCGCAAGAAGGCATGGATTGACCGTGGCAGAGGTGGAAAACGATTTCAAGTTGCTCTGCTCATACATGAGTTCACCAACTATAAGAGTCGTAGAATTCCAACATGGAACCGAAGCAAGACACAAACAGAGGAAGTCTTGATTTTTAAAATAAATTGTAATGAACGGA  >Ss-AA_clean.1_(paired)_contig_282  CCAUGGCCUACCUCGGGAGAACACUGAUAUGGACUGGCCUGAUGUUUCCCUGUGCAAAGCGUCGUUGAGGCCCUUCUCCAGACUGCUGUUAAUGUGAGGUGGUAGUGUUGAACCCUGCAGGGUCAAUUUGUCCGUUCGUUCAAAGGGUCUUGCUGAGUUGGCGUAUGCUAACACCAUUGCCAGUUCGCUCAACGUGAUGCUGCGGUAUCCGUCAUCGGGACCCAAUACGCCAGGGAACUGUAGGAAUUGCUUAUCAUCGACCACACGCAUCUUAGGAGUUAUUGGGUCCUCUGUGCUUAUAGCUGGAUCUGCUGUGUGAUAUGUUACUUGUGUGCUGGCAUUUAACCGAAGGGCUUUGCAGUGUUUGACCGAGGUUGUGCCAGUUACUUGCACGAAUCUGAUCUGCCUAGUGGCAUCUUGAUGAGAAAGCUUUCCCACAUCACCAUAGGCUAUAGCUAAGGUGAUGCCCAUGACUGCUGCCAUGGUUGUUAGCUGAUCGGGUGUACAGUAGGCUGGCAACCCACAAGCUAAUAUAUCUGUCAUCGCAGACUCUGCAGUUUCAUACAGACUGGUGGUUUGUAGGUGGUAGGCUAAACAGCGGUGAGCACACCACCCAUCAUCGGUGGGGUUCAAUGCUAUUUGAGAGCUCUCUAUUGUGAAACUGUACGUACCCUUCUUCAAUCCUGACGGUUGGUCAAUUAAUGGUUGCCUACGCAAAAAUCUGUAAGAUAAAGGAUUCUCUGGUGAUGGCACGCCGAGGGUUGACAAUGCAUCUUGCGCGUUAAUUAUCACGCUGGCUGUAGUUUGCUGGUCAGUGAACUCGAGCGCAUCGGGUUGCUUGACUCCUACUGUUUUGUUGUCGGCAUGUUUAAUAUAUUUGUCCCUCCAGUUGUUCAUAAUGUCACUGGUGGGUUGGUAGUAAUCACUAACUGAUACGCACAUGGAACAUCGGUGUGAACGAUCGCACGAAGUUUCUAUGUCACAAACGCAGCAUGGUUCUAUGACUAUACCUGUGGUAUUGUCCCUGGUUCUGGACUUCACACUGUUUUUAAUGUCUGCCAUAUAGGUGUCGACGUCUAGCAAUAUCGGCUGGUUGGCUGGUAUGUGUAUAUCCCAUUUCCGGUAAUUGCCGAGUUGUUCAGGGUUAAUCACUCCGUGGGCGUGCAGUGGAUAUGUGGCAAAAGAUGGUGGACCUCUAGAAGUACCAUAUGGCAAUUGUGGUUGUUGGAUUGUGUGGGCCCAAACUGUGUAUGAGAUGUGCCACUUGUUCCUCACGCAAGUGAACGCUAUUGCUACGUCUUUUAUGUAGCCCAGCAGCCAUUCACCAGGCUCUAUUGAAACUUCAUUCUCUGGGUUACAACAUGGUCUGUGUACACAAUAGGCCAGUUGCGCAUUAUAUAGCUCCAUGUCCAUCAAACUCUGCUUGUGUGUCUCCUUGAACUCGAGCCAGGCUUUGCUGACCUCGCUGCUUGGCACGGAGCGUACUAACACACAAGUUGAGCAGUUAACUUUUGCAUCUACUGAUGGCAUCGUCAACUUGAGCUGGUAACCUUUUUGCCGGUAACUUUGUCUUAGGUUCUUCAUGAACGAAAUGCUGGAGUCUGUGUUGGUUAGCUGUGGCAGGUGUGCCAGCACACCGGAAGCUGCCUCAGCCUUCACUAAAGAAGUCUGUAUGAUAUCCUGACUUGGGCUUCUUGGUACAUUGUGGCCCAGAGCCGAACUUGUUACUGCGUAUGACCAGUAAUCCUGGUCAUAAGCUACGGGCGUUAUGCAUUGAUACACUCGUGAUCGUACACAUUCUUGUGUUGUGCCAGCUCCUCCAUGGUGGAAGACAACUUUUGAUUUAGUAAAGUAUGUCCUAUGGUUACCUGAUUUUAUGGUUGUUAGCAUACCUGGGUGCAUUUGUUGCAGGUCGGUGAUCACUUGGCUCACUUUGUCUGUUAGACCUCCAGUGAUCCAGAUCACCUGGGAUCCCAGCUUGUUCAGUGCUGUAAUUAAUUUCAUGCAAUGGUCCACUAGCCAGUCAUCGACCAUAGAACCAUAAGUAACUGUUACUAUGUUUGAGGGUUCCAGUUGGAUAGGGUCACGCACUGACAUGCUAGGCAUAGUCAACUCAGAAUGAGAAGAGUAGGGUGAUUGAAAUUGGCCAGCUUGCAGGCUCAAUUUGACAAUAUUUUCAUCUAUGAGGUAAAUCCUGUGUGGUGUGUAAUCAAAUGCGGAAGUGGUGGUUAAUGGUGGCAAUUGCAGCUGCGACCUGAGCUCGUUAACACCAUCUAGAUGCAUGAUUUCUAGCAUUGGUUCUUGUAAGUUCAUCCANNNNNNNNNNNNNNNNNNNNNNNUAUUCGGCUAGUGUAGCAGCUUGGGGUGUGAUGGUGGUUCCCAUCACUAGUUGAUACUGGCCACGGUGUGACACGACUUCUGCCGGUAAUUCUGCAUAAUUCAGCUGUUGGGAAAAAUUCUUGAUCGCUUUGUCUAAGUCUAGCAAUGAACCUGAUUUUAGUUUGGAAUAGCUUUCCAAUUGCUCAGAGAUGUUGAAGUCUGCGUACAAUACCCGAAUUUUCCCAGUAGCAUCAGUUAUGUUAGAUUGCCUUGGCACGACCAUUGUGAAUGAAUUGAAGAAUGGACUGAUCAUGAUGGCCAGCGUCAGCAAUGGCAAGAUGUCGCCGGUAGAACCUAUAGUUAUCAGUAAGACAUUCAUACUACUGGUAGUUAGCUCAUUCUUUUCAGUGUAAGUUGGCCGUGGAUCUAUCAGUUCACCUUCUCUAGUUGAUCUUCUUGCUAUGAGUGGUUUACUGCUAAGUGCAGUGUAAGUGCUAUUGUCCACCCAUGGUUCGCCUCUGUCUGUCCGGUCACGCAUCAAUUUGGGUAGUGAGGUCAUGGUUGUCCAAGCCAGUCUGGUGACCAACUUAGCUAAGGGUUUAUCAUGGAUCUCACUUGAUCGUUGACUGGUACUAAGAAUCAGCGUAGUGCCCUGAGUUGGGUAAACUGCUACUACUCUUCUUUGCUCUUUAACGACUUGUAUUUGCAUUUUUCGUAAGUUGCUUGCAGUCUUUUGUAGGUGGCCUUCCUUACUAGAUAUUUUUGCAUCAGAAAGUAAUUCUAACCAUUCAUGAACUUGUAGAUCCAGCCCUGCUGUCUUGGCCAUGGUUGACAAUAUUGCCAAACCAUUGUCUUUUAGGUGUUCUACUGUGGGGGCAAGCAACUGACUUAACACCUGUUCUUUAUCUCUGCUAUUGUCCAUCAAUUCAGCGGCCCUCUUGAUGUCCUUAACCAUUUUUCCCUGGCUUAACCAUGCGGCCAGUGCAGCAUCCAGUGCUUCUGACGGUGACUCGUGGUACUGAUCUGUUAAUCCGGAUUCGGAGAAGUAGCGUGCAUGCAACAAGACUCUAGCAUACACUACCAAAUCGUCAAACGUGGUUCCUUCACGCAAGCAACGUAGACACAUCGAUCUGUACAUUUUCUUGUCUAUUUGAAUGACACGUGAUUCGAUCACUGGCCUCCUGGUCAGUUGUAGAGCAUCAGUGGUUAGGACUGGGACACGUACCUUGAUUUUCCGGCGGCUAGUUUGGCCAGAAAGCGUUGCGUGUAACCAGGGUCCAGCUAGGGCAGUGGGCUGCAUUAUUAUCUUGCUUACUCUGAUGAUACCAAUGUCAUGCUCUGUAGUAGCUCUGAAAGUGUUAACACCAUUUGUCCAUCCUCCUUGCCUUUCUAUCAUACCUGGUGCUUCACUGGUUGGCAUUAGUUGUGUCGGCGUUAUUGGUCUGUAUCUGUGGUUUUGCAGAAUUAUUUCCUUUGAUGUUGGGAAAGCGUAGAAAAUCUGGUCGUGAUUCGUGCUCACUAAAUUUUCAAUUUCAGCUAGAGAUGUGUUACUUGCAGAUAGGUUGAUUAACGUGGUGCAAGACUCAGGUAACUUGUUCAUCACAUACUUCUCGUUGGAGCCAUCGGUGUGGCUAGCCAUUUGUUUGGCGAGGGCUUGGAACGCUGUUUCGUCCGCAUUUACAUCUGUCUUGGCUGACCUGAUUAUUUUUGUGAUUAUGGUCUCUGCUAGCAUGCAGUUCUUUGUUAUGCCAGAAGCAAACUGGGCAUCUUUGCUACAAAUGAAAGUUUUCCAGUUGGACCAGCCAUUGAUUAAUGGUAUGUGAGGGUUAUUAGACACGACUGCCAAUUGUCUAGCAGUUGUUUGUUGUUUGUAAUCCACCGCUGACAUGACCACAGUUUCUAACAUCUCGAUUAUCGGAUCAGACGCUGAUGCGUAACUAUGUUUGUUGACUGGCAGUCCAGGUGAUAAAGACUUUACCGUGUUUCUCCACAAUUUGUGUGUUUGCUCCGGUAGGUAUAUCGGCUUGAGGUCAUUGCGCUUGGCAAGGACCGUCAGUUUUCCAGCUUUGUCUAAAAUGUAGCUAUGCACUCGCGAGUUGAUCCUCUCAUGUUCGUCAAGGGAAAGCAUGAGCCCUUCAAUUUCAAGUGUUCCUAUUGUCGUAUCACCGGUGUCUAACAGCGCACGCACCAGUGAAGAUAGUCUACCAGGGCUGGCACUGGCCAUCACGUGCUGAGGCAGUGUAUGUAUUGAAUACACGUUGGAGGCCAUGGCUUCCAGUUGUGGUGGUAUCACUGUGUUCCUAGUGACAAAUAACAAGAUUGAAUCGUUCACCAUCAUGGCGAUUUGUGGCACAGCGUUCAACAUAAAUCCGACUGUCACUGCAUCUGUCUCGUUUAAUUCAGUGUCGCUGGUACUCACUUCCAGAUUUAAACUCAUCAGAGUGUUUAUGAGAUGCAUUUGUUGAGCUGACUUCGUACGACUUGUCGGCAAUCCAGUUACUUGGGAUAUGGCAUUAGUCAGAUUGCCGAGUUUUGUAUCCAAAUAUCUGGCUAGUCUUAACAUUGUGGUCAGAUUUGCGGUAGCUGUAUUGUCGCUUGGCAUUGGUUGUAAUGUUAGUUGUGUGUCUGGCAUUUUGAAAUAUAGCUUUUCCCAUUCCAACCAUUCUAUAGCUCUUAGUAGGUCAGGGUUAUUCAGUUUUACGUUAGAUUUCAGGGUGACCACUCUAUCACAACAUUUCACACGGUUAAUGCUUAUGUGCACCUCUCCACUAAAAUAACCUGCACUUCGCUUCAGCAUGGUGGUGCCACACAUGAAACAGUGUCCUGAGUGGUUAACUGACCAGGUUUUGUUGUUCCACUCGAACGAUAGGGUUUGAGCUGCCAUUUGGUAGGUUCUAGACAACAUGUUCAGCCAGUCUAAUUUGUUCAUGGAAGGUGAUGUUAACUCAAGCCUGCCUUGCCACUUGGGUUUCUGCACUUCCAGGUUCUCAUAAUUGAUGACGCUGUAUGGCACUACGUCUUGUUGUUCAGUCACUUGAGACUGCAUGUCAAUCACAUGUUGUGUUGUUACUUUCCCUAGUGGAUCACUAAGUUUAGCAACUUUAAUGCUUCCGGUAGGGUCUUGCGAGACUAAGAAACUUGCCACCAGGAAUCCUAAUUUUUUAAUGACUAUAACAUCAUUGUCACCGUCUUCCUUCAGCGCCACGGUCACAUUGUACUUGUUUUCAUAUCUCGCUUUGGUUGUGUUGAACACUUGUCUGUUGAUUGGUCUUUUAACCACCUUAUGGUCCAUUUUUGCUUUGGAGAAGUAUUCAGCUAACUUAUUGAGAGGGUUCACCCACUGGAGACCAGCUCCGGAAAUGCUGCUGCCGAACCUUUCAGUAAGUGAAUGAGUUGGUUUGAAUAAGCCUACUGACACCCAUAUUAGGUGUUUCUUGGCUCUGGUUGCAGCUGACACACAGUACUUCCUGUUGACCUCUAUGCCAGCUUCACCACCUGUGCGGUGUUGUACUACUAACACACAGUCGGCUUCCUGAGAUUGAAACGUGUGCACAGUUGAAAUGGUGAGUUUUUGAAUUCCAGCUGCUUUGUGGUAAUGUUUGAGAGCAUUCUUGAACUUGAUGGCUGUUGCAUUGUGAAACGUCAACAACACAUCAACUUGUUGUUCAGCAGCCGUCUUGAUGAUGCCAUGUACGUCUAAGUCAGCCAAGCAGGCGGUAGUCACACUCGUGUCAUGGGUGGCUAACGUUUCAAGGUUGGGUAUGAUUUUCCCCAAUUCUUGGGCCAAUGGGUUGCCAUAUCGGCGGGUCUGUGUUUUCCUGUCGGUUUUUGGUAUGUGCUUGAAUAGGUUACUCCGUUCGCGUGUGCCUGGCAAUGUAUCAGUGUCGAUGACACCAAUCUGGUCUUCAUCUCCCAUAAUAAACAAGCCCUGUGGGUUAUGGCGCAUCAAUUGCAUCAGAGUGACAGGUUCAAUCAUGGUGGCCUCGUCAAUCCACAUCUUGUCUGCAGACAAGUCUUCUGUUUGCAGGGCUCUCUCUACUGACAUUACUCUAUCAAUUUUCUUGUUUUGCUUCUUUAGUUUGUCCCGAAUUGACUGCACUCCGUUAGUCGUCAUCGCUAGAGCCACAUCAUUCUCUCUCAUUUCUUGAGCUAGCAUGGUGGAUUUGCCCCAUCCUCCUGUAGCUAGCAGGCCUUCUGCUUUUGAUAACACCUCCAUUAACUUUACUGUGCGGUUUGGCGUCAUCAGCAGUGAAACUAUUGAACGCAAGGCACUGCCGGCUGACACUUUGCUGUAUCCUAUUGUCAUCUUAGCUGCUCCUGGUCUUUUGCUGACUCCCAAUGAUACCAUACCAUUCUCAACUGCUAUUUGAACAGGAGUUGGCUUGUCAUAACCUAUAGUUUUUGGUAGUAUCACGACAUCACCUGUCUUUAAUUUAGUGCUGCUGACAUCUAUGCAGGUCUUGUUGCCUUGCUUUGUCAGUAUCGGCACAUCCACCCACACUAAUUUGUCAUCUUGAUCCAAUAUAUGGUUGAAACAGCAAGCCUCCUUGUAUUGAUACACCUUGGCCCUAACAAUUUCCGGCAUUUGUGACCAUGUGAGGGAUGGAUCAUCGGAAUGGUCCAUUGGCUGCCUCAGCCAUGUGUUCUCGAUAGUCCUUGGGUCAUUGUUGAUUAACAAUUUGACACUUUGUUCAUAGCGGUCGUCUACUUCACAGUUAAACUUUGGCGUCGGGUGAGCUGUGAAUGGUAUUGUAGUGUGUAUGUCCUUGUCAACACAAUUGAAGCUUAAACGCUGUUUUCCGUCUAUUUUUGAGUAGCCGAGUUGCCAUAACCCUGAAUUAGCCCAUGAAGUGUUCAUUAUGCUUGAUUUAGCAACUAGAAGCUGGGCCCUCAAAUGUUGUUCCCUGGUCAGGAUGAACGGGUCAGCAUCUUGUAUUUCACCUUUGGUUACUCUCUUGACUGCUGUUCUCAGCACUGCUAAUGUGCAACUGUCUUGGAAGGCAAUUGGCCCAAGAUGUAGUUGUUGUUGCAAGCACUUGGCUGGGUACCAAUGAUCUAUUCCAACAGAUUUAAUCUUAUUCGAGUGCACUAGCACAGGGUACCAAUCGUCUAAUGACAUUUUUGUCACUCGGGUCGAGCUAUCUGUUAUGGUGAUAAGGUUGCUGGCAGUGUUCCGGGCUAUGUUGUGUAGAUCAGACUCACUAAACCAACCAUCUUUCUUAGCGAUUCUGGUCAUCACCUCUAGUGGUGGACUGUUGGGAUCGAGGUAAUGCCAACUAGUGUAGCCACAUAUGGCCCCUGGGUUGGAGUCUUCAAACACACUCAAUAUAGUGAACAUGUUUGUGCCCCCGCUUAGCUGGCCAAUUGGUACGAACGUGGCCCUGCUACGGAAUGGCUCAUCACCUGGUUGUAUUUUGUCAACAAACUCUACUGGUUCUUGACUGCCAGCUGUAGCCAACGUAAACAUGGCAAAUUGCAGGUUAGUGCGUUGUAUUAAUUCAUUAAGCAAAUCAUCUUCUUUGUUUGAGUCUGUCUGAGUGUUGAGGUUCGAAGGUGUUGACCUUGAAAUUUUGGGCGUGUUUUCCUGAUUUUUCCCUGUGUCAUCCUCUUUGAUCUCAAUGGAAUCGGGGUCAAGAGAAUUGUUGAUGGGGCUGGCCUUUGGUGCUUCUCUGGGAGGUGGUGUGAUCAGGAGGUUUUUCUUGUUACCCUCGCAACUUGGGCAGUCGCCUUUAAAUAAUGGGUGCAUGUCUGACUUGAAACUAUGAGCAUGUUGGUACACAGCACCGCAGUGGCACAAGUGUUGAUGUUCUUUGUCUCCAAUGACAGUACAUGGUUGUAAAGCGGUCCGUACUUUGACAAUGCGGUUGUCCGGUGUGACGUAGUCGCGAUAUGUGGCAUCAACGGGUCUGGGCCUUACCGGGGCUAAGUCUUUAGUGUCAUUGUUGCAAGUAGGUGUCACGCGUUGUUGUGUACGCUCGUGCGCAGCUGGUUCUGUUGACCAUUCCCACGUUUUGCCGAAGUCUAUUUGCUCUUCCUCUGGUACGUGUUCACAGCUGAUGCAGCGGUUGCUCUCGGUAUGUGUGGGCUUGCCACAGCAGUUGCAUUGUUCUCCUUGUUCAUCACCAUGUCCUAUGCAAACAUGAUCACAGUUGUGAAAGCAGGCCUCUGAACAACACGCGCACGGGUUGUUUUCUGAUUCCAAGCCGCAGCAAUCACAUGUUUCAUCGCCCGUGUGGACACAGUCAUUAUGCUCGUGGGUACAGUGUUCAACAGCACCCAGUUCGGUUGGGUGUAAAUUUUUAAUCUUAAUUUGGUCUAGAGAAUAUUUUUCCGUCCAAUCCUCUAAUUCGUCCCAAACUGGGUGGUUAGCGUAUGUUGUGAUGACGGACAUGCUGUUUUCGUCCAGUGAGUUAAGCACUGAAUUGAUUGUGGAUGAGCUGUCACCUAGCAUUAACAUCAAGCUUUCCACUGCUGCUUGAGCUGUUGCUAGAGCCAUUCUGCUAAAGUGUGCUAGAGCUGGCGACUGAGUGCUCAACUUGGCAAUCAAGCUUCUUUUUGCGUGUGCUCUUUUAGUCAACGCAUAUGCCACACUCGCAUGUGUAGCAGCAUCAUCUGCUGUGAUAUGGUAAUUGUUGUACGUGGUGUCAUCUAUAGAAUAUCGUGCAUGGGCCAUGGCCAGACCGUAGUCUAACAUGCCUUGUAAACCACUGGCACCUGACAGAUUCCUUGUCAUCAUUGUUCUAAUCACGUCUAAUUUUAGUUCUGUCUUGGUGACAGUCAACAAAGGAAUACCUAUAGCGCCCAACACUGUGCUGUAUGUCAGUCGCGGGUAGUUGAAGGUGUUAACUUUUGCCACUUUGGCGAAAUAAUAUUUUUCUAUAGCAUUCUGUUCCAACCUGAUUGCUGAUAUGGUCACCACUGUGACAACGGCAUCGAGGUCUUUAGUUGUGCUCAUGUAAUUGUGAGAACCGUCGCUGUAGACGGUGUGGUCCAUCAAUUCUGAUAGUGUGUCUCUCUCCACUCUAGAUACGGUGUCAUCUGAAUCGAGUGUGUAUAAGUAACUAUUUCCAUUGAUUGACCAUUCACCUCUCUGCCCAGACAAAGUGCCACUUACUCCAGUAGUGUCAUCUCUUUUUGGUAGUAUAAUCGUGACUAGCUGUGGUCUUGUGCUGAGUGCUUCAUACAAUGUGUGUUGUGUAAUGUUUAUUGAUGUGCCAUCAAUUAUCAGGUGGUUUAGUCCGCCUGGUGCGUCUGCCAUGGAGGUGGAUUGCAGCCAACCGUCGAUUGGCUCACCCCAUGACCUUGUGCUAAGAAUGCUUGUCGUGCCAUGUGGUGUAUUGUGAAAUGCUUGCACGGCAGUCAUUAAAGUGUAGCCCAAAACAGUGGACUGCUGCAUCACUGGUCCCGGGCCAGUUAUGAGAUGGUACGAAGGGAAUGCUACUUCCAAGCUAGCCAUUGUCUGUUCCGACACAUUGAUGGAAGUUCUCACCAUGGUGACUUCAAACCGAUUAAAGAGACCUCGGACGACCCUCAUGUCAUGGUUUUCAGCUAUUUGUCUGUAUGUGUCAGCUCUUUUUAGGUCUAUUCCGUCCAAGUAACCAGAUAGUCCAAGGCGAUCCAGCUGUGAUCCGAGGUGCACUCUGUCUAGCAUGUUAUUGAUUGGCGUGAAUAUCCACUCAUUGAGUGACGUUAUAAUCUCUCCUGUAAGUUCUGUUAAACACAUUGGACAAAGAAAACCUUCAACGAGUACAUCCGGCUUAGCCAAUUGUUUAGCGAUGAUCGAUUGUACUUCUUGCUGGUCCAUUUCUAACAACACGUCAAUAGCUUCUUCAUCUGAGCCAGCUUCAUUGUUUGGUAAUGGUAUUGUGACUUGCACGGGUACUUGUACAACCAAACCACAGCAAUGACAUCUUACAGCUGACACAGCACCUGUUUCUGGUUUAUAUUGAUUAGGUACCCACAAGUUGAACGCUAUGGCUAACCCAUCUUUGUAAACUAUUUCAUGCAUGGCAUCUUUCCAAAUGAAUCUGUAGUUGACGAAAAAUCUAGAACUUGCUAUGCUCUUGUGCAGAAAUUCCCUCCAGCCAUGUUUAUGUGCCAUGGCUUUCAACCAUGGGGUGAAUUCACGGAUGUGAUCUCUGGCGGGUUCAUCUUCAUCUUGAUAGUUCUCAUUAGUGAUGUCGGGAUUUAACUCUGACACACACAGCUUCACCAUUUCCUCAAACAUGUCGUGGCCGAAUUCUACUCUACCUGGUUCUUGUUCGUCGUCUGAGUCGUCUGGUUCCUCUGGGAUUGUAGCACUAGCAUAAUUUUGAGGUAGUCCAUCGAAGGUUGGCCCUUCGAUGUUUUCGAACUGUUUUCGUGGCAAAGCUAUUGGUAGCAGUGCAUUUGGUACUAUUGGGCCCUCAUCAGUGAGAUGGGACUGGCAGUAAACUUUUUCCCCGUUUUCAUCCUCAUAGUACGGAGUAAACUCUCCAAGGUUCUCUAUAUAGGAGUAUGUGUUAUACUCUUCUUCAUCUUCUUCAUCUUCUAUGGGUAUAGGAGGGAGCAUCAGAUGGGGAUAUUUCCGUUUAAUUUCUCUGUGCCGGAUUGUCCGGCGAUGUGAAACAACGGUGACCUUGGUUUGUUUGUUUUUAAAUUGUAAUUUUUGGGUGGGCCCGAGGACAGGCCCACCCAAGUUUUGAAGAUUGUUAUUUUUAUUUUUAAGUAAAUUAAUGAAAUUAGCCAAGGAGAUCGG  >Ss-AA_clean.1_(paired)_contig_6278  CCUCGGGUGGCAGCAGAGAUGCAGUAUUUCCGGUUUACUUCAAUACCAGCUUCACCACUCACUCUAUGUUGGACAACCAACACGUUAUCAGCCUCUUGUGACUGGAAUGUGUGUACAGUGGACACGAUAAGUUUGCGUAUUGCGGUGGUGUGGUGGUACUGUUUGAGGGCAUUCUUGAAUUUGUUAGCUGUGGCAUUGUGGAACGUCAGCAGGACGUGGACGUUGUGCUCAGCGGCCAUUUUUAUGAUGCCAUUGACGUCAAGCUCUUGUAUUGCUGUGGUUGUCACGUCCGUAGUCCUGUCAGACAAUGUUUUGAGACCAGGUAUGACCUUGGCCAGCUCAAGUGCAAGUGGGUUGCCGUAUCUCCUGGUCUCGGUCUUGGACUCUACGUCUUUAAUAUGUUUGAACAAAUUGCUGCGCAUCCUAGUGCCAGCUAGGGUAUCCGUGUCUAUAACUCCUAUUUGAUCUUCAUCACCCAUGAUGAAUAUGUUGUCUGGUUCAUGCUUCAUUAAUUGCAUCAACGUGACGGGCUCGAUCAUGGUGGCUUCAUCCACCCACAUAGUGCUAACGUGCAAGUCGUCAGUUUGCAAGGCUCUCUCAACUGACAUGACGCGAUC  >Ss-AA_clean.1_(paired)_contig_15215  GUUGUCUUGGUGUGGGCUUGGGCAAUCGCUCAGGCUGUUUUGGUCUACUCGUUUUGAUUGGCUCACCGGAAUCAGUUGGCCAAACCCAGGUCUUGCCGUAGUCGAUUGUCUCGUCGGGGACUGGCUCAUCGCAAGACAAGCAUGUAGCGUUUUCAGAAUCCAUCUUGACACCACAGCAGUUGCAUACUUCACCUUCUUGAUUUCCGUGACCAACACAUGAGUGUUCACAAGCGUGCUGGCAGGGUGUUUCGCAACAAGGACACAUCUCCGAGCUAGCUGGUUUACCACAACACUCACAUUCC  >Ss-AA_clean.1_(paired)_contig_14516  GUAUAAUAUUUAGAAUGUCUGCCACAUCACAAUUGUUAUGUAACGCAUUUGCCUCUAGCCUGUCUUCCAUAGUUGUCCCUAACGGGACUGUACCAUUGAAUCCCAGCUCAAGGAGUCGCUUGCUAGUUAAAUAAUGGUUUCCAAUCAUUAAUAGGUACGAUGCGGUCUUCGACUUGAACUCGUCACUAUCUGCGUUGUAUGGUGAAUAUGCUAACUUAUCCUUUAGCCUCAUGAAGUUGUGUACGGCUACGUACCUACCGUUGACCCACAUCACAAUGAGUUGAAGGAAGACUCCUGAAUCUUCUGACGUGGUGAAAGUACAUUUAACAUUGUGGUUAUUGGCUGCAACUAUUGCUACAUAUUUACCGUCGAAAUCAUCGUCCGUGAGUAUCAGGCUAUCAUCACCCAGUGUGUAGACAUGGUUCACAGCUUUGCCUUUGCAUAUGUCAGAUAAGAGUAACAAGUUACGCAUCUCAUUCCCUAGUGAUGUCAUAGCGCCACCAGACAAGUGCAUUGUUGGCAUGAAACCUGUCACAUUAUCUGGUGUUUUU  >Ss-AA_clean.1_(paired)_contig_192  GUGCAAUAGCACAUUACUAGAAUUACUCAUUUCUAGCAACCGGAGUCUGUAGGAUUUCAAGCUUGUCACAUAAAUCUGACAAGUUCGAGAUAUAGUAGGAUUCUCAUGGUUUAAGAUUGGUGAUUUCGAAGUAGCGUUCACCAUCAUCAUCCAUUAAUGCAACAUUGGUGGGAUCUAACUCACCAAACAUUAAUGUCAUGUUGAUAGUGAAUUCAAGCUUCUUGAAACCACGCCUUCAAAGCAUAUCUAUAUGACUUAUAGAUUGUGAAGAAUUUCUAAGAUUUGACAUCAGUUUAUCUGGUUUAUCAAGUCUCAUGAGAGACAUCGCAUCUAUAAGGUCCAUCUCAUCCAUGUCACGUACCCUCAUAAGGGUCCGCUUCAUGUCUAAGAUUUUAUUAUAGACUGCUAAGACCAUGGGAUGCCGCUUAAGAAGCAUCAAGUCGAAAUCUCCUUUUCGGAAUUCCGAUUCGAACUUAUCGUAGAAACAAAUAAGAUCGUAUGAUGCCUCUACAGUAGAACUUACUAAACCUAGACACAAGAUCUCACGGAUAAAACCGUGAAUUAGAUCUCCCUUUGGUAUUAUGAUUUCUCAGAACCUUAUUUUCCUUGAAAGGUACAAACGUAUCUGUUCAUGGGUAUUAAGGUUGAGAGAUUGUCUAAUAACAAAGACAAUAUCUUCAACAGACGACCUAAGAUUGGAUCGAGAUAUAAAUCUCUUUCUAAUCUUUACGUCUUUGAAGACAUUUAAGAUCAAUUCAGUAGCACUACCUUUCCAGAAGGUUGUAUUAGAAUAUAAGUAUAGAACAAGUUGUUUUACAACAACAGGUAAUACAUUUAUAUUAGAUACAAUCCCUCUUAAAGGUAGUCCUGAGACCUCAAUGCCCUUCCUUACUCAUCUUUUAGCAAAUUCAUAUGUAUUUUUCGAUACAUGAGUCUUUGCAACUGAGAUGUCCACACCAAGCUUGUUCAUAAUAAAUAUAUACUUUCUGGCAACGGCAUCAUGAUUAAUCACAAUGUCAUCACCAAGAAGAAUAUAUCUAUUAAAGUCCACUAUUCCGCACAGACGUGCAGCAUAGUGUACUACAAGAUGAUGUGUUAGAGUAAAAGUAGUCCAGGAACUAUAAGCCCCCAUCGGUUGUCCAACACUGUAACGUGUUCAACCACCGGAGGGAAGUUUAUAAUCCCUUUCAACUAAAAUAUUCCUCCAAGCACGUGCUUUUGCGCCAUCUCCUAGGAGAUGGGCUAUCACACGCUCUUGUAGUGAUAUUGGGAAACGGUCAGUGGCACUGGUUAGAUCAAGUGAUCAAAACUUAUGCCCCAUGGUCUUACCCCAGUUAUUGAAGGGAUCCUGAGUAAAAGUCCUAUCACAUGGUAGUGUUCUCAAUUUCUUGAGAAUACCAUCAUGUAUAGGUUUAAGAACCAAUUGACUAUAGUAAUCUACCAUAGCUAUUGGUCUCAUUUUCAACUCUGGAUCUUCAACUACUGAUAUCUUACCUAUACCAUUUGUCGAGUUACCAAUAUGGAAUGCUCGGUGAUCCUUGAAUACAACUUCAAGAAACUCACCAAUCAUAACUUUGAAAGAUCCCUCGCCAAUCAAUGAUUGGAAGUGAUCUAACAUUGCUAGAUUUCAGUGUCCCAUAUGGAACAAGGCAAAUGGACCAGUUAGUGUUGCUUUUCCCAUUGGUGAGGACUUAUUACUUACAUAAUGUAAGGAAUUAUCCCACUUAGGAGAGAAGCUUAGAUUAUAUUUACUAAUGAAAUCUUUAAUGAAUUCAUCGGGUAUUUCGAAGCUCUUAUCCCCUUUAAAAGGGUCUGAGAUCGAACUAUAAUCUGGCACUACUUGGUUCUUUUCCUUCAUUGUAGGGAUUAUAGAUCUUGUGUAACCCAAGAGAGUAAGAACAAAUCUUAUCAUCUUGGUGUCACCACUAUCUAUCAAUUCCUUCAAGUAAAGGAACUUUGUAGGAAAACCAUCAGUAAGUGAAACCAAGUUGGAAUUAGAUCGUAGUGGUUGACCACUUAUGUAUCUUGUAAUAUGAAGACGAGCUGUCUUCAUAUACUUGAUAGUAAAUGGUAAACCAGAUUGCUUCCUCAUUGAUUCCACUUGCCCAAUAUACCUCUUACCUAAUAUCCGGUUGUGAAAUAGUAAGUCUAAUAACCUUAAAAUUAUUAACUUGCUAGUUUUCAUGAUUGUAUAUUAAAAUAAGAGCACCUAUAGUGUCCAGUUGUUAAACAUAGCUGGAUCCCGACUUUACCUACGGGUGAGUCACUAUAGGUUCCUCCGCAGGUGGCCGGCGUCAUAGAUGCUAAAUCUAUGAUCGGUGCCCGCCAACGGUGAAGCAAGUUACCCAGGAAUACAGUUUAUUAAACUGUAACCCAGGCUACAAACUACUCAGUAAUCCAAGGCUUUGAGUCCUCGGCUGAGUUGAUCGCAGGAUACACACAAAGUGUGUAUGGCUCUAUUGAGCCGCUUUUGGGGACCGAAAGGUCUCCAAG  >Ss-AA_clean.1_(paired)_contig_187  UUACUAUCAAGUAUAUGAAGACAGUACGUCUUCAUAUUACAAGAUACAUAAGUGGUCAAACGCUACGAUCCAAUUCCAAUCUGGUUUCACUAACUAAUGGUUUCCCUACAAAGUUCCUUUAUCUGAAAGAACUAAUAGAUAGUGGUGACACUAAGAUGAUAAGAUUUGUUCUUACUCUCUUGGGUUACACACGAUCUAUCAUUCCAACAGUUAAAGAGAAGAACCAGGUAGUUAUAGAUUAUAGCUCCAUUUCUAACCCUUUUAAGGGUGAUCUGGACUUUAACAUACCUGAUAAAUUCAUCUCUGAAUUUAUUGGUAAGUAUAAUCUCGGUUUUACUCCUAAGUGGGAUAAUUCCUUACAUUACGUAAGCAAUAAAUCAUCACCUAUGGGUAAAGCCACACUAACUGGUCCAUUUGCAUUGUUCCAUAUGGGGCACUGAAAUCUAACAAUGUUGGACCAUUUCCAAUCUCUGAUUGGAGAUGGACCCUUCAAAGUUAUGAUUGGUGAGUUUCUUGAAAUAGUAUUCAAGGAUCACCGUGCCUUCCAUAUUGGGACAUCUACAAAUGGUAUAGGUAAGAUAUCAGUAGUUGAAGAUCCUGAGUUGAAAAUGAGACCGAUAGCUAUGGUUGAUUACUAUAGUCAAUUGGUUCUUAGGCCAAUCCAUGAUGGAAUUCUUAAGAAAUUAAGAAAUCUACCACAGGAUAGGACUUUUACUCAGAAUCCUUUCAAUAACUGGGGUAAGACCCAUGGCCACAAGUUUUGAUCUUUAGAUCUAACUAGUGCCACUGAUCGUUUCCCAAUAUCACUUCAAGAGCGUGUAAUCGCACAUCUAUUAAAAGAUGAGGUGAAAGCACGUGCAUGAAGGGAUAUUUUAGUUGAAAGGGAUUAUAAACUUCCAAAUGGAGGUUGAACACGUUACAGUGUUGGACAACCAAUGGGAGCUUAUAGUUCUUGGACAACUUUUACUCUAACACAUCAUCUUGUUGUUCACUAUGCUGCACUUAAGUGUGGUAUAGUGGAUUUUAAUAAGUAUAUACUUUUAGGUGAUGACAUUGUGAUUAAUCACGAUGCAGUCGCUAGAAAAUACAUAUCUAUUAUGAACAAGCUUGGUGUGGACAUCUCUGUUGCAAAGACACAUGUGUCAAAGAACACAUAUGAGUUUGCUAAGAGAUGAGUAAGGAAAGGCAUUGAGGUCUCAGGCCUACCAUUAAGAGGUAUAGUAUCAAAUAUAAAUGUGUUACCAGUUGUUGUGAAACAACUUGUAAUGUAUCUAUAUUCUAAUGCUACACUUUGAAAUGGUAGUACUACUGAGUUGAUCUUAAAUGUCUUCAAGGAUGUUAAGAUUAGAAAGAGAUAUUUAUCACAAUCUAAGCUUAGAUCAUCCGUUGAAGAUAUUGUCUUUGUUAUUAGACAAUCUCUCAGUCUAAAUUCCUAUGAAGAGAUACGGUUGUAUCUUUCUAAGAAGAUUAAGAUCUGAGAGGUGUUAAUUCCAAAGAGAGAUCUAAUUCACGGUUUUAUCCGUGAGAUCUUGUGUCUAGGAUUAGUAAGUUCUGCUGAAGAUGCAUCGUUCGAUCUUACUCGUUUUUACGAGAAGUUCGAAUCGGAAUUCCGUAGAGGAGAUUUCGAUUUGAUGCUACUAAAGCGGCAUCCCAUGGUCUUAGCUGUCUAUAAUAAAAUCUUAGACAUGAAACGGACCCUUAUGAGGGUUCGUAACAUGGAUGAGAUGGACCUUAUAGACGCGAUGUCUCUCAUGAGACUUGAUAAACCAGAUAAACUGAUGUCAAAUCUUAGAAAUUCUUCAACGUCUAUAAGUCAUAUAGACAUGCUUUGAAGGCGUGGUUUCAAGAAACUUGAAUUCACUACCAACAUGACAUUAAUGAUUGGUGAGUUUGAUCCCACCAAUGUGUCAUUAAUGGAUGAUGGUAAUGAAGACUCUUUCGAGAUAACUAAUGUUGGUUAUCCCUACUAUGAGAUACCCAACCUUAAACCUUGGGAAUCCUACUAUAUCUCAAACUUGUCAGAUUUAUGUGACAAGCUUGCAAUCCUACAGACUCCGGUUGCUAGAAAUGAGUAAUUCUAGUAAUG  >FirsU_Contig50  UAAAGGAUGCAGAAAAAGCCCGAGCUUGGCGUAAUAUAUUGGUUGAAAGGGAUUAUAAGCNUACCAUCUGGAGGUUUCACACGAUAUAGUGUUGGACAACCGAUGGGAGCAUAUAGUUCUUNGGACAACCUUUACUCUAACACACCAUCUUGUAGUCCACUAUGCCGCUUAUUUAAGUGGUANUAAUAGACUUUGAUAGAUAUAUACUACUUGGUGAUGAUAUUGUGAUUAAUCACGAUAAAGNUCGCUAGACAGUACAUACGAAUCAUGAACAAGUUAGGUGUUGACAUCUCCACCGCGAAGANCACAUGUAUCUAAAAAUACUUACGAAUUCGCCAAGAGAUGGGUAAGGAAGGGCAUUGAGANUUUCGGGACUUCCCCUAAGGGGUANUAGUGUCUAAUGUAAAUGUGUUACCUGUUGUUGUUAAGCAACUUGUGACAUACUUAUAUUNCUAACACCACACUCUGGAAGGGAAGCUCCACUGAGCUGAUCUUAAAUGUUUUCAAAGGUGNUCAAGAUUAGGAAGAGAUUUGUUUCUCAACGUAAUCUUCGUAACACCGUUGAAGAUAUUGNUCUUUGUAAUAAGGCAAUCACUCUCUCUCAAUUCUUAUCAGGAGAUACGUCAGUAUCUCUNGUAAGAAGAUUAAGAUUGAGGAAUUGAUUAUUCCAAAUUCAGAUCUAGUUCACGGUUUUANUCCGUGAGAUCUUGUGUCUAGGUUUAGUAGCUUCCGCUGAAGAUGCGUCAUUCGAACUUUNCUCGUUUUUACGAUAAGUUUGAGUCAAAUUUCCGUAAAGGAGAUUUUGACUUGACACUUCNUAAAGCGUCAUCCCAUGGUCAUUGCUGUCUAUAAUAAAAUUUCAAACAUGUACCGAACCCNUUUUAAAGGUAAGGAACAGGGAUGAAAUGGAUCUUAUAGAUGCGAUGUCUCUCAUGAGACNUAGACCAACCAGAUAAACUGGUAUCUAAUCUUAGAAAUUCCUCAACAUCUAUAACUCAUANUAGAUUUGCUUUGAAGGCGUGGUUUCAAGAAGCUUGAAUUCACGACUAACAUGACAUUAANUGAUUGGUGAGUUUGAUCCCACCAAUGUUGCAUUAAUGGAUGAUAGUAGUGAAAACUACUNUCGAGGUAACCUCGCUUAAGCCAUGAGAAUCCUACUAUAUCUCUAACUUGUCAGAUUUAUNGUGACAAGCUUGAAAUCCUAAAGACUCCGGUUACUACAAAUAUGUAUUUCUAGUAAUGUG  >Ss-AA_clean.1_(paired)_contig_430  UUAUCGUGGGCCUAAUAGACCGAGUGUAACCUAACAAUGUUAAUACGAAUCUUAACAUUUUAGGGUCACCCCCAUCUACCAAAGGCUUAAGAUAAAGAAACUUUGUAGGAAAGCCAUCAGUUAGUGAAACCAUCGAAGAGUUGGAUCUUAGCGGUUGACCACUUAUAUAUCUUGUAAUGUGAAGACGAACUGUCUUCAUAUACUUGAUAGUAAA  >Ss-AA_clean.1_(paired)_contig_3529  UCGUGGGCCUAAUAGACCGAGUAUAACCUAACAAUGUUAAUACAAAUCUUAACAUUUUAGGAUCACCUCCAUCUACUAAAGGCUUAAGAUAAAGAAACUUUGUAGGAAAGCCAUCAGUUAGUGAAACCAUCGAAGAGUUGGAUCUUAGUGGUUGACCACUUAAAUAUCUUGUGACAUGGAGACGAACCGUUUUCAUGUACUUGAUAGUAAAAGGUAAAC  >Ss-AA_clean.1_(paired)_contig_429  GAGAUUAUACUUCUUAAUGAAUUCACAGAUGAAUUCAUCGGGAAUUUUAAAACCCUCUCUACCUUUAAAAGGGGCAGAGAUUGAAGAAUAAUCUGGAGACACCUGGUUCCUUUCCUUUAUCGUUGGCCUAAUAGACCGGGUGUAACCCAACAAUGUUAAUACGAAUCUUAACAUUUUGGGAUCACCCCCGUCUACUAAUGGCUUAAGAUAAAGAAACUUUGUAGGAAAGCCAUCAGUUAGUGAAACCAUCGAAGAGUUGGAUCUUAGUGGUUGUCCACUUAUGUAUCUUGUAACGUGAAGACGGACCGUCUUCAUAUACUUGAUAGUAAAAGGUAAAC  >Ss-AA_clean.1_(paired)_contig_641  GAGGUUUCACACGUGACAGUGUUGGACAACCGAUGCGAGCAUAUAGUUCUUGGACAACCUGUACUCUUACACACCAUCUUGUUGUCCACUAUGCCGCUUAUUUAAGUGGUAUAGUAGACUUUGACAGAUAUAUACUGCUUGGUGAUGAUAUUGUGAUUAAUCACGAUAAAGUUGCCAGACAGUACAUACGGAUCAUGAACAAGUUGGGUGUUGACAUCUCUGACGCGAAGACACAUGUAUCUAAAAAUACCUAUGAAUUCGCUAAGAGAUGGGUAAGGAAGGGCA  >Ss-AA_clean.1_(paired)_contig_624  CAGGUAACACAUUUACAUUAGACACUAUACCCCUUAGGGGAAGUCCCGAAAUCUCAAUGCCCUUCCGUACCCAUCUCUUGGCGAAUUCAUAAGUAUUUUUAGAUACAUGUGUUUUCGCGACAGAGAUGUCAACACCCAACUUGUUCAUGAUUCGUAUGUACUGCCUGGCGACUUUAUCGUGAUUAAUCACAAUAUCAUCACCAAGUAGUAUAUAUCUAUCAAAGUCUACUAUACCACUUAAAUAAGCAGCAUAAUGGACUACAAGAUGGUGUGUAA  >Ss-AA_clean.1_(paired)_contig_198  GUGCAAUAGCACAUUACUAGAAAUACUCAUUUCUAGCAACCGGAGUCUUUAGGAUUUCAAGCUUGUCACAUAAAUCUGACAAGUUCGAGAUAUAGUAGGAUUCUCAUGGUUUAAGAUUGGUGAUUUCGAAGUAGUCUUCACCAUCAUCAUCCAUUAAUGCAACAUUGGUGGGAUCAAACUCACCAAUCAUUAAUGUCAUGUUGGUAGUGAAUUCAAGCUUCAUGAAACCACGCCUUCAAAGUAUAUCUAUAUGACUUAUAGAUUGUGAAGAAUUUCUAAGAUUUGACAUCAG  >Ss-AA_clean.1_(paired)_contig_67  CCCCAGAAGCUGUGCAGAAGCACAUUACUAGAAAUACAUAUUUGUAGUAACCGGAGUCUUAAGGAUUUCUAGUUUAUCAGAAAGAUCUGAUAAAUUAGAGAUAUAGUAGGAUUCUCAUGGCUUAAGGGCGGUUAUUUCGAAGUAACCUUCAUCAUCGUUAGACAUUAAUGCCACAUUGGUGGGAUCAAACUCACCAAUCAUUAAUGUCAUCUCGGUGGUGAAGUGUAACUUCUUAAAACCACGCCUUCAAAGCAUGUCUAUAUGGGCUAUAGACGUUGAAGAGUUUCUUAGAUUUGACAUCAGUUUAUCUGGUUUGUCAAGUCUCAUAAGAGACAUCGCGUCUAUUAAGUCCAUCUCGUCCAUGUUACGGACCUUCAUCAAGGUACGUUUCAUGUCCAAGAUUUUAUUAUAGACGGCAAGAACCAUGGGAUGUCUAUGGAGAAGCUUCAGAUCGAAAUCUCCUUUACGGAAUUCCGAUUCGAACUUAUCGUAGAAACGAGUAAGAUCGAACGAAGCAUCUUCAGCAGACUCUACUAGACCAAGACACAGGAUCUCACGGAUAAAACCGUGAACUAGAUCUCCCUUUGGAAUAAUCAAUUCCUCAAUCUUAAUCUUCUGACAGAGGUACCUUCGUAUCUCUUCAUAAGAGUUUAGAGAGAGUGAUUGCCUUAUAACAAAGACAAUAUCUUCUACCUGGGAUAACAGAUUGGAACGAGAUAGAAAUCUCUUCCUAAUCUUAAUCCCUUUGAAGACAUUUAAGAUCAGCUCAGUAGUACUACCACCUCAGUGUGUUGCGUUCGAAUAUAGAUAAUUCACAAGUUGUUUUACAACAACCGGUAAAUUGUUAAUAUUAGAAACAAUACCCCUUAGUGGUAGACCAGAGAUCUCAACGCCUUUCCUUACUCAUCUCUUCGCGAAUUCAUAUGUGUUCUUUGACACAUGCGUCUUUGCAACAGAGAUGUCCACACCAAGUUUGGUCAUAAUACGUAUAUAUGCCCGGGCGACUUUAUCAUGAUUAAUCACGAUAUCGUCACCUAGCAAUAUAUACCUAUUAAAGUCUUGUAUACCACACAGAUGUGCGGCAUAUUGGACGACCAUAUGGUGUGUUAGAGUAAAAGUUGUCCAAGAACUAUAAGCCCCCAUCGGUUGACCAACACUAUAACGUGUAAAUCCACCGGUAGGAAGUUUAUAGUCCCUAUCAACUAAUAUAUCCCUUCAGGCUCUGGCCUUAACCGGGUCUUUUAAAAGACCGGCUAUCAGCCUCUCCUGUAGAGAUAUAGGGAAACGAUCUGUAGCACUAGUUAGAUCGAGUGAUCAAAACUUAUGCCCCAUGGUCUUACCCCAGUUGUUGAAGGGAUUCUGAGUGAAAGUCCUAUCUUGAGGGAGGUUCCUUAAUUUCUUAAGGAUUCCAUCAUGGAUGGGACGAAGAACCAAUUGACUAUAGUAAUCAACCAUAGCUAUUGGUCUCAUCUUCAACUCAGGGUCUUCAAUUACUGAUAUCUUUCCAAUACCAUUUGUGCUUAUACCAGUAUGGAACGCUCGGUGAUCCUUGAAUACUACUUCAAGGAACUCACCAAUCAUAACUUUGAAAGGGCCUUCUCCAAUAAGCGAUUGGAAAUGGUCUAACAUAGUUAGAUUUCAGUGUCCCAUAUGGAAUAAGGCAAAUGGACCAGUAAGUGUGGCCUUUCCCAACGGUGAGGACUUAUUACUUACAUAAUGUAAGGAAUUGUCCCAUCGUGGAGUGAAGGACAGAUUAUACUUCUUGAUAAAUUCAUCUAUGAACUUAUCGGGUAUUGUAUACCCUAUCUUACCCUUGAAAGGGUUCGAUAUUGAACUAUAGUCUACCACUACCUGGUUCUUCUCCUUUACUGUAGGCCUGAUUGAUCUAGUAAAGCCUAAGAGAGUUAAGACAAAUCUCAACUCCUUUGGAUUACCAGAGUCUAUCAGGCGCUUAAGGUAAAGGAACUUUGUUGGAAAACCAUUAGUAAGCGAAACCCGUGAAGAGUUAGAUCGUAGCGGUGUACCACUUAUGUAUCUUGUAAUAUGAAGACGAACCGUUUUCAUAUACUUGAUAGUAAAUGGUAGACCUGAUUGCCUCCUCAUUGAUUCCACUUGUCCAAUAUAUCUCUUCCCUAAUAACCGAUUGUGAAAUAGUAAGUCUAAUAACCUUAAAAUUAUUAACUUGCUAGUUUUCAUGAUUGUUUAUUAUCGAAGAGCACCUAUAGUGUCCAGUUGUAAACAUAGCUGGAAGCCCGACCAAUUACGGGAGGGUCACUAUAGGUUCACCCGCAGGUGGCCGGCGUCAUACACACUAAGUGCAUGAUCGGUGCCCGCCAACGGUGAAGCAAGUCACCAGAAUCCAUGCUUAUUAUGCAUGGACUUGGUUAACAACCUACUCAGGAAUCCAGUUGAAACUGGCUGAGUUGAUCGCAGGAUAUAAGCGUAAAACACUUAUAUGGUUCUAUUGAACCGCUUCU  >FirsU_Contig5  ACCUUAUUUAAGCUUAUCAAGUCAUUGGCUUCAUCGAAUUCAGUACGGAAAUCUAAAUAGNAGUUCCUUAUUUGAAUUAAUGGAGUCAAUGAUUGGUAUAAACUUUCUAUAGUCAACCGUUNACCCGGCCUCGCCAUGGAAGGAACAUUAUUUGUGACAGCAUGCUCUGUACGGGUCGUAAGNAACCGCAUAGACAUGUCUGCCAACUUAUCUAUCUCCCUAUCCUUCAUGUACAAGACCGCCNCUUUUAUAGGCGUCAAAUACCUGAGGGAUGGCCAGUGAACCGAACCCUGCAGCCUCAGCANAACCGUAAAAGGUCAGACUGAGCUACCAGGUAAGGUUUAUUGGACAUCAGGAGAUAAGAUNAGAAGUUUGGAAGAGUGGACCAGGCUAUUAACUAGUCUGGGUUUAUCCUCCUUUCCAAUCNUUCAAUAAGUUCUCUCAGAACCGAGGAGUGUUUCCGGAGACGAAAAGAGGUAGGUUACCCNCGCUCAACAACGGUGGUUGACAGCGUAGCAACUAAUCAAACAUAGUUGUUUAUGUCUGCANAUCAGCGCAGUGAUAGGGAAACUUGAUAUCUCAACGGACUGUAGACCGAANUCUUUUGGCGAACUCAAAUAAAAUUUCACUUACGUGAGUUUUAACCGAGGAGAUUUCGACNUCCAAGUUGAGACAUUACCUCCAAGUAUUUCGCCGCUAGGGCGUGGUCACAUAUAACUAUNGUCAUCCCCCAGAAUCUUGUACCUAGGAUUGUCAAUGUUUAAAACCAUUGCACAAUACUGNGACUACAAAAUGAUGGGAUAGGGUGAACAUAGCUCAUGAUGACUUUGCCCCUAGAGGUUGNACCACACGCGAACUUAACCGUGGGGCCACCAGGCGUGUCGAACGGAAGAUCGAUCAUAGCNACGUACUCAGGCGGUCGCAUAUUGCUCACUUGUUAACCAAGCCAGGACCAUUUCCUGUACNGAAUAUAGGAAAUCUGUCCGUGGCGGAGGUUAGAUCAAAUGAAUAAUAUGGACCACCACCNGUACAACAAGUCCUUGAACCCUUUGUUUUGGUCGAAAGUACAAUCCUGAGGUAUGGACUUNCAACACACGAUAUAGUCUAUCGUGCAAUGGAGUCAAUGCGCUCUGGGUUAUAUAAUCGACNUAUUGCAAAAGGUCGGGACUUGUACUCCUUAUCCUUCUUAAUGGAAAUCCUACGAAGAAGNGCUACCAGUUUUAUCUGGAUCCAGCUUUGUAAGUUUUCAUAGAAUACCAUUAGUGGUUUCNUACGAAUCCUUUCAAGCUGGAAACCUUCUCCUUAAACUCAGGGCCACCAAGAGUGUAAAGNACACUCCAGGAUGUCCUGAGGGAUAUGCAUUAAGUCAGAGAAAGAGGUGGCUAUGGAGAUNCCCAUUCGGCCCGCUAGUGGACACCCACGGAAAAGUGAACGAUGUGGGCUCAUCUAGACGNGCUUAUCCCCAUACGCUUCAUUACAAUAGCAACACUCGCCCCCGUGAACGGGUUUGACCCNCGAACACGGACGAGUAAUACUAUCGUAGGAAGCGAUGACCCCUGUUCCUAAAAUCAGUCUNCGAUAGUUGUAAAACUGUCAAGACGUAAGAGAUUAGGAGGAAGUCUUUUCGCAAGAUUCCNGAGUCUAACCUGUAAUGGUAACAUCAUAGGUAGAUUCGUUUUCUUACAUAAAGAGAUUCCNAAAGGGUCGUGGAAGCUCCUGGCCAGCAAGGGUACGGGUGAUGGCUAACCGUGACAGCUUNAAACGCUGUGGCGGUCCACUUUCACCCCUUGGUCUUCCCUCACAGGAAGAACAAGUGUAUNUGCCUGUCGGGUAAACGCGGCGAGAUUCGGAGAAGUUCCUUUGUAAUAGUACUUACGGACNUCACUUCAUUAGUUUUAAAUCUAGUGGUUGAGUCAUAAGACUACCAAN  >Ss-AA_clean.1_(paired)_contig_2607  AUGGUUUAGCUGAAUAGCCAACUAGCGUGAUUACCUACCGGAGAGAAACCUAUUCAGCUCUUAAUACUGAACCGAAAGGUCACUGUAUAAGCGGUUUAGAUUAAGAGGAUGUUCAAUUGAUUAGUCUUAUGACUCACUCACUAGUUUUAAAACUAAUGAAAUGAGUCCGUAAGUACUUCUACAAAGGAACCUCUCCUGAUCUAGCCGCGUUUACCCGUCAAGAAGUGCACUUGUUCUUCCUUGGAGGGAAGACCAAGGGGUGAA  >Ss-AA_clean.1_(paired)_contig_283  CACUAGUUUUAAAACUAAUGAUGUGAGUCCGUAAGUACUAUUACAAAGGAACUUCUCCGAAUAUAGCCGCGUUUACCCGACAAGCAGUACACUUGUUCUUCCUGUGAGGGAAGACCAAGGGGUGACGGUGGACCGCUACAGCGUUUAAGCUUUCACGGUUAGCCAUCACUCGGACGCUUGCUGGCCAGGAGCUUCCACGACCCUUUGGGAUUUCCUUAUGUAAGAAAACCAAUCUACCUAUGAUGUUACCAUUACAGGUUAGGUUGGGGAUCCUACGGAAGGAUUUCCUACUAAUAUCUUACGUCUUGACAGUUUUGCAACUAUCAAGAAUGAUAUUAGGUACAGGGGUCAUCGAAUCAUAUGACAGCAUCACCCGUCCCUGUUCUGGGAGUAAUCCCUUUACAGGAGCGAGCGUUGCUAUCGUUAUGAAACGAAUGGGGAUACGUCGCAUAGAUGAACCCACAUCUUUCACUUUCCCUUGGUGUUCAACUAGUGGGCCAAAUGGGGUAUCCAUUGCUACCUCUUUCUCAGACUUGAUGCAUAUUCCUCAGGAUCUUCUUGAGUGUUUGUACACUCUCGGAGGUCCUGAGUUUAAAGAGAAGGUCUCUAGCCUAAAAGGAUUUGUAGAAACCACUAAUGGUAUUCUAUGAAAACUUACUAAGUUAGAUCCAGAUAAAACUGGAACCCUUCUCCGACGAAUAUCCAUCAAGAAAGAUAAGGAGUAUAAAUCCCGACCUUUUGCGAUUGUCGAUUACAUAACCCAGAGCGCGUUGACCCCUUUGCAUGAUAGGUUAUAUCAUGUAUUGAAGUCAAUACCUCAGGAUUGUACUUUCGACCAAAACAAAGGGUUCAAGGACUUACUGUACGGAGGUGGCCCAUACUACUCGUUUGAUCUAACCUCGGCUACGGACCGAUUCCCUGUAUUCGUACAGAGAAUGGUUCUUAGCUGGUUAACAAGCG  AGAAAUAUGCGGACGCCUGAGUACAGGCCAUGGUUUCACUC  >Ss-AA_clean.1_(paired)_contig_284  CUAGCCGCGUUUACCCGACAAGUAGUUCACUUGUUCUUCCUGUGAGGGAAGACCAAGGGGUGAAAGUGGACCGCGACAGCGUUCAAGCUAUCUCGGUUAGCCAUCACCCGGACUCUUGCUGGCCAGGAGCUUCCACGACCCUUUGGAAUCUCCUUAUGUAAGAGAACAAACCUACCUAUGAUGUUACCAUUACAGGUCAGGCUUGGAAUCUUGCGUAAGGACUUCCUACUAAUAUCAUACGUGCUGACAGUUUUACAACUGUCACGGAUGAUACUAGGAACAGGGGUCAUCGAAUCAUAUGACAGUAUCACUAAACCAUGUACAGGGAGUAAUCCCUUUACUGGUGCAAGUGUAGCUAUCGUUAUGAAGCGAAUGGGGAUAAGUCGCAUAGAUGAACCCACAUCUUUCACUUUCCCUUGGUGUUCAACUAGUGGUCCGAACGGGGUGUCCAUUGCUACCUCUUUCUCCGACUUGAUGCAUAUUCCUCAAGAUCUUCUCGAGUGUUUGUUCACUCUUGGAGGUCCUGAGUUUAAAGAGAAGGUUUCAAGUUUGAAAGGAUUCGUAGAAACCACUAAUGGUAUUCUAUGAAAACUAACAAAAUUGAAUCCGGAUAAAACCGGAAAUCUUCUCCGUAGAAUAUCUAUCAAGAAAGAUAAAGAGUACAAGUCUCGACCGUUUGCAAUUGUCGAUUAUAUAACCCAGAGUGCAUUGACUCCUCUACAUGAUAGAUUAUAUCGCGUAUUGAAAUCAAUUCCUCAGGAUUGUACUUUCGACCAAAACAAAGGGUUCAAGGACUUACUGUACGGAGGUGGACCGUACUACUCGUUUGAUCUAACUUCAGCUACCGACAGAUUUCCUAUAUUCGUACAGGAAAUGGUCCUAAGCUGGUUAGUUAACGAGCAAUACGCUUCCGCCUGAGUACAGGCAAUGGUGAAACUUCCUUUCAGAACGCCAGAAGGCACUGAGGUAAAGUUCGAGUGUGGACAACCUCUAGGGGCAAAGUCCUCUUGGGCGAUGUUUACCAUCUCUCAUCAUUUUGUGGUCCAGUAUUGCGCUAUGGUUUUAAACAUAGACAACACUAGAUACAAAAUCUUGGGGGAUGAC  >Ss-AA_clean.1_(paired)_contig_1263  UGUCUAUGUUUAAAACCAUAGCACAAUACUGGACUACAAAAUGAUGAGAGAUGGUAAACAUAGCUCAUGAUGAUUUCGCCCCUAGAGGUUGACCACACUCGAACUUUACCUCGGUACCUUCUGGUGUGCUAAAUGGUAGUUUCACCAUUGCCUGUACUCAGGCGGCGGCAUACUGCUCGUUUACUAACCAGCUAAGAACCAUUUCCUGUACGAAUAUAG  >Ss-AA_clean.1_(paired)_contig_2610  CGUCAAAUACCUGAGGGAUGGCGAGUGAACCGAACCCUGCAGCCUCAGCAAACCGUAAAAGGGCAGACUGCGCUACCAGGUAAGGUUUAUUGUACAUCAGGAGAUAAGAUAGAAGCGUGGCAGAGCGGACCAGACUAUUAACUAGUCUGGGUUUAUGCUCCUUUCCAAUCUUCAAUAAGUCCUCUCAGAACCGAGGAGUGUUU  >Ss-AA_clean.1_(paired)_contig_238  CCUGCGUUUAAGCAGGAAGAGGGACUCCGAAGAGUAUUUUUAGAGUUAUAAGACGAUUGAAAGUCUUUGUUUUGAGCUAGAUUUUAAAAUCUCCUCAAUCCAAAUUCUCUCAAGAAUUCUUUUCAACUUUAACAAUAUUACCGGAAAUUAACGGUUUUAAUUGUGUUAGUGUGAAUUGAAUAUUAUUAUCCAAUACACUUUCUCAUGUAUAAACCGGAAGCAGAGAAUCAGUUGUAUCUUGAUUUAUACGACUUAUAAAAGUCAUAUAUUCAAAAUCAUCCUCAAUUCUUUCUCGUCACAAUCCUUUCAUUGAAAGUUUUCAAAGUUUUGAUAGAGUACUUACUGACACCGUUUUAUUACGGUGAAGGUUAGCUAUUUUAUCAAACUUUUCAACUCUUAAUGAAAGUGCCGAGUCUACUAAAGUAGUAUUAGGAUCAUUUUCUCAUUUAAUGAUUUUAUCUUUCAUUGAUGAUAAAUGAUUAUAAUAACCAUUUAGUAGAGGGUAUCCAGAUAAGAUAUUUAAAUCCUCAUAACUAGUUUUGAAUUUAUUUUCAAUUCUAGUAUAUUCGGUUAAUAUCUGUCUAGAUACUUUCUCUGCUUCAGACACCAUUCCACCUGAUAAUAAUAGCUUCAUAAAAUGAAGAAUUAUAUUAUUACCAGGUACCAUGAAACUAUCUUCUCUAGCCUUAUUACAUAGAUAAGCUCUAAGCUCAUCAUAUGUCAAUUUGUCUAAAGAAUAUCUCAUGGCAUGGUGGAAAUCAUAUAGCAUUUUAUGCAUUUGUCUAAAAGAAUUUCUUCUUUUACCAAAUGGUAACCUACUAUAUAACUCACACAUUAGAUUUAUCAAAGGUAUUGGUUGAAUUGGAUUUUUAUCAAAGUAAGAACAAAGGGUUGUAUAUACAACGCCUGGGUUCUUCCAUUGAUUAAGUAUUCCUUUUAAAGGAAUUCCACUUAUUUCAAUACCUCCCUUUAUUCAUCUUUUAGCAAAUUCAUAUGUAUCUUUUGAUACAUGUGUUUUGUUAAUAGAUAUAUCAACACCAAGUCUUGUCAUAAUAGUUAUAUAUUUAUUAGCGACUUUAUCGUGUUUUAUAACGAUAUCGUCACCUAAUAUAAUAUAUUGACUAAAGUUGUAUUCACCGCAUAGAUAUGCAGCUCAUGCAACGACAAGAUGAUGCGAGAUCGUAAAGGCGGCUCAGGAACUAUAAGCUCCCAUUGGUUGACCAACAUUAUAAAAAUGUUGUUCACCAUUGGAAUCAAUGUAAGGUCUUGAGGUUAAUAAAUCUGCUCAUGCAUCACAAAAUUGCUUGUUUUCAUAAAUAUAUGAUAACAACUUCUUUUGCAAUAGUAAUGGAAAUCGGUCCGUAGCUGCACUAAGAUCUAGAGAAUAGAACUUAUGGUGAUUUUGGUAUCAAGUGUGUUUUGGAUCUUGAGUAAAAGUCCUAUCACAUUUUAAUGUACUUAAUUUAUUAAGUAGCAUUUCAUGAAUAGGUCUAAGAGUAAAUUGACUAUGGUAAUCUACCAUAGCAAUAACUCUUCUUUUUAAUUCAGGAUCUUUAACAAUUGAUAAUUUACCUGUAUAAUUAGUCCAUUUAGGAUUAAUUAUAUCCUCACUUUUAUUAAAAUUAUUAUUCCAUGCAGUACUAUAGAAUUUGAAAAAUUCAUCUCUAUAUGUACCUAACAUGGUUGAUAUAUUUUGUAAUUGUGGGUAACUUAAUGAUAAUAUUGAUCAUAAUGAUGAAUAUGUAGCAGGUCCAUUAGGACUUGACUUCAUACUUACAUAAUGAUCUUUAACAUCAUAAGUAGGUAAAUCUGCUUUUAGAUUAUUUUUACUUAUUCAAUUCUUGAUAAACCAUGCAGGGAUAGUAUAACUUUUCCCUUUGUAUGGUAGAUCAAUUGUUGAAUAAUCUGGUUUGAUCUUUAAUUCUUCUUUCUUCGUUGGGACAACAGUCCUAGUAAAAGAAAGUAGAGUUAAUAAAAUUCUUAAUUCUUUAUUAGUUUUAACAAACUUUUUAAGAUAUAAGAAUUUCUUAGGUCAACCAGAAUGAUCUAACGCAACUCCUUCUUGAUUUGACAACAAAGGUUGUCCACAUAUAUAUCUUGUAAUAUGAAGCUUUACAGCUUUAUAAUACUUGAUCGUAUAUUUAAGGCCAGACUUUUGUCUCAUUUCGUCGAAGGUUUCCAAAAACCGAGUAAUUUCUCAUGUAUUAUUAUUAAAAUUGAAACAUAGUCUUAAUAACCUUAUUAUGAUUAUAUUUCUAAUUUUAUUAUUCAUGAGAAUCUUGCAUUUAGAUAGUAUCAGUUAAUACUACGUACCGUCCAAGUCCGAUACUUCAAUUCCCAUAUAAAGGAAUUGUAUAUCUUGGCUUGGUCAGUUACUAUAAAAGAUAGUGAACAAAUUCAUUUUGUAAGAUCAUAAGCUGAUUUCCACUGCUGUCGCGUUAGAUGGCUGUCUAUCAUAUGAUAGCCCUAAUCGUCACGCCGUGGUCACUUAAUCUCUCACACUGAUAAUUUGAUAUAUUCUUAUAUCGCAACUGAGCCUGCCAAUUACCCAAUUUCGUGUUUUCAUAACUCAUCAAGUGAUAGACAUUAUGUCAUUUCCAUCUUGAGUCUUGUUGAUGAAACGGAUAACGGGGAUAAUCUUCAAUUAAUCUUAUCUUUGAUUAAUCUAACCAUAAUUCUUUAAUUAGAAUUAUCCGAUUCUUCGGGAUUCUCCUGAUCUAGCAAUAGAUAGAUCGGA  >Ss-AA_clean.1_(paired)_contig_188  GAUCUACCGGAUAUUAGGUAAGAGGUAUAUUGAGCAAGUGGAAUCAAUGAGGAAGCAAUCAGGUUUACCAUUUACAAUCCGUUACAUGAAGACAGUCCGUCUUCACAUAACGAGAUAUAUAAGUGGCCAACCGCUACGAUCAAAUUCUUCACUGGUUUCGCUAACCAAUGGUUUUCCUACAAAGUUCCUAUAUUUGAAGGAAUUCAUUGAUAAUGGUGAUCCCAAAAGUGUUAGAUUUGUACUAACACUGAUGGGUUACACCAGAUCAAUUAUUCCAACGAAUAAGGAGAAGAACCUAGUUAAGAGGGAUUAUUCAUCUAUAUCGCUCCCGUUUAAGGGGGAUAUAGAUUUUAACAUUCCUGAUGAGUUUAUUGCUAAAUUCAUGAGGAAGUUUAAUCUAAGCCUCCACCCGAGAUGGGAUAACUCAUUACAUUACAUUAGUAAUAAGUCAUCACCUCUUGGUAAGGCGACUCUUACUGGUCCAUUUGCACUAUUCCAUAUGGGUCACUGGAACCUUAAUAUGUUAGAACAUUUCCGAUGUUUAAUCGGAGAUGGUUCUUUCAAAAUUAUGGUUGGUGAGUUCCUUGAAGUUGUAUUCAAGGAUCACCGAGCGUUCCAUACGGGCGAUAGUACAAAUGGUAUAGGAAAAAUAUCAGUUGUUGAAGAUCCAGAAUUGAAAAUGAGACCAAUAGCUAUGGUUGAUUACUAUAGUCAAUUGGUUCUUAGACCAAUCCACGACGGUGUUCUUCAGUUAUUGAAGAAGCUACCGCAGGAUAGGACUUUCACUCAAGAUCCUUUCAACAGAUGGGGUAAGACCAUGGGGCACAAGUUUUGAUCACUUGAUCUAACUAGUGCAACAGAUCGUUUCCCGAUUUCUCUUCAAGAGCGUGUAAUAUCCCAUCUCCUAGGAGAUGCAGAUAAGGCACGGGCUUGGAGAAACAUCCUUGUUGAUAGGGAUUAUAAACUUCCCAGUGGUGACCUAACACGUUACAGUGUCGGACAGCCAAUGGGAGCUUAUAGUUCCUGGACUACUUUUACUCUAACACACCAUCUUGUUGUUCACUAUUGCGCAUGAAAAUGUGGAAUAGAGGACUUUGACAAGUAUAUAAUGUUAGGUGACGAUAUUGUGAUUAAUCACGAUAAAGUCGCAAGACAAUAUAUAAAGGUCAUGACAAGACUCGGUGUGGACAUCUCAAAUGCAAAAACGCAUGUAUCUAAAAACACAUACGAGUUUGCCAAAAGAUGAGUAAAGAAAGGCUUAGAGAUCACGGGUCUUCCUCUAAGAGGUAUAGCCUCGAAUACUAAUAACCUUCCAGUUGUUGUAAAACAAUUGGUUAGUUAUAUGUACUCGAGUGCUACUCAUUGAAGAGGAAGCACUACUGAGUUGGUCUUAAGUGUCUUCAAAGGGAUCAGGAUUAGAGGGAGAUUUAUUUCUCACUCAUCUCUUAGAUCCCAAGUUGAAGAUAUUGUCUUUGCUAUAAGGCAGUCACUUAAGCUUAACUCCGAUCAGGAAAUACGUAAGUA  >Ss-AA_clean.1_(paired)_contig_789  AACGUGUGGAACCUCCGGAUGGUAGCUUAUAAUACCUUUCAACCAAUAUAUUACGCCAAGAACGCGCCUUUUAGUCAUCCUUUAAAAGAUGUGAAAUUACACGCUCUUGGAGUGAUAUAGGGAAACGGUCUGUAGCACUAGUUAGAUCAAGCGAUCAAAACUUGUGCCCCAUGGUCUUACCCCAGUUGUUGAAGGGGUUCUGGGUAAAAGUCCUAUCCUGAGGAAGAUUUCUUAAUUUCUUAAGAAUCCCCUCAUGGAUUGGUCUCAGAAUCAACUGACUAUAGUAAUCAACCAUAGCAAUUGGUCUAAGCUUCAACUCAGGAUCUUCAACAACUGAUAUCUUACCUAUACCAUUUAAACUAUGCCCAAUGGUAAAUGAACGGUGAUCCUUGAAUACAACUUCAAGGAAUUCACCUAUGAUGAUGCGGAAAGAACCAUCUC  >Ss-AA_clean.1_(paired)_contig_1716  CCUCAUUGAUUCCACUUGCCUAAUAAAUCUCUUACCUAUGAUCCGAUUAUGAAAUAGUAAGUCUAAUAACCUUAAAAUUAUUAACUUGCUAGUUUUCAUGAUUAUAUUAUAGAAUAAGAGCAUCUACAGCGUCCAGUUGUUAUACAUAGCUGGAUCCCGGCUUUACCUUCGGGUGGGCCACUAUAGAUUCACCCGCAGGUGGCCUGCGUGACAGACACGAAGUCUGGCAUCGGUGCCCGCCAACGGUGAAGCAAGUCAUCCAAGAUAAGUUUUAUUAAAACUUACCCGGACUAUAAUCUACUCAGUAACCCACAGCUUAUGAGGCCAUGGCUGAGUUGAUCGCAGGAUACAUACUAUUGUAUGUAUGGUUCGAUAGAACCGCUUUUGGGGACCGAGAGGAGAUCGGAA  >Ss-AA_clean.1_(paired)_contig_781  GAUCUACCGGAUAUUAGGUAAGAGGUAUAUUGAGCAAGUGGAAUCAAUGAGGAAGCAAUCUGGUUUACCAUUUACAAUCAAGUAUAUGAAGACUGUACGUCUUCAUGUUACUAGAUAUAUAUCUGGUGAACCGCUAAGGUCCAAUUCUUCACUAGUUUCAUUAACUAAUGGUUUUCCUUCAAAGUUCUUGUACUUAAAGGAUUUUAUCGAUAGAGGUGAUCCACGAAUGGUUAGAUUUGUUCUAACAUUAAGGGGUUACACUCGUUCAAUAACACCUAC  >Ss-AA_clean.1_(paired)_contig_312  GGAGACCUUUCAGUCUCCAAAAGCAGCUCAAUAGAGCUAUACAUGGUUUUAAUCAUGUAUCCUGCGAUCAAUUCAGCCAUGACUUCCAUGCCAUGGAUUACUGAGUAGAUUGUUAGCUCAAGAUAGUUUAAUAAACUAUUUCUGAGUGACUUGCUUCACCGUUGGCGGGCACCGAUCAUGGGUUUAAACCAUGACGCAGGCCACCUGCGGGCGAACCUAUAGUGACCCACCCGUAGGUAGAGUCGGGAUCCAGCUAUGUAAACACAACUGGACACUAUAGGUGCUCUCUUUUAUACAACAAUCAUGAAAACUAGCAAGUUAAUAAUUUUAAGGUUAUUAGACUUACUAUUUCACAGCCGGAUAUUAGGUAAGAGGUAUAUUGGGCAAGUGGAAUCAAUGAGGAAGCAAUCUGGUUUACCAUUUACUAUCAAGUAUAUGA  >Ss-AA_clean.1_(paired)_contig_189  GUUUACCAUUUACUAUCAAGUACAUGAAAACAGUACGUCUUCAUGUUACAAGAUACAUAAGUGGUCAACCACUAUUGUCUAAUUCUUCUCUGGUUUCACUAACAAAUGGCUUUCCUACAAAGUUCUUAUACUUGAAGGAUUUAGUAGAUAGUGGUGAUCCAAAAAUGUUGAGAUUAGUCUUAACAUUGAUGGGUUAUACCCGAUCUAUAAAACCAACAAGUAAAGAAAAGAACCAAGUAAAGGUAGAUUACUCUUCUAUAUCUGCCCCGUUUAAGGGUAGAAAGGAAUUUAAUAUUCCUGAUGAAUUUAUUACUAAAUUUAUCAAGAAGUUUAAUCUAGGCCUUUACCCAAGAUGGGAUAAUUCAUUACACUAUGUGAGUAAUAAAUCAUCUCCCUUAGGGAAGGCAACCUUAACUGGUCCAUUUGCACUAUUCCAUAUGGGACACUGAAAUCUUAAUAUGUUAGAAUAUUUCCGAAAAUUAAUCGGAGAUGGUUCUUACCGUAUUAUGAUUGGUGAGUUUCUUGAAGUAGUAUUCAAGGACCACCGAGCGUUCCAUAGUGGAUGUAGUACAAAUGGUAUUGGGAAGAUAUCAGUUAUUGAAGAUCCAGAGUUAAAAAUGAGACCAAUAGCUAUGGUAGA  >Ss-AA_clean.1_(paired)_contig_190  GUUUACCAUUUACAAUCAAGUACAUGAAGACUGUCCGUCUUCAUGUGACAAGAUAUAUAUCUGGUGAACCGCUUAGAUCUAAUUCUUCACUGGUUUCUCUAACUAAUGGUUUUCCUUCAAAGUUCUUGUACUUAAAGGAUUUUAUUGAUAGAGGAGAUCCAAAAAUGGUUAGAUUUGUUCUAACAUUGAUGGGUUACACCCGUUCAAUAAAACCUACAAGUAAAGAAAAGAACCUCGUAAGUGUUGAUUAUUCUUCUAUUUCAUCCCCUUUUAAAGGGAGAAAAGGUUUUAAAAUACCAGAUGAAUUCAUUAAGGAAUUCAUUAAGAAGUAUAAUCUUGGCCUUUACAUAAAGUGAGAUAACUCAUUACAUUACAUUAGUAAUAAGUCAUCUCCAUUAGGUAAAGCCACACUAACAGGUCCAUUUGCACUGUUCCACAUGGGACACUGAAAUCUUGAUAUGUUAGAACAUUUCCGUAAGUUAAUCGGAGAUGGUUCUUUCAAAAUCAUGAUUGGUGAAUUCCUUGAAGUUGUAUUCAAGGAUCACCGAGCAUUCCAUACGGGUUGCAGUACAAAUGGUAUAG  >Ss-AA_clean.1_(paired)_contig_790  CUAUACCAUUUAAGCUAUUCCCGAUGGUAAAUGAACGGUGGUCCUUGAAUAUAACUUCAAGGAAUUCACCUAUCAUGAUACGAAAAGAACCAUCUCCGAUUAAUAAUCGGAAAUGCUCUAACAUAUCAAGAUUUCAAUGACCCAUGUGGAAUAGGGCAAAUGGACCAGUAAGUGUCGCUUUUCCCAUAGGUGAGGAUUUAUUGCUUAUAUAAUGUAAGCUGUUAUCCCACUUUGGAGAAAAAGAGAGAUUAUACU  >Ss-AA_clean.1_(paired)_contig_132  AACAUUAAUGGGUUACACUCGUUCAAUAAAACCUACAAGUAAAGAAAAGAGCCUAGUUAAUGUUGAUUAUUCUUCUAUUUCAUCUCCUUUUAAAGGGAGAGCAGGAUUUAAAAUUCCUGAUGAGUUCAUUAAAGAAUUCAUUAAGAAGUAUAAUCUCGGCCUUUACAUAAAGUGAGAUAAUUCAUUACAUUAUAUUAGUAAUAAAUCGUCUCCUUUAGGUAAGGCCACAUUAACAGGUCCAUUUGCACUGUUCCAUAUGGGACACUGAAAUCUUGAUAUGUUAGAACAUUUCCGUAAACUUAUCGGAGAUGGUUCUUUCAAAAUCAUGAUUGGUGAGUUCCUUGAAGUAGUAUUCAAGGAUCACCGAGCGUUCCAUACUGGUUGCAGUACAAAUGGUAUAGGAAAGAUAUCAGUUAUUGAAGAUCCAGAGUUAAAAAUGAGACCAAUAGCUAUGGUAGAUUACUAUAGUCAAUUGGUUCUUAGACCUAU  >Ss-AA_clean.1_(paired)_contig_66  CUCAGGAUAGGACUUUUACUCAGGAUCCCUUCAACAAGUGGGGUAAGACCAUGGGGCAUAAGUUUUGAUCGUUAGAUCUAACUAGUGCUACAGAUCGUUUCCCUAUAUCACUUCAAGAAAGAGUUAUAUCAAAUCUCCUAGGAGAUGCUGAUAAGGCUCGGGCUUGACGUGAUAUACUUGUUGACAGGGAUUAUAAACUGCCAACUGGAAGUUGAACACGUUACAGUGUUGGACAACCAAUGGGAGCUUAUAGUUCUUGAACAACAUUUACUCUAACACACCAUCUUGUGGUCCACUAUUCCGCAUGAAAAUGUGGUAUAGAGGACUUUAACAGAUAUAUACUGCUAGGUGACGAUAUUGUGAUUAAUCACGAUAAAGUUGCUAGACAGUACAUACGAGUUAUGCACAAACUUGGUGUGGACAUCUCUGUUGCAAAAACACAUGUAUCGAAAAAUACAUAUGAAUUUGCGAAGAGAUGAGUAAGGAAAGGCAUAGAGAUCACGGGUCUACCUCUUAGAGGAAUAGUAUCAAAUACAAAUAAUCUCCCAGUUGUUGUAAAACAACUGUUGAUGUACUUGUAUUCAAAUACUACUCUUUGAGGAGGUAGCACUACUGAAUUAAUCUUAAAUGUCUUCAAGGGAAUUAAGAUAAGAAAGAGAUUUAUAUCUCAAUCCAAUCUUCGUUCCCAAGUUGAAGAUAUUGUCUUUGUUAUAAGACAAUCUCUCAAACUUAACUCUUAUGAAGAGAUACGAAGGUAUCUUUCUAAGAAGAUUAAGCUUGAGGAAUUGAUUAUUCCAAAGGAAGAUCAAGUUCACAGUGUUAUCCGUGGGAUCUUGUGUCUAGGUCUAGUAGAGGCGGUUGAGGACUCAGCUAGUCAAAUAGCUAGGUUCUACAAGAAGUUUGAAUCAAAGUUCCAGGUUGGAGACUUUGACUUGAACCUCUUGAAACGCCAUCCCAUGGUUCNNUCUUGCAGUCUAUAAUAAAAUCUUGCACAUGAAGCGUACCCUUUUAAGAGUGCGUAACAUGGACGAGAUGGACCUUAUAGAUGCGAUAUCCUUAAUGAGGAUUGACAGCCCAGAUAAACUGGUAUCAACCCUUAGAAAUUCUUCCGUGUCUAUAACCCAUAUAGACAUGCUUUGAAGACAUGGUUUCCAGAGAUUGGAGUACAUACUGAAGUUAAUUAUCUUAAUUUUAGUGAAGUAGAUCCAUUCUCUAUAACC  >Ss-AA_clean.1_(paired)_contig_1018  UGGGUUCUAAGUUUAGAUUGAGAUAAAAGUCUCUUUCUUAUCUUGAUGCCCUUGAAGACACUUAAGACCAAUACAGUUGUGCUUCCUCCUCAAAGGGUAGUAUUUGAGUACAAAUAGCUUACAAGUUGUUUCACAACAACAGGUAGGUUAUUCGUAUUUGAUACUAUUCCUCUUAGAGGAAGACCCGUGAUCUCUAUGCCCUUUCUUACUCAUCUCUUCGCAAAUUCAUACGUAUUUUUAGAUACGUGUGUCUUCUGCUUAGAGAUGUCCACACCUAGCCUAGUCAUAAUACGUAUAUAUUCUCGGGCGACUUUAUCAUGAUUAAUCACGAUAUCAUCACCAAGAAGUAUAUACCUAUUAAAGUCCUCUAUCCCACAUUUAUGUGCGGCAUAGUGGACAACUAGGUGGUGUGUUAGAGUAAAAGUAGUCCAGGAACAAUAAGCACCCAUUGGUUGUCCAACACUGUAACGUGUUAGAUUACCAUUCGGUAACUUAUAAUCCCUUUCGAUAAGGAUAUC  >Ss-AA_clean.1_(paired)_contig_46  AAGGCCCGAGCCUGGAGAGAUAUCCUUAGCGAAAGGGAUUAUAAGUUGCCUAAUGGUUAUCUAACUCGUUACAGUGUUGCACAACCAAUAGGUGCUUAUAGUUCCUGGACCACUUUUACUCUAACACACCAUCUAGUUGUCCACUAUGCCGCAUACAAAUGCGGAAUAGUAGACUUUAAUAAAUAUAUACUUCUUGGUGAUGAUAUCGUGAUUAAUCACGAUAAAGUCGCCCGAGAGUACAUACGUAUUAUGACUAGACUGGGUGUGGACAUNNNNNNNNNNNNNNNNNNNNNNNNNNNNNNNNNNNNNNNNNNNNNNNNNNNNNNNNNNNNNAGAGGAAUAGUAUCAAAUACUAAUAACUUACCAGUUGUUGUAAAACAAUUAGUAAAUUAUAUGUAUUCGAAUCCUACUCUUUGAAGAGGAAGCACAACUGAAUUGGUCUUAAGUGUCUUCAAGGGUAUCAAGAUUAGAAAGAGAUUUGUUUCUCAAUCUAAGCUUAGAACCCAAGUUGAAGAUAUUGUCUUUGUUAUAAGGCAGUCACUUAAGCUUAAUUCCUAUCAAGAGAUACGUAAGUAUCUCUUGGAUAAGAUUAAACUUGAGGAACUGAUUAUUCCAAAGGAAGAUCAAGUUCACAGUGUUAUCCGUGGGAUCUUGUGUCUAGGUCUAGUAGAGGCUGUCGAGGAAUCGGCUAGCGAGCUAACACGGUUCUAUAAAAGAUUUGAAUCAAAGUUCCAAGUUGGAGACUUUGAUCUAUCUCUUUUAAAACGCCAUCCCAUGGUUCUUGCAGUCUAUAAUAAAAUCCUGUCCAUGAAACGGACCCUGAUGAGGGUUCGUAAUAUGGAUGAGAUGGACUUAAUAGACGCGAUAUCCUUAAUGAGGAUAGAUAGCCCAGAUAAACUGGUAUCUAACCUUAGAAACUCUUCAACGUCUAUAACCCAUAUAGACCUGCUUUGAAGACAUGGUUUCCAGAGAUUGGAAUACAUUACUGAAGUCAAUUAUCUCAACUUCAGUG  >Ss-AA_clean.1_(paired)_contig_37  CCGAGUCAGUUCGCUGGCCGAUUCCUCGACAGCCUCUACUAGACCUAGACACAAGAUCCCACGGAUAACACUGUGAACUUGAUCUUCCUUUGGAAUAAUCAGUUCCUCAAGUUUAAUCUUAUCCAGGAGAUACUUACGUAUUUCCUGAUAGGAGUUAAGCUUAAGUGACUGCCUUAUUACAAAGACAAUAUCUUCAACUUGGGUUCUAAGUUUAGAUUGAGAUACAAAUCUCUUUCUAAUCUUAAUACCCUUGAAGACACUUAAGACCAAUUCAGUUGUGCUUCCUCUUCAAAGAGUAGUAUUAGAAUACAUAUAAUUUACAAGUUGUUUUACAACAACUGGUAGGUUAUUAGUAUUAGAUACUAUUCCUCUCAGAGGGAGACCCGUGAUCUCUAUGCCCUUUCUUACUCAUCUCUUAGCAAACUCAUAUGUAUUUUUAGAUACAUGAGUCUUUUGCUUUGAGAUGUCCACACCAAGUUUAGUCAUAAUACGUAUGUACUCUCGGGCGACUUUAUCAUGAUUAAUCACGAUAUCAUCACCAAGGAGUAUAUACCUAUUAAAGUUAACUAUUCCACACUUAUGUGCGGCAUAGUGAACAACUAAAUGGU  >FirsU_Contig27  CUCAAUCUAAACUUAGAACCCAAGUUGAAGAUAUUGUCUUUGUUAUAAGACAGUCCCUUANAGCUUAACUCCUAUCAGGAGAUACGAAGGUACCUCUUGGAUAAGAUUAAACUUGAGGAAACUGAUUAUUCCAAAGGAAGAUCAAGUUCACAGNUGUUAUCCGUGGGAUCUUGUGUCUAGGUCNUAGUAGAGGCUGUCGAGGAAUCGGCCAGCGAAGCUGACACGGUUCUAUAAAAGAUUUGAGUNCAAAGUUCCAAGUUGGAGACUUUGAUUUAUCCUCUUUUAAAACGCCAUCCCAUGGUUCUUGNCAGUCUAUAAUAAAAUCUUAUCAAUGAAGCGUACCCUUAUGAGGGUCCGUAACAUGGAUGAAGAUGGAUUUAAUAGACGCGAUAUCCUUAAUGAGGAUAGAUAGCCCAGAUAAACUGGUAUCUAANCCUUAGAAAUUCUUCAGCGUCUAUUACCCAUAUAGACUUACUUUGAAGGCAUGGUUUCCANGAGAUUGGAAUACAUAACUGAAGUUAAUUAUCUUAAUUUCAGUGAAGUAGAUCCGUUCUCNUAUAACCAUGCUUAAACCAUGAGAAUCCUACUAUAUCUCUAACUUGUCAGAUUUAUGUGANCAAGCCAGAAAUCCUAAAGACUCCGGUUACUACAAAUAUGUAUUUCUAGUAAUGUGCUUC  >Ss-AA_clean.1_(paired)_contig_308  AUUCCGAUCUCCUCGUGAGAGGUGAUAACCAAUGCAACCCCACAUAUCAUUUGAUUAAUCAGAUGAUAUGAUUUAUGCAUUGUCCUAUAGAUUUCAAGCUAUAGGUAGUACAACUACAUUCUCAGAAGUUGAGGAUAACAGGCCAAAACCUGUAAUCUUUCCUCUAUGAGACUAGCGAAGCGAUCAACUGAUCUCAUUAAUGAGAAAUUCCGUCCUUCCACUAUACUUUUAGUACUAGUAGGUAUGGCGAAAUUUCGCAUGUAAUACGAUCAGUCAGAGCCCGUACUAUCGAUCUUCUGAAUCCUCUUCAUUAAUUCAUCAUACUCAUUAUAAAUAGCUAUAGCAGCUAAGUUUAUAGGAGUAUCUUGUCAAAAUGAUUUGGCCUUCAGAAUUUCGAAACUGGAGUGUCCUUGGUAUACAUCCUCUAUAUACCUAUCCCAUUCUGUCCGCAUUAUAUUAACAGUUUCCUUUAAUGGAAAGUUAACACGAUCGUCAGAAAAGAAUGUGUGUAUAGCGGAUGAGCUGAAGACCUCAACAGCAAUAUUUGAGAUUAUGUUCACACAUAUCUCAAGACUGAUGUGAGGAAGGGGUAGUUGAAGGGUGUGAGACAGUUCAUUAAUGAACGUCUCACCAUCCUGAAGACCCCGUACUAGUGAUAGUACACCUUCGGCUGUCUUAACUCUCCCUGUUAACUUCUUACGAAGUCGAGAGGGAAGAUUAAGAACAAUACCAUAGAACAAUUCAGCGCUUGAAGCAAUUGAACUACACUCUCAACCCCUUACCUUCUGCUCGAGAAGAAGGUCAGUAAGAGCUGAACAGCUCUUUCUCACUUCCUUCAAUGAGCUAAUCGGGAAAGGAGUAAUCUCGUCACCUUUAUAGUAUAUUCUUUUCGCAAAUUCAAAGAAGUCCUUGGACUUAUGAGUCUUUGCUAAAGAGUAAUCUACAUGAAGUGACUUGAUUAUUUUCAUGUAUAGUUCACCCACUUGAGCAUUACCAAUUACAAUAUCAUCUCCUAGUAGGGCAUAAGGUAGAGAUUUCCAUCUCAUACCUAAUUCCCUACAGCAGUAGUAGAUAAUGUAAUGAUGUGUUAAAGCAAAGGAGUUGAAUGAUGAGUAUGCACCCAUUGGAUUACCGGCAUUAUACUUGUAUAAUGAACCGCGAUACUCAAAUGGGGUGUCUACCAUCACACUACUCCAAGCUUCAACAUACUCGUGGGGCAGUUGGAACUUCAGUAGAUCUUUGAUAACCGAAAUUGGAAAUCUAUCUGUGGCGGCCGAAAGGUCAACACUGUAAUAGAUGUCGCAAUCUCCGUUAAAAAGGAGUUUCCUGAACUUUCCUUGCUCCAAGGUACAGUCCUGCUUAAUCUUCGAAAGCCCCCUAGCAAGUCAGGAAUGAAGUGGUUUAAGAGCUAGCUGACUAAAAUUGUCAACUACUCCUACAACCCUCAUCUUUCCUUCCUUAUCGGGGAAACAAGAAAGCCGACGACAACAAUUCUUGUUGUUAUCAAACUUCUUGUAGUACUUUUGGAAGAACUCACUAAACACCCUUGGACUGAAAUUUCUAAUACGGGCUCCUACUAGGGGUGACAUCUUACUCAGAUUCAUCUUUAGAGAAGUCGGCAUAGAAUGAAUGUCAAGUAAAGCAGUAUUAAGUACACGGCCAUUUGGCCCAUUACUUAGUCUGUAAACCUUCUCAUUCACCUUCAGUUUGCGGGGUAGUGAGAAAGCAGUGAUAGGCUUGUAACCCAAUGAUCGUCAAAAGCUAGCCAUAUGCUUACUUAAAUCAGGUACAUCCCCUUUUACAGGUUGUGUGACUGUCGUAAGAUCUGGUUCACUAAUGAAUUUUAAGGAUCUUGUUGCUGUUAGUAUCGUUAGGACUAGAGCGAUUGCUCGCCUAUCUCCUCCACGUACUAAUGGAAUAAGAUCCCCUAAGACCACUGGGAUACCAUCGCUUGUUGAUCUGGCCAACGAAUUUCUAAGAGGGUUACCUGAUAAGUAAUUGUAAAAAUUACCUCUCACGGCCUUGAUAUACUUUACAGUAUAGUCUUGACUGUGAUUGUUCAGUACCCUUUGAAGUUUGUUGCAUAGAGCUUCGAAGUGAUGUAGUGGUAAUGAAGUACCGCAUGAAGCGAUACAUAAUCAUUUGACUACUUUGAAAGCAAAAGAGAAAAGUUCUAAACGAACCUUUCUACCAAAGCUAUUAUUUAGUUUUGUUUUCGUUUUCAUAGUAAGUAUAAAUAGAUUAUCGCCUAUUCCCCGACUAGCACUAUCAGGCUUGAAGGCCACUGUAUACUAAUAAUAGUCCCCAACCAUCUUAUGGAUAGGGACGAUAUAUUAGUAUCCUCCAAGGGAUUUAACCUCUACACGUACCAUUCAUUCCUGAAUGGCAGAAUUCUCACCCGUCCGUUCUGGACAGUGGUGAUCUAUGUAGGUGAGAGGGUAGUGCUUACCCUAGGCCCAGGGAUAUAUGGGACUCUGUUUUACCAGAGUGUUUUCCUCUUGCGAGAUCGG  >FirsU_Contig17  UACAUCUUAUCCGAUGCUCCUUCACAGGAGUCAUAUAUGUGGUGCAAAUCACAUAUAUGGNGUAGUCACUUGAAUGACAGAGUAUCAUGCAACCCUUCCGGUUGUAGGUUAUUCUUUUAUUNAUAAGAAAUACCUUCGGAACCGAUCUAGAAUUUGUUGUACACUAGUGCUCCCAAAACCAANUAUCCGCAGCACCGCCGUGGAAAAAGUUUUGGGGAUCUGAAAAUCUAGCAUUAUCAUGAUNCUCAUGAUUUUACUAAAUUAACAAAUUUAUGGGCCAUCUUAGUAUUAUUAACCAUAACCUNGAACCUUCGUUCUUUUAUGAGUCAUAAUACUUUCAAUGUCCAUAAGCAUAAGUGUAUCACNACAUCUCCUUUAAUGUUAAAUCACCCCUUUGAGAGUCUUGCGUUUCAGCAAUUCUCUCAANAGUGGUUCUCUAUACAUUGAAGGAUUGGAUGAACAUGGUCUUCUAUAAUAUUAUCUUCAANGUUCAACAAACUCGUUGAUUUUAUGAUAGAACUCCACUAUCAACCGGGAUGAUUUCUGUANCCAAUGCAGAAUAAGUCAAGUUAAAGACCCUCAAUACUUCAUUGAGGAUUAACUUUUCAGNCUCCUCCUACAAUGAACUGCGAAUUUCGUGUUCACAAGCAGAUUUGGUUCCUAACCUCAUNCUGGUGUGCUCAACGAGAAUCGAAGUCUCAUGAAGAAGGAGAUUGGCUUAAGCGUCUCCANAGAUCUUCCUAUAACCAUAAGGUUUCCUAGAAAAAUCUUGGACUUUAUACAAGAAAUCAGNCAACACUUUCAACUAGACUUCUGUCUGUUCAGGACUUGUUACCUUUCAAUUGUCAAUCAANAUAAUAUUUGAAAACAUGUUGACAUAUUUUCAAUAUUAUCUAGAAGACCUUUGAUCGGUANGACCGGUCACCUCACCACAUCGGGUAUCAAUCCAUCUCUUUGCAAAUUCAUAUGUAUGUUNUCGACACAUGAGUCUUUGCUUGAGAUGUUUCAACACCAAGUGCGUGAAGCAGUCUGAUGUNAAGUUUUAGCCACUUUAUUGUGGUUUAUUACUAUGUCAUCACCCAACAUUAUAUACCUGUNUAAAAUUAUCGAUUCCAUUAAGGAGUCCACAAUAUCUAACUAAUAUAUGAUGAGAAAGUGNUAAACAUAGGUCAGGAGGAAUAAGCUCCCAUAGGCUGUCCAACUCUGUAUGAGAUGGACUNCACCCUCAUAGGAGAAUUUUUCACCGACCAUAACAUCAGCCCAAUCUCUUGUAAAAGCAGNAAUUUGUCAGCAAUGACAAAAUCUGUUUUUGCAAUUCAAUUGGGAAACGAUCAGUCGCUGNCAGUCAGGUCAAUACUUCACAGUAAGUUCGUAUCCACUUUAAAGUGGAAAGCAGGAUCCUNGAGAAUAGGUUCUAUCUGAAUCUAUCUUAGAUAACGCUUUAAAUAGACUAUCUGAUAAUGNGUUUAAGGGUAACCUGAGACCAUCAAUCAAACAUACCUAUCACACGCAUCUUACAUUCCGNGAUCUUUAACAAUACUUAACCUCCUUACCUCUGGAGCAGCCUUGCUUCCGCAAGGCCUAANCUUCAUCAGGUUUCGAGAGUGAAUAUUGGUAUAGGUCCUUGAAGUAUUUGAAUAGACUAUNUUGAAAGCAUCCCCAUGAUUGCUGAGGCUGUAGCAGUACUAUACCUUUUCAUAGAGUAUANGAGCUGUUAAAGUCUGUGGCCCAUGGGGUCCGCUUUUAGUUGUCAACUCUAGAUCUGACANGACCAUGAUCAGGAAUUGAUAGACAUAUAUCAUUCUGGCUUAUAACUUUUCUAAGUAGGCNGGUUGUCAAGUUUAUUGAUAACAGCCGUACUUGGAUUAGUUAUAGGUUCCAGUGAUAUAGNGUAUAUCCUCUCCUUUCCUUGGUCUGAUUGUCCUUGAAAUGUUGAUUAAAGUAAACACAANAUUUAAUUAAAUUCAGUGAUUCUUUAAUCAAGGAUUCACACUCUUUAAAGAUUAGCGGAANAUCCUGUUCUUGUCAAUGAGACACCAAUGUUAUUUUCCAUUAAUGGAGAACCACAGUAGUNAUCUUGUGACAUGUAAACGUGCCUGUUUAAGGUACUUUACAGUGAAGAGGGUCCCAUUAUNUCUUGAGAAGAUUAUGAAUUCUUUUCAUAACAAGUCCAAUUACGUAGGUGUGUCUUCCAANUAUUUAAGAACAACAUACAGAUUAAUUUUAUUAAUUUAUAUGUUUGUUUUUUCAUCUUGGNUCUACACAUUCGAUUGAUUGUCACUGUUGGUGACCUGUUACCAUUCGAAAGUUUCUCCCUNUCUCUAUCAUAUUUCUCCACAUGGCUUUGCUUUGCACAAACAAAGUGAUCCACGCGAUUANAUAUGGUAAAGACUGGAUUGACAAUGGAACUGCAACAGGCGAGAGCAACAGAAGAAUAGGNGUGAGUAUUACUACCUUAGGGUAAUAGUCCUCAN  >Ss-AA_clean.1_(paired)_contig_572  ACAAGUCAAAACUCCUCUAAUCACGCCGGCAUCUCCAUCAUGCAUCCGCAGACGGAGAAUACCGGGAAUGAUCGAGGGGAGCCCCCUGACAAUGGAAAUUGGCAUCUCGUUAGUAACGAGAACCGGAUUUCCCGACACGAAGUGCUGAACGAUCCGGUAGCACUCUUUUAGAUACAAAACAGUGAACUUACGUCCAUUGUGAGUAAUAAGAGAGCGCACCCGGACCGCGAACUUCAUGAAGUUACGCUUGUCUAACUGAAGUGCUCAUAUCAAGAGUCGGAAAUAUUGAAAGAAUAGAAAAAGAUUCAAUUCUUUAAUAGGACCAACUUUGCUAAGACGACCCGCCUCUUGUUUCCAAGAGGAAUGGUUAGUUUUAAAUUGAGUUUUCAUAAAAUUUAAAGAAUUGUUGCACAUUCCUACGUCCAACAAAGGACAUGCGCUUAGACGCGCCCUCGGAAUGCUCAGCGCCAAGUGGCCGGCGUCAAAUACACGAAGUCUUUGAGCAGUGCCCGCCUGACCUGCGACAACUUAAAACUCUGAAAACUCAACCGAAGCUAAUCCUUACCGGAUUGAAU  >Ss-AA_clean.1_(paired)_contig_8104  CUGAACAAUCCGGUAACACUCUGUCAAGUACAACACAGUGAACUGACGUCCAUUGUGAGUAAUAAGAGAACGUAUGCGGACCGCAAACUUCAUGAAGUUAAGCUUAUCCAACUGUAGUGCUCAUAUCAAAAGUCUGAAAUACUGAAAGAAACGAAACAAGUUCAGUUCUCUAGUAGCACAAACUUUGCUAAGACGACACGCCUAUUGUUUCCAAGAGGAAUGGUUA  >Ss-AA_clean.1_(paired)_contig_123  AAUAUUGGAAGAACAGGAAAAGAUCCAGUUCUUUAAUAGGACCGACUUUGCUAAGACGACCCGCCUCUUGUUUCCAAGAGGAAUGGUUAGUUUUAAAUUGAGUUUUCAUGAAAUUUAAAGAUUUGUUGCACAUUCCUAUGUCCAAAAGGACCUGCGCUUAGACGCGCCCUCGGAAUGCUCAGCGUCAACUGGCCGGCGUCAAACACACUAAGUGUUUGAGCGGUGCCCGGUGGACCUGCGACAACUUAAAACUCUGAAAACUCAACCAAGCUAAUCCUUACCGGAUUGAAAGCCCGGUCGAGGUCUCAGGCUUCAGCACACCCGAAAGUGCUUGUGACUUCGUCAAAGACGAUCACCGCUGUUUGGGGAUAAGCUUUGCUCCAACCAAAACUGGUGCCAAUCGGCACCCCGCACGUCCGGAUAGACGUAAGGGAUACUCCCUGGAUC  >Ss-AA_clean.1_(paired)_contig_74  GAUAGACAGGAUCUGAACCUGUAAAUCAACCGGUAAACGGUCCGUGGCAGCACUUAGAUCAUAAUAGUAAAAAUUCUGAUCUUCCAACUUAUCUUCCCUCUUCAAUUGACGAAGACGGUCCAGGGGGGCACUUUGAUUAAAAGUACCAUCCUGGGGAACCCGUCGGAGACAAUCGAAAAUAGCCGCUGACAGAGGUGCCAUUACAGACUGCGUAACAGCGUCUGUGAUGGCGAACACUCUGACUUUCCCCGCAGCCUCCUCCUUAAAAGACAACCGACCAAGGUGUAAAGCCUUGGAAGGAAGGUCUCGUAAGACUGAGACUUCGGAAAGAAGUCCUAACCUAAAAUCCUCACCACCAGGGAAGAGUUUGAUAAAAUCAAGCAAAGCCCCAAAGUUUGGACGAGAUGACCAGGCCUUGAUAUCUAACCAGAUACCUAGACCGGCCACCUUACAGUUAGGUCCCGCGGAUCGGGAGAACAACCAACGGAUAGGUCGAAGCUCCCCUAAAGCAGGGAGAGACGACUUAACCGCCAAAAGUUCAUAAAUCGGAAGAGUAGAAGAUAACCCAGUGAAGGGACCAACAAUAGUGUCGAAUUUAACGUCACCGGGAAACUUUAUCACUCGGAAAACCGAGAGACAAGUUAAGACUCCUCUAAUCACACCAGGAUCACCGUCGUGCAUACGCAAACGGAGAACACCGGGAAUGAUCGAGGGGAGUCCCCUGACAAUGGAAAGCGGUAACUCGUUAGUAACGAGCACCGGAUUUCCCGACACGAAGUGCUGAACGAUCCGAUAACAUUCCUUUAAGUACAACACAGUGAACUUACGUCCAUUGUGAGUCAAAAGGGAACGCACCCGGACCGCGAACUUCAUGAAGUUACGCUUAUCCAACUGAAGUGCUCAGAUCAAAAGUCGGAAGUACUGGAAGAAGAGGAAUAAAUUCAACUCCUUCACAGGCCCAACUUUGCUAAGACGACCCGCUUCAUGUUUCCAUGAAGAGAAAUUAGUUUUAAAUUGAUUUUUCAUGAAAUUUAUAAGAAUUGUUGCACAGUCCUACGGUUCUAAUGAACCCUGCGUUUAAUACACGCCCUCAGACUGCUCAGCGCAUAGUAGCCUGCGUCUAACACUCGAAGUGUUAGAGCGGUGCCCGCUAUACCUGUAACAACUUAAAACUCAGAGAAAUCAACCAAACGCUAAUCCUACUGGACUAGAUCCGGUCGAGGUCUCAGGCUCUAGCACCCCAAAUGGUGUUUGUGACUUCGUCAGAGA  >Ss-AA_clean.1_(paired)_contig_1060  AGGAAAGACGGCCAAGGUGUAACACCCUGGUAGUCAGGUCCUUAAGGGCCGAGGCCUCCUUCAGUAGGUCCUUCUUGAAAUCCUCUCCACCCGGGAAAAGCUUGAUAAAAUCAAGCAAAUCCCCGUAGUUUGGACGAGAUGACCAGGCCCUGAUAUCUAACCAGAUACCCAGACCGGCCACCUUACAAUUAGGACCCGCUGAACGAGAGAAGAGUCAACGGAUAGGUCCCAACUCCCCCAAACAGGGGAGGGAAGACUUAACCGCUAACAAUUCGUAAAUUGGAAGGGUUGAAGAUAACCCAGUGAAGGGACCCACUAUAGUG  >Ss-AA_clean.1_(paired)_contig_593  CGGUCAGUCAUAACAGACUUCCAGAGAACACCGAACCGCUCUCCCAAAUAGAUGGACAGGAUUUGCACCUGACGAUCUACUGGAAGACGGUCCGUGGCAGCACUUAGAUCAUAAGAGUAAAACUUCUGACCCUUCAAUUUACCAUCCCUCUUUAAAGUUCGCAACCGGUCCAAAGGGGCACUUUGAUUAAAAGUACCAUCCUGAGGGACCUGUCGCAGAAGGUUAAAGAUCGCGGAAGAUAAGGGAGCCAUCACCGACUGCGUUACCGCGUCGGUUAUGGCAAAGACCCUAACCUUCCCGGCGGCCUCUUCCUUAAAGGAAAGACGGCCAAGGUGUGAAACCUUGGUAGUCAGAUCCUUAAGGGCCGAGGCCUCCUUAAGUAGGUCCUUCUUGAAAUCCUCCCCAGCCGGG  >Ss-AA_clean.1_(paired)_contig_35  CGGAAAUCAGACGGAUAUGAUGAGAACCCGACAGGAUAAUAGAGUUCAGAUAUCGAAAUCUGGAACUCUAUUGCCUUACGGACUGCAGUUUGCCAGGUACGAACAUCAAAACGAUGUUUAACCCGACGAACUGCAUCAAUAAUCUGAUUCGCUCUAACUGGAGUCAGCGAGCUAGACAUCGUCAAAAACGAUGCUAGCCCUACUUUAGUUGGAACGAAACCGAAAGGUCCUUUGAUGGUUCAAAGAAUAUUGUCCAACAUGCUCUUACGAACAGUUGGAACUUUACUAAUUAAACUAUCCACAGAUUCCUCAGUGAAAGUAACACCUUUACCGCUAAGGUCCCGUAAUAGAGAUGGAACUCCAUUAAGGGACUGCAAAGCGACUAAGGCAUUACUCGCACCCAAAGGUGUAAGCUCAACAUCAUCACGGACUAAUCUCUUAGCAAACUCAAAAGAAUGCUCUGAGACCAAAGUCUUAGAUAAAUUGAUCUUAACCCCGAGGAUAUCAACCAUUAUAGAAUGGUAGGAUCUUGCGACCAGAUCGUUAGCAAUAACAACAUCAUCUCCUAGCACGGCAUAGUCACCAAAGUUAGGUAUAGAAACCCGACGGGCGGCUAGACGUACUACAAAAUGAUGGGUUAAAGCCAACAUACCCCACGAACUUAGGGCUCCCAUGGGCUGACCAACGGAGUAACGAAACUGUUCUUCCUCAGCAGGAAGACCAGGAACCGUCACUACUUUGGCCAUCCAAUCUCUGUCAGUCAUAAUUGACUUUCAAAGAGUACCGAACCGUUCACCAAAAUAGAUGGACAGGAUUUGCACCUGAAGAUCUACUGGUAAGCGGUCCGUGGCAGCACUAAGAUCGUAGGAGUAAAACUUCUGACCUUCUAACUUACCCUCGCGUUUCAGUUGUCUCAACCGGUCCAAGGGGGCACUUUGAUUAAAAGUACCAUCCUGGGGAACCCGACGAAGACAAUCGAAAAUAGCGGUUGAAAGAGGAGCCAUCACCGACUGCGUAACCGCAUCGGCAAUAGCGAACACUCUAACUUUACCAGCGGCCUCUUCUUUAAAGGAUAGACGACCAAGAUAUAAAACCUUAGUAGUCAAAUCCCGUAAAGACUGGACCUCAGAUAAAAGACUCAACUGAAACUCUUCACCACCAGGGAAAAACUUAACAAAUUCAAGUAAAUCCCCGAAGUUAGAGCGAGAUGACCAGGCCUUGAUGUCUAACCAGAUACCAAGACCGGCCACCUUACAGUUGGGUCCAGCCGAACGAGAGAACAAUCAACGGAUAGAUCCCAAACUCCCUAAGGAUGGGAGUGAGGACUUAACCGCCAAAAGUUCGUAAAUUGGAAGAGUAGAAGAUAACCCAGUGAAGGGACCCACUAUCGUAUCGAAGCUUGCGUUACCGGGGAACUUAAUAACUCGGAAAACCGAGAUACAAGUUAAAACUCCCCUAAUCACACCGGUAUCUCCGUCAUGCAUACGCAGACGAAGAGCCCCGGGAAUGAUCGAGGGAAGCCCCCUAACAAUGGAAAUAGGUAACNNNNNNNNNNNNNNNNNNNNNNNNNNNNNNNNNNNNNNNNNNNNNNNNNNNNNNNNNNNNNNNNNNNNNNNNNNNUUUAGUAGGACCAAUUUUGCUAAGACGACCCGCCUCUUGUUUCCAAGAGGAGUUAUUAGUUUUAAAUUGAAUUUUCAUGAAAUUAAUAAGAUUUGUUGCACACUUCUACGACUAUAAAGUCCUGCGUUUAUACACGCCCUUGAAGUGCUCAGCGCCCGGUGGCCUGCGUCACACACACGAAGUGUGGGAUCGGUGCCCGCCUGACCUGUAACAACUUAAAACUCAGAAAACUCAACCAAACACUAAUCCUAUCGGACUAGAAAUCCGGUCGAGGUCUCAGGCUCCAGCACACUGUCAAACAGUGCUUGUGACUUCGUCGUCAGACGAUCACCGCUGUUUGGGGAUGAGCUUUGCUCCAACCAAAACUGGCACCUAAAGGUGCCCUGCACGUCCAGAAGACGUAAAGGGGGAACCCUUGGAUCAGCUUCG  >Ss-AA_clean.1_(paired)_contig_23  CUUCUCGCUCUGCAGUCACUUAAUGGAGUUCCAUCUCUAUUACGUGACUUGAACGGGAAGGGUUUUACUUUCACCGAGGACUCGGUUGAUGAUUUAAUGAGUCGUGUUCCAACGGUUCGUAAGAGCCAGUUGGAGACGAUUCUUUGAACCAUCAAAGGACCUUUCGGUUUUGUUCCGACUCGAGUAGGACUAGCAUCGUUUUUGACGAUGUCUAGUUCGCUGACUCCAGUCCGAGCAAAUCAGAUUAUUGACGCUGUUCGCCGAGUUAAACAUCAGUUUGAUGUUUCUACUUGGAAGACAGCGGUCCGAAAGGCAAUAGAGUUCCAGAUUUCUAUAUCUGAACUUUAUUACCCUGUCGGGUUCUCGUCAUAUCCGUCUGGUUUCGAAAACUCUCCACCCACCCCCCCCCCCCCCCCCCCCCCCCCCCCCCCCCCCCCCCCCCCCCCCCCCCCCCCCCCCCCCCCCCCCCCCCCCCCCCCACGCGCCCUCC  >Ss-AA_clean.1_(paired)_contig_22  GUUAAACAUCGUUUUGAUGUUCUAACUUGGCAAACAGCUGUCCGAAAGGCAAUAGAGUUUCAGAUUUCUAUAUCCGAACUCUAUUACCCUGUCGGGUUCUCGUCAUAUCCGUCUGGUUUUGAGAACUCUCCGGUCCGGAGAGCCAUCAUACACCAGAACUCCCUCGACCUUCAAUCCCUUGGAGCAUCUGCUCCUCGGUGACGGUUGGUCUUUGGAGGUCCCUUAAUAAUGAUGGAUUAUUAUAGGGCGAGCUAUGAGCAAGAGAUUGUCGCAUACAUCAAGGAAUUGAUCUGUGAUCAAUCCU  >FirsU_Contig6  GAAACUUCAAAGGAUUGAUCAAUGAUCAACUCUUUGAUGUAUGCGACAAUCUCUUGCUCANUAGCUCGCCCUAUAAUAAUCCAUCAUUAUUAAGGGACCUCCGAAAACCAACCGUCACCGANGGAGCAGAUGCUCCGAGGGAUUGAAGGUCAAGGGAGUUCUGGUGUACGAUCGCUCUUCGANACCGAAGAGUUUUCGAAACCAGACGGAUAUGACGAGAACCCGACAGGGUAAUAAAGUUCANGAUAUAGAAAUCUGGAACUCUAUUGCCUUUCGGACCGCUGUCUUCCAAGUGUUAACAUCANAACUGAUGUUUAACUCUUCGAACAGCGUCAAUAAUCUGAUUUGCUCGGACUGGAGUUAGCNGAACUAGACAUCGUCAAAAACGAUGCUAGUCCUACUCGAGUCGGAACAAAGCCAAAAGGUNCCUUUGAUGGUUCAAAGAAUCGUCUCUAACUGGCUCUUUCGAACAGUUGGAACACGACUANAUCAAAUCAUCAACCGAGUCCUCGGUGAAAGUAACACCCUUCCCACUCAAGUCACGUAAUNAGGGAUGGAAUUCCAUUAAGUGACUGAAGAGCGAGAAGUGCAUUACUUGCACCCAAGGGUNGUUAGUUCAACGUCCUUUGAAACUAAUCUCUUAGCAAACUCAAAAGAGUGCUCAGAGACUNAGUGUCUUAGAAAGGUUGAUCUUAACUCCAAGGAUGUCAACCAUGAUACUAUGGUAGGAUNCUUGCGACCAGAUCAUUAGCGAUAACAACAUCAUCACCUAGAACAGCAUAGUCCCCGAAGNUCGUGUAAAGACACGCGACGGGCGGCUAGUCGAACUACGUAAUGGUGUGUUAAAGCUAACNAUCCCCCAUGAACUAAGGGCCCCCAUGGGCUGACCAACAGUAUAGCGGAACUGUUCUUCCNUCAGCAGGAAGACCAGGGACCGUUACAACUUUGGCAGACCAAUCUCGGUCGGUCAUUACUNGAUCGCCAGAGAGUACCGAACUGUUCACCAAGGUAGAUAGACAGGAUUUGCACCUGUAGANUCCACCGGUAAACGGUCCGUGGCGGCACUUAGAUCAUAAGAGUAAAACUUCUGACCUUCCAACUUACCUUCCCUCUUCAAUUGACGAAAGACGGUCCAGGGGGGCACUUUGAUUAAAAGUACCAUCCUGGGGAAUCCGUCGAAGACAAUUCAAAGAUCGCCGCGGAAAGAGGAGCCAUCACAGACUGCGUUACCGCAUCUGUUAUAGCAAAACACUCUUAUUUUCCCUGCCGCCUCUUCCUUAAAAGACAACCGACCAAGGUGUAAAGCCUUUGGAAGGGAGGUCUCGUAGGACAGAGACUUCAGAGAGAAGUCCUAAUCUGAAAUCCUCACCCACCGGGGAAGAGCUUGAUAAAAUCAAGCAAGGCCCCGAAGUUAGGACGAGAUGACCAGGGCCUUGAUAUCUAACCAGAUACCUAGACCGGCCACCUUACAGUUAGGUCCCGCAGAACGGGGAGAACAAUCAGCGAAUAGGUCGGAGCUCCCCCAAAGGAGGGAGAGACGACUUAACAGCCAAAAAGUUCGUAAAUUGGAAGGGUAGAAGAUAACNCCAGUGAAGGGACCCACAAUAGUGUCGAAUUUAACGUCACCGGGGAACUUAAUCACUCGGNAAAACCGAGAGACAAGUUAAGACUCCUCUGAUUACACCGGAGUCACCGUCAUGCAUACGCNAAACGGAGGACACCGGGAAUGAUCGAGGGGAGCCCCCUGACAAUGGAAAGCGGCAUCUCGNUUUGUAACGAGAACCGGGUUUCCCGACACGAAGUGCUGAACGAUCCGGUAACAUUCUUUUNAAGUACAACACAGUGAACUUACGUCCAUUGUGGGUCAAAAGAGAACGUAUCCGGACCGCGNAACUUCAUGAAGUUACGCUUAUCCAACUGAAGUGCUCAGAUCAAAAGUCGGAAGUAUUGGNAAGAAGAGGAAUAAGUUCAACUCCUUCACAGGUCCAACUUUGCUAAGACGACCCGCUUCANUGUUUCCAUGAAGAGAAAUUAGUUUN  >FirsU_Contig2  CCCUCCGAUGGAAACUUCAAAGGAUUGAUCACAGAUCAGUUCUUUGAUGUAUGCGACAAUNCUCUUGCUCAUAGCUCGCCCUAUAAUAAUCCAUCAUUAUUAAGGGACCUCCGAAGACCAANCCGUCACCUAGGAGCAGAUGCUCCGAGGGACUGAAGGUCAAGGGAGUUCUGGUGUACGAUNAGCUCUUCGAACAGAGGAGUUCUCGAAACCAGACGGAUAUGACGAGAACCCGACAGGGUANAUAAAGUUCAGAUAUAGAAAUCUGAAACUCUAUUGCCUUUCGGACCGCCGUUUUCCAAGUNGUUAACAUCAAACUGGUGUUUAACUCUUCGAACGGCGUCAAUAAUCUGAUUUGCUCGGACNUGGAGUCAGCGAACUAGACAUCGUCAAAAACGAUGCUAGUCCUACUCGAGUCGGAACAAANGCCAAAAGGUCCUUUGAUGGUUCAAAGAAUCGUCUCCAACUUGCUCUUACGAACAGUUGGNAACACGACUAAUUAAAUCAUCAACCGAGUCCUCGGUGAAAGUAACACCCUUCCCGUUAAGNGUCACGUAAUAGAGAUGGAACUCCAUUAAGUGACUGCAAAGCGAGAAGUGCAUUACUUGCNACCCAAGGGUGUUAGUUCAACGUCUUUCGAAACUAAUCUCUUCGCAAACUCAAACGAGUGNCUCAGAGACUAGAGUCUUAGACAAGUUGAUCUUAACUCCAAGGAUGUCAACCAUGAUACUNAUGGUAGGAUCUUGCGNACCAGAUCAUUAGCAAUAACAACAUCAUCACCUAGAACGGCGUAGUCACCAAAAUCGUGUAAAAGAGACACGACGGGCGGCCAACCGAACUACANUAAUGGUGCGUUAAAGCUAACAUACCCNCAUGAACUAAGGGCUCCCAUGGGCUGACCAACGNGUAUAACGGAACUGCUCUUCCUCAGCANGGAAGACCAGGAACCGUGAUAACUUUGGCAGACNCAAUCCCUGUCGGUCAUAACUGACCGUNCAGAGAGUACCGAACUGUUCACCAAGGUAGAUANGACAGGAUCUGAACCUGUAAAUCAACCNGGUAAACGGUCCGUGGCAGCACUUAGAUCAUAAUGAGUAAAACUUCUGACCUUCCAACUUACCUUCCCUCUUCAAUUGACGAAGACGGUCCAGGNGGGGCACUUUGAUUAAAAGUACCAUCCUGGGGAACCCGUCGAAGACAAUCAAAGAUCGCCNGCGGAAAGAGGAGCCAUUACAGACUGCGUUACCGCAUCUGUUAUAGN  >FirsU_Contig38  NAUCCAAGGUUUCCCUUUACGUNCUUUCCGGACGUGCGGGGCGCUUUGGGCGCCAGUUUUGGUUGGAGCAAAGCUCAUCCCCANAAUAGCGGUGAUCGUCUUUGACGAAGUCACAAACACUAUUAAGUGUGCUAGAGCCUGAGANCCUCGACCGGAUCUAAUCCAGUAGGAUUAGUGUUUGGUUGAUUUCUCUGAGUUUUAAGUUNGUUACAGGUAUAGCGUGCACCGAUCUUACAUUUAGUAUGUAAGACGCAGGCUACUAUGCGNCUGAGCAGUCCAAGGGCGUGUAUUAAACGCAGGGUUCGUUAGAACCGUAGGGCUGUGCAANCAAUUCUUUAAAUUUCAUGAAAAAUCAAUUUAAAACUAAUUUCUCUUCAUGGAAACAUGANAGCGGGUCGUCUUAGCAAAGUCGGACCUGUGAAGGAGUUGAAUUUAUUCCUCUUCUUCCAAGUACUUCCGACUUUUGAUCUGAGCACUCCAGUUAGAUAAGCGUAACUUCAUGAAGUUCGCCGGUCCGGGUACGUUCUCUUUUGACUCACAAUGGACGUAAGUUCACUGUGUUGUACUUANAAAGAAUGUUACCGGAUCGUUCAGCACUUCGUGUCGGGAAAUCCGGUCCUCGUUACUAACNGAGUUACCGCUUUCCAUUGUCAGGGGACUCCCCUCGAUCAUUCCCGGUGUCCUCCGUUUGNCGUAUGCAUGACGGUGACCCCGGUGUGAUUAGAGGAGUCUUAACUUGUCUCUCGGUUUUCNCGAGUGAUCAAGUUCCCCGGUGACGUUAAAUUCGACACUAUUGUGGGUCCCUUCACUGGGNUUAUCUUCUACUCUUCCGAUUUACGAACUUUUGGCGGUUAAGUCGUCUCUCCCCCCUUUANGGGGAGCUUCGACCUAUUCGUUGAUUGUUCUCUCGUUCUGCAGGACCCAAUUGUAAGGUGNGCUGGUCUUGGUAUCUGGUUAGAUAUCAAGGCCUGGUCAUCUCGUCCUAACUUCGGGGCUNUUGCUUGAUUUCAUCAAGCUUUUCCCGGGUGGUGAGGACUUUAGAUUGGAUCUUCUCUCUNGAGGUUUCUUCUUUACGGGACCUUCCUACCAAGGUUUUGCACCUGGGUCGGUUGUCCUUUNAAAGAGGAAGCUGCAGGGAAGGUAAGGGUCUUUGCUAUCACAGAUGCGGUUACGCAGUCUNGUCAUGGCACCCCUUUCUACUGCAAUCUUCGAUUGUCUAAGACGGGUUCCUCAGGAUGGUNACUUUUAAUCAAAGUGCCCCGUUGGACCGUCUCCGUCAACUGAAGAGAGAGGGUAAAUUGNGAAGGUCAGAAGUUUGACUCCUAUGACCUUAGUGCAGCCACCGAUCGUCUUCCN  >FirsU_Contig28  CAAAAUUGGUCCUACUAAAGAACUGAACUUGUUUCUUUUCUUUCAGUAUUUCCGACUUUUNGAUAUGAGCACUCCAGUUGGAUAAGCGUAACUUCAUGAAGNUUUGCGGUCCGGAUACGCUCNUCUUAUUACUCACAAUGGACGUAAGUUCACUGUUCUGUACNCUAAAAGAGCGUUAUCGGAUNCGUUCAGCACUUCGUGUCGGGAAAUCCGGUACUCGUCACUNGACGAGUUACCUAUUUCCAUNUGUCCGGGGGCUCCCCUCGAUCAUUCCCGGUGUCCUUCGUNCUGCGAAUGCACGACGGUGACGACUGGUGUGAUUAGAGGAGUCUUAACUUGUAUUUCGGUU  >FirsU_Contig62  UCAAGGAGCCUCGCUUUAGCGGGUAUGACACCCCGCUGGAGAGCCUUAAAGAGGUAGCGCNCCGAUGGCGCAGUUCUUCUCUCACCUNCUUCAAAGAAAAUCUUUCCUUUGAAGCGAGAUGANGAAGGCGAAAUCCUCGAAAGUCUGUCNGCACGAAGGGGUCACCCCCCCCGAUGGAUACCUCNAAAAGACUGAUCAGAGAUCAGCUCUUNUGAUGUAAGCGACAAUCUCUUGCUCAUAACUCGCNCCGAUAAUAAUCCAUCAUUAUUAAGGNGACCUCCAAAGACCAACCGUCACCUUGGAGCAGANUGCUCCUAGGGAUUGUAAGUCUAAAGNAGUUCUGACUAACGAUCGCCUCCCGAACGGGAGANUAAUCGAAAAUCAGACGGGUAUGACGNAGAAACCUGUAGGGACAUAAAGUUCAGAUAUAGANAAUCUGGAACUCUAUUGCGUUACGGANCUGCAGUUUGCCAGGUACGAACAUCAAAACGAUGNUUUAACCCGACGAACUGCAUCAAUAA  >FirsU_Contig19  AUUAGCUAUAACAACAUCAUCUCCUAGUACAGCAUAGUCACCAAAGUCGGGUAACGAAACNCCGACGGGCGGCUAGCCGCACUACGAAGUGAUGGGUUAGAGCUAACAUACCCCAUGAACUNAAGUGCUCCCAUAGGCUGACCAACUUGAUAACGNGAACUGUUCUUCCUCAGCAGGAAGACCGAGGAACCGUGACCACCUUGGCCAUCCAAUCUCGNGUCAGUCAUAACAGACUUCCAGAGAACNACCGAACCGCUCUCCCAAAUAGAUGGACAGGAUNUUGCACCUGAAGAUCUACGGGAAGACGGUACN  >Ss-AA_clean.1_(paired)_contig_69  CUGGGGGGUUUCGGGCCAAAGCCCGAAUACUCCAGCACCCCAGGUUUCCGAAAGGAAGCCUUUUCUGCUAGAGCCGCAUCAGUUACCUGAUGAGGGUCAUGAAUACCCAAACUUUUUAUUGCUAAAGGUUUGUUCAGUACUCAAGUCUACAGGACACGUAAAAGUCGGUGGAUUCUUAUUAUUUACAGUUAUAAUAUCGAUUCCCAAAUCUUCGGGAACUCGCUAUGAUAUCUUUUACUAAUAAGACCUGACUUGAUAUUAGCCUCUUUAUCCCAUCUCAAUGCAUCAAAACGUGAUGAAUCGAGAGAAGAUAAGUACCGCAUUACUGCGGAAGUACCACCAACAGAAUAUAUCUCCGUAAAGAGAGUAUGUUCUAGAGUGUACUUAGCUGUAUCAUACCUGUCCACAGUUUUGAUAAACUGUAGGAUGUAGAAUCAGAAUCCGGGAGAGAAAAUAGAAAAUAUUGUAUCAAAUACAAUAUAUCUAGGAUCUCUAACCAUAGAUGAUUUAUAGAAAUUCUUAAGAAAGAACAUAAAGUCUUUUUUAGGAGUAACAGAUGAAAAUUGCUCAGUAGCAAUUUUAACUGAUACAUCUAUAAUAUCAUAAUGGUUUGCUUCUCCACUAUUUCAUAAAGCCAUUGAAUACAUGUCUAUCGCAAAAGCGUUCGACAAGUUGGGCUUUUCUCUAAGAACCUUAGAGAAGAAAUUUCAAAGCACUAAUUCACUCUUCACCUUGAUAAAACGAGGGGAAGAGGAACAAAUGCUAACAACGUCACAAAAGUUGAUGAUCUUUACGGAAAUAGCCUCUCUUAAGACAGAAGAGACUAUGGUCCUAUUCCUGAUGGACUGGAGAAUUAACCCAGGCCCAAAAGGCGUGUAAUCUAGAUCUGGCCCAAUUAAUCGCUUAGCAAACUCCAUGAACUCGAAAGAAGUUAUGGAUUUGUAAGGAUUAAUGGACAGACCUAGAUCGUUCAUCAAAUCAGUAUAAUGGGAAGACACCUGAUCAUUCCUGAUCACGAUAUCAUCCCCAAGUACCGCGUAAUCAUCAAAGAAACCAACAAACCCUGAACGGGCAGCGGCUUCCUGGACAAUCACAUGAUGUGUCAUUGCCAGCAUCCCUCAAGAAGAAUAGGCACCCAUGGGCUGUCCUACUGAAUAUUUAAUUCGUUUAAAAUGUUCAGGAACAUACCAUGAGAUGUCAAGAAGAUCACGCCAGACCUCCCCAGGAAACCCAUAGUGAGACAGAAUGUCACACUGGAUAUCCACGGGAAGUCUGUCGGUAGCCGCAGUUAGAUCAAAACAGUGAAAGGCAACUGGGUCUUUCCCUAGCUCUUUAUCUGAGAGCGCCCUCUCAACGAGACGUUGAGCGGGAGCAAUCUGAUCAAAAGUACCAUCAGUAGGAAUUUUCUUCAAAAUAGAAAACAACUUACGAUGUAAAGGGUUCAGACACAGCUGCAACCACCAGUUAGUUAUUGCAACUACUCUGGCUUUCCCUGCCUGAUCUCGAACUACGGAUAAUCUUCCUUCUUCUAAUCGAGAGGAUGUUGUGAAACUUAUGAUCAAGUAGAGAGGCCCGAAUAAGAACCAGAUAUUUAGCAAUCAUAUCAAUCAAAAUCAAGAUUUUGAUUGUACUAAAUACCGAAGACAAGGGAGAGGAUGGUGCAACAAUGCCAAGGCAUCAAGCAACGAUCCUCACGCCGCCUUCUUCGAAUUCGGCCCAGAAGACUCACCACCUAUGAUAAACAUAGGUAGAGAGGCGAUAUUGAUCUUACCUUUCCAUAACCUACUAGCCGCCACCCUCACGUCAAAUGAACGACAAACUCCACUAAAAGGGAGUUCGAUCGUAUCAUAUGAAACGACGGGAGACGUAGCAAGCAGACGGAAAACAGAUAAUAACGUCAGGAUAAAUCUAAUUUCUUUCUGAGAAGAUGCUCCACUACGAAUGUAGCGACGCAUCCCACCGGGAAUAAUCUUAGGUAAUCCACUUUUGUCAACUCUAACAAAUAUCUUACGACACUUGUCUUGUUGACCAGCCAGAGAUCUAGUAACCAAUCUAAAACAUUCCUUAAGAUAAAGGAAUGUAAAAUUGGAACCAGACUUGCUGAUGAGGGUUAAAACCCUUUCAGCAAACCGCUUAACGUGUGACCUGUGUAUUGAAGUACUGAAUAUUCAUGAAGCCACAAGGAAAUACAGAUGAAUCUCCUUUGUAGAGAUUCAGUCCUUAACUCCUUGAGGCUUUUUAUUUAAAUAGGAAAAAUAUUUUACAAUAUUCUUCUUGUUUAGAUUAAAUAUGAACUUCAUGUAUAAAUCAAUAUAUGUUGCACACACCGUUGUGUUUAUCCGUAAAGGACAAACAAUACCCAUUCAGGCUGGUCUGCGAUGUGCUCAGCGCAUAGUGGCCUGCGUUAUAUACACUAAGUAUAUAAUCGGUGCCCGCUACACCUGACUGAUGAACCCCUCGAAUGAGGUGAACAUACACGAAAUUAGCAAACUUAUUGCUAUAGGACGUCUCAUGAUUCAAAGAAUCACUGGGUAUCACGACCAUAAUGGCCGAGUACCAGAGUUGGGGCGUACGUCCGUACGAUCACACGGGGUGUGACGGA  >Ss-AA_clean.1_(paired)_contig_148  CGGACCUACGUCCGAAUACUCCAGCACCCCACAAGCGAAAGCUUGAUUUCUGCUGAAGCCGCGUCAGUUACCUGACGAGGGUCUUGGAUAUCCAUAUCAUAUUUUCAUAUGAUAUGUUCAGUACCCAAGUAUACAGGAUACAUCCGAUAAGGGGAUUCUCAUUAUACACAGCUAUAAUAUCGAUUCCCAGAUUUUAGGGAACUCGCUAUUAUACCUUUUACUAAUGAGAUCUGACUUGAUGUUAGCUUCCUUAUCUCAUCUUAAUGCAUCAAAACGUGAAGCAUCAAGAGAUGAUAAGUAACGCAUUACUGCGGAAGUUCCUCCUCGAGAGUACACCUCGGAAAAGAGAGUAUUCUCAAGAACGAACUUUGCUUCAUCAUACCUGUCUACAGUCUUGAUAAACUGUAAGAUGUAAAAUCAGAAUCCGGGAGAGAAAAUAGAGAAUAUUGUAUCAAAUACAAUAUACCUAGGAUCUCUAACCAUAGAUGAUUUAUAGAAGUUCUUAAUAAAGUAGAGAAAAUCUUUACUAGGAUUAAGAGAUGAAAAUUGCUCACGAGCAAUUUUAAUCGAAACUUCUAUAAUAUCAUAAUGGUUUGCUUCACCACUAUUUCAUAAAGCCAUUGAAUACAUGUCUAUCGCGAAAGCGUUCGACAAGUUUGGCUUUUCUCUAAUAACUUUAGAGAAGAAAUUUCAAAGCACAUGUUCCAUCUUCACCUUUAUAAAACGAGGUGAGGAUGAGCAUAUGCUAACAACGUCACAAAAGUUGAUGAUUUUAACGGAUAUGGCCUCUCUUAAAACAGAAGAGACGAUAUUCCUAUUCCUGAUGGACUGGAGAAUUAAUCCAGGUCCAAAAGGCGUGUAAUCUAGAUUCGGACCCAUUAAUCUCUUAGCAAACUCCAUGAACUCAGAAGAGUUGAUGGACUUGUAAGGAUUAAUAUCCAAGCCUAGAUCGUUCAUCAACCUGGUGUAGGAUCUGGCAACGUCAUCAUUCCUGAUAACGAUGUCAUCUCCUAAAACAGCAUAAUCGUCGAAGAAACCGACUAACCCUGAAAGGGCAGCGGCUUCCUGAACGAUCACAUGAUGUGUCAUUGCCAACAUCGCCCACGAAGAGUAGGCACCCAUUGGUUGCCCAACCGAAUACUUGAUUCUUGUCAUGUGUUCGGGGACAUACCAAUUGAUGUCGAGUAGAUCGCGCCAGACCUCCCCUGGAAAACCAAGUGAUUUAAGAAUAUCAACCUGGAGAUCCACUGGGAGUCUGUCGGUAGCGGCGGUUAAAUCAAAGCAAUGGAAGACACCUUUAUCUUUCCCCAGCUCUCGAUCAUCAAGCUCCCUUUCAACCAAACGUUGAGCAGGAGCGAGUUGAUCAAAAGUACCAUCAGUAGGAAUUCUCUUCAAAAUAGAGAAUAACUUCCGAUGUAAAGGGCGAAGGCAAAGUUGUACUCACCAGUUAGUUAUAGCAACUACCCUGGCUUUCCCUGCUUGAUUUAGAACCACCGAUAAUCUACCUUCCUCUAAGCGGGACGAAGUUGUGAAACUUAUGAUAUAGUAGAUCGGCCCGAAUACUAAUCAGAUAGAUAGUAAUCAAAUAAGUCAAAAUCAAGAUUUUGACUUUACUAAAUAUCGAAGAUACGGGAGGGGUCUAUGUAAGGAUGCAACAGCAUCCAACAAAGAUCCUCACGCAGCUUUCUUAGAAUUCGGCCCAGAAGAUUCACCUCCGAUGAUAAACAUCGGAAGAGAAGCGAUAUCUAUUCUACCUUUCCAUAUCCUCUUAACUGCAACUCGAACGUCGAAUGAACGAAU  AGAUCCGCUGAAAGCGGACUCUAUAGAUUCAAAGGACACAAGAGGAGAAGUAGGGAGGACACGGAAAACAGAUAAUAACGUCAGGAUAAAUCUAAUUUCUUUCUGAUGAGACGCUCCAGCACGCAAGUGCUGGCGCAUCGCAGCAGGAAUAAUCUUAGGUAAUCCGCUUUUGUCAAUUCGAACAAAUAUCUUACGACACUUGUCUUGUUGACCGGCCAGGGAUCUGGUAACUAACCUUAAGCACUCCUUAAGAUAAAGGAAUGCAAAGUUAGACCCAGAUUUGCUAAUAAGUAAAAUAAUCUUAUCAGCAAACCGUCGGGUGUGGACCCUGUAUAUUGAAGUACUGAAUAUCCAAGAGGUCACCAGGAAAUACAGAUAGAUUUCCUUUGUAGAAAUCCAAUCCUUAACUCCUGAAGACCUUUUAUUUUUAUACGAGAGAUGUCGAAUGACAGCUCUCUUAAAAGAUAAAAAUCUUGAUUUCAUGUUUCAAUCAAUAUAUUUUGCACACAUCGUUAUGAUAUAGUCCAAGGACUACAUCAGAACCCAUUCAGACUGGUCACCGAUAUGCUCAGCGCAUGGUAGCCGGCGCCAUAUAUACUAAAUAUAUGGGCGGUGCCCGCCACACCUGGUUGACGCACCCUUAUUAAAGGGAACGAAACACGAAAUUGCAAACUUAUUGCAAUAGGACGUCUCAUGAUCGUAAGAUCACUGGGUAUCACGAAAUUAAUCGAGUACCAGAGUUGGGGCCACGUCCGGGACUAUCACACGGGGUGUGACGGUAG  >Ss-AA_clean.1_(paired)_contig_99  CAGAAGUCUAUGUCGGGAGCGUUGAUGGACGGAAAGUAUCACUUGUGGAUUAAACAAAGUUCAGAUUUUAACUCUUUAAAAUCAAUUUUGUUUUUAUCCAUGAUUGGAGUCCUGUUUUACGAGGUCGACUCAGCCUUACAACCCUUUUCUUCGCGGUGAUAGGGACACCGUCCGUAAGUGGGCGAGGCGUGGGGUUCAUAGAACUUCGUAUUAGAAGCUUAUACUGAUUUCCACUGCUGUCGCGUCUUCGGUCAGAUCUGUCCUUGUAUACCAACCAGGAUGUAUUUAGUCCUGCGGAAGCAUACAAGGUCCUCGAGCAGAUUGUUAACAGUUCGCUGCCAUGUCAGUACGAGGCUGCUCCUUCCAAUCCGUUCUCACGCCCUCCUUUGACAGAGGAGGGUGGGUAUACGGGGCAUGGACAGGAGUGUCAGUCACUUACUACCACCCAAACCCAGGGUGUUGUCUUAGUAAGAGACCUCUCGACCUGAAACAGGCUAGACGUAACACGUCAACUAGCUAUUAGCAGGGUCAACUCAAGGCAGUUCAGAUCCGGGGCCGACGCCGUGGUCAUAUAAACCUCUAG  >Ss-AA_clean.1_(paired)_contig_154  UUUCCGUCCUUCAACGCUCCUGACAUCGACUUCUGUAUAGAUCGAUUCGAAAGAAUCUUUCUAACAAGAGGUCGUGUCGCGGCGGCCGAUGAAAUGAAAGCAUCGCGACUGAUCUUAACCCGAUGAAAAUCGGGGCGUCCAUUGACCGGAAAGGUCGGUGCUCCACUUUCAAAGAUCGACCACUUACCAAAAGCUUAUUUAAGCCUAGGUUUACGGCGGUCCUUGAAGAGUGAUCCAGACCUGGUAUAUUUCCGAUGAUGUAUGACCUUGUUGUCAUGCUCACGGAUUAUACUAGGAGGGAAACCCCUUAACUUAAAGUCGAUAACUGAACCGUCUCGGAUGACAUGUCAUCCAGAGGGCUCUGAAAUUGCUUUAGCGCUAAGAAAGUUAGAGCUCCAACCCUUCUCCGAGCGACCUACUUGAACAAAAUUCAAGUGGAUGU  >Ss-AA_clean.1_(paired)_contig_469  UUGGGUGGUAGUAAGUGACUGACACUCCUAUCCUUGCCCCGUAUACCCACCCUCCUCUGUCAAAGGAGGGCGUGAGAGCGGAUUGGACGGAGUAGCCUCGUACUGACAUGGCAACAAACUAUUAGCAAUCUGCUCAAGGACCUUGUACAUCUCCGCAGGACUAAAUACAUCCUGGUUGAUGUGCAAGGACAGAUCUGUCCGAAGACGCGACAGCAGUGGAAAUCAGUAUAAACUUCUAGUAUGAAGUUCUAUGAACCCCGCGUCUCGCCCACUUACGGACGGUGUCCCUAUCACCGCGAAGAAAAGGGUUGUAAGGCUGAGUCGACCUCGUAAAACAGGACUCCAAUCAUGGAUAAAAACAAAAUUGAUUUUAAAAAGUUAAAAUCUGAACUUUGUUUAAUCCACAAAUGAUACUUUCCGUCCAUCAACGCUCCUGACAUUGACUUCUGUAUAGAUCGAUUCGAA  >Ss-AA_clean.1_(paired)_contig_1539  UGCGAAGACUUUGCAGCAAGGGAAGGGGUAAGUUCUGGAGAUCUCUCAAGGAUUGCGCGAUUGAUAGCCCUUUCGGCCCGGCUGAGGACAUCCACUUGAAUUUUGUUCAAGCGGGUCGCUCAGUGAACGGUUGAAGCCCUAGCUUCCUUAAUGCUAAAGCUAUUUCAGAUCCCUCCGGAUGACAUGUCAUUCGAGACGGAUCUGUUAUAGACUUUAGGUUAAGAGGCUUCCCUCCUAAUAUUAUCCGUGAGCAUGACAGAAGUGUCAUGGCCCAUCGGAAGUAUAUUAGAUCGGGAUCAUUCUUCAAAGAUCGUCUCAACUCAAGACUUAAAUAAGCCUUGGGAAGAUGGUCGAUCUUGGAUAAUGGGGCCCCAACCUUUCCGGUCAAUGGAUGCCCCGAUUUUCAUCGGGUUAAAAUCAGUCGCGAUGCUUUCAUUUCAUCAGCCGCUGCGACUCGACCUCUGGUUAGAAAGAUUCUUUCGAAUCUAUCU  >Ss-AA_clean.1_(paired)_contig_174  CCAAGACAUUUAUUACUGACUUUCCUACAAUGAAUAAAACUUUAUUCGGAGUAAGAGAACCGGUAACUAAAUAUCUAAGACGGCUAUCUGUUAAAGCAGAUGCGGAAGCCAAGUCCCGACCAUUUGCGAUCUUAGACUACUGGACUCAGUCAGCCUUAACACCGUUGCAUGAUCGAUUGUACGACAUCUUGAGGUCAAUACCUCAAGAUUGUACUUUCGACCAGCAGAAGGGUGUUGAGAUGAUGAGCCAGUAUGAUAAGUCGCAGAUGUUCUCACUGGACCUAACAGCCGCCACUGAUAGAU  >Ss-AA_clean.1_(paired)_contig_175  GAGAUCUCCAGAACUUACCCCUUCCCUUGCUGCAAAGUCUUCGCGAGAUCGGAGGUGAAGCUUUCGGCUCUUCUGCAGACGCAGCCAUGACAUUUAUCUCGGACUUCCCCACAAUGAAUAAAACUUUAUUCGGAGUGAGGGAACCCGAGACUAAAUAUCUAAGACGGCUAUCUGUUAAAGCAGACGCGGAAGCUAAAUCUCGACCAUUUGCGAUUUUGGACUACUGGACUCAGUCAGCCUUAACACCGUUGCAUGACCAAUUGUACAAGAUCUUGAGGUCAAUUCCUCA  >Ss-AA_clean.1_(paired)_contig_1126  CUUGAGGAAUUGACCUCAAGAUUUUGUACAAUUGGUCAUGCAACGGUGUUAAGGCUGACUGAGUCCAGUAGUCCAGGAUCGCAAAUGGUCGAGAUUUAGCUUCCGCGUCUGCUUUAACAGAUAGCCGUCUUAGAUAUUUAGUCUCGGGUUCCCUCACUCCGAAUAAAGUUUUAUUCAUUGUGGGGAAGUCUGAGAUAAAUGUCUUGGCUGCGU  >Ss-AA_clean.1_(paired)_contig_6259  CGUUGCAUGAUCGACUGUACACUAUCUUGAGGUCAAUACCUCAAGAUUGUACUUUCGACCAGCAGAAGGGUGUAGAGAUGAUGAGCCAGUACGACAAGUCGCAAAUGAUCUCGCUGGACCUCACAGCCGCCACUGAUAGAUUCCCGAUGCUAUUCCAGCAUAAGUUUCUAGAGUGGUUUGCGUGACAGGAAUACGCUGAUAACUGAUCCCGAAUCAGGGUCGGCUACCCUUUCAUCUAUGACGGGACUGAGUUGAGAUUCAACUGUGGUCAACCUCUAGGUGCAAAGAG  >Ss-AA_clean.1_(paired)_contig_176  CAGACGCAGCCAAGACAUUUAUAUCGGACUUCCCCACAAUGAAUAAAACUUUAUUCGGAGUGAGAGAGCCCGAGACUAAAUAUCUAAGACGGCUAUCUGUUAAAGCAGACGCGGAAGCCAAAUCUCGACCAUUUGCGAUCUUGGACUAUUGGACUCAGUCAGCCUUAACACCGUUGCAUGAUCGACUGUANNNNNNNNNNNNNNNNNNNNNNUAUGACAAGUCACAAAUGUUCUCGCUAGACCUAACAGCCGCCACUGACAGAUUCCCUAUGCUUUUCCAGCAUAAGGUUCUGGAGUGGUUUGCGGGACAGGAAUACGCUGACCACUGAUCCCGAAUCAUGGUUGGCUACCCCUUCAUCUACGAGGGAACCGAGUUGAAAUUCAACUGCGGGCAACCUCUAGGUGCAAAGAGUAGUUGAGUCAUGUUCACACUAUCUCACCACGUUGUAAUGGUAAUUGCGGCGAUGAGAACCAACUCAC  >Ss-AA_clean.1_(paired)_contig_397  UAAUGGUAAUUGCGGCGAUGAGAACAAACUCACCGCUUCGCAACUACGUUAUACUUGGUGAUGAUGUUGUGAUAAGGACCAAGCGAUUAGCCUCUGAAUAUAUGAGGAUAAUGGACGGUCUUGGUGUAGACAUAUCUCCUAGCAAAUCUCAUCAAUCCAAAGGAUAUUUUGAGUUUGCUAAGACAUGAAUAUACCGCGGCCGCCCAGCUAGUGGUUUUCCGAUCAAAGGAGUUUACUCUACAAUAGGCUGGAUAUCAGAAUUGAUUCCAGUCCUAGUGGAUAUAGCUCCAAUGAGAGGUUUUCCGCUCCCUUUCUCAGUAGGGAACCUCUGGAAUUUCUCUCAAGACUGAUCUAGGCUGAUGACCGGUUAUAAGCGACACCAGAUUAAUCUGUCGGCGAAAAUAUACCGAUCAUUGCUCUUCAUC  >Ss-AA_clean.1_(paired)_contig_718  UAAUGGUAAUUGCGGCGAUGAGAACAAACUCACCGCUUCGCAACUACGUUAUACUUGGUGAUGAUGUUGUGAUAAGGACCAAGCGAUUAGCCUCUGAAUAUAUGAGGAUAAUGGACGGAUUAGGUGUGGACAUAUCUCCGAGCAAAUCUCAUCAAUCCAAAGGAUAUUUUGAGUUUGCCAAGACAUGAACAUACCGAGGCCGUCCAGCUAGCGGUUUUCCGAUCAAAGGAGUGUAUUGCACAAUAGGCUGGAUAUCAGAAUUGAUACCAGUCCUAGUGGACACUGCUCCAAUGAGAGGUUACCCGCUCCCUUUCUCAGUAGGGAACCUCUGGAAU  >Ss-AA_clean.1_(paired)_contig_11577  GGGGCCGAUCUGACGUAGUCUUUUCGACAUCACUUGGUUAACACCAGAACACUGGUACCAUAUCAAGUAUUCUGUGUGUCAACCAAUGGGUGCCGAUUCCUCAUGAGCCAUGUUGGCUACGACACAUCGUGUGAUUGUACAAGAAGCUGCAGCCCGUUCAGGGUUCGUAGCUUUCUUUGAUGAUUACGCUAUCCUUGGAGACGAAAUUGUCAUUCGUAAUGACUCUG  >Ss-AA_clean.1_(paired)_contig_64  GGGAUCGGGACUACUCCCGAAUACUCCAGCACCCCGGUUUGUUAAACCUUUUAGCAGGAGCCACAUCAGUUACCUGAUGAGGGUCUUGAUUGUCCCCAGUGGAAUUAUCUUCCGCAGGGUUACAGUGCAAUACUCAUAGAGCAUGCUAAGUAGGAUGGUGUUUUAUUAUUGGAAACUAUAAUAUCGAUUCCCAGAUUUUAGGGAACUCUUUAUUAUACUUUUUACUAAUAAGACCUGACUUGAUAUUGGCUACCUUAUCUCAUCUCAAUGCAUCAAAACGUGAUGCAUCGAGAGUAGAUAGGUACCGCAUUACUGCGGCAGUACCUCCUUGG  >Ss-AA_clean.1_(paired)_contig_331  CGAUCUUUUCUUAAGAAUUGAAAAUAAUUUCCGAUGGAGUGGUCUAAGAACCAGUUGUAUUCACCAGUUAGUUAUAGCAACUACUCUGGCUUUUCCUGCUUGAUCACGCACAACAGAUAGUCUCCCUUCUUCCAAACGAGACCGUGUUGUGAAACUAAUGAUCAAGUAAAGAGGCCCGAACAACAAUCAAAUAUUUAGUAAUCAAAUAAGUCAGAAUAAAGAUUUUGACUUUACUAAAUACCGAAGGAUGGGUAGAGGUCGAUGUAACAACGCAAGAGCGUCUAACAACGACCCCCAACCAGCCUUCUUGGAGUUCGGCCCAGCUGACUCACCCCCUAUAAUAAAUAUAGGGAGUUUGGCGAUAUUGAUCUUAUGCUUCCAUAUUCUUCGUACAGCACGACGUGUAUCAAAUGUUUUAAAAAGCCCGGUAAAACCGGACUCAAUAGAACUAUAUGACACA  >Ss-AA_clean.1_(paired)_contig_597  AGUGGAUUUCUGUGAAAGAAAUUCCUCUGUAUUUCUUAGUGGCCUCUUGAUUGUUCAGUACACCUAUUCAUAGAUCACAUGUUAAGCUGUUUGCAACUAGGGUUGUUACCCUAGUUGAGAGAUCUGGUCCUAACUUCACAUUCCUGUAUCUCAAGGAAUGUUUCAGGUUAGUUUCCAGAUUUCUAGCAGGUCAGCCGGAUAAAUGUCGUAAGAUCUUUGUUCGUGCUGACAAAGAUGGGUUGCCGAAGAUCAUUCCUAUCUCAAUGCGUUUACUGUUACGUAGACGCCGUGAGAAUCAGAAAG  >FirsU_Contig18  UGGCAUCAUCCGUCACACCCCGUGUGAUUGGCAAGGACGUUGCCCCAACUCUGGUACUCGNACCUUUAUCGGUCGUGAUACCCAGCUGUUCUUAUGAACAGUGAGACGUCCUAUAGUAAUANAGAUUACUAUUUUCGUAUAGGUUAUUGGAUACACAGUAUUUCCUCUUUGUUUAGAGUUAANUACUUUGCAUCCUUUAACCGUCAACAGGCUUGGCGGGCACCGCUUAUACACUUAGUGUGUNAUGACGCAGGCUACUCGGCGCUGAGCUCAUCGUAGGCCCGUCUGAAGGGCGCUCUCCCCANGAUUCGGAAUGAAUCUGUGUGGGGAAGCGGUGAGUGCAACAUAUCUUGAUAAAUGUAUGANAAAUCAUAAUUUUAUUUUUUAACUCUUGUUUAAACACUGUUGGUACAAUAUCCCCUAGGGNGUUUGUACAAUAGGGUUUCAUCAAGAUUUUAUUAUAAAACUAAAAGGCCUUUAGGAGGUAAAGGAGUGGAUUUCGAUAAAAGAAAUUCCUCUGUAUUUAUUAGUGGCCUCUUGAUUGUUCAAGUACACCUAUUCAUAGAUCACAUGUUAAGCUGUUUGCGACUAGGGUUGUUACCCUANGUUGAGAGAUCUGGUCCUAACUUCACAUUCCUGUAUCUUAAGGAAUGUUUCAGGUUAGUUNUCCAGAUUUCUAGCAGGUCAGCCGGAUAAAUGUCGUAAGAUCUUUGUUCGCGCUGACAAANGAUGGGUUGCCGAAGAUCAUUCCUAUCNUCAAUGCGUUUACUGUUACGUAAACGCCGUGAGNAAUCAGAAAGACAUUCGGUUCAUCCUGNACGUUAUUAUCUGUCUUCCGUGUUCUUCCCACAACACCUGUUGUGUCAUAUGGUUCUAUUNGAAUCCGGUUUUACUGGGCUCUUUAAAACAUUUGAUACGCGACGUGCUGUGCGAAGAGUANUGGAAGCAUAAGAUCAAUAUCGCCAAACUCCCUAUAUUUAUUAUAGGGGGUGAGUCAGCUNGGGCCGAACUCCAAGAAGGCUGGUUGGGGGUCGUUGUUAGACGCUCUUGCGUUGUUACAU  >Ss-AA_clean.1_(paired)_contig_1170  GAGCAAGGACAGGGAGGGAUUUACACCCCUUUCUGACUUUGUUCCACAGGUGGGACUAACCGACCAUCCGCCAUAACUUUGUUAUUUUGGAUGCCAAGGAAAGUUCUGCUUGCGACCUGGUUUUAAUUCUUUGCGGUUGAAGUCCCUUCAGCUGAGGGAAUGGUGGUAAGUCUCACGACUUAAACCGUUCCCAAAACUGCUCAGGGUCAUCAACUGUAAAGUAGUUUAAUCACUCUUGCCGCGCUGUCCUCUCUACUUCUAGAGAUGCCAGGGUGGGGAGUGAUGCAACCAUGUCGAGUUCUUGUUCGACGGAAUGGAAAAUCUUUUC  >Ss-AA_clean.1_(paired)_contig_153  CCCCGGGGGUGAGGAUUUCAAAGGGGGUCUGCUCCGGGAAGCUUCGGUUCUUUAGGAUUCGUCUACCAAGGUUUUACACCUCGGACGACUUUCCUUUAAGGAAGAAGCUGCCGGGAAGGUGAGGGUGUUCGCCAUAACCGAUGCGGUAACGCAGUCGGUAAUGGCACCCUUAUCCUCCGCGAUCUUCAAUCUUCUGCGACAGGUUCCUCAGGAUGGUACUUUUAAUCAAAGUGCCCCUUUAGACCGGUUGCGGACCUUGAAGAGAGACGGUAAAUUGGAGGGUCAGAAUUUUUACUCUUAUGAUCUAAGUGCUGCCACGGAUCGUCUUCCUGUGGAUCUUCAGGUGCAAAUCCUGUCGAUCUACUUAGGGGAGCGAUUCGGUAAUCUCUGAAAGUCAGUAAUGACUGACCGAGAUUGGAUGGCCAAAGUGGUGACCGUUCCUGGUCUUCCAGCUGAGGAAGAGCAGUUCCGGUAUCGCGUUGGUCAGCCCAUGGGAGCACUUAGCUCGUGAGGUAUGUUAGCUCUAACUCAUCAUUUCGUGGUACGGUUAGCCGCCCGUCGGGUUUCUUUACCCGAUUUCGGUGACUAUGCCGUAUUAGGAGAUGAUGUUGUUAUUGCUAAUGACUCUGUCGCAAGGUCCUA  >Ss-AA_clean.1_(paired)_contig_15  CGUCGGGUUAAACAUCGUUUUGAUGUUUGUACCUGGCAAACUGCGGUCCGAAAGGCAAUAGAGUUUCAGAUUUCGAUAUCUGAACUCUAUUAUCCUGUCGGGUUCUCAUCAUAUCCGUCUGAUUUCCGGUGUUCUCCUGUCAGGGAGGCCAUCGUUAAUCAGAACUCCUUGGACUUGCGUAAACUAGGAGCAUCUGCUCCUCGUUGACGAUAAGUCUUCGGGGGUCCCUUAAUAAUGAUGGAUUAUUACAGGGCGUCGUAUGAACAAGAGAUU  >FirsU_Contig42  CUCCGAAGACCAACCGUCACCUAGGAGCAGAUGCUCCAAGGGAUUGAAGGUCGAGGGAGUNUCUGGUGUAUGAUGGCUCUCCGAACAAGAGAGUUCUCAAAACCAGACGGAUAUGACGAGAAACCCGACAGGGUAAUAGAGUUCAGAUAUAGAAAUCUGGAAUUCUAUUGCCUUUCGGACGGGCUGUUUGCCAAGUUAAAACAUCAAAACGAUGUUUAACCCGACGAACAGCAUCAAUAAUNCUGAUUUGCUCGGACUGGAGUCAGCGAGCUAGACAUCGUUAAAAACGAUGCUAGCCCUACNUCGAGUCGGAACAAAACCGAAAGGUCCUUUGAUAGUCCACAGANAUCGAUUCCAGAUGGCUNCUGACGAACCGUCGGGACCCGACUCAACAGACUAUCGACCGAGNUCCUCGGUGAAAGUAACNACCCUUCCCGCUCAAGUCCCGUAACAGAGAUGGAACUCCGUUANAGAGACUGCAGAGCGAGNAAGUGCAUUACUAGCACCCAACGGAGUAAGUUCAACAUCCCUANGACACCAAUCUCUUUGCNAAACUCGAAAGAAUGCUCAGAGACUAGAGUCUUGGAAAGGUUANAUCUUAACUCCGAGGAUAUCCACCAUGAUACUAUGGUAGGACCUUGCGACCAGAUCAUUANGCAAUAACUACAUCAUCACCCAGGACCGCGUAGUCGCUAAAAUCGUGUAAAGAAACACGGNCGAGCAGCUAAACGGACUGUGUAAUGGUGUGUUAACGCUAACAUACCN  >Ss-AA_clean.1_(paired)_contig_202  UCCGAUCUCACACUUUCGUGUGGGUAACCUCCCUCGACCCCGAGCGUAAGCUCUUUUCCGAGGGGGACGGCACUUAACUUUGCCGUUCUCACUAGGAAUCGCAGGUUCUUAUCUCUGCUACCUUGGAUACCAGUUUACUGGCUUUCGAGGCAAGUGAGAUAACCGCUUGCGACCGCGUCUUAAUACGCUCAGGUGCCAAUCCUUUAAGUUGUGGAAAGGGCUCUAAAUCUCACGAUUUGAAGUCCUCCCAGAACUGAAGGGGUGACGCUCAAGUAUAUUGAGACAACUGCCUUAACCCUUCUAAGGAGCUCUUUUCUAGAGCCCCUAAGACGGGUAACGUAGCCCAGGAAUUGCUAGCGCUUGGAGCGUAUCUCUCUUUGAUUUCCUCUAUUGUCUCACUUCUACCUUCUCUAGCGGCUAGGCUCUGAUAUGGGGUUCACCCUAUAUCAGCCAACCCGUUAAUGAAGUUGUGAAGCCUAGAUAGCUCUCGUUUCUUCCCCUCGACAACGACACUUUGAAGAGUUCGUAAGAACAAUUCAGAAGCCUCGUUGUCACCCAGCAUUAAAGGUUUACCGUAGGCGGUGUCUAAGAAUUUCUUAGUCCACUCCGAAGAUAAGUCUUUUGUUGCUAGGGCGAAAGGAAGGGCGAUUACUAUCUUUCUAUGUAGGUUGAUUUUCAAUCUAUCAUAGCGAGUCAUUAUAGAGGUUAACGAGGAGACGAACGUGUCGAGGUUACCUACGGAGAAAGGGAGAGGAUAUCCCCUCAUUGGAGCUACAUCGAUUAGCACGGGUACCAAUUGCUGGUAUCUAUGCAAGGUCGUGUAUACUCCCUUGAUAGGAAAUCCGCUCACAUGGCGAGAACCGAAACCUCAUGUCUUGGCGAACUCGAAUAAGUCUUUCGACUUAUGAGAUUUCGCUUCUGAUAUUUGGACUCCGAUCUCUGCCAUUAUCUCUAAAUAGGCGGUGGCUAGCUUCUUAGUAGAUAUCACGAUAUCAUCACCAAGUAUAUAGUAUUUAGCAUUCGAACCACAUCUGUGUUUCGCAAUCUGAACUAUCACGUGGUGUGACAAAGUGAACAUAGCUCAAGAACUCUUUGCUCCUAAGGGUUGACCUGUUCGGAAGAAUAGGUCUUCCCCCUGGAAAGCAAAGGGAUGCUUGACCAUGGCAUCUACUCAGGAGUUAGCGUACUCGGCAUCUAUCAUUCAGGUCAAGACAGUUCUCUGUAUUUCUAUAGGGAACCUGUCUGUUGCCGAUGAUAGAUCCAACGAAUAUUUCCACUGCGAGGUGUUGUCCAUCAUCCCCACUACACCCUUUUGCUGAUCGAAGGUACAAUCGGCUGGAAUUUCUUUCAGCUUCUUAUACAAUAGGUCGUGCAAAGGAGUUAGUGCGGCCUGUGUCCAAUACUCAACAAUGGCGAAAGGACGGGACUUCCCCUCUGAAUCUGGUUUGAUGCUCAAUCUCCGCAAGUGGGAUGUCUUAGGCGGUUUUGCGGCGAAGUAAUUCACCAGCAUCACUGCUUUUGACAUCCACCCGUGAAUUUUGGUCAUCGCAGAUUCAAGGAUAGGUCCUCCUAACGUAUAUAAGUUGGACCGUAAGUCGUCUGGUAGAAUCAUUAGAUCCGAUAGCGCUCUAGCUAUAGACAACCCGUUUGGGCCUGCUGAGGAUACCCAUUGGAAUCUUUUCCAAAUGGGUCUCUCCUCCCGGGUUACCCCCAAUGCACGUAGUGCUAUCGCAAUCUCAGCUGAACCUGGUAGCUUGCUACCUGUUCACGGAGCUGUGAUAGAACUAGUGUUUAUCGGCUUCCCUCCCAUCACUAUCCGAGAUAUAGACAUUAAUGUCAAACAUCAUCGGAACAAGUCAUGGUCAGGAUCCGAUUUUCUAAGUGCCUUCCUUACCUCUAGUGGUAACACUUUUGGCAGGAAGUCUAUCUUAGAAAUAGGGGUUCCGGUCUUCUCUUUUAUAGGGAAGCCGGAUUUCCAUCGGGUCAGACAUAGUCGGUUCGCUUUCAUCUCAUCUGCUGCAGCCACCCUACCUCGAGUUGUAUAGAUUUUUUCAAAUCUACUCAACGUCGAGGCCUGAGCGGCUGCCAGUAGUUUGGGAUCUAGGUGUGCGAAAUAAAUACUUGCGAUUCAGUCUAAUGAGCUUUUAAGUUUAUAAAAGUUUAUUUUAUUGAUUCGCAUUGUUUAUCGUCAUACUUACGGGGUCGACGUGCCCUUCUGCCAGACCUCUCGCGGAGUCGGGUACCCGCGCGAAUCUCCCUGGUCCUACAAACCCUUAACGGGUUUACACCUUUGUAAAUGUUUCCGGUUGCUGCAAGCUGAACGGUUUUACUCCGACAAGCGAUUGUCGACAGUAGAGCCGGAAGCCUCAUCAAUCAGUGAGACCCCUUUACAAAGUAUGGAUUCAGGAGACCCGCGUAUCACCACUAGUUGCGAGGUGAGUUGGUAGCUCCUUUCGGAGGUGUCCUCACCCGGGGUUCCGUGGUGUUUACCCUGUCUUUGACAGAUCGGA  >Ss-AA_clean.1_(paired)_contig_226  GCAGUGACCAUACCUCUCUCCCGGCACUCAGACGCGGCAUUGGUGUUCAAAAGAGCACUUAUGGCGGUCGAGAAGGAAGGGAAACAGGCAGAGCUAGAGAGGUUAGAGCUAUUCGCCGAAGGGCUGAUGACCACACUAGACUCAAAUUGAGCUAGCAUGGAGCAAGAGUCCUACGAGAAUAGCGCUAAUGACAACUCAGACAGCGAAAAGAUAUUCCAUUCCGUCGAACAAGAACUCGACAUGGUCGCAUCACUCCCUAUUCUGGCUUCUCUAGAAGUAGAGAGGACAGAACGGGAAGAGUGAUUAAACUACUUUACGGUCGAUGAUCCUGAAGAGUUUUGGGAUAGGUUUAAGUCUUGAGACUUACCCCCAUUCCCUCAGCUUAAAGGAUUACAACCGCAAAGAAUUAAGACCAGGUCGCAAGCAGAACUUUCCUUGGCAUCCAAAAUAGUAAAACUAUUGCGGAUGGUCGGUUAGAAUCACUUGUGAAACAAAGCCAGAAAGGGGUGAAAAUCCCUCCCUAUCCUUGCUCUGAGAACUUCUUCUCG  >Ss-AA_clean.1_(paired)_contig_737  ACUGAGCUAAGUUGAUGACUCAAUAUAAGCGUCACCAGUUGAAUCUGGCGGCGAAAAUAUACAAGUCAUUACUCUUUAUUCUGUCUGUAAAAGAGAUAGAGAGCCACUGGACUGGAUUUUUCAUCCAGGCCGUGACCUCCUCUCUCUCCCGACCCUCAGACGCGGCAUUAGUGUUCAAAAGAGCACUUAUGGCGGUCGUAAAGGAAGGGAAACAGGCAGAAUUGGAGAGGCUAGAACUGUUUGCCGAAGGUAUAAUGACCACUCUGGACUCAAAUUGAGCAAGUA  >Ss-AA_clean.1_(paired)_contig_227  UAUAUAGGUCAUUACUCUUCAUUCUGUCUGUUAAGGAGAUAGACAGUCACUGGCCGGGUUUUUAUAUCCAGCACGUGACUUCUUCUCUUUCUCGACCCUUAGACGCGGCAUUGAUGUUCAAAAGAGCAUUGAUGGCGGUCGUAAAGGAAGGGAAACAGGCAGAGUUGGAGAGGCUAGAGCUAUUCGCGGAAGGCCUAAUGACCACGCUAGACUCAAAUUGAGCUAGUAUGGAGCAAGAAGCCUACGAGGAUAACUCCGGCGGCAGCUCAGACAGCGAAAAGANNACAUGGUUGCAUCACUCCCCAUCCUGGCCUCUCUAGAAGUAGAGAGAACAGAGCGGGAAGAGUGAUUAAACUACUUUACUGUUGAAGAUCCUGAAGAGUUUUGGGAUAGGUUUAAGUCGUGAGACUUACCACCUUUCCCUCAGCUUAAAGGGCUUCAACCGCAAAGAAUUAAAACCAGGUCGCAAGCAGAACUUUCCUUGGCAUCCAAAAUAACAAAGUUAUGACGGAUGGUCGGUUAGUCCCACCUGUGGAACAAAGUCAGAAAGGGGUGACAAUCCCUCCCUGUCCUUGCUCUGAGAA  >Ss-AA_clean.1_(paired)_contig_916  GCAACCAUGUUGAGUUCUUGUUCGACGGAAUGGAAAAUCUUUUCGCUGUCUGAGCUGUCGCCGGAGUUAUCCUCGUAGGCCUCUUGUGCCAUGCUGGCUCAAUUUGAGUCCAGAGUGGUCAUUAGACCUUCGGCGAACAGUUCUAGCCUCUCCAGUUCUGCCUGUUUCCCUUCCUUUACGACCGCCAUUAGUGCUCUUUUGAACAUUAAUGCCGCGUCUAUUGGUCGGGAGAGAGAAGAAGUCACGUGCUGGAUAUAAAAACCCGGCCAGUGGCUGUCUAUCUCUUUAACAGACAGUAUGAAGAGUAAUGACCUGGAUAUUUUCGCCGCCAGAUUAAUCUGGUGUCGUUUAUACCUGGUCAUCAGCUUGGCUCAGUCUUGAGAGAAAUUCCAGAGGUUCCCUACUGAGAAAGGGAGCGGGUAACCUCUCAUUGGAGCGAUGUCCACUAGGACUGGUAUCAAUUCUGAUAUCCAGCCUAUUGUGGAAUACACUCCUUUGAUCGGAAAACCGCUAGCUGGACGGCCUCGGU  >Ss-AA_clean.1_(paired)_contig_140  UUAAGGGUCUUCCCCGCUCCUUUCAAGGAGCCUCGCUUUAGCGGAUUUGACACUCCGUUAAGCAGCCUUAAAGAGGCAGCGCCCGAUGGCGCAACUCUUCUCUCACCUGUUCAAAGAAAAUCUUUCCUUUGAAACGAGACGAGAAGGCGUAGUCCUCAAAUGUCUGCCGCACGAAGGGGUCCCCCCCUCCGAUGGAAACUUCAAAAGACUGAUCCGAGAUCAGUUCUUUGAUGUAUGCGACAAUCUCUUGUUCAUAUGACGCCCUAUAAUCCUCCAUCAUUAUUAAGGGACCCCCAAAGACUAACCGCCAGCGAGGAGCAGAUGCUCCAAGCUGUUGUAAGUCUAGAGAGUUCUGAUUCGUGAUGGCCUCCCUGACGGGAGAACAUCGGAAAUCAGACGGAUAUGACGAGAACCCGACAGGAUAAUAAAGUUCAGAUAUAGAAAUCUGAAACUCUAUUGCCUUUCGGACCGCAGUCUGCCAAGAUGCAACAUCAAAACGAUGUUUCACUCGGCGUACCGCGUCAAUAAUCUGAUUCGCUCUGACUGGAGUUAGCGAGCUAGACAUCGUUAGAAACGAUGCUAGCCCUUCUCUAGUUGGAACGAAGCCAAAAGGCCCUUUGAUAGUUCAUAGAAUAUUAACCAACUGGCUCUUACGAACCGUUGGGAUAUUGCUAACCAAACUAUCAACCGAGUCCUCGGUGAAAGUAAUACCCUUCCCACUCAGAUCUCUUAAU  >FirsU_Contig3  ACGUGAGAGGGGGGAAGACCCGGUUAAGGGUCUUCCCCGCUCCUUUCAAGGAGCCUCGCUNUUAGCGGGUAUGACACCCCGUUAAACAGCCUUAAAGAGGCAGCGCNCCGAUGGCGCAACUCNUUCUCUCACCUGCUCAAAGAAAACCUUUCCUUUGAAACGGGACGANGAAGGCGUAGUCCUCNAAACGUCUGUCGCACGAAGGGGUCCCCCCCUCCGAUGGAAACCUCNAAAGGACUGAUCAGANAAUCAGCUCUUUGAUGUAUGCGACAAUCUCUUGCUCAUACGACGCNCCUAUAAUAAUCCAUNCAUUAUUAAGGGACCCCCGAAGACUAAACGUCAACGAGGAGCAGANUGCUCCAAGCUUUUGUAGGUCUAAGGAGUUCUGAUUAACGAUGGCCUCCCGAACAGGAGANACAUCGGAAAUCAGACGGAUACGACGAGAACCCGACUGGGUAAUAGAGCUCAGAUAUAGANAAUCUGAAACUCUAUUGCCUUUCGGACUGCAGUCUGUCAAGUCAAGACAUCAAAACGAUGNUUUAACCCGACGGACUGCAUCAAUAAUCUGAUUCGCUCUAACUGGAGUUAGCGAGCUAGANCAUCGUUAAAAACGAUGCUAGCCCUUCUCUAGUUGGAACGAAGCCAAAAGGCCCUUUGAUNAGUUUAUAGAAUAUUAACCAACUGGCUCUUACGAACCGUUGGAAUAUUACUAACCAAACUNAUCAACCGAGUCCUCGGUGAAAGUAAN  >FirsU_Contig221  GGGUAUGACACCCCGUUAAACAGCCUUUAAAGAGGCAGCGCCCGAUGGCGCAACUCUUCUNCUCAUCUGCUCAAAGAAAAGUUUUCCUUUGAUGCGGGACGAGAAGGCGUAGUACUCAAAUNGUCUGUCGCACGAAGGGGUCCGCCCCUCCGAUGGAAACUACAAAGGAUUGAUCACAGAUCNAACUCUUUGAUGUAUGCGACAAUCUCUUGCUCAUAGCUCGACCUAUAAUAAUCCAUCAUUANUGACGGGACCUCCGAAGACCAACCGUCACCGAGGAGCAGAUGCUCCAAGGGAUUGAAGGUNCGAGGGAGUUCUGGUGUAUGAUGGCUCUCCGAACAGGAGAGCUCUCAAAACCAGACGGAUNAUGACGAGAACCCGACAGGGUAAUAGAGUUCAGAUAUAGAAAUCUGAAACUCUAUUGCCUNUUCGGACGGCUGUUUGCCAAGUUAAAACAUCAAAACGAUGUUUAACCCGACGCACAGCAUNCAAUAAUCUGAUUUGCUCGGACUGGAGUUAGCGAGCUAGACAUCGUUAAAAACGAUGCUANGCCCUACUCGAGUCGGAACAAAACCGAAAGGUCCUUUGAUAGUCCAUAGAAUCGAUUCCANGAUGGCUCUUACGAACCGUCGGGACCCGACUCAUCAGACUAUCAACCGAGUCCUCGGUGANAAGUAACACCCUUCCCGCUCAAGUCCCGUAACAGAGAUGGAACUCCGN  >Ss-AA_clean.1_(paired)_contig_85  CUGGACUACUCCCGAAUACUCCAGCACCCCGGUCUGUUAAACCUUUUAGCAGGAGCCACAUCAGUUACCUGAUGAGGGUCUUGAUUGUCCCCAAUGGAAUUUUCUUCCAUAGGGUUACAGUGCAAUACUCAUAGGGCAUGCUAAGUAGGAUGGUGUUUUAUUAUUGGAAAUUAUAAUAUCGAUUCCCAGAUCUUAGGGAACUCUUUAUUAUACUUUUUACUAAUAAGACCUGACUUGAUAUUGGCUACCUUAUCUCAUCUCAAUGCAUCAAAACGUGAUGCAUCGAGAGUAGAUAGGUACCGCAUUACUGCGGCAGUACCUCCUUGG  >FirsU_Contig26  GCCAUUACCGACUGCGUUACCGCAUCGGUUAUGGCGAACACCCUCACCUUCCCGGCAGCUNUCUUCCUUAAAGGAAAGUCGUCCGAGGUGUAAAACCUUGGUAGACAAAUCCUUAAGAACCNGAAGCUUCCCGGAGCAGACCCCCUUUGAAAUCCUCACCCCCGGGGAAGAACUCGAUAAAANUCAAGUAAAUCUCCGAAGUUAGGACGAAGUGACCAGGCCUUGAUGUCUAACCAGAUACCANAGACCGGCCACUUUACAAUUGGGACCCGCAGAACGGGAGAAGAGUCAACGGACUGGUCCUNAGUUCCCCUAGACGGGGAAGAGAGGACUUAGCCGCUAACAAUUCAUAAAUUGGAAGGGUANGAAGAUAACCCCGUGAAGGGACCCACUAUAGUGUCGAAGCUCACGCUACCAGGGAACUUANAUAAUUCGGAAAACCGAAAUACAAGUCAAAACUCCUCUAAUCACGCCGGCGUCUCCAUUANUGCAUCCGCAGACGAAGAACACCGGGAAUGAUCGAGGGGAGCCCCCGGACAAUGGAAAUAGGUAACUNCGUCAGUGACGAGAACCGGAUUUCCCGACACGAAGUGCUGAACGAUUCGGUAGCACUCCUNUUAGGUACAACACAGUGAACUUACGUCCAUUGUGAGUAAUAAGGGAGCGCACCCGCACCGNCGAACUUCAUGAAGUUACGCUUGUCUAACUGAAGUGCUCAUAUCAAAAGCCGGAAAUAUUNGAAAGAAAAGAAAAAGAUUCAAUUCUUUAAUAGGACCGACUUUGCUAAGACGACCCCCCCUCUUGUUUCCAAGAGGAAUGGUUAGUUUUAAAUUGAGUUNUUCAUGAAAUUUAAAUAAUUGUUGCACAUUCCUACGUCCAAAAGGACCUGCGCUUAGACGNCGCCCUCGGAAUGCUCAGCGCCAGGUGGCCGGCGUCAAAUACACGAAGUAUUUGAGCGGUNGCCCGCCUGACCUGCGACAACUUAAAACUCUGAAAACUCAACCGAAGCUAAUCCUUACCGNGAUUGAAUCCCGGUCGAGGUCUCAGGCUUCAGCACACCAAAUGGUGCUUGUGACUUCGUCNAAAGACGAUCACCGCUAUUUGGGGAUAAGCUUUGCUCCAACCAAAACUGGNCGCCCAAAGCGCCCCGCACGUCCGGAAAGACGUAAAGAGAAACCUUGGAUCAGCUUCGGC  >Second_Contig2  UUAGGGAACUCUUAAUUAUACUUUUUACUAAUAAGAUCUGACUUGAUAUUUGCUACCUUANUCUCACCUCAGCGCAUCAAAACGUGAUGCAUCGAGGGUAGAUAGAUACCGCAUUACUGCGNGCAGUACCUCCCUGAGAAUAAAUCUCUGAAUAGAGGGUAUUCUCAAGGGUGUACUUGGCANGUAUCAUAUCUGUCUACAGGCUUGAUAAAUUGUAGAACAUAAAAUCAAAAACCGGGACCGNAAAAUCGCAAAUACAGAGUCAAANGACAAAGUAUCUGCUAUCACGGGCCAUUGAUGAUCUAUAGAAGUUCUUAAUAAAGUAAAGNAAAAUCUUUACUAGGAUUAAGCUCUGAAAAUUGCUCAAACGCAAUCUUCACAGAUACAUCNUAUAAUAUCAUAAUGGUUUGCUUCUCCACUUUCUCAUAAAGCCCUAGAAUACAUGUCUAUNCGCAAUAGCGUUCGACAAGUUGGGCUUUUCUCGAAUAACCUUCGAGAAGAAGUUUCAAAGNCACAAUUUCGGCCUUCACCUUUAUAAAACGAGGUGAUGACGAAACUAUGCUAACUACGUCNACAAAAGUUGAUGAUUUUCAAGGAUAUAGCCUCUCUUAUAACAGAAGAGACNNNNNNNNNNNNNNNNNNNNNNNNNNNNNNNGAUGAUAUGGACUUGUAAGGGUUAAUUUCCAAACCAAGANCCUUCCAUCAACAAGCUAUACUGGUGAGCAACAGAGUCAUUACGAAUGACAAUGUCGUCUNCCAAGGAUAGCGUAAUCAUCAAAGAAAGCCACGAACCCUGAACGGGCUGCAGCUUCUUGGNACAAUCACAUGAUGUGUCAUAGCCAACAUGGCUCAUGAGGAAUAGGCACCCAUUGGUUGANCCCACAGAAUACUUGAUAUGGUACAAGUGUUCUGGCGUUAACCAACUGAUGUCGAGAAGANCUACGUCAGAUCGGCCCCGGAAAACCUAAAACUGAUAAAAUAUCAGAUUGAAGAUCAACCNGGAAGUCGAUCCGUUGCUGCGGUUAGAUCAAAACAGGAGAAUACAACCGGAUCUAGACCANGCCUCUAAAN  >Ss-AA_clean.1_(paired)_contig_126  UAAGGGUCCCUCCCGCUCCGUCUAAGGAGCCUCGCUUUAGCGGAUUUGACACUCCGCUUGAGCAGCCUUAAAGAGGUAGCGCCCGAUGGCGCAGUUCUUCUCUCACCGCCUCAAAGAAAAUCCUUCCUUUGAAGCGAGAUGAGAAGGCAUACUCCUCGAAUGUCUGUCGCACGAAGGGGUCACCCCCUCCGAUGGAAACUUCAAACGUCUGAUCAGAGAUCAGAUGCUUGAUGUAUGCGACAAUCUCUUGCUCAUAGCUCGCCCUGUAAUAAUCCAUCAUUAUUAAGGGACCUCCGAAGACUAACCGCCAUCUAGGAGCAGAUGUUCCUAGAGACCGUAAAUCUAAAGAGUUCUGGCGCACGAGGAAUUCCCGAACAGGAGAAUUCUCGAAGCCAGACGGAUAUGACGAGAAACCGACAGGGUAAUAGAGUUCAGAUAUAGAAAUCUGAAACUCUAUCGCCUUCCGGACCGCAGUUUGUCAGGUAGAAACAUCGAAACGAUGUUUUACCCUACGAACGGCGUCAAUAAUCUGAUUCGCCCUAACUGGAGUUAGCGAGUUAGACAUCGUCAGAAACGAUGCUAACCCUUCUCGAGUUGGGACGAAACCGAAAGGUCCUUUGAUAGUUCAAAGAAUCAGUUCCAACUGGCUUUUACGCACAGUGGGAACACGACUAAUUAAACUAUCUACGGUUUCUUCGGUGAAAGUAACACCUUUAUUGCUCAAGUCGCGAAGUAGAGAUGGAACUCCACUAAGGGACUGAAGAGCAACUAAAGCAUUACUCGCACCCAAGGGCGUUAGUUCGACAUCUCUCGACACCAGUCUCUUAGCAAACUCAAAAGAAUGCUCAGAAACAAGUGUCUUUGACAUGUUGAUCUUAACUCCGAGGAUAUCAACCAUGAUACUAUGGUAGGAACUUGCGACCAGAUCAUUAGCAAUAACCACGUCAUCACCAAGUAUGGCAUAGUCACCGAAGUCGAGUAGACCUACUCGACGGGCGGCUAGACGUACUACGUAAUGAUGGGUUAAAGCUAACAUACCCCAUGAGCUUAAGGCACCCAUAGGUUGACCAACGCGGUAACGAAACAGUUCUUCCUCAGCAGGAAGCCCCCGUAUCGUUUCCACUUUGGCCAUCCAAUCUCUAUUAGUCAUAACUGACUUCCAUAGAGAACCGAACCGCUCACCCAAAUAGACGGACAGGAUUUGCACCUGUAAAUCUACUGGAAGACGAUCGGUGGCUGCACUAAGGUCAUAGGAGUAAAACUUCUGACCUUCCAAUUUACCCUCUGACUUCAAGAGUCUCAGGCGGUCCAAGGGUGCACUUUGAUUAAAAGUACCAUCCUGGGGAACGCACCGGAGACACCUGAAGAUGGCGUCCGAGAGUGGUGCCAUCACUGACUGCGUAACCGCAUCGGUGAUAGCAAAAACCCGGACCUUACCAGCGGCCUCCUCCUUAAAAGACAACCGACCGAGGUGUAACACCUUGGUAGGAAGGUCCCGAAGGGAGGAAGCUUCGGUUAGGAGGGCCGAACGGAAAUCCGAACCUCCGGGAAAGUACUUGAUAAAAUCAAGUAAAUCCCCGAAGUUAGGACGAGAUGACCAGGCCUUGAUAUCUAACCAGAUACCUAGACCUGCCACCUUACAGUUCGGUCCCGCCGAUCGAGAGAACAGUCAACGGAGAGGACCGAACUCCCCCAAAGGAGGGAGAGAGGACUUCACCGCCAACAGUUCAUAAACCGGAAGGGUAGAAGAUAACCCAGUGAAGGGACCCACGAUAGUGUCGAAGCUUACGUUACCGGGGAACUUAAUCACUCGGAAAACCGAGAGACAAGUUAAGACUCCUCUAAUUACAUCAGGGUUUCCGUCGUGCAUCUGCAGACGGAGACUACCUGGAAUAAUCGAGGGGAGCCCCCUAACGAUGGAAAGAGGUAACUCGUGAGUAACGAGUACCGGAUUUCCCGACACGAAGUGCUGAACGAUCCGGUAACACUCUUUCAAGUACAACACAGUGAACUUACGUCCAUUGUGAGUCAGGAGAGAGCGCACCCGGACCGCAAACUUCAUGAAGUUACGCUUGUCCAAACGAAGUGCUCAGAUCAAAAGACGGAAGUACUGAAAGAAAAGAAACAAGUUCAAUUCUUUAGUAGGACCAACUUUGCUGAAACGACCCGCUUCAUGUUUCCAUGAAGAGAAAUUAGUUUUAAAUUGAUUUUUCAUGAGAAAUAAAUAAAUUUGUUGCACAUCCCUACGGUUCAAAGAACCAUGCCUUUAGACAGGCCCUCAGGAUGCUCAGCGCGAAGUGGCCGGCGUCACACACACGAAGUGUGCGAUCGGUGCCCGCCUCGCCUGCGACAAUUUAAAACUCAGAGAAAUCAACCAAACACUAAUCCUACCGGACUAGAAAUCUGGUCGAGGUCUCAGGCUCUAGCACGCCAAAGGCGCUGUGACUUCGUCAUAGACGAUCACCGCUAUUUGGGGAUGAGCUUUGCUCCAACCAAAACUGGUGUCCAAGGACACCCCGCACGUCCGGAUAGACGUAAGGGCAAGACCCUGGAUCAGCUUCGGCUG  >Ss-AA_clean.1_(paired)_contig_12119  CAACCAUGAUACUCUGGUAGGAACUUGCGACCAGAUCAGUAGCGAUAACAACAUCAUCACCUAGAUCGGCGUAGUCACCAAAAUCGUGUGAAGAGACACGACGGGCGACCAACCGAACUACAUGAUGGUGUGUUAAAGCUAGCAUACCCCAUGAACUAAGGGCUCCCAUGGGCUGACCAACGACAUAACGGAACUGCUCUUCCUCAGCUGGAAGCCCAGGUACCGUCACCACCUUGG  >Ss-AA_clean.1_(paired)_contig_76  AGGGGUAAAUCCCUCCCACUCUACAGAGUGCCUCGGGAUCCCUGAUUUCCUCCUACGCAUUGUUCACCUACAUAGUAGGCGGCAGAACAAGCGAGCCUAGGGCUCGGUCGCUACGGCGCCUGACUACCCGACGGCCCCACGGUGUGGUGUAGCUAGAUAGCCAACUAGCGCGAUUGCCUACCGGGGAGAAACCUAUUCAGCUCAUAUUACUGAACCGAAAGGUCACUAUAUGGGCGGUGUAGAUUAAGAGGAAGUUCAAUUGGUAGUCUUAUGACUCAACCACUAGUUUUAAAACUAAUGAAGUGAGUCCGUAAGUACUAUUACAAAGGAACUUCUCCGAAUCUAGCCGCGUUUACCCGACAGGCAAUACACUUGUUCUUCCUGUGAGGGAAGACCAAGGGGUGAAAGUGGACCGCCACAGCGUUUAAGCUGUCACGGUUAGCCAA  >Ss-AA_clean.1_(paired)_contig_73  GCGAACUCAAAUAAGUUUUCACUUACGUGAGUUUUAACCGGAGAUAUGUCUACACCCAGUUGAGACAUCACCUCUAAGUACUUAGCCGCUAGUGCAUGAUCACAUAUAACUAUGUCAUCACCCAAAAUCUUGUAUCUAGGAUUGUCAAUGUUUAAAACCAUUGCACAAUACUGGACGACAAAAUGAUGGGAUAAGGUGAACAUAGCUCAUGAUGAUUUAGCCCCUAGAGGUUGACCACAUUCAAACUUAACCCUGGGUCCGCCAGGUGUGUCGAAUGGUAAAUCAAUCAUAGCUCGUACUCAGGCAGUUGCAUACUGCUCACUUGUUAACCAUGCUAAUACCAUUUCCUGUACGAAUAUAGGAAACCGGUCAGUAGCAGAAGUUAAGUCAAAUGAGUAGUAUGGACCACCUCCGUACAACAAUUCCUUAAACCCUUUGUUUUGGUCGAAAGUACAAUCCUGAGGUAUAGACUCCAAUAUGCGAUAUAAUCUAUCGUGUAAAGGGGUCAAUGCGCUCUGGGUUAUAUAAUCGACUAUGGCAAAUGGUCGGGACUUGUACUCCUUAUCUUUCUUGAUGGAAAUCCGACGGAGGAGGUUACCAGUUUUAUCUGGGUCCAGCUUUGUAAGUUUUCAUAGAAUACCAUUAGUGGUUUCUACGAAUCCUUUAAGGCUGGAAACCUUCUCCUUAAACUCAGGACCUCCUAGGGUGUAAAGACACUCUAGGAUAUCUUGAGGGAUAUUCAUCAAGUCUGAGAAAGAGGUAGCUAUGGAGAUCCCGUUCGGCCCACUGGUGGAUACUCAGGGGAAAGUGAAAGAUGUGGGUUCAUCUAGACGACUAAUCCCCAUUCGUUUCAUAACGAUAGCGACACUCGCCCCCGUGAAAGGGUUUGAUCCCGAACACGGACGAGUGAUGCUGUCAUAUGAUUCGAUGACCCCUGUUCCUAGUAUCAGCCGUGACAGUUGCAAAACUGUCAGAACGUAUGAUAUCAGGAGGAAAUCUUUUCUUAAGAUCCCGAGUCUGACCUGUAAUGGCAACAUCAUAGGUAGAUUCGUUUUCUUACAAAGAGAAAUUCCAAAGGGUCGUGGAAGCUCCUGGCCAGCAAGUGUACGGGUGAUAGCCAACCGAGACAGCUUGAACGCUGUAGCGGUCCACUUCCACCCCUUGGUCUUCCCUCACAGGAAGAACAAGUGUAUUGCUUGUCGGGUAAACGCGGCUAGA  >Ss-AA_clean.1_(paired)_contig_2  UCGACCGUCACCCGGCCUCGCCAUGGGAGGAACAUUAUUUGUGACAGCAUGGACUGUACGGGUCUAAUGAACCGCAUAGACAUGUCUGCCAACUUAUCUAUCUCCCUAUCCUUCAUGUACAACACUGCUCUUUUAUAGGCAGCGAAGACUUGAGGGAUAGCCAAUCCGCUAAACCCUGCAGCCUCGGCAAACCGUAAAAGGUCAGACUGAGCUACCAGGUAAGGCUUAUUGGACAUCAGGAGAUAAGAUAGAAGCUUAGCAGAGUGGAUCAAACUAUUCACUAGUCUGGGUUUAUACUCUUUGCCAAUCUUCACUAAGCUUUCCCAGAACCGAGGAGUGUUUCCGG  >Ss-AA_clean.1_(paired)_contig_207  UCACUUCACAAGUGGAAUAACGCGCAACCCCCCGGGUACGAAUACCCUAUUUGUUGCGGUAAUUGAGUCCCUAAGUACACUACUUAGGAUCUUCUCCUUUCGCUAGCAUAGUCAAUUCCUUGACUAGCGCCUUUAUCAAGGCGGCUCGCGUCUGACCGACCAGCAAUUUUGGGCGAUUUGGUUGUAGACCGUUUAAAUUAGGCAUUGGCCUGAUCGGAUGAUCAUCCAACACCUUAUUUAGCGUCAACAGAUCUUCGGCUUCCUCGAAUUCUUUCCGGAAGCUCACGUAGAGCUCCUUGUUAGAAUCGAUGGAAUCGAUGAUCGGUAUGAAGCGUCUAUAAUCGACCAACACCCGGCCUCGCCAUGGGAGGAACAU  >Ss-AA_clean.1_(paired)_contig_143  GCCUAUUGUGGAAUACACUCCUUUGAUCGGAAAACCGCUAGCUGGACGUUUUCGGUAUAUUCAUGUCUUGGCAAACUCAAAGAAUCCUUUGGAUCGAUGAGAUUUGCUAUCAGAUAUGUCUACACCUAGACCGUCCAUAAUCUUCAUAUACUCAGAGGCUAAUUUCUUGGUCCUUAUCACUACAUCAUCACCUAGGAUUACGUAGUUGCUGAGCGGUGAGUUUGUUCUCAUCGCCGCAAUUACCAUCACCACGUGGUGAGAUAGUGUAAACAUGGCUCAGCUACUCUUUGCCCCUAAAGGCUGUCCACAACUGAACUUCAGUUCGGACCCUUCAUAGGAGAAAGGGUAGUCGACCAUGAUAUGGGAUCAAUGAUUAGCGUAUUCCUUACCUGCGAGCCACGACAGGACCUCAUGCUGGAAUUGCAUGGGGAACCUAUCAGUGGCCGCUGUUAAGUCUAAAGAAUACAUUUGCGAAUUAGGGUACUGGCUCAUAAGCUCGACACCCUUCUGCUGGUCGAAAGUACAGUCUUGAGGAAGUUUCUUCAAGAUCUCGUACAACCGAUCAUGCAACGGUGUCAGGGCCGAUUGAGUCCAGUAGUCUAAAAUCGCGAAUGGGCGGGAUUUUGCUUCCGCAUCUGCUUUAAUAGAGAGUCGCCGAAGAUAUUUUGUCGUAGGCUCCCGAACGUUGAAUAAAGUUUUAUUCAUCGUCGGGUGGUCUUCGAUAAAUGUCUUAGCGGCCUCUAUGGCAGAGGCGAAUGCGUCCCCACCAAUUUCUUUUAGGCUUUGCAGCAAGGGAAGGGGGAGGUUGUGGAGAUCCCUCAAGGAUUGCGCGAUUGAUAGCCCAUUCGGCCCGGCUGAGGACAUCCACUUGAAUUUUGUUCAAGUGGGUCGCUCAGUGAAAGGUUGGAGCCCUAGCUUUCUUAAAGCUA  >Ss-AA_clean.1_(paired)_contig_144  UUAUCACUACGUCAUCACCUAGGAUUACGUAAUUGCUAAGCGGUGAGUUUGUUCUCAUCGCCGCGAUUACCAUCACUACGUGGUGAGAUAGUGUAAACAUGGCUCAGCUACUCUUUGCUCCUAAAGGUUGUCCGCAACUGAAUUUCAGUUCGGAUCCUUCAUAGGAGAAAGGGUAAUCGACCAUGAUGUGGGAUCAAUGAUUAGCGUAUUCCUUACCUGCUAGCCACGAUAGUACCUCAUGCUGGAAUUGCAUUGGGAAUCUGUCGGUGGCCGCUGUCAAGUCUAGAGAAUACAUUUGCGAGUUUGGAUAUUGGCUCAUAAGCUCGACACCCUUCUGCUGAUCGAAUGUACAGUCCUGAGGAAGUUUCUUCAGGAUACUGUAUAGUCGGUCAUGCAACGGUGUCAGGGCCGAUUGAGUUCAAUAGUCCAAUAUCGCAAAUGGACGGGAUUUUGCUUCCGCGUCCGCUUUAAUAGAUAGCCGUCUAAGAUAUUUCGUCGUGGGCUCUCGAACAUUGAAUAAAGUUUUAUUCAUGGUCGGGUGGUCCUCGAUGAAUGUCUUGGCUGCUUCUAUAGCUGACGCGAAUGCAUCCCCGCCAAUUUCUUUUAGGCUUUGCAGCAAGGGAAGGGGGAGGUUGUGGAGAUCCCUCAAGGAUUGCGCGAUUGAUAGCCCAUUCGGCCCGGCUGAGGACAUCCACUUGAAUUUUGUUCAAGUGGGUCGCUCAGUGAAAGGUUGGAGCCCUAGCUUUCUUAAAGCUA  >Ss-AA_clean.1_(paired)_contig_24  CUCAUCAAGAUUUUAUUAUAAAACUAAACGGCCUUUAGGAGUUAAGGCGUGGAUUUCCAUAAAAGUAAUUCCUCUGUAUCUCCUGGUGGCCUCUUGAUUGUUCAGUACAUCUAUUCAUAGAUCACAUGUUAAGCUGUUUGCGACUAGGGUUGUUACCCUAGUUGAGAGAUCUGGUUCUAACUUCACAUUCCUGUAUCUUAAGGAAUGUUUUAGACUAGUUUCCAGAUUUCUAGCAGGUCAGCCGGAUAAAUGUCGUAAGAUCUUUGUUCGUGCUGACAAAGAUGGGUUACCGA  >FirsU_Contig15  UUACUAGAUAUAUAUCUGGUGAACCGCUAAGAUCUAAUUCUUCACUGGUUUCAUUAACUANAUGGUUUUCCUUCAAAGUUCUUAUACUUAAAGGAUUUUAUUGAUAGAGGUGAUCCACGAANUGAUUAGAUUUGUUCUAACAUUAAUGGGUUACACUCGUUCAAUAAAACCUACAAGUAAAGNAAAAGAGCCUAGUUAAUGUUGAUUAUUCUUCUAUUUCAUCUCCUUUUAAAGGGAGAGCAGNGAUUUAAAAUUCCUGAUGAGUUCAUUAAAGAAUUCAUUAAGAAGUAUAAUCUCGGCCUUUNACAUAAAGUGAGAUAAUUCAUUACAUUACAUUAGUAAUAAAUCGUCUCCAUUAGGUAAGGCCCACAUUAACAGGUCCAUUUGCACUGUUCCAUAUGGGACACUGAAAUCUUGAUAUGUUAGAAACAUUUCCGUAAAUUAAUCGGAGAUGGUUCUUUCAAAAUCAUGAUUGGUGAGUUCCUUGAAGUUGUAUNUCAAGGAUCACCGAGCNGUUCCAUACUGGUUGCAGUACAAAUGGUAUAGGAAAGAUAUCAGUUCAUUGAAGAUCCAGANGUUAAAAAUGAGACCAAUAGCUAUGGUAGAUUACUAUAGUCAAUUUGGUUCUUAGACCUAUCCAUGACGGAAUUCUUCAAAAAUUGAAGAACCUCCCUCAAGACAAGGACUUUUACUCAGGAUCCUUUCAAUAAAUGGGGUAAGACCAUGGGGCACAAGUUUUGAUUCACUUGAUCUAACAAGUGCCACAGAUCGUUUCCCGAUUUCGCUUCAGGNNNNNNNNNNNNNNNGUUACAGUGUUGGACAGCCGAUGGGUGCCUAUAGUUCCUGGACUACUUUUACUCUAANCACACCAUUUAGUUGUUCACUAUGCCGCACAUAAGUGUGGAAUAGUGGACUUUAAUAGGUNAUAUACUCCUCGGUGAUGAUAUCGUGAUUAAUCAUGAUAAAGUCGCCCGAGAGUACAUACNGUAUUAUGACUAAACUUGGUGUGGACAUCUCAAAGCAGAAGACUCAUGUAUCUAAAAAUANCAUAUGAAUUUGCUAAGAGAUGAGUAAGAAAGGGCAUAGAGAUCACGGGUCUUCCUCUGANGAGGAAUAGUAUCUAAUACUAAUAACCUACCAGUUGUUGUAAAACAACUUGUAAAUUAUANUGUAUUCGAAUACUACUCUUUAAAGAGGAAGCACAACUGAAUUGGUCUUAAGUGUCUUCANAGGGUAUUAAGAUUAGAAAGAGAUUUGUAUCUCAAUCUAAACUUAGAACCCAGGUUGAAGNAUAUUGUCUUUGUAAUAAGGCAGUCACUUAAGCUUAACUCCUAUCAGGAAAUACGUAAGUNAUCUCCUAGAUAAGAUUAAACUUGAGGAACUGAUUAUUCCAAAGGAAGAUCAAGUUCACANGUGUUAUCCGUGGGAUCUUGUGUCUAGGUCUAGUAGAGGCUGUCGAGGAAUCGGCCAGCGNAGCUGACUCGGN  >Ss-AA_clean.1_(paired)_contig_634  GGUCCCGACCAGUGAUUUGCAUCACCAAGGUUUCUGCCAUUAAACCUAACGGUUUUCCGGUAGUAGCCUUGGAAUCAAAUCUCGCGUUGGACUGACUAAAUAGCUCCACCAUGAUGUUAGCAAGGAUGUUAGUCCCUACUUCAUCAGGAAUGGGCUUUAGGAUGAUCCCUACCCGCCUGGCCAUCUCAGUAAGAGACGAACCAGCCGGAGAGACUCCUCACAUGAUUUUAAUCAUGUUGUUAGUCAGGUAGGCAGUUUUUGAGAACACCUUCGUUAAACGCGAAGGCAUUCUCCUAAACUGUCCAUACCACCUUUCAACCAUAACGUCGACAGGCUUAAACUGCCAACCCUUCUUAACCAUUUCUUCCGUUAACAGGGCGGCCAAGAGAUAGGAUCUCUUGGUGCUCUGCUUCAUGGCUGAAAUAGGGAAGGGCGUUAUCUCAUGACCUUUGUGGAUUCAUCUCUUUGCAAACUCAAGAAUAGUUUUACUAUGAUGAGUUUUUAAAGGGGAUACAUCCACCCCAAGGUCAUUCAUAACCGACAGAUAUCGACUGGCAACUCUUCCAUCUCCUAUUACAAUAUCGUCUCCUAAGAGACAAUAAGGUAAGGUCCGUCACUCAACACCUUCUAGCCUACAACAAUAGUAGACUAUAUAGUGAUGAGCGAUUGCGAAAGAUGCUCAUGAUGAGUAGAACCCCAUUGGGUUACCAACCUCAUAGCGGAUAGGCUUGUAAGCCUUUCCCCUACCGGGGCCGGUAGACACCAUAAAGGGCUCUCCCACCAUAAGAUCUCUCCAGGCCUUAACAUAGGAGGCCGGAAGAUGGCUGGAAAGCACCAACUCGAUUACCUUUAUAGGGAAUCGGUCGGUGGCUGAAGUAAGAUCAAUGGAAUGAUAUUCCGUUGCUCCCUUCAGACCAUCCAUAAAUGCUUCCUGGUUAAAAGUGAAAUCCUGAGGGAUCCUCUUAAGGAUUCUGAAUAAGUAAUUAUGCAGAGCCCUUAACACGGUUUGACUAAAGUAAUCUCCAAUGGCGAUUACCCUAGUCUUACCUUCCCGAUCCGGAAAUGACGAGAUUUUCCUUGUAAAGGGAAUUUCUCGGAAAUCCUCUCGGUGGAUCUCCCGCAGGAAAUCAAAAUUAGCCCGGAACACGUCCAUUAGAUGGGAAAUCUUCUCUCCACCUAACACCCCGAUAUUUUUAAUCAUAUCCGGUGUUAACAAAUCAAAGUCUGACAACCAUGAUCACAAAGCGUGACAUGAUGUCGGA  >Ss-AA_clean.1_(paired)_contig_1286  UCCGACGUCAUGUCACGCUUUGUGAUCAUGACUGUCAGACUUUGAUCGGUUAACACCUGAUAUGAUAAAAAAUAUCGGGGUGUUAGGUGGGGCGAAAAUUUCUCAUCUAAUGGAAGUGUUCCGGGCUAAUUUUGAUUUCCUGCGGGAGAUCCACCGUGAGGAUUUCCGAGAAAUUCCCUUUACAAGGAAAAUCUCGUCAUUUCCAGAUCGGGAAGGUAAGACUAGGGUAAUCGCCAUUGGUGAUUACUUCAGUCAAACCGUCCUAAGGGCUCUGCAUAAUUACUUAUUCAGAAUCCUUAGAAGGAUCCCUCAGGAUUUCACUUUUAACCAGGAAGCAUUUAUGGAUGGACUGAAGGGAGCAACGGAAUAUCAUUCCAUUGAUCUUACUUCAGCCACCGACCGAUUCCCUAUAAAGGUAAUCGAGUUGGUGCUUUCCAGCCAUCUUCCAGCCUCCUAUGUUAAGGCUUGGAAGGAUCUUAUGGUGGGGGAACCCUUCAUGGCGGCUACCGGAUCUGGUAGGGAAAAGGCUUACAAGCCGAUUCGCUACGAGGUUGGUAACCCUAUGGG  >Ss-AA_clean.1_(paired)_contig_635  AGAACACCUUCGUUAAACGCGAAGGCAUUCUUCUGAACUGUCCAUACCAUCUUUCAACCAUAACGUCGACAGGCUUAAACUGCCAACCCUUCUUAACCAUUUCCUCCGUUAACAAAGCGGCCAAGAGAUAGGAUCUCUUGGUGCUCUGUUUCAUGGCUGAAAUGGGGAAGGGUGUUAUCUCCUGACCCUUGUGGAUUCACCUCUUUGCAAACUCAAGCGUAGUCUUACUACGGUGAGUUUUUAAAGGGGAGACAUCCACACCAAGGUCAUUCAUAACCGACAGAUAUCGACUGGCAACUCUUCCAUCUCCUAUCACAAUAUCGUCUCCUAGGAGACAAUAUGGUAAGGAUCGUCACUCAACACCUUCUAGCCUACAACAAUAGUAGACUAUAUAGUGAUGGGCGACAGCGAAGGAUGCUCAUGAUGAGUAGAACCCCAUAGGGUUACCAACCUCGUAGCGAACUGGCUAGUAAGCCUUCUCCCCACCAGAUCCGGUACCCACCAUGAAGGGUUCCCCCACCAUAAG  >Ss-AA_clean.1_(paired)_contig_896  UUGUGGAUUCACCUCUUUGCAAACUCAAGAGUAGUUUUACUAUGAUGAGUUUUCAAAGGGGAGACAUCCACACCAAGGUCAUUCAUAACCGACAGAUAUCGACUGGCAACUCUUCCAUCUCCUAUUACAAUAUCGUCUCCAAGGAGACAAUAUGGUAGGGUCCGUCACUCAACACCUUCUAGCCUACAACAAUAGUAGACUAUAUAGUGGUGAGCAACAGCGAAGGACGCUCAUGAUGAGUAGAACCCCAUAGGGUUACCGACUUCGUAGCGGAUUGGCUUGUAAGCCUUUCCUCUACCGGAGUUAGUAGCCACCAUGAAGGGCUCGCCCACCAUAAGAUCCUUCCAAGCCUUAACAUAGGAGGCUGGAAGAUGGCUGGAAAGCACCAACUCGAUUACCUUAAUAGGGAAUCGGUCGGUGGCUGAAGUAAGAUCAAUGGAAUGAUAUUCCGUUGCUCCCUUCAGACCAUCCAUAAAUGAUUCCUGGUUAAAAGUGAAGUCUUGCGGGAUCCUUCUUAGAACUCUGAAUAAAUAAUUAUGCAGAGCUCUUAGAACGGUUUGACUGAAGUAAUCACCAAUGGCGAUUACCCUAGUCUUACCUUCCCGCUCUGGAAAUGACGAGAUUCUCCUUGUAAAGGGAAUUUCUCGGAAAUCCGCACGGUGGAUCUCC  >Ss-AA_clean.1_(paired)_contig_649  UGCUCUGCUUCAUGGCUGAAAUGGGGAAGGGCGUUAUCUCAUGACCUUUGUGGAUUCACCUCUUCGCAAACUCAAGAGUAGUUUUACUAUGAUGAGUUUUUAAAGGGGAGACAUCCACACCCAGGUCGUUCAUAACCGACAGAUAUCGACUGGCAACUCUUCCAUCUCCUAUUACAAUAUCGUCUCCAAGGAGACAAUAAGGCAAGGUCUUUCACUCAACAUUCUCUAGUCUACAACAGUAGUAAACUAUAUAAUGGUGAGCAAUAGCGAAGGACGCUCAUGAUGAGUAGAACCCCAUAGGGUUACCGACUUCGUAGCGAAUUGGCUUGAAAGCCUUCUCCCUACCGGAGCCAGUAGCCACCAUGAAGGGCUCACCCACCAUAAGAUCCUUCCAAGCCUUGACAUAGGAGGCUGGAAUGAGGCUGGAAAGCACCAACUCGAUUACCUUAAUAGGGAAUCGGUCGGUGGCU  >Ss-AA_clean.1_(paired)_contig_2035  UGUUGUUAGUCAGGUAGGCUGUUUUGGAGAACACCUUCGUUAAUAGCGAAGGCAUUCUUCUAAACUGUCCAUACCAUCUUUCAACCAUAACGUCGACAGGCUUAAACUGCCAACCCUUCUUGACCAUCUCCUCUGUCAACAGAGCCGCCAAGAGAUAGGAUCUCUUGGUACUCUGUUUCAUAGCGGAAAUGGGGAAGGGCGUUAUCUNNGACCUUUGUGGAUUCACCUCUUUGCAAACUCAAGAGUAGUUUUACUAUGAUGAGUUUUUAAAGGGGAGAUAUCCACACCUAGGUCAUUCAUAACCGACAGAUAUCGACUGGCAACUCUUCCAUCUCCUAUUACAAUAUCGUCUCCGAGGAGACAAUAAGGCAAGGUCUUCCACUCAACAUUCUCUAGUCUACAACAGUAGUAAACUAUAUAAUGGUGAGCGAUAGCGAAGGAUGCUCAUGAUGAGUAGAACCCCAUAGGGUUACCAACUUCGUAGCGAAUUGGCUUAAACGCCUUUUCCCUACCGGAGCCAGUAGCCACCAUGAAGGGCUCACCCACCAUAAGAUCCUUCCACGCCUUGACAUAGGAGGCUUGAAGAUGGCUGGAAUGCGCCAACUCGAUUACCUUAAUAGGGA  >Ss-AA_clean.1_(paired)_contig_1377  CUACUGCUAUUGAUGCGACAUCGGUAUUCCUGUUACGGAACACUGAGUCAUCCAGGGGGAUGGUCAUUGCCUUUAACAGUAACGGUCAUUCACCUGCACCUAUGGUGUCAAUUUCGCGAGCUUUCUUGAUCAAAGUCAAGUAUUGCUCCUCAAUCGACCCAUAGGCAUGCAAUAGCGGAAUAUUCAGGAGAUCCAUCAUUUGAGGAUGGUCCCGACCAGUGAUUUGCAUUACUAAAGUCUCUGCCAUCAAACCUAACGGUUUUCCGGUAGUGGCUUUAGAAUCAAAUCGAGCGUUGGACUGACUAAAAACUUCCACCAUGAUGCUAGCAAGGAUAUUAGUCCCUACUACAUCAGGAAUGGGCUUUAGGAUGAUCCCUACCCGCCUG  >Ss-AA_clean.1_(paired)_contig_3142  GCCCGUUAACUCGGGAUCACAACCUCCUCAACUUAUUACAAAAUACACUAGAACAACAAAAUACAAAUGAUUUGUCGCACUAGUUUGUAAUACAUUGAGUUGGAUCAUUUGGGCGUCAAGCCCAAAUGGAUUGGCUCUACGGGAUUUUCAACCCUUCCUUGGGCACAUGGAAGCAGUUCUAAUAAAUCGAGGACCGCGUGGUCUCAUCGAGAUGUUAAAACUGGUUCGUAUGUGUGUAGUAAACUACUUGUCAGGUAGUGAGCUACGUCCCAAGGGAGUUAGGUUGGUCACGAAGGCUAGGUUGCCUUACUCUUUAGGCCCUCUUAUUAAUAAGGUCAUAGAUGGCGACCCACU  >Ss-AA_clean.1_(paired)_contig_958  AGCCCCCUGAAACUCCCCUUGAACACCUUCAAGGGACUCUCUUAGACGAGAUAUUUUUACUCUUGUCUACGACGAGUGCGAUCUAUUUCCAUUCAGACCUAGUGGUCUUUUCACCGCUAGGAACCUGCUCUGGAGUCGUAUUUUGUGUUACCUUCUUAUCAGUUCAUCACUGUGCAGAAAGGGCCGGAAUAUCACUUGAUAUCCCAGUGGAUCUAAAUCCAUACACAUUAUCACCUCUCCAGGGAAAGGAGAUCUGAGGAAUAGAAAGGUGUCACCCUCCCUAUCCCCCCACGCGCCAUAGUUGGUCCGAACCAAUAUUACACCGAUGACUUAAGAUCAUCUACGCCCUCCUUGCUCAAAUGAACAAGAAGGCCGCAGAUUUUCUUAAUGUCUGGGUCAACAGUAUCUAAAUUGUUAGAGCAUGACUUAUAACUAAGUACAUCUCGAAGCAAGGUAAGAGACUGGAAAACACUCUCUCAAUUUCUUAAGAGAGAAUUCCGAGCCUUUACUUUUUGUAGAGGUGAUUGACCGUUCGCAGCUGAUAAGCUUUUAACAUCUAGAAAAUAAAUUUCUAGAAGCUUGUCAGUCGGCAUUCGUGUAAGGUCCGAAAGAGGUGCAACAGGAGAUUGAAUAGUAAGAAGUAGUCGGUUUAUAACCGAACCACUCUGGGGUGUUAAACCUUUCUUAACUGCUGAGGCAAGAGAAGAAGACCCAAAAGUCGUCUUCUGCAAGCGAUCAGCCAGUUGUACUUGAGGAAGGGAUGUGAACUUAUCAGCUUGGAAGUAAUAACCAGGUGGCCCAUCUUUUUCAAGAUCAGCCAUCUGCUUAACCUUCCCGGAUCUCGCAAUAAGAUCAAUGAUCAGAUUACGAAGAUCCGAGUCUGAUAAAGAAGGAACAAAAUGUCUUAAAAAGACUCCAGGAUCCGUCUUACUAAACGUAAGAACAGAAAACUGAAGGGCACGGGCCAAUAAUGCAUAAGCAGUAUUGUUAUUCCCGGCCGAUUUUGAACCACGACCCAUAAGCUGCAUAAGCUUAUAGGGCGACACAUCCAACUUCCGAAGUAAUCCUAAAAAGGAUGAGAAAGUACUCCCGAUGGACGAGAACUCUUUCAAUGAAAGAGGACUCGCAUCUAAACCAUCUACAUAAAAUCUCUUUGCAAACUCUAAGGCUAUCGGAGACGAUAAACCCUUGGCCAUGUUGAUAUCAACCCGAAGACACACUGUCAUCAAGUAAUGAUAGACGUCAGCAACCGACUUAUCGGCUAUGACGACAUCAUCACCUAAGACAAUGUAGUCUUUGAACAGCACGACCCGUCUAAAAGCUAGACUGGCCGCGUACUGUAUCACCAAAUGGUGAGUUAAUGCCAACAUACCCCAAGAGCUUAAAGCUCCCAUAGGCUGACCAACACUAUACUUAACUACAUACUUGACACUCUUAUAAUCAAGCAUGUAGUCCCGUCCUACCAACAGACGAGCCCAGGCAACAGCAAAAUCCCGAUUAACUAAAGUAGUUAAAAUCGAGACUUGCAGUUGCAGAGGCAGCCUGUCAGUAGCCGCCGAUAGAUCAAACGAGUAAAAGUAGGAGCCUGGAUGGGCUUUAAUAAAAGCCUCCAGCCUACCUAUCUGAUCGAAAGUAGCAUCGAAAGGGAAGAAUUUAAGCCAAGCAAAAAUUGCUCGGUGCAAUGGUUGAAGCAGUCACUGAGUAACUGCAUCAACCAUAGCAAAAAUUCUAACCUUUCCAGGCUCUUCCUUCGCAGCCAAUUUUCCUACGAAAACUGACCGCGAGGUAUCAGGAUCGAUUGACGAGUAGUACUUCCAAAAAGGAGACUUCUCUCAACGACCACUCGAAUAAGGAUCCAAUAUGCUUAAGAAGACAUCCCAUGAAAACAUGGACUUCAACUUACGCAUAGCGGUAAUUCAACCAGCGGUAUUAUUCUGACCCUUAGGACUACAUGGCCCACUUGAAUGAAUCAAGAACGGGUUAAUGGUUUCUCGGAUUCGGAACGGAGUAGGGAUUAACCCACUCCGGACGAAAUCUGAGAACCAACCAUCGAAGUCAGAAGGGAGAGAAUAAGCGCUAGGGCUUGUGAUUGUCGAAAGCUUAAGAGAGCCUUUGAAAUCCAAAACCCUAUAAAGGUUGAACACCGUCAAUACCGCUUUAACAAUUUUCAUGUCUCGUUUUCGGAUCAUCAAUCUGAAAUAACUAGGUAUAAUUCUAGGUAUUCCAGACCGAGUCAACGAAACCAAGACACCAAAAGAGUUAAUAGCAAGCUUAUUGGGGUCCUUAGCCACAUACUUCAUUACAUAGAUAGAGCAAGCUUUCAUGUAUAGACAUACAUACGAAAACCCACUCUGUCUCUGUAACGUGGUUAGGGAUAUAACUAAGUUAUAAUACGCCCUAAUUUUAGAUACCGUUGUGUCACCCCAGGUCACUUUAAGGAUUACAAGUAAUCCUUUAUUGAUUCGGUAGAGGACUUUCGUUCCUCUAUGCCAAGAGUACAAAUUCUUCAUUCUAAAGAGUGUUGCUCGCAGUAAUUUAAUUAAAUUAUUGUUGACAAUACUCACAGAAUAAGUCCUUUGCACUCUCCCCCGUCAUCCAUUUCUGGAUCGGAAGGCAGGUAUACAACCGGAACUAAGGUUAGUUCCCCCCGUCAUGAGAAGCAAGAAGAUCACUCUUCCUCUCCUCACUGCAGGGAAUUAUAAUUAGUAUAUAGCGCAAUGGCCAUAACUAGAACAAAAGGGCUGUCAACCCUAUUGAUCUAUGAGGCUGCGAGACCUCCAAAUAUGCACCACCAACACCAUACCUAUAAAAUAAUGUAUUUUAUAGUUAGAAGCUCCUCUUAGACAAGAUAACAAGUAUCUCAUUUAAGAGAAACCCGGUAACUUUACCGGGGUCCCAAGGACUUACGUCCGAAUCG  >Ss-AA_clean.1_(paired)_contig_867  GAGACCUGUUAAGGUCUCUCCCCCACCCCGGGUUUUCCACCCGGUUUUUAUGACAAUGCAGUUUAGUACACUGUCGGAGCCCUCUAAAGGGGAUUCCUUCAAGACUCAUGUAUUAUUCAUACAUGAUGAACUUACGUUCUACUAAAUAACCUGUUCGGCUGAAUCCUUGAGAUGAAGCUAAUCAGGAGUAUACCGGCGAUAAAGUCUCCACAGAAGAAGUUCUCUUCUGUCGGACGAUAUCGAAACGCAUUCAUCAGCGCUCAACUCUCUCUGCAAUAGGGAGAGCUUGCUCUAAUGAAAGCCGCGGAUACUUGGCAUGGUCAUGAUUGAUUUUGUCAAUCUCCUCUACCAGUUCGUACAUAUCAAACUUGUCACAAUUAGUGAUAAGUUCGGUAACUCAACCAUCAAGUUGAGCUACUGCGAUUGUACCGAUAGGGAGAGAAGCAAAAUCGACCCCGUCUUUUACAAGACACCGAGCAUAGGCUCCGGAACCGGUCAAUUCAUCAUAUGAAUUUUCCAGUUCCUUCGCCCUCUGCAAGGCUCUUAAAAGGACGGACGCAGUAAUGUCAGACUCUAGCUCAUCAAAGAGUUCUUCACGUUCUUCAACUGAAGAAACUCUCAAUAAGUCUAGUUUCUCGACUUUACUGUUUAACAAUUCCUUCUGGGCAGUCAGGACUGAUAACAAGGGCAGACUGAACUUCGAUGUAUUAAAAUCGAAUUCGUCAUCCUUGGGAUCAAUCAGGACCGUCACUAACGCCUUUAGCGAAAUCUUUCCACUUUUGAAAAGAGAUCCUAAUAGGGCUAAUGAAGGUAACAUCACAGACUUAACGUCUGUUUUGUAAAACCGACCCAGAAGUGUUGAUAAAAUAGUGUUUGUUCGGAUAAGACCUAGACCAGCGAAAUGUAAAACAUUUCCCACUCUAGCUCCCAUCGAUGUAGCGGAAAUCAGUUGUUUAACUGAUAUCCCCGAUACAUUGAUCCCUUGCACUACCGUACGUUUCGCAAACUCAAAUACUGGACGAGACGGAGAAGAUAUAGACUUAGAAAGAUUAAUCUCUAAGCCGAUCUUCUUCAUCAACUCCAAGUAUUCGGCUGCAACUGCGUGAUCGAAGAUAACCAGAUCAUCACCAAGAAUUUCAUAAUUCUCGAACCAACCCACAGAAUUGACCGCCCUAGAGGCGCAAUACUGAAGGAGGUAGUGAUGAGUCAAAGCUAACAUCGCUCAUGACGAUAAGGCCCCCAUGGGUUGCCCAACAGUAUAGGUCAUAGAUUUCUCUGUGAUCCCAUACUUCUUCGCUCCACCCGGUAAGUAAUACUCCCGUAGUGUCAAUAAACCCGCUCAAGCGUUUCCGACCUUUACAGGUAAGAUCCGAUCAAGCAGGGCGGAUUGAUAAACUACAGGUAAACGAUCGGUGGCUGAGCUUAAAUCAAAUGAAUAAGCACAGUCAGCGACCGAAGCCUUACUAACCGACCGACGCACAGACGCAUCCUGGUCAAAAGUACCAUCAUUGGGGAUUAAACGAAGUAAAUCGAAUAAUGCCGAAUGAAGGGGCUUGAGAAGGGAUUGUGUCCAUACGUCGACCAGAGCAAAGACCCGAAGUUUUCCUGCAGCCUCCUCUUUGAACGAUAGUUGACCCAAACAAUCCACAGAAGAUUUCUUCUGAUUAAGAUUAUUCGGACCAACUAGCGCGGAAAGAGAAAGUGCCUCCUCAAAUAUCUUCUUAAAAUAUACUGCUUUGGUUAAAGCAAUAUACUGUACAAAGAAUUUGAAGACCUUCGAUCGAGACAUAGCAACAGCGUCUGUUAACAGACCGUGCCAUGAAACCCGAUUAGAAGGGGAAGAACUCUGGAUAAAGGCAACAGCUUUUGGAGUAAUAUUGACCGACUCUCGCCAAGAGGAAUACUUUUCUAAUCUAGAAAAGGGAUUCCCUCGGGGAGCGUCGUCGAUAUAAUCCAAAAGCCCCCAUGAGAAAGCUUUAUCACCUGUAUAAUCCCCAUAUAUAGACUGAAGCUUCGGAACGAAUUCCGAUAGCGUCACUCUAUAUAAACUAAAACAAGACAGUCAAAACUGUAUUGUCUUAGUGCACCCCCGCUGAAUUCGACGGCGAUCCCUCUUAUUAAUAAUAGAAGGAAGCCCCGAAUAAAGCCGAGGUAGAGGGAUAUUAGGUUCUAAGCCUCUCAAAGAUUCAAGUGAAUCUCCCCCUAGGAACUUUUGAAGCGCUACAUGAUUCGCCUUAACCCACUUAACAGUGGUCAGGGAACCAUGAUCACGCUCAAAAGUAACUAGACGAGAGACCAGGUUAUGUAGAAUUUUAGCUCUUGAAGGGACAGAUGAUGGAGUUCCAUAUGAAAGCAAUAUUAUUGCUCUCACAUGCAACUUUACACCCUCUCACAAACGUGAGACGGAAAACAUCUUCUCUCGAGUUACUCCGAAAGCGCCCAAACUCUUAAGAAUUGCCAUAAAAGAAUUAUUUUUANNNNNNNNNNNNNNNNNNNNNNNNNNNNNNNNNNNNNNNNAGGUAACCUGCGUCACAGACUCGAAGUCUGUGAUCGGCGUACACCGGGCCUGAAGAGCUACUGAGAUCGAAACAAGACUGCGCUGUUCCUCGUAAGAGGGACGCCAGUUCUCGGACCGGUCAAGGCGGGAUCACUAAGAGUGAUCUAGUUCUACCGAACCACAAAACACGACUAUCACACUUCAGGUCAAACUCGGGGCUCUCGAAAGAGACCCCCGGGGCGAGGUUCAGUGUAACAGCUGGGCGAUC  >Ss-AA_clean.1_(paired)_contig_536  CGCGAGAGUCUCUAAGGGCAAUCCACCAUCCCUUCCCUUUCAAGGAAGUAUUCGUGGACCCUUUUAACUGAAAAUUCAGUCAGAUAUGAUCAUCAUCUCUGAUUUAGCAUACUCUGAUGAGCCUCCAGCAGGAUCUGAGAGUCAGAUAACUGACCUUCCGAUCUAGAAUCGUUAUUUUUUUGAAUCCGGUAUUAUCCGGAUUCCAAAAUCUAACUUUCGACCACCUCCCGGUCCGAAAUCAAGAUUUAUCUUGGUUCCGAAAUCGAAUUGUGGUUUAAAAGUUGGGCGUUCUUCAACAUAUGCUUCGGAUUCAAUGUACUUUCCGUCGAAUUUAACAAAAUCCGACUUCACGAAUCAACGAGGAUCAUUAAUAUCUUUAAUAAAGUCCAAGACUUUUAAAGGAUUGUCAAUGAUCUCCUUAUUGAUCACUGUGGGUGUAUAAAACACCAAAGUUUUCUUGAUUCCCUCUAGCUUCUCCAGAUCUUGCAACAGGGCUGAUAAUUCCAAAUCUACAAAUACCUGCUUAUUAUAAUAAUCCUUAAGAUUACGAUAAUCAUUCAAGUAUUUCAAUUUGAAACCAAAAGUCUUCAUUGCAAAACCGGUUCCCAUACUUAGGUCGACAUCAAGACCAAGUCUUAAGUUUCGGAUGUUAACAUAUGACCUUCCUUUAUCAAGGAAGAAUAUGUCAGCCAUCGGACUUAACAUAAAGAACUGCUUUCGGAAUUCUCCGAACUCAGGUCCUUGUGUAUAAGCCCGCUCGACGAAAGGCUUGUCCAACUCUAGAGGCUUACCAGACGCCUUUCAAGCGUCCAUAUAAGAUUCUAAAGCUGGAGUGGUAAUGAUCUCACCUAGUAUAUUAACUCUAAUGGCCAUCAAGUCAGUCUUGUCCAGCGACUCGACCAUCCUGUCGAUUCGAUGCAACAAGAUCCGCUUGAAAGUUAUAGAGUUAACCUCAGCGAAAAAUCUCUCACGCAAUGGAAGCUUUGAGGUUCUAAUCUCACCGCUUUCAAAGUAUGAUUUCAAUACCUGAGAAAUAAGCUUGGGUUUCAUUCAGGAGAUAUUUCUCCCGAAGAAAGCCAAAGGCUUAGUUCUAUCAGUUAUCAGAGAUAAAACGCUGGAUAAGGGAAUCACUUUAUUCUGAUAUAGCUGUGUAAGAUAACCAACCAUCGGAUAUAUCAUAUCCAGACGAUUUUUAUCGUCUUUAGAUAUUUUACAUCCUAAAGUUAGAAUCUUGAAUGGAUCUUUACCUCACUUAUUUCGAAUAAGCCGAGAUACCACUGCUAACCGUCCGAAGAAACUAUUAGAAGAUAAUAGUUCUUUGAAGGGUAAAGCAGAGACAUCAACAGCGUUAAUCGAAGUCCGUUUGGCAAACUCCAACACAGGGCGUGAUUCAGCGAUAAUUGACUUUGUCUGGUUUAUAGAAACCCCAAGCUGUUUACACAGCUCAAGGUAACGAUAAGCCACGACUCUAUCAAAUAAAGCCAAAUCAUCCCCUAAAACUAUAUAAUCUUUGUACCACACACCCGGCUGCGUUCUCCCGGUGUACAAUGCGCAGAACUGAACCAUCAUAUGAUGAGUUAAGUUCAGCAUUGCCCACGAGGACAAAGCACCCAUAGGUUGUCCAACCGCAUACCGAAGACUACCUUCUGGGAUUCCAUAAUCAUUUUUAGUGAUUACAUAAUCUCGAUUCGUUAGAAUCUGUCCUCAAAGAUCACCGACACCGAACAGAGCGUUCAGUACGGCAAUCUGAGAAGACAAGGGUAACCUAUCAGUAGCAGCGGACAGAUCAUAACCAAAGGAGCAACCAUACUUAAGAGAAAGCUCUUGAGCGUACUUAACUCCCUUGUCCUGAUCGUGCGUACAAUCAUUCGGUAGUUUCUUGAAAAGGGCAAACAGUGUUCGGUGUAAAGGUUCCAGCAAGGAUUGAGUAAUUACAUCAACCAUUGCGAAAACCCUUAACUUCCCAGCUGCUUCCUCUUUGAAAGAAAGCUUACCAAGAUGAUUGUGCUGUACUUUAGAUUUAGCACCGAAUCGCGAAAUGGCGAAUUCGAUGUUAGAGAAUAAAGUUGAAAAAGCCAAAGAAUUAGUCCUUGAAAUGUAAUCCUGCACCGCUUUGAAAAGAUCUUUUGAAUCUCUCAAACCGAUGUAGGAUCCCACCAAGUGACUAUAACUCCGUGGUCCCUGAGGUGAUGACUUAACAAUAGGUAGCAACCUAUUGGCAGUCAAAUCUCGAAUAUCCAUACUAAGAAACUUCUGUAAUAGAAGGUUAGAAUUAUCUUUCAACCAACUAUUGAAGUCGCGAAGGUGGAUAUUGGAACCAUUGAAACUAUCAGUAAUAGUCGAAAGUUUUGGAUUGAAAGGAAUCUUUAUCACUCUAUAAAGAGAAAAUAAAGAUAACCAAAAUCUUAUAACUCUGUAACUAUUAUUACAAAUCGAGGCCCGAUCAGUUGUUUUAAUAACUGAUGGAAGACCCGACUUUGAUAGUCUCGGGAAAUUAUAGUCAGGCUCAACUUCUCUCAGUGAUGAGAAAGGUUGACCAGCUAAUUUCUUUUGAACAGCAAGCUGACACGCUUUUAGAUACUUAACUACAUACACUUCUCCGUGAUUCCUAUUCAUCUUAAGAAUAUGAACACCGAAGUUGUGUAGCAUUCUAAAUCGGGAAGUCUCCUUAGUGCUCAAGAACGACAAGACGGCAACACGCCAUCCAAUCGUCUUCAACACUAACAUCAAAUGUUUUGCAUUUGAUAGUGAGAUCAUAGAACCUGCUUUAUAGACAUCUGAAUAUAAUUUAAAACCAGAGAAGAAACCAGUAUUUUUUUGUGAAAUAUUAGUUUUCUUCAUGCUUUUAAAAAAUAUGUGUUGCACACCCAAGCAAGGGAUUAAAUCCCAAAGCUCCUCUUAACCUUGGAGUUUACUUGAGUGCUCAGCGUCAGGUGGCCUGCGUCAUAGACUCGAAGUCUAUGAUCGGUGCCCGCCAAACCUGGAAGAAAAGAAACUUUCUUGUAACCAGAUGGCUACAAAGCAGCAUCCUAUUUCCGCUGUUCCCGUUAGGGGACGGCAGACCAUAGGGUACCACCCGCUUUACAAUGAUCUAAGUGAUCAAAGCAGUAAAGCUAUCGUUGUUUAACCAUGUAGAACCAGACAGAGUAAAAUCUUAAUCUGGCGACACAUGCGUUUUUUAGUAUCCCGUCCAAGGACGGAGAUCCAAUAAACUGGUUAAAGGACUGAUUAAGUCCGGAUUCCCAGAGGGGCUAACACCCCA  >Ss-AA_clean.1_(paired)_contig_59  CUCGCUUUAGCGGAUAUGACACCCCGCUUGAGCAGCCUUAAAGGGGUAGCGCCCGAUGGCGCAGUUCUUCUCUCACCGCCUCAAAGAAAAUUCUUCCUUUGAAGCGAGAUGAGAAGGCGUACUCCUCAAAAGUCUGUCGCACGAAGAGGUCACCCCCUCCGAUGGAAACUUCAAACGUUUGAUCAGAGAUCAAAUGCUUGAUGUAUGCGACAAUCUCUUGUUCAUAGCUCGCCCUAUAAUAAUCCAUCAUUAUUAAGGGACCUCCAAAGACCAACCGUCAUCGGGGAGCAGAUGUUCCCAGAGACUGUAAGUCUAAGGAGUUCUGGUGUAUGAGAAGCCUCCGAACAGGAGAAGUCUCAAAACCAGACGGAUAUGACGAGAAACCGACGGGGUAAUAGAGUUCGGAUAUAGAAAUCUGAAACUCUAUUGCCUUCCGGACCGCAGUUUGUCAGGUAGAAACAUCGAAACGAUGUUUCACCCUCCGAACUGCGUCAAUAAUCUGAUUUGCUCUAACCGGAGUUAGCGAGUUAGACAUCGUUAAAAACGAUGCUAACCCUUCUCGGGUUGGAACAAAACCGAAAGGUCCUUUAAUAGUUCAAAGAAUCAGGUCCAACUGGCUUUUACGCACAGUCGGAACCCGACUAAUUAAACUAUCGACGGUUUCUUCGGUGAAGGUAACACCCUUGUUGCUUAAGUCGCGAAGUAGAGAUGGAACUCCACUAAGGGACUGAAGAGCAACUAGAGCAUUACUUGCACCCAAGGGCGUUAGCUCAACAUUCUGCGACACCAGCCUCUUAGCAAACUCGAAAGAGUGCUCCGAAACUAGCGUCUUAGACAUGUUGAUCUUAACUCCGAGGAUAUCGACCAUGAUACUAUGGUAGGAACUUGCGACCAGAUCAUUAGCGAUAACUACAUCAUCACCGAGCACGGCGUAGUCACCAAAAUCGAGUAAUCCUACUCGACGGGCGGCUAAACGUACCACGUAAUGGUGAGUUAGGGCUAACAUACCCCAUGAGCUUAAUGCUCCCAUAGGUUGACCAACGCAGUAGCGAAACAGUUCUUCCUCAGCUGGAAGCCCUGGUAUCGUUUCCACUUUGGCCAUCCAAUCUCUAUCGGUCAUAACUGACUUUCAAAGAGAACCGAAACGCUCACCCAAGUAGAUAGACAGGAUUUGCACCUGCAGAUCUACCGGUAAGCGAUCGGUGGCAGCACUAAGAUCAUAGGAGUAAAACUUCUGACCUUCCAGUUUACCCUCUGACUUCAAGAGCCGUUGGCGAUCCAAUGGGGCACUUUGAUUAAAAGUACCGUCCUGAGGAACCCGCCGAAGGCACUUAAAGAUGGCAUCAGAUAGUGGAGCCAUCACCGACUGCGUUACCGCAUCAGUGAUCGCGAACACCCUGACCUUUCCUGCGGCUUCCUCCUUAAAGGAUAACCGACCGAGGUGCAACACCUUGGAAGGAAGAUCCCGUAACGCGGAAACCUCAGCUAAGAGGGCUGAACGAAACUCCGAACCGCCAGGGAAGAAUUUGAUAAAAUCAAGUAAAUCCCCGAAGUUAGGACGAGAGGACCAGGCCUUAAUAUCUAACCAGAUACCAAGACCUGCUACCUUACAGUUCGGCCCAGCCGAUCGAGAGAACAGCCAACGGAGCGGUCCAAAAUCUCCCAACAGUGGGAGGGAGGACUUAACCGCUAGUAGUUCGUAAACGGGAAGAGUGGAAGAUAACCCAGUGAAGGGACCCACAAUAGUGUCGAAGCUUACGUUACCGGGGAACUUAAUCACUCGGAAAACCGAGAGACAAGUUAAGACUCCUCUAAUCACAUCAGGGUCUCUGUCGUGCAUAAGCAGACGGAGGUAACCUGGAAUGAUCGAGGGGAGCCCCCUAACGAUGGAAAGCGGUAACUCGUGAGUAACGAGUACCGGAUUUCCCGACACGAAGUGCUGAACGAUCCGGUAACAUUCUUUCAAGUACAACACCGUGAACUUACGUCCAUUGUGAGUCAAGAGAGAACGAAUCCGGACCGCAAACUUCAUGAAGUUACGCUUGUCCAAACGAAGUGCUCAGAUCAAAAGUCGGAAAUACUGAAAGAAGAGAAACAAAUUCAAUUCCUUAGUAGGACCAACUUUGCUGAAACGACCCGCUUCAUGUUUCCAUGAAGAGAAAUUAGUUUUAAAUUGAUUUUUCAUGAGAAAUAUUAAAUUUGUUGCACAUUCCUACGGUUCAAAGAACCAUGCCUUUAGACAGGCCCUCGGAAUGCUCAGCGCGAGGUGGCCGGCGUCACAUACACUAAGUAUGUGAGCGGUGCCCGCCUCACCUGUAACAACUUAAAACUCAGAGAAACCAACCAAACACUAAUCCUACCGGACUAGAAAUCUGGUCGAGGUCUCAGGCUCUAGCACGCCAAAGGCGCUGUGACUUCGUCCUAGGACGAUCACCGCUAUUUGGGGAUGAGCUUUGCUCCAACCAAAACUGGUGUCCAAGGACACCCCGCACGUCCGGAAAGACGUAAGGGCAUGACCCUGGAUCAGCUUCGGCUG  >Ss-AA_clean.1_(paired)_contig_39  UUCCGAUCUGUAAAACUUCUGACCUUCUAAUUUACCAUCCCUCUUCAACUCUCGGAGACGGUCCAGGGGGGCACUUUGAUUAAAAGUACCAUCCUGGGGAACCCGUCGAAGACAGUCGAAGAUCGCGGCGGAUAAGGGAGCCAUCACAGACUGCGUUACCGCGUCAGUGAUAGCAAAAAUCCUUACUUUCCCCGCAGCUUCUUCCUUAAAGGACAACCGACCAAGGUGAAGAACCUUGGAAGGGAGGUCUCUAAGGACAGAGACUUCGGAGAGAAGCCCUAAUCGAAAAUCCUCACCAUCAGGGAAAAGCUUGAUAAAAUCAAGCAACGACCCAAAGUUAGGACGAGAUGACCAGGCCUUGAUAUCUAACCAGAUACCAAGACCGGCCACCUUACAAUUAGGUCCCGCCGAACGGGAGAACAACCAACGGAUCGGUCGCAGCUCCCCCAAAGGAGGGAGAGACGACUUAACCGCUAGAAGUUCAUAAAUCGGAAGGGUAGAAGAUAACCCAGUGAAGGGACCCACUAUAGUGUCGAAUUUAACGUCACCGGGGAACUUAAUCACUCGGAAAACCGAGAGACAAGUUAACACUCCUCUAAUCACACCAGUAUCACCGUCAUGCAUACGCAGACGAAGAACACCGGGAAUGAUCGAGGGGAGUCCCCUGACUAUGGAAAGCGGUAACUCGUUAGUAACGAGUACCGGAUUUCCCGACACGAAGUGCUGAACAAUCCGGUAACACUCUUUCAAGUACAACACAGUGAACUUAUGUCCAUUGUGUGUCAAGAGAAAUAAAUUCAACUCCUUCACAGGACCGACUUUGCUGAAACGACCCGCUUCAUGUUUCCAUGAAGAGAAAUUAGUUUUAAAUUGAUUUUUCAUGAAAAUUUAAGAAUUGUUGCACAGUCCUACGGUUCAAAGAACCCUGCGUUUAUACACG  >FirsU_Contig49  GACCAGAUCAUUAGCAAUAACAACAUCAUCACCUAGAACCGCGUAGUCGCUGAAGUCGUGNAAAAGAAACACGACGAGCAGCUAAACGGACUAUGUAAUGGUGUGUUAACGCUAACAUACCNCCAUGAACUAAGGGCUCCCAUGGGCUGACCAACGGCAUAACGGAACUGCUCUUCCUCAGCNUGGAAGACCAGGUACCGUCACCACCUUGGCAGACCAAUCCCUGUCGGUCAUCACUGACCGNCCAGAGAGUACCGAACUGUUCACCAAAGUAGAUAGACAGGAUUUGCACCUGUAAGUCUACNGGGUAAACGGUCCGUGGCAGCACUUAGAUCAUAGGAGUAAAACUUCUGACCUUCUAAUUUNACCAUCCCUCUUCAAUUCUCGGAGACGNGUCCAGGGGGGCACUUUGAUUAAAAGUACCAUCNCUGGGGAACCCGUCGAAGACAAUCGAANGAUCGCGGCGGAUAAGGGAGCCAUCACAGACUGCGUUACCGCAUCAGUGAUAGCAAAAAUNCCUUACCUUCCCCGCAGCCUCUUCCUUAAAGGACAACCGACCAAGGUGAAGAACCUUGGANAGGGAGGUCUCUAAGGACAGAGACUUCGGAGAGAAGCCCUAAUCGAAAAUCCUCGCCAUCNAGGAAAAAGCUUGAUAAAAUCAAGCAAUGACCCGAAGUUAGGACGAGAUGACCAGGCCUUNGAUAUCUAACCAAAUACCAAGACCGGCCACCUUACAAUUAGGUCCCGCCGAACGGGAGAANCAACCAACGGAUCGGUCGCAGCUCUCCCAAAGGAGGGAGAGACGACUUAACCGCUAGAAGNUUCAUAAAUUGGAAGGGUAGAAGAUAACCCAGUGAAGGGACCCACUAUAGUGUCGAACUUNAACGUCACCGGGGAACUUAAUCACUCGGAAAACCGAGAGACAAGUUAACACUCCUCUAAUNCACACCAGUAUCACCGUCAUGCAUACGCAGACGAAGGACACCGGGAAUGAUCGAGGGGAGNUCCCCUGACUAUGGAAAGCGGUAGCUCGUUAGUAACGAGUACCGGGUUUCCCGACACAAANGUGCUGAACAAUCCGGUAACACUCUUUCAAGUACAACACAGUGAACUUACGUCCAUUGUGNUGUCAAGAGAGAACGAACCCGGACUGCAAACUUCAUGAAGUUACGCUUGUCUAACUGAAGNUGCUCAGAUCAAAAGUCGAAAGUACUGGAAGAAGAGAAAUAAAUUUAACUCCUUCACAGGNACCGACUUUGCUGAAACGACCCGCUUCAUGUUUCCAUGAAGAGAAAUUAGUUUUAAAUUGNAUUUUUCAUGAAAAUUUAAGAAUUGUUGCACAGUCCUACGGUUCUAAGAACCCUGCGUUUNAUAUACGCCCUCGGUCUGCUCAGCGCAUAGUGGCUUGCGUN  >Ss-AA_clean.1_(paired)_contig_50  ACUGUGUUGUACCUAAAGGAGUGCUACCGAAUCGUUCAGCACUUCGUGUCGGGAAAUCCGGUUCUCGUUACUGACGAGUUACCUAUUUCCAUUGUCCGGGGGCUCCCCUCGAUCAUUCCCGGUAUUCUCCGUCUGCGGAUGCAUGAUGGAGAUGCCGGCGUGAUUAGAGGAGUUUUGACUUGUAUUUCGGUUUUCCGAAUUAUUAAGUUCCCUGGUAGCGUGAGCUUCGACACUAUAGUGGGUCCCUUCACGGGGUUAUCUUCUACUCUUCCAAUUUAUGAAUUGUUAGCGGUUAAGUCCUCUCUUCCCCCUUUAGGGGAACUAGGACCGGUCCGUUGACUUUUCUCCCGUUCUGCGGGUCCCAAUUGUAAAGUGGCCGGUCUUGGUAUCUGGUUAGACAUCAAGGCCUGGUCGCUUCGUCCUAACUUCGGAGAUUUACUUGAUUUUAUCAAGUUUUUCCCCGGGGGUGAGGAUUUCAAGUGGGGUUUGCUCCGGGAAGCUUCAGCUCUUAAAGACUUGUCUACCAAGGUUUUACACCUCUGACGACUUUCCUUUAAGGAAGAAGCUGCCGGGAAGGUAGGGGUGUUCGCCAUAACCGAUGCGGUAACGCAGUCGGUAAUGGCACCCUUAUCCUCCGC  >Ss-AA_clean.1_(paired)_contig_28  UCCUCGGUGAAAGUAACCCCCUUCCCACUCAAGUCUCUUAAUAGUGAUGGAAUACCAUUAAGCGACUGGAGGGCAAGGAGAGCGUUACCUGCACCCAAGGGUGUAAGUUCAACAUCGUCUCGAACCAAUCUCUUUGCAAACUCAAAGGAAUGCUCAGAGACUAAAGUCUUAGACAAGUUGAUCUUAACACCAAGGAUAUCGACCAUGAUACUAUGGUAGGACCUUGCGACAGAGUCGUUAGCAAUAACAACAUCAUCUCCCAGUACGGCAUAGUCCCCAAAGUCAGGUAAAGAAACCCGACGGGCGGCUAGUCGCACUACAAAAUGAUGGGUUAAAGCCAACAUACCCCAUGAGCUAAGUGCUCCCAUGGGCUGACCAACGCGAUAGCGGAACUGUUCUUCCUCAGCAGGAAGCCCAGGGACCGUAAUCACUUUGGCCAUCCAAUCUCGGUCAGUCAUUACAGACUUCCAGAGAUGGCCGAAUCGCUCCCCCAAGUAGAUAGACAGGAUUUGCACCUGAAGAUCUACGGGAAGGCGAUCCGUGGCAGCACUUAGAUCAUAGGAGUAAAACUUCUGACCCUCCAAUUUACCGUCUCUUUUUAACGCUCGCAACCGAUCCAACGGGGCACUUUGAUUAAAAGUACCGUCCUGAGGAACCUGUCGCAAGAGAUUAAAGAUCGCGGAAGACAAAGGGGCCAUGACUGACUGCGUUACCGCAUCAGUUAUGGCGAACACCCUCACCUUCCCAGCGGCCUCCUCCUUGAAGGAAAGACGACCAAGGUGCAAUACCUUAGUAGUCAGACCCUCAAGCGAGGAGGCUUCACGAAGAAGACCCAAUUUGAAAUCCUCACCCCCAGGAAAGAACUUGAUAAAAUCAAGCAAAUCCCCGAAGUUAGGACGAGAUGACCAGGCCUUGAUAUCUAACCAGAUACCCAGACCGGCCACCUUACAAUUAGGGCCCGCUGAACGGGAGAAAAGUCAACGGAUCGGUCCCAACUCUCCUAAAACAGGAAGAGUGGACUUAACCGCUAACAAUUCAUAAAUUGGGAGAGUAGAAGAUAACCCAGUGAAGGGACCCACUAUAGUGUCGAAGCUUACGCUACCGGGGAACUUAAUAAUUCGGAAAACCGAAAUACAAGUCAAGACUCCUCUGAUCACACCAGUAUCUCCGUCGUGCAUACGCAGACGAAGAACUCCGGGAAUGAUCGAGGGGAGCCCCCGGACAAUGGAAAUCGGCAUCUCGUCAGUGACGAGAACCGGGUUUCCCGACACGAAGUGCUGAACAAUUCGGUAGCACUCCUUCAAAUACAAAACAGUGAACUUACGUCCAUUGUGAGUAAUCAAGGAGCGCACCCGGACUGCUAACUUCAUGAAGUUACGCUUGUCUAACUGAAGUGCUCAUAUCAAAAGCCGGAAAUAUUGGAAGAACA  >Ss-AA_clean.1_(paired)_contig_119  GUCCUCGGUGAAAGUAACCCCUUUCCCACUCAAGUCUCUUAACAGUGAUGGAACACCAUUAAGCGACUGGAGAGCAAGGAGAGCGUUACCUGCACCCAAGGGUGUAAGUUCAACAUCGUCUCGGACCAAUCUCUUUGCAAACUCAAAAGAGUGCUCAGAGACUAAAGUCUUAGACAAGUUGAUCUUAACACCAAGGAUAUCGACCAUGAUACUAUGGUAGGACCUUGCGACAGAGUCGUUGGCAAUAACAACAUCAUCUCCCAGUACGGCAUAGUCCCCAAAGUCGGGUAAAGAAACCCGACGGGCGGCUAGUCGCACUACAAAAUGAUGGGUUAAAGCCAACAUACCCCAUGAGCUAAGUGCUCCCAUAGGUUGACCAACGCGAUAACGGAACUGUUCUUCCUCAGCAGGAAGCCCAGGGACCGUAAUCACUUUGGCCAUCCAAUCUCGAUCAGUCAUAACAGACUUCCAGAGAUAACCGAAUCGCUCUCCCAGGUAGAUAGACAGGAUUUGCACCUGAAGAUCUACGGGAAGACGAUCCGUGGCAGCACUUAGAUCAUAGGAGUAAAACUUCUGACCCUCCAAUUUACCGUCUCUUUUUAACGCUCGCAACCGAUCCAACGGGGCACUUUGAUUAAAAGUACCGUCCUGAGGAAUCUGUCGCAAGAGAUUAAAAAUCGCGGAAGAUAAGGGGGCCAUGACUGACUGCGUCACCGCAUCAGUUAUGGCAAACACCCUAACCUUCCCAGCGGCCUCCUCCUUAAAGGAAAGACGACCAAGGUGUAAUACCCUUGUAGUCAAACCUUGAAGCGAAGAGGCUUCACGAAGAAGACCCCACUUGAAAUCCUCACCCCCAGGAAAGAACUUGAUAAAAUCAAGUAAAUCCCCGAAGUUAGGACGAGAUGACCAGGCCUUGAUAUCUAACCAGAUACCCAGACCGGCCACCUUACAAUUAGGGCCCGCUGAACGGGAGAAAAGUCAACGGAUUGGCCCCAACUUUCCUAAACAAGGAAGAGAGGACUUAACCGCUAACNNNNNNAUCACACCAGUAUCUCCGUCAUGCAUACGCAGACGAAGAACUCCGGGAAUGAUCGAGGGGAGCCCCCGGACAAUGGAAAUCGGCAUCUCGUCAGUGACGAGAACCGAGUUUCCCGACACGAAGUGCUGAACAAUUCGGUAACACUCCUUCAAAUACAAAACAGUGAACUUACGUCCAUUGUGAGUAAUCAAGGAGCGCACCCGAACUGCUAACUUCAUGAAGUUACGCUUAUCUAACUGAAGUGCUCAUAUCAAACGCCGGAAAUAUUGAAAGAACAGAAAAAGAUUCAGUUCUAUAAUAGGACCGACUUUGCUAAGACGACCCGCCUCUUGUUUCCAAGAGGAAUGGUU  >FirsU_Contig22  UUUGAUCUGAGCACUCCAGUUGGAUAAGCGUAACUUCAUGAAGUUCGCGGUCCGGGUACGNUUCUCUUUUGACUCAUAAUGGACGUAAGUUCACUGUGUUGUACUUAAAAGAAUGUUACCGNGAUCGUUCAGCACUUCGUGUCGGGAAACCCGGUUCUCGUUACAAACGAGCUACCGCUUUCNCAUUGUCAGGGGACUCCCCUCGAUCAUUCCCGGUGUCCUCCGUUUGCGUAUGCAUGACGGNUGACCCCGGUGUGAUUAGAGGAGUCUUAACUUGUCUCUCGGUUUUCCGAGUGAUUAAGUUNCCCCGGUGACGUUAAAUUCGACACUAUUGUGGGUCCCUUCACUGGGUUAUCUUCUACCCUNUCCAAUUUACGAACUUUUGGCUGUUAAGUCGUCUCUCCCUCCUUUGGGGGAGCUCCGACCNUAUUCGCUGAUUGUUCUCCCGUUCUGCGGGACCUAACUNGUAAGCUGGCUGGUCUAGGUAUNCUGGUUAGAUAUCAAGGCCUGAUCAUCUCGUCCUAACUUCCGGGGAUUUACUUGAUUUUGUUAAGUUUUUCCCUGGUGGUGAGGAGUUUCGGUUGAGUCNUUUUAUCCGAGGUCCGAUCUUUACGGGACUUGACUACUAAGGUUUUACAUCUUGGUCGUCNUAUCCUUUAAAGAGGAGGCCGCUGGUAAAGUUAGAGUGUUUGCUAUUACCGAUGCGGUUANCGCAGUCGGUGAUGGCUCCUCUUUCAGCCGCUAUUUUCGAUUGUCUUCGUCGGGUUCCCCNAAGAUGGUACUUUUAAUCAAAGUGCCCCCUUGGACCGGUUGAGACAACUGAAACGCGAGGNGUAAAUUAGAAGGUCAGAAGUUUUACUCCUACGAUCUUAGUGCUGCCACGGACCGCUUACNCAGUAGAUCUUCAGGUGCAAAUCCUGUCCAUCUACUUUGGUGAACGAUUCGGUACUCUUUNGAAAGUCAAUUAUGACUGACAGAGAUUGGAUGGCCAAAGUAGUGACGGUUCCUGGUCUUCNCUGCUGAGGAAGAACAGUUUCGUUACUCCGUUGGUCAGCCCAUGGGAGCCUUAAGUUCGUNGGGGUAUGUUGGCUUUAACCCAUCACUUUGUAGUACGUCUAGCCGCCCGUCGGGUUUCUANUACCUGACUUUGGUGACUAUGCCGUGCUGGGAGAUGAUGUUGUUAUUGCUAACGAUCUGGNUCGCAAGAUCCUACCAUUCUAUAAUGGUUGAUAUCCUCGGGGUUAAGAUCAAUUUAUCUANAGACUUUAGUCUCGGAGCAUUCUUUUGAGUUUGCUAAGAGAUUAGUCCGUGAUGAUGUUGNAGCUUACACCUUUGGGUGCGAGUAAUGCCUUAGUCGCUUUGCAGUCCCUUAAUGGAGUUCNCAUCUCUAUUACGGGACCUUAGCGGAAAGGGUGUUACUUUCACUGAGGAAUCUGUGGAUANGUUUAAUUAGUAAAGUUCCAACUGUUCGUAAGAGCAUGUUGGACAAUAUUCUUUGAACUANUCAAAGGACCUUUCGGUUUCGUUCCGACUAGAGUAGGGCUAGCAUCGUCUUUGACGAUGUNCUAGCUCGCUGACUCCAGUUAGAGCGAAUCAGAUUAUUGACGCAGUUCGUCGGGUUAAACNAUCGUUUCGAUGUUCGUACCUGGCAAACUGCGGUCCGAAAGGCAAUAGAGUUCCAGAUUUNCGAUAUCUGAAN  >FirsU_Contig31  CGUAUCACGAAUACCACGGAGACCCCCAGGGGUAAAUCCCUCCCACUCAGCAAUGAGUGCNCUCGGGAUCCCUGAUCAUCCUCACUAUGUACCGUUCAUCUGAGUUAUCAGAGGCAGAGCGNGACAAGCCUAGGGCUUGGUAGCUACGGCUACUGACAACCCGAUAGCCCCACGGUAUGGUUNUAGCUGAAUAGCCAAUCAGCGCGAUUACCUGCCGGGGAGAAACCUAUUCAGCUCAUAAUANCUGAACCGAAAGGUCACUUUAUGAGCGGUGUAGAUUAAGAGGAAGUUCAAUUGAUUAGUCNUUAUGACUCAAUCACUAGUUUUAAAACUAAUGAAAUGAGUCCGUAAGUACUACUACAAAGNGAACCUCUCCUAAUCUAGCCGCGUUUACCCGUCAAGCAGUGCACUUGUUCUUCCUUUGAGNGGAAGACCAAGGGGUGAAAGUGGACCGCUACAGCUUNUUAAACUGACACGGUUAGCUAUCACUCGUACGCUUGCUGGCCAGGAGCUUCCACGACCCUNUUGGGAUCUCUCUUUGUAAGAAGACGAAUCUACCUAUGAUGUUACCAUUACAGGUGAGACNUCGGAAUCUUGCGAAGAGAUUUCCUCCUAAUAUCAUAUGUCUUGACAGUUUUGCAACUGUNCGAGAAUGAUCUUAGGUACAGGGGUCAUCGCUUCAUACGAUAGUAUCACUCGCCCGUGUUNCAGGUAUAAACCCGUUCACGUCGUCGAGCGUUGCUAUUGUUAUGAGGCGAAUGGGGAUAANGUCGUUUGGAUGAACCCACAUCCUUCACUUUUCCUUGGGUGUCCACUAGUGGGCCAAAUGNGGAUCUCCAUAGCUACUUCCUUUUCUGACUUGAUGAAUAUCCCUCAAGGCAUCCUGGAGUNGUCUUUACACUCUAGGAGGUCCUGAGUUCAAGGAGAAGGUGUCCAGUCUUAAAGGAUUCGNUAGAGACCACUAAUGGUCUUCUAUGAAAACUUACAAAACUGGACCCAGAUAAAACUGGUANACCUGCUCCGACGGAUUUCCAUCAAGAAGGACAAGGAGUACAAGUCGAGACCCUUUGCAANUAGUCGAUUAUAUAACCCAGAGUGCAUUGACGCCUUUACACGAUAGAUUAUAUCGCGUAUNUGGAAUCGAUACCUCAGGAUUGUACUUUCGACCAAAACAAAGGGUUUAAAGACUUGCUGUNACGGGGGUGGACCAUACUAUUCAUUUGAUUUGACCUCAGCUACUGACAGAUUUCCUAUAUNUCGUACAGGAAAUGGUCUUAGCCUGGUUAACCAGUGAACAGUAUGCUUCCGCCUGAGUACNGGGCCAUGGUAGAACUUCCAUUUAGCACACCGGGUGGUCCCGAGGUUAAGUUCGGGUGUGNGUCAGCCACUAGGUGCAAAAUCAUCAUGAACUAUGUUCACCAUUGCCCAUCAUUUUGUGGNUCCAGUACUGUGCAAUGGAGUUAUCCAUUGACAAUCCUAGAUACAAAAUUCUGGGUGAUGNACAUAGN  >FirsU_Contig24  NACUACCGGACAAANAUCCGUUUGGUGGUGCUAGUGUUGCUUUAGUAAUGGCUCGUAUGGGGAUUCAUCGUUUAGNAUGAGCCCACUACUUUUACCUUCCCGUGAUGUUCAACUAGUGGACCGAAUGGGGUGUCCANUAGCAACGUCUUUCUCCGACCUGAUGCAUAUCCCUCAGGAUAUCCUAGAGUGUUUGUACANCUCUAGGAGGACCUGAAUUCGAAGCUAAGGUGUCUAGCUNUGAAAGGUUUCGUAGAAACCANCUAAUGGUAUUCUAUGGAAGCUAACAAAGUUAGACCCAGNAUAAAACUGGUUCCUUACUCCNGUAGGAUAUCUAUCAAGAAGGACAAAGAGUAUAAGUCCCGGACCUUUUGCCAUAGUCGAUUAUAUAACCCAGUCGGCAUUGACUCCUUUACACGAUAGAUUAAAAUCGUGUCUUGAAGUCCAUCCCUCAGGAUUGUACUUUCGACCAAAACAAGGGGUUCAAGGGACUUAUUGUACGGAGGCGGCCCAUACUACUCGUUUGAUUUAACCUCGGCUACGGACCGGGUUCCCUGUAUUUGUACAGAGAAUGGUUCUUAGCUGGUUAACCAGCGAGAAAUAUGCGGACGCCCUGAGUACAGGCCAUGGUUUCACUCCCGUUCCGAACACCAGAUGGACAGGACGUGAAGUUUUGAGUGUGGGCAGCCACUAGGUGCAAAAUCAUCAUGAGCUAUGUUCACCAUUGCCCAUCAAUUUUGUGGUCCAGUACUGUGCAAUGGAGCUAUCCAUUGACAAUCCUAGAUACAAAAUUCCUGGGUGAUGACAUAGUUAUAUGUGAUCAUGCACUAGCGGCUAAGUACUUAGAGGUGAUGUUCCCAACUGGGUGUAGACAUAUCUCCGGUUAAAACUCACGUAAGUGAAAACUUAUUUGAGUUUCGCCAAAAGAUUUGGUCUACAGUCCGUUGAGAUAUCAAGUUUCCCUAUCACUGCGCUGAAUUGCAGACAUAAACAACUAUGUUUCAUUAGUUGCUACN  >Ss-AA_clean.1_(paired)_contig_1865  UCUCCUCGUGAGAGGUGAGAACCACUAUAUCCCCACUAAUGGACAAUUAAUUGUCCACUAGAUUUAUAUAGUGUCUUGCGGAUAUCAAGCCGCAAGUAGUACCACUACAUCUCCAGAAGUUGAGGGUAGCAGACCAAAAUCUGCAACCUUUCCUCUAGGAGAUUAGCGAAGCGAUCGAUGGAUCUCAUUAAAGAGAAAUUUCGUCCCUCCACAAUACUUUUAGUACUUGUGGGGAUCGAAAAGUUUCUCAUGUAAUAAGAUCAAUCAGAGCCGGUACUAUCGAUCUUCUCAAUCCUCUUCAUUAAUUCAUCAUACUCUUUAUAUAUAGCCAAAGCGGCUAAUGAUAUAGGAGUAUCCUGGAAAAAUGAUUUGGCCUUGAGAAUUUCGAAACUGGAACAUCCUUGGAAUUUCUCAUCUAUUCAUCGAUCUCAGUCGCGCCUCAAUAAAUUCACUGUUUCCUGUAAAGGGAAAUUGUGUCUAUUGGGGUCGGCAAAGAAGGUAUGGAUAGCUGAGUUACUGAAGACCUCAACAGCAAUAUUUGAUAUGAUAUUUUUACAUAUCUCCAUAUUAAUGUGAGGUAAUGGUAGUUGAAGGGUGUGAGCCAAUCCGUUUAGGAAAGUCUCACCAUCCUGAAGACCACGUACUAGUGAUAGUACUCCUUCAGCAGUUUUUACUCUCCUAGACAUCUUCUUUCGAAGACGGCUAGGUAGUGAUAAAACAAUUCCGUAGAAUGUUUCAGCACUUGAUGAAAUGUCACCGAACUCCCAACCUCGAUCUCGCUGACCCAUUAGCAGAUCAGUUAGUCCAGAGAUGGACUUCCUGUUCUCCUUAAGGGAGCUAACCGGGAAGGGCGUAAUCUCCUCUCCAUCGAGGAAUACUCUCUUGGCGAACUCAAAGAACCUAUCGGAUCUAUGAGUCUUGGCAAGUGAGUAAUCAACAUGUAGAGAAGAUAUUAGUUUCAUGUACAUCUCACCCACGAGCUUAUUACCAAUUACUAUAUCAUCACCAAGUAGGGAAUAGGGUAAGGUUUUCCACCUCACACCCAGUUCUUUACAACAGUGAUAUAUUAGGAAAUGGUGUGUUAAUGCAAAUGAAUUGAAGGAUGAGUAGGCACCCAUAGGAUUCCCGGCAUUGUACUUGUACAAUGAUCCGCGGUAUUCGAACGGUUGCCCUACCAUCACUUCCUUUCAUGCAUCAACAUACUCAUGGGGCAAUUGGAACUUUAACAAUUGCUCUAUUACCUGAAUAGGGAAUCUAUCAGUAGCUGCCGUUAAAUCGACACUAUAGAAGAUAUCUAUAUUCUUGUUUAGCAACAAUUGUUUAAAUCUUCCUUGCUCAAGGGUACAAUCCUGCUUUAUCUUUGAUAAACAAACGGAGAGCCAAUUAUGUAAUGGUUUUAGUGCAAGUUGACUGUAAUGGUCAACUACACCUACCACUCGCAUCUUUGCCUCUUUGUCUGGGAAUACUGAAAGCCGACGACAACAUUUCUUGUUGUUGUCAAACUUAUGGUAUAUCCUGUCAAAGAACUCACUAAAAACAGGAUUCUUAAAGUUACGAAUUCUAGCACCAAUUAGUGGGCUAAUCUUAGUAAGACUAGCUGCUAAUGUUGCCGGAAUGUAUUGAAUGUCUUGGAGAGCAGUGUUAAGUGCACGGCCAUUUGGGCCGUUACUUAAUCUGUAAACCUUCUCAUUCACUUUAAGUUUUCUGGGUAGUGAGAAAGCGGAUAUAGGCUUAUAUCCCAGUGACCGUCAAAAGCUUGCCAUAUGCUUAGUAAGAUCAGGUACAUCCCCUUUUAAGGGUUGUGUGAUCGUAGUAAGAUCUGGCUCACUUUGGAUUUUUAGUGAUCUUGUAGCUGUUAGUAUCGUUAGGAUAAGAGCGAUUGCUCGCUUAUGUCCUCCACGAACUAAAGGUAUCAAGUCACCUAAGAUCACUGGAAUACCAUCGCUUGUUGAUCUAGCCAACGAGUUCCGGAGAACGUUACCUGAUAAGUAAUUAUAAAAAUUACCUCUAAUAGCUUUUAUAUACUUAAUUGUAUACAAAGGGCCAUUAUUGUUCAGGACGGUCUGGAGCUUGUUGCAUAGAGCUUCCAAGUGAUGUAGUGGUAAUGAAGUAUUACAAGAAGUAAUACACAAUCACUUGAUUACCUUGAAAGUAAAUCCAAACAACUCCUUACGGAGCUGUGAGGAAAAACUAUUUUUAUUAAUAGUUGUUUUUGUUUUCAUAGUAAUCUUGUGAUAAAGUCGCCUAUUCCCCGACUAGCACUGUCGAGUCCAAGGGCCAGCACUAAUCCGAAAGAUCAGUGUCCCUUGGAGGUACUUGCUUGAUUGCGUACCGUGUAUUCCUACACGGCAGAAUUCCCAAUCUGUCCGUUCUGGACAAGACUGGUCUACAAUCAAGUGGAGGGAGGUGCCACACCCUAGGCCCGGGGAUAUACGGGACUUCCAAUUAGGAAGUGUUUUCCUCUUUCGAGAUC  >Ss-AA_clean.1_(paired)_contig_90  CCCGAUCUCGACUUCACGUCGGAUAAACACUGUUGAACCCCGGGAGCGGUAACCUCAGGUAACACUGAGCUUCUACCACUACCUCAAUGUAAGCAGUGCACCUAGUGGCCUCCGAUACUCUACCGCCAAGACCUUAGGUACCAUAGUGACCACGGCGUGGCGGCUCUGGGUUACUACAAAACCCCAGCGUAAGGAUCGCAGGACUAAAUACAUCCUGGAUGCUUAAGCGGGGAGUAGCCUACCCAAAACGCGACAGUAGUGGAAAUCAGCUAUGACCGCGACACAUUUCACCAAGGGAGGUAAUUCCUUGGGAUGGAUGUGGCCGGCUUGUCCCUUUGGACCCUGACAGUAGGACGAUCGGAGAUCGCUACUAGGCGGCAUCCCACCACCGCGAGAUGGUAGGUUGUAGGGCGCGUCGACCCCGUAAAACAGGACGAUUAUUAUGAACAAAAUAAACUUCAAGUUAUUAAAAACAGAAGUCGCUUUUGUUCACAGAUGAUACUUUCCAGGACUAGACUUUAUCACCCAGGCCGACGUGGACUACUGUUUGGAUAGGUUCGAAAGAAUCUAUCAAACCAGGGGUCCACUAGAAGCGGCCCGGGAAAUGAAAGCUAGCCGGUUAUUGCUAACCAAGUGGAAAGCAGGACAACCAAUGACCGGGUCCGUAGGAGCCCCAAUGUCUAAAGUGGACUUCCUACCUAAAGCGUACCUUACGCUAAAAGUAAGACGUCGACUGAAAGACGACCCCGACCACGAACUAUUUAGGUUCUGUUUAACAUUGUUAAGCAUAUCCAAAAUAAUAAUGGGAGGGGCACCUCUAGACUUGUCAAGUAUUACUAACCCAUCAACGUCUAAAACGACGCUAACGGAUAUAGAAAUAUCGACAGGUCUGAAGGCGCUGGGACUACGUCCGGGAAUGCUGAGACCUAAGUGAAAGAGAUUUCACUGAGCGUCUACAGCCGGGCCAAAUGGUCUAUCCAUCGCACAAGCUAUACGAGAUCUCGGCUUGCUACCCGACGGUCUGUUGACCAGUAUGGUGCAUAUAGCCGGGGAUUCUUUCGGUGAAGCCGUGAAGGGUUGUAAGGGGUUAAUUAGCGCUUACCCGACUUUAUGAAAACUUUUCAGUAAAGUGGAGCCGCGUACUAAAUAUCUCCGUAAACUCUCCAUUAAGGACGACACCGAGGGAAAAUCCCGACCUUUCGCGAUCCUGGAUUACUGGUCGCAAUCAGCUCUGACUCCUCUUCACGACGAACUGUAUAGAAUUUUGGGAUCGCUCCCUCAAGACUGUACAUUCGAUCAACAAAAAGGGGUUAGGUUGAUGCAACAGUACCCCAGGUCGCGUAUGCACUCAUUGGAUCUAAAGUCUGCCACCGAUCGCUUCCCGCUAGAAUUGCAGGAGCGUAUCUUGGCCUGACUUACAGACGCCGGUUAUGCCUCCGCUUGGAAGGACGUAGUAAAUGGUUACGACUUCGAGUUCCAAGGGCGGUCUCUCCGAUGAACGGUGGGACAACCCCUGGGAGCAAAAAGCUCGUGAGCCAUGUUUACACUAUCACACCAUGUGGUCGUUCAGGUCGCAUUAAGUCGGGCGAAAGCCCGAAGCGAUUCCUACGUACUACUAGGUGACGAUAUUGUUCUACGAACAACCAAGUUGGCAAAUGCUUACUUAGAGUUAAUGGAGGGACUCGGAGUAGAAAUAAGUCCCAGCAAGUCGCACUCCUCUAAAGGAAUGUUCGAAUUUGCCAAGACUUGAAUCCACUCCGGAUCCCACAUCAGUGGUUUCCCAAUCAAAGGUCUUCACGAAACAUUAUUCAAAAUAUCAGAAUUGAUACCAGUAAUAACGGACGUGGGACCCAUGAGAGGUUAUCCACUCCCAUUCUCAACAGGUAACCUCGCCGAGUUCUCGGCCGAGCUAUCUAAUAUGAUGAGUAAGUUUUCUCGUCAGAGAAUAAAUCUCGGACGAAAAUUUCAUCUGAUGACAUCUUUCGUUCUUGCCUGCUGUGAUAAUGAGUGAAUGAAAUCAUUCGUUCAUCAGAGCACAGGAUACCAGACGGAGCACUACCAAGAGCUGUUCAAAAGAGCAGUGAUGGUAAUAGUCCGUGAUGGUAAAGAGCGAGAGCUACAUCAGCUUACUCUCUUCGCUCUCGGUGUGACGAAGUCAAUUCACUCCGGUUGGAGUCAAUUGUCAUACGACGCACUUAUAGCGGAGAGUCAGCGGAUAAUCGCCGAAGGAAGAUCCUUCAAGGAGAUUGCCGCGAUACAUAAAAGGAUAGCUACGUCCAAAACGAUGGUUAGUACCACCGCCCCGGGUACCCUUUCCGUGGUGGAUCAGCUGUCGUCGACCCUUCCUGUUCUGGGAGCUCUUGUUACGGAGCGAUCAGACAGGGAGAGCUGACUGAAUAACCUUGACGUGUCCAAUCCCGAUCAGUUUUGGGAAGGAUUUAAGUCGUGAGACUUGAAACCCUUUCCUCAACUAAAAGGUUUGAGACCCGAAAGAGUUAAGACAGUGACCCAAGCAAGAACCAGCUUAGCUAGUAAAUUAGAUAAAUUUCUAAAACUAGUAAGUUAGUCUGUGACUCUUAAGCGACAGGGGUGUAAUACCCUAUCCCCUCGAAGUCCCUUGAAAAGGGCGGGUGAGGUGAGAUUAUCCAGCGUAAAGCUGGA  >FirsU_Contig43  UAUAGUGGGUCCCUUCACUGGGUUAUCUUCUACCCUUCCGAUCUAUGAACUUCUAGCGGGUUAAGUCGUCUCUCCCUCCUUUGGGGGAGCUGCGACNCGAUCCGUUGGUUGUUCUCCCGUUCNGGCGGGACCUAAUUGUAAGGUGGCCGGUCUUGGUANUUUGAUUAGAUAUCAAGGCCUGGGCNAUCUCGUCCUAACUUCGGGUCAUUGCUUGAUUUUANUCAAGCUUUUCCCCGACGGCGAGGAUCUUCCGAUUAGGGCUUCUCUCCGAAGUCUCUGCCCNUUAGAGACCUCCCUUCCAAGGUUCUNUCACCUUGGUCGGUUGUCCUUUAAGGAAGAGGCUGNCGGGGAAGGUAAGGAUCUUCGCCAUNCACUGACGCGGUAACGCAGUCUGUGAUGGCCCCCUNUAUCCGCCGCAAUCUUCGACUGUCUNUCGACGGGUUCCCCAGGAUGGUACUUUUAAUCAAANGUGCCCCCCUGGACCGUCUUCGAGAAUUGAAGAGGGAUGGUAAAUUGGAAGGUCAGAAGU  >Ss-AA_clean.1_(paired)_contig_3545  ACCAUGCACUCGCCCUCGAUGCUAGACUUGGAAAUUACGAGAUGCCUGUUCACAUCUCUGACUGCCUCAGCUCUGCUGGUGUCCCUACACCCGCGUACCCGACAAUGAAGCAUGCUCACCCUGGCCACAAGCGACUUGAAAACUACCUCUACCAAGUCGCCUGGCCAGCCCUCAUCGGCCAAGACUCUGUAACCGUCGCCUUCUGCAAGCAAAAGAAGUUUGAUGUUAUUCAGCAGUACAACCCGAAGUUUAAGGAGCUUGUCAACUGUGACCUCACUCACAAGGACCUGAGUCGUUAUGGCCCAACGGUUCUCCCUGAGGUUCUAAACAAUGACACCCUUCUGCUUUACGACGCUGGUCAUUUCUUAACUGACUCCGCCGUGGCUGCCUUGUUCGUCCGGUACCCGAACCUGCAGUACAUCUACGCAGGUUCGAUUAUCCCAGCUGAGACCAAAGAACACUGCGCGUCGGCCUACCCUGGCUUUUACACAUUGGAAUACUGCGGAAAUGAAGUUGUUUACCAUCUUGAGGAUGACCCCGCCGGCUCCUACCGUCAACCUUUGGACGCCCACAAACUGCUGAACCCUUCCCGUGUUUCAGUCGUUUCGCCCACUCAAACGGUUUGCUGGCAGAGUUCUGUGCUUGGGAGCUUCUUGGCACAUCACCUCCUGCUGUUCAGCAGGAAAUUCUCCCAAGUGCCUCCGGAGACCUUUACGUACAAAGUUCCCAGAUGCAUACGCCUCCCUCAACCUUUCCUUCGCCGUCGGUUUGAUGAGAGACUUGGCCACACUGUUGUUUUCACUGAGGCAUACUCCAUGGGUCGAAUCUACAAGGCAAACCUCAAGAAGAACAAUCCUGAAGACACUGUGUCGAAGGUUAGGAGCAUGAUGAGUCAGCCAAAGUACGCCGGUUUACCUGAAACCGCCUGGGAAGAGAUGAGACUUGUUUGGGACGCUGUGAAUCAAAUCGGACUGUUUGAUCCAAGGUACCUCAAUCUCACAUCCGGUUUUAUGAGUUCCGUUAAGGUGGCCAUCAUGGACUACUUCACUCCUGAUCGCUCAGCCCGCACUGACUUGAUGGUCGACCUCCUGCUUCGCGGUAUGCACUACCUGUUGCCGAACUGGUUGUCCUUUCCAAUGCUCAUGGGUGAACUUGUCUAUGCGCUUCAGAACCACCAAGCGGCGCAUGCUUCUGCCACCAUCGUCAUUUUGAUCGGAGGUCUUGCCAGCCCGGUUUUCGCUGCAUGUUGGACUGUCUUCGGAAUCGGUCGGCUUGCCUACUCAGCGUACUACUCAUCAAAGGUCUCAAAGGUUGACUACCUUCUUGACUCGGCGACGGCUCGCCCUUGGGUCAUCACGUUUCCUCCUCGGACGGCAAAGUACCAUCAGCGCAACUUUCACCUCAACCAGCCGUUCCUUCAACCCAAACCCUCGUUCUUGAGCGUACCACUGUUCUCUGACAAGCUGUGUUCCUUUUGUCCCAACAAGGUUCACAGUGUCACCUUGGUUUGUCUAGACUGUGGUACUUGCCCGGAGCACAAGGUUUACGACGACUCCCUGGUGCGACCACGACCCA  >Ss-AA_clean.1_(paired)_contig_13690  CAUUGGAAAGGACAACCAGUGUGGUAGUAAGUAAUGCAUGCCACGGAGCAGAAGAUCAACCAUUAAGUCAGUGCGGGCGGAACGGUCAGGCGUGAAGUAGUCCAUUAUAGCGACCUUGACAGAGCUCAUGAAACCAGAGGUGAGAUUGAGGUACCUGGGGUCAAAGAGUCCUAUUUGGUUCACGGCAUCCCAAACGAGCCGCAUCUCCUCCCAAGCAGUUUCGGGGAGGCCGGCAUAUUUGGGCUGGCUCAUCAUGCUUCUCACCUUAGACACAGUAUCCUCAGGGUUGUUCUUUUUGAGGUUGGCCUUGUAGAUACGACCCAUUGAGUAUGCUUCGGUGAAGACUACAGUGUGACCAAGCCUUGCGUCAAACCGACGACGGAGAAAGGGCUGAGGAAGACGUAUGCAUCUGGGAACCUUGUAAGUGAAAGUCUCAGGAGGAACUUGAGAGAACUUUCGACUGAACAAAAGAAGGUGAUGUGCCAUGUAGCUUCCGAGGACCGAGCUUUGCCAACAAACUGUCUGGGUGGGCGAAACGACUGAAACUCGGGAGGGGUUCAGAAGUUUGUGAGCGUCCAAGGGUUGUCGGUAGGAGCCCGCUGGGUCAUCCUCAAGGUGGUACACGAUCUCAGUGCCACAGUACUCCAAGGUGUAGAAGCCUGGGUAUGCCGAAGCACAAUGUUCCUUGGUCUCAGCCGGGAUGAUUGAACCUGCGUAGAUGUACUGCAGUUUUGGGUACCGAACGAACAGGGCGGCAAUAGAAGAGUCAGUUAGAAAGUGGCCAGCAUCAUAAAGCAGAAGGGUGUCAUUGUUUAGCACCUCAGGCAGGACAGUUGGGCCAUAACGGCUCAAAUCCUUGUGGGUGAGGUCGCAAUUGACAAGUUCCUUAAACUUGGGGUUGUACUGCUGAAUAACAUCGAACUUCUUUUGCUUGCAGAACGCUACGGUGACAGAAUCCUGACCAAUGAGUGCUGGCCAAGCGACCUGGUAGAGAUAAUUCUCCAAGCGUUUGUGACCAGGGUGAGCAUGCUUC  >Ss-AA_clean.1_(paired)_contig_10849  CGGCAAGAUUGUACCGGCAGUUAUCGUCGUAGGUCCCAGGUUCACCAGUGUCGCGACCAAGGUCCUUUUCACCAAUAAUGUCGGAGUAGACACCCACCUUCCAGGAAACAUAAAACUCAGAGAGAUGUUUUGGGAUGGAGAAGCGAGCUUGAUGCCGUUCAGCAAAAGCGACACUUUCACCUUUCUGAGAGGAGUCAAAAGCGGUGUAGUCGUUUGUAGUGCUCCUGAUGUCUUUCCAGUGCUCAAUGGUCCAGUCCCUGAGAUCUUCAUUACUUGUGCCGCAGUGCAAAAAGAUGUUCUUGGGUCUAUCUCUAUGCUCAACUCGACGUAUGUAGCGAUUCAUAGCACCAAGAAGAAUUAACACAAGGUCAUGGCAUGUUGCAAUUGUCUGACCAGGUUUGGCGGGCAUCAAGAAAGCCUCCAUUUUCCCUUUGAGUUGGCUCUUGAGAAAGUGACUUACAAGGUUGACUGGACGGUCAGGAUCUGAGCGGUACUCGUUGUUGGCAAGCAUCUGUUCAGUCUUCUUUGUCAGCUUGACAGUCUCAUUCUCCGAUAUGCACUCCAGAAAGAGCUCCUCGUCAAAAGGGAUGGUAGCGUCAGGGUUCCAACCAUGAAGUUCGCAGUACUUGGUGUACAGAAGCUCACCGAUCCAGGCCUCAUCGUGGUAGUACUUCCAGUUACGGUCAGUGGAACCUUUAACAAGGCGUUUCUUAAUUGUUGCAAGCAUCAAGGCGUCAUCCUUCGGAGUCUGGACGGGGAACCAGGAGCCGGGUCGACCGAUAACUUCCUCCCUGUAAAAGCAGGCUGUGGGACCACGAUCAGUCAUCAAUUCUCUGGCCUCCCUGAGAAGGUUUGCUCCCUCUUCCAACCUGCACAGAACAUACUCGUCAUUGGCCUCGGGAAAAUGGGUUGAGGCGCGGGGAUGAGGAAUGAAAACUGACGCUGGUUUAGGGUCGGAAACUGG  >Ss-AA_clean.1_(paired)_contig_11935  CUGCGACGAGUCGAAAGCAGUGUAGUCGUUGGUCGUGCUUCUGAUGUCCUUCCAAUGCUCUAUGGUCCAGUCGCGAAGGUCUUCGUUACUUGUGCCGCAAUGGAGAAAGAUGUUCUUAGGUCUGUCUCUGUGCUCCAUACGACGUAUGUAGCGAUUCAUGGCUCCGAGAAGAAUUAACACAAGGUCGUGGCAGGUGGCGAUUGUUUGACCGGGUUUGGCCGGCAUCAGAAAGGCCUCCAUCUUUCCUUUAAGUUGACUCUUGAGGAAGUGGCUUACGAGGUUGACGGGACGGUCAGGAUCCGAACGGUAUUCGUUGUUGGCAAGCAUUUGCUCGGUCUUCUUGGUUAGCUUGACGGUCUCAUUCUCUGAUAUGCACUCCAGGAAGAGCUCCUCGUCGAAGGGUAUCGUGGCGUCGGGGUUCCAACCAUGUAGUUCACAGUACUUGGUGUAGAGAAGCUCACCGAUCCAGGCUUCGUCAUGGUAGUACUUCCAGUUGCGAUCCGUUGAACCUUUGACGAGACGUUUCUUGAUCGUUGCAAGCAUCAGAGCGUCAUCCUUCGGCGUCUGAACGGGAAACCACGAGCCGGGGCGACCGAUGACCUCCUCUCUGUAGAAGCAGGCGGUUGGGCCGCGAUCAGUCAUCAGCUCUCUGGCCUCACGAAGAAGGUUGGCGCCCUCUUCGAGUCUGCACAGCACAUACUCAUCAUUGGCUUCGGGAAAGUGGGUUGAGGCUCGAGGGUGAGGGAUGAACACUGAUGCGGGCUUGGGGUCAGACACCGGGUUGAGACGAAUAGACUGAAGCAAGCUCUUUGUCAUCGUGGGUGCGCGCUCCCAGUUGUCAGUGUAUUGCCCAGAGGCUCUUGGGAGGACCAGACGUUUGGCUCGAAGCAGGACUGCGUCCUCUUGACUGAAGCCGACUAAGGUCAGGCCGGACAGCUCAGACCGAAAGAGGUGCGUGUAAGCCAUGUCUGCAGGACCAAAAGACAACGGGUCGUUGUUGAACACUGAAGAGAACUUGAGAAGUUUGCCCACGAUGUGGUGGGAGUUCGGGUCAGAUCCGUGACGCAUGGAGUCAGCAACAAGAAUCAAGGUGUCAGAGCAACGGAGGAUACCGGAGACGAGGGUGGUGGUUUCCAUCAAAGACUGCACAACGUUAGUGUAACGGAUCUGUACUCUCGGGAAAGACCUGGACUGGGCCUGCUGGAAGGUGAAGGCUUUAAAGCCAGCUUGCAGGAGACCAGCGCACUCAACAUCACUGGCGACAAGAACGGGGUCUUUAUGGUUCAGCAACGUUCGGAAGAGGACCGCACCCUUGGCGGGAUUGGAAGUAGGAACAUUGAAGAAGUUUGCCAGAAGUUGAGGAGCACGAUGGAACUGUAAGAGGUACUGCGAGCCGCACUUUUGAGCCCAGUAGACCGCCUCGUUGAGGGUGCUGGAGAGACCACUGUCGGGAUGAGUCGAGUGAAACCUGCAUUGGCAAUGAUCACCAAGAAUUAUAACUCUCUUAAUGCUAGGGUAAAGAACGCAGAGGAGAUCUACGUAACCCGGGGGAAAGAGAGAGAUUUCAUCGACCACGAUGACAGGCCGCGACCUGACCAUGGCUGUUUCCCAGGUUGACACCAUCAUCUUCGAGUCGUAGGCAUUUAUCUUGCUGACCCAGUCUUCGCGCAUUUUGACUCUCGGAACAAUGACACCAACCUCGAAGUCUUUGCAGAAGGCUGGGUUGAGGUUGAGAAAGUCCUGAAGAGGAGCGGACUUGCCACAGCCUGGUGCUCCAAGAACAGUGGCCACCUGGACCGAACGUGAGCUGUCGUAACUGUCAACAAGGGAGUCCAGGCGGGCAGAGAAAGCAUUGACAAAGAUCUUACCCUCGUUGGCUUUGAUCGUACCCGUGUAACCUUUCUUGAUGUCUGAGAUCAAGCCAGAAGCCCUAGACUUGUUGAUGCGUGCGCGUUUGAAAUUCAAGUGUAGGGGGGUCCUACUUGAGGCGUUGAAAGACGCGAGCAAUCGCAUGAAGCUGUCGGGUUGGUUGGGUGUGGUCUUGGCAAGAUUGGCCAGGGGACGAGGAGGUCGAGCUACGGGCUCCCAGUGCGGAAUGCCGGCGACAACAGUGAAGUUAAAACCAAGGCAGUUUGUGUCUUUGACGCCAACGCGGCUGGGAACACCUUGAGGGACGUUGAGCAGUUUGGCGCAACAGCGGAACCUGAUGCAGGCAACUGCAAAGAAGCGUUCGUCAAAACCUGGAGACGGGAGCAGAUCCACUGACGCAGACGGGCCGAGGUAGUCCUUCACAGAGCUCCACACUACAUCCUGACUGACACCGACCGCCGACGCGAAAGCAUUUAAGGCGCAAGUAUUCGGUGUGACAGCGUCCACAAGGCCAGGCGUGAUUCUCAGGUUAGGGUGCAAGGCGUUGUAGAUGUCGGUGUGGGGUUGACCAGAAAUGGUGUAUAUUGCGACUUCCUGGGGCAGCACGUCAACACAGACAUGACACUGGGCGUCAUCAGGAGCCGAAGUCCAGAGGCCAUAACCAGACUUGCAACGCGCCCACCGUUCAUUGCUUGCCCCGAAAUCACCGGAGUGUUGAGGACAACGGCAGUCCAGGUGAGCGUUGGUGCUGGUCUCUAUGGGGCCAAAGUCAAGGACGGAUGCCGAGUCGCCAGGCAGAAUUUCAUCAUCGGAACCGCUGAUAUCGGCGGGCGGCGCGGUUGGUGCAGAUCCAGGUAGUCGAACGACAGUGGGUCCGGAGAAAUCGACUUCCGUGGGAGAGGCGGACGGUGCAGCGUGACCCUGUGGGGGGUCGGGCUCUGGCAGUCGUGCGACAGGUGGUCCGCCAAAGUCAAUCUCAGUCGGGGAGCCGGAAGGGAAAGCGUGACCGUAUGCUGGUGUUGUCAGGCCGCAAGGGGAAGUGAAGACAGGAAUGUUA  >Ss-AA_clean.1_(paired)_contig_14854  CCACCAAGGUAAGACCAGACAGCUCAGAUCGGAAGAGGUGUGUGUAAGCCAUGUCUGCGGGACCAAAGGAAGAGGGGUCAUUGGUGAAAACAGAGGAGAACUUGAGAAGCUUGCCAACAAUGUGAUGAGAGUUUGGGUCAGAACCGUGGCGCAUGGAGUCAGCGACCAAGAUCAAGGUGUCAGAGCAGCGAAGAAUGCCGGAAACAAGGGUGGUGGUUUCCAUCAGAGACUGCACAACGUUUGUGUAGCGAAUUUGGACUCUGGGAAAUGAUCUCGACUGUGCCUGUUGAAAUGUGAAGGCCUUGAAUCCAGCCUGCAGCAGGCCGGCACACUCAACGUCACUGGCGACAAGAACGGGGUCCUUGUGGUUCAACAGUGUCCUGAAAACAACGGAACCUUUUGCGGGGUUUGAAGUUGGGACGUUGAAGAAAUUGGCCAGUAGUUGUGGCGCACGAUGAAAUUGCAGGAGGUACUGCGAACCGCAUUUUUGAGCCCAGUAGACAGCUUCGUUGAGCGUGUUGGAGAGACCACUGUCGGGACGGGUCGGGUGAAAUCUGCACUGACAAUGGUCACCAAGGAUGAUCACCCUUUUAAUUCCUGGGUAGAGAACGCAGAGAAGGUCAACGUAACCGGGGGGAAAAAGUGAGAUCUCAUCUAUGACUAUGACAUGUCGUGACCUAACCAUUGCGGUUUCCCAAGUGGACACCAUCAUCUUGGAGUCGUAGGCGUUGAUCUUACCAACCCAAUCCUCACGCAUUUUCACUCUCGGAACGAUGACGCCAACCUGAAAAUCCUU  >Ss-AA_clean.1_(paired)_contig_18967  CCUGCCAGUACAUCGUGCCCCGUGGUCUCCCGAUCUUGAUUGGUGGCGAUGACUCUGCCCUUAACGCUAGGCUGAGGAAGUCCCCAUUUUGGCUGUUCUUUGAGAAGCAUCUCCGCAUUGUUAGCAAAACAGAGGAAACCGAUCGGCCACAAUUCUGUUCUUGGUACGUAACUUCCGCUGGAGUCUUCAAGGAUCCCAAGCUCAUGAUGUUGAAACUCAUGUACCAUGCCGCC  >Ss-AA_clean.1_(paired)_contig_4648  CGACAUCGGACUCGAGCAACGACACAGCGUAGGACAGAAGACCAGUCAGACCUUCGGUGAAAACCAAGUCGGAGAAAAGGAGCUUGGUGACAGAACCGUCAAGAUGCCGAUGAAAGAAGCGAAGAAGGUAGCCAAGGCAAAGGAGGUCAUCAAAGUCGCAGUAGUCGACCAGAGUGUCGCCAAGCCGAUAGCCCUCUGAGACCUCAUGAGCAUAAGAUUCCGUGACUUUGUCGAGAUCACCAAAGGCAGCAUGGUACAUGAGCUUCAACAUCAUCAACUUUGGGUCCUUGAAGACGCCAGCGGAGGUGACAUACCACGAGCAAAACUGCGGACGAUCAGUCUCUUCGGUCUUGCUGACAAUGCGGAGGUGCUUCUCAAAGAACAACCAGAAGGGGGACUUACGCAAUCUUGCAUUGAGUGCGGAGUCAUCACCACCAAUCAGGAUUGGGAGACCGCGGGGCACGAUGUACUGGCAGGCUAUGACGGCAAGAUUGUACCUGCAGUUGUCGUCGUACGUUCCUGGCUCACCAGUGUCACGACCGAGAUCCUUUUCGCCAAUGAUGUCAGAGUAGACACCAACUUUCCAGGACACGUAGAAUUCGGACAGAUGCUUGGGGAUGGAAAAGCGAGCUUGG  >Ss-AA_clean.1_(paired)_contig_9241  GGCCAAGCAACCAGCAGGCAAAUUCGAUAGCCACUUCGUCGCAACCAGAGUCCCAGGCGGUGUAAUCAUUGGCGGUACACGAGCCGGGGCGCCAGAACUCGCGGUACCAGGCGUUCAUUUGGCGUGGGGAGUUGCGACAAUGCAGGUACGUGGUUGGCAAACGGUACUUCAGGGCGAGGUUCUCAAGGUACAUUGCAAAGGGGGCGUCCCGGAAGAUUCGGAUGUGGUUGAACUCAGAGAUCAUCUGGCCUGCCUUUGCGGGCAGGAACCGGGCCUCCUCCUUCUUGACGUACUGGGUCUUCAUGAAGAGCUUUGCAUAACGGAGAGGCUGGUCGAUGUCAUUCUUGUUGACGGCGGAAUCAAUCUGACGGGCGGAUCGCUUGGAUACCCAGGGGGAGUAUGCGUCCCGGCCUGCACGCUCGAAGAGUUGUUUGUCGAACGACUGGCGUUCCCACGCGGAGACGUUAAAGAACUUCCUGAAACCUACCUUUAUCUGCUUGAGCCGCUGGCCAUCCAUCCGAGUGAGCUUGGUCUUUGAGGCACCCACAGUGAUACGCUUGGCGAUGGCCAUUCUCUCAGUCAUGACGUCGCCACGAGAGUGGCGAAGAAUGGCAUUGGGGCCGUCCUCGACAUGUUGUUGUGUGGCCCAGGUAUACUCGCCCCGAAACUCGCGAUCCUUGGGGUCAAGUAGAGUAUCUACCAUCAACGCGGGUUCAGGGGCAGCAAUCAGACGGGCUGGUGGGGGCGGGGCGGAGCGCUCAGAGGGCAUGGGGCCGAGGCGGUGAUCAGCGGUAAUCGGGGCAACAAACUUGAGAACCUCGCGGACAUGCUCGAGCUUAUGGACGUUGGCGGGGGGAGCUUGCCAUGAGAACGCGGCUCCUCCGGCAGUGCGUGUGGAACGCAUCGCGGCUUCAGAGCGGGGUGUCCAGAAGUCACCAAGGAUGUCUCUCGGGCGCUCGUGCCAGUCCAGCCGAUCUUGUUCCGUUACGCGUCCCACCACGACAGCGGGGGCGGCUAUUCCAAUGCUGGCGAGGGACGCCGCAGAGAGGCAUCUCGACAUGUGGCCUCGAACAGCUCGCGAAACCAACAUCCGGGGAUCGUCGCGAAGGGUCAAGAUGCCGCGGGAGUUCUCAGAGGCGCAGGCGAGAAUUGCAGAGAUGAUCGUGCUGCGCCCAUAGGAAGACUCAUCCACCGUGCUGAGGGAGCUAAGGGGACCCAUGUGAAGCAGCAUGUUACCACGGGCCCGGGUUAGCGCGGUCCACCAAGCGUGUUCAGUAGCGGUUUGGGAGAGACCUCCCAGGUCAAUGCUCACAUCGCCGUCGAUCGUUAUACCCUGGCUGUCGCGGAACGUGAGAGUCUUUUGGCCGCCAGAGUUUUGAGUCUCAGCAAAGCGGGGGGACACCACCAGGAAUGGUAUGUCGGGCAGGACCUUAGAGACCAGGGCGAUAACGCCGUCAUAGGCCGUGGCUGCACCAGCGCACGGCAAACCGAACAAGGACGAUAUACGAGGAGACAAGCGGUAUGAGACAGUGGCGUAACGGUCAGAAAGGUUGGCGAGCCUAUCAGAAGUGGAAGGCAUCGAUCGGUCAAUAACGUCAGUGGCAGGGAAGGCAGUGCGGGCUUGACAUACGUCGAAGGUGGUGUAG  >Ss-AA_clean.1_(paired)_contig_5513  GGGUGGGCAGGGAGGGAAAACCAUAGCGGGUGCAGACGCGAAGGAGGACAGAGUACGCUGUGGCAAGGUGAGGAUCCUCAGGGGCAGAGUGCUCAGCAGAUUCUCGAAUGGCCAGGUCUAUUGAUCGCCAGUAGUCAGGGUCAUUGCGGCCAAGAGCCACGCCAUACUGAGCCCGGUGCAGGACCACCUGUGGGCCAAGCCGUAUGUUGUCGCCACCGAAUUCAUAACCGCAGAACAAGAGACGGGAAGACUGUUCAGGUUUCGCGGUCAUCUUCCAGCCCUUGGAUUGAAAGCCAAUCUGGCGAGUAAAGUUGCCGGCCAGGAUGCAGUCAUCACCAGAGAAAGCAGCAGGCGUGCCGAGGGGACAGUCAAGGGAGGCCCCAACGAGGGCGGCAUUGAGGAGGGUGUUGACAAUCCAGGUGUAUCGAUCACCAGACUCAACCUUUGGCACAUGGGGGCCAAGGUGAGAACGGGUGAACAGGCGCUCGUGUCGGUACAUCCGGAUAUAGUCCUCAGGUAUGCCGCUUAGGCCAAGCAACCAGCAGGCGAAUUCGAUAGCCACCUCAUCACAACCAGAGUCCCAGGCGGUGUAAUCAUUGGCGGUGCACGAGCCGGGGCGCCAAAACUCCCGGUACCAAGCGUUCAUUUGGCGUGGGGAGUUGCGGCAAUGCAGGUACGUGGUCGGCAGGCGGUACUUCAGGGCGAGGUUCUCAAGGUACAGCGCAAACGGAGCGUCCCGAAAGAUUCGGAUGUGGUUGAACUCAGAGAUCAUCUGACCCGCCUUUGCGGGCAGGAACCGAGCCUCCUCCUUCUUGACGUACUGAGUCUUCAUGAAGAGCUUUGCGUAGCGGAGAGGCUGAUCAAUGUCGUUCUUGUUAACCGCGGAAUCGAUCUGACGGGCGGAGCGCUUGGACACCCAGGGGGAGUACGCGUCCCUGCCGGCACGCUCGAAGAGCUGCUUGUCGAACGACUGGCGUUCCCAGGCGGAGACGUUGAAGAACUUCCUAAAGCCGACCUUUAUCUGCUUGAGCCGUUGGCCAUCCAUUCGAGUGAGCUUGGUCUUUGAAGCACCCACAGUGAUGCGCUUGGCGAUGGCCAUUUUCUCAGUCAUAACGUCGCCCCGGGAGUGGCGAAGAAUGGCAUUGGGACCGUCCUCGACAUGUUGUUGCGUGGCCCAGGUGUACUCGCCUCGAAACUCACGAUCUUUGGGGUCGAGCAAGGUGUCCACCAUCAAUGCAGGUUCAGGGGCAGCAAUCAGACGAGCUGGCGGGGGCGGGGCAGAGCGUUCGGAGGGCAUGGGGCCAAGGCGGUGGUCAGCAGUGAUCGGGGCAACGAACUUCAAGACCUCGCGGACGUGCUCAAGCUUGUGAACGUUGGCGGGGGGAGCUUGCCACGAGAACGCGGCGCCUCCAGCAGUACGUGUGGAACGCAUCGCGGCCUCAGAGCGGGGUGUCCAGAAGUCACCAAGGAUGUCCCUCGGGCGUUCAUGCCAAUCCAGUCGAUCUUGUUCGGUUACCCGCCCCACCACGACAGCAGGGGCGGCAAUCCCAAUGCUGGCUAGAGAAGCGGCGGAAAGGCACCUGGACAUGUGGCCUCGAACGGCUCGCGAAACCAACAUCCGGGGGUCGUCACGAAGAGUCAAAAUGCCGCGGGAGUUCUCAGAGGCGCAGGCGAG  >Ss-AA_clean.1_(paired)_contig_5512  GCCAGCUGGUAACGGUCAAAACGGCCGAUAGUUAAUAGUUUAUCCACAUAAUGGUGUUGCACAGUUUAUACACGUGUGAGUGUGGUCCAUGUACGUCUAUUCAGACGAAGAGUCACACCACGCUCGAAAGCGGGGUGGGCAGGGAGGGGAAACCAUAGCGAGUGCAAACGCGAAGGAGGACAGAGUACGCGGUUGCGAGGUGAGGAUCCUCAGGGGCAGAGUGUUCAGCAGAUUCCCGAAUGGCCAGGUCAAUCGAUCGCCAGUAGUCAGGAUCAUUGCGGCCGAGAGCCACGCCGUACUGUGCUCGGUGAAGGACCACCUGCGGGCCAAGCCGUAUGUUGUCGCCGCCGAACUCAUAACCGCAAAACAAGAGACGGGAAGACUGUUCAGGUUUCGCCGUCAUCUUCCAGCCCUUGGAUUGGAAGCCAAUCUGGCGAGUGAAGUUGCCGGCAAGGAUGCAGUCAUCACCAGAGAAACCAGCGGGUGUGCCAGUAGGGCAGUCAAGGGAGGCACCAACGAGGGCGGCGUUGAGGAGGGUGUUGACAAUCCAGGUGUAUCGGUCACCAGACUCCACCUUUGGCACAUGGGGGCCAAGGUGCGAGCGGGUGAACAGUCGUUCGUGUCGAUACAUCCGUAUAUAGUCCUCAGGGAUGCCGCUAAGGCCAAGCAACCAGCAGGCAAAUUCGAUAGCCACUUCGUCGCAACCAGAGUCCCAGGCAGUGUAAUCAUUAGCGGUGCACGAUCCGGGGCGCCAGAACUCCCGGUACCAUGCGUUCAUUUGGCGUGGGGAGUUGCGGCAAUGCAGGUACGUGGUCGGCAAGCGGUACUUCAGAGCGAGGUUCUCGAGGUAAAGCGCGAAGGGGGCGUCACGAAAGAUCCGGAUGUGGUUGAACUCAGAAAUCAUUUGACCAGCCUUUGCGGGCAGGAACCGGGCCUCCUCCUUCUUGACAUACUGGGUCUUCAUAAAGAGCUUAGCGUAGCGGAGGGGCUGAUCAAUGUCAUUCUUGUUGACGGCGGAAUCAAUCUGACGGGCGGAGCGCUUAGAGACCCAGGGGGAGUACGCAUCCCGGCCAGCACGUUCAAAGAGUUGCUUGUCAAACGAUUGGCGUUCCCAGGCAGAGACAUCAAAGAACUUCCUGAAACCGACCUUGAUCUGCUUGAGCCGUUGACCAUCCGCCCGAGUGAGCUUAGUCUUUGAAGCGCCCACAGUUAUACGCUUGGCGAUGGCCAUUUUCUCAGUCAUGACGUCGCCGCGAGAAUGGCGAAGAAUGGCAUUGGGACCGUCCUCGACAUGCUGUUGCGUGGCCCAAGUGUACUCGCCUCGAAACUCGCGGUCCUUGGGGUCAAGAAGGGUAUCAACCAUCAACGCGGGUUCAGGGGCGGCGAUCAGGCGGGCUGGUGGAGGCGGGGCAGAGCGCUCAGAAGGCAUGGGACCAAGGCGGUGGUCGGCAGUGAUCGGGGCAACAAACUUCAGGACCUCACGGACGUGCUCAAGCUUAUGUACGUUGGCGGGGGGGGCUUGCCAUGAGAACGCGGCUCCUCCGGCAGUGCGUGUGGAACGCAUCGCGGCUUCAGAGCGGGGUGUCCAGAAGUCGCCGAGGAUGUCUCUCGGGCGUUCGUGCCAAUCCAGUCGAUCCUGCUCCGUCACCCGGCCCACAACAACAGCGGGGGCAGCGAUUCCGAUGCUGGCAAGGGAAGCCGCGGAAAGGCACCUGGACAUAUGACCUCGGACAGCUCGCGAAACCAACAUGCGGGGGUCGUCACGAAGAGUCAAAAUGCCGCGAGAGUUCUCGGAGGCACAGGCGAGAAUUGCUGAAAUGAUCGUGCUGCGUCCAUAAGAUGACUCAUCCACCGUGCUGAGGGAGCUGAGGGGACCCAUGUGGAGCAGCAUGUUGCCACGAGCUCGGGUGAGUGCGGUCCACCAGGCGUGUUCAGUAGCGGUUUGGG  >Ss-AA_clean.1_(paired)_contig_13466  GCGAGUAGAGUCAGCGAGCGUUUGCGUGCGACUAAUCUGCAGUAUGUGAUGAGCAAACUUGGACUGCAGAAGGGAGACAUGCAAACACUCGUUGCCAGGAGUAACCAACUUGUUGGCCGUAAGCCAGGACCGAGCGGCGAGAGGUUGUUCAUACCGGCCACCCUUGUCACCCUCAGGAAAGUAUAUGAUCUUGCCGUUCUUACAGUAUCGGAAGUCAUACAGGGAGGGGGUAAGGGCAGGGAGGUCCCAGAGGGUCUCAGGUGGUAUCACGCAAGUGGCAAUCAGAUGGUUGAGGUGUGGGUUGGCGUCGAACCAGGAACCGACAGUUGGGGCAUCUAACAUUUGGAUAACAUCAUGAAGGAACCAGACAGGUGAGGACAUCUUCGGGGGCUGGUAUUCAAGAUCAGCGAAGCUGCCAGGCGUGUUUCGCAGCUUGGUGGACGCGUGAAUACCAGAAGCGGGGCGGAUGUUCAUGUUGACGCUUUGGUAACGAGCGAAAUCGCGACCAUCAAGAUGGAGAUUGUAACGUUCAGAUGCGGGUGGCAGCUUAACGUCGUCAUAACACAUUUUCUUGAUCUUGGCCUCCUUGAUAAACAUUGGAGCCCAAUCCUGGUUGCGCAGAUUUUCACGAGCAAGGGCAAGAUUGCGGUUUUCAAGCGCAACAUGAACAGGGUGAGGAUGAGGGGGGGGAUGAGGAACAGGGGCAGG  >Ss-AA_clean.1_(paired)_contig_15254  UUAUCCAAAUGUUAGAUGCCCCGACUGUCGGUUCCUGGUUCGACGCCAACCCACACCUCAACCAUCUGAUUGCUACUUGCGUGAUACCACCUGAGACCCUCUGGGACCUCCCUGCCCUUACCCCCUCCCUGUAUGACUUCCGUUACUGUAAAAACGGUAAAAUCAUAUACUUUCCUGAGGGUGACAAGGGCGGCAGGUACGAACAGCCUCUCGCCGCUCGGUCUUGGCUCACCGCCAACAAGCUGGUCACUCCCGGCAAUGAGUGUUUGCAUGUCUCCCUUCUGCAGUCGAAGUUUGCUCAUCACAUCCUGCAGAUCAGUCGCACGCAGACGCUCGCCGACUCAACUCGCAGUUUCGACCUACCCAGCCUCUAUGAGGUGGGUCGACACAUCAUGCU  >Ss-AA_clean.1_(paired)_contig_82  GCAUCGGAUAUGCAAUCCAUUUCUUCCUACGCGUAUAUACCUAGACAACGGGUUUUACGGUUAUGCUCAGGCCUUCUACCAUCAAGCAUCAAAGCUCUUGCAGGUGGGGAGACGCGCGUUGAGAUCCGCAGGCUUGCACCUGGCUCAACACCUCCCUCUCUUGCAGUUAGCACCGUCCUACUCCACUAGAGGACUGCACUGGGCGCGCCGGAUCCCGUCAAGGAUCUUAAAGAAGGUCGGGACGUCGGACAGGCAGUGGCGUGAUACCCGAUUAAGCAGUCUGACGUUGACUCCUCUCGGUACUACCAUAGCAUCCAAACCAGUUGUACACAUGACUCGCCACGCUUGGGUUUCCCGUCGCUCCCCAAGCCUGAAUCACUACACCAACUGGUGACAUCCUCCCCAACUCUCGGACUACCUCACCCCCAUGCUCUCUAGGUUUCGUGGUGUUAGGGGAAUUACCCCCCUGCGUCGAACCCACAAAUGACGCCAUGCACAGACAACCGUCUGAACACGCCAGCUGCACCCCUUCCCCACUAGUAUACUUCGGAAUACUCCGCAUGCCUCACCGAGCUGUGGUCCAUGUCGCUAAUACAACGAUGUAGACUGUGACCUGUCUACUUGUAGUUCGCCGACGAUCCUUUCACCAGUGCCCCAGAGGUUAACUCCACUUCCCCCGCUUCCUCACACCACCCCAACAACCCACCCAGCAAUUAGAGCUCCAUCCGGCGUUGACUAUAUCUAGUCUUUCAACCGCCUUCCGGACAGGAAGAGGUCAACUCUGUUGCCAACCAGGUCAGUGUCCGCGAAUGGGCCAUCGCCGACGUGGACAUCCUCUCGCAAGGUUGUCCAGUCCCAAUGAUCCACACUCGCCACUAACUCGGCCUCCAUCAGAAUCUGCUCCUCCACACCGAUCCCCCAGGCUCUCUGGAAGGAUAAGCGAGCAUCCAUGCACACACCCCUGGUGCCCGACUUGGCAGCAUAUGCCGGAGCCAAAGCGUGUCGCAGCCGGUCCUCCAACACCACGGAAGGGUCCUUCAGGUCGCGGUAUGCCUCGAGACGCUCUAGAGCCCUGGCAAAGUAGGCCUCUAGAAGCGGUAUACCGCGGGACAGUGAUAACUCAGCACGGCACACAGCCUUCAACGCAGGUCCAACAUAUACGCUCUGGUCAUAAUGC  >FirsU_Contig1  CAGUGAUAACUCAGCACGGCGCACAGCCUUCAACGCAGGUCCAACAUAUGCGCUCUGGUCNAUAAUGCCGAUAACCAGAGAACGCGUUAGACAAGGUCUUCAGUGGGUGCCUUACCAUAGUNAUAGUGGUCGCCGUUAUAGCACGGUUUACACUGUCCAAAUACAACCUCCUCGUAGACAGUNAGUAGGCUUCUCUACAGUGAGCUCCUGCCCUACAACAGUGUGUACCAUGGAACUGAAGAANGNNNNNNNNNNNNNNNNNNNNNNCCGUACCCUCUCGGCCAUGCACGUCUCCACGAACACCNAAAGCAUUAUCACCGUCAGCCAGAAAGGUUGUUCUGAAAGCACCAAGCGAAGACUCAGCUNGAGCGCAGGAUGGCAUCUACACAAGAUCCCAUGACUAAGGUGUUGCCUAAGCCAGUGUUGNAAAUCUCCAGAUGCGCGACAUCCCUCCCUCUCAAACUUGAUCCCACCAAGUGUUUUUCCUNUUCAACUUCCGUUGAGUCUNNNNNNNNNNNNNNNNGCUUCGAAAGCCUUACCAUCUAUCUNCGAAGACUGUACACUCCCCUACUGACUCCAUCUUCCUCCGUAUCAGAGAAGCCCUCUGCUNCCCCGUUCAACCCCUUGCCUACGACUCGGGUUGGUGUGACACCCCCUCUACCGUACUUCCNACCGCCGCCAUAACGCGUGCUCGAGUGGUUUCAAGUAAGAGGCCAGCUCUAGGUUGAAUCNUUGGCGAACGACACAUGAUCAUUCUAGGCUUACUUUGUUUUAAAAGAGGGUUAAACUUUUNCAGCCUUGAGGAACCCAGUAAGUCGUCUGUCGUACUUAGACAACCGGGGUUCCUCAACCANGAGAUUCGAGAGCUGCUUGGUAUCGCUGUCGCAUCCUACCCGAAUAGGACUCGACCACCUNGCUGCUUGGUCCACCUCUGUAUGCCGAGCCUUGACAAGAGAACUCCCACUCUUGCAAAUGNCUUUAUCUAGCUCCGCGCAUACAGGAUCGGCGGGUGUGGGACCAAGAGUCCUGAGCCGCANGGGCUGCCUCCUCGUUAUGCACACAGUUCGCGUGUACAGCGGGAGCCCACAGCCCAUCCANCAGAAGGUACCCAGACUCGGUACAUUCUUCGUCUUGCCUCUGGGCCGCAACUAUGUCGUGNUGGUGUCCAACGAUGGUAUGACCAUCCUGGCGCCUGGUCGUAUUUUAUGUUGUUCCUUGGNAACCGACACAGACACCAAUACCCGAAUUGGAUACACCCCACUAAGUUGUUGCCNCUAGGCAAGACCACCCCGGUGACGCACUCAACCCGCAUAACAUCGCCUACAGCCCUGGUANAACUGUCGGAAGAGCGAUGUGGUCCUGCCGGCGUGUAUGUCCUCCGAUGCCCUCUAGGAUNUGCCUGCCGCUCACUCCACCCAAAGACUCGUUGGCCAUUUGCUCUGGUCCGCUGACUCUCNAUAGCCAAAGCAACAGUGCCCGCAAGCACCAUUGCUAGGUAUGAGUCGGUCAUGCCCGACNGCAGUAGCUAUCUGCCUAGCCUUUGCGGUAAGAGCGAGUUGCAGCUCCCUAGUCCUCCGCNUUGUUGGUUGCCGAAACAACCAACCCUGCCAGAAGACGCGCGGACGCGGCUAACACACUANCCACCUAUGCGCACCGUCAUGUAACAAUCCUCUGAGGGUUCGUCCUUGGCGGUGUUGUUN  >FirsU_Contig57  AAACCUAACUGCUUUAGUGGCUGGUAGUCGCACUACUACCCUGCCUUUAACCUCACCUUCNUACGACGCCCAAGUCCACCUCGGUGGACGUGGUUACGCCAGGUUGUGAUGGUAGCUCCGCNCAGUGACAACGGUGGAUAUACCAUAUAGGGUACAUCCCACCGGCCGCUGGAACCCUGUAANGUAGUCUUUGAUAACAUACUCAGCAGGCGGAGCUGCCUUCGGCAGCCUGACGACAGGGGUNGUGCCUCCCCUCGUCGUGAUCGAGGGAGAACGCUGAUUCAGCGAAGCUCUCGAUCGGGUUNUGGAGUUGUUGAUUCUAUUUUGUUUUCCCUUAAANAAUUUGAACAUCAUAAAAUCAUUUGGNUGUGAGCCACCACCUCCUGUGCGGGUGCGCCGCUNAGUCGCCAGUCAACAACGGUGGUUGUGAGUCCACCAACUAACAGAUACCGUCUGUCACGGNGAAGGUGCCUGUGGUUGCAGGCGCAGACAUCUGUGGGCCGUCUGCUUGGUGUCGGUGGACNAUCCUCCAGAGACCACUGCAUCAACCACCGAGUAGCGUCCUCCAGGGUCAUAGCUAUACUNCAUCGCUACCCUGGGCUCCGAUCGCACAUCCGGUCGCGGUCAACACCUCCCUCUCUUGCANGUAAACACCGUCCUACUACCCUAGAGGACUGCACUGGGCGUGCCGGACCCCGUCAAGGGUNCUUAAAGAUGGUCGGGACAUCGGACAAGGCAGUGGCGUGAUAAUCGAUUGAGCAGUCUGANCGUUGACUCCUCUCGAUACUACCAUAGCAUCCAGAACCAGUUGUUUACGUAACUUACAACNGCUUGUGUUUCCCGUCGCUCCACAAGCCCAAGUCACUACACCAACUGGUGAUAUCCUCUCNCACCUUUCGGACUACUCCACCCUACAUGCUCUCUAGGUUUCGUGGUGCUAGGGGAAUUACNCCCUCUGCGUCGAACCCACAAAUGACGCCAUGUACAGACAACCGUCUGAACACGCCAGCUNGCACCCCUUCUCCACUAGUAUACUUCGGAAUACUUCGCAUGCCUCACCGAGCUGUGGUCCNAUGUCGCUAAUACAACGAUGUAGACUGUGACCCAUCUACUUGUAGUUCGCCGACGAUCCUNUUCACCAGUGCCCCAGAGGUUGACUCCGUUUCCACCGCUUCCUCGCACCACCCCAAUAACNCCACCCAACAAUUAGAGCUCCAUCCAGCGUUGACCAUAUCUAGUCUUUCAACCGCCUUCCNGGACAGGAAGAGAUCAACUCUGUUGCCGACCAAGUCAGUGUCCGCAAACGGGCCGUCGCCNGACAUGGACAUCCUCUCGCAAGGUUGUCCAGUCCCAAUGAUCCACGCUUGCCACCAGCUCNAGCCUCCAUCAAAAUNNNNNNNNNNNNNNNNNNNNNNNNNNNNNNNNNNNNNNNNNNNNNNNNNNNNNNNNNNNNNNNNNNNAAAGCGUGGCGCAGCCGGUCCUCCAACACAACGGAAGGGNUCCUUCAGGUCACGGUACACCUCGAGACGCUCUAAAGCCCUGGCAAAGUAGGCCUCUAGANAGCGGUAUACCGCGGGACAAUGCUAACUCAGCUCGACACACAGCUUUCAACGCUGGUCCANACAUAAGCGCUCUGGUCAUAAUGCCGAUAACCAGAAAACGCGUUAGACAAGGUCUUUAGCNGGAUGUCUCACCAUAGUGUAGUGGUCGCCGUUAAAGCACGGUUUGCACUGUCCAAAUACANACCUCCUCGUAGACGGUGGUAGGCUUCUCUACGGUCAGCUCUUGCCCUACAACAGUGUGCNACCAUGGAACUGAAGAAGGAGCGUACCCCCUCGGCCUUGCUCGUUUCCACGAACACUAAANGCAUUGUCACCGUCAGCCAGAAAGGUAGUUCUGAAGGCACCAAGCGCGGACUCAGCUGAGNCGCAGGAUGGCAUCUACACAAGAUCCCAUGACUAGGGUGUUGCCCAAGCCAGUAUUGAAANUCUCCAGAUGCACGACAUCCCUCCCUCUCGAACUUUAUCCCACCCAAUGUCUUUCCUUUCNAGCUUACGUUGAGUCUGCAAUAGGUCGUGUAGACGUGCAUCACGAGGGUACACUUUCUUGNUAN  >Ss-AA_clean.1_(paired)_contig_224  UCUGGUUGCAGUCGUAGGUAGGGCCGCCCAGGUGCCGAGGCACCCGCGUAAUAUGGUAAGAUAUUGUCUCCCUCCGCGGACUUUUGGGGUUCGACGUGUGGCAUGGUCUUGCCACGGAAUCAUAUCGCUUCAUCUUUCGCGCCAAUUAUGGUAUAUGGAAACCUCCACUACUAUGUUAUUCCGCCACAUUAGUGGUCAUAGGGACGGAACGUCUUGUCAAUGCCUCCCACAAUGGCGAGGAAUGUGUGCGCUUGAGAGCUUAGACCCUACAGCAACACAUAUGAACCUCGCAUGGAAGUUACAACGUUUUCUACCCAAGCGCAUCGGGUAGCAAGCUACUCCCGCUGCGCCAGAUGCCUAAUAUACACCAUGCACGCCUGUAUUGUACACCAGGUAAUUACAAUCAAAGCUAUGUGUUAACGGUUACACGAACCUAGGGCCAAGAGUUCCACCCGUGCCGUACACAACUAUACCUAGAACCGCAGUAUCUUUACACUCCGUCCAGCUGCAAUGCCCUUCGGGGCGGGCCCCCCCGAGAGGGGGUCCUGAGAAUAGGGGGCGAUUUCUGCGUUUGCCGCGUCGAUUUGGUUGAGUUGCGAGCAUCAUGGCGGAACAGGACAAAGCGCGAUAACGUAAUAACACUUUAGGGAUUCGUGUCUACGUGCUACCUUGUCUCCUUACCAAUCAUACAUCCGUCACGAUCUAAUAGGGCAUGAAUGCGGGGUUCCUCCCCACCCGCUACCCCUUUCAAUUGACGCAAUGUUCCUCGACUUUCCUCCUCUAAACGACAGCGCAAGUGGCUAGUACAUUUUACGUAAUCUCGUGAGAUGCCACCUCACAUAAAGCAAUUAACCAGGGUGGGUGAUUAACCCGUAUACUGGCAGUGCUCAAUUACGACUGGCCCGCUCUAUUUAUGUGCCUAAUACAACCAUGGAGUUGGCGCACCAUUUCCUUUCUUCUCGUUAGCGAUAACAACUGGAUCAAGCUUGGGAUCCUGACUACUAGUAGUUUUCCGGCGACCUUCCCUCUCCAGGUUUACCUGACGGACCUCCUUAGCAAUGGAACCUUUACCAUCACCAGAGACAACAAACAAACCUGCACCCGUGUCUUCACCUAUAACACCACUUCGGUACGAGUGGCCGCGCAACAAGCAUGGACGCGCGUUAACGCCCACGACUGAUGAAUUUGUUGCCCGGAUUCCACCACCACGAGGCACGCGCCUGUGAUCCUUUACACAGAGCCCCGCUUUCCUAUGCGUACCAGAGUUCCCACCUUGAUCUUUUCCCCAGCUUCAACAGCCAUGCUAGCAAUUCUAGCAGGUUCCGGCGGUGAGUAUAUCUACUCCUUCAAACGCCUUCCCCCCAGGAAGAGAUCAACCCUGUUUCCGACCAAGUCCGUCUCGUCGUCCGGGCCAUUCCCCACUAGCACUGAGUCAAAGUGCUCGGCGGGUGUCCAAUGGUCCAGACUCUUAGACAGCUGAUCUUCUAUCAGGAGCUGCUCCUCCACACAGAUGCCCCAACUCUCGGCAAACGAUGAGCGAGCAGCGUCCGUACAUCCCUUGUACCAAUAAUGUUCGCCAGCCUUGAACGGGAUUGAAUGAUGACCAUACUUCAGUCUUUCCUCUAAGAAACUAGUUGGAUCCUUCAGGUCGCGGUACGACGCGAGUUUUGCUAAAGCCUUGGCAAAGUAAGCCUCAAGCAACGGGAUACCUCUGGACAGAGAUAGUUCGGCUUGACAUACCGCCUUCAAAAGGGGCCCUGUGAACUUAGACUGGUCGUAAUGCCGAUAACCAGAAAAGGCGUAAGACAGGGUUUUCAAAGGGUGUCUAACCAUGACGUAUUCCUUGCCGUUGAAACAAGGUUUACAUUGUCCAAAGGUAAUGUGUUCCAAGACAUCAACAGGUUUCUCAACCGUCAUCUCGUGACCACAAACAGACGAAAUAUAACGUGCAAAAUUGGUUCUCAACCGCUCAGCAACAAUUCUGUCAACGAAAAGUAGUGCAUUGUCUCCAUCGGCAAGAUAGGUAGCCUUAAAAGGACCGAGUUCGGUAACGGCUAAGCGCAGGCCGGCUUCAACUGCAGAUCCCAUGAUGAGGGUGUUGCCCAAACCAGUAUUGAAAUCUCCUGAAGCUCUACAGCCCUCCCGCUCAUACCUGAUACCGCCCACAGUCUUACCUAUCAACUUCCUCUGGGUGUUCAACAAAUCGUUAAGUUCCUUGUCCUUCCUAAAAACCGCUUUAUACACGCUAUGUUCCAAAUCUAUCUGCAACAACGAGACGUGGGCCUCGAAAGCCUUACCGUCAAUCUCGAACACAACGCAGUCCCCCACGGAAUCCAUUUUCUCCUUAAGGAUGGCCGCUCUUCUGGGCCCAUUCAAUCCUUUGCCUACAACGCGCGUAGGUGUGACACCCCCCAUACCGAAUUUCCAACGCCUCCAGAGUGCAUGCUCCACAGGCUUCAGGUAUGAGGCUAGCUUAAGGUUGAAUCUGGGAGAACGACACAUGAUCAUUCUCGGCUUACUCCGUUUAUGCAACGGGUUGAACUUUUCAGCCUUGAGGAAAGCACUGAGUCGGCGAUCGUACUUGCCCAACUCAGGUUCCUCAACCAGUGAAUCAAAAGCUUCCUGAUAUCUUCGUCGCAACCUACCGGUGUAGGAUGCGACCACCUCCUCUAGGGUCAUCCGCUCAACCUGAAGCUUGACUAAGAGUCUACCCAACCUCUUAAAUGUUUGCUCAAAAUCAGGAUUGACCGGUUCAUCCGGUGUUGGCCCUAGGGUGCGCAUCUUCAGCGCUGCCUCCUCAUUAUGUACGCAGUUUGCAUGAACGCCAGCCGCCCACAAUCCGUCGACCGCGGGUACCCAGCAUCUGUACAUCUUCCUUUUGCCCUCCUCGCAACCAAAACCACGCGAUGGGUCUAAUGUCGGCAUAACCAAACGGGCAUCUGCGCGUAGCGGCAGUCCCUCGGAACUGCCUACGCAAACACCUAUGCCUGUAUUAGAUACCCCCCAUCAAGUUUUGGCCCUUGGUAGGACCACCUCCUCUGGUAGUUUGCCUAGAACAAAGUAUUUCAACAGUCCCUUAAAGCCUGGUACGUAUUGUUCGGGCAUCUUUCCUUCAGCCAUAGCCAUCGAUUGUUUCUGUCUUGCCAAGCCAUUGGGCCCACCCAUAGCAAGGAAUGCAUUCACUUCAGUGAAAUUGGCGGUCAUUGCCAUGACAACGGUACCUGAAAGUACCAUUGUCAUAUAGGACAACCCCAUACCAACCUCAUUUCCGUACUGCAUAGCCUUAGCUUUGAGCGAGUACAACAACUCUUGUGUUUUAGAUUUGAACAUGGAAGCUAGCAAAAGCUUGUUCAACAGUCUAAGUGAAACGCACAACGACCUUCCUGCUUUUUCAACGGAAAGAUACACGUCAACGACCUCCUGGUCUGUUGCGCUUGCGAUCUUGACAACGACUUGGUGACUACCAACCACCUUUACUUCCCUUCCGAGGAUAACUUUCAAAUACCUUCUAACGUGUUCAUCGGAGGUCUUACCACCACAAUUUUCACGGUCGAAAAAGUAUUCGCAAGCAAUAGACUCUAGUAUGUGAGUCUCCUUUCCUCGUCUGCGAAGUCUGUUGCCCGAGUAGACGAAAUCAUCUUCCACUGGGGCCUGUGCUAGCUUCACACCAGCGCCGACUGGUGUUAACUGUUUUUGCACAUAAGGCAACCGCACAGUUGCUUCUGUUUUAACCUCACCUGUUAUUCGCUCAUCUUCCGGUAAGAAGAUGCUCGAACAAGUUGCUUCCACCUCCAAAGGUUCCUCUGGUUGCCAGUAGGCAUUAAGACAUGAGAAACCUCCGCAUCUCCACCUACCAUCUUCAUACUGAAAGUCAUUGACCUUGUGAUGAUUUUGGUAGUAGUAUGGAUUACGGGGAGGCGCUUGAGGCAACUUGACGAUCACGGGUUGCUUCCCGCGCGAAUGGUCGAGGGAGAACGCUGUUGCAGCGAAGCUCUCGACCGGGGUCGGAGUGGGUGACGAUUUAAAUAUGUUCCAUCUCAUUCUAAAUCAUCUGGUGAAGUCCACCACACCCUGUGGGGAGCUGCCCCUUAAGCGCCAGACAACGCCGCUGUCUUUCCAGCAGUCAAGAUUCUGUCUUUCCAGAGUCAGGGAAAAUGUGCGGUGGCAAUU  >FirsU_Contig60  ACAGACACCAAUACCCGAAUUGGAUACACCCCACUAAGUUGUUGCCCUAGGCAAGACCACNCCCGGUGACGCACUCAACCCGCAUAACAUCGCCCACAGCCCUGGUGAACUGUCGGAAAAGNCGAUGUGGUCCUGCCAGCGUGUAUGUCCUCCGAAGCCCGCUGGGACUGCCUGCCGCUCACNUCCACCCAAAGACUCGUUAGCCAUUUGCUCUGGUCCGCUGACCCUCAUAGCCAACGCAACNAGUGCCCGCGAGCACCAUUGCCAGGUAUGAGUCGGUCAUGCCCGACGCAGUCGCUAUCUGNUCUAGCCUUUGCGGUAAGAGCGAGUUGCAGCUCCCUAGUCCUCCGCUUGUUGGUGGCUGANAACCACCAAACCUGCAAGAAGACGCGCGGACGCGGCUAACACACUACCACCUAUGCGCACNCGUCAUGUAACAANUCCUCGGAAGGUUCGUCCUUGGCGGUGUUGUUGAGUUUGACUCGCUGNCGUCACUGUCAGCNUUUCCUCCCCAUCGGGUUAUCAAGCCGAGCCAAUGUCGAGCAGGCUGUUCGGAGGUCUUANCCACCUUCCCCAGACUGAUGCGACACGUCUCGCUUGGCGAUCGACUCUAAUAGACGAGUCNAAUCUUCCCCUCUUGCGGAGGCGGAAUCCAAACUCAGCAUCACGUAACUCACCCCGUUCAUUUUCCUAGAACCGACGGGCCGUACUUGGUACUGGCUUGAAUUCGCGACUCGGCUUCUUUUUCUCCNAAAGAAGCGGAGCCAGUGCUCGCUUCGGUUUGACGCCCAUCAAACCUAACUUGCUUAGUANGCGGGUGGUCGCACAACUACCCUGCUUUUAACCUCGCCUUCUACGACGCUCACGUCCACCNUCUGUGGACGUCACCACGUCAGGUGGUGAGGGUUGCUCAGCCAGUGACAAAGGCGGAUAUNACCAUAUAGGGUACAUCCCACCUGCCGCUGGAACCCUGCAAGUAGUCCUUGAUAACGUACNUUGGCAGGGGGAACAACCUUCGGCAACCUGACGAUAGGGGUGUACCUCCCCUCGUCGUGANUCGAGGGAGAACGCUGAUGCAGCGGAGCUCUCGAUCGGGUCCGGAGUUGUUGAUUCCAUUNUUGUUCCUUUUAAAAAAUUUAAACAUAAGAAAAUCAUUUGGUAUUAGCCGCCACCUCCUANUGCGGGUGGGCCGCUAGUCGCCAGUCAACGACUGUGGUGGUGAN  >Ss-AA_clean.1_(paired)_contig_29  UCUAACUUAGUGGGGUGUAUCCAAUUCGGGUAUUGGUGUCUGUGUCGGUUCCAAGGAACAACAUAAAAUACGACCAGGCGCCAGGAUGGUCAUACCAUCGUUGGACACCACACAACAUAGUUGCGGCCCAGAGGCAAGGCGUAGAAUGUACCGAGUCUGGGUACCUUCUGUGGAUGGGUUGUGGGCUCCCGCUGUACACGCGAACUGUGUGCAUAACGAGGAGGCAGCCCUACGGCUAAGGACACUUGGUCCUACACCCGCCGAUCCUGUAUGUGCUGAGCUUGAUAAAGCAUUCGCAAGAGUGGGAGUUCUCUUGUCGAGGCUCAACAUACAGAGGUGGACCAAGCAGCAGGUGGUCGAGUCCUAUUCGGGUAGGAUGCGACAGCGAUACCAAGCAGCUCUUGAAUCUUUGGUUGAGGAGCCCCGGUUGUCUAAGUACGACAGACGACUGACUGGGUUCCUCAAGGCUGAAAAGUUUAACCCUCUUUUAAAACAGAGUAAGCCUAGAAUGAUAAUGUGUCGUUCGCCAAGAUUCAACCUAGAGCUGGCCUCUUACCUUAAACCACUCGAGCACGCGUUAUGGCGGCGGUGGAAGUACGGUAGA  >Ss-AA_clean.1_(paired)_contig_381  ACAACCUGGCGUGACCAAGUCCACCGAGGUGGACUUGGGCGUCGUAGAAGGUGAGGUUAAAGGCAGGGUAGAAGUGCGACUACCAGCCACUAAAUCAGUUAGGUUUGAUGGGCGUCAAACCGAAGCAAGCACUGGCUCCGCUUCUUUGGAGAGAAAGAAGCCGAGUCGCGAAUUCAAGCCCAGUGCCAAGUACGGCCCGUCAGUUCUAGAAAAUGAACGGGGUGAGCUACGAGAUGCUGAGUUUGGGUUCCGCCUCCGCAAGAGGGGAAGAUUGACUCGCCUUUUAGAGUCGAUCGCCAAGCGAGACGUGUCGCAUCAGUCUGGG  >Ss-AA_clean.1_(paired)_contig_534  CCCCCGAGUGCAACUCGUAGAGCCGUCUUUCCCCCAAGCACGCCCACAAAAGAUUUUGUGUUGGAGGGUUCCUCCCUGGGGAACUCAUUGUCGUAGAUAGGGAUAUUAGAGGUAGACCUGGAUUGGCUCCCCAGCAUUCCCGACGCAUCCUUUUCCACCAUGACUGAGCUGGAUGUACACCAGCCAUGGCCAUCAGUUUUGACGGGGUCGUUGACCACCCUUGAUCUCUCGGUUUCUUUUAGCUAGUCUUGUUCUAGCGGUUUUGACUACCUGUCAGUGCCUCCCUGCGCCCCGUUGUUGCAUGCUCCAAGACACACAUGACGCCAUUUAAGUAGCUGGCCAAACUAACGGAUCUGCUACACACCUCUACCACCGAAGUGAGGCUUCACAAUAACCCGACGAGGACUUCCUUAAGGGUUCAACACCUAUGACGCUCCCGUGUCUCCACUAGGGUCAUUCUUCAUCUCUGCCUCCCCAUCUACUCCAGCUGACCUGCCCAGAGUUAGUGGGUGUGAACUACCAACCUCACAACCGUCAAUCCCCCUAUGUGGUAGUUUCAAACCAUGCGUCAUGCAAACCAGGCUCGGCUUGUCGCCAAUCAGUGACACCAAGGCAAUCCUUGUAUGUCUGAUCAGCGUUGUAUGAAAAGGAUUCUUCUAGCUCCUUCUGCUCCUCAGCAGUAAGUCCGAAGGCUCUUUCAAAGAACACCUGGUCUCCAGCAGCACAGCUCUCGACUCCCUCCGCUCAGCGAGCCACGCACCCAACAUGAAAUUGUCAACAAAGGCAUGCUCCUUCAGUUUCUUGUUCGUGUUUGUCACCUCCAUCAUGUGUAACGCCCAUGCCUGCAGCACUGGGACGCCAAGGCCAAGGAGAGUUCGCAUCUGGAGACACCCUGCAAAUAAGUGAGUGCAAAGUUGGGUUCUCUUAGCCACUUGUGGCUAGAGGUGGCGCCAGACAACACCUUGCGGUAGUCUCUGAUCAUGGUCCACCCAUACUUUGCACCCAUCCACACAGGUGCCGACUGUCCAAAUCUGAUCUCUUCGACCACACUCACCGGCUUCUCGACAACUAGCUCCUGACCCGACCAGGCACAACUCCGUGUGGCAAACUCCGCCAUCACCUGUGUCAAGUCCUCAGACUCGACGAAGACCAGGCAAUUGUCACCAUCAACAAGAACAUCGAAUUUGACUCCGAAACUCUUCAUGACAGCUACAACGACCGCCAACAUGACAAUGGUGUUACCCAUCCCCGUGUUGAAGUCUCCGGACGCGCGAGCGCCCACACGUUCAAACUUUGCUCCACACUCGAGUCGACCUCUGAGAACCAGCUGCUCGUUGAGCAAGGCAACCAGCUCCCUGUCACCAGGGAACGCGGCUUUGUACACUCCUUGCUCUCCUUGCAGCUGGCUGACACCAACAUGUGCCUCAAAAGCAGCUCCGUCCGCCUCAAACACUACACAAUUCUUGAAGUUGUUGUACUUGCGUACAAUAAGAUUUGCCCUCCGCCGCGGAUUGAGACCCUUCGCAACCAAUCGACCUUGUCCACCUAUACGCAUGCACUUGGCGGUUAGAGUCCCCCAGAGCCAAUGCUCAAAAGGUUUCAAUCUGGAGGCCAAUGUGAGGUUGUACCUAGGUGAUCUAGGACAUAUAAGCCGUGGUUUCAUCCACUUUGGCCCCACAUUGAACUUCUCAGCCUUGAGAAAAGCCUUUAUCUUCGCAUCGCGACGAUUCAACGGUUCGUCAUCUCUGAGAGACCUGCAUGCUUCUAGGUAUCUACGUUGCAUGGCACCCGAGUAAGACAUCGCCGUCUCCUCUAGGGUCCACUUGCCUUCCCGAAAGCGUCUGGCGUGUCUUGCCAGCGCACGGAAUACUGCAUCCACCUCCGGGGACACUGCUUGAAAAACUUGUGGAGGUAGUGUGGAAGCCAUCAAACGCAAUUUCAGCGCUUGGAUCUCGUUGUGUGCGCACCCCCUGUGCACCUGAGGUACCCAACACCCAGUAACUGGUGUGGGCCACCACGUCCUCAUCUGUCUACGACUCUCCUUGCAUAAGCCCAGAUCAACCCUAGCUAUGUCCAGGGACAUACCGACUGGUACGACAUCAUCAGGGAUGAUGCCGCAGCAGACAGCUGCUAGAUUAACCGGGCCUUCUUAACAGGAUGGGGAGAGAAGGGAUCCUAGACCAGCAUCCACCAGAACUGUCUUUGCCGCCUCUUCAGGCUUGGUGUCCAAACAGGCUCUCGCGACCGCACCUGGGAGGACCAAUGGGCCCAACCAGAGCGGGAGUUCUUCCUGCUUGAACCAGUCAACUGCUCGGGUUCUCAACCCACGUAGCAGCUCUGGUGUUCUCUCCCUGAGAAAAGAGUACUGGGACAACUUCGCGUCAAGGCCUGGGAUAACCAGUUCUUGACCCUCAGGAGACUCCACCACGACAUACGGAUGUAUGACGGGGUUUUGUUGCGAAGUGUUAUGGACUGCUCCGAUACCUAGGAGCUUCAUCCCUCGGGAUGAACAUGCUAACAGCGCGUUCAGUUCCGGAGAUCUUGCUGACGUAGGGAGGUCUGGUGUCCACCGCCCACGAAAGUAUUGACCAACGGAACCUACUGGUCCCUGUAGAAUGUCCUGGAUCAUGCGAGUAGCACGACCACGAGCCCGGAGACGUGGCACCCGCGAUAGGGCACCACUAGCACUCAAAUGUGCCAAAUUCUCUUGCUGAGCCAAGGUCUGUCCAUAACCUAUGGCUUGAUUGACCCGGUUCGCACCGGGCCCUUGCUCAACAGGCAAGGGUGCUGUUGAGUCGCCGAAUUGUAGAGUGCUACGGACCCACCUGAUGAGGAGAGGCAGGUCGUAGCAGAGCAUCGCCAAGUGAACCCAACCUGAGUACAUCAAGAAGGUUGGGGCAUUCCACAUGACAACGAGGAAGAGUUUUAUGGAGAACAGUCCAUAGAACGCGGCAGAAACGACUAUGAACCGGGGGAAAGUGUAGAGGAGCAAUGUUGCGGACUGCCACAUCACCCAGAGCAACCACCACGAAUACAACCAAGAGCUGAGCAGGACGCGCACGAUUGUCUCUGGUCCACGUCUGCCAACCUUAUAACUUAGUGUGACAAGUGCUACUGUCAAUCCUACUGAGAGAGCCACUAGGAUAGGUAGCACGACAACACCAGCCAAAAAGUCAACAACAACUAUCUCAUACGAAAAGAUAGCUGGUUUGGCACCAAAGAGAAUGAUUGAAUGGUUGAAGUAUCUGUACAAGGCAGACACCCAAUCCGGGGUGAUCCAGGUGAUCGAGGGACUUGAAUCGAGUCCGAACAUCGCGAAGAUGUAUGCCAACCAAGAGGUUGACUGUUGAACGAUGAAUUCGUAACGAACGUUUGCUGUGUAUUCGUAGUCGUCGUGAAUCAUCGUGUGUUGGGGGGGUUGGAGGGUUUUUCCAUCUAUCAAGGAAAAUUACCCUGGCCUGAAAACUGGACGUGCCACACGCCAUGGUUGUGAAGCCAUACCGUGUGUACUAAAUCACCCCCUGGGCAGGGGGGCCCUCUUGAGGCAGAAGCUUACCUUGCUUGCGUCCACGACGCCUGGGUCCACGAAAACCCGUCCACACAUUGCUCUAAGCAAACGCGUCAAACCCCCGCCAGGGUGAGCACAAUGUGCGGAUAAGGAUGAGAUGAGAGUGGAGAUCAGUAUUUAUACACCAGUGAGAUCUCAACCCAGUGUGAUGGAAUUGCCUUUGUAAUUCAUGACCUACUCUUGCCGGAACUGGUCAGGGACUUUGUUUUGGUAGUGUGUUCUACCCCUCUUGUGGUACCCAUUGUCGAUCGGGUGCACGGUCAUCUAGCUUCUUGAUAUGGUGUGGCUAGCGAAUCCCAUGUGAGAGAAUCCCUAGGGCUUGCCUGACAGGCAGUUCCCCAAGAUCGGAAG  >Ss-AA_clean.1_(paired)_contig_195  AGCUCUUUUCGUACUACGAAGACUGAGCCACUUUUGGUUACCAGGCAAGAUUUGACUCUGCCUAAUCUCCAAAAGUCUCGGGAUCUUCGUAGGUUGAAAAGACCUACCCAGCUGCCACUUCCGGGUUGCCAUCCAACCCCCAAUAUCCUUCACGACUUCCUCGGUGACUUGUUCACGCGGAAAGGAUUGGAAUUCGUUACCUGCCAUCACUAUAUUGUGAGGACAUGGUGCCGGCUCCAAUGAAGGUAUAUUAGAAGCCCGAAGCGAGGCUUCUCUCGUCCAUAGAGCUCCCGAAAAGGCAUACUUCCAACAUGAUCUGGCGAGUCUCCCGAAAAAAUUCAUAUCCUGAGCAACGCAUCCCCACCUAAGUAGGUAUGCAUGCCACCGUAGAAAGCAGAUCCCUCGUGCGUACCA  >Ss-AA_clean.1_(paired)_contig_1976  CCAAUUCUCGUAUGGCAAUCCGUCCUGGUUAUCCACACCAGGCAGUGGUAUCUGUCUUACCCGCGAGUCAACGCGUAAUAAUCUCGUUAGGAGACGAACUUAAGUCAGAUUGGUGGCUUAUUAAAAAUCCUCGGCGAAGGCGACUGCAGCUAGAAACGGCAGAAGAAAACCUUAAACUAACUCAUGAUGGCCACAACAAAUAAGAAUAACGUACUUGCUGAACCCAUCAUUCAAGAAUUAGAGGAUCCGGCGACAAACUGUCGUCUUUACAAAGCCCGCACCAAUAAUCUGGUCAAGCGCGCAACACUAACGGUGGCGGAACUUCACGCCCAUAACAUAGAACCUUUUCUCCUGAAAGAUGGAGACUGCUUGAACGUUUACAAGCAGGUGAAGUCAUAUCUCUCACGA  >Ss-AA_clean.1_(paired)_contig_2128  AAAUAUCUUUCGACAGAUAAUUCUUGACUUGCUUAUAGGUAUCCAGACAGGUUCCAUCUUUUAUGAGAAAAGGUUCUAUCUUGAGGGCGUGAAGUUCCCCUACCGUUAGCGAUGCGCGCUUGACCAGAUCAUUGGUGCGGGCUUUGUAAAGACGACAGUUUGUCGCCGGUUCCUCUAACUCUGGAAUGGUGGGUUCAACAAAAACGUUAUCACUUUUAGUUGUAGCCAUCAUGAGUUAAUUCAAGGUUUCCUUCUGCCGUUUCUAGCUGCAGUCGCCUUCGCCGAGGAUUUUUUACAGACCCCAAUUCAGUCUGAGAACGU  >Ss-AA_clean.1_(paired)_contig_628  AAAUAUCUUUCGACAGAUAAUUCUUGACUUGCUUAUAGGUAUCCAGACAGGUUCCAUCUUUUAUGAGAAAAGGUUCUAUCUUGAGGGCGUGAAGUUCCCCUACCGUUAGCGAUGCGCGCUUGACCAGAUCAUUGGUGCGGGCUUUGUAAAGACGACAGUUUGUCGCCGGAUCCUCUAACUCUAGAAUGAUGGGUUUGACAUGCUUUUUCUUGUAUGUUGUAGCCAUCGUGAGUUAAUUUAAGGUUUUUUUCUUCUGCCGUUUCUAGCUGCAGUCGCCUUCGCCGAGGAUUUUUUACAGACCCCAUGUAGGUCUGAGAACGUCUCCUAACGUGAUUCUAACGCAUUUAGA  >Ss-AA_clean.1_(paired)_contig_872  GUCAGACGGAUUUUCAGAUCCUCAAGCAUAGACCCCUGCAUGCAUUCGCAUGCAUCAGGGAGCAAUUUCUUGAUCGACUGGAAGGCCAAUUGAACUUCAGGUAAGUCAGAGAUAUCUUGUGAAAGAUAUGACUUCACCUGCUUAUAAACGUUCAAACAGUCUCCAUCUCUCAGGAGAAAAGGUUCUAUCUUGAGGGCGUGUAAAUCCGCCACCGUUAGCGUUGCGCGCUUGACCAGAUCAUUGGUGCGGGCUUUGUAAAGACGACAAUUUGUCGCCGGAUCCUCUAACUCAGGAACGAUGGAUUCAAUUACGCGGUUAUGUGUAGUUGUGGCCAUCGUGA  >Ss-AA_clean.1_(paired)_contig_20  GGGGAAUUUUCUAUAUUUUUUUUUUUUUUUUUUUUUUUAAAAAAAAAAAGAAGAAUUUUUCUCAAUAAAUUUUUUUUUUCCCCCAUUUCCUUACCCCAUGAUCCGCCCUUAAGGGACCUUCUCGUCUUACAACGACCGUUUGCACGAUCAGACAAAACUAAGACAAUUCUCUCCGCUGUCAUGGUUGAAAAACCAGAUAAUGGACAUCCCUAUCAGUCA  >Ss-AA_clean.1_(paired)_contig_277  UAUCUGUCUUACCCGCGAGUCAACGCGUAAUAAUCUCGUUAGGAGACGAACUUAAGUCAGAUUGGUGGCUUAUAAAAAAUCCUCGGCGAAGGCGACUGCAGCUAGAAACGGCAGAAGAAAACCUUGAACUAACUCACGAUGGCCACAACAAAUAACAAUAACGUAUCUAUUGAAUCCAUCGCUCCAGAGUUAGAGGAUCCGGCGACGAACUGUCGUCUUUACAAAGCCCGCACCAAUGAUCUGGUCAAGCGCGCAACGCUAACGGUGGCGGAACUUCACGCCCUUCACAUAGAACCUUUUCU  >FirsU_Contig126  AAAUAUCUUUCGACAGAUAAUUCUUGACUUGCUUAUAGGUAUCCAGACAGGUUCCAUCUUNUUAUGAGAAAAGGUUCUAUCUUGAGGGCGUGAAGUUCCCCUACCGUUAGCGAUGCGCGCUNUGACCAGAUCAUUGGUGCGGGCUUUGUAAAGACGACAAUUCGUCGCCGGAUCCUCUAACUNCUGGAACGAUGAAUUCAAAAUGCUUUUUAUCGUAUGUUGUGGCCAUCGUGAGUUAAUUCANAGGUUUUCUUCUGCCGUUUCUAGCUGCAGUCGCCUUCGCCGAGGAUUUUUUACAGACCCCNAUGUCAGUCUGAGAACGUCUCCUAACGUGAUUCUAACGCAUUUACUUGCGGGUGGAGUCANGACCACCACUGCCUGGUGUGGAUAACCAGGACGGAUUGCCAUACGAGACAUGGCCGUAUGNCUGUUAACGAN  >Ss-AA_clean.1_(paired)_contig_278  CCAAUUCUCGUAUGGCAAUCCGUCCUGGUUAUCCACACCAGGCAGUGGUAUCUGUCUUACCCGCGAGUCAACGCGUAAUAAUCUCGUUAGGAGACGAACUUAAGUCAGAUUGGUGGCUUAUUAAAAAUCCUCGGCGAAGGCGACUGCAGCUAGAAACGGCAGAAGAAACCUUGAAUUAACUCACGAUGGCCACAACAAAUAGUGAUAACGUAUUUGUUGAAUCCAUCGUUCCAGAGUUAGAGGAUCCGGCGACGAACUGUCGUCUUUACAAAGCCCGCACCAAUGAUCUGGUCAAGCGCGCAACGCUAACGGUGGCGGAACUCCACGCCCUUCACAUAGAACCUUUUCUCCUGAAAGAUGGNNNNNNNNNNNNNNNNCACGAGAUAUCUCUGACUUACCUGAAGUUCAAUUAGCCUUCCAGUCGAUCAAGAAAUUGCUCCCUGAUGCAUGUGAGUGCAUGCAAGGAUCUAUGUUGGAGGAUCUGAAGACCCGUCUGACUCGCCCCGCUCCUACCCUUCCACCGGGUUAUGUAGCCUUUUGUCGAAAGAUUGUUAAAGAACUCUUUCCAAAUGGCUGGGAUCAUAACUGGAAGUCGGCAGUGGCAAACUUCUCUCCCUCUCUCGGCUCUUGUGUAAAUUAUUCUCGUAAGUCGGGCGGCCAAUUGGCAGCCCUCGCUACUGAAGGACAAUCUGCAUGGCAUGAUGGUUUGGACAAACCUCAUGGUGCCUUGAAAGGGGAGCUUCUCCUGGUAAACUCGUCGGGGAAA  >Ss-AA_clean.1_(paired)_contig_40  AGUAGCGAGGGCUGCCAACUGGCCGCCCGACUUACGUGAAUAAUCAACACAAGAGCCGAGAGAGGGGGAGAAAUUUGCUGCUGCCGUCUUCCAGUUAUGAUCCCAGCCGUUUGGAAAGAGUUCUUUGACAAUCUUUCGACAAAAGGCGACAUAACCCGAUGGAAGGGUAGGAGCGGGGCGAGUCAGACGGAUUUUCAGAUCCUCAAGCAUAGACCCCUGCAUGCAUUCGCAUGCAUCAGGGAGCAAUUUCUUGAUCGACUGGAAAGCCAGCUGUACCUCCGGCACGUCAGAAAUAUCUUUCGACAGAUAAUUCUUGACUUGCUUAUAGGUAUCCAGACAGGUUCCAUCUUUCAGGAGAAAAGGUUCUAUCUUGAGGGCGUGAAGUUCCCCUACCGUUAGCGAUGCGCGCUUGACCAGAUCAUUGGUGCGGGCCUUGUAAAGACGACAGU  >FirsU_Contig56  NUNUAGUCUCGUAAGGCGGGUGGCCAGUCGGCUGCUCUCGCGACUGAAGGACAAUCGGCAUGGNCAUGAUGGUUUAAAUAAACCUCAUGGUGCCUUGAAAGGGGAGCUUCUCUUGGUAAACUCGNUCGGGUAAACCUCGACCUCUUACGAGGUUUGAGGCUGACUCGUCUUACCUUCGUCCAUUGNCUCGGUCUGAUUUACGAUCAGAUCUCGAAGAACCCCUGGUUGCUUCGUGGCGAUGUAACGNGCUGAGAAGUUGAAGAAUGCNCGGCUUUUCUGGGAGUGGCGAGACUUCUCUCAUCUCCGGGNGACUACGUCUCCGCAUCUGANCAAUCUCCCUAUCGAGAUUGCAGAACUUAUUCUUGACGUGGCUUGGUCGAACUCGAACCANAGUCCCAGCGUCGGUGUUUCGGUAUGCAUUAGCUGCACAGAGACCCGAGCUGACGUUCAGNACGUCAAGAUCACACCAUUGAUUCAUUUNNUACCUAGCUUUCCGCUGGGCGACGAAGGAUNAUGGCAACGGUGCCUCCGGCAUUGAUCAAUGGUGAUGACAUCCUUGUCGAAAAGGAUGAUAAAGUUCUACAAUCUUUGGUCAGGAACGAUCAAAGAUGUCGGAUUCGAAGUCGAGCAGUCCAAAGACAUCCGUGUCUCUCGAAUGGGGUACUAUUAAUUCCACGCUUCUUCGCAAGAAGGGGGNGAAAUUUAGUUCCUGUUCAGACAACGAGGAUGGGACUGAUCCGCCUAUCUAGGAAUCCAGNCUAACCUGGGCGCCUCCUUUGAAAAAUUCGCCCGCGUCGGCGAUCCUGCUCGAUGGUAN  >Ss-AA_clean.1_(paired)_contig_4328  CGGGCGGCCAAUUGGCAGCCCUAGCUACUGAAGGACAAUCAGCAUGGCAUGAUGGUAUGAAUAAACCUCAUGGUGCCUUGAAAGGGGAACUUCUCUUGGUAAACUCGUCGGGAAAACUUCGACCUCUUACGAGGUUUGAGGCUGACUCGUCUUACCUUCGGCCAUUGCACUGUCUGACUUACGAUCAGUUCUCGAAGAACCCUUGGUUGC  >Ss-AA_clean.1_(paired)_contig_131  CUGGGUUUCCCCGAUGAGUUUACCAAGAGAAGUUCCCCUUUCAAGGCACCAUGAGGUUUAUUGAUACCAUCAUGCCAUGCCGAUUGUCCUUCAGUCGCGAGAGCAGCCAACUGGCCACCCGACUUACGAGAAUAAUCGACNNNNNNNCCGAGAGAGGGGGAGAAAUUUGCCGCUGCCGUCUUCCAGUUAUGAUCCCAGCCAUUUGGAAAGAGUUCUUUGACAAUCUUUCGGCAAAAGGCGACAUAACCCGAUGGAAGGGUAGGAGCGGGGCGAGUCAGACGGAUUUUCAGAUCCUCAAG  C>Ss-AA_clean.1_(paired)_contig_937  GAAGACCCGUCUGACUCGCCCUGCUCCUACCCUUCCAUCGGGUUAUGUAGCCUUUUGUCGAAAGAUUGUUAAAGAACUCUUUCCAAAUGGCUGGGAUCAUAACUGGAAGACGGCAGCUGCGAAUUUCUCCCCCUCUCUCGGCUCUUGCGUCGAUUAUUCUCGUAAGUCGGGUGGCCAGUUGGCUGCUCUCGCGACUGAAGGACAAUCGGCAUGGCAUGAUGGUUUGAAUAAACCUCAUGGCGCCUUGAAAGGGGAACUUCUNNNNNNNNNNNNNNNNNNNNNNNNNNNNNNNNNNNNNNNNNNNNNNNNNNNNNNNNNNNNNNNNCGUCCAUUGCACGGUUUGAUUUAUGAUCAAAUCUCGAAGAACCCUUGGUUGCUUCGUGGCGAUGUUACGGCUGAGAAGUUGAAAUCCGCUGGCUUUUCUGGGAGUAGCGAGACUUCUCUCAUCUCCGGGGACUACGUCUCCGCUUCUGACAAUCUACCUAUUGAGAUUGCAGAACUUAUUCUUGACGUGGCAUGGUCGAGUUCGUACCAUGUCCCAGCAUCAG  >Ss-AA_clean.1_(paired)_contig_783  GUGUAGAUUAUUCUCGUAAGUCGGGCGGCCAAUUGGCAGCCCUCGCUACUGAAGGACAAUCUGCAUGGCAUGAUGGUUUGAAUAAACCUCAUGGUGCCUUGAAAGGGGAACUUCUCUUGGUAAAUUCGUCGGGGAAACCCAGACCUCUUACGAGGUUUGAGUGUGACUCGUCUUACCUCCGUCCAUUGCACGGUCUGAUUUAUGANNNNNNNNNNNNNNNNNNNNNNNNNNNNNNNNNNNNNNNNNNNNNNNNGAAGUUGAAGAAUGCCGGUUUCUCUGGGAGUGACGAGACUUCUCUCAUCUCCGGGGACUACGUCUCCGCAUCUGACAAUCUUCCUAUUGAGAUUGCAGAACUUAUUCUUGACGUGGCUUGGUCGAGUUCGAACCAAGUCCCAGCAUCGGUGUUUCGGUAUGCUUUAGCUGCACAGAGACCCGAGCUGACGUUUGAGCGUCA  >FirsU_Contig39  GUAGCCUUUUGUCGAAAGAUUGUUAAAGAACUCUUUCCAAAUGGCUGGGAUCAUAACUGGNAAGACGGCAGCUGCGAAUUUCUCCCCCUCUCUCGGCUCUUGUGUCGAUUAUUCUCGUAAGNUCGGGUGGCCAGUUGGCUGCUCUCGCGACUGAAGGACAAUCGGCAUGGCAUGAUGGUUUGNAAUAAACCUCAUGGCACCUUGAAAGGGGAACUUCUCUUGGUAAACUCGUCAGGAAAACCUNCGACCUUUUACGAGGUUUGAGGCUGACUCGUCUUACCUUCGUCCAUUGCACGGUUUGAUCNUAUGAUCAGAUCUCGAAGAACCCUUGGUUGCUUCGUGGCGAUGUAACGGCCGAGAAGUUGNAAGGAUGCCGGCUUUUCUGGGAGUGGCGAGACUUCUCUCAUCUCCGGGGACUACGUCUCCNGCAUCUGACAAUCUCCCUAUCGAGAUUGCAGAACUUAUUCUUGACGUGGCUUGGUCGANGUUCGAACCAAGUCCCAGCGUCGGUGUUUCGGUAUGCAUUAGCUGCACAGAGACCCGAGCNUGACGUUUGAGCGUCAAGAUCACACUAUUGAUUCAUUUGUCCCGAGCAUCGGUCAAAUGANUGGGAAGCUAUUUAUGCUUUCCAUUGCUCUGUAUCCAAAAUUACCUAGCUUUCCGCUN  >Ss-AA_clean.1_(paired)_contig_3619  CAGAAGGAUACUGCUGAUGGGUAUUAAAGCGAGAUUUUUCAAAAAAAGUGUCUUUGAGAAUGAUAACCAGAGUAAUAGAGGAUGUGUAGAAGUUGUUUCGGAAAUCGUGAGGUUCGAGAUAGACGAUGCUUAAGUCUAGGAGGUGCAAGGAAUAGAAAUUGAUGUGAUGUAUCCUCUGCCGGGCCUAGACUUUGCGUCUCCAUGAGGUCCUCUGAUGGUCGUAGGCCAUUUCGUGGACGAUCGUGAGUGAGGUGAUGUUCGUGAAGGCCAUUAUCUGGACUUUGGGGCAGGGUGGUUUGGUGCUGGGUGGUUUGGUGGAGGUAAUCCAGUAGUGUCAUAUGCGCCACGGCCCAUGUUGGAAGAUGCGUUCUCCUUGAAAAUCAUGAUGACUGUGUGUAUUUGAAGAGUUGCUUUUUUGGCUUGCGUUCACAAGGUUUCCUCCUUUAACGGGAAAGGGAGGGUAACUGGUGUGGAAGAGCUUCUUUGGUAAGCUAUUAGCUACAAGCUAUUUCCAGGGGUAAAUCGCCGAGGAUGAAUCGAUGUGGAUAUGGAUAUGGGUGUGGAUGUGGAUGGCAGGCCUAAUGGCUAAUUGUCGUAUCGUAAAUAGACUUUUCGGUUUCCACAGAUGACCUGAAAUGAUCUGAUUGAAUGAAAGUUUGAAGAACUGAAUUGAACUGAAGUGAAUUGAAUUGAAUGAUUGAUUGAAUGAUUGACUGAAGUGAAGAUGGGCGAAUCGCCUGGUAUUAUUUGAAAACUGUUCUGGUGAUGUUUCUAUUUAAGAUUUCGUGUUGCUUUGACUAUCUGAGAACUUUGCGACAAAGUCUGCGACGGUGCGAGCAGGUCAGACGCUGGAGCGUUGAACCGGAUAGCUGUGACUUUAAUGCACAGGGUUCGUGUGAAAGUACCUAGCCGAUAGGGAUGCUGCGUGAACUGCUGCCGCCGGAUAUCAACCUCUUGACGCCGAUGGAAGUAAUGCCAAUGGUUGUGAGAAGAUGGUUUGAGAGUAGGAGGAGGUAGUUUGUUUGUAUAGUCGGUAGAGUGUACACUGCCAAAAGUGGAAAGGACGAUGAAUGAUCAAGACGCAAGAUGAAAGGACGAGAUACUUGAGGAGGAUGAUGAUGAUGAUGGAGGAAUUCACAA  >Ss-AA_clean.1_(paired)_contig_1492  ACUUCUCCCCCUCUCUCGGUUCCUGUGUAGAUUAUUCACGUAAGUCGGGCGGCCAGUUGGCAGCCCUCGCUACUGAAGGACAAUCUGCAUGGCAUGAUGGUUUGGAUAAACCUCAUGGUACUUUGAGAGGGGAACUUCUCUUGGUAAACUCGUCGGGGAAACCCAGGCCUCUUACAAGGUUUGAGUGUGACUCGUCUUACCUUCGUCCAUUGCACGGUUUGAUCUAUGAUCAAAUCUCGAAGAACCCUUGGUUGCUUCGUGGUGAUGUAACGGCCGAGAAGUUGAAGAAUGCCGGCUUUUCUGGGAGUGGCGAGACUUCUCUCAUCUCCGGGGACUACGUCUCCGCAUCUGACAAUCUCUCUAUCAAGAUUGCAGAACUUAUUCUUGACGUGGCUUGGUCGAGUUCGAACCAAGUCCCAGCAUCGGUGUUUCGGUAUGCUUUA  >Ss-AA_clean.1_(paired)_contig_7164  AUAAACCUCAUGGUGCCUUGAAAGGGGAACUUCUCUUGGUAAACUCGUCGGGAAAACCUCGACCUCUUACGAGGUUUGAGGCUGACUCGUCUUACCUUCGUCCAUUGCACGGUCUGAUUUAUGAUCAGAUCUCGAAGAACCCCUGGUUGCGUCGUGGUGAUGUGACAGAUGAAAAGUUGAAGAAGGCCGGCUUUUCUGGGAGUGGCGAGACUUCUCUCAUCUCCGGGGACUACGUCUCCGCAUCUGACAAUGUCCCUAUCGAGAUUGCAGAACUUAUCCUUGACGUGGCUUGGUCGAGUUCGUACCAAGUCCCAGCAUCGGUGUUUCGGUAUGCAUUAGCUGCACAGAGACCCGAGCUGACGUUCAGACGUCAAGAUCACACUAUUGAUUCAUUUGUC  >Ss-AA_clean.1_(paired)_contig_758  CCCAGGUUAGCUGGAUGCCUAGAUCGGCGGAGCAGUCCCAUCCUCGUUGUCUGAACAGGAACUAGAUUCCCCCCCUUCUUACGAAGAAGCGUGGAAUUAAUAGUACCCCAUUCAAGAGACACGGAUGUCUUGGACUGCUCGACUUCGAAUCCGACAUCUUUGAUCGUCCUCGACCAAAGAUUGUAAAACUUAUCAUCCUUCUCGACAAGGAUGUCAUCACCAUUGAUUAAUGCCGGAGGCACCGUUGCCAUAUCCUUCAUCGCCCAGCGGAAUGCUAGGUAAUUCUGGAUACAGAGCAAUGGAAAGUAAAGAUAGCUUCCCAUCAUUUGACCGAUACUCGGAACAAAUGAAUCAAUGGUGUGAUCU  >Ss-AA_clean.1_(paired)_contig_359  CCAUCCUCGUUGUCUGAACAGGAACCAAAUUCCCUCCCUUCCUACGAAGGAGCGUGGAAUUAAUGGUACCCCAUUCUCGAGACACGGAUGUCUUGGUCUUCUCGACUACGAACCCCACUUCUGCGAUCGUCCCCGACCAAGAAGUGUAGAACUUAUCAUCCUUUUCGACAAGGAUGUCAUCACCAUUGAUCAAUGCCGGAGGUACCGUUUCUUGAUCCUUAACUGCCCAGCGGAAAGCUAGGUAGUUCUGAAUACAAAGAAGCGGAAAGCAAAGAUAGCUUCCCAUCAUUUGACCGAUACUCGGGACAAAUGAAUCAAUGGUGUGAUCUUGACGUUCAAACGUCCGCUCAGGUCUCUGUGCAGCUA  >FirsU_Contig21  NUUCGUAGGUUGAAAAGACCUACNCCAGCUGCCACUUCCGGGUUGCCAUCCAACCCCCAAUAUCCUUCACGACUUCCGCAGUGANCUUGCUCACGUGGAAAGGAUUGGAACUCAUUACCUGCCAUCACUAUAUUGUGAGGACAAGNGUGCCGGUUCCAAUGAAGGANAUAUUAGAAGCCCGAAGCGAGGCUUCUCUCAUCCAUAGAGNCUCCCGAAAAGGCAUACUUCNCAACAUGACCUGGCGAGUCUCCCAAAAAAAUUCAUAUCCUNGAGCAACGCAUCCCCACCUANAGUAGGUAUGCAUGCCACCGCAGAAAGCAGAUCCCUCGUGNCGUACCAACGAGACGGAUCGNCCAACACGGGCGAAUUUUUCAAAGGAGGCCCCCAGAUUAGNCUGGAUGCCUAGAUCGGCGGNAGAAGACCCAUCCUCGUUGUCUGAACAGGAACUAGAUUCCCUCCCUUCCUACGAAGGAGANGUGGAAUUAAUAGUACCCCAUUCUCGAGACACGGAUGUCUUGGUCUUCUCGACUACGAACNCCCACUUCAGCAAUCGUCCCCGACCAAGAAGUGUAGAACUUAUCAUCCUUUUCGACAAGGNAUGUCAUCGCCAUUGAUCAAUGCCGGAGGUACCGUUUCUUGAUCCCUAACUGCCCAGCGGNAAAGCUAGGUAGUUUUGGAUACAAAGAAGCGGAAAGCAAAGAUAGCUCCCCAUCAUUUGANCCGAUACUCGGGACAAAUGAAUCAAUGGUGUGAUCUUGACGUUCAAACGUCCGCUCAGGUNCUCUGUGCAGCUAN  >Ss-AA_clean.1_(paired)_contig_6355  GAGUAUCGGUCAAAUGAUGGGAUGCUAUCUUUGCUUUCCGCUUCUUUGUAUUCAUAACUACCUGGCUUUCCGCUGGGCAGUUAAGGAUCAAGAAAGGGUACCUCCGGCAUUGAUCAAUGGUGAUGACAUCCUUGUCGAAAAGGAUGAUAAAUUCUACACUUCUUGGUCAGGGACGAUUGCAGAAGUGGGGUUUGUUGUCGAGAAGACCAAGACUUCCGUGUCUCGAGAAUGGGGUACCAUUAAUUCCACG  >Ss-AA_clean.1_(paired)_contig_630  UUGAUCAAUGCUGAUGACAUCCUUGUCGAAAAGGAUGAUAAAUUCUACACUUCUUUGUCAGGGACGAUUGCAGAUGUGAGGUUCGAAGUCGAGAAGUCCAAGACAUCCGUGUCUCUUGAAUGGGGUACUAUUAAUUCCACUCUUCUUCGUAAGAAGGGGGGGAAUCUAGUUCCUGUUCAGACAACGAGGAUGGGACUGCUCCGCCGAUCUAGGCAUCCAGCUAACCUGGGCGCCUCCUUUGAAAAAUUCGCUCGCGUUGGCGAUCCUGCUCGAUGGUACCAUCGAGGGAUUUGCCUUUUGCGGUGGCAUGCAUCCAUCCUCAGGUGGGGAUGCGUUGCUCAAGAUAU  >Ss-AA_clean.1_(paired)_contig_418  AGUGACCUGUUCACGUGGAAAGGAUUGGAAUUCGUUACCUGCCAUCACUAUAUUGUGAGGACAAGGUGCCGGUUCCAAUGAAGGAAUAUUAGAAGCCCGAAGCGAGGCUUCUCUCAUCCACAGAGCUCCCGAAAAGGCAUGCUUCCAACAUGACCUGGCGAGUCUCCCAAAAAAAUUCAUAUCCUGAGCAACGCAUCCCCACCUAAGUAGGUAUGCAUGCCACCGUAGAAAGCAGAUCCCUCGUGCGUACCAACGAGACGAAUCGCCAACACGGGCGAAUUUUUCAAAGGAGGCUCCCAGAUUAGCUGGAUGCCUA  >Ss-AA_clean.1_(paired)_contig_1160  CUUCGUAGUACGAAAAGAGCUGCUAAGAUCGCGAUUUGGAGGUUCUUUCCAAGAGAAUCACUAGGUCGUAAGACUACCCAGUUGUUUUCCCUAUCUGGUCAAAUGCGAUGCGGUUUUGGUCGGUCUUAUUGGACCCGACCCGAGAGGUGUACCGAUGUUCGGGUACCAGUCGAAUUAUGUUCUAAGCAUAAUCUGGUCCCACAACACGUCUCGGUGGACGUUGUCGUUAGUCAGCGUGAUAAUUCCCUGUUCGGUAAGGGUUUUAGCAUGCUCAUGAACUCCUCGGCUCGAGAGGAGAGGAUGAGGCGAAUUGUCGCUGACUACGACGCUUUGUAAUGAC  >Ss-AA_clean.1_(paired)_contig_150  AGAGAAUCACUAGGUCGUAAGACUACCCAGUUGUUUUCCCUAUCUGGUCAAAUGCGAUGCGGUUUUGGUCGGUCCCAUUGGACCCGACCCGAGAGGUGUACCGAUGUUCGGGUACCAGUCGAGUUAUGUCAUAAACAUAACCUGGACCCACAACACGUCUCGGUGGACGUUGUCGUUAGUCGGCAUGAUGUUUCCCUGUUCGGUAAGGGUUACAGCAUGCUCAUGAACUCCUCACUUCGAGAGGAGAGGAUGAGGCGAAUUGUCGCCGACUACGACGCUUUGUAAUGACGGCUUGUUCGUCGCCGUCGGAGGGGGGGGAAAAUAGAGAGGGCGCUCGUAUCUAGUAACGGGAUACUCGGCCUGGGGUGGUCAUGGAGAUGGACACCGGUCAACGGUAGUAUUCUUACCCAAAGAUGGGGAAGUAACGUUCUUGACUG  >Ss-AA_clean.1_(paired)_contig_94  GAGCUUCAAUUGCCCAUCUAUAUCACCUCAACUUCCUCACCUCAAUCACUAUUACAUUUCCCUCAUCCUUACCGCGUGAUUCGCCCUUAAGGGACCUUCUCGUCUUACAACGACUGUUUUCACAGUUAGGCAUCCUUAAGACAACACUCUCCGCCGUCAUGGUUAAAAAACCAGAUAUUAGACAUCCCUAUCAGUCAAGAACGUUACUUCCCCAUCUUUGGGUAAGAAUACUACCGUUGACCGGUGUCCAUCUCCAUGACCACCCCAGGCCGGGUAUCCUGUUACUAGAUACGAGCGCCCUCCUUAUCUUUCCCCCCCUCCGACGGCGACGAACAAAGCCGUCAUUACAAAGCGUCAUAGUCGGCGACAAUUCGCCUCAUCCUCUCCUCUCGAAAUGAGGAGUUCAUGAGCAUGCUGUAACCCUUACCGAACAGGGAAACAUCAUGCCGACUAAUGACAA  >Ss-AA_clean.1_(paired)_contig_149  AUCCGCCCUUAAGGGACCUUCUCGUCUUACAACGACCGUUUUCACAGUCAGACAAAAUUAAGACACUUCUCUCCGUCGUCAUGGUUGAAAAACCAAUAUUGGACAUCCCCAUCAGUCAAGAACGUUACUUCCCCAUCUUUGGGUAAGAGUACUACCAUUAACCGGUGUCCAUCUCCAUGACCACCCCAGGCCAAGUAUCCCGUUACUAGAUACGAGCGCCCUCUUUAUCUCCCCCCCCUCCGACGGCGACGAACAAAGCCGUCAUUACAAAGCGUCAUAGUCGGCGACAAUUCGCCUCAUCCUCUCCUCUCGAUAUGAGGAGUUCAUGAGCAUGCUAUAACCCUUACCGAACAGGGAAAUAUCAUGCCGACUAAUGACAACGUCCACCGAGGCGUGU  >Ss-AA_clean.1_(paired)_contig_280  ACGUUACUUCCCCAUCUUUGGGUAAGAGUACUACCGUUGACCGGUGUCCAUCUCCAUGACCACCUCAGGCCGAGUAUCCCGUUACUAGAUACGAGCGCCCUCUUUAUCUCCCCCCCCUCCGACGGCGACGAACAAGCCGUCAUUACAAAGCGUCUAACUUACUUGACAAAAUACUUCAGGAGUCCCGAAUUACGACUGCGUGUGGGUCACGCCCCCAGUCCGAAGACCGUGUCUGCCACCUUAAGUGGUGUAUCUCCAAGUAUUGAUUGUAUAAGUUAGAUAACUUGUCCAUCAAAGGUAGAAACAAUUCGCCUCAUCCUUUCCUCNNGAGGAAUUCAUGAGCAUGCCAAAACCCUUACCGAAAAGGGAAUUGUCUCUUCUACCAAUAAUAACAUCCACCGAGCAGUGUUGUGGGAUCAAAUGGUGCUUCACGCACAAGUCAUUUGGUACACGCACAUCGGUACACUUCUCGGGUCGGGU  CCAAUAAGACCGACCAAUACCGCAACGCAUUUGACCAAGCGGGGAAUACAACUGGCAGAACUUACAACCCAGGGAAUUCUUCGGGAAGA  >FirsU_Contig12  NGNGAACCAGCACCUUGUCCUCACAAUAUUGUGAUGAAAGGUAAUGAGUUCCAAUCCUUCCCGNCGUGAGUUGGUCACCGCGGAAGUCGUGAAGGAUAUUGGGAGUUGGAUGGCAACUCGGAAGGUGGCAGCUGGGUAAGUCUUUUCAACCUACGAAGACUUCAAGGCUUUUGGAAAUUAGACAGGAGUCAAAUCUUGCCUGGUGACCGAAAGUGGCUUGAGCUUCGCAGUACGAAAAGAGCUGCAUAAAAACGCGAUUUGGAGGUUCUUUCCAAAAGAAUCACUAGGUCGUAUGACUACUCAGCUGNUUUUCCCCGUCUGCUCAAACGCGUUGCGGAUUAGGCCGGUCCUGGUGGACCCGACCCGAG  >FirsU_Contig30  AGCUACCCGCUAGAGGUAGGGGACUAACCACGCUUUCGCGUUGACUCCGGGAUCCAUGCCNGCCGGUCUGAAACUUCUUCAGAGAGUCACUACAAGGUUCUUUCGCUGAUCCUCUUAAAAGNCGACGACUCUUACUCAUUCCAUAGUGAUACGGUACUAGUCUACUUCAGGAAGGGUUCAAANUGUGACCCAACCAUCCGUUCUUUAUAUCCUAUUUCCUAAGUUCAUUCCUCAUGGGUGCGGNCACCUAUUAAGUCCCCUUUUUCCGGCCUCUAAUCGAGGUACUCGGGAGAAGGUUCACGAGNCUGAGCUCAGACCGCCGUUAACCACCCAUUCAUGAUGCUUAAGUCCUUAGAGACAUAACCNAUUAACAAUCACUACUUCCUUUUCAUUUGUCAUUAAACUUCUAAGCAUCCCUUUGAUGCANACUCCAUUGUCCCAACACAGCCUUUAUCUUCACUCCGUGUAUACCAGACCCCAAUGGGGGNAGUAGAUGCGAAGGGGGCGAAAAGUUGUACAUUCUUUUAUUUAAUAUCUCAACACGGCCANACGGGCGAGCGUGUCAAGAAGGGAUGGUCAGUCUUCCACUUUGUGUUAGAAUGAUCAAGGNACCGAAGGGAAGCGUUCACUCACGAGAGUGUAUACCUCCUUAGGCCACAAGAACUUCCACNUGGAAUCCCUUCCACGUGUACAGCCUCUCCGCCAGAGAGAAUUUUUCCUUAGAACGAUCUNUUAUAUGCUUGUCUAUUAAUGCGAACAGAUAGAGGCUUCACUCUUUUGCCUAUCCACAUUNUCAGUGUUAUCACGUUGGGCUUCUGCAGCCAUGCUUGUGACAAUUGCGUAGAACCGCUCUNUUGGGAGGAGGCCCAGGGCACGUCUCCUGAGACCUGUCUUGACCAGUUUUGAUUUCUUCANGCCUUGCCCACCGCAAGCGCCUGACGAAACCACUUCUUCCGAACGAGCGUCGCACGCCAANUAUGGGCCAAUCUCGGCUAUAUCCUCAAGCACUCCUCGUAGACUCACCUCAUGCCGAAGCNAUUUGGAUCACCUGGAAUACAUGGGAGGUACUAAAGCCUUUCAUGCCCACAAUGAUGGCGNCGCAACAAAGCGCCUGGUUCGUUUCUAUUGGGUCGAAGAAAUCCGAGCGUCGCCUUGGCGNACUAAACUCCUCUUCUUGGUAUCGUAUGACUGACUAUUGAGGUCCAGCCAUCGAGAAGAGNAUCUCGGUCUUACUUUCAUUAACUUCGAGUCCAUACUUACCGGUCACACGACGCCACUCCNUGGUAUAGAGCGGCGGUGCCGGCAUACAUUAUGUCGUCUCCGUUAAUCUUCACCCUCUUCNACUUUCCCGCGGCCUGGCCCGUAGAGAUUAUCAACUGCAAUCUCAAACGACGCCUUGUUCNAAAAGACAAAGUAUCGGGAAGUUCACCAAACUUCCCAUGGGGGAACCACGUAGCAUAGNGACCGGAUUCCAUUCGUUCAAACCACCGCAUGUCUGUGAAGGACUCAACAAACACUUCCCNUUUCGUACUCCGUAAGCUCAGAGGUCCUGGAGAUCUCGCGUACAAUGACUUCUACGGCUUNCCCUGUAUAUUUUAUUUGUCGCGGCAGUAUAAUCGCCGCUUAUUAACAGUUCACCUUCUUNGUCUAUCAUUGGCUAUCUCCUCGAAGUCCUCUUUUAAUACAUCCCCCCGAACACACCACUNUUUUGUCGCUGAUGUGUCGGUACAAGGCAUUAUGCAGGGGAGUCAACACUCUUUUGACCUNCCGCACUUUGCAUAGUCACAACCCUAAACUUUCCCUUUGUUUUUGCACAUCCUAGUCGUANCCACGUUCCUUUGACAGAACGGACCGUUUCUUGGGACUGCUAAGGUUCCGCCUAACAUUGNUGCUACACUCUGCACACCCUUGAACCCCCGGGACGUAAUCGCCCAAAACUGGUUCCUCUUNCACUCUUUCUGGCAGAAACUAAUCUACCACCCCAACCCCGGAUCAAAUGACGAACCUUCUNGUGCUAGCUGUUCUAGCUCGAAGAUCGUAGGACGGUCACUGGGCGGUGUAGGAUUCCCCANCACUGGCAACCCAUGCCUUCUUCUCCUCACNUACUUUUCCUUUUGUCACAUUUCUUACAUGNGUACAUCGAAAAGUCUGUUGACGGACUUAANCCAUCAUCGCCAGACGUCGACCACGUCUCGNAAUUGCGCUGGAAGCAGUUCAUCACACCGGNUGUUCCACUCACACCACCAGGCUCUAAUGCNUGGAACAGUUCGCUUCCCGGUCGACCGGGAAGACGACAUCAUGAGGAAUCUUAUAUUCCANUACGCAUGAUGCGAACUGCUCUCAGAAGAGCUAUUUUCAGGGACCCUGCUGCAGGACAGCNGGGUACAGGUACCUACCUUUCGGUGCUUCCCUGAAUUUCCAAUAGACAUGACAGACGUUANUCACGCGAUCUGCUUGCUCGGAAAUAGUCUCUUAAGGAGAUCUGN  >Ss-AA_clean.1_(paired)_contig_1694  ACUUCAGGGAGGGUUCAAAUAUGACCCGACCAUCCAUUCUUUAUAUCCUAUUUCCUAAGUUCAUUCCUUGCGGGUGCGGCACCUAUUAAGUCCCCUUUUUCCGGCCUCUAAUCGAGGUACUCGGGAGAAGGUUCACGGGCUGAGCCCAGACCGCCGUUAACCACCCACUCAUGAUGCUUAAGUCCUUAGAGACAUAACCAUUAACAAUCACUACUUCCUUUUCAUUUGUCGUUAAACUUUCAAGCAUCCCUUUGAUGCAACUCCUUUGUCCCACCACAGCCUUUAUNNNNNNNNNNNNNNCCGACCGGCGCGCGUGUCAAGAAGGGAUGGUCAGUCUUCCACUUUGUGUUAGAAUGAUCAAGGACGGACGGGAAGCGUUCACUCACGAGAGUGUAUACUUCCUUGGGCCACAAGAACUUCCACUGGAAUCCCUUCCACGUGUACAGCCUCUCCGCCAGAGAGAAUUUUUCCUUAGAACGAUCCUUAUACGCUUGUCGAUUAAUGCGAACAGAUAGAGGCUUCACUCUUUUGCCUAUCCACAUUUCAGUGUUAUCACGUUGGGCUUCUGCGGCCAUGCUUGUGACAAUUGCGUAGAACCGUUCCUUGGGAGGAGGUCCAGGGCACGUCUCCUGAGACC  >Ss-AA_clean.1_(paired)_contig_4006  CCUUCUUGUCUGUCAUUCGCUAUCUCCUCGAAGUCCUCCUUCAAUACAUCCCCCCGUACACACCACUUUUUGUCGCUGAUGUGUCGGUACAAGGCAUUAUGCAGGGGAGUCAACACUCUUUUGACCUCCGCACUUUGCAUAGUUACAACCCUGAAUUUCCCCUUUGUUUUUGCACAUCCCAGUCGUACCACGUUCCUUUGACAGAACGGGCCGUUUCUAGGGACCGCCAAGGUUCCACCCAACAUUGUGCUACACUCUGCACACCCUUGAACCCCCGGGACGUAAUCGCCCAAAACUGGUUCCUCUUCAUUCUCUCUGGCAGAAACUAAUCUACCACCCCAACCCCGGAUCAGAUGACG  >Ss-AA_clean.1_(paired)_contig_5114  AUGAACUGCUUCCAGCGCAAUUCGAGACGUGGUCGACGUCUGGCGAUGAUGGUUAAGUCCGUCAACAGACUCUUCGAUGUACCAUGUAAGAAAUGUGACAAAAUAAGAAGUAGGGAGGAAAAGAAGGCAUGGGUUGCCAGUGUGGGGAAGCCUACACCGCCCAGUGACCGUCCUACGAUCUUCGAGCUAGAACAACUAGCACAGAAGGUCCGUCAUCUGAUCCGGGGUUGGG  >FirsU_Contig88  NUCCAACGUUUUCUAGAUACAUCCACUANAACGCAGAAUGUAUCUGAAAGUGAUUUUUGGAANGUGCAUACAUCCCAAUUGAUCGUAAUGNAUAUUAAUGAACUUCUUGGAGUAGCAGUUUUCGNCUCUAUCUCAACAGGAGUCCACUUUGGNAACAAUACGCAGGAGGAACUCUUAGUGUUCAACNACAGAGAAAAAUUGAUCGCUCUUCUCANACAUCCGAGAGGCUGCUGAGGAAACUGUCCCCGAGGCUCCAGCCCAAUAAGCUCUAUUCUNCAUCUCAUAUGCGCAAUUCCUAAAUGAAAGAUCUCUUGAAUUAUUUAAUAAAACUUAGGANGUUUAACAUAAAUCCUUAUCAGCGAUAUGUCUACUCUANNNNNNNAUCUUGUGUAAACUGNUCUAGUCCGACUGAUGUUGCAAUCACACAGUCUAGCAAUUUGCAGGACCUUCAAACGCAGNGUGAAUGCUGCCAAAUCUCUUCAUCGAACUUUGGAGAAUAAAAUAGGAUUCUUUCAAAAUNGCUUUGAUCUUGACAAUGCAAGAUAAAACGGAACUCUCUUGGGAUAUCUUAGCUCAACUUNUUAAUCGAAACUAAGUUGAAGAUUUGCAUGUGCCCGGACAAAGGAUAUUAUCUUUUGCUGNAUCAAAAGCUCUCCUUUUUCGGUAACUCUAGCAUUAGAUCAAGCUAAGACAAACAUGUCGNGGUUUAUAUGUAUUUUUGAAUGGUGAGUAUGGACUACAUAUGUCAGACUCGGGAUCAAUGNGGAUCGGGAAAUACUAACAUCACCGAACCAGAUGUCAUUAUUUUCCGAAUUCGAAAGUCUNCCGCCAUCUGAUAUAAUUCCUAAUCGAGAUUAUUUCUCUCGAUCUACAAGUCAAGGUAUGNAUUGACGCAAUCUCUAUCGAGAGACCGGCUUUUCAGUGGGAAAAAUUUAACGUAGAGAGANAGAUUGGAUACUAAUCCACGUUAUCUUUCUGAGUCGAUCAUCUGCGAUCAAGCUGAGCUCNAUCAGGCGAGAUAUUUGUUCUGCUCCGACGGAUAAUCGGGAAUAUCGCAUUCGUCACACUNCGCUACGACAUCACAUGCACAGCAAUUGCUGCAGCUAGUACCGCAUUCCCGAAUACUCUUNAAUUACUACAUCAAUGACCAGUACGUAUGUCACGGAUGAACAAGACAAAUUGACAUCCCUNAAUAUGAAUUUCCGUUACAAACAACCCACCUCAGAUAUUAUUUAAUAAAACUUAGGAGAANAAACAUACGUACAUCACAUACGAACUUAGAUUUACAAUAAUGGACAUUCUCGAAAAUCAANCCUCAAUUUGAUUUUUACUCUUUUGUCUGCGCUCUAGUUUUGUUAGAAGAUCUAACUAAGNAGAGUCUGGGAAUCUAAUCUUCUAGAAACCUCGAGCGAGUUUUCACUAGUUACGGAAGAUNGGCGAAUUCGAAGAAGUUGAGGUCACAUCUGGCUCGAACUUAGGAUAAUCAAAGGUCCUANAGACAUUGUACAUUAAAUCUUCGAAUUCACGUGUAAAUAAGGUAUUAAAUAAAACUUAGGNAGAUUCAAUAAAAACUUCAUCAUGAAUUUCAGCGAAGCAUUCGAGUUCGAUUCGAUUCGGNGAACGUCGUUCUGCUGCUCCAGCAGAGAAGCAUCUCCGGAGUCCCAUACUUUCCUCUUUANAUGGAUAGAUUGAGGUUGCUCUAUAACGAAGUAUUACACUACAAAGAGAAAACUAGAUUUNCCGCAAAGCAAAUUCCCUUAUAUCAAAUCGGAUGUUCCACAAAUAAUUAAAACAAUUUGUNAAGCAUAAUCUAAUUCACUCCUAUUCUGGUCUCGAACGCGUUGCGACGCCUUGGAUAUUANGAUUUAAUAGGAACAUCAACUUCGCCGCAUAUUGUCACGCCAGACCAAUAUCCCGAACUANUUUACUCGCACCUUGAUAUCAAGAAAAAGUCUAAAAAUAUCCUUAGCUGAAUCAUCAGAGNCUUUAUCGCUCAGAAUUAUGUGCGUUUAAGAAGUGGGGAACGUUGCCGGAAGUUCAAAACNUUUAUAGAAAAUGAGGAGAAAUUCAUUCCUGAGGCUAAGGAGGACACAUACCUUCAUUACNGAAUCCUAUAGAUACUGGGAUUUUCUAGUCGAGAAAUAUCGACAACGGAAACAGAAAAAANCAAUUUGGGAAACAAGGUCUCACUAUCGGAGAGUCGAUUUUCUUUUUUUAUGAUGGAUUCNAUUAUGGAACAGAUUGGAUUUAGUACUAUGGUUACCGAUGAAAAUGGAAAACCUAAAAAANAUAGCACCUAUAAGAAGAAUGUAUUCUUUUGAACAACUUCAGAUGAUUCAAGACGUAUGUNCUUGCGCGAUUUAAUGCCUUUCUUGCUUUGGAUGCUAAUAUGCACAAUUCUAACCAGCAANCUGCGAACAUUUCUUAAAAAAUUAUUAUUAUGGCAGGAGGCUGUACUUAAAGAAUAUGGANAACAAAGGAUACGAACUAGUUAAAGGUCCAGAAUCCGUAACGAAAUCUUACCUAACAUCANUUAACACAAGGAGAUGUAUUACCUCUUAGCUCAUUCGUCCGUACAUGUGCCAAAUUGCAANGAAAAGGAACGAAAAUUAUCUAAAACAAAAACAUGUCCUUUAACGUCUCAAUUAAUAGAUNCUUAUAGAAUCUACCAACGAUAUUUCAAUUGUUACCGAACUUUUCGGCUGUACUAAGAUGNUCUGGACAUCCAUUUGUCUAUGCUGAGACUUCUUCUAUUUCGGUUAAGCAAGAAGCGUGUNCCGACGGGAAACCUAGAUCUUUUGGCAAUUAGAGAUUACCAUGCGCAUUUUAAGCGACUUNGUACUAGACCGAUAUCUUAAAAAGAAUCUAGUUUGGCCUGCUUUCACAGACAAAGAUGGANCCUAGGAAGAAUACCAAAUUGUAUAAUCUUUGGAAAUCUGGGAUAACAAAAAUUAAUGACNUCAAGUUAUCCAUUAUCAGAUCUUUAUGAGGUCGAAUUUCCAAAAUUUAUGGAGUUUGAUNUAUUCUCCGGACUAUCUAGAUAUGAUAGACGAUAAGGCAAUCUGUCCCGGAGCAGAUAAANUCUUCUGGCUUCUGGUUUAAACAAUCAGGAGAAAGUUAUCGACGGUUGUUAGAAUCUUUANAUCAAAAGAGCCGAUGUCGAUACAUUUCAGAUAGUAGAACGUAUGCGGAAAGGAAAAUUUNCACAUUGAUGAACGAAUUGUAGAGUUGACUCAAAAAGAGCGAGAGUUUAAAACUUCUGCUNCGAUGUUUUUGUAAAUUGACAUUUGAAGUUCGCCUGUUCUUCGUGUUAACUGAAACUAAUNCUGAAGAGAUUUAUGGGCGGUGAUUCUGGAGAUAAUGGGUAUUUACCUCAACAAACAAUGNACGAUGACUAAUACUAAAUUGAAGAAACGAUUAUAUGAUCUCACUGCCAUAAACAAUAGANGAUAAUACAUGUCUAGUAGAGGUAGAUUUCUCGAGGUGGAACUUAAGAUGGCGGGCAUGUNUCUGUGAAUCCUAUUUCCCGAUCGUUAGAGAAAAUAUUUGGACUCCCGGGAGUCUUUUCUNCAAGCUCAUUCGUUUUUCUCUCAGUCAACAAUCGUCAUGACAGAUAAAAACACCCUUCCUNCCUGGAGUGACACCGAAUUCGCAUGCAUCUACUUGGCCCGAGUCGGAUUUAGUUUGGAGANAACCACUUGGGAGGAUUCGAAGGAAUUCAACAAACUCUUUGGACUAUUUGUACGUUAGCGNAUGAUGUAUUACGCAAUUCAAGAUGAACAAUGUUCAUUUCAAAUGGCGGGACAAGGAGAUNAAUCAAGUCUUCUUUUUAUCCUUCAAUACCAAAAAACAAUCACUGAGUCUAGCAUUACAANUCCUUCCUAUACUCAGUAGAAAGAAGAUGCGAACGACUUAAUCAUGAAGUUAAACCAGAGNGAGUGUAUUGAUUCUCGAACUGUAUUAACGUACGGAAAGGAGAUUUACAUACGUGGUGUANCACACUAUGUAUAGUCUUAAGUUCUCUUCCAGGGCCUUUUCGAGAUUAGACUAUACUAUUNCCAUCCCUGAGCAAGGAAAUAUCUGGUGUGGUUGCUAAUUCGGUAGCCGUGGCUGGUACANCUUCAUAAUUCAUUUAGGGCUGUUUGGUGGAAGCAUAUUCAAGUAAUUCUACUGUUACGUNCGUCGACUGGCAUCUCCUCUUUACGCCUUAGAACACAAAGGACUUUCUCGUUUAUUAAGANUCCACAACUUCAAGAAAAGUACUCUUAAUUCCCGGAUNNNNNNNNNNNNNNNNNNNNNNNNNNCUAUGAUGCCCUGGACUCGUUACUUUAGCAAAGGAGAAUCAGACGAUUUGUCCUUCGANUGUUGCAGCAACAUAUUAUUUGUCUCAAACGGAACCAAUCGUGAAGAAUUAUAUGUGGUUNGCUUCGAACUCAAGAGUUUGUACCGCACAAAAUUGAUGCGACCAAUCUCAUCAAUGAUCCNUCAUUCUAUUCCCAUCGAUAAGCCAAACGACGCAUCUCAUCUUAUAUCGAAUGCUGUAUCNUAAGGCAUUACCAAGUCUAGUUAAUAAUAAAGAUAUCAAGCAAUUGAUAUCAAAUGAUCUNGAGGCAGCAAGGUGAGAAUUAUAAAUUGCUUUUGACUAAGAUGAAACCACUUCACCCUCANAAUUGCGGCAGAUUUAUUCGAAUUAACUCCUGCUGGUCUGUAUAAUAAAACAAUUAAACGNCUUCUCAAUGACCAGAACUAUUGAAAAAAUUAUUCCGGGCAUCAAUUUCAUAGAUAGAAUNAUCUCAAGCUAAUUCUACAAUUUUAUCAGUUCUUCUUGAUAGAUGGGUUCUAGCUGCAAANGAAGCCUGGUGGACGACAUCCAGCUCCGUUUGAGAUGGCUACUGAGUUACGAACACAGUGNGAAUUUAGGACUCGAUAAUACUUCGAUAGGAGUUUACACACCUUUUGAUUUCGAAAUAGGNAUAUUUCAAACCUGGCAUACCUCUCAUUAGUGCCACUACAAGCACGAGGAAAGAUAUUCUNUACCACUUGCGGGAAUGCACCUCCCAAUUUCGGCACUUCCACAAAACAAAAGCUAUCCGANUCAUGGAUACAGAAUUGUGAGCACAAAUUCAACCAUGAAAGAUCUCAAGAACGCAGUUUUNGACUUAUUCUGAACUACAAGGAGACCCGUCAUUAAAGCCUCUAUUCCAAUCUAUAAUAUCNUGCUCGUUCUCCCUGGACGCUUGAGAAAUUAAUUCCCAUUUUCCCAGAAUCAAUCGGUGGNAACUGCAGUCCACCGUCAUAAAAGUACAUCUCACGUAUUUUCAGUUCUGGGAAGUUCAUCNGGUACCAACUCAUAUCAUAUUCAGUUCCGAUCAGGCGGGUAUUUUGUCAGGAGGAGAUGCNAGAUUAUCCUGUUGUAUUCCAAACGCUUUACAUGACACUUACAAAUCUCUACCAAAUACUNAUCUACAACGGAUAAUAUACUUCCCCCGAAUAUGUGUUAUUACAUUCCAAAUAAACUACGNCGAAAUCAAUACUUCUAUCUCGAAAAUUCCUCCAGAGCAACUGACAGAAAUCAAAUGGCC  >FirsU_Contig83  CUCGUUUAUUAAGAUCCACAACUUCAAGGAAAGUACUCUUAAUUCCCGGAUCACUAGGANGGAUUACCUAUGAUGCCCUGGACUCGUUAUUUUAGCAAAGGAGAAUCAGACGAUUUGUCCNUUCGAUGUUGCAGCAACAUAUUAUUUGUCUCAAACGGAACCAAUCGUGAAGAAUUAUAUGNUGGUUGCUUCGAACUCAGGAGUUUGUACCGCACAAAAUUGAUGCGACAAAUCUCAUCAAUNGACCCUCACUCUAUUCCUAUCGAUAAGCCAAACGACGCAUCUCACCUCAUAUCGAAUGCUNGUAUCUAAGGCAUUACCAAGUCUAGUUAAUAAUAAAGAUAUUAAGCAGUUGAUAUCAAAUNGAUCUGAGACAACAAGGUGAAAAUUAUAAGUUGCUUUUAACGAAGAUGAAACCACUUCACNCCUCAAAUUGCGGCAGAUUUAUUCGAAUUAACUCCUGCCGGUCUGUAUAAUAAGACAAUUNAAACGCUUCUCAAUGACCAGAACUAUUGAAAAAAUUAUUCCGGGCAUCAAUUUUAUAGAUNAGAAUAUCUCAGGCUAAUUCUACAAUCUUAUCAGUUCUUCUUGAUAGAUGGGUUCUAGCUNGCAAAGAAGCCUGGUGGACGACAUCCAGUUCCGUUUGAGAUGGCUACNNNNNNNNNGGCANUACCUCUCAUUAGUGCCACUACAAGUACGAGGAAAGAUAUUCUUACCACUUGCGGGAAUGNCACCUCCUAAUUUCGGCACUUCAACAAAACAAAAACUAUCCGAUCAUGGGUAUAGAAUUGNUAAGCACAAAUUCAACCAUGAAAGAUCUCAAGAACGCAGUUUUGACUUAUUCUGAACUACNAAGGAGACCCGUCAUUGAAGCCUUUAUUCCAAUCUAUAAUAUCUGCUCGUUCUCCGUGGANCGCUUGAGAAAUUAAUUCCCAUUUUCCCGGAAUCAAUCGGUGGAACUGCAGUGCACCGUCNAUAAAAGUACGUCUCACGUAUUUUCAGUUCUAGGAAGCUCAUCGGUACCCACUCAUAUCANUAUUCAGCUCCGAUCAGGCGGGUAUCUUGUCAGGAGGAGAUGCAGAUUAUCCCGUUGUAUNUCCAAACGCUUUACAUGACACUCACAAAUCUCUACCAAAUACUAUCUACAACGGAUAAGANUACUUCCCCCGAAUAUGUGUUAUUACAUUCCAAAUAAACUACGCGAAAUCAAUACUUCUANUCUCGAAAAUUCCUCCAGAACAACUGACAGAAAUAAAAUGGCCUGAUCUUACAGCAAAUANAGUUAGCGUGGGUAGGGGAAAUCUUUGCAUCCGAAAUCCCAGAGAUUCCACUUCCAUCUGNUCAUACCACAUAUAUUAUCUCCAUCAUCUGAUCUGGACUUAAUCUAUUCUUACAUCGAAUNCCUGUAUCUCUCCCGGUGCAGAUCAGAAGAAACCUUAUGAUGGCAUACUUGGUACGGUAGNAUAUCUUUGACUUUAAAGAAAUAUCCCGAAUAGAUCCAAUUACAGUCGAGCAUGCAAUUUNGUUGGAAUAUUAUAACUGAUGCAUAUUAUUCGAUAUUUGUAUCUAAAGCACCCGAUAAAUNCUCUUCCUCUAUUCAAGAAGAUUCUAAAAACUAAAGCAACAUAUUAUUCAGGAGAGUGGGNUGAGAAUCCGUUUACAUCCUAUGUUCGAGAAUUCCCAAUAUAAUCAGCUCCGUCGAAUAANAUCUUCAGCCGACUCGAUCGGGUUACNAAACGUCCAGUAGACUAUAUGGCCACGCAAUUGANUAAGAAUGGCUCAUACGCUAUUGUCANACUCGAGGAAUGAGCAGUAUACCACGUUUAAUCCNUAUUCAGUAACUGGAGAGAAUCAUCUNGCUCGAUUAGGAAGAAGGAGAUUGAUUUUAGCUCNACGCAAUAGCAACUUAUCCGAUGCAANUCACUCACAAGUUUGAGAAAAUCCGUAUUAAGCANUUGAACCACCUACCGAACUUCUAAAACAAGACCCAGCAACUUAUAUCUCUAUAGUGACAANGAAAAAUCUCUCGGAANNAGAUAUCUAAUCUAGAGUACACACUUCCAGAUAUGCCGUGCUNUAUUCUUGCAUCGCACAGACAAGGAAGCUAUGCGUAUGCUUAGAGACAGACCACGAUACGNAAGAUAAAAAAGAGACAGUAAACCAUCCGUUGAAAUUCGCAAACCAUGGGAUAUGCAAGGNUUGAUUACUCGAAUUAUUACGGGGCCCUAGAACCAACCGAUCCGCCAACACGCAACGAACNGUAAUAGUAAUGAUAGACUCCGCAUACUUCGUCGUCGAACCAUAGGAAUAUAUUCACCUCNUAUAUAGUGACUGGAAUGCCAUUUUUGAAAUCUUAUUUGAAAGAGGAUUAAGGAGACAACNAAAAAUUCCACGUAUUAGGUGUAGGACGAGGUGCGACUUCUCGUGCGUUAGUAGAUCGUCNAUGUAGGUGUUGUAGGGUAUGAUCUCAUCUCUUCUUUCCCCUCAAUUUCUCAUCGAUCGGNCAUCAUAUAAACCUCCUGAACUGAUCAUGUCUGCUAAUACUGGAAAUUUCUCCUGGAGCANAUCACACCUAUAUGGAUGAUGGUGAUGUCUUAAAAGGACACUUGGACUGCUUUGAUGAAUNCCAAACCACUCCUGGUUAUUGACUUAGACAUAUCUUUCCAGCUGCUGAAGACAUUGAUGCNUUAGAUUGCCAAUCGAUUCAGAAAUCAUUUUGAGAUAUGUCGGUAGUGAAGAUGAGAUUANAGUGCCUUAUAAGUAUGAUACGUCCUACGCUGGUCUUUUCUCUGUGUGUUUCUGAGAAUCNAAGUAUCAGAUGUCGUCCUCUACACCACCCAGUUAGGUUCGCUCGGGAAUGGUAAUUAUCNAGAGCAUCGAGUUUAAAACAAAGAAUGAGAUCAAUUAUUCAUAUGCCUCCACAGAGUUAGNUAUCCCAAUUCUGGAAUGUGGAACCGAAUAUCUCUAGACAUUUCCAAUUAGANNGAAGUANUAUAUUCACUUAUGGGAUAUAAUGCAUGCAAGUUUGAAUCCAAUAAGUCUAACAGGUAAANGAUCUUCGAUGUCGGGCAAUUUGUGAUAAUAUUUUCAAGUAUGAUAGUUGAAUGACAUUUNGACUUGAACUACUAUUUAAUAAAACUCAGGAGGAUGCCUUCGUAUAAGGAGAAGUAUUUCNUCAAUCUGCAAUCGUUUAACUACAAUGGAAAGGCGAGUAUUACUUGAUGAAUCAAUCCUGNCUAGAUUUACGGCAAACUAUCCUAAAUCUGCAAACUCAGUUAAAUAAUCCGAAAAAAGAANCUCGAUGAAUUUCUAAUCAAUCAGCUGCACCUAGCACUUACCGAGUUGAUUAAAUAUGCANAAUCCUCAAUUUCAAUUACCCGCGCAUUUCAAAGGUAAAGAAAUCAAUGAAAUUUUAACCNAUUACUACUGAGAUGAUAUUUAAUGCGGUAUCUUUAUUGAGGGAUGCAACUGUUCGAGCUNCAAACAAUAAUUUAAGACAUUCCAUCCAAACCUAUCAAAGAUAAUCAACCCUCUAGAAAUNCAUUUAAUCAAN  >FirsU_Contig89  UGUAUAGUCUUAAGUUCUCUUCCAGGGCUUUUUCAAGAUUAGAUUAUACUAUUCCAUCUCNUGAGCAAGGAAAUAUCUGGUGUCGUUGCUAAUUCGGUAGCCGUGGCUGGGACGCUUCAUANAUUCAUUUAGAGCCGUUUGGUGGAAGCAUAUUCAAGUAAUUCUACUAUUACGUCGUCGACNUAGCAUCCCCUCUUUAUGUCUUAGAACACAGAGGACUUUCUCGUUUAUUAAGAUCCACAAN>Ss-AA_clean.1_(paired)_contig_398  GAAGAGAACUUAAGACUAUACAUAGUGUGUACACCACGUAUGUAAAUCUCUUUUCCGUACGUUAAUACAGUUCGAGAAUCAAUACAUUCCUCUGGUUUAACUUCAUGAUUAAGUCGUUCGCAUCUUCUUUCUACUGAGUAUAAGAAGGAUUGUAAUGCUAGACUCAGUGAUUGUUUUUUGGUAUUGAAAGAUAAAAAGAAGACUUGAUUAUCUCCUUGUCCCGCCAUUUGAAAUGAACAUUGUUCGUCUUGAAUUGCGUAAUACAUCAUCGCUAACGUACAAAUAGUCCACAGAGUUUGUUGAAUUCCUUCGAAUCCUCCCAAGUGGUUUCUCCAAACUAAAUCUGACUCGGGCCAAGUAGAUGCAUGCGAAUUUGGUGUCACUCCAGGGGGAAGGGUGUUUUUAUCUGUCAUGACGAUUGUUGACUGAGAGAAAAAAGAAUGAGCUUGAGAAAAAACACCUGGGAGUCCAAAUAUUUUCUCUAACGAUCGGGAAAUAGGAUUCACAGAACAUGCUCGCCAUCUUAAGUUCCACCUCGAGAAAUCUACCUCUACUAGACAUGUAUUAUCCCUAUUGUUUAUGGCAGUGAGAUCAUAUAAUCGUUUCUUCAAUUUAGUAUUCGUCAUCGUCAUUGUUUGUUGAGGUAAAUACCCGUUAUCUCCAGAAUCGCCGCCCAUAAAUCUCUUCAGAUUAGUUUCAGUUAACACGAAGAACAGGCGAACCUCAAAUGUCAAUUUACAAAAACAUCGAGCAGAAGUUUUAAACUCUCGUUCUUUUUGAGUCAAUUCUACAAUUCGUUCAUCAAUGUGAAAUUUUCCUUUCCGCAUACGUUCCACUAUCUGAAAUGUAUCGACAUCGGCUCUCUUNNUCCGGAGAAUAAUCAAACUCCAUAAAUUUUGGAAAUUCGACAUCAUAAAGAUCUGAUAAUGGAUAACUUGAGUCAUUAAUUUUUGUUAUCCCAGAUUUCCAAAGAUUAUACAACUUGGUGUUCUUCUUAGGUCCAUCUUUAUCUGUGAAAGCAGGCCAAACUAGAUUCUUUUUGAGAUAUCGGUCUAGUACAAGUCGCUUAAAAUGCGCAUGGUAAUCUCUAAUUGCCAAAAGAUCUAAGUUUCCCGUCGGACAUGCUUCUUGCUUAACAGAAAUAGAAGACGUUUCAGCAUAGACAAAUGGAUGUCCAGACAUCUUAGUACAGCCGAAGAGUUCGGUAACUAUCGAGAUAUCGUUGGUAGAUUCUAUAAGAUCUAUUAAUUGAGACGUUAAGGGACAUGUUUUUGUUCUAGAUAAUUUUCGUUCCUUUUCUUGCAAUUUGGCACAUGUACGGACAAAUGAGCUAAGAGGUAAUACAUCUCCUUGCGUUAAUGAUGUUAGGUAAGAUUUCGUUACGGAUUCUGGACCUUUAACUAGUUCGUAUCCUUUAUUUCCAUAUUCUUUAAGUACGGCCUCCUGCCAUAAUAAUAAUUUUUUAAGAAAUGUUCGUAGUUGCUGAUUAGAAUUGUGCAUAUUAGCAUCCAAAGCAAGAAAGGCAUUAAAUCGCGCAAGACAUACGUCUUGAAUCAUCUGAAGUUGUUCAAAAGAAUACAUUCUUCUUAUAGGUGCUAUUUUUUUAGGUUUUCCAUUUUCAUCGGUAACCAUAGUACUAAAUCCAAUCUGUUCCAUAAUGAAUCCAUCAUAAAAAAAGAAAAU  >Ss-AA_clean.1_(paired)_contig_3470  AGAAGAUUGGAUACUAAUCCACGUUAUCUUUCUGAGUCUAUCAUCUGCGAUCAAGCCGAGUUCAUUCGGCGAGAUAUUUGUUCCGCUCCGACGGAUAAUCGAGAAUAUCGCAUUCGUCACACUCGCUACGACAUCACAUGCACAGCAAUUGCUGCAGCUAAUACCGCAUUUCCGAAUACUCUUAAUUACUACAUAAAUGACCAGUACGUAUGUCACGGAUGAACAAGACAAACUGACAUCUNNNNNNNNNNNNNNNCAUACGAACUUAGAUUUACAAUAAUGGACGUUCUCGAAAAUCAACCUCAAUUUGAUUUUUACUCGUUUGUCUGCGCACUAGUUUUGCUAGAAGAUCUAACUAAGAGAGUCUGGGAAUCGAAUCUUAUAGAAACCUCAAGUGAGUUUUCACUAGUUACGGAAGAUGGUGAAUUCGAAGAAGUUGAGGUCACAUCUGGUUCGAACUUAGGAUAAUCGAAGGUCCUAAGAAAUUGUACAUUAAAUCUUCGAAUUCACGUGUAGAUAAAAUAUUAAAUAAAACUUAGGAGAUUCAACAAAAACUUCAUCAUGAAUUUCAGCGAAGCUUUCGAGUUCGAUUCGAUUCGGGAACGUCGUUCUGCUGCUCCGGCAGAGAAGCAUCUCCGAAGUCCAAUACUUUCUUCUUUGAUGGAUAGAUUGAGGUUGCUCUAUAACGAAGUAUUACACUACAAAGAGAAAACUAGAUUUCCUCAAAGCAAAUUCCCUUAUAUCAAAUCGGAUGUUCCACAAAUAAUUAAGGCAAUUUGUAAGCAUAAUCUAAUUCACUCCUAUUCUGGUCUCGAACGCGUUGCAACGCCUUGGUUAUUCGAUUUAAUAGGAACAUCAACUUCGCCGUAUAUUGUCACGCCGGACCAAUAUCCUGAACUAUUUACUCGUACCUUGAUAUCAAGAGAAAGUCUCAAAAUAUCCUUAGCUGAGUCAUCAGAGCUUUACCGCUCGGAAUUAUGUGCGUUUAAGAAAUGGGGAACGUUGCCAGAAGUUCAAAACUUUAUAGAAAAUGAGGAGAAAUUCAUUCCUGAGGCCAAGGAGGACACAUACCUUCAUUACGAAUCCUAU  >Ss-AA_clean.1_(paired)_contig_4115  GUGGAUUAGUAUCCAAUCUUCUCUCCACGUUAAAUUUUUCCCACUGGAAAGCUGGUCUCUCGAUAGAGAUUGCGUCAAUCAUACCUUGACUUGUAAAUCUAGAGAAAUAAUCUCGAUUAGGAAUGAUAUCGGAUGGCGGCGACUUUCGAAUUCGGAAAAUGAUAACAUCUGGUUCAGUGAUGUUAGUAUUUCCCGAUCCCAUUGAUCCCGAGUCUGACAUAUGUAGUCCAUACUCAUCAUUCAAAAAUACAUACAAACCUGACAUGUUUGUUUUAGCUUGAUNNNNNNNNNNNNNNNNNNNNNNNNNNNNNNNNNNNNNNNNNNNNNNNNNNNNNNNNNNNNNNNNNNNNNNNNNNNNNNNNNNNNNNNNNNNNNNNNNNUGAUUGAAGGUCCUGCAAAUUGUUAGACUGUGUGAUUGCAACAUCAGUCGGACUAGACAAUUUACACAAGAUGACGAGGUCUUGGCAGUUAUUCGAUAGAGUAGGCAUAUCGCUGAUAAGAAUUUAUGUUUAACUCCUAAGUUUUAUUAAAUAAUUCAAGAGAUCUUUCAUUCAGGAAUUGUGCAUACGAGAUGAGAAUAGAACUUAUUGGGCUGGAGCCUCGGGGACAGUUUCCUCAGCAGUCUCUCGGAUGUUGAGAAGAGCG  >FirsU_Contig195  NUUAAAACAAGACCCAGCAACUUAUAUCUCUAUAGUGACAAGAAAAAUCUCUCGGAAAAUNAUCUAAUCUAGAGUACACGCUUCCUGAUAUGCCGUGCUUAUUCUUGCAUCGCACAGACAAGGGAAGCUAUGCGUAUGCUUAGAGACAGACCACGAUACGAAGAUAAGAAAGAGACAGUAAACCCAUGCAUUGAAAUUCGCGAACCAUGGGAUAUGCAAGAUUGAUUACUCGAAUUAUUACGG  >Ss-AA_clean.1_(paired)_contig_1094  UCGUCAUGAAACCACUCUCCCUCAGCCUGGAAUCAAGAACAUUUUCCCUUCGAGUGCAGCUCUUUUAACGGAGAGUACUAAGGCAAUCAACGCAUGUCGAGAAAGAAUAAAUGCGUUAGAAGCAUUAGUUCAUUCCCAGGGUCAACAAAUUUCUGACUUACAAGAUCAGAAUUCUGCACUCCGAGUUGAAAUUCAGGCAUGUCACUCGUCUGUUUCUUUGCAUAAGCAGACAACAGCCGAUGCCAUUAGCGCAGCUUCUGCUGAUCUACGUGCUCGGAUUGUCGACGCAGUGUCCAUUUAUAAAGAGACUCCAGGAUUAGUGGAAAGAUUGACAGCAGCAACCAACAAUAUAUUAUCCAUGUAUCCGGAGGAAUUAAAAACAGGUUUGAAGAAAGUAGAAUUGACGAAGACGGAUCAAGUAUUGAUGCAAAAAAUCACUACCACCUCAACUUUACCUAAGGCCAAGCUUCCGAAGCAUCUCAGAAAGUAGACCGUAAAUCUAUGUUCGGAACUGUAAGAUUAUUUAAUAAAACUUAGGAGAUUCAAUUAUAGUCUCAUUCUCGUCUCACACCACUUCUCAUCUCAUUCUCAUUAUUACCAUGUCUGACUCUCGACGAACCAUUCCGGAUGUCUUUAAAGACAAGGAUACCUAUGGAGUGAAUAUUGAAGCUAACCGCGACCGACUCCCUACUAUCGAUUUGACCAGAAAUUGUGAAUACUCGGAGAUUUUUGUUAGUGCAACUGAUCUUCCCGUUCGUAUGUGCUUGGCUGGAAUUCAACAUGCAGAGGAUUCUGACGACAUCGCCAAACUAGUCAUCGGUGCGAUGAUUUCUGCAAGCGGAGUCAAUUAUUCUACUGCUCGAUUGGCUAAUGUAUUCAAAGAUACUUCAGCUGAAAUCAAGAAUUGGGUUAUAUCUCGAUUUAGUCAUGUCCCGUCUGAAGAUGCCAGCGAAGCCGAGCUUCCCGCGUUGCCUAAUUCAUGCGUUCCAAAAUGGUAUGAGGAGUUGGAAGUUCCCGAUGAAUCAGUGUUGUUUGCCUACGGGGUUGAUUCGAUGGAAAUUGCGGCUUUUGCCGGAAUAUUGGCUAUUGCUGUAGCCAAGCAACCGAGCCCAGAGAAUCUAGACGCGUUUAAUCUUAAACGCCGAAACGCUAUCUCUCAGUUCAUGCCUUCAGGUGAACUGAAAGUCUUUACUGACAAUUCUCCUUAUCUAUCUCAUGCAGUGUUGAGCAAGAUUAACAAGACAUUCAACUCCAUCAUUCGUGACCGGGCCUUGGUUAUGAGCUCGAUUGUCGACAAGGACGAUCCAAUGGUUUCAGGCA  >Ss-AA_clean.1_(paired)_contig_2273  GACACUGCGUCGACAAUCCGUGCACGUAGAUCAGCAGAAGCUGCGCUAAUGGCAUCAGCUGUUGUUUGCUUAUGAAGGGACACAGACGAGUGGCAUGCCUGAAUUUCAACUCGGAGUGCAGAAUUCUGAUCUUGUAAGUCAGAAAUUUGUUGACCUUGGGAGUGAACUAAUGCUUCUAAUGCAUUUAUUCUUUCCCGGCAUGCGUUGAUUGCCUUAGUGCUCUCCGUUAAAAGAGCUGCACUCGAAGGGAAAAUGUUCUUGAUUCCGGGCUGAGGGAGAGUUGUUUCAUGACGAUGUGNNNNNNNNNNNNNNNNNNNNNNNNNNNNNNNNNNNNNNNUCUUGUUCCACGGGAAUCUCUCUCAUAUUUCCAGGUAAGGUGCCAGUUAAUGCAGCAAUAGAGGUUCUUAAUUCCUGAUCGUUCUCUAUGACACUUCCGAGUCCUUUUAUCAAUUCGAGUUCCUCGAA  >Ss-AA_clean.1_(paired)_contig_1824  GAUGUAUCUAGAAAACGUUGGAGAGCAUGGGCAGCAGCGAAAUAUUCUGUUUCGAGAUCGCUGAAUUCUCUGAAAAAUUGAGGAUACUUUCUUGCAUACUUGGUAAUCAGCAAGAGAGGACUUAGGGAUGCCCCAAAGGUAAGACGAAACAUAACGUAAAACAUACGUUGAGUGCCUGAAACCAUUGGAUCGUCUUUGUCAACAAUCGAGCUCAUAACCAAGGCCCGGUCACGAAUGAUGGAGUUGAAUGUCUUGUUAAUCUUGCUCAACACUGCAUGAGAUAGAUAAGGAGAAUUGUCAGUAAAGACUUUCAGCUCACCAGAAGGCAUGAAUUGAGAUAUAGCGUUUCGGCGUUUAAGAUUAAACGCAUCUAGAUUCUCAGGGCUCGGUUGCUUAGCUACAGCAAUAGCCAAUAUUCCGGCGAAAGCUGCAAUUUCCAUCGAAUCGACUCCGUAGGCAAACAACACUGAUUCGUCGGGA  ACUUCCAACUCCUCAUACCAUUUUGGAACGCAAGAAUUAGGCAAUGCGGGAAGCUCGGCUUCGCUGGCAUCUUCAGACGGGACAUGACUAAAUCGAGAUAUAACCCAAUUCUUGAUUUCAGCUGAAGUAUCUUUGAAUACAUUAGCCAAUCGAGCAGUAGAAUAAUUAACACCGCUUGCAGAAAUCAUCGCACCGAUAACCAGUUUGGCAAUGUCGUCAGAAUCCUCUGCAUGUUGAAUUCCAGCCAAGCACAUACGGACGGGAAGAUCAGUUGCACUAACAAAAAUCUCCGAGUAUUCACAAUUUCUGGUCAAAUCGAUAGUAGGGAGUCGAUCGCGGUUAGCCUCAAUAUUCACUCCAUAGGUAUCCUUGUCUUUAAAGACAUCUGGAAUGGUUCGUCGAGAGUCAGACAUGGUAAUAACGAGAAUGAGAUGAGAAGUGAUGUGAGACAAGAAUGAGACUAUAAUUGAAUCUCCUAAG  UUUUAUUAAAUAAUCUUACAGUUCCGAAUAUAGAUUGACGAUCUACUUUCUGAGAUGCUU  CGGAAGCUUAGCCUUAGGUAAAGUUGAGUUAGUAGUGAUUUUUUGCAUCAAUACUUGAUC  >Ss-AA_clean.1_(paired)_contig_4907  CGCUCUUCUCAACAUCCGAGAGGCUGCUGAAGAAACUGUCCCCGAGGCUCCAGCCCAAUAAGUUCUAUUCUCAUCUCGUAUGCACAAUUUCUGAAUGAAAGAUCUCUUGAAUUAUUUAAUAAAACUUAGGAGUUAAACAUAAAUCCUUAUCAGCGAUAUGCCUACUCUAUCGAAUAACUGCCAAGACCUCGUCAUCUUGUGUAAAUUGUCUAGUCCGACUGAUGUUGCAAUCACACAGUCUAGCAAUUUGCAGGACCUUCAAUCAAAAGUAAAUGCUACCAAAUCUCAUCAUCGAACUUUGGAGAAUAAAAUAGGAUUCUUUCAAAAUGCUUUGAUCUUGACAAUGCAGGAUAAAACUGAACUCUCUUGGGAUAUCUUAGCUCAACUCUUGAUCGAAACUAAGUUGAAAAUUUGCAUGUGCCCUGACAAAGGAUAUUAUCUUUUGCUGAUCAAAAGCUCUCCUUUUUCGGUAACUCUAGCGUUAGAUCAAGCUAAAACAAACAUGUCGGGUUUGUAUGUAUUUUUGAAUGAUGAGUAUGGACUACAUAUGUCGGACUCGGGAUCAAUGGAAUCGGGAAAUACUAACAUCACUGAACCAGAUGUUAUCAUUUUCCGAAUUCGAAAGUCGCCGCCAUCCGAUAUCAUUCCUAAUCGAGAUUAUUUCUCUAAAUCUACAAGUCAAGGUAUGAUUGACGCAAUCUCUAUCGAGAGACCAGCUUUCCAGUGGGAAAAAUUUAACGUGGAAAGAAGAUUGGAUACUAAUCCAC  >Ss-AA_clean.1_(paired)_contig_2381  CUCCUAAUUUUGGAACUUCAACAAAACAGAAAUUAUCUGAUCACGGGUAUCGCAUUGUAAGUACGAAUUCAACUAUGAAAGAUUUGAAGAAUGCAGUACUCACAUACUCUGAAUUACAAGGAGACCCUUCGUUAAAACCACUGUUUCAAUCGAUUAUUUCUUCUCGUUCUCCUUGGACCCUAGAAAAAUUAAUACCAAUUUUUCCCGAAUCAAUUGGUGGAACUGCUGUUCAUCGCCACAAGAGUACAUCUCAUGU  >Ss-AA_clean.1_(paired)_contig_1368  UAUCCAUUAUCACCCGAAUCACCUCCCAUAAAUCUCUUAAGAUUAGUUUCUGUUAAGACAAAGAAUAAACGACCUUCGAAUGUUAGUUUACAGAAACAUCGAGCAGAGGUUUUAAAUUCACGUUCUUUUUGAGUCAGUUCGACGAUUCUUUCAUCGAUAUGGAAUUUGCCUUUUCUCAUCCGCUCCACAAUCUGGAAAGUAUCUACAUCAGCUCGUUUAAUCAAUGAUUCUAGAAGUCGACGAUAACUUUCUCCAGAUUGUUUAAACCAGAACCCAGAUGAUUUAUCUGCUC  >Ss-AA_clean.1_(paired)_contig_100  GAAAGGGGGGGCAGAGCGGAAUCUAGAUUAAACAGACUUUAUUGCUACCAAGCUGGCCUUCUGGCUGGUCUUGGUGGCAAGUCCAAUCGCCCAGCGUUAAAGUAGGCAGACACCUCGCGCUAGUUUUUUCUUAAAUCUUGACUCAGCUACCUGGUAUCCGAAUCUUUGAAGGUGUCUGCAAUAGAUGAUAAGUGUGAUUCUUGGAGACACUCGUUCAAUGGCGGCGAAUUGGCAGCGCUUUCCAAGAGUUGGAUCUGCUUCAAGAUCACAUCAAGCGAUUCGGGCUCCUUCAUGCCUUCAAUCUCACUCCUCGCCAGUGCCAGUUGAUCACUUAGUUGACCUACAAGGAUCUCAGCUGUCUCGAUAGCUCUAAUAGCCUGAGCAGAGACAGUCAGCCAGUCUGCUUCGUAUGGGAGGGAUUGGGGCUCCUGAGGUAUUUGAAGAGAAUGAAGCUCCGCAAGUGUCUUAAUUCUCGUGAUCAGAGAUCUAGUCUUUGCUUGCACGGUUGAAUGGAUGAAAGCUAUUUUAGCUUUCUCUAGGCUUUCAAUAGCUCCUCCCUCGAUCAGCAUCCUCAACUUCGUCUCGGCGAUAGAAAGCUUUUCCUUGUACGAGGGUGACAUUCUGAUCUUAAUCAGUUUUUCUUAAAUGAUCACAGCUGCAGUGACCAUCCCUGUCGACCUCUCAUCAUCAACCUCGGAAGAAUCCGAGAGGACAUGUUGAAUAUAGAUGCCUGGCGAACACUUGGAACUGGAUGUCCUUGCACCUCGCUUGGGGCGGAGCUUUGGGCAAGGUCCGACAUCUUAUCUAUCAGAUCCUCAGUAGAGAGGGGACCGUGUUCAAACAUCAGGUUGAGUUUCCCAAGCAGAUAGAGUGCUAUGACGAUAUCUUCUGUCACAUCUUUUCCGCGCCGGUGGUUGCUUCCUGACAUGCUCCAAUCAACAAACAUUAAGAGGUAGUCUUUUGCAACUCCUACACUAACGCCUGGAGGAACUCGCCCCUUGAGCAACAUAGUUCUGUUCAGUUCAAAUUGGUUAGGGUAGAACGGAGAAUCCGGCGGAGAAUCGAGUGGGUCAACAGUUUGGAAGACCGGGUCACAAGCAUCUGUUGGUCGGCUUACAGCUUGGCACCCAGACGUCCGGGCCACUAACCAGUAUUCCAGCUCGUUAAUUGCAGAUCUGAAGAUGCAAAACGUUGAAAAUGUCCCUCUACACUCUUGGAAGAAGACGCCGGCUUCGGAGCUGGUCAUCUCUCGCCUAGUAACUAUUACCGUCUUGAUCAUACUCUUCAACACAGGGAUAAUGUCUUCCCAUAUCAUGCGCCUCCCCGGUUGAAUGUCAAUGCACACAACGCUGGGUUGCUCUGACAAGACAUCAGAGUGAACUUUGCUAUCGAACCAGUCUCCUCCAUGGUUGAAUACACUCUUGGCCCACCGCCAUGGGAGAGUAGGAGAGAGGAUAGGUAUCUCUGGAGGAAGGUAUCCGGUUCCUAGAGAACUGACUGAUGGAAGAUCAGAUAACAAGUCUACUCCAACAACAGAGCGUGCUCCCAAUGCGAAUACGUCUCGUGCCAUCGCUCCAUUACCGACUCCCACACAUAAGACUGGACCUUGCACUAUCGAACUCCGGACUAUUGAGAGAAUUCGAUAGCUCAAUCUGGAUAUAGAGGACCCUCCAAUACACCACCCAAGGUUCCUGCUUAUUAGACGUCGACUGUCAGAUCUCCUAUCGAUAGUGAGUAGUGGUUGUGAGAGGGGACUAGAUAAAGGGAAUUCGCCUGCUGUUCCUAACUGCCAGACAAUGUCAGGAGGUGGUAGAGGAGGAGUUGGUGACCUCUGAGAGGCAACGGAACGAGGACCAGUUCGCAAAUAUCUCUUGCUGUCAUCAAAGCUCAUAGCGGUUCUUGCUAGAACUCUUCCUGCAAGCACUUCGGAAGCUAGAACCGAUGACUCCUCGAAUUCUGGAUUUGCGAGGAGGUUCAAGAUCGAGAUGUGAUGGGUCACUCUCUCUGACUCCCUGGCGAGGCCUCUCAACCCGUUGAGAUGUCUCCGAUAUAUCCUUCUGCAAUCAUCAACUGAACCUCGAAUGCCAAGCAGGACAACGGCGCAGGCGACAUAGCAUCCCAGGACUCUCGACGGAGUCGCUCGCUCUGCGUAUGAUGACAGUAUCAUUCUAGAGUUGCUAAGAUUGCGGAUAUCCCGGUAACACCGAUGAGCCUCAUCCCUUAUAUAUGACGACAGGCGCGAUUUCAAGACAGACCCCCCAUGCUUUCCGGGUGCGAUCAGCACUCCUGAUGCCUGGACCCAUGAUUGUUGCCGGACCUGAGGGUUGUUAGCAAAUCUAGCGAGAGGUAAUGCUGCAACGCCAGAUAACGCGUCGAUGUAUGCGGAUAAGAUUACUCGAUCCAUCUUCCCUGUUUCGGAUAGAGCCAUCUCUACCGAUCGCGCAAGAACACCGCUCUUCCUGCAGCGAUCAUCAUUGCUUGCAAUCCAACUCCUAUGAGGCUACCCACAUCGAGAGAUAGUCCCGACGGAGCAUCUUUGGAGUCUAUUGCAGCCUCUGAUAAGACAGGGUUUUCGAGUUGAUCCAGGAAGUGGUGGAUCAUCAAUUUUAUUCCCACUUCGGUUGAGCAAGAUGGUGGGGUCUUCCACAGAGGGGCGAUAGACAUUGGCAUCUCCUCGCUCAGAACUUGAUAUAAGAUGUUGCUUGCACAAACAAGGGGAUUCUCUUGGAGAUUCCGUGUCAAAGACAACUCUGGAGCCUUUCCCUCAGACGAAACUGGAACACUAGUUAUGAGCAUUUCCGGAGUGAUGUCAUAGUGAAGGCGUAGAGUGGGAUUACCCGCAAAAGAUUCGGGGCUGGCUCGUAUGCAUUGAAGCCCAAAGAAUGAGUGCUCCUGAAAGCAGAAGGAGUAAUCUUUGAUCCCUCCACUAAGGCUAGUCAUUGAGUCUGUCUGGACAGUGGAAUGCUGGGUGAGUGAGAUAGGCCCUAACCACGCAAAAGCUUUUUCCUCAACGGUGCUAUCCCACCGAUGCGCAGCUACCCCUCCAACUACUGUAGGCAUAAACACUUCGAGGUCCCGUUCAUCAACCGUCGUCCUUUGCCUCAAAACUCCUCUUAUCGCACGCUUCAAUUCUCCACUGGCUGUCAACUGUGACUCCAUCAUUAACAAUCUCUUAGCUGCUAAAGCCGCUUUCUGAGUUCCCAAGAUCUCAACACCGGUCACUCGUCUAUGUUCCCACGUCUUGUCUCCCCACCUUCCCGGGUGUGGUCCCUUAGUGCUCAUCAUCUCAUGCCAGGGAACUUGACUAGUGAAUUCUAUGUAGUCAAGACUGUGCAAGUUCAUUCCUGGAUCCGCCUUGCAUUCGAGAGGGUGGAGUACUGUUACCCCUUCUAACUUAUUUGAUCCAUCUGGAAACCAUCUGUCACGAUAUUUGACUGCUCUCGAGAAGAUACUCCUGGAAGGGAGAGCACUGGUGAGGCCAGCUGCGGUUGACUGACUCAUAAAUGUUCUUAGUCGAGUCACUCUCUUGUAAUCUGCAUAGAUAACCGCAUGCCCAUAGUUAAUAGGAGCGUUGUUGCGUACCAACUGUUGUAUUGUCCUGGUGCCUGUGAACUUUUUAAACACUUUCUUCUUUACGCCAAUAACAGAGAGUUCAACCAGGUCUGCCAUGAGCAAUGGAUAAAACGGUUUAAGCUUGGUGAGGAAAGAUGCUAGAGACCUCCCGAAUUCGUCGACUGACGUGGACAUUAUCUGCUUGAGGUCAAUGUUUUGAGUAAUCGAGGGGACAACCGUCUUGAUAGCUUGGUCAACUUGCACAUCAGCACCGACGCUUGGCAAGAUAGGGACAGAGAAUGGAUCUUCUAAAAGGGUGGCGGGGUCUGGAUCAGGCUUCCAUGGCAAUGGUGUUUCUAGGUAUCCCAAGUACUGCUGUGCAGUGCGGCUAACAUGCGAUAGGCACACAAGAGCCGCGAUAGAGGAGCUUAGUCGAUCUGAACUACCUCCGAAACAAAAAUCAGUCGGUAUCUGAAUGGGGAGUCCACCUAGGUCGCUAGGAAUCGUGCAAGCCUGGUCAAGAUCGUGUUGGCUCCAGGAGUAAUUAAGAAGUUGUCUACUUGGGAGUGUUUUUGAUGCUCUUGGCAAGUACUCUCUAAAGAGUAUCAGAAAAUAUAGGUAUCCAAGAGAGGGAUCACUAAAUGCAUCUGCAGAGGCUCUACAGCCAGAACUUAUUGCAGAUAACCCGACCACUAGCCCCGGAAUGUUCGAGGUCCCAACAGGUGCGACCGUUGCUGCAUGUUUUAAGGUGGUUGGGAUCAAGACUCCAUCCUGCCAGCAGAUCUUGCUAUAAGUUACAGUUGAGCGAGAUGUCAAGCACUCAUCCGGUUUGAUGACUUGGUUAAGGCGUGCAAACCUCUUGCUGAGCUCCUCUGUACACAAGGACGACACUUCUAGUGCCUGCACCAUCUUAUCUUUCUGGUGAUCUCUCUGGUAGGACACUUCUAGGAUCUGAUUGUCACCUUGCCCUAAGAGCUUGUAUGCAACAACGUUAAGAUCCCGCAUGACAGCUUUAAUCUCUGCUUGUGUACAUGCUGUCCACUGAGCCUGCAUGAUCCCCUCAAACCCACCCAAGUGAUUUCUCCACACAUAUUUGCUCUCUGGCCACUCGCGAGGGAAACUCCCGGGCACCACGCCGUCAGGUGUCUCUGUUGAAACCCGAACAACAACUGUAGAGUUCUCGAAGAUCUCGUGUCCUUUUGAGAAUGAUCCUGGUAGUCCAAAGAUCGAGUCCGCGAUGUUGCUGACGGGAUCCACUACUACUCCUCGCCACAGCAGGUUCCACCGAGAUAAGUCGACUUCUAUAAACACAGUAUCUAUAUCAGGAUUCGAUUGACGAGCAGCCAUCCCCUCUAGAUGCUGCAUCGUUCCCUUCCUUCCCUUCGUCAUCGAUUGGUCUGGCAAAUAAUCUUUCAGAAGCCGCUUGAAGUUCUUCUCUGCUAGAGUGAGGAAGACGCGAACAGAGAAUUCAAGCAUGACGAAGAGUCUAGCUUCGAGCUUGAAUUCUCUUUCUUUGGGGUAGAGCGACAUAGAUAUGUCAGCCAAGGACAAAUUCAAGGUCCUGAUAUGCUCACAGAUGGCUUUGGGAUCGAACUCCUUCAUGCUCAGAAUUCGGAUAAUCAACUUCUUGGAUCUCUCAUUCGGUAUGUGCGCUAGAGAGGGCGCGUCCUUUUUGCAACUAUCCCAUGCCUUCCAAGCGUCUUGCGGAUCCAGUCCGGUCGACUUGUCAUCCAUCAUGUCCAAGUAAUCGAUGUUGUAAUCAAAGUCUAAAAGUUUUGGCCACGCUGAGUAAUACCAGUCUUGAUAAGCAUAAUCUGAUGGACCAAAUACGAGCACCCUCCUCUCAUGAAGCUUUUGAAGCCUAGUCUUCUUACCUGGAGAGUGGAUCAAAGCAGGCCAUCGAGCGUGCUUCUUGAUAUAACCUUUUAGCAGCAUGUCGCAAAAUUCUGCUCUCAUUCGCAUUGUCUCCCCAAAACCUGGAUUACCUCGAGUGGUACCGUGACUCCUAGCUGACUGGGACGCAAUCUCUGGAUCGAUGAGGGGGUGGCCUGAGAAUUUCAGACAGCCGAAGAGCUCCGCUGCUUCUCUGGGCAUUGUAACAGAGAGCGCUAGGUCUUCAAGCCUACUUACUAAUGGGGACGAUCCGUCGGAUGUUAGUUUUGCCUCCUUUUCUCUUUGUUUGAUAAUCAUCAACUCGAAUGCAUCACCCUUGCACUUCCCAUCUCCUAUUUGCAUUAAUCUUGCUUUGAAUACAGACUCAACAGCCUUUGCCAGCUCGUAUCCAGGAUUGUCGUAUGCGAACACACAAUGCUCUUGCCACUGGAGCAGCCUCAAGAGGCUUUGAGUAGUCGUCUCGGGGAUAGAGUGAUCGAUAGAGAUCAGGAGGAAUGAAUUUCUCCUGUAGAUUGCCAUAGAUCUAAUACAUAAGAAAUCGUCCCAAGAGGUGGAGUAAAGAACUCCCUUCACCUUGAAGAGUACCACUUCUCCCUCAACCCAAAUCUUUGACAGCCAGUCAUGGUGAAUGGUACAUGUAAUAGAGCCCUGUCUAUUUUGCAGCCCCUGAUCUUGUCUAUGAUGAAUUAUCAUCUCAAGUAGCUGAUCAGCUCUCGCGUACCUAGCGUAGAUCUCAAGAGGUGUUGCCCUAGGGGGGUUCCGGGAAGAUAGUGGUGUGACAGUAGAGUCAAGCUGAGUUCCAAUUCCAUCUCGAAUAGUAUCGUAGAGACGUCCAGCCUCAAGGACAGCCGUCUUAGGAUCAGAGAAUGACGGAUUUUUGGACUCGGUAAGAGCAUUCAAGAUAAGGAACCGCGUUUGCACAUCUAGGUCUUUGAUUGGAUCCCUCUCUUUUAGCACCCGACACGCUAAAGAGACACACGAUCUGUCGAGAUGGCUGAGCCGACCUACACCGCGCUGCUGGCACAUCGCCUUUAUGAGAGCUAGCUCUAUAAGCCUAUUGCUAGAAGAUUCAUCUAGCGAAUACGUCCUGCUGAUAGGAACGGACAAACUUUUCGAGAGAGACAGACUAGACAGGAUGUCUCUCUUAAUAGUAAUGAGUAUGGGGCUAUCCAAUAUGUCCCCAAAUGUCUUGACUCGAUUCAUGUAAACUGUGUCGAAAUCAUCGUCAAAAUCCAUGAUCGUGAUCUUCGUUCUGGUCUUAACUGGAUGUUUGUUCUGAUCUUAACCGGUUUUUCUUAAAUCUGCUCAACUGUCGCUAACAUAGCUGAGGAUAUAAUCUCAUGCUGAAUGAGUUUCACUUUGUCCCGGAGUACAGAAUCUGACUCAUUGAUUGUCCUAACAACCUCAAAUCGAAUCUCUCUCCUCUUUUCUCGCGCUAGAUUGCCGAUGCUCAAACUUGCUGUUAAGACUGCGGCCUCACAGGAUAAAUGCUCACUUGCAGAUGCUACUGCAAUAUAAGUAGCAAGAGUCGACGGUGCAACUGUUAAGCCGGCCAGCCCUAUAUCAACAACUCUACUUAGAUCAGACAAGCCGAUAUGAACUGGUAACUGCUCGCAGAAUUCGUGGAUUGCGGUCAUCUCUAUUGAGAAUGGCGCAAUCACUUCAACUGGGAGUCGAAGCAUAGCGACACGAGCAUCUAAUGAGGUGCUGCGAGAAAUUAGGAUUAUCGUCCGAAGAGCUGCCACGCGAGUGAGAAGAGUAAUUUCUGGAUGACUAGACACCACUACCCCGAGUAUUGAAAAUGACCAAACUGGUCCCGAGAAUGACGGUGGCAACCUUAGCCUAGACAUUAUGUUCUGUUCUUGCUAGGUUUUUCUUAAAUCACCAAAUUAAGCGAACGUGGAUAGAUGACCAAUGAACUGAAUAUGUUGAGCUGAUAGAGAAAAAUGGAGAACAGUGAUCAUUGCAGAGCAGCUUCUUGACGCCCACCGACACCUGAGAUCACAGGUCGACCUUUGGAUUGAAGGUAAGCAUUGGCUCUUGCUAGAACGUCUGAGAAAGGACUAGUAUCAAUGAAGUUCCCGGCGGUAGCUUCGGUCUGUUUCUUAAAGCAGACUGCUAGUGCAAGAAGAGCUCCUCGAGUGGUUGAGGAGAAGAGACGGUGCUGUGACCCAUACCUGACCUUAACGAAUCCUCUCAAGUUCUCAUCUUCCUCCAUGAAAGCUCCCAUCGCGUCAAUGAAGCUAUUGAUAGCUGGGGAGAGCUCGGCGAAACCUUGCAACUCGGGGUAGGCGGAUAACAACUCGGCGAUAAACAUCGGAGCCCCCAACCCGUAUCCAUCUGUUAAGCGGAAUGUCAUCAAGAAGGCAUCGAGCUCGCGAGUUGUGCUCCCCUGGUUAACGGCAACUGCCCAUCGGGUUGCCAGAUGCUUGCGAAUUGACUUGUAUGCAUUGAAAGAUCUCUCAAUUCCUUUGAGUGUCGCGAAGACUGGAAGGAGACUCUCUCUGAAAACUGGGUAAUCAUCAGGUAGGAUCAGCGCAUUCUUGAUUGCGAGCACUCGAUUGUGGCGGAACGCAGUGAUGUUCUCGAGCGAUACAUUCUUCCCGAUUGGAAACAUCUCAAUUCCCCAUCGUGCAGCAACCUCCACGAUACCAACUCCCCACAAUGUGGCAUCUUCUUCGAGAUCAACUACUUUCAAGUCGAUAACGUUCUCUGCAGACACAGAGCUACCGGAGGCAACAGCUUCGCCUGACAUUCUGUUCAGGACUCCAGGUCCAUCUACUUGGACCACAAUCCGAUUGAAUUGACCAGCCUGAUCUCGCUGGAGUCGAUCAACCGUACCUGGGAAGAUCAAUUCGGUCCCAGUGAGGACAUAGGAAAUCAUAUCCGGUGCUGAUUUCAUUUCUGUCGAUUGGUAAGCUUCAAUCAACAGAAUCGCGACGAAGGCCCGAAGCUUAGCUUCAUCUUUUGGACCAACUUCUCCAAAGUUGACCUUAAUGUAGUUUAUGGUUUCUGUGAGGGACGCAACCUCAACCGCUCUGUUAGAGGAUCUGAAGAUCGAUGCUGAGGCCAAGCCGAGAACUUGGGCGCGAGAGUGAGUAGCCAUGUCGAGGGCUUGUAGGAUGAUUCGAAAUGAGGGAGUACUGAUCGAGAUUAUAGAAGUGUGGUGAUAGAUGUUGUCUGUUCUUGCUCGGUUUUUCUUAAAUCAAUGACAACUCAAUUUAACUAAAUCUGGUCUCUUCGUGUCUCUUACUCGAAUGAUAAUUUCAGCUUUCUGGCUCCUUUAGAGGAUGCUUUCAUCUUGGGUGAUUGGAUGGGAGCUCUCUCAGCAGCCUUGGUGACUGAAUCUUGAGCCUUGAUCAUCUCCUUCUCAAUCAUCGCAGCUUGAACAUCAGGAGACGGAUUAUCACGAACGAUCUUCGCAGCUUGAUUAAGGACUGUUAAUGCGUCAGAAUCAAUGGAUUUGACUCGAGAGCUAACAUCUUCCGCUACAACCUUGAGUGAGUUAAUGGUGGCAUUAAGGCUUGCAAUUUUGGCGUCUCCGGCAUCCAGACGUCGUAGUCCCGGCCAGCUGCUCGUCAGUGAUUUUAUGAGCUGUGGGAUUCAAAAUCUCUCGAUUAGCGCUUCUAAUUCCCAGCACGCCGAUAGCUCUCUUAGGAGCAUCAUUGCUUCCUGCUGGUGUAGAGGUGAAUUCUACCUCGGACUCCUCAGCCGCUAACUGAGAGCUCUCCUUUUUGAUGAUAGGGGUUUCCUGGAAAUCAUCUUCUGAUUCGCUCCCUGAGAUGUCGGGCCUGCUCUGAGCGUCGAUUCGAGCUUGUGGGGUUCUAGUUGGUGGUGCGCUGCCGGACACUACUGGCUUUGGAGCUGAGCUUGGAGUUGUGGGGAGUUCUUCGUCAACUGAGUCUGCCCAAGAUGCCUUCGAUAGGGAUGACAUCAUCCGAGAGACUCCCAUCGAGAAGGAUCUCCCGCUCACGUUGCUGUCCAAGGACGCAGCGGCUUCUUCGCUAAUCAAUUGUGGGUUCUGCUUAUUCGCCAUUGAGGACACGACAAGGACGAUAUCAGUUAGGAGUAGGUCGAUUGACAGAUUUGCUUUGUCUGUU  >FirsU_Contig7  CCGAUCCGAAAGGGGGUGCAGAGCGGAUUCUAGAUUAAGCAGACUUUAUUGCGACCAAGCNUGGCCUUCAGGCUGGUCUUGGUUGCAAGUCCAAUCGCCCAACAUAAAUCAGGCAGACACCNUCGCGCCGAGUUUUUCUUAAAUCCUGAUCCGAUUAACUAGGUUGUGUAAUCUUGAAUGUUNUCUGCUAUGGAAGAUAAAUGAGACUCUAUGAGACAAUCAUUAGAGGGAGGUGAGAUAGCANGCAUCCUCUAGCAGUUGUAUCUGCCUUAAGAUAACAUCAAGCUGUUUUGACCCUGCUCUCNUCGGUCGAUCUUCUUUUGGCUUCCAUCAGCUGAUCACUAAGCUGGCCUACGAGAAUCUCCNGCCAUUUCGAUAGCUCUGAUAGCCUGUGCAGAUGUAGUGAGCCAGUCUGCUUCAUACGGANAGAGAUGGAGGUUCUUGAGGGACUCUGAGAGAGUGGAGUUCUGCCAUCGUCUUGAUCUUANAUAAGUAGAGAUUGUGUUUUCGCUUGAACAGUUGAGUGGAUAAACGCGAUCUUGGCUUUCNUCGAGACUUUCAAGUGCACCUCCCUCGAUGAGUGCUUUUAGUUUACUCUCUGCUAUAGACNAAUUUUUCCUUGUAAGAGGGUGACAUUCUGAUCUUGUGUGGUUUUUCUUAAAUAAUUACANGCUUCAGAAACCAUCCAUGCCUGUCUCUCGUCAUUAAUCGAGGCAGGAUUCGAGAUGACANUAUUGAAGAUAGAUGCUUGACGAACAGCUGGAACCGGAUGUCCCUGUAUCUCGAGCGGGGNCAGUGCUUUGAGCAAGAUCAGAUAGUCCCUCUAUCACAUCUUCAGUCGAGAGAGGACCGUNGUUCAAACAUUAGAUUGAGUUUGCCUAACAAGUAAAGUGCUAGAACUAUAUCCUCUGUGANCAUCGCGCCCUCGUCUAUGACUGCUUCUAGACAUACUCCAAUCAACAAACAUCAAGAGGUNAAUCUUUUGCCACUCCCAAGCUUGCACCAGGAGGCACUCGUCCUUUUAGUAGUGUAGUUCNGAUUCAGUUCGAACUGAUUUGGGUAGAAGGGAGAAUCAGGUGGAGCAUCGAGAGGAUCCANAGUCAGCAAAGAUUGGAUCACACGUGUCUGUUGGGCGGCUAACGGACUGGCACCCUGAUGNUGUGUGCGACUACCCAGUACUCCGUCUUAUUGAUAGUAGACCUGAGUAUACAGAAUGUGGNAGAACGUUCCUCUACACUCUUGGAAGAACACUGCGGCUUCAGGUUCAGUCAACUCUCGUCNUAGUGACUAUGACUGUCUUUAUCAUGCUCUUCAGAACAGGGACAAUGUCCUCCCAUAUCANUUCGUCUCCCUGGCUGAAUGUCAAGACACACCACACUUGGUUGUUCGGACAGAACGUCAGNAGUGGACUCUACUAUCGAACCAGUCACCUCCGUAGUUAAAAACACUCUUAGCCCAUCUCCNACGGGAGAGUUGGAGAAAGGAUCGGAAUCUCUGGAGGAAGAUACCCAGUCCCGAGCGAGCNUCACGGACGGAAGGUCUGAUAACAAAUCUACUCCAACGAUGGAUUUUGCUCCUAAUGCAANACGCGUCCCUGGCCAUAGCACCAUUGCCCACUCCCACGCAUAAGACUGGACCUUGUCUUANUUGAGCUCCGAACUGUAGAAAGAACCCUGUAGCUCAACCGCGAGAUUGACGAUCCUCCGANUGCACCACCCAAGAUUUCUACUCACCAGUCUCUGGCUAUCGGUUUUCCUGUCUAGUGCAANGCAGAGGUGAUGAGAGCGGACUAGAUAUCGGGAAUUCGGCAGCUGUUCCCAUCUGCCAUGNCAAUAUCCGGAGGGGGGAGCGGAGGAGUGGUGACCCUUUGAGAUGCAACUGAUCGUGGGCNCUGUCCUAAGAUACCUCUUGCUAUCCUCAAAGCUCAUAGCAGUUCUCAAAAGCACCCUGCNCUGCUAGGACUUCUGAAGCGAGAACUGAGGAUUCCUCAAACUCUGGGUCCGCAAGGAGAUNGCAGGAUGGUAAUGUGGUGGACUACCCUUUCAGACUCCUUUGUGAGGCCUCUUAAUCCGUNUGAGAUGCCUUCUAUAUAUUCUCCUGCAAUCAUCCACUGAUCCUCGGAUGCCCAGCAGAANCGAUUGAGCAGGCAACGUAGCAUCCCAGAACUCUUGAAGGAGUAGCUCGUUCAGCGUAUGNAUGAUAAUAUCAUUCUAGAGUUACCGAGAUUUCGGAUAUCUCUGUAGCACCGAUGGGCCUNCAUCCCUGAUGUAUGAUGAUAAUCUUGACUUCAAGACGACUCCUCCGUGCUUUCCCGGAGNCAAUUAGCACUCCUGAUGCCUGAACCCACGAUUGUUGGCGGACUUGAGGGUUGUUAGCAANACCGAGCUAGAGGUAACGCUGCAACUCCAGACAGAGCGUCAAUAUAAGCAGACAAGAUCANCUCUGUCCAUUUUCCCAGUCUCGGAUAGAGACAUCUCUAUAGACCGCAGAAAUACAGCUCNUUCCUGCUGCGAUCAUCAUCGCUUGCAAUCCGACUCCUAUUAGACUACCGACAUCUAGAGNACAACCCUGACGGAGCAUCUUUAGCAUCAAUCGCAGCUUCAGACAAGACAGGAUUCUCGANGCUGGUCUAGAAAGUGGUGGAUCAUCAGCUUAAUUCCAAUUUCGGUUGAACACGCGGGCGNGAGCUUUCCACAAUGGGGCAAUGGGUGCAGGCAUCUCCUCGCUUAGAACUUGGUAAAGGANUGUUACUCGCACAAACUAGAGGAUUCUCUUGGAGGUUCCGUGCUAGAGAUAGUUCAGGAGNCCUUUCCUUCAGCUGAAACUGGAAUACUAGAGAUAAGCAUAUCCUGGGAUAUAUCAUAGUNGAAGACGCAGCGUUGGAUAGCCACUGAAUGAUUCGGGGCUAGCCCUAAUCAGUUGAAGUCNCAAAGAACGAGUGCUCUUGAAAACAGAACGAGUAAUCUUUAACCCCACCACUGAGACUACNUCAUUGAAUCCGUCUGAACGGUGGAGUGUUGAGUGAGUGAGAUAGGUCCGAGCCAUGCAANACGCUUUCUCCUCAACUGUACUAUCCCACCGAUGUGCAGCUACUCCGCCUACUACUGUAGNGCAUGAACAUCUCUAAAUCGCGCUCGUCAACCGUAGUUCUCUGCUUUAAAACUCCUCUUANUUGCACGUUUGAGCUCUCCACUGGCUGUCAACUGAGACUCCAUCAUCAGCAGCCUUUUAGNCAGCUAGGGCUGCUUUCUGGGUCCCAAGAACUUCGACACCAGUCACACGUCUGUGCUCCCNAGGUCUUAUCUCCCCACCUUCCUGGGUGCGGCCCUUUGGUGUUCAUCAUUUCAGGCCAUGNGGACUAAGCUCGUAAACUCGAUAUAAUCAGAACUCUGAAUCUUCAUCCCCGGGUCAGCUGNUACACUCGAGAGGGUGAAGUACCGUGACACCUUCUAACUUGUUUGAUCCGUCGGGAAACCNACCUCUCACGAUACUUUACUGCUCUGGUGAAGAUGCUUCUGGAGGGAAGUACACUAGUCANUCCCAGCUACUGAUGAUUGACCCAUAAACGUUCUCAGUCGAGUCACUCUCUUAUAAUCGGNCGUAGAUUACUGCGUGCCCGUAAUUAAUGGGAGCAUUAUUGCGGACUAGCUGCUGGAUCGNUCCUCGUACCUGUGAACUUCUUGAACACUUUCUUUUUGACACCAAUCACCGAGAGUUCAANCCAGAUCUGCCAUCAACAAUGGGUAAAAGGGCUUCAACUUAGUAAGAAAGGAUGCUAAGGNAUCGCCCGAACUCAUCUACAGAUGUGGACAUAAUUUGCUUGAGAUCGAUAUUCUGUGUUANUAGAUGGGACUACCGUCUUGAUAGCUUGAUCAACCUGCACAUCAGCUCCAACACUCGGCANGGAUCGGAACAGAGAAUGGAUCUUCCAGUAGAGUUGCAGGAUCUGGAUCAGGUUUCCAUGNGUAAUGGUGUUUCUAAAUACCCUAGGUACUGCUGCGCAGUUCGACUUACAUGCGAGAGGCNAUACUAAAGCUGCUAUGGAAGAGCUAAGCCGAUCUGAGCACCCCCCGAAACAGAAAUCAGNUAGGUAUUUGGAUAGGCAGGCCACCCAAGUCGCUAGGAAUUGUGCAGGCUUGAUCAAGGUNCGUGCUGACUCCAGGUGUAAUUGAGAAGUUGUCUACUCGGCAGUGUCUUAGACGCUCUAGNGUAGGUAUUCUCUGAAUAAGACUAAAAAGUACAGAUAGCCGAGAGAUGGAUCAGUGAACGNCAUCAGCAGAAGCCCGGCAGCCUGAACUGAUAGCUGAUAACCCAACUACAAGGCCUGGGANUGUUAGAUGUUCCGACUGGUGCUACUGUAGCUGCAUGCUUAAGGGUGGUAGGGAUCAAGANCUCCAUCUUGCCAACAGAUCUUGCUGUAAGUCACGGUCGACCGAGAUGUUAGGCACUCGUNCUGGUUUAAUUACUUGAUUGAGACGAGCGAAUCUCCUACAGAGUUCUUCAGUGCAUUGUGNCUGAUACUUCCAAGGCCUGUACCAUCUUGUCCUUACCAUGAUCUCGUGUGUAAGAUAUUUNCCAAGAUCUGAUUAUCACCUUGCCCCAAGAGCUUGUAAGCAACAACGUUCAGAUCACGCANUCACUGCUUUGAUUUCAGCCUGUGUGCAAGCUGUCCAUUGAGCUUGCAUAAUCCCCUCGANAACCACCUAGGUGAUUCCUCCACACGUAAUCACUUUCCGGCCACUCUCGAGGAAAACUACNCAGGCACUACACCAUCUGGCGUUUCCGUGGACACUCUAACUACAACUGUGGAGUUCUCAANAUAUCUCGUGACCUUUUGAGAAUGAUCCAGGCAAUCCGAAGAUAGAAUCGGCAAUAUUGCNUAACCGGAUCUACCACUACUCCUCUCCAUAGGAGAUUCCAGCGAGAGAGAUCAACCUCAANUAAAGACGGUGUCUAUAUCUGGAUUCGACUGUCGCGCUGCCAUUCCCUCUAAGUGCUGCANUUGUCCCCUUUCGCCCUUUCGUCAUCGAUUGAUCUGGCAGAUAGUCUUUCAUCAACCUCUNUGAAGUUCUUCUCUGCAAGUGUCAGAAAUACGCGGACAGAAAAUUCGAGCAUGACAAAGANGCCGCGCUUCCAGUUUAAAUUCUCUCUCCUUCGGGUAUAGGGACAUAGAUAUAUCGGCCANGAGACAGGUUCAGAGUCCUUAUUUGCUCACAGAUAGCCUUGGGAUCAAAUUCUUUCAUGCNUGAGGAUCCGUAUGAUAAGCUUCUUUGACCUCUCGUUUGGUAUGUGAGCCAAAGACGGAGNCGUCCUUCUUGCAGCUGUCCCAUGCCUUCCAGGCAUCUUGAGCAUCCAGCCCAGUCGAUUNUGUCAUCCAUCAUGUCUAAGUAGUCAAUGUUGUAAUCAAAAUCAAGAAGUUUUGGCCAUGNCCGAAUAGUACCAAUCUUGGUAAUCAUAGUCUGACGGACCAAACACUAGCACUCUUCGCUNCGUGGAGUUUCUGAAGUUUGGUCUUCCUUCCAGGAGAGUGAAUCAAAGCCGGCCAUCGAGNCAUGCUUUUUUAUGUAACCUUUCAAAAGCAUGUCGCAAAACUCUGCUCUCAUUCGCAUCGNUUUCUCCGAAACCAGGGUUUCCGCGAGCUGUACCGUGGCUUCUUGCCGAUUGAGAUGCGANUUUUCGGAUCUAUGAGAGGGUGGCCAGAGAACUUUAAGCAGCCAAACAGUUCAGCUGCCUNCUCUGGGUAUAGUAACAGAUAGCGCCAAACUCUCUAAUUGACUCACAAGUGGUGAUGACUNUAUCGGGUGUCAGUUUAGCUUCUUUCUCUCUCUGCUUGAUGAUCAUCAAUUCGAACGCAUNCUCCCUUGCAUCGUCCGUCACCCAACUGCAUUAGUCGUGCUUNUGAAUACCGAUUCAACAGNCUUUUGCCAACUCGUACCCCGGGUUACCAUAUGCGAAGAUACNAGUGUUCUUGCCACUGGANGCAAUCGCAGAAGACUUUGGGUAGUCGUCUCGGGGAUCGAGUNGAUCCAUGGAGAUCAAUANAGAACGAGUUUCUUCUAUAAAUGGCCAUCGACCUGAUGCACAACGAAAUCAUCCCAAGAAGNUAGAGUACAAUGAUCCCUUAACCUUGAAGACAACUACUUCUCCCUCAACCCAGAUCUUUGNAUAGCCAAUCGUGAUGAAUAAUGCACGUUAUAGAGCCCUGCUUGUUCUGCAAUCCUUGAUNCUUGUCUGUGAUGGAUGAUCAUUUCGAGCAGCUGAUCUGCUCUCGCGUACCGAGCGUAGANUCUCAAGAGGGAUAGGUCUCGGAGGUUUCCGGGAAGAGAGUGGUGUUACAGAAGAGUUGANGCUGAGUGCCAAUACCAUCACGUAUUGUGUCGUACAAGCGUCCGGCCUCAUAAACAGCUGNUCUUCGGGUCAGUGAACGAUGGGUUUUUAGAUUCAGUGAGAGCAUCCAGUAGAAGGAACCNGUGUUCUAACGUCUAAAUCCAGUAUUUGAUCUCUCUCUCCAGUCAAUCGACAAGCUAGAGNAAGCACACGAUCGGUCUAAGUGGCUGAGUCGCUGGACACCGCGCUUUUGACAUAAAGCCUNUCACUAGGGCUAACUCGAUUAGUCUGCUGCUGGAUGAUUCGUUCUGAGAGUAUGUCUUUGNCUAUAGAGAUUGAUAAACUUUUUGAUAAAGAGAGACUAGAUAGGAUAUCUCUCUUAAUCGNUUACGAGUAUGGGACUGUCUAAUAUGUCGCCGAAUGUUUUUACUCUGUUCAUAUAGACCGNUAUCGUAGUCAUCAUCGAAAUCCAUGAUGAUGAAGUGUUUGCUCUGAUCUUGCCCGGUUUNUUCUUAAAUCUAAAUCUAAUCAACCGUUGAUAUCAUAGUUGAAGAGAUAAUAUCGUUCUGNGAUGAGCUUUACCUUAUCGCGGAGUGUUGUAUCUAGCUCAUUUAGGGUCCUGACGAUCUCNAAAUCGGAUUUCCCUCCGCCUAACUCUCGGUAAAUCUCCUAUGCUUAAACUAGCCGUCAGNGACUUUAGCUUCACAAGACAAGUGCUCACUAGCUGACGCCACUGCCACGUAAGUCGCCAGNGGUUGAUGGAGCGACGGUUAGUCCAGCCAGACUAAUAUCGACUACCCUAUUUAAAUCUGANCAAGCUGAUGUGAAUAGGUAGCUGCUCACAGAACUCGUGUAUUGCUGUCAUCUCUAUCGANAAAUGGUGCUAUAACUUCAACUGGGAGUCGGAGUAGAGCUACACGAGCGUCUAAAGAAGUNGCUCCUAGAGAUGAGGAUGAUCGUCCGAAGAGCAGCCAAGCGAGUUAGGAGAGAAAUCUCNGGGAUGGCUCGACACUACUACCCCCAGUAUCGAAAAUGUCCAGACUGGCCCUGAAAAUGANUGGUGGUAAUCUUAAUCUAGACAUUGUAUUCUGUUCUUGCACGGUUUUUCUUAAAUCUUUNAAGGCAGUGGGCAAGCGAGUGUAGAUAAGUAGAGAGCGACAGUGAAUGGUACGAUUGGAANAAGUAGAAGAUCGUUAAUUAUUGAAGAGCAGCUUCUUGACGUCCUCCCACGCCAGAGAUCNACAGGUCGACCUUUAGAUUGAAGGUAGGCGUUGGCUCUAGCAAGAACAUCUGAGAAGGGGNCUUGUGUCAAUGAAGUUGCCAGCGGUUGCCUCAGUUUGCUUCUUGAAGCAGACUGCCAAANGCGAGGAGUGCUCCUCUAGUAGUGGAUGAGAAAAGGCGAUGUUGCGAUCCAUAUCUGACCNUUUACAAAUCCUCUCAAAUUUUCGUCCUCUUCCAUGAAAGCCCCCAUUGCAUCAAUGAAGNCUGUUGAUAGCUGGGGACAAUUCAGCAAACCCCUGCAGCUCUGGGUAGGCAGACAACAAUNUCAGCAAUGAACAUUGGAGCUCCCAAGCCGUACCCGUCCGUCAAGCGAAACGUCAUCAAGNAAAGCAUCAAGCUCACGAGUUGUGCUUCCUUGAUUAACAGCAACAGCCCAACGAGUGGCCNAAGUGCUUCCGAAUAGAUUUAUACGCAUUAAAUGAUCUUUCAAUUCCUUUUAGUGUUGCANAACACAGGAAGGAGGCUCUCUCUGAAAACUGGGUAGUCAUCAGGGAGGAUCAUUGCGUUCNUUGAUCGCAAGCACUCGAUUGUGGCGGAAUGCUGUGAUGUUUUCCAGCGACACGUUCUUGNCCAAUAGGAAACAUCUCGAUUCCCCAUCGUGCUGCGACCUCGACAAUACCUACUCCCCAGNAGUGUCGCGUCUUCCUCCAGAUCAACCACUUUCAAGUCAAUUACAUUCUCAGUAGAUACCNGAUGUCUCCACAGCUGCAGCCUCCUCUGUCAUCCUUGCGAUGAUUCCUGGUCCAUCUACUNUGAACCACGAUUCGAUUGAAUUGACCAGCUUGGUCUCUUUGAAGGCGAUCAACUGUCCCCNGGGAAGAUCAACUCGGUCCCUGUAAGAACAUACGAGAUCAUGUCCGGUGCCGACUUCAUCNUCUGUCGAUUGGUAUGCUUCGAUGAGUAGAAUCGCUACAAAGGCUCGAAGUUUCGCCUCGNUCUUUAGGACCGACCUCGCCAAAGUUCACCUUGAUAUAGUUGAUAGUCUCGGUGAGAGAUNGCAACUUCGACAGCUUUGUUGGAGGAGCGAAAGAUGGAUGCAGAGGCCAAGCUGAGAACUNUGGGCACGAGAGUGAGUUGCCAUUAUGAAACUUAGUAGACUAGCGUACGGUGAUUUAGUGNCUGAUGCAAGUGAUGAAUGUAUGAGGAUAGAUUUUGUCUGUUCUUACCCGGUUUUUCUUANAAUCAAUAACAAUUCGGUUAGGUUGUUCGUAAGAUCUCACUAAUUAACUUCAUUCAAAUGNACAACUUAAGCUUUCUAGCUCCCUUUGCUGCUGCCUUCAAUUUUGGAGACUGGAUAGGGGNCCCGCUCUGCUGUCUUAGUAAUAGAGUCCUGGGCUUUCUCGAUCUCAUGCUCUAUUUUAGNAGGCCGGGAUAUCCGGAGAUGGAUUGUCACGGACUAUCUUGGCAGCCUGGUUGAGGACUGNCUAGUGCGUCGGAAUCGAUAGAUUUCACUCUAGAACUGACAUCUUCAGCCACAACCUUAANGCGAGUUAAUAGUGGCGUUGAGAUUCGCGAUCUUGGCAUCCCCUGCAUCAAGACGCUGGANGAAGAAUCUUGAUGACAUUAUCUUGAGCCUCUACAAUUUGUUGAAGCUUAUCGAGUGCCANCUUUCAUCGUAGUUCCUGCUAGUUGUUCGUCCGUGAUUUUCUGAGCAGUAGGAUUUAAGANUCUCACGCUGAAGGCUUCUAGCACCAAGAACACCGAUUGCUCUCUUUGGAGCAUUGUCUCNUCUCGGCAGGUGUUGUAGAGAACUCUACCUNCAGAUUCCUCAGCUGCUAGCUGGGAACUCUNCCUUCUUGACUGCAGGGGAUUCUUGGAAAUNCAUCAUCCGAAUCGCUCUCAGAAAUAUCAGNAUCGACUCUGAGCAUCGACACGAGCUGGUGAAUGUGCCAGCAGGGGGUGCACUGCCGACGGCCGCUAGCUUAGAAACUGGAUUCGGAGUAANUGGGAAUUUCUUCACUCACAGAAUCCGCCCAGGAAGCCUUCGCGAGCGACGACAUCAUUC  >FirsU_Contig206  CAGACAAGACAGGAUUCUCGAGUUGAUCUAGGAAGUGGUGGAUCAUCAGCUUGAUUCCAANUUUCGGUUGAGCACGCAGGCGGAGCUUUCCACAAUGGGGCAAUGGGUGCAGGCAUCUCCUNCGCUUAGAACUUGGUAANAGGAUGUUACUCGCACAGACUAGAGGAUUCUCUUGAAGGUUUCGUGCUAGAGAUAGUUCANGGAGCCUUUCCUUCAGCUGAAACUGGAACACUAGAGAUGAGCAUAUCCUGGGAUAUAUCANUAGUGAAGACGCAGCGUUGG  >Ss-AA_clean.1_(paired)_contig_2473  GUAAUCUUGAAUGUUUCUGCUAUGGAAGAUAAAGGAGACUCUAGGAGACAAUCAUUAGAGGGAGGUGAGAUAGCAGCAUCCUCUAGCAGUUGUAUCUGCCUCAAGAUAGCAUCAAGCUGUUUUGACCCUGCUCUCUCGGUCGAUUUUCUUUUGGCUUCUAUCAGCUGGUCACUAAGCUGGCCUACGAGGAUCUCCGCUACUUCGAUAGCUCUGAUAGCCUGUGCAGAUGUAGUGAGCCAGUCUGCUUCAUACGGAAGAGAUGGAGGUUCUUGAGGGACUCUGAGAGAAUGAAGCUCUGCCAUCGUCUUGAUCCUUACAAGUAGGGAUUGUGUUUUCGCUUGAACAGUUGAGUGGAUAAACGCGAACUUGGCUUUCUCGAGACUUUCCAGUGCGCCUCCCUCGAUGAGUGCUUUUAGUUUACUUUCUG  >Ss-AA_clean.1_(paired)_contig_1376  AUGAGGCUUUGGCCGUCUACCUCGUCCAGCCCUGCUGGGUGGAUACAGCAUACCUAAUUUUGAAACACCAACUGGAAUCUUAGAACAAAGUAUAAGAUAAAAACGGCCUUUAUCAUAUUGAGACUUGUAUAGUCAUAACUAGUACGGUGAUUUUGGGUGAAUGUUAAAAUAAUAAGAUGACUCUAUGGCGUUAUUAAGUGUGUCUGGUAUUCUUAAGUAAUGACAUCAUUCCAGAGUACUUACUCUAAACGGGGUUUUCUUAAAUGUAACUUGACUUAAAUCUAGAUAACACUUACAUAUCGGAAUAUAAUUGGACACAUUAAAUGAGGAACAAAGAAGGACUUCAACAGCUAUAAAUAAAUUUAACGUUAGGAACCUAUUUAUCCUAGCGAUGAUGAGAAAACGAACUUGGUUUUGAUCUUGGGGAUACUCUGCCUAGGGUGGUGGAUGAAGCACUAGAUACUUGUGAAACAGCAGAUUGUUUUCGUGCUUUUUCUUCUCGACUGAAGGGGUCUUUUGAUAGAUCGAUAGAAGGUUUCACGGGAAUUCUACCUUUUGAAUGCCGUGCUUUAUCUAAGGUUGAUACUCUAGUUUUAUUGUCAUCAACUAUGCUUUGCGAUCUACUGGAAGGUCUACUGCUUGAAUGUUUUUCAUUAUACUUUCUAUACUUGCUAGACAUGUCGUAUGGCUUUUUGAGUGCACUCGGGCAGUCAGGGUAGCGCUUCUUAGCCAUGUAAUGAGCCUCAUGAACAGUCGGUUCGUGUUUUGAUGCAGGUACUAUCCACGACUCCGAUGCUUUCGACCUGUGACCUAAUGGAUCAGAGUACUUGUUACUCUUUUUCUUGAAAUGGUGAGUAGCAGCGUUAACCGCCUUUGACGAUGGUACAGUGUCAGGAUUAAUUGAUAGUGUCGUCGCAGAUUCGUCAUUUGCUAGUUUAGUUGCAUCGUAACCAUAAAAGUUCAAGUCUUCAUCUGAUUGAGGAACAACAUGUUCUCCGGAAGACAAUUGUUUGACAUUAUAGUCUAACCUUUGAUUGAUAUAAGUCAUGUCAUUUCUCAACUCUUUAUUUUCUUUGAUUAGUAGUUCAUUCUCCGUAGACAUUUUGUCCAUACUAAACUUAAUUACACUGAGUUGUUCUCUCUCAGUCGUCUGCAUCUCUACAUUCAUUGCUAUCUUAUUGAAGUCUGCUGCUAGAUAUGACAGAUCGAUUAAUCUUUCUUCUAAAUCGAUGACUUCUAGUCUAGCAUCCAUUAGUUCAACCGCCAAUCUAUCUUUUUCUUGCUGCAGGGCUCGCACUUGUUUCUCCAUCAUCAUCAACUUGGUUAAAACGGAUAUUCCCAUUUCGAAAAAAGUGAUAGAAUAACUAUCAGUUCACUUUGUUUUAUUAAAUGUCUGGUCCUAAGUUUUCUUAAAUUAACUUGGCUCAGAAUAAGUGACCUAGCAAUUGGGGAAGAGUAGUACUUAACUCAACUCGGAAUUUGUGAGUCAGCUUACUGACGAUUUUGUGAUUGUUUAGUUGUCGUAAGAUGUAUUUUUGAGAAAACAACUUCGAAACCUCUGCCUUGAGCUCCGGAACACUCAAGUCUUGAUAUUCUAAACAGAGCUGGAGCUGGAGAAUGCUCGAAGUAAGUUUGGCAAAAGAAUAUUUGUCUAGUAUUUUAUGCUUACUACUUUUUGCAGCUACGAAAGUUUCUAGGAGCAACUGCAGUGAUCCUUCUACAGUAGGUCUAUUUGGCCACGGAUUACAAAUCUCAGCUAGUUUUUCCUCCAUAGUGGAGAACACAAUUUCUGAGUGACGGUGUAUAUCAUGUAUAACAUGAUGAUUGGAUCCGAAACUUCCCUUGCUUAACACAAUUCCUCUAACCGGCUUCAGUAUAAAAUAGACGUGUUUGUAGUCAGUAAUGCUUUCAGAGGACAAAGCGAGUAUCUCGUAUCCGGACACGCCUAAGUCCUCAUAGAUGGUUUGGAUUUCUGACUCUCUCAAAAUCAGUUUGAUUAUUACAAGUGUUUCAUCAAAUAACCCUGCAAUUGGAUCAAGCAGGGAUAGGUUAAACCUCUCCACUCCCUUUUCUAUAUCUAUGAUUAUUGUAUCAUAAUUGUUCUGUAAAACAGAUGCAGACACUCUCGGAUCAAGCCAGUCACCCGUCGAGUUGUAAGUUUCAGCUGUUAUUGCAAUCCGAUGAUUGAGUCUAGGAGACUGAAUACAUGCAGGAGGCAACCAAGUUGAAUAUCGGUGAGCAAUGUUUGGUAUAGACGAUUGCAAGUCCAGUAAUUCUAUCCUAGAAUUGGUCAAGUCCAACAAUCCUCUAGCAAAUCCUCCUUGUCCAGAUCCCACAACUAGAGUCUUUCUAAAGUGACUCGGGUAAUGAUGGAGCGCACAGUGAAUAACAUCUAGAGAUCGAGAGUAAACUCCAGUGUAUCUGUUUGUAGAAAAUUUCGCCAUAUUCCGCUCAAAUUUUUUCAGCCAACUAUCUUCUUCAGUUACUAAGGGUAGCGUGCGGACACCAGCUUCGACUGCUUUCCGGUUGACUCGAGACACAAAUCUGGUGGAGCAUGUAAGGCUUAGAGGAGGAAUUGGUAUGUGUUCCUGCUUUAUACAGAAUUUUCUUGCAUUUCUAAGUGCCUCAGAGUAUGUUGCUUGCAACAGGGCUACCUUUGUCUCUGAGAGUCCUCGAACAUACUUGUCCUUGAAGAAAUGAUCAAACUCGGAAACAUAUAGACUAAUCUUCUCUGCUUGGGUAAUCAGUCGACUGGCUUUCUUCCUGAUUUUAUCCACUACUAUUGACAUAGAUCCCACUG  >Ss-AA_clean.1_(paired)_contig_1375  CAGUGGGAUCUAUGUCAAUAGUGGUGGAUAAAAUCAGGAAGAAAGCCAGUCGACUGAUUACCCAGGCAGAGAAGAUUAGUCUGUAUGUUUCUGAGUUUGAUCAUUUCUUUAAGGACAAGUAUGUUCGAGGAUUAUCAGAGACAAAGGUAGCCCUGUUACAAGCAACAUACUCUGAGGCACUUAGAAAUGCAAGAAAAUUCUGUAUAAAGCAGGAACACAUACCAAUUCCUCCUCUAAGCCUUACUUGCUCUACCAGAUUCGUUUCUCGGGUUAACCGGAAAGCAGUUGAAGCUGGUGUCCGCACACUACCCUUAGUCACUGAAGAAGAUAGUUGGCUGAAAAAAUUUGAGCGGAAUAUGGCGAAGUUUUCUACAAACAGAUACACUGGAGUUUACUCUCGAUCUCUAGAUGUUAUUCACUGUGCGCUCCACCAUUAUCCGAGUCACUUUAGAAAGACUCUAGUAGUUGGAUCUGGACAGGGAGGAUUUGCUAGAGGAUUGUUGGACUUGACCAAUUCUAGAAUAGAAUUACUGGACUUGCAAUCGUCUAUACCCAACAUUGCUCACCGAUACUCGACUUGGUUGCCUCCUGCAUGUAUUCAGUCUCCUAGACUCAAUCACCGGAUUGCAAUAACAGCUGAAACUUACAACUCGACAGGUGACUGGCUUGAUCCUAGAGUAUCUGCAUCUGUUUUACAGAACAAUUAUGAUACAAUAAUCAUAGAUAUAGAAAAGGGAGUGGAGAGGUUUAACCUAUCUCUACUUGAUCCAAUUGCAGGGUUAUUUGAUGAAACACUUGUAAUAAUCAAAUUGAUUCUAAAAGAGUCAGAAAUCCAAACUAUCUAUGAGGACUUAGGUGUGUCCGGAUACGAGAUACUUGCUUUGUCCUCUGAAAGCAUUACUGACUACAAACAUGUCUAUUUUAUACUGAAGCCGGUUAGAGGAAUUGUGUUAAGCAAGGGAAGUUUCGGAUCCAAUCAUCAUGUUAUACAUGAUAUACACCGUCACUCAGAAAUUGUGUUCUCCACUAUGGAGGAAAAACUAGCUGAAAUUUGUAAUCCGUGGCCAAAUAGACCUACGGUAGAAGGAUCACUACAGUUGCUCCUAGAAACUUUUGUAGCCGCGAAAAGUAGUAAACACAAAAUAUUAGAUAAGUAUUCUUUUGCCAAACUUACUUCGAGCAUUCUCCAGCUCCAGCUCUGUUUAGAAUAUCAAGACUUGAGUGUUCCGGAGCUCAAAGCCGAGGUUUCGAAGUUGUUUUCUCAAAAGUACAUCUUACGACAAUUGAACAAUCACAAAAUCGUCAGUAAGUUGACUCACAAGUUCCGAGUUGAGUUAAGUACUACUCUUCCCCAAUUACUAGGUCAUUUAUUCUGAGCCAAGUUAAUUUAAGAAAACUUAGGACCAGACAUUUAAUAAAAACGGAGUAAACUGAUAGUUAUUCUAUCACUUCUUUCGACAUGGGAAUAUCCGUUUUAACCAAGCUGAUGAUGAUGGAGAAACAAGUGCGAGCCUUGCAGCAAGAAAAAGAUAGAUUGGCAGUUGAAUUAAUGGAUGCUAGACUAGAAGUCAUUGAUUUAGAAGAAAGAUUAAUCGAUCUGUCAUAUCUAGCAGCAGACUUCAAUAAGAUAGCAAUGAAUGUAGAGAUGCAGACGACUGAGAGAGAACAACUCAGCGUAAUUAAGUUUAGUAUGGACAAAAUGUCUACGGAGAAUGAAUUACUAAUCAAAGAAAAUAAAGAGUUGAGAAAUGACAUGACUUAUAUCAAUCAAAGAUUAGACUAUAACGUCAAACAAUUGUCUUCUGGAGAACAUAUUGUUCCUCAAUCAGAUGAAGACCUGAACUUUUAUGGUUACGAUGCAACUAAACUGGCAAAUGACGAAUCUGCGACAACACUAUCAAUCAAUCCUGACACUGUACCAUCAUCAAAGGCGGUUAACGCUGCUACUCACCAUUUCAAGAAAAAGAGUAACAAGUACUCUGAUCCAUUGGGUCACAGGUCGAAAGCAUCGGAGUCGUGGAUAGUACCAGCAUCAAAACACGAACCGACUGUCCAUGAGGCUCAUUACAUGGCUAAAAAACGCUACCCUGACUGUCCGAGUGCACUCAAAAAGCCAUACGGCAUGUCUAGCAAGUAUAGAAAGUAUAAUGAAAAACAUUCAAACAGUAGACCUUCUAGUAGAUCGCAAAGCAUAGUUGAUGACAACAAAACUAGAGUAUCAACCUUAGAUAAAACACGGCAUUCGAAAGGCAGAAUUCCCGUGAAACCCUCUAUCGAUCUAUCAAAAGACCCCUUCAGUCGAGACGAAAAAGCACGAAAGCAAUCUGCUGUUUCACAAGUAUCUAGUGCUUCGUCCACCACCUUAGGCAGAGUAUCUCCACGAUCAAAACCAAGUUCUUUUUCUCAUCAUCGCUAGGAUAAAUAAGUUCCUACCGUUAAAUCUAUUUAUAGCUGUUGAAGUCCUUCUUGGUUUCUCAUUUAGUGUGUUCAAUUAUAUUCUAAUAUGUAAGUGUUAUCUGGGAUUAAAUCAAGUUGCAUUUAAGAAAACCCCGUUUAGAGGAAGUACUCUGGAAUGAUGUCAUUACUUCAGAACACCAGGCACACUUAAUAACACCAUAGAGUCAUCUUAUUAUUUAACAUUCCACCCAAAAUCACCGUACUCGUUAUAACUAUACAAGUCUCAAUAUGAUAAAGGCCGUUUUUUUCUUAUACUUUUUUCUAAGAUUCCAGUUGGUGUUUCAAAAUUGGGUAUGCUGUAUCCACCCAGCAGGGUUGAAAAAGGUAGACGGCCAAAGCCUCAU  >Ss-AA_clean.1_(paired)_contig_6637  CGUAGACGAAGUUUUUGGAAUGAAAGUUCCGGGUCCAUGGAAAUCGAUAUCUGGUUCGUCGAGUAUUUCUCCCGUUGGAGAAAUUAAUGCCCUGGUCUCGACGUUUGGAACAGGUUCUUGUGGGGUUCCAGACAAUGUGCCAGUGAGCGCUGCAAUAGAAGUUCUUAGCUCCUGAUCGUUUUCAAUUAUAUUCCCUAGUCCUCUGACAAUCUCCGUUUCUUCGAAAUUACUUUC  >Ss-AA_clean.1_(paired)_contig_651  CUGGUACCUACCUCCAGAAAUUCUUUUUACAUAUCAUCAUAGACUCAUGACACACGAGGUCAAGAUGAGCAAUUCCGCAGAUCUCAUAAACGAAGAAAGUAAUUUCGAAGAAUCGGAGAUGAUCAGAGGACUAGGGAAUAUCAUCGAAAAUGAUCAAGAGCUGAGGACUUCUAUUGCGGCACUCACAGGCACGUUAUCUGGAACCACACAAGAACCUCUUUCAAAUCUUGAAAACCAUGCACUAAUCUCUCCAACAGGAGAAAUACUCGAUGAGCCCAAUACCGAUUUUCGUGGACCCGGAACUUUCAUACCAAAACCUCCGUCUACAGCGCAUCGACAUGAAGUAUCUCUUCCGCAACCAGGAAUUAAGAAUAUCUUUCCUUCGAGUACUGCACUCCUUACUGAAAGCGCAAAUGCUAUCAAUGCAUGCCGGGAGAGAAUCAAUGCGUUAGAAACGCUAGUCCAUUCCCAAGGAAAACAACUAGUUGAAUUAAGAGAGAAAAAUUCAGCAUUGCAAAUCGAAAUUCAGGCUUGUCAUUCGUCUGUUUCGCUCCAUAGACAAACAACUGCUGAUGCGAUCGGAGCUGCAUCUGCUGAUUUACGCGCACGAAUUGUUGAUGCUUUGUCUAUUUACAAAGAGACUCCCGGCUUGGUUGAACGUUUAACUGCAGCAACGAAUAACAUCUUAUCAAUGUAUCCUGAGGAACUUAAGACGGGUUUGAAGAAAGUAGAAUUAACUAAGAAUGAUCAGGUCUUAAUGAAGAAGAUUACUGAAAAUUCUACUCAACCAAAGAUGAAAUUGCCGAAACAUCUCAGAAAGUAGUAUUUCCUUUCGAAUAGUAAGAUUAUUUAAUAAAACUUAGGAGAUUACCAUACAAUCUCAUUUUAGUUCAAUAGUACUCAUUUGAAAUCGAA  >Ss-AA_clean.1_(paired)_contig_650  CUCGAUAUUUACACCAUAGGUGUCUUUGUCUUUAAAGACAUCUGGGAUCGAUAGGCGUGAGUCAGACGUUGUAAGCAGCCUUUCUAUUUCCAAUGAGUACUAUUGAACGAAACUGAGAUUGUAAGGUAAUCUCCUAAGUUUUAUUAAAUAGUCUCACCGUUCUGAAUAAAAUACUAUUUCCUGAGAUGUUUUGGCAAUUUCAUUUUAGGUUGAGUAGAAUUAUCAGUAAUUUUCUUCAUCAACACUUGAUCAUUCUUAGUCAAUUCUACUUUCUUCAAACCCGUCUUAAGUUCCUCAGGAUACAUUGAUAAGAUAUUAUUCGUUGCUGCAGUUAGUCUUUCAACCAAGCCAGGAGUCUCUUUAUAGAUGGACAAUGCAUCAACAAUUCGUGCACGUAAAUCAGCAGAUGCAGCUCCAAUUGCAUCAGCAGUCGUUUGCUUAUGAAGAGAAACAGAUGAAUGACAGGCUUGAAUUUCGACUUGCAACGCUGAAUUUUUCUCUCUUAAUUCAGCUAUUUGUUUUCCCUGAGAGUUGACUAACAUUUCUAACGCAUUGAUUCUCUCCCGACAUGUAUUAAUGGCAUUUGCACUUUCAGUGAGGAGCGCGGUACUUGAAGGAAAUAUAUUCUUAAUUCCUGGUUGUGGAAGAGAUACUUCAUGUCGAUGCGCUGUAGACGGAGGUUUUGGUAUGAAAGUUCCGUGUCCAUGAAAGUCGAUAUCUGGUUCAUCGAGUAUUUCUCCCGUUGGAGAAAUUAGUGCACGGUUCUCGAGGUUUGGAACAGGUUCUUGUGUGGUUCCAGACAACGUGCCAGUGAGUGCUGCAAUAGAAGUUCUCAGCUCUUGAUCAUUUUCAAUUAUAUUCCCUAGUCCUCUGACAAUCUCCGUUUCUUCGAAAUUACUUUCCUCGUUUAUUAGCUCAACGGAAUUGCUCAUCUUGAUCUCGUUUUAAAUGAGUCUGUGAUGAAGUGUAUAAGAAAUUUCUGGAGGUAGGUACCAG  >Ss-AA_clean.1_(paired)_contig_1093  GAUUGAAUGCAUCAAGAUUUUCCGGAGUUGGUUGUUUAGCGACAGCAAUUGCUAAAAUACCAGCAAAUGCAGCAAUUUCCAUAGAGUCUACUCCGUAAGCAAAGAGUAUUGCCUCGUCAGGAACUUCAAGCUCAUCAUACCAUUUUGGAAUGCAUGAAUCAGACAAUGGAGGCAAAUCAGCUUCACUUGCAUUCUCGACAGGGACAUGACUGAAUCGAGAUAUAACCCAAUUCUUGAUAUCAGUGGCAGUAUCCUUGAAUGGGUUAGCCAAUCGUGCAGUGGAAUGAUUUACACCACUUGCUGAGAUCAAUGCACCGAUGAUAAGUUUAGCAAUAUCCUCGGAGUCCUCUGCAUGUUGAAUUCCAGCGAGACACAUGCGAACAGGAAGAUCAGUGACACUCACGAAAAUAUCGGAGUAUUCACAGUUGCGAGUUAAAUCGAUUACAGGAAGUCGAUCGCGAUUAGCCUCAAUAUUUACACCAUAAGUGUCCUUGUCUUUAAAGACAUCUGGGAUUGUUCGGCGUGUAUGAGACAUUGUAAGCAGUUUUUCUAGUUCUAAUGAGUACUAUUGAACGAAAAUGAGAUUGUAAGAUAAUCUCCUAAGUUUUAUUAAAUAAUCAUAUAAUUCUAACUAACAUAUUACUUUCUGAGAUGUUUCGGCAAUUUCAUCUUCGGUUGAGUCGAAUUAUCAUUAAUUUUCUUCAUCAAUACCUGAUCAUUCUUAGUUAAUUCGACUUUCUUCAAGCCUGUUUUGAGUUCUUCAGGAUACAUCGAUAAGAUGUUAUUCGUUGCAGCAGUUAGUCGUUCAACCAAUCCGGGUGUCUCUUUAUAAAUGGACAAUGCAUCAACGAUUCGUGCACGUAAAUCAGCAGAUGCAGCUCCAAUCGCAUCCGAUGUCGUUUGCUUAUGAAGCGAAACAGACGAAUGACAAGCUUGAAUUUCAACUUGUAGUGCUGAAUUUUUAUCUCUUAAUUCGACUAUUUGUUUUCCUUGAGAAUUGACUAGCAUCUCUAAAGCAUUGAUUCUCUCCCUGCAUGCAUUAAUAGCAUUUGUACUUUCCGUGAGGAGCGCGGUGCUCGAAGGAAAGAUAUUCUUAAUUCCUGGUUGUGGANNAGAGAUACCUCAUGUCGAUGCGAUGUAGAAGGAGGUUUCGUUACAAAAAUUCCAGGUCCAUGAAAGUCGGCAUCUGGUUCAUCAAGUAUCUCUCCAGUUGGAGAAAUGAGUGCACGGUUUUCAAGGUUUGGAACAGAUUCUUGUGUAGUUCCAGGUAAUGUGCCAGUGAGUGCUGCAAUAG  >Ss-AA_clean.1_(paired)_contig_925  UCCUUACUCUUAUCUAAAGCUAAUGUUACCGAAAAAGGCGAGCUCUUAAUAGAUAACAAGUAAUGCCCUUUAGCAGGACACAUACAGACACGUAAUUUCGUCUCCAUGAGAAGUUUAGCCAAAAUCUCCCAAGAAAGCUCAGUCUUAUCUUGCAUGUUAAGGAUUAAUGCAUUUUGGAAAAAUCCAAUUUUCUUGUCCAGUGUACGAUGGAGAGAAGUCGUAGCAUUUAUUUGCUCCUGUAGACGAUCCAACUCACCAGAAACGGUAACUUGUAACUCAGUUGAGCUUGAAAAUUUGCAAAGGAUUGAGAGAUCCUGACAGUUAGCUAGGUGGAUAGACAUACUGAUGGAAAUGACUUAUGUGUAACUCCUAAGUUUUAUUAAAUAGUGCGAGAGAUCAUUCGUAUGGAAAAGCGAUAAAUCAGAUCAGAGUUGAGAUUAUUGGGCAGGAGCUUCAGGAACAGCUUCUUCAGCAACCUCUCGAAUAUUGAGAAGAGCAAUAAGUUUCUCUCUGUGUUGAACACUAAGAGUUCCUCCCGCAUAUUGACUGAGAGUAGAUUCUUGUUGAGACAAGGCAAAAACAGCAACACCGAGAAGCUCAUUAAUGUCGUUUCUAUCAAUGGGAAUAUAUGCGCUCCCAAAUAUAACUUUGAGAUACAUUCGACGCUUGGUAGAAGUAUCGAGGAAACGUUGGAGAGCAUGAGCAGCAGCGAAAUAUUCAGUCUCAAGAUCACGAAACUCUUUGAAGAACUGAGGAUAUUUUCUAGCAUACUUAGUAAUCAGUAAGAGAGGACUGAGUGAUGCACCAAAAGUGAGUCGAAACAUCACAUAAAACAUUCGUUGAGUACCGGAGACCAUUGGGUCAUCCUUAUCAACGAUGGAGCUCAUCACAAGAGCACGAUCUCGAAUGAUCGAGUUGAAUGUCUUGUUGACUUUACUNNCGUGUGACAAAUAUGGAGAAUUGUCAGUGAAAACCUUAAGCUCACCUGAAGGCAUGAAUUGAGAGAUUGCAUUUCGUCGUUUAAGAUUGAAUGCAUCAAGGUUCUCAGGAGUCGGUUGUUUAGCAACAGCAAUUGCUAAGAUACCAGCAAAUGCAGCAACUUCCAUAGAGUCAACUCCGUAAGCAAAGAGGAUUGCCUCAUCAGGAACUUCAAGCUCAUCAUACCAUUUCGGAAUGCAUGCAUCGGGCAAUGGAGGUAAUUCAGCUUCACUUGCGUCCUCUGCAGGAAUAUGACUGAAUCGAGAUAUAACCCAAUUCUUGAUAUCAGUUGCAGUAUCUUUGAACGUAUUAGCCAAUCGUGCAGUAGAAUGGUUUACACCACUAGCUGAGAUCAAUGCACCGAUGAUUAGUUUAGCAAUAUCAUCAGAAUCCUCAGCAUGUUGAAGUCCAGCAAGGCACGUGCGAACAGGAAGAUCAGUGACACUCACAAAAAUAU  >Ss-AA_clean.1_(paired)_contig_3817  AUUCUAUCAAUGGGAAUAUAUGCGCUCCCAAAUAUAACUUUGAGAUACAUUCGACGCUUGGUGGAAGUAUCGAGGAAACGUUGGAGAGCAUGAGCAGCAGCGAAAUAUUCAGUCUCAAGAUCACGAAACUCUUUGAAGAACUGAGGAUAUUUUCUUGCAUACUUAGUAAUCAAUAAGAGAGGACUGAGUGAUGCACCAAAAGUGAGUCGAAACAUCACAUAGAACAUUCGUUGAGUACCGGACACCAUAGGGUCAUCCUUAUCAACGAUGGAGCUCAUCACGAGAGCACGAUCGCGAAUGAUUGAGUUGAAUGUCUUGUUGACUUU  >Ss-AA_clean.1_(paired)_contig_952  AUAGUACUCAUUUGAAAUCGAAGAAUUGUUUACAAUGUCUCACUCCCGACGAACUAUCCCAGAUGUCUUUAAAGACAAAGACACUUAUGGUGUAAACAUUGAGGCUAAUCGCGAUCGACUUCCUGUAAUUGAUUUAACUCGCAACUGUGAAUACUCUGAUAUUUUUGUGAGCGCCACUGAUCUCCCUGUUCGUAUGUGUCUUGCUGGAAUCCAACAUGC  >FirsU_Contig67  GGAUAUUUUCUGGCGUACUUAGUAAUCAAUAAGAGAGGACUGAGUGAUGCACCAAAAGUGNAGUCGAAACAUCACAUAGAACAUUCGUUGAGUACCGGAUACCAUUGGGUCAUCCUUAUCANACGAUAGAGCUCAUCACAAGAGCACGAUCGCGAAUGAUUGAAUUGAAUGUCUUGUUGACUNUUGCUCAGAACUGCGUGCGACAAAUAUGGAGAAUUGUCAGUGAAAACCUUGAGUUCCCCGNGAAGGCAUGAAUUGAGAGAUUGCAUUUCGUCGUUUAAGAUUGAAUGCAUCAAGAUUCUCANGGAGUUGGUUGUUUAGCAACAGCAAUUGCUAAGAUACCCGCAAAUGCGGCAAUUUCCAUANGAGUCUACCCCGUAAGCAAAGAGGAUUGCUUCAUCAGGAACUUCAAGCUCAUCAUACCAUNUUUGGAAUGCAUGCGUCUGGCAAUAGAGGUAAUUCAGCUUCACUUGCGUCCUCUGCAGGANAUAUGACUGAAUCGAGAUAUGACCCAAUUCUUGAUGUCAGUUGCGGUAUCCUUGAACGUANUUUGCCAAUCUCGCAGUAGAAUGGUUCACACCACUGGCCGAGAUCAAUGCACCGAUGAUUNAAUUUGGCAAUAUCAUCAGAAUCCUCUGNCAUGUUGAAGUCCAGCAAGGCACAUGCGAACAGGAAGAUCAGUGACACUCACAAAAAUAUUCAGAGUAUUCACAGUUUCGAGUUAAAUCGAUAACAGGAAGUCGAUCACGAUUAGCCUCGAAUAUUUACACCAUAGGUGUCUUUGUCUUUAAAGACAUCUGGGAUCGAUCGGCGUGAGUCAGGACAUUGUAAGCAGCUUUUCUAUUUCCAAUGAGUACUAUAGAACGAAACUGAGAUUGAAANGGUAAUGUCCUAAGUUUUAUUAAAUAGUCUCACCGUUCUGAAUAN  >FirsU_Contig68  ACUGCGUGUGACAAAUAUGGAGAAUUGUCAGUGAAAACCUUAAGCUCUUCUGAAGGCAUGNAAUUGAGAGAUUGCAUUUCGUCGUUUAAAAUUGANAUGCAUCAAGAUUUUCAGGAGUUGGUNUGUUUAGCAACAGCGAUCGCUAAAAUGCCAGCAANAUGCUGCAACUUCCAUAGAAUCUACUNCCAUAAGCGAAGAGUACGGCUUCAUCAGGAAUUUNCAAGCUCAUCAUACCAUUUUGGAAUGNCAUGUAUCUGGUAAAGGAGGUAACUCAGCUUCACNUUGCAUUCUCUGCUGGGACAUGACUGNAAUCGAGAUAUAACCCAAUUCUUGAUAUCAGUUGNCAGUAUCCUUGAAUGGAUUAGCCAAUNCGUGCAGUUGAAUGAUUUACACCACUGGCCGAGANUCAAUGCACCAAUGAUGAGUUUAGCANAUAUCAUCAGAGUCCUCGGCAUGUUGGAUUCCAGNCAAGACACAUACGAACAGGGAGAUCAGUGGCGCUCACAAAAAUAUCAGAGUAUUCACAGUNUGCGAGUUAAAUCAAUCACAGGAAGUCGAUCGCGAUUGGCCUCAAUGUUUACACCGUAAGNUGUCUUUGUCUUUAAAGACAUCUGGGAUAGUUCGUCGAGAGUGAGACAUUGUAAACAGUUNUUUCGAUUUCAAAUGAGUACUAUN  >Ss-AA_clean.1_(paired)_contig_951  CUCAGUUUCGUUCAAUAGUAUUUAUUUGAAAUAAAAAACUGCUUACAAUGUCUGACUCACGCCGAUCCAUCCCAGAUGUUUUUAAAGAUAAAGACACUUAUGGUGUAAACAUCGAAGCUAAUCGUGAUCGACUUCCUGUUAUCGAUUUAACGCGAAACUGUGAAUACUCUGACAUCUUUGUGAGUGUCACUGAUCUUCCCGUUCGCAUGUGCCUUGCUGGGCUUCAACAUGCAGAGGAUUCUGAUGAUAUUGCUAAACUAAUCAUCGGUGCAUUGAUCUCGGCUAGUGGUGUGAAUCAUUCUACUGCACGAUUGGCUAAUACGUUCAAGGACACCGCGACUGAUAUCAAGAAUUGGGUUA  >Ss-AA_clean.1_(paired)_contig_2258  AUUAUAUUCAGAAUUCGAAAGUCUAUACCUUCUGAUAUAAUUCCUAAUCGCGAUUUCUUUUCUCGUGCAAUUAGUCAAGGCAUGAUAGAUGCAAUCUCUAUUGACCGUCCAAGUUUCCAAUGGGAGAAAUUCAGAGUGGAAGAUAGAUUGAACACUAACCAUCGUUACCUAUCUGAAUCAAUUAUAUGUGAUCAAGCUGAGUUCAUUCGUCGAGAUAUUUGUACUGCUCCUUCUGACGGUAGGGAGUAUCGAAUCAAUCAUACUCGUUAUGAUUUUACAUGCACUGCAAUCGAUUCGGCAAAUACUGCAUAUCCGAAUACU  >Ss-AA_clean.1_(paired)_contig_2349  UCUGGUGAGUUGGAUCGUCUACAGGAGCAAAUAAAUGCCACAACUUCUCUACAUCGCACAUUGGAUAAAAAAAUUGGAUUUUUUCAAAAUGCCUUAAUCCUUAACAUGCAAGAUAAAACUGAGCUUUCUUGGGAGAUUUUGGCCAAACUUCUCGUGGAAACGAAGUUACGUGUCUGUAUGUGUCCAGCUAAAGGGCAUUACUUGUUAAUUAUUAAGAGCUCGCCUUUUUCGGUAACGUUGGCUCUAGAUAAGAGUAAGGAAGACAUGCCAGGUCUUUAUGUCU  >FirsU_Contig95  CUAAGUACGCCAGAAAAUAUCCCCAGUUCUUCAAAGAAUUUCGUGAUCUUGAGACUGAAUNAUUUCGCUGCUGCUCAUGCUCUCCAACGUUUCCUCGAUACUUCUACAAAACGUCGAAUGUNAUCUCAAAGUUAUAUUCGGAAGCGCAUAUAUUCCCAUUGAUAGAAACGACAUUAAUGAGCNUUCUCGGUGUUGCUGUUUUUGCCUUAUCUCAACAAGAAUCUACUCUUGGUCAGUAUGCGGNGAGGAACUCUGAGUGUUCAACAUAGAGAGAAACUUAUUGCUCUUCUCAAUAUUCGAGAAGNUUGCCGAAGAAGCUGUUCCCGAAGCUCCUGCCCAAUAAUCUCAAAUCCAAUCUGAACUAUNCAUUUUCUUAUCUAAACGAUCUCUCGAAUUAUUUAAUAAAACUUAGGAGUUACACAUAAANUCAUCUCCAUCAAUAUGUCUACCCUCUUAGAUAACUGUCAGGAUAUUUCAAUCCUUUGCANAAUUCUCAAGCUCAAUUGAGUUACAAGUUACCGUGUCUGGUGAGUUGGAUCGUCUACAGGNAACAAAUAAAUGCCACAACCUCUCUCCAUCGAACAUUGGAUAAGAAAAUUGGAUUUUUCCNAAAAUGCCUUAAUCCUUAACAUGCAAGAUAAAACUGAGCUCUCCUGGGAGAUUUUAGCGANAACUUCUCGUGGAAACGAAAUUACGUGUCUGCAUGUGUCCAGCUAAAGGGCAUUACUUGUNUAUCUAUUAAGAGCUCUCCUUUUUCGGUAACAUUGGCUCUGGAUAAAAGUAAGGAAGACANUACCAGGUCUCUAUGUUUUCCUGAACAAUAAGUACGGACUUCAUAUGUCGGAUUCGGGAUNCACUUGGAUCCGGAAGCACAAAUUUGACUGAACCCGAUGUCAUUAUCUUCAGAAUUCGGANAGUCUCCACCCUCCGAAAUAAUUCCUAAUCGCGAUUUCUUUUCUCGUGCAAUUAGUCAAGNGUAUGAUAGAUGCAAUCUCCAUUGAUCGUCCAAGUUUCCAAUGGGAGAAAUUCAGAGUCGNAGGAUAGAUUGAACACUAAUCAUCGUUACCUGUCUGAAUCUAUUAUAUGCGACCAGGCUGNAGUUCAUUCGUCGAGAUAUUUGUACUGCACCUACUGAUGGUAGGGAGUAUAGAAUCAAUCNAUACUCGUUAUGAUAUCACAUGCACUGCAAUUGCUUCGGCAAAUACAGCAUAUCCGAAUANCUCUCAACUACUACAUUAAUAAUCUUUAUGUUUGUCAUGGUUAAUCN  >Ss-AA_clean.1_(paired)_contig_4783  AUGCAUCAACAAUUCGUGCACGUAAAUCAGCAGAUGCAGCUCCAACUGCAUCAGAAGUCGUUUGCUUAUGAAGAGAAACAGAUGAAUGACAAGCUUGAAUUUCAACUUGCAACGUUGAAUUUUUCUCUCUUAAUUCAACUAUUUGUUUUCCCUGAGAGUUAACUAACAUCUCUAAUGCAUUGAUUCUCUCCCGACAUGCAUUAAUGGCAUNNNNNNNNNNNNNNNNNNNNNNNNNNNNNNNNNAGAGAUACCUCAUGUCGAUGCGCCGUAGAAGGAGGUUUUGGUAUGAAAGUUCCAGGUCCAUGAAAGUCGAUAUCCGGUUCGUCGAGUAUUUCUCCUGUUGGAGAAAUUAGUGCACGGUUAUCGAGGUUUAGAACAGGUUCGUGCGUGAUUCCAGACAACGUACCAGUGAGCGCUGCAAUAGAAG  >FirsU_Contig29  UAGAGAGUCUCUCUUCGACAGUGAGUCCUCGCUGGAUGCGCGAUGGGGAAUAUCGGAUAGNAAUUGUUUGAGCUCUAACCGUCGCAUCUCUUAACAGAGAUACUACGUUAAAUAUCAUCUCNAGUUGUCGUAAUCAAGAUUUCAUUGAUUUCUUUUCCUUUAAAAUGCGCUGGUAAUUGAAANUUGAGGACUAGCAUAUUUAAUUAAUUCGGUAAGAGCUAAAUGUAAUUGAUUCAACAUAAANUUCAUCUACUUCCUUAGGAGGAGCAUUCAAUUUAACUUGGAGAUCUAGUACCGUUUGUCGNUAAUUCCAGCAAUAUAGUUUCAUCGAGUAACACUCUUCUCUCCAUUGUCGUUAACCUAUUNACAGAUAUGCGUAUAUUUCUCCUUAUACGAAGGCAUUAUCCUAAGUUUUAUUAAAUAAACNACAUAAAAUCGAUCUAGAUUAAUUAUCAUAUUUGAAAAUAUUAUCACAAAUGGCUCGGCANUCUCAGAUCUUUCCCAGUCAUAUCCGUAUUAGUUAAAUUUGUGUGCAUGAUAUCCCAUAANAUGAAUGUAAAUUUCAGAUACUUCCGUUCGUCUUGCCUCGUAUAAAGCAGAUAACUUUGUNACGUAAUGAGAGUACUGUAUCUGUAACAUCUAAUUGGAAAUGUCUAGAAAUAUUGGGUUCNAACAUUCCAAAACUGCGACACAAGCUCCGUAGAUCCAUACGAAUAGUUGAUUUCAUUUUUNCGUCUUGAAUUCUACACUUUGAUAAUUCCCUGCUCCAAUAGACCCCAAUUGAGUCGCAUANCAGAACAAUGUCAGAUACNNUUAUUAUCAGAAAUACAGAGAGAAAUAAUAAUGGUAGGGUNGUAUCAUACUUAUUAAGCAUCGUAUUUCAUCCUCAUUUCCUGCGUAUCUGAUGAUCACUUNCAGAAUCGAUUGGUAAUCUGAGAAGCAGUGUUUUUAAUUUUUGGAAAGAAAUGUCUAAGUNCUAUUACUAACAUUGGUCUCAUUUCAUUGAAACAAUCGAGUUCACCUUUUAAGACAUCUCNCAUCAUCUGAGUAAGUAUGAUUACUCCAUGAGAAAUUCCCAGUAUUGGCCGACAUGAUUANAUUCUGGAGGUUUAUACGAGGCAGAUCUGUGUGAUAUAGAUGGGAAAGAAGAGAUUAAAUNCGUACCCAAUCACACCUACGUGUCUAUCGACUAACGCACGGGAUGUUGCACCUCGUCCGANCUCCAAUUACAUGGAAUUUCCGUUGCUUGCUUAAUCCUCGAUCGAAUAAUAUUUCAAAUANUUGCAUUCCAAUCACUAUAAAGAGGAGAGUAGAUUCCUACUGUCCUACGAUGAAGUAUUCNGUAAUCGAUCAAUAACACUACGAUUUUGUUUCAGUACUACAUCAUUCGGUUCAAGAGAUCNCAUAAUGUUCUGUAUAUUCCACUUUGCAUAUCCCGUGGUUCGAGAAUUUUAGAGGAUGCGNUUAUUGUAUCCUUUUUAUCUUGAAACUUUUCCCUGUCUCGAAGCAUACGCAUUGCUUCCUNUAUCGGUACGAUGCAAGAACAGGCAUGGCAUAUCAGGGAGCGUGUAUUCUAAGUUCGAUANUCUUCCGAGAAAUUUUCCGUGUAACCACUGAGAUAUAUGUCGCCGGAUCUUGUUUCAGAANGUUCCAUCGGCGGUUCAAUACUUAAUACCGAUUUCCGAAGACUAGUAAGAGAUUGGAUAGNGAUAUGUAGCAAUUGCAUGCGCUAAAAUUAGUCGUCUUCUUCCUAGUCUAGCCGAAGAUUNCUUUCCAGUUGCUGAACAGGAUUAAUCGAGGUAUACUACUCAUUCCUCUAGUCGAUAAUANGUGUAUGUGCCAUACGCAGGAACUGAGUAGCCAUAUAAUCCACAGGUCGCUUAUACCCAGNAAUGAGUGGGUUGAAGAUUUAUUCUACGACGUUGAUUGUAUUGGGUUGUAGAAAACAUUGNGAUGUAGUCUGAUUCUUACCCACUCUCCUGAAUAAUAAGUAGCUUUUGUCUUUAAGAUUUNUCUUGAAUAGUGGCAGCGAUUUGUCAGGGGAAUUUGAAACGAAUAUUGCAUAAUAUGCGUNCAGUUAUAAUAUUCCAACAGAUAGCAUGUUCAACGAUAUUAGGAUCGACUCGUGAAAUUUNCUUUAAAAUCGAAGAUGUCUGCUGUUCCUAGUAUUCCAUCAUAAGGUUUCUUCUGGUCAGNCGCUUGAAGAAACACAUGAUUCUAUAUAAGAGUAAAUCAAAUCUAAAUCCGAGGUUGGUGNAUGAAAUAUGUGGUAUUACUGAGGGGAGUGGAAUUUCAGGUAUUUCAGAUGCGAAGAUCUNCUCCUACCCAUGCUAAUUUGUUAGACGUAAGAUCGGGCCAUUUAAUUUCUACAAUCUGCUNCUGAAGGAAUCUUAGAAACCGAAGUAUUAAUUUCACGUAAUUGACUUGGGAUAUAAUAGCNAAAUAUUAGGAGGAAGUAUAGUGUUACCAGUAGACAAUAUUUGAUAUAAAUUAGUCAAUGNUCAUGUAAAGAGUCUGAAACACCACUGGAUAAUCUGCAUCUCCACCUGAUAGAAUACCUGNCUUGGUCCGAGCUAAAGAUAAUAUGUGUGGGCACAGAAGAACUCCCUAAAACGGAGAACANCAUGAGAUGUACUCUUGUGGCGAUGAACAGCAGUUCCACCAAUUGAUUCGGGAAAAAUUGNGUAUUAAUUUUUCUAGGGUCCAAGGAGAACGAGAAGAAAUAAUCGAUUGGAACAGCGGUUNUUAGUGAAGGGUCUCCUUGUAAUUCGGAGUAUGUGAGUACUGCAUUCUUCAGAUCUUUCANUAGUUGAAUUUGUACUUACAAUACGAUAUCCGUGAUCGGAUAAUUUUUGUUUUGUUGAAGNUUCCAAAAUUCGGAGGUGCUCGACCGCAAGUAGUCAAUAUGUCUUUUCUAUUUUCGGUUGNUAGCACUAAUUAAUGGUAUGCCAGGUUUGAAAUAGCCAAUUUCAAAAUCAAAAGGAGUAUNAAACUCCGAUCGAAGUAUUAUCUAAUCCAAGAUUCCAAUGAGUCCUUAACUCAGUAGCCANUUUCAAAGGGAGGUGGAUGACGUCCUCCAGGUUUCUUAGCAGCUAAUACCCAUCGAUCAANGAAGAACUGACAGAAUUGUAGAAUUAGCCUGAGAGAUUCUAUCAAUGAAAUUAAUUCCCGNGGAUAAUCUUCUCUAUAGUUCUCGUCAUGGAGAACCUCUUAAUAGUCUUAUUAUAGAGACNCUGCCGGUGUUAACUCAAAUAAAUCUGCCGCAAUUUGUGGGUGCAGAGGUUUCAUCUUUGNUCAAUAAAGAUUUAUAAUUCUCCCCUUGUUGCCGUAGAUCGUUCGAUAUCAAUUGCUUGANUAUCCUUAUUAUUCACUAUUCCAGGUAAUGCUUUAGAUACAGCAUUAGAUAUCAAAUGAGNAUGCAUCAUUAGGUUUUUCAAUCGGGAUUGAAUGUGGAUCAUUAAUUAGAUUAGUCGCAUNCAAUUUUGUGCGGUACAAACUCUUGAGUCCUAAGAAGCCACAUGUAAUUUCUGACAAUUGNGUUCUGUUUGAGAUAAGUAGUAUGUAGCUGCAACAUCAAAAGACAAAUCAUCUGAUUCUCNCUUUACUGAAAUAUCGUGUCCAUGGCAUCANUAGGCAGUCCACCCAACGAUCCUGGAAUUANAAAGCACUUUCCGAGAAGUUGCAGAUCGUANAUAACCGCAAGAUACCUUUAUGCUCAAGACNUAUAAAGCGGUGAAGCUAAUCUCCGACGUANAUAAUAAAAUUACUUGAAUAUGCUUCCACCNACACGGCACGAAAAGAAUUAUGAAGCGUUCNCAGCUACUGCAACAGAAUUUGCAACAACUCNCAGAUAUUUCCUUACUUAGAGAAGGAAUUGNUGUAAUCUAAUCUAGAGAAAGCUCUAGAUGNAGAAUUUAAGACUAUACAUAGUAUGAACUCCGCGGAUAUAGAUUUCUUUUCCAUAGGUUANGGACAGUUCGGGAAUCAAUACAUUCUUCUGGCUUUACCUCAUGAUUCAAUCGCUCGCAUCNUUCGUUCUACAGAGUAUAGAAAAGAUUGCAAUGCAAGACUGAGCGAUUGUUUUUUAGUGUNUGAACGACAAGAAGAAGACCUGAUUGUCACCUUGUCCUGCCAUCUGGAAGGAGCAUUGUUNCGUCUUGAAUGGCAUAGUACAUCAUAGCCAAUGUACAUAUUGUCCAAAGUGUUUGUUGAANUCCCUUCAAAGCCUCCUAGAUGAUUUCUCCAUACUAAAUUUGAUUCUGGCCAAGUAGAAGNCAUGUGAAUUUGGUGUGACACCUGGAGGCAAGGUAUUUUUAUCUGUCAUGACAAUAGUAGNACUGAGUGAAGAAAGAGUGUGCUUGAGAGAAUACACCUGGUAGUCCGAAGAUUUUUUCUANAUGAUCUGGAAAUGGGAUUGACCGAGCAUGCUCUCCAUCUCAGGUUCCAUCUGGAGAAAUNCUACUUCCACUAGACAAGUAUUCGUUCUAUUAUUUACAGCAGUUAAAUCGUAGAGUCUUUNUUUUGAGCUUAGUAUUCGUCAUGGUCAUAGUUUGUUGAGGUAAGUAUCCAUUGUCACCCGNAAUCACCUCCCAUAAAUCUCUUAAGAUUAGUUUCUGUCAAGACAAAGAAUAAACGAACUUNCGAAUGUUAGUUUACAGAAACAACGAGCGGAAGUUUUAAAUUCGCGUUCCUUUUGAGUCANGUUCAACAAUUCUUUCAUCUAUAUGGAAUUUACCUUUCCUCAUCCGCUCGACAAUUUGGANAAGUAUCUACAUCUGCUCGUUUAAUUAAUGAUUCUAGAAGUCGACGAUAACUCUCUCCGGNAUUGUUUAAACCAGAACCCAGAUGAUUUAUCUGCUCCCGGACAUAUCGCUUUAUCGUCAANUCAUAUCAAGAUAAUCAGGAGAAUAAUCAAAUUCCAUAAAUUUUGGGAAUUCAACAUCAUNAGAGAUCUGAAAGCGGGUAACUUGCAUCAUUGAUUUUUGUUAUUCCUGAUUUCCACAAAUNUGUAGAGUUUGGUGUUCUUCUUAGGACCAUCUUUAUCUGUGAAGGCUGGCCAUACCUGAUNUCUUUUUAAGGUAACGAUCUAGAACUAAUCUUUUAAAGUGUGCAUGAUAAUCUCGUAUUGNCCAAUAGAUCUAAGUUACCGGUAGGACAUGCCUCCUUCUUGACGGAAAUAGAUGAAAUCUNCAGCGUAAACAAAUGGAUGACCCGACAUUUUAGUGCAUCCAAAUAAUUCUGUUACUAUGGNAAAUAUCAUUCGUAGAUUCGAUGAGUUCUAUUAAUUGAGAUGUCAAUGGACAUUCAUUCGNUUUUAGAUAAUUUUUUCUCUUUUUCUUGAAGUUUCGCACACGUACGUACGAAUGAGCUUANAAGGUAAUACAUCACCUUGAGUCAAAGAUGUGAGAUACGACUUAGUUACUGAUUCAGGACNCUUUCACGAGUUCAUACCCUUUGUUUCCAUACUUCUUAAGUACCGCCUCUUGCCAUAAUANAUAAUUUUCGUAAGAAUUCACGGAGCUGUUGAUUAGAGUUAUGCAUAUUUGCAUCUAGAGNCAAGAAACGCAUUAAAACGAGCUAAACAUACGUCUUGAAUCAUUUGAAGCUGUUCAAAAGNAAUACAAUCUUCGAGUAGGUGCCAACUUCUUUGGCUUCCCAUUCUCAUCCGUGAUCAUCUNUACUAAAGCCUAUUUGUUCCAUAACAAAUCCAUCAUAAAAAAAGAAAAUUGAAUCAUUAANUGGUGAGUCCUUGUUUGCCGAAUUGUUUUUUCUGCUUCCGUUGUCGGUAUUUUUCUACUANGGAAAUCCCAAUAUCGAUAGGAUUCGUAAUGAAGAUACGUAUCUUCUUUUGCUUCUGGUANUAAAUUUCUCCUCAUUUUCUAUGAAGGUUUGAACUUCCGGUAAUGUUCCCCAUUUUUUGANAAGCACAGAGUUCUGAGCGAUACAGCUCUGAUGAUUCUGCUAGUGAAGUUUUCAAGCUUUNCUCGCGCUAUAAGUGUACGUGUAAAAAGAUCAGGAUAUUGAUCUGGAGUUACUAUUUGAGNGAGCCGUAGAGGUCCCUAUUAAGUCCAAUAACCAAGGAGUUGCUACCCGUUCGAGUCCAGNAAUAAGAAUGCAAUACAUUAUGUUUACAUAUAUUUUUUAUUAUCAACGGAACAUCUGAUUNUGAUAUAAGGAAAUUUACUCUUCGCAAAACGAUUUUGCUCUUUAUAAUGCAAUAUUUCAUNCAUAAAGUAAUCUUAACCUAUCCAUAAGAGAGGAGAGAAUGGGACUACGGAGAUGUUUUUNCCGCAGGAGCAGCAGAACGACGCUCUCUAACCGAAUCAAACUCGAAUGCCUCACUGAAAUNUCAUAUUACUGAUUUGAGCGAAGUGACCUCCUAAGUUUUAUUAAAUAAUAGAUGUACUUUNGGAAUUCUAGUAAGUAUUAUACAUUUGUGCAUUGAUCAUUUGCAAUCAUACCCUUUAACCNUGAAAUUAUUUCGAUUUCGUCAAAUUCUUCAUCUUCUGUAACUAAAGAAAACUCACUUGANUGAUUCAAUCAAGUUUGNUUUCCCAUACUCUUUUGGUUAGAUUCUCUAAUAAGACCAACGCAGCAUACAAAAGCAUAUANGGUCGAACUGCGGUUGACUAUCAAGUAUAUCCAUUCUUGUUGUNACGACUCUGAUUCUUACGUGAAUAUGGAUGGCUNCCUAAGUUUUAUUAAAUAAUCAUCAGANGAAGAUUUCAAACUAGAAGUCAUUAGUGUCGUUNUCCCUGCAUUCGGAUUAACCAUGACAANACAUAAAGAUUAUUAAUGUAGUAGUUGAGGGUANUUCGGAGAUGCAGUAUUCGCCGAAGCGNAUCGCAGUGCAUGUAAUAUCAUAACGAGUAUGAUUGAUUCGAUAUUCCCUACCGUCAGUANGGAGCAGUACAAAUAUCUCGACGAAUAAACUCAGCUUGAUCACAUACGAUUGACUCAGAUNAGGUAACGAUGAUUGGUGUUCAAUCGAUCCUCCACUCUGAAUUUCUCCCAUUGAAAGCUUNGGACGAUCGAUAGAGAUUGCAUCUAUCAUGCCUUGACUAAUUGCACGAGAAAAGAAAUCGNCGAUUAGGAAUUAUAUCN  >FirsU_Contig47  NAUCCACAUUCGAUUCCAAUUGAAAAACCCAAUGAUGNCAUCCCAUUUAAUAUCUAAUGCUGNUAUCUAAAGCAUUACCUGGAAUAGUGAACAACAGAGGAUAUCAAACAAUUGAUCUCAAACGNACCUACGGCAACAGGGAGAGAAUUAUAAAACACUAUNUGACGAAGAUGAAACCUCUACACCNCACAAAUUGCGGCAGAUCUAUUCGAAUUAACACCUGNCCGGUCUUUAUAAUAAGACUAUUANAGCGAUUCUCUAUGACAAGAACUAUUGAAAAAAUUANUCCCAGGAAUUAAUUUCAUUGAUANGAAUAUCGCAAGCAAAUUCCACAAUUUUAUCAGUUCNUUCUUGAUAGAUGGGUAUUAGCUGNCUAAAAAACCUGGAGGACGUCAUCCGCCUCCCUUUGNAAAUGGCGACUGAACUAAGAACUCNACUGGAAUCUCGGAUUGGACAAUACAUCAAUUGGAGNUUUAUACCCCUUUUGAUUUUGAAANUUGGGUACUUUAAACCCGGUAUGCCACUAAUUAGCGNCCACCACUGCUAAUAGAAAAGACAUUUUAACUACUUGUGGUCUGGCACCUCCUAACUUUGNGAACUUCAACUAAGCAAAAAUUAUCAGAUCAUGGAUACCGCAUUGUAAGUACGAAUUCAANCUAUGAAAGAUUUAAAGAAUGCGGUACUUACAUAUUCGGAAUUACAGGGAGACCCUUCUUNUAAAACCACUAUUUCAGUCGAUCAUUUCUUCUCGGUCUCCGUGGACUUUAGAAAAAUUAANUACCGAUUUUUCCCGAAUCGAUUGGUGGAACUGCUGUUCAUCGCCACAAGAGUACAUCUCNAUGUCUUCUCAGUUUUAGGGAGUUCUUCAGUGCCAACACAUAUUAUCUUCAGCUCUGACCNAAGCAGGUAUCUUAUCGGGCGGAGAUGCAGAUUAUCCGGUAGUAUUUCAGACUCUUUACANUGACGUUAACUAAUUUGUAUCAAAUAUUGUCUACCAAUAACACUAUACUUCCUCCUAAUANUCUGCUAUUAUAUCCCGAGUCAAUUACGUGAAAUUAAUACCUCUGUGUCUAAGAUUCCUUNCGGAACAGGCUAUAGAAAUUAAAUGGCCGAACCUUACAUCUAACAAAUUAGCAUGGGUGGNGAGAGAUCUUUGCGUCUGAAAUACCUGAAAUUCCGCUUCCCUCAGUAAUACCUCACAUCUNCAUCGCCAACAUCCGAUUUAGACCUGAUUUAUUCUUAUAUAGAAUCAUGCGUUUCUACUANGUGCUGACCAGAAGAAACCUUAUGAUGGAAUACUAGGAACAGCGGAUAUUUUUGAUUUCANAAGAAAUUUCGCGAAUAGACCCUAAUAUCGUCGAACAAGCGAUCUGUUGGAAUAUUAUAANCCGAUGCAUAUUAUGCAAUAUUUGUUUCAAAUUCCCCGGACAAAUCUCUACCACUCUUCANAGAAAAUCUUAAAGACAAAAGCUACUUAUUAUUCUGGAGAAUGGGUAAGGAUCAGACUACNACCCAAUGUUUUCCACAACCCAAUACAAUCAACAUCGUAGAAUAAAUCUCCAGCCUACUCNGAACUGGCUAUAAGCGGCCUGUUGAUUAUAUGGCUACUCAACUCCUACGUAUGGCACACANCACUAUUAUCGACUAGGGGAAUGAGUAGUAUACCUCGAUUAAUACUUUUCAGUAACUGGANAGGAAUCUUCAGCUAGAUUAGGAAGAAGACGACUAAUUUUAGCGCACGCAAUAGCUACGUNACCCUAUUCAAUCGCUUACAAGUCUUAGGAAAUCGGUGUUAAGUAUUGAACCUCCUACAGNAACUUCUAAAACAAGAUCCGGCUACAUAUAUCUCAGUUGUAACACGAAAAAUUUCUCGGANAGAUAUCGAAUUUAGAAUACACACUCCCUGAUAUGCCAUGCUUAUUCUUGCAUCGUACCGNAUAAAGAAGCCAUGCGUAUGCUUAGAGAUAGGGGAAGGUUCCAAGACAAAAAGGAUAUAANUAACGCAUCCUCUCAAAUUCUCGAAUCAUGGAAUCUGCAAAGUAGAAUAUUCAGAACAUUNAUGGAUCUCUCGAACCGAAUGACGUGGUAUUGAAACAGGAACGUAGUAUUAGUGAUCGACNAUCGAAUACUUCGCCGUAGAACAAUAGGAAUCUAUUCUCCUCUUUAUAGCGAUUGGAAUGNCAAUAUUCGAAAUAUUAUUCAAUCGAGGACUAGACAAGCAACGGAAAUUUCACGUUAUUGNGUGUCGGAAGAGGUGCUACAUCCCGUGCUCUAGUAGAUAGACAUGUAGGUGUUAUAGGAUNACGAUUUAAUUUCUUCUUUCCCAUCAAUAUCACACAGAUCUGCCUCAUAUAAACCCCCGGNAAUUAAUUAUGUCGGCUAAUACCGAAAACUUCUCGUGGAGUAACCAUACUUACUCAGAUGNAUGGAGAUGUUUUAAAAGGGAGAUUGGAUUGUUUUGAUGAAUGUAGGCCUAUGUUGGUAANUUGACUUAGACAUUUCUUUCCAACAUUUAAAAACAUUGCUUCUUAGAUUACCAAUCAAUUNCAGAAGUGAUUGUGAGAUAUGUAGGAAAUGAGGAUGAAAUACGAUGCUUAAUAAGUAUGANUACGUCCCACUAUUAUUAUCUCUCUCUGUAUUUCGGAUAAUAAAGUAUCAGAUAUUGUUUNUAUAUGCAACUCAAUUAGGAUCUGUUGGAGUAGGUAACUAUCAAAGUGUAGAAUUCAAAANCCAAAAAUGAAAUUAGUUAUUCAUAUGGAUCUACAGAACUAGUAUCGCAAUUCUGGAAUGNUUGAACCUAAUAUUUCUCGACAUUUCCAAUUAGAUGUAACAGAUACAGUACUCUCGUUACNGUACAAAAUUAUCUGCUUUAUACGAGGCAAGACGAGAGGAAGUAUCUGAAAUUUACACCCNAUUUGUGGGAUAUUAUGCAUACAAAUUUAACUGAUACAAAUAUGACAGGAAAAGAUCUUANGAUGUCGAGCCAUUUGUGAUAAUAUUUUCACAUAUGAUACUUAAUGUCUAAAUCUAACGUNGUUCAUUUAAUAAAACUUAGGAUAAUGCCUUCGUAUAAAGAGAAGUAUACGCAUAUCUGUNAAUAGAUUAACGACAAUGGAAAGAAGAGUUUUACUCGACGAAACCAUAUUACUAGAGUUANCGACAAACAGUUAUAGAUCUUCAUGCUAAAUUGAAUGCUCCUCCAAAAGAGAUCGAUGAANUUUAUGUUGAAUCAAUUACAUUUAGCUCUGACGGAAUUAGUCAAAUAUGCUAGUCCUCAANUUUCAGCUUCCAGCGCAUUUUAAAGGAAAGGAAAUUGAUGAAAUUUUGGCUACGACAACUNGAAAUGAUAUUCAAUGUAGUAUCUUUGUUAAGAGAUGCGACAGUCAGGGCUCAAACAAUANUUAUCAGAUAUUUCCCCUAGACAAUCAAACGAGGACUCAUCUCCUAGAAAGAAUAACAUCNGAACAGCGUAGUUCUCUGUCAAAUACCUCGAGCUUGACUACAAUUGAAAGUCAAGGUAAUNAGACGCAGCUUUGCAAUGACACCUAAAGCGUUAUAGUCGAAUCUCAUUUAAUUCUAUUUANACAAAAAUUAAUUAUUCAAGAAGAGAACAAUAUAUCUAACAAAGCUACGGGAGUACGAUC  >Ss-AA_clean.1_(paired)_contig_558  UGUUUUGUUGAAGUUCCAAAAUUAGGAGGUGCUCGACCACAAGUAGUCAGUAUGUCUUUCCUAUUCUCAGUUGUAGCACUAAUUAACGGUAUGCCGGGUUUGAAAUAGCCAAUUUCAAAAUCAAAAGGAGUAUAGACUCCGAUCGAUGUGUUAUCUAAUCCAAGAUUCCNNUCCAAUGAGUCCUUAAUUCAGUAGCCAUUUCAAAGGGAAGUGGAUGACGUCCUCCCGGUUUCUUAGCAGCUAACACCCAUCGAUCAAGAAGAACUGACAAAAUUGUGGAAUUUGCUUGGGAGAUUCUAUCAAUAAAAUUAAUUCCUGGGAUAAUCUUUUCGAUAGUUCUUGUCAUGGAAAAUCUCUUAAUAGUCUUAUUAUAAAGACCAGCUGGUGUUAACUCAAAUAAGUCUGCCGCAAUUUGCGGGUGCAGAGGUUUCAUUUUUGUCAAUAAAGACUUAUAAUUCUCCCCUUGUUGUCGUAAAUCAUUUGAUAUCAGUUGCUUGAUAUCCUUAUUAUUCACUAUUCCGGGUAAUGCUUUAGAUACAGCAUUCGAUAUCAAAUGAGAUGCAUCAUUAGGUUUCUCAAUCGGAAUUGAAUGUGGAUCAUUUAUUA  >Ss-AA_clean.1_(paired)_contig_977  AAUGAACUUAAAGGUAGUACGUCACCUUGAGUUAACGAUGUGAGAUACGAUUUAGUUACCGAUUCAGGACCUUUCACGAGUUCGUACCCUUUGUUUCCAUACAUCUUAAGUACCGCCUCUUGCCAUAAUAAUAAUUUUCGUAAAAAUUCACGGAGCUGUUGAUUAGAGUUAUGCAUAUUUGCAUCUAGAGCAAGAAACGCAUUAAAACGAGCCAAACAUACGUCUUGAAUCAUUUGAAGCUGUUCAAAUGAAUAUAAUCUUCGAAUCGGUGCGAGCUUCUUUGGCUUUCCAUUCUCAUCAGCGAUCAUCUUACUAAAACCUAUUUGUUCCAUAACAAAUCCGUCAUAAAAAAAGAAAAUGGAAUCAUUAAUGGUAAGUCCUUGUCUGCCGAAUUGUUUUUUCUGCUUCCGUUGUCGAUAUUUUUCUACCAAAAAAUCCCAAUAUCGAUAAGAUUCGUAGUGAAGAUACGUAUCUUCCUUUGCUUCUGGUAUAAAUUUCUCUUCAUUCUCUAUGAAGGUUUGAACUUCUGGUAAUGUUCCCCAUUUUUUGAAAGCACAGAGUUCUGAGCGAUACAGCUCUGAUGAUUCUGCUAGUGAAGUUUUCAAACUUUCUCGCGCUAUGAGUGUACGUGUAAAUAGAUCAGGAUAUU  >FirsU_Contig125  UUUCGAGAAGUUGCAGAUCGUAACAGCCGUAAAAUGCCUUUAUGUUCUAGACUAUAAAGANGGCGAGGCUAAUCUUCGGCGUAAUAAUAAAAUUACCUGAAUAUGCUUCCACCAUACAGCANCGAAAAGAAUUAUGAAGUGUUCCAGCUACUGCAACAGAAUUUGCAACCACUCCGGAUAUUNUCCUUACUUAGAGAAGGAAUUGUGUAAUCCAAUCUUGAGAACGCUCUAGAUGAGAAUUUGNAGACUAUACAUAGUAUGAACUCCGCGAAUAUAAAUUUCUUUCCCAUAGGUCAACACGGUUNCGAGAAUCAAUACAUUCUUCCGGCUUUACCUCAUGAUUUAAUCGCUCACAUCGUCGUUCCNACUGAGUAAAGGAAAGAUUGCAAAGCAAGACUAAGCGACUGUUUCUUAGUGUUGAACGACNAAAAAGAAAACUUGAUUAUCGCCUUGUCCGGCCAUCUGAAACGAACAUUGUUCGUCUUGANAUCGCAUAAUACAUCAUAGCUAACGUACAAAUCGUCCAAAGUGUUUGUUGAAUUCN  >Ss-AA_clean.1_(paired)_contig_557 UUCUAUCAAUGAAAUUAAUUCCCGGAAUAAUCUUCUCAAUAGUUCUCGUCAUGGAGAAUCGCUUAAUAGUCUUAUUAUAAAGACCUGCCGGUGUUAAUUCGAAUAAAUCUGCUGCAAUUUGCGGGUGUAGAGGUUUCAUCUUCGUUAAUAAAGAUUUAUAAUUCUCUCCUUGUUGCCUCAGAUCGUUCGAAAUCAACUGUUUGAUAUCCUUGUUAUUCACUAUCCCAGGUAAUGCUUUAGAUACAGCAUUAGAUAUCAAAUGAGAUGCAUCAUUUGGUUUUUCAAUCGGAAUAGAAUGCGGAUCAUUUAUUAAAUUAGUUGCAUCAAUUUUGUGCGGUACAAAUUCUUGAGUUCUAAGAAGCCACAUAUAGUUUCUUAUUAUUGGUUCCGUUUGAGACAAAUAGUAUGUGGCUGCAACAUCGAAAGAUAGAUCGUCUGACUCUCCUUUACUGAAAUACCGAGUCCAAGGCAUCAUAGGUAGUCCGCCUAAUGAUCCUGGGAUUAAAAGCACUUUUCGAGACGUGGCAGAUCGUAAUAAUCGUAAAAUGCCUUUAUGUUCAAGAAUAUAAAGCGGCGAAGCUAAUCUCCGACGUAAUAAUAAAAUUACUUGAAUAUGUUUCCACCAAACGGCACGGAAAGAAUUAUGAAGUGUUCCAGCUACUGCAACAGAAUUCGCAACAACUCCAGAUAUUUCCUUACUCAGAGAAGGAAUCGUAUAAUCCAAUCUCGAAAACGCUCUAGAUGAAAAUUUGAGACUAUACAUAGUAUGAACUCCACGUAUAUAAAUUUCUUUUCCAUAUGUCAAGACGGUUCGGGAAUCAAUACACUCUUCCGGUUUUACUUCAUGAUUUAAUCGCUCACAUCUCCGUUCUACCGAGUAAAGAAAAGAUUGUAGAGCAAGGCUGAGGGACUGUUUUUUUGUGUUAAAUGAUAAAAAGAAGACUUGAUUAUCUCCUUGCCCUGCCAUUUGGAAAGAACACUGUUCGUCUUGAAUUGCAUAAUACAUCAUAGCCAAUGUACAAAUUGUCCAAAGUGUUUGUUGAAUUCCUUCAAAACCCCCUAGAUGAUUUCUCCAUACUAAAUUUGAUUCCGGCCAAGUAGAAGCAUGUGAAUUAGGUGUGACACCUGGAGGGAGAGUAUUUUUGUCUGUCAUGACAAUAGUCGAUUGUGUGAAAAAAGAAUGGGCUUGAGAAAAUACACCCGGUAAUCCAAAUAUUUUUUCCAGUGAUCUCGAAAUAGGAUUAACAGAACAUGCUCUCCAUCUUAGAUUCCAUCUCGAGAAAUCCACCUCUACAAGACAAGUAUUAGUCCUAUUAUUAACAGCAGUUAAGUCAUAAAGUCUUUUCUUCAAUUUUGUAUUUGUCAUGGUCAUGGUUUGUUGUGGUAGAUAUCCAUUGUCACCUGAAUCACCUCCCAUAAAUCUCUUAAGAUUGGUUUCUGUCAAAACAAAGAAUAGACGAACUUCGAAUGUUAGUUUACAGAAACAUCGAGCAGAGGUUUUGAACUCGCGUUCUUUUUGAGUCAGUUCCACAAUUCUUUCAUCUAUAUGAAACUUACCUUUUCUCAUACGCUCAACAAUUUGAAACAUAUCCACAUCUGCCCUUUUGAUUAAUGAUUCUAGAAGUCGACGAUAGCUUUCCCCUGAUUGCUUAAACCAAAACCCAGAUGAUUUAUCUGCUCCUGGACAUAUUGCUUUAUCGUCGAUCAUAUCAAGAUAGUCGGGAGAAUAAUCAAAUUCCAUGAAUUUUGGGAACUCAACAUCAUAUAGAUCCGAUAGCGGAUAACUUGAAUCGUUGAUCUUCGUUAUUCCUGACUUCCAUAAAUUAUAGAGUUUGGUUUUUUUCUUAGGACCAUCUUUAUCCGUGAAUGCUGGCCAUACCUGGUUCUUUUUUAGAUAACGAUCUAGAACUAGUCUUUUAAAGUGUGCAUGAUAAUCUCGUAUUGCUAAUAGAUCUAAAUUACCAGUAGGACAUGCUUCUUUCUUUACUGAUAUAGAUGAAGUUUCAGCAUACACAAAUGGAUGACCUGACAUCUUAGUACACCCAAAUAAUUCUGUUACUAUUGAGAUAUCAUUUGUAGACUCAAUAAGGUCUAUUAAUUGAGAGGUUAAUGGACAUUCGUUUGUUUUGGAUAAUUUUUUCUCUUUUUCUUUGAGUUUUGCACAAGUACGUACAAAUGAGCUUAAAGGUAGUACGUCACCUUGAGUUAACGAUGUUAGAUACGACUUAGUUACCGAUUCAGGACCUUUUACGAGUUCGUACCCUUUGUUUCCAUACUUCUUAAGUACAGCCUCUUGCCAUAAUAAUAAUUUUCUCAAGAAUUCACGAAGCUGUUGGUUAGAGUUAUGCAUAUUUGCGUCUAGCGCAAGAAAUGCAUUAAAGCGAGCCAAACAUACAUCUUGAAUCAUUUGAAGCUGCUCAAAUGAAUAUAACCUUCGAAUUGGUGCCAAUUUCUUCGGCUUACCAUUUUCAUCAACAAUCAUCCUACUGAAACCUAUUUGUUCCAUAACAAACCCAUCAUAAAAGAAAAAAAUAGAAUCAUUAAUGGUUAGUCCUUGUCUGCCAAAUUGUUUCUUCUGCUUCCGCUGUCGAUACUUUUCUACCAAGAAAUCCCAAUACCGAUAGGAUUCGUAAUGAAGAUACGUAUCCUCUUUUGCUUCAGGUAUAAAUUUCUCCUCAUUUUCUAUGAAUGUUUUAACUUCCGGUAAGGUUCCCCAUUUUUUGAAGGCACAAAGUUCUGAUCGAUAUAAUUCUGAUGAUUCCGCUAAUGAAGUUUUCAAGCUUUCUCGUGCUAUUAGUGUACGUGUAAAUAAAUCGGGGUAUUGAUCAGGAGUCACUAUUUGAGGAGCCGUAGAAGUCCCGAUUAAAUCAAAUAACCAAGGAGUUGCUACACGUUCGAGACCGGAAUAGGAAUGAAUUAUAUUAUGUUUACAGAUAUUUUUUAUAAUCACCGGAACAUCUGACUUGAUAUAAGGAAAGUUACUCUUUGCGAAACGAGUUUGCUCUUUAUAAUGUAAUAUCUCCUCAUAAAGCAAUCUCAACCUAUCCAUAAGAGAGGAGAGAAUAGGACUACGAAGGUGCUUCUCUGCUGGAGCAGCAGAGCGACGCUCCCGAAUCGAAUCAAAUUCAAAUGCCUCACUGAAAUUCAUAUUGCUGAUGUAAAUGAAGUGAUCUCCUAAGUUUUAUUAAAUAAUGUAUGUACGUUAUAGUUCUAGUAAGUAUUAUACAUCUGUGUAUUGAUCAUUUGAAAUCAUACUCUUUAACCCGAAAUUAUUUCAAUUUCGUCGAAUUCUUCGUCUUCUGUAACUAAAGAGAACUCACUUGAUGAUUCAAUUAAGUUUGUUUCCCAUACUCUUUUAGUCAGAUUCUCUAAUAAGACUAACGCGCAUACAAAAGCAUAUAAGUCGAACUGUGGUUGACUAUCGAGUAUAUCCAUUCUUGUUAUAUGACUCUGAGUCUUACGUAAAUAUGGAUGUCUCCUAAGUUUUAUUAAAUAUCACAAAAGAAAAUCUCAAGCUAGAAAUCAUUAGGUUCAUUUCCCUACAUUCGGAUUAACCAUGACAAACAUAAAGAUUAUUAAUGUAGUAGUUGAGAGUAUUCGGAUAUGCAGUAUUUGCCGAAGCGAUUGCAGUGCAUGUAAUAUCAUAACGAGUAUGAUUGAUUCGAUACUCUCUUCCAUCAGUAGGAGCAGUACAAAUAUUCUGACGAAUGAACUCAGCUUGAUCACAGAUAAUUGAUUCAGAUAGUUCACGAUGAUUCAUAUUCAAACUAUUCUCCACUCGGAAUUUUUCCCACUGGAAGUUUGGACGAUCAAUAGAAAUUGCAUCUAUCAUGCCCUGACUAAUCGCGGGAGAGAAGAAAGCGCGGUUCGGAAUAAUAUCAGAAGGUGGAGAUUUCCGAAUUCUGAAUAUAAUGACAUCUGGUUCGGUCACAUUAGUACUUCCAGAUCCGAAUGAUCCUGAAUCAGACAUAUGGAGUCCAUACUUAUUGUUCAAGAAGACAUAGAGACCUGGUGCUUUUUCCUUAUUUUUAUCUAAAGAUAAUGUUACCGAAAAAGGCGAGCUCUCAAUACUUAACACGUAAUGCCCUUUAGCUGGACACAUACAGACACGUAACUUCGUCUCCAUGAGAAGUUUGGCUAUAAUCUCCCAAGAAAGCUCGGUUUUAUCCUGCAUGUUGAGGAUUAAAGCAUUUUGGAAAAAUCCGAUUUUCUUAUCUAAGGUACGAUGUAGCGAAGUUGUAGCAUUUAUUUGUUUCUGUAGGCAAUCCAAUUCACCAGAAGCAAUAACUCUUAACUCAACUGAGCUUGAGAAUUUGCAGAGGACUGAAAUAUCUUGACAAUUAUCUAACAAAGUCUGCAUAUUGAUGGAAACGACUUAUAUGUUUCUCCUAAGUUUUAUUAAAUAAUGCGAAAGAUCAUUCAUACGAAUAAGUGAGGAUUGAGAGUCAAGCUGGUCUUAUUGGGCAGGAGCUUCUGGAACAGCUUCUUCGGCGACUUCUCGAAUGUCGAGAAGAGCGAUAAGCUUCUUUCUAUGCUGAACACUCAAAGUUCCUCCAGCAUAUUGACCAAGAGUGGAUUCUUGUUGGGCCAAUGCAAAUACGGCUACACCAAGAAGCUCAUUGAUAUCAUUCCGAUCAAUAGGAAUAUAUGCGCUCCCGAAGAUAACCUUAAGAUACAUUCGACGUUUUGUAGAAGUAUCGAGAAAUCGUUGGAGAGCAUGAGCAGCAGCGAAAUAUUCAGUCUCAAGAUCACGAAACUCUUUGAAGAAUUGAGGAUAUUUCCUGGCAUACUUAGUAAUCAAUAAGAGAGGACUAAGUGAUGCGCCGAAAGUGAGUCGAAACAUCACAUAGAACAUUCUUUGAGUUCCGGACACCAUCGGGUCGUCUUUAUCAACAAUAGAACUCAUUACGAGAGCACGAUCGCGAAUGAUCGAGUUGAAUGUCUUGUUGACUUUGCUCAGAACUGCGUGUGACAAAUUACGUUAUACUAGGUGAUGAUGUUGUGAUAAGGACCAAGAAGCUAGCCUCUGAGUAUAAGAAGAUAAUGGACGAUUUAGGAGUAGACAUAUCUGAUAGCAAAUCUCAUCAAUCCAAAGGAUAUUUUGAGUUUGCCAAGACAUGGAUACACCAAGGUCGUCCAGCUAGCGGUUUUCCGAUCAAAGGAGUCUAUUCCACAAUAGGCCGGAUAUCAGAAUUGAUACCGGUCCUAGUGGAUAUAGCUCCUAUGAGAGGUUACGCACUUCCUUUCUCAGUAGGGAACCUCUGGAGUUUCUCACAAGAUUGAGGUAAUCUGAUGACCGGGUAUAAACGCCAUAGAAUUAAUCUAUCGGCAAAAUUAUAUCGGUCAAUCCUCUUCAUCUUGACUAUUAAACAGGUUGAUAGUUAUUGGUCUGGAUUUUUCAUCCAGGCAAUCACUAAUAGCCUGCUCCGUCCUCACGACGCGGCAUUGGUGUUCAAAAGAGCACUGAUGGCGGUCAUAAAGGAAGGAAAACAGUCAGAGUUGGAGAGGUUGGAGUUAUUCGCGGAAGGUCUUAUGACUUCACUUGACUCAAAUUGAGCCGAGAUGGAGCAAGAGACCUACGAGAAUCACUCCUCAGAAAACCCUAAUGAUGAAAUAGUACUCCAUUCCGUCGAACAAGAACUCGACAUGGUUGCUUCACUCCCUAUCCUGGCCUCCCUAGAAGUAGAGAGGACCGAGAGAGAGGAGUGAUUAAACUACUUUACGGUAGAUGAUCCUGAAGAGUUUUGAGAAAAGUUCAAAUCGUGAGAUUUGGCACCUUUCCCUCAACUUAAAGGACUUCAACCGCAAAGAGUAAAGACCAGGUCGCAAGCCGAACUUUCCUUGGCAUCCAAAGUAAUAAAAUUACUGCGGAUGGUCGGUUAGCCUAGCUUGUGAAGCAAAGUCAGAAAGGGGUGAAAAUCCCUACCUGUCCUUGCUCUGAGAACUU  >Ss-AA_clean.1_(paired)_contig_981  UCGGGGGAGUUUGAAACGAAUAUUGCAUAAUAUGCGUCAGUUAUAAUAUUCCAACAGAUCGCAUGUUCAACGAUAUUAGGAUCGACUCGUGAAAUUUCUUUAAAAUCGAAGAUAUCUGCUGUUCCAAGUAUCCCGUCAUAAGGUUUCUUCUGGUCAGCACUAGCAGAAACACAUGAUUCUAUAUAAGAGUAAAUUAAGUCUAGAUCUGAAGGUGGUGAUGAGAUGUGUGGUAUUACUGAAGGGAGUGGAAUUUCGGGUAUUUCAGACGCAAAGAUCUCCCCCACCCAAGCUAAUUUGUUAGACGUGAGAUCCGGCCAUUUAAUUUCUAUAGCCUGCUCUGAAGGAAUCUUAGAAACUGAAGUAUUAAUUUCACGUAAUUGGCUUGGGAUAUAAUAGCAGAUAUUAGGAGGAAGUAUAGUGUUACCGGUAGACAAUAUUUGAUACAAAUUAGUUAAUGUCAUGUAAAGAGUCUGAAACACCACUGGAUAAUCUGCAUCUCCACCUGAUAAAAUACCCGCUUGGUCUGAGCUAAAGAUAAUGUGCGUGGGCACAGAAGAACUCCCUAAAACGGAGAAUACAUGAGAUGUACUCUUNNNNNNNNNNNNNNNNGGUUUUAAUGAGGGAUCUCCUUGUAAUUCGGAAUAUGUGAGUACUGCAUUCUUCAAAUCUUUCAUAGUUGAAUUCGUACUUACAAUGCGAUACCCGUGAUCAGAUAAUUUUUGUUUUGUUGAAGUUCCGAAAUUAGGAGGUGCUCGACCGCAGGUAGUCAAUAUGUCUUUUCUAUUUUCAGUUGUCGCACUAAUUAGUGGUAUUCCAGGUUUGAAAUAGCCAAUUUCAAAAUCAAAAGGAGUAUAGACUCCGAUCGAUGUGUUAUCUAAUCCAAGAUUCCAAUGAGUCCUUAACUCAGUAGCCAUGUCAAAGGGGGGUGGAUGACGUCCUCCAGACUUCUUAGCAGCUAACACCCAUCGAUCAAGAAGAACAGACAAAAUUGUGGAAUUUGCUUGAGAGAUUUUAUCGAUGAAAUUAAUUCCUGGGAUAAUCUUUUCAAUAGUUCUUGUCAUGGAGAAUCUCUUAAUAGUCUUAUUAUAAAGACCAGCCGGUGUUAACUCAAAUAAAUCUGCCGCAAUUUGCGGGUGCAGAGGUUUCAUUUUUGUCAAUAAAGAUUUAUANNNNNNNNNNNNNNNNNNNGAUACAGCAUUAGAUAUCAAGUGAGACGCAUCAUUUGGUUUCUCAAUUGGGAUGGAAUGCGGAUCAUUUAUUAGAUUAGUUGCAUCAAUUUUGUGCGGUACAAAUUCUUGAGUUCUCAGAAGCCACAUGUAGUUUCUUACAAUUGGUUCUGUUUGAGAUAAGUAGUAUGUAGCUGCAACAUCAAAAGA  >Ss-AA_clean.1_(paired)_contig_1357  CGAGCAGAGGUUUUAAAUUCACGUUCCUUUUGAGUCAGUUCGACGAUUCUUUCAUCUAUAUGGAAUUUACCUUUCCUCAUCCGCUCGACAAUUUGGAAAGUAUCUACAUCUGCUCGUUUAAUUAAUGAUUCUAGAAGGCGACGGUAACUUUCUCCAGAUUGUUUAAACCAGAACCCGGAUGAUUUAUCUGCCCCUGGACAUAUUGCUUUAUCGUCAAUCAUAUCAAGAUAAUCAGGAGAAUAAUCAAAUUCCAUAAACUUUGGGAAUUCAAUAUCAUAGAGAUCCGAAAGCGGGUAACUUGCAUCGUUGAUCUUUGUUAUUCCCGAUUUCCACAAGUUGUAGAGUUUAGUGUUCUUCUUAGGGCCAUCUUUAUCCGUGAAGGCCGGCCAUACCUGGUUCUUUUUGAGAUAACGAUCUAGAACUAGUCUUUUAAAGUGUGCAUGAUAAUCUCGGAUUGCCAAUAGAUCUAAGUUACCAGUAGGACAUGCUUCCUUCUUGACUGAAAUAGAUGAAAUCUCAGCGUAUACAAAUGGAUGACCUGACAUUUUAGUACAUCCAAAUAGUUCUGUUACUAUUGAAAUAUCGUUCGUAGAUUCGAUAAGUUCUAUUAAUUGAGAGGUUAAUGGACAUUCAUUUGUCUUUGACAAUUUUUUCUCUUUUUCUUGAAGUUUUGCACAUGUACGUACGAAUGAACUUAAAGGUAGUACGU  >Ss-AA_clean.1_(paired)_contig_1426  GUCCAAAGUGUUUGUUGAAUUCCUUCGAAGCCUCCUAGAUGAUUUCUCCAUACUAAAUUUGACUCUGGCCAAGUAGAGGCAUGUGAAUUUGGUGUGACACCUGGAGGAAGAGUAUUUUUAUCCGUCAUAACAAUAGUUGAUUGUGUGAAGAAGGAGUGCGCUUGUGAGAACACACCUGGUAGCCCGAAUAUUUUUUCUAGGGAUCUAGAAAUAGGAUUAACGGAACAUGCACGCCAUCUUAGGUUCCAUCGCGAAAAAUCCACUUCCACUAGACAAGUAUUAGUUCUAUUAUUGACAGCUGUUAAAUCAUAAAGUCUUUUCUUCAACUUGGUGUUAGUCAUGGUCAUAGUCUGUUGUGGUAAGUAUCCAUUAUCACCUGAAUCACCUCCCAUAAAUCUCUUAAGAUUGGUUUCGGUUAAGACAAAGAACAAACGUACUUCGAACGUUAGCUUACAGAAACAUCGAGCAGAAGUUUUAAAUUCGCGUUCUUUUUGAGUUAAUUCUACAAUUCUUUCAUCUAUAUGAAAUUUACCUUUUCUCAUACGCUCGACGAUUUGGAAUGUAUCCACAUCUGCUCUCUUAAUUAAUGACUCUAGAAGUCGACGAUGACUUUCCCCUGAUUGCUUAAACCAAAACCCAGAUGAUUUAUCAGCUCCUGGACAUAUCGCUUUAUCGUCAAUCAUAUCAAGAUAAUCGGGAGAGUAAUCAAACUCCAUAAAUUUCGGGAACUCGACAUCAUAGAGAUCCGAUAGAGGAUAACUCGCAUCGUUGAUCUUAGUUAUUCCUGACUUCCACAAAUUGUAGAGUUUGGUAUUUUUCUUCGGACCAUCUUUAUCAGUGAAUGCUGGCCAUACCUGGUUCUUUUUGAGAUAGCGAUCUAGAACUAGUCGUUUGAAGUGUGCAUGAUAAUCUCGUAUUGCUAAUAGAUCUAAAUUACCAGUAGGACAUGCUUCUUUCUUUACUGAGAUGGACGAAGUCUCAGCAUAAACAAAGGGAUGACCUGACAUUUUAGUACACCCAAAUAGUUCUGUUACUAUUGAUAUAUCAUUUGUAGAUUCAAUAAGUUCUAUUAAUUGAGAAGUUAAUGGACACUCUUUCGUUUUUGAUAAUUUUUUCUCUUUUUCUUGAAGUUUUGCGCACGUACGCACGAAUGAGCUUAAAGGUAGUACAUCGCCUUGAGUUAACGAUGUAAGAUACGACUUAGUUACUGAUUCAGGCCCUUUUACGAGUUCGUACCCUUUGUUUCCGUACAUCUUAAGUACAGCCUCUUGCCAUAACAAUAAUUUUUUCAAGAAUUCGCGGAGCUGUUGAUUGGAAUUAUGCAUAUUUGCAUCUAACGCAAGAAACGCGUUAAAACGAGCUAAACAUACGUCUUGAAUCAUUUGAAGCUGCUCGAAUGAGUAUAAUCUUCGGACUGGUGCUAACUUCUUCGGCUUUCCAUUUUCAUCGGUAAUCAUCUUACUAAAACCUAUUUGUUCCAUAACAAACCCAUCAUAAAAAAAGAAAAUAGAAUCGUUAAUAGUGAGUCCCUGUUUACCGAAUUGUUUUUUCUGUUUUCGCUGUCGAUAUUUUUCUACUAAGAAAUCCCAGUACCGAUAAGAUUCAUAAUGGAGAUACGUGUCCUCUUUCGCUUCUGGUACAAAUUUCUCUUCAUUUUCUAUGAAUGUCUGAACUUCUGGUAAGGUUCCCCAUCUUUUGAAAGCACAGAGCUCCGAGCGAUAUAACUCUGAUGAUUCUGCUAAUGAAGUUUUCAAACUUUCUCGUGUUAUGAGCGUGCGUGUAAAUAAAUCAGGGUAUUGAUCUGGGGUUACUAUUUGAGGAGCUGUAGAGGUCCCUAUUAAAUCCAAUAUCCAAGGAGUUGCUAUGCGUUCGAGGCCAGAAUAGGAAUGCAGCAUAUCAUGCUUGCAGAUAUUUCUUAUUAUUACCGGAACAUCCGAUUUGAUAUAAGGAAAAUUACUUUUCGGAAAACGAUUGUGCUCUUUGUAAUGUAAUAUCUCAUUAUAAAGUAAUCUUAAUCUAUCCAUAAGAGAAGAUAGAAUAGGACUACGAAGGUGCUUUUCAGCUGGAGCAGCAGAACGACGCUCUCGAAUUGAAUCAAACUCAAAUGCCUCACUGAAAUUCAUAAUGCUGAUGUAAGUGAAAUGAUCUCCUAAGUUUUAUUAAAUAAUGUAUGUGCAGUAGAAGUCUAGUGAGUAUUAUACAUUCGUAUUUCCAUUUUCUGCAACUCUACCUUCUAACCCGAAAUUAUUUCAAUUUCGUCGAACUCUUCAUCUUCUGUAACCAAGGAAAACUCACUUGAUGAUUCAAUCAAGUUUGUUUCCCACACUCUCUUAGUUAGAUUUUCCAAUAAUACUAACGCGCAUACGAAAGCAUAUAGAUCGAACUGCGGUUGACUAUCAAGUAUAUCCAUUCUAGUCACGUAAUUCUGAUUCUUAUGUUAAUAUGGAUGUCUCCUAAGUUUUAUUAAAUAAUCACUGAAGAAGAUUUCAAAUUAGUAGUUUCUGGGUUCCCUUUCCUACACCUAAAUUAACCGUGACAAAUAUAAAGAUUAUUGAUGUAGUAGUUGAGAGUAUUCGGAGAUGCCGUGUUUGCCGAAGCGAUUGCAGUGCAUGUAAUAUCAUAACGAGUAUGAUUGAUUCGAUAUUCUCUUCCAUCUGUUGGAGCCGUACAAAUAUCUCGACGAAUGAACUCAGCUUGAUCACAUAUAAUAGAUUCAGAUAGGUAACGAUGAUUAGUAUUCAAUCUAUCUUCCACUCUGAACUUUUCCCACUGAAAACUUGGGCGAUCAAUAGAGAUUGCAUCGAUCAUGUCUUGACUAAUUGUACGAGAAAAGAAGUCGCGAUUCGGAAUUAUAUCAGAAGGUGGGGACUUCCGAAUUCUGAAUAUAAUGACAUCUGGUUCAGUCAAAUUAGUACUGCCGGAUCCAAGCGAUCCCGAAUCUGACAUAUGGAGUCCGUACUUAUUGUUCAAGAAGACAUAAAGACCUGGCAUUUCUUCCUUACUCUUAUCUAAAGCUAAUGUUACCGAAAAAGGCGAACUCUUAAUCGAUAAUAAGUAAUGCCCCUUAGCUGGACACAUGCAGACACGCAAUUUCGUUUCCAUGAGAAGUUUAGCUAAAAUCUCCCAAGAAAGCUCGGUUUUAUCUUGCAUGUUGAGGAUUAAAGCAUUUUGGAAAAAUCCAAUUUUCUUAUCCAAUGUGCGAUGAAGAGAAGUUGUGGCAUUUAUUUGCGCCUGUAGACUCUCUAAUUCUCCAGAACCAACAACUUGUACCUCAAUUGAACUUGAAAAUUUGCAGAGGACUGAAAUAUCCUGAUAAUUCGCUCGGAGGGCCGACAUAUUGGUGAAAACAAUUUAUGUGUAACUCCUAAGUUUUAUUAAAUAAUAUGAAAAAACAUUCGUUGAAAAGAUGGAAAAAACAUAUCAGAGUUGAGAUUAUUGAGCAGGAGCUUCGGGGACAGCUUCUUCGGCAACUUCUCGAAUAUCGAGAAGAGCGAUGAGUUUCUUUCUAUGCUGAACACUGAGAGUUCCUCCUGCAUAUUGACCAAGAGUAGAUUCUUGUUGAGCCAAGGCAAAUACAGCAACGCCGAGAAGCUCAUUUAUAUCAUUCCUGUCAAUAGGAAUAUAUGCGCUCCCGAAUAUAACCUUGAGAUACAUUCUACGUUUGGUAGAAGUAUCGAGAAAUCGUUGGAGAGCAUGAGCAGCAGCAAAAUAUUCCGUCUCAAGAUCAGGAAACUCUUUGAAGAAUUGAGGAUAUUUUCUGGCAUACUUAGUAAUCAAUAAGAGAGGACUAAGUGAUGCACCGAAAGUGAGUCGAAACAUCACAUAGAACAUUCGCUGAGUCCCGGACACCAUUGGAUCAUCUUUAUCAACAAUAGAACUCAUUACGAGAGCACGAUCGCGAAUGAUAGAAUUGAAUGUCUUGUUGACUUUGCUCAGAACUGCAUGUGAUAAAUAUGGAGAAUUAUCAGUGAAAACCUUAAGCUCAC  >Ss-AA_clean.1_(paired)_contig_1219  UACGAUCUGCAACUUCUCGAAAAGUGCUUUUAAUUCCAGGAUCUUUAGGUGGACUGCCUAUGAUGCCAUGGACACGGUAUUUCAGUAAAGGAGAGUCGGAUGAUUUGUCGUUUGAUGUUGCAGCCACAUAUUACUUAUCUCAAACAGAACCAAUUGUAAGAAACUACAUGUGGCUUCUUAGGACUCAAGAGUUUGUACCGCACAAAAUUGAUGCAACUAAUCUAAUAAAUGAUCCACAUUCGAUUCCAAUUGA  >Ss-AA_clean.1_(paired)_contig_344  AUGGCACAUACACUAUUAUCGACUAGAGGAAUGAGUAGUAUACCCCGAUUAAUACUUUUUAGUAACUGGAAGGAAUCUUCAGCUAGAUUAGGAAGAAGACGACUAAUUCUAGCGCACGCAAUUGCUACAUACCCUAUUCAAUCGCUUACAAGUCUUAGAAAAUCGGUAUUAAGUAUCGAACCUCCUACGGAACUUUUAAAACAAGAUCCGGCUACAUAUAUCUCAGUUGUAACACGAAAAAUUUCUCGGAAGAUAUCGAACUUAGAAUACACGCUCCCUGAUAUGCCAUGCUUAUUUUUGCACCGUACCGAUAAAGAAGCUAUGCGUAUGCUUAGAGAUAGGGGGAAGUUCCAAGACAAAAAAGAUACAAUAACGCAUCCCCUCAGAUUCUCGAAUCAUGGAAUCUGCAAAGUAGAAUAUUCAGAACAUUAUGGAUCUCUUGAACCGAAUGAUGUAGUAUUGAAACAAGAACGUAGUAUUAGUGAUCGACUUCGAAUACUUCGCCGUAGAACAAUAGGAAUCUAUUCUCCUCUUUAUAGCGAUUGGAAUGCAAUAUUCGAGAUAUUAUUCAAUCGUGGACUAGACAAGCAACGGAAAUUCCAUGUUAUUGGUGUCGGAAGAGGUGCUACAUCCCGUGCUCUAGUUGAUAGACAUGUAGGAGUUAUAGGAUACGAUUUAAUUUCGUCUUUCCCAUCAAUAUCACAUAGAUCUGCCUCAUAUAAACCCCCGGAACUAAUUAUGUCGGCUAAUACCGAAAACUUCUCGUGGAGUAAUCAUACUUACUCAGAUGAUGGAGAUGUUUUAAAAGGAAGAUUGGAUUGUUUCGAUGAACGUAGGCCAAUGUUGGUAAUUGACUUAGACAUUUCUUUCCAAAAUUUAAAAACAUUGCUUAUGAGAUUACCAAUCAAUUCAGAAGUGAUUGUGAGAUACGUAGGAAAUGAAGAUGAAAUCCGAUGUCUAAUAAGUAUGAUACGUCCCACUAUUAUUAUCUCUCUCUGUAUUUCGGAUAAUAAAGUAUCAGAUAUUGUCCUAUAUGCAACUCAACUGGGAUCCGUUGGAGUAGGUAACUAUCAAAGUGUCGAAUUCAAAACCAUAAAUGAAAUUAGUUAUUCGUAUGGAUCUACGGAACUAGUUUCGCAAUUCUGGAAUGUUGAACCCAAUAUUUCUCGACAUUUCCAAUUAGAUGUAACCGAUACAGUACUCUCGUUACGUACAAAAUUAUCUGCUUUAUACGAGGCAAGACGAACGGAAGUAUCUGAAAUUUACACCCAUUUGUGGGAUAUUAUGCAUGCAAAUUUAACUGAUACGAAUAUGACAGGAAAAGAUCUUAGAUGUCGAGCCAUUUGUGAUAAUAUUUUCACAUAUGAUACUUAAUAUAUAAUUCUAAUGUGUUCAUUUAAUAAAACUUAGGAGAAUGCCUUCGUAUAAAGAGAAGUAUACGCAUAUCUGUAAUAGGUUAACGACAAUGGAAAGAAGAGUCUUACUCGACGAAACCAUAUUACUUGAGUUACGAAAAACAGUUCUAGAUCUUCAUGCUAAGUUGAAUGCUCCUCCCAAAGAGGUCGAUGAAUUUAUGUUGAAUCAAUUACAUUUAGCUCUGACGGAAUUAGUCAAAUAUGCUAGUCCUCAAUUUCAACUCCCAGCGCAUUUUAAAGGAAAGGAAAUUGAUGAAAUUCUGGCUACAACAACUGAAAUGAUAUUUAAUGUAGUAUCUUUGUUAAGAGAUGCAACAGUCAGAGCUCAAACAAUAUUAUCAGAUAUCUCCCCUAAACAAUCUAACGAGGACUCAUCUUCUGGAAAGAAUAACAUCGAACAGCGUAGUUCUCUGUCAAAUUCCCCGAGUUUGACUAAAAUUGAAAGUCAAGGGAAUAGACGCAGCUUUGCCAUGACACCCCAAGCGUUAUAGUCGAGUCUCAUUUAAAUCUAUUUAACAAAACUUAAUUAUUCCAGAAGAGGACAAUAUUUAUAACAAAGCUACGGGAGUACGAUC  >Ss-AA_clean.1_(paired)_contig_1352  GAAAAUUAUUAUUAUGGCAAGAGGCGGUACUUAAGAAGUAUGGAAACAAAGGGUAUGAACUCGUGAAAGGUCCUGAAUCAGUAACAAAGUCGUAUCUUACGUCUUUAACUCAAGGUGAUGUACUACCUUUAAGCUCAUUUGUACGUACGUGUGCAAAACUUCAAGAAAAAGAGAAAAAAUUGUCAAAAACAAAUGAAUGUCCUUUGACCUCUCAAUUGAUAGAACUUAUCGAAUCUACGAAUGAUAUUUCAAUCGUAACAGAACUAUUCGGGUGCACUAAAAUGUCGGGCCAUCCCUUUGUUUACGCUGAGAUUUCAUCUAUUUCAGUCAAGAAGGAAGCAUGUCCUACUGGUAACUUAGAUCUAUUGGCAAUCCGAGAUUAUCAUGCACACUUUAAAAGACUAGUUCUAGAUCGUUA  >Ss-AA_clean.1_(paired)_contig_1912  UACACUUUGAUAAUUCCCUGCUCCGAUAGACCCCAAUUGAGUAGCAUAUAGAACAAUGUCAGAUACUUUAUUAUCUGAAAUACAGAGAGAAAUAAUAAUAGUAGGGUGUAUCAUACUUAUUAGGCAUCGUAUUUCAUCCUCGUUUCCUACAUACCUGAUGAUCACUUCAGAAUCGAUAGGUAAUCUAAGAAGCAGUGUCUUUAAUUUUUUGAAACAAAUGUCUAAAUCUAUUACUAAUAUCGGCCUAAUGUCAUUGAAACAAUCGAGUUCACCUUGUAAGACAUCUCCAUCAUCUGAGUAAGUAUGAUUGCUCCAUGAGAAAUCCCCAGUAUUGGCCGACAUGAUUAAUUCUGGAGGUUUAUACGAGGCAGAA  >Ss-AA_clean.1_(paired)_contig_2096  CUCAUGUAUUCUCCGUUUUAGGAAGUUCUUCUGUGCCCACGCAUAUUAUCUUUAGCUCUGACCAAGCGGGUAUUUUAUCAGGUGGAGAUGCAGAUUAUCCAGUGGUGUUUCAGACUCUUUACAUGACAUUAACUAAUUUGUAUCAAAUAUUGUCUACUGGUACCUCUAUACUUCCUCCUAAUAUUUGCUAUUAUAUCCCAAGUCAAUUACGUGAAAUUAAUACUUCGGUUUCUAAGAUUCCCUCAGAACAGAUUGUAGAAAUAAAAUGGCCGGAUCUUACGUCUAACAAAUUAGCAUGGGUAGGAGAGAUCUUUGCGUCUGAAAUACCUGAAAUUCCACUCCCUUCAGUAAUAUCACAUAUCUCAUCACCCACGUCGGAUUUAGACUUGAUUUACUCUUAUAUUGAAUCAUGUGUUUCUUCUAGUGCUGACCA  >Ss-AA_clean.1_(paired)_contig_2204  UGUAGUCUGAUCCUUACCCAUUCUCCAGAAUAAUAAGUAGCUUUUGUUUUUAAGAUUUUCUUGAAGAGUGGUAGAGAUUUGUCUGGGGAAUUUGAAACAAAUAUUGCAUAAUAUGCAUCGGUUAUAAUAUUCCAACAGAUCGCUUGUUCCACGAUAUUAGGGUCUAUCCGCGAAAUUUCUUUGAAAUCAAAAAUAUCUGCUGUUCCUAGUAUUCCAUNNUCAUAAGGUUUCUUCUGGUCAGCACUAGUAGAAACGCAUGAUUCUAUAUAAGAAUAAAUCAAGUCUAAAUCAGAUGUUGGUGAUGAGAUGUGCGGUAUAACUGAGGGAAGCGGAAUUUCAGGUAUUUCAGACGCAAAGAUCUCUCCCACCCAGGCUAAUUUGUUAGAUGUAAGGUCCGGCCAUUUAACUUCUAUAGCUUGCUCCGAAGGAAUCUUAGACACAGAGGUAUUAAUUUCACGUAAUUGACUCGGGAUAUAAUAGCAGAUAUUAGGAGGGAGUAUAACAUUAUUGGUAGACAAUAUUUGAUACAAAUUAGUUAAUGUCAUGUAAAGAGUUUGAAAUACUACCGGAUAAUCUGCAUCUCCGCCCGAUAAGAUACCUGCUUGGUCAGAGCUGAAGAUAAU  >Ss-AA_clean.1_(paired)_contig_3112  UCGCUUUAUCGUCAAUCAUAUCAAGAUAAUCAGGAGAAUAAUCAAAUUCCAUAAAUUUUGGGAAUUCGACAUCAUAGAUAUCUGAGAGCGGGUAACUUGCAUCAUUGAUCUUUGUUAUUCCCGAUUUCCACAAGUUGUAGAGUUUGGUGUUUUUCUUAGGGCCAUCUUUAUCAGUGAAGGCCGGCCAUACCUGGUUCUUUUUGAGGUAACGAUCUAGAACUAGUCUUUUAAAGUGUGCAUGAUAAUCUCGGAUUGCCAAUAGAUCUAAGUUACCAGUAGGACAUGCUUCCUUCUUGACUGAAAUAGAUGAAAUCUCAGCGUAAACAAAUGGAUGACCUGACAUUUUAGUACAUCCAAAUAGCUCUGUUACUAUUGAAAUGUCGUUCGUAGAUUCGAUAAGUUCUAUUAAUUGAGAGGUUAAUGGACAUUCAUUUGUUUUUGACAAUUUUUUCUCUUUUUCUUGAAGUUUUGCACAUGUACGUACAAAUGAACUUAAAGGUAGUAC  >Ss-AA_clean.1_(paired)_contig_4528  UCAAGAAAAUCUUAAAGACAAAGGCUACUUAUUAUUCUGGAGAGUGGGUAAGAAUCAGACUACAUCCGAUGUUUUCUACUACACAAUACAAUCAACGUCGUAGAAUAAAUCUUCAACCUACUCAAUCCGGGUAUAAACGACCUGUUGAUUAUAUGGCUACUCAGUUCCUGCGUAUGGCACAUACACUAUUAUCGACCCGAGGAAUGAGUAGUAUACCUCGA  >FirsU_Contig247  UAUUCGUUUCAAACUCCCCCGACAAAUCGCUACCACUAUUCAAGAAAAUCUUAAAGACAANAAGCUACUUACUAUUCAGGAGAAUGGGUAAGAAUCAGACUACAUCCAAUGUUUUCUACAANCCCACUACAACCAACAUCGUAGAAUAAAUCUUCAACCUACUCAAUCUGGGUAUAAGCGACNCCGUUGAUUAUAUGGCUACUCAACUUUUGCGGAUGGCACAUACACUAUUAUCGACUAGAGNGAAUGAGUAGUAUACCCCGAUUAAUCCUGUUCAGUAACUGGAGAGAAUCUUCAGCUAGACNUAGGAAGAAGACGACUAAUUUUAGCGCAUGCAAUUGCUACAUAUCCUAUCCAAUCUCUUANCAAGUCUUCGGAAAUCAGUAUUAAGUAUUGAACCGCCGAUGGAACUUUUAAAACAAGAUCNCGGCAACAUAUAUCUCAGUUGUUACACGAAAAAUUUCUCGAAAAAUAUCGAACUUGGAAUNACACGCUCCCUGAUAUGCCAUGCUUAUUCUUGCAUCGUACCGAUAAGGAAGCAAUGCGUANUGCUUCGAGACAGGGAAAAGUAUCGAGAUAAAAAGAAUACAAUAAUGCAUCCUCUAAAAUNUCUCGAACCACGGAAUAUGCAGAGUGGAAUAUACCGAACAUUAUGGAUCUCUUGAGCCGANAUGACAUAGUACUAAAACAAGAUCGNNNNNNNNNNNNNNNNNNNNNNNNNNNNNNNNNNNNNNNNNNNNNNNNGAGGACUAAGCAAGCAACGGAAAUUCCAUGUAAUGGAGUCGGACGAGNGUGCAACAUCUCGUGCUUUAGUCGAUAGACACGUAGGUGUUAUAGGGUACGACUUAAUCUNCUUCUUUCCCAUCUAUAUCACACAGAUCUGCCUCGUAUAAACCUCCAGAAUUAAUCAUGUNCGGCAAAUACUGAAAAUUUCUCAUGGAGUAAUCAUACUUACUCAGAUGAUGGAGAUGUCCNUAAAAGGUGAACUCGAUUGUUUCAAAGAACUGAGACCAAUGUUAGUAAUAGACUUAGAUANUUUCUUUCCAAAAAUUAAAAACACUACUUCUUAGAUUACCAAUCGAUUCCGAAGUAAUCANUCAGAUACGUAGGAAAUGAAGAUGAAAUACGAUGUUUAAUAAGUAUGAUACAUCCUACUCNUUAUUAUUUCUCUUUGUAUUUCUGAUAAUAAAGUAUCUGACAUUGUUCUAUAUGCCACUCNAAUUAGGGUCUAUUGGAACAGGGAAUUAUCAAAGUGUAGAAUUCAGAACGAAAAAUGAAANUCAAUUAUUCAUAUGGAUCUACAGAGCUUGUUUCGCAGUUCUGGAAUGUUGAACCGAAUANUCUCUAGACAUUUCCAAUUGGAUGUGACGGAUACCGUACUCUCAUUGCGUACCAAAUUAUNCCGCUUUAUAUGAGGCGAGACGAACGGAAGUAUCGGAAAUCUACACUCAUUUGUGGGAUANUCAUGCAUACAAAUUUAACUAAUACAAACAUGACCGGGAAAGAUCUGAGAUGCCGAGCCGNUUUGUGAUAAUAUUUUCAAAUAUGACAAUUAAUCUAGAUCUAUUUUAUAUGUUUAUUUAANUAAAACUUAGGAUAAUGCCUUCAUAUAAAGAGAAAUAUACACAUAUUUGUAAUAGGUUAANCGACAAUGGAGAGAAGAGUAUUACUCGAUGAAACCAUAUUGCUUGAGUUACGGCAAACGGNUACUAGAUCUUCAAUUUAAAUUAGAUGCUCCUCCGAAGGAAAUAGACGAAUUUAUGUUGANAUCAAUUACAUUUAGCUCUCACUGAAUUAAUUAGAUAUGCUAGUCCUCAAUUUCAGUUACNCAGCGCAUUUUAAAGGAAAAGAAAUUACUGAAAUCUUGACUACAACAACUGAGAUGAUAUNUUAAUGUAGUAUCUUUGUUAAGAGAUGCGACCGUUAGAGCUCAAACGAUUCUAUCUGAUANUUUCUCCUCAAAAAUCGAAUGAGGACUCAAUUUCGAAGAGAAACACUCUUGAACAACGUUNAUUCUUUAUCAAGGACCGCAAGUUUGACUAACAUUGGAAAUCAGGGUAAUAGACGCAGUUNUCGCAAUGAUACCUAAAGAAUUAUAGUUGAGUCUAGUUCAAAUCUAUUUAACAAAACUUANAUUAUUUAAUGUGCAUAUACUUAUUUCCUUGAUAUUAUAAGAUUAUAUCGCCGUAAGAUUNUUUUAUUAUUUGAAUCGCAACGAAGAAUAUAGACUAGGACGAUAUAUCCAACAAAGCUACNGGGAGUACGAUCGGGACAGAUCGUAUAAAAUACUCAAACCAUAGCCGUGAAGGAGACAGANGUN  >Ss-AA_clean.1_(paired)_contig_927  UCCAUAAAUUUCGGGAACUCGACAUCAUAGAGAUCCGAUAGAGGAUAACUUGCAUCGUUGAUCUUAGUUAUUCCUGACUUCCACAAAUUAUAGAGUUUGGUAUUUCUCUUUGGACCAUCUUUAUCCGUGAAGGCUGGCCAUACCUGGUUCUUUUUAAGAUAGCGAUCUAGAACUAGUCGUUUGAAGUGUGCAUGGUAAUCUCGUAUUGCUAAUAGAUCUAAUUUGCCAGUAGGACAUGCUUCUUUCUUUACUGAGAUGGACGAAGUCUCAGCAUAAACAAAUGGAUGACCUGACAUUUUAGUACACCCGAAUAGUUCUGUUACUAUUGAAAUAUCAUUGGUAGAUUCAAUAAGUUCUAUCAAUUGAGAAGUUAAUGGACACUCAUUUGUUUUUGACAAUUUUUUCUCUUUUUCUUGAAGUUUUGCGCACGUACGCACGAAUGAGCUUAACGGUAGUACAUCGCCUUGAGUUAACGAUGUAAGAUACGACUUAGUUACUGAUUCAGGCCCUUUUACGAGUUCGUACCCUUUGUUUCCGUACUUCUUAAGUACAGCCUCUUGCCAUAACAAUAAUUUUUUCAAGAAUUCACGAAGCUGUUUAUUGGAAUUAUGCAUAUUUGCAUCUAGCGCAAGAAACGCGUUAAAACGAGCCAAACAUACAUCUUGAAUCAUUUGAAGCUGUUCGAAUGAGUAUAAUCUUCGGAUUGGUGCCAACUUCUUCGGCUUUCCAUUUUCAUCGGUAAUCAUCUUACUAAAACCUAUUUGUUCCAUAACAAACCCAUCAUAAAAAAAGAAAGUAGAAUCAUUAAUAGUGAGUCCUUGUUUACCGAAUUGUUUUUUCUGUUUUCGUUGUCGAUAUUUUUCUACUAAGAAAUCCCAGUACCUAUAAGAUUCAUAAUGAAGAUACGUGUCCUCUUUCGCUUCUGGUACGAAUUUCUCUUCAUUUUCUAUGAAUGUCUGAAUUUCUGGUAAGGUUCCCCAUUUUUUGAAAGCACAGAGCUCCGAGCGAUAUAGCUCUGAUGAUUCUGCUAAUGAAGUUUUCAAACUUUCUCGUGUUAUAAGUGUGCGUGGGAAUAAUUCGGGGUAUUGAUCCGGGGUUACUAUUUGAGGAGCCGUAGAGGUCCCUACUAAAUCCAAUAUCCAAGGAGUUGCUAUGCGUUCGAGGCCAGAAUAGGAAUGCAGCAUAUUAUGUUUGCAGAUAUUUCUUAUUAUUACUGGAACAUCUGAUUUGAUAUAAGGAAAAUUACUCUUCGCAAAACGAUUGUACUCCUUGUAAUGCAAUAUCUCAUCAUAAAGUAAUCUUAAUCUAUCCAUAAGAGAAGAGAGAAUAGGACUACGAAGGUGCUUUUCAGCUGGAGCAGCAGAACGACGCUCUCGAACUGAGUCAAACUCAAAUGCCUCACUAAAAUUCAUAGUGCUGAUGUAAGUGAAAUGAUCUCCUAAGUUUUAUUAAAUAACAUAUGUGCAGUAGGAUUCUAGUAAAUAUUAUACAUCCGUAUUUAAGUUGUCUGCAACUCUACUUUCUAACCUGAAACUAUUUCAAUUUCGUCGAAUUCUUCAUCUUCUGUAACCAAGGAAAACUCACUGGACGAUUCAAUCAAGUUUGUUUCCCACACUCUCUUAGUGAGAUUUUCCAAUAAUACUAACGCGCAUACGAAAGCAUAUAGGUCGAACUGCGGUUGACUAUCGAGUAUGUCCAUUUUCGUCACGUGAUUCUAAUUCUUAUGUUAAUAUGGAUGUCUCCUAAGUUUUAUUAAAUAAUCACCAAAGAAGAUUUCAAACUAGUAGUUUCCGGUUCCCUUCCCUACAUCUGAAUUAACCGUGACAAAUAUAAAGAUUAUUGAUGUAAUAGUUGAGAGUAUUCGGAGAUGCCGUGUUUGCCGAAGCGAUCGCAGUGCAUGUAAUAUCAUAACGAGUAUGAUUGAUUCGAUAUUCUCUUCCAUCCGUUGGAGCUGUACAAAUAUCCCGACGAAUGAACUCGGCUUGAUCACAUAUAAUAGAUUCAGAUAGGUAACGAUGGUUAGUAUUCAAUCUAUCUUCCACUCUGAACUUUUCCCACUGAAAACUCGGGCGAUCGAUAGAGAUUGCAUCGAUCAUGCCUUGACUAAUUGUACGAGAAAAGAAGUCGCGAUUCGGAAUUAUAUCAGAAGGUGGGGACUUUCGAAUUCUGAAUAUAAUGACAUCUGGUUCAGUCAAAUUAGUGCUACCGGAUCCGAGUGAUCCAGAAUCUGACAUAUGGAGUCCGUACUUAUUGUUCAAGAAGACAUAAAGACCUGGCAUUUCUUCCUUACUCUUAUCUAAAGCUAAUGUUACCGAAAAAGGCGAACUCUUAAUCGAUAAUAAGUAAUGCCCUUUAGCUGGACACAUGCAGACACGCAAUUUCGUUUCCAUGAGAAGUUUAGCUAAAAUCUCCCAAGAAAGCUCGGUCUUAUCUUGCAGGUUAAGGAUUAAGGCAUUUUGAAAAAAUCCAAUUUUCUUAUCCAUUGUGCGAUGAAGAGAAGUUGUGGCAUUUAUUUGCUCCUGUAGACUCUCUAAUUCACCAGAACCAAUAACUUGCAACUCAACUGAGCUUGAAAAUUUGCAGAGGACUGAAAUAUCUUGACAAUUCGUUAGGAGGGCCGACAUAUUGGUGAAAACAAUUUAUGUGUAACUCCUAAGUUUUAUUAAAUAAUAUGAAAAAUCAUUCGUUGAAAAAAGACAAAUCAGAUCAGAGUUGAGAUUAUUGGGCAGGAGCUUCGGGGACAGCUUCUUCGGCAACUUCUCGAAUAUCGAGAAGAGCGAUGAGUUUCUUUCUAUGCUGAACACUGAGAGUUCCUCCUGCAUAUUGACCGAGAGUAGACUCUUGUUGAGCCAAGGCAAAUACAGCAACGCCAAGAAGCUCAUUGAUAUCAUUCCUGUCAAUAGGAAUAUAUGCGCUCCCGAAUAUAACCUUGAGAUACAUUCUACGUUUGGUAGAAGUAUCGAGAAAUCGUUGGAGGGCAUGAGCAGCAGCAAAAUAUUCCGUUUCAAGAUCAGGAAACUCUUUGAAGAAUUGAGGAUAUUUUCUGGCAUACUUAGUAAUCAAUAGGAGAGGACUAAGUGAUGCACCGAAGGUGAGUCGAAACAUCACAUAGAACAUUCGUUGAGUUCCAGACACCAUUGGAUCAUCUUUAUCAACAAUAGAACUCAUUACAAGAGCGCGAUCGCGAAUGAUUGAAUUGAAUGUCUUGUUGACUUUGCUCAGAACUGCAUGUGAUAAAUACGGGGAAUUAUCAGUAAAAACCUUAAGCUCACCUGAAGGCCUGAAUUGAGAGAUUACAUUUAGUAGUUUAAGAUUGAAUGCAUCAAGGUUCUCAGGAGUCGG  >Ss-AA_clean.1_(paired)_contig_1525  AUGUAGCUGCAACAUCAAAAGACAAAUCAUCUGAUUCUCCUUUACUGAAAUAUCGUGUCCAUGGCAUCAUAGGCAGUCCACCUAACGAUCCUGGAAUUAAAAGCACUUUCCGAGAAGUUGCUGAUCUUAAUAACCGCAAGAUACCUUUAUGUUCAAGACUAUAAAGCGGUGAAGCUAAUCUCCGACGUAAUAAUAAAAUUACUUGAAUAUGCUUCCACCAUACGGCACGGAAAGAAUUAUGAAGUGUUCCUGCUACUGCGACAGAAUUUGCAACCACUCCGGNNNNNNNNNNNNNNNNNNNNNNNNNNNNNNNCAAUACAUUCUUCCGGCUUUACCUCAUGAUUCAAUCGCUCACACCUUCGUUCUACGGAGUAGAGAAAAGAUUGUAAGGCGAGGCUAAGCGACUGUUUUUUAGUGUUAAAUGACAAGAAGAAGACCUGAUUGUCACCUUGUCCUGCCAUCUGGAAAGAACAUUGUUCAUCUUGAAUGGCAUAGUACAUCAUAGCCAAUGUACAAAUCGUCCAAAGUGUUUGUUGAAUUCCUUCAAAACCUCCUAGAUGAUUCCUCCAUACUAAAUUUGAUUCCGGCCAAGUAGAAGCAUGUGAAUUUGGUGUGACACC  >Ss-AA_clean.1_(paired)_contig_1881  GAUCGUACUCCCGUAGCUUUGUUGGAUAUAUCGUCCUAGUCUAUAUUCUUCGUUGCGAUUCAAACAAUCAAAAAUCUUACGGCGAUAUAAUUUUAUAAUAUCAAGGAAAUAAGUAUAUGCACAUUAACUAAUUAAGUUUUGUUAAAUAGAUUUGAACUGGACUCAACUAUAAUUCUUUGGGUAUCAUUGCGAAACUGCGUCUAUUACCCUGUUUUUCAACGUCAGUCAAACUUGCGGUCCUUGAUAAAGAAUAACGUCGUUCAAGAGUGUUUCUCUUCGAAAUUGAGUCCUCAUUAGAUUGUUGGGGAGAAAUAUCAGAUAGAAUC  >Ss-AA_clean.1_(paired)_contig_1075  CUCCCGUAGCUUUGUUGAAUAUCUUGUCCUAUUCUACAUUCUUUAUUGCGAUUAAAAUAAAAUAAUUAAAAAUCUUAUGGCUAUAUAACUUUAGGAUAUUAAGGAAAUAAGUAUAUGCACGUAGAAGAAUUAAGUUUUGUUAAAUAGAUUUAAACUAGACUCGAUUACAAGUCUUUAGGUGUCAUUGCGAAACUGCGUCUAUUACCUUGGUUUUCAAUGUUAGUCGAGCUGGCGGUCCUUGAUAAAGAAUAACGUCGUUCAAGGGUAUUUCUCUUCGAAAGUGAGUCCUCACUAGAUUUUCGAGGGGAAAUAUCAGAUAAAAUUGUUUGGGCUCUAACUGUCGCAUCCCUUAACAAAGAUACUACAUUAAAUAUCAUCUCAGUUGUCGUAGUCAAGAUUUCAUUAAUUUCUUUUCCUUUGAAAUGCGCUGGUAAUUGAAAUUNNUAAUUAAUUCAGUAAGAGCUAAAUGUAGUUGAUUCAACAUAAAUUCAUCGAUUUCUUUGGGAGGAGCAUUCAAUUUAACUUGAAGGUCGAGCACCGUUUGUCGUAAUUCAAGCAAUAUGGUUUCAUCGAGUAAUACUCUUCUCUCCAUUGUCGUUAACCUAUUACAAAUAUGCGUGUACUUCUCUUUAUACGAAGGCAUUAUCCUAAGUUUUAUUAAAUAAACACAUAAAAUCGAUCUAGAUUAAUUAUCAUAUGAGAAAAUAUUAUCACAAAUGGCUCGGCAUCUCAAAUCUUUUCCAGUCAUGUUUGUAUUAGUUAAAUUUGUGUGCAUGAUAUCCCAUAAAUGAAUGUAAAUUUCAGAUACUUCCGUUCGUCUUGCCUCAUAUAAAGCGGAUAAUUUCUCGCGCAAUGAGAGUACAGUAUCCGUAACAUCUAAUUGAAAAUGUCUAGAAAUAUUGGGUUCCACAUUCCAAAAUUGCGAAACAAGCUCUGUAGAUCCAUAAGAAUAAUUGAUUUCAUUUUUCGUUUUGAAUUUUACACUUUGAUAAUUCCCUGCUCCGAUAGACCCCAAUUGAGUCGCAUACAGAACAAUGUCAGAUACCUUAUUAUCAGAAA  >Ss-AA_clean.1_(paired)_contig_405  AAGAUUAUCCCAGGAAUUAAUUUCAUUGAUAGAAUAUCUCAAGCAAAUUCUACCAUUCUUUCAGUCCUCCUAGAUCGAUGGGUAUUAGCUGCUAAGAAGCCUGGAGGGCGUCAUCCACCUCCUUUCGAGAUGGCAACUGAGUUAAGGACUCAUUGGAAUCUUGGAUUAGACAAUACAUCAAUCGGAGUUUAUACUCCUUUUGAUUUUGAAAUUGGUUAUUUUAAACCUGGUGUACCACUAAUUAGUGCAACGACUGAAAAUAGGAAAGAUAUUUUAACUACUUGUGGUCGAGCGCCUCCUAAUUUUGGGACUUCGACUAAACAAAAAUUAUCCGAUCAUGGAUAUCGCAUCGUAAGUACGAAUUCAACGAUGAAAGAUUUAAAGAAUGCAGUACUUACAUAUUCCGAACUACAAGGAGACCCUUCAUUAAAACCACUAUUUCAAUCAAUUAUUUCGUCUCGUUCUCCUUGGACCUUAGAAAAAUUAAUACCAAUUUUCCCUGAAUCGAUUGGUGGAACUGCAGUUCAUCGUCACAAGAGUACAUCUCAUGUAUUCUCUGUUUUAGGUAGUUCUUCGGUGCCGACACAUAUUAUCUUUAGCUCUGAUCAAGCAGGUAUUUUAUCUGGUGGAGAUGCAGAUUAUCCGGUUGUGUUUCAAACCCUUUACAUGACAUUAACGAAUUUGUAUCAAAUACUGUCUACCUGUAAUACUAUGCUUCCUCCAAAUAUCUGUUAUUAUAUCCCGAGUCAAUUACGUGAAAUUAAUACUUCUGUGUCUAAGAUUCCUUCGGAUCAAGCUAUAGACAUUAAAUGGCCGGAUCUCACGUCUAAUAAAUUAGCAUGGGUAGGAGAGAUAUUUGCGUCCGAAAUUCCUGAAAUUCCGCUCCCUUCAGUAAUACCACACAUAUCAUCGCCAACAUCCGAUUUAGACUUGAUUUAUUCUUAUAUAGAAUCAUGCGUUUCUUCUAGUGCUGACCAAAAGAAACCUUAUGAUGGAAUACUCGGAACUGCAGAUAUUUUUGAUUUCAAAGAGAUUUCGCGAGUAGAUCCUAAUGUCGUUGAACAAGCGAUCUGUUGGAAUAUUAUAACUGACGCAUAUUAUUCGAUAUUUGUUUCAAAUUCUCCGGACAAAUCUCUACCAUUAUUCAAGAAAAUUCUAAAGACAAAAGCUACUUAUUACUCAGGAGAAUGGGUUAGAAUUAGACUACAUCCGAUGUUUUCUACCACUCAAUACAAUCAACAUCGUAGAAUAAAUCUUCAACCUACUCAAUCUGGAUAUAAGCGGCCCGUUGACUAUAUGGCCACUCAACUAUUACGCAUGGCACACACGUUGUUAUCAACUAGAGGAAUGAGUAGUAUACCUCGACUAAUACUAUUCAGUAACUGGAGAGAAUCAUCCGCUAGAUUAGGAAGAAGACGAUUAAUUUUAGCGCAUGCAAUUGCUACAUAUCCUAUUCAAUCGCUUACAAGUCUCCGAAAAUCGGUAUUAAGUAUUGAACCUCCAACGGAACUUUUAAAACAAGAUCCAGCGACAUAUAUCUCAGUUGUGACACGAAAAAUCUCUCGGAAGAUAUCGAACUUAGAGUACACGCUCCCUGAUAUGCCGUGCUUGUUCUUGCAUCGUACCGAUAAAGAAGCAAUGCGUAUGCUUCGGGACAGAGGAAAAUUUCAAGACAAAAAGAAUACAAUAACGCAUCCUNNNNNNNNNNNNNNAACAGGAACGUAGUAUUAGUGAUCGAUUACGAAUACUUCGUCGUAGAACAAUAGGAAUCUAUUCCCCUCUUUACAGUGAUUGGAAUGCAAUAUUCGAAAUAUUAUUCAAUCGAGGAUUGGACAAACAACGGAAGUUCCAUGUAAUUGGAGUCGGAAGAGGUGCUACAUCCCGUGCUUUAGUCGAUAGACAUGUAGGUGUUAUUGGGUACGACUUAAUAUCCUCUUUUCCAUCUAUAUCACAUAGAUCGGCUUCGUACAAACCACCGGAAUUAAUUAUGUCGGCGAAUACUGAAAAUUUCUCGUGGAGUAAUCAUACAUACUCAGACGAUGGAGACGUUCUAAAGGGGAAACUGGAUUGUUUUGAUGAAAAUAGACCAAUGUUAGUAAUCGACUUAGACAUUUCGUUCCAAAAAUUAAAAACACUGCUUCUUAGAUUACCAAUCGAUUCCGAGGUUAUCGUGAGAUAUACGGGAAAUGAAGAUGAAAUACGAUGCUUAAUAAGUAUGAUACACCCCACUACUAUUAUCUCCCUCUGUAUUUCUGACAAUAAAGUAUCAGAUAUUGUUCUAUAUGCAACUCAAUUAGGAUCUAUUGGAGUAGGCAAUUAUCAGAGUGUAGAAUUCAAAACUAAAAAUGAAAUCAAUUAUUCAUAUGGAUCCACCGAACUUGUUUCGCAAUUCUGGAAUGUGGAACCUAAUAUUUCUAGACAUUUUCAAUUAGAUGUGACGGAUACCGUGCUUUCAUUACGUGAGAAAUUAUCUGCCUUAUACGAGGCACGACGAACAGAAGUAUCUGAAAUCUACACUCAUUUGUGGGAUAUUAUGCACACAAAUUUAAACGAUACAGGCAUGACUGGGAAAGAUCUUAGAUGCCGAGCCAUCUGUGAUAAUAUUUUUGCAUACGAUACUUAACAUCUCGAUCUUAUGUGUUUAUUUAAUAAAACNNCCUUCGUAUAAAGAGAAGUAUAUGCAUAUUUCCAAUCGGUUAACGACAAUGGAAAGAAGAGUUUUACUUGACGAAACCAUAUUACUUGAAUUACGACAAACAGUUCUAGAUCUUCAUGCUAAAUUGAAUGCUCCUCCUAAAGAAAUAGAUGAAUUUAUGCUGAAUCAAUUACAUUUAGCCCUUACAGAAUUAAUUAAAUAUGCUAGCCCUCAAUUUCAACUGCCAUCGCAUUUUAAAGGAAAAGAAAUUAACGAAAUCCUGAUCACAACAACUGAAAUGAUAUUUAAUGUAGCAUCUUUACUAAGAGAUGCGACAGUCAGAGCUCAAACAAUUUUGUCAGAUAUUUCUCCCAAACAAUCUGUUGAAGACUCACCUUCAAGAAAAAACAAUAUUGAUCGGCGGUACUCUCUGUCACGGUCCACGAGCUCCACAAAAAUUGAAAAUCAGGGUAAUAGACGCAGCUUCGCAAUGACACCUCAAGAACCAUAGUCCAGUCUGGAUUCGAUUUAUUUAAAAAAACUUAAAUAUUCGAUGUAUAUAUUUAUUUUCAUCAUAUAUUAAAAAUUGUAUCAUACACUUAAUUUUAAAUUUAAUCAAGUUGUAAGUAUGAUUCGGAAAAAGAUAAAUAUGUUCAACAAAGUUACGGGAGUACGAUCGGGACAGAUCGUAUAAUAUACUCAAAC  >Ss-AA_clean.1_(paired)_contig_512  ACAAGUGAUUAGCGAAGAAGCUAUAGCUUCAUUGGACAACAAUGUGAGUGGAAGAUCCUUUUCGAUGGGAGUGUCCAAGAUGAUGUCAUCUCUUUCGAGAGCCUCUUGGGCAGAUGCAGUAGAAGAAGAGCUUCCCAAGACGCCUGAAUCUUCAGCAAAACUAGCUGUAAGCGGAAGUGCAAAGCUCACGGGUGCAUCACCUGCGAGAAUUGACUCUCAAAGUAGAUCGGACGCUCCCGAGAGUGAUUCUGAGGAUGAUUUCCAAGAAUCUCCUGUCAUCAAAAGAGAGGGGUCUCAGUUAGCUGCAGAAGAGUCUGAAGCAGAGUUCAACUUGAGCCCGGUUGUCAAGAGUAGCGUCUCGAAGCAGGCCGUAGGCAUCUUGGGCGUCAGGGGUAACCCGCGUGAGAUAUUGAACCCUACCGCUCAUAAGAUUACCGACGAACAGCUCGCAGGGACAACAAUGAAAAUAACACUCGACAAGCUCCAGCAGGUCGUAGAGGCUCAGGAUAACGUUAUCAAAAUCCUCUUGCAACGCUUGGAUGCCAGUGAUACUAAAAUGGCCAGCCUGCUUGCGUCUAUUAACUCGUUGAAAGUUGUGGCUGAGGAUGUCAGCUCCCGAGUUAAAUCUAUUGAUUCUGAUGCUCUUGACGUUUUGAACCAAGCUGCGAAGAUCGUGCGAGAUAAUCGAGUUCCCGAUGCACCUAUUGCCAAGAUAGAGCAGGAGCUCGAGAAGACUCAGGAUUCGGUUGCGAGGACUGCCGAGCGAGCUCCCAUCCAGUCUCCUAAGAUCAAAGCAUCCAAGGGAACAAGAAGAUUGAAAUUAUCAUUUGAAUAAACUAUUGAAUUGAGUGACAUCGAUUAUCUGGAAGUUGGACAUCUUAACAUCGGGAGAUUUAAGAAAAACCAAGCAAGAACAGACAACAACUUUAGUCUCGUAUCAAUAAUUCUUUAUCAUAAUCUCUUUCGCAUUUCGAACAAUCUUCUAUCAUGGCCGUCCACUCCCGAGCACAAGUCCUAGGACUCGCUUCUGCAUCUCUCUUCCGAUCUUCUAACAGGGCAGUCGAAGUUGCUUCUCUGUCUGAGACGAUCAACUAUAUCAAGAUCAGCUUUGGUGAAGUUGGUCCAAAGGACGAAGCAAAAUUGAGGGCUUUUGUCGCUGCGCUUCUAAUUGAAGCUUAUCAAUCCACAGAGAUGAAAUCUGCCCCUGACAUGAUAUCUUACGUUCUAACUGGAACAGAGCUCAUUUUCCCUGGAACAGUCGAUCGGCUUCAAAGGGAUCAAGCUGGUCAGUUCAACCGUAUCUCCGUCCAAGUCGACGGCCCGGGCAUUCUUGAUAGAAUGACCAAUGAACCAGGAGCUGCUGAGACAACUACCUCUGCAGAGAACGUCAUCGACUUGAUGGUUGUCGAUUUGGAAGAGGAUGCCACUCUCUGGGGAGUUGGUAUAGUCGAGGUUGCUGCUCGGUGGGGAAUCGAGAUGUUUCCAAUUGGCAAAAAUGUUUCGCUUGAGAACAUUACUGCGUUCCGCCACAAUCGUGUACUGGCUAUCAAGAAUGCUUUGAUUCUCCCUGAUGAUUAUCCUAUCUUUCGAGAGAGUCUCUUGCCCGUCUUUGCAACCUUGAAGGGAAUUGAAAGAUCGUUCAACGCAUACAAGUCGAUCAGAAAGCACCUCGCUACGAGGUGGGCAAUCUCUGUCAACCAAGGAAGCACAACUCGCGAGCUUGACGCCUUCCUCAUGACGUUCAGGCUCACCGAUGGGUAUGGACUUGGUGCUCCUAUGUUCAUCGCUGAGCUUCUCUCAGCAUAUCCUGAACUACAAGGAUUCGCAGAACUCUCCCCUGCCAUUAAUAGCUUCAUUGACGCAAUGGGAGCCUUCAUGGAGGAAGACGAGAAUCUGAGAGGAUUUGUCAAAGUGAGAUACGGAUCCCAACAUCGACUUUUCUCUUCUACUACGAGAGGAGCACUGCUUGCUUUAGCUGUCUGCUUCAAAAAGCAGACUGAAGCUACAGCUGGAAACUUCAUCGACACCAGCCCUUUCUCAGAUGUACUAGCUAGGGCCAAUGCUUACCUCCAAUCCAAAGGCAGACCUGUCAUCUCUGGAAUUGGAGGUCGACAAGAAGCUGCCCUUCAAUAGACAUCGCCCUCAUUCAUCAUGAAUCUGAUUUUCAAUUGUCAUACCUUUACGUUCAUUAUCGAUCCUUGCCUCUAUUUGAGAUUUAAGAAAAACCGUGCAAGAACAGAGUCAAUCAAGAGCUGAACUUUAUGUCUAGACUACGUCUGCCCCAGUCCUUCUCAGGACCUGUAUGGACAUUUUCCAUCUUAGGUGUGAUAGUGUCGAAGCGCCCAGAAAUCCUGUUGCAGACCCGGUUAGCUGCCCUGCGAACACUCAUUCUUGUUUCUAGGAGCACAUCGCUCAACACUCGCGUGUCAUUGCUUCGGCUUCCUAUAGAAAUCAUAGCUCCGUUCUCUCCAGAGAUGACGGCUGUCCAUGAGUUCUGCGAGCAAUUGCCCGAUCAUAUUAGUGUUUCAGACUUGAAUCGCGUUGUUGACAUUGGACUAGCGGGAUUGACAGUGGCUCCAUCAACACUCGCCACGUACAUUGCUGUGGCAUCAGCGAGUGAACACCUCUCUCGCGAGGCAAGAGUCCUUACUGCAAGUUUAGGAAUUGGAGAGCUCCCGCGGUCUAGAAGGAGAGAGAUUCGUUUUGAGGUUGUGAGAAUUCUCAAUGAAUCUGACGUCGCCUUGAGAGACAAAGUGAAGCUAAUCCAGCAGGAUAUCCUCUCGUCCUCGAUCCCGUCUGCGAUUGAGUAGAUUUAAGAAAAACCAGGCAAGAUCAGAAUUAAACUAUCGCCAUGGAGUUCGAUGAUGAUUUUGACACUGUAUACAUGAACAGGGUUAAGACCUUUGGCGACAUUCUCGAUAGUCCAAUAUUGACCACAAUAAAACGAGAUAUAUUCAACAGCUUGUCCUUAGCUAGAAGUCUUUCGAAGCCCAUAUCUAAAACUUACUCUCAGGAUGAAUCUGCAAGCGGACGAUUAAUCGAGUUGGCACUAAUCAAAUCACUAUGCCAGAGACAAGGUGUCCAUCGAUUAGGUCACCUCGAUAGAUCUUGCGUGUCUCUGGCUUGUCGCAUUGUAAAAGAGCAGGCCCCUAUUAUCGACUUAGAUGUUGCUACAAGAUUCCAGAUCCUUAACGCCUUGACCGAGUCCUCAAACCCGUCUUUUUCCGAUCCAAAGAGUGCAGUUUUUGAAGCGGGAAGGCUCUAUGACACAGUGCGAGAUGGGUUAGGAAUUCAAUUGAAUUCUAAUGUGACUCCGCUCCUGAACCGGAAGAACCCGCGCUCAGAGCCACUUGAAAUAUACUCAAGGUACUCGAGAGCUGAUCAACUUCUCGAGAUGAUCAUUCACAACCGUCAAGAGCAAGGACUUCAAAACAAACAAGGAUCAAUAACUUGCAUCGUUCAUCAUAAUUGGCUGUCUAGGAUUUGGAUUGAAGGAGAUGUUGUUCUAUUUAAAGUAGAGGGAGCUUUGUACUCAACGUCAUGGGAUGAUUUCCUGUGCAUUAGGUCGAUGGCUAUUUACCGGAGAAACUCCUUCCUACUUAUAGCUAUCGAUCACUCGAUACCUGCCACCACUACCUCCAGCCUUUUAAAGCUCCUUCAGUGGCAAGAGCAUUGUAUCUUCUCUUACGGAAAUCCAGGAUACGAGCUGGCCAAGGCUGUAGAGUCAGUUUUCAAAGCAAGACUAAUGCAGCUUGGAGACGGGCAGUGUGUAGGUGAUGCCUUCGAGCUUAUGAUCGUUAAACAGAUGGAAAAAGAAGCUAAGUUGACUCGAGAUAAGACAUCUCCUCUUGUUGAAAGACUGCAAAAUCUAGCGUUGUCUGUCAAAUCACCAAGAGAAGCUGCUGAGUUGUUUGGUUGUCUCAAAUUCUCAGGACAUCCUCUGAUAGAUCCGGAAAUUGCUUCGCAGUCUGCUCGGUCUCAUGGCACAGCAAAGGGAAAUCCUGGAUUCGGAGAGACAAUGCGAAUGAGGGCAGAAUUCUGUGACAUGCUACUCAAAGGCUACAUCAAGAAGCACGCCCGCUGGCCACCAUUAAUCCACUCGGCAGGCCGGAAAACGAAACUUCAAAAACUUCAUGAAAAGAGAGUAUUAGUAUUUGGACCGCUUGACUAUGAUUAUCAAGAUUGGUACUAUGCAGCCUGGCCUAAACUUCUUGAUUUCGACUACAACAUCGAUUAUCUAGACAUGAUGGAUGAUAAGUCAACCGGCCUCGAUCCUCAAGACGCGUGGAAAGCUUGGGAUAGUUGCAAAAAAGAUGCUCCCUCUUUGGCGCAUAUCCCAAAUGAGAGGUCAAAGAAGUUGAUAAUUAGGAUCUUGAGCAUGAAAGAGUUUGAUCCUAAGGCCAUCUGUGAGCAAAUACGGGUAUUGAACCUGUCAUUAGCAGAUAUUUCUAUGUCUCUCUACCCAAAAGAGCGAGAGUUCAAGCUGGAAGCUCGAUUGUUUGUAAUGCUAGAAUUCACAGUGAGGGUUUUCCUUACACUGGCUGAGAAGAACUUUAAGCGUCUUCUGAAAGAUUAUCUACCCGACCAAUCGAUGACGAAAGGUCGAAAGGGCACGAUGCAACAUCUAGAGGGAAUGACUGCACGGCAAUCAAAUCCUGAUAUCGACACCGUUUUUAUAGAAGUCGAUCUUUCUCGAUGGAAUCUCUUAUGGCGAGGAAUUGUCGUAGAUCCUGUUAGUAAUAUCGCAGACUCCAUAUUUGGACUUCCAGGAGCAUUCUCUAAGGGUCACGAGAUAUUCGAGAACUCGACAGUUGUUGUACGAGUAUCCACUGAAACACCAGAUGGAGUGGUUCCCGGGAGUUUUCCUCGAGAGUGGCCAGAGAGCAAGUACGUGUGGAGAAAUCAUCUAGGAGGGUUUGAAGGAAUAAUGCAGGCGCAAUGGACUGCUUGCACUCAGGCUGAGAUCAAAGCCGUUAUGAGAGACUUGGACGUAGUGUCUUACAAACUGCUCGGUCAGGGAGACAAUCAGAUCUUAGAAGUGUCUUACAAUCGUGACCACGACAAAGAUAAAAUGGUUCAAGCGCUGGAAGUGUCAGCUCGGUGCACAGAAGAGUUGAGUAGGCGGUUUUCUAGGCUCAAUCAAGUAAUCAAGCCAGAUGAGUGCUUAGCAUCUCGAUCUACCGUCACUUAUAGUAAGAUCUGUUGGCAAGAUGGAGUGUUGAUACCCACAACUCUGAAACACGCAGCUACAGUGGCUCCGGUUGGUACUUCCAACAUUCCUGGUCUCGUUGUCGGUCUCUCGGCAAUAAGCUCUGGAUGCAGGGCAUCAGCCGAUGCUUUUAUCGAUCCGUCGCUUGGGUAUUUGUACUUCUUGAUACUGUUUAGAGAAUAUCUCCCUAGAGCAUCUCGAACACUCCCCAGCAAACAGCUUCUCAAUUAUGCCUGGAGUCAAGAUCAACUAGAUAGUGCCAGUACUAUCCCCGGUGACUUAGGAGGACUCCCAAUACAGAUACCAACGGAUUUUUGCUUUGGAGGCACUUCAGACAGGUUGAGUUCCUCAGUUGCUGCUCUUGUCUGUUUAUCUCAUGUGAACAGAAACUCACAGCAAUACCUCGGCUACUUGGAGACAUCGUUACCCUGGAAACCUGACCCUGAUCCUGCCACCCUCCUUGAAGAUCCUUUCUCUGUCCCUAUACUUCCUAGCGUUGGAGCUGAUGUUCAAGUAGAUCAAGCUAUUAAGACUGUAGUUCCUUCAAUUACCCAGAACAUCGAUCUCAAACAGAUCAUGUCCACUUCGGUUGAUGAGUUUGGAAGAUCUUUGUCUUCCUUCUUAACACGACUUAGACCUUUUUACCCACUACUAAUGGCAGAUCUUGUUGAGUUAUCAGUGAUCGGAGUCAAGAAGAAAGUCUUCAAGAAAUUCACUGGGACCCGGACCAUACAGCAACUUGUCCGAAACAAUGCUCCGAUCAACUAUGGACAUGCAGUUAUUUACGCAGAUUAUAAAAGAGUCACAAGAUUAAGGACGUUCAUGUCCCAAUCCUCUCAAGCAGGCCUUACCAGUACCUUCCCUUCUCGUAGUAUUUUCUCCAGAGUAGUGAAGUAUCGAGAGAGGUGGUUUCCGGACGGAUCUAACAAGUUAGAAGGAGUGACAGUCCUUCAUCCUCUAGAAGCUGUUGCAGAUUCGGGACUCAAAAUACAAAGUCCAGAUUACCUGGAGUUCACUAGUCAGGUUCCUUGGAACACUAUGAUGAGUACGAAAGGACCUCAUCCAGGACGGUGGGGGGACAAAACUUGGGAGCACCGUAGAGUAACUGGCGUCGAGGUUAUCGGAACACAAAAAGCUGCACUGGCGGCAAAAAGGUUGCUCAUGAUGGAGUCCCAAUUAACAGCUGGCGGAGAGCUCAAGAAAGCAAUACGGGGUGUCCUCCGACAACGAACUACGGUGGAUGAGCACGAUCUAGAGAUAUUCAUGCCGACUGUUAUAGGGGGAGUAGCUGCUCACCGAUGGGAUAGCACUGUUGAAGAAAAGGCCUUUGCUUGGCUCGGCCCAAUCUCUUUGACACAACAUUCUACUGUUCAAACGGAUUCUAUGGGCAGCUUAAGUGGGGGAGUCAAGGACUACUCGUUUUGUUUUCAGGAGCAUUCGUUCUUCGGUCUUCAGUGUAUGCGAGCAAGCCCAGAGGCUUUCGAUGGAAACCCUACUCUGCGUCUCCAUUAUGACAUCUCUCCUGAGAUGCUAAUAACGAGUGUUCCGGUAACUGCAGAGGGAAAAGCUCCUGAGCUCUCUUUGGCUCGAGAUCUGCAAAAGAAUCCGCUUGUCUGUGCCAGCAACAUUUUAUAUCAAGUGCUUAGUGACGAGAUGCCAGCGACUAUAGCCCCUAUAUGGCAAACUCCACCGUCAUGCUCCACAGAAGCAGGGAUCCGGUUAAUGAUUCAUCACUUCUUGGAUCAACUUGAAAACCCUGUCUUGUCAGAAGCUGCAGUAGAUGCCAAGGAUGCUCCGUCAGGAUUGUCACUCGAUGUGGGGAGCUUGAUUGGCGUGGGACUUCAGCCUAUGAUGAUGGCUGCUGGAAGGGCUGUGUUCUUACGGUCUGUAGAGAUGUCGCUCUCAGAGACGGGCUUGAUGGACAGAGUUAUACUCUCAGCUUACAUUGAUGCUCUUUCGGGAGUCGCUGCUCUUCCGUUGGCUCGAUUUGCAAACAAUCCUCAAGUCCGCCAGCAAGCAUGGGUUCAGGCAUCAGGAGUACUUAUAGCUCCUGGGAGACAUGGCAGUUCCGUGCUUAAAUCACGAUUGUCCUCUUAUAUCAGAGAUGAAGCUCAUAGGUGUUACAGAGACAUUCGCAAUCUUAGCAAUUCUCGAAUGAUCUUGUCUUCAUAUGCAGAGCGAGCCACUCCGUCUAGGGUACUUGGAUGCUAUGUGGCAUGCUCAAUAGUACUGCUGGGCCUCCGAGGCUCUAUUGACGAUUGUCGGAAGAUCUACCGGAAACACUUGAAUGGCCUCAGGAACCUGGUUAGAGAAACGGAUAGGAUUACGCAUCAUGUCACAAUUCUGAACCUCCUAUCUAAUCCAGAGUUUGAAGAGUCAUCAGUCUUAGCAACAGAGAUAUUGGGAGGACGAGUCCUGACGCGAACUGCGAUGAGCUUCGAAGAUAGUAAGCGAUACUUACGAACGGGGCCUAGAUCGAUCGCAUCUCAGAGACUCAUUACCUCUCCCUCUCUACCCGCUACUAUCACUUGGCAGAUGGGAGUGGCUGGUGAGUUCCCAUUAUCUAGCCCACUGUCGAAACCCCUCUUAACUCUUGAUAGACGGACUGACAGUUUGCGCUUGGUAAGCAGGAAUCUUGGAUGGAGCGUUGGAGGGUCCUCCAUUUCUCGGCUGAGCUACCAGGUCCUCUCGGUAAUCAGCAGUGCUCUGAGACAAGGCCCUGUCUUAUGUGUUGGAGUCGGGAAUGGGGCAAUGGCAAGAGAUACGUUCGCUCUCGGGGCUACUUGCGUUAUUGGAGUAGAUCUUCUUUCAGAUCUACCAUCAGUGAGUUCCUUGGGAACAGGAUAUCUUCCCCCCGAAAUCCCGGUAGUAGAUCCAAUUCUUAACUGGAGAUGGGCAAAAGGAGUGUUUAAUCAUGGUGGCGACUGGUUCGAUGAGAAAGUCCACUCUGAUGUGCUAUCUGAACAGCCCGGUGUUAUCUGCAUUGACAUCCAACCGGGGCGAAGGAUGAUAUGGGAGGACAUUCUACCAAUCUUAAAGAGCAUGAUAAAGACUAUCAUAGUGACAAGACGCGAACUCACACCUCCAGAAGCUGCAUUGUUUUAUCAAGAGUGUAGGGGUACAUUCUCGUCGUUUUGCAUGUUCCAAUCUACUAUAAAUGGAACUGAGUACUGGCUAAUAGCUCGAACGUCCGGAUGUCAAUCAGUAAGCCGACCCACUAGUGCUGUUGAACCAGUCUUCCUUCGAUUAGACCCUCUUGAUUCCCCUCCGUUGGAUUCUCCAUUUUAUCCUAAUCAGUUUGAAUUGAAUAGGACAACUCUAGUGAAAGGAAGAGUGCCUCCAGGAGUUAGUCUUGGGGUGGCUAAGGAUUACUUGUUAAUCUUCGUGGAUUGGAGUAUGUCAAAGAGGAGUCAUAGGCGUGGAAGAGAAGUGACAGAAGAUAUUGUACUAGCUCUAUACUUGCUGAGUAGGCUUAACUCUAUGUUCGAACACGGUCCGCUUACUAAAGAAGAACUAACAGAGCAGUUAGCUGACUUGGCCCAAAGCUCAGCUCCGCCAGAAAUCCAGGGACAUCAAGUGCCAGCGGUCCGGCAAGCGGCGAUAUUUAACAUGGCAUCCAGAGUUUUGCCUCGACUUAUGAGAGAAAGUAGGAACAGAUGGUCUUUACAACUGUGAGCAUUUAAGAAAAAACCAUGCAAGAUCAGAUCAUGUCACCGUCAUACAAAGAGAAAUUGUCCUUAGCUGAAACGAAGCUUAGAAUGCUCAUCGAGGGAGGUGUUCUCGAGAGCUUGGAAAAGGCAAAAAUAGCAUUCGUACAUGCAACAAUCCAAGCCAAGACACAGUCCUUGUUGAAUAGAAUCAGAGAUGUAGCAGAGAUUCACUCUCUUCAUGCACCUCCGAGUACUCCUCAACUACCUUUUGAAGCUGAAUGGAUGACUGUUUCCUCUCAGGCAAUCAAUGCAAUAGAAACAGCCGAAUUAUUAGUAGCCCAAUUGAGUGAUCAACUGAUUUCGGAAAAAGCCAAGACUCGGCCUCCCCGAAUCAGCGGAUCACUUGAUACGAUCCUGAAACAAAUCCAAAUGAUAGAAGAUUCAGCAAACUCACCUCCAUCAAUCGAUUGCCUUGAUGAGUCGCAUUUAUCUGCCAUGGGAGAUACUUUCAAGAAACUGACUGAGCAGACUCAUAAUUAAGAAGUUUGGUUCAUGAUUUAAGAAAAACUGGGCUCGAGGUGUCUGCCUAUUCGGAGUUCGUUGAUCAAACUAGCAACCAGGACCAGCAUAGACAGCCAGCCGGGUCGCAUUGAAGUCUGGUUAAUCUAGAUUCUGCUCUGCACCCCCUUUCGAAGGUGCACAGAU  >Ss-AA_clean.1_(paired)_contig_513  CUGUCAAUCGACCUAAUCCUUUUUCAAAUCUUCCCUAUCGUGGACUCAAUGGCAAGCAAGCAGAACCCGCAAGUGAUUAGUGAAGAGGCCGUUGCUUCGUUGGACAACAAUGUGAGUGGUAGAUCCUUCUCGAUGGGAGUAUCCAAGAUGAUGUCAUCUCUCUCGAGAGCGUCCUGGGCAGAUGCAGUUGAGGAGGAACUUCCCAAGUCUCCAGAUUCUGCAGCGAAGCUGACUGCCUUUGGGAGUGCGAAACUAGUAGGCACCUCACCUGCUAGAGUUGACUCUCAGAGUCGAUCAGAUGCUCCUGAGAGUGAGUCUGAGGAUGACUUCCAAGAGUCCCCUGUCAUUAAAAAGGAAGGAUCACAGUUAGCUGCUGAAGAAUCUGAAGCAGAGUUCAAUUUGAGUCCAGUCGUCAAAGGCAGCGCUCCUAAGCAAGCCGUGGGGAUCCUGGGUGUUAGGAGUAAUCAACGUGAAAUAUUGAACCCUACUGCACAUAAGAUCACCGAUGAACAACUCGCUGGAACAACAAUGAAGAUCACACUUGAUAAGCUCCAGCAAGUUGUGGAGGCUCAAGACAACGUGAUCAAGAUCCUUUUGCAACGCUUAGAUGCCAGCGAUACUAAGAUAGCCAACCUGCUUGCUACUAUCAAUUCUUUGAAGGUCGUGGCCGAGGACGUCAGCUCUCGAGUCAAAUCCAUCGAUACUGAUGCUCUUGAUGUGUUGAACCAAGCUGCUAAAAUUGUGCGAGAUAAUCAAGUUCCCGAUGCUCCGAUUGCUAAAAUAGAGCAGGAACUCGAGAAGACUCAAGACUCGGUUGCAAAGACUGCCGAGCGAGCUCCCAUCCAAUCUCCAAAGAUCAAAGCGUCCAAGGGAGCAAGACGAUUAAAGCUGUCAUUCGAGUGAACUGGUGAUCUGAUGAACAUCGAUUGAGUGAGAGUUGGAAUUUGGGACAUCUUAACAUCAAGAGAUUUAAGAAAAACCGGGCAAGAACAGACAACAACAUUAGUCUCGUAUCAACCACACUCAAUCACAAUUUUCCACGCACUUCUAACUAUCGUCUAUCAUGGCUGUCCACUCUCGAGCACAGGUCCUAGGACUAGCUUCUGCCUCUCUCUUCCGAUCUUCUAACANNAUCAACUAUAUCAAGAUCAGCUUUGGUGAAGUCGGUCCAAAGGAUGAAGCAAAAUUGAGGGCUUUUAUCGCUGCGCUCCUUAUUGAAGCUUAUCAAUCCACGGAGAUGAAGUCUGCUCCUGACAUGAUAUCUUAUGUUUUGACUGGAACAGAGCUUAUCUUUCCUGGAACAGUCGAUCGACUUCAAAGAGAUCAAGCUGGUCAAUUCAAUCGUAUCUCAGUUCAAGUUGAUGGUCCGGGUAUACUCGAUAGAAUGACCAAUGAACCUGGAGCUGCUGAGACAACCACCUCUGCAGAAAACGUCAUCGACUUGAUGGUUGUCGACUUGGAAGAAGAUGCAACUCUCUGGGGAGUUGGAAUAGUCGAGGUUGCUGCGCGGUGGGGAAUCGAGAUGUUUCCCAUUGGCAAGAAUGUCUCGCUUGAAAACAUCACUGCUUUCCGCCAUAAUCGUGUGCUGGCCAUUAAGAAUGCUUUGAUUCUCCCCGAUGAUUAUCCCAUCUUUCGAGAGAGCCUCUUGCCCGUUUUCGCCACCUUGAAGGGAAUUGAAAGAUCGUUCAACGCAUACAAGUCGAUUAGAAAGCACCUUGCUACAAGAUGGGCUAUCUCUGUCAAUCAAGGCAGUACUACCCGUGAGCUGGACGCCUUCCUCAUGACAUUCCGACUCACUGAUGGGUAUGGGUUGGGUGCUCCUAUGUUCAUCGCAGAGCUUCUCUCCGCAUAUCCUGAACUACAGGGAUUUGCAGAGCUCUCUCCUGCUAUCAAUAGCUUCAUCGACGCCAUGGGGGCUUUUAUGGAAGAAGAUGAGAAUCUGAGAGGGUUUGUCAAAGUGAGAUACGGAUCCCAACAUCGACUUUUCUCUUCUACUACAAGAGGAGCACUGCUUGCCUUAGCUGUCUGCUUUAAGAAGCAAACUGAAGCCACAGCUGGAAACUUCAUCGACACCAGCCCCUUCUCGGAUGUGCUUGCAAGGGCCAAUGCUUACCUCCAGUCUAAGGGCAGACCAGUCAUCUCUGGAAUCGGAGGUCGACAAGAAGCUGCUCUUCAAUAGACAUCUCCCUUGCUCACCGUGAAUCUGAUCUUUCAAAUAUGAUGUCCUUUUCCAAUCAUUACUGACCUAUGCCACUACUUGUGAUUUAAGAAAAACCGAGUAAGAACAGAAUCAAUUAAGAGUAGAACCUUAUGUCUCGAUUGCGUCUUCCACAAUCCUUCUCAGGUCCCGUAUGGACAUUUUCUAUCUUGGGCGUGAUAGUGUCGAAGCGUCCAGAAAUCGUGUUACAGACCCGUUUAGCCGCCUUGCGAACGCUUAUCCUGGUUUCUAGGAGCACAUCACUCAACACCCGCGUGUCGAUGCUCAGGCUUCCUAUAGAGAUUAUAGCUCCGUUCUCCCCUGAGAUGACAGCUGUUCAUGAGUUCUGUGAGCAGUUACCCGAUCAUAUUAGUGUCUCAGACCUGAACCGCGUUGUUGAUAUUGGGUUAGCGGGAUUGACAGUGGCUCCGUCAACACUUGCAACAUAUAUUGCUGUAGCAUCUGUCAGCGAGCACCUCUCUUGUGAAGCAAGGGUCCUUACUGCAAGUUUGGGAAUAGGAGAAUUACCACGGUCUAGAAGGAGAGAGAUCCGUUUUGAGGUUGUGAGAAUUCUCAAUGAGUCUGAUGUCACCUUAAGAGACAAAGUGAAGUUAAUCCAACAGGAUAUCCUCUCGUCCUCGAUCCCGUCUGCAACUGAGUAGAUUUAAGAUUUAAGAAAAACCAGGCAAGAUCAGAAUUAAAUUAAAUCAUCAUCGCCAUGGAGUUCGACGAUGACUUUGACACUGUAUACAUGAACAGGGUUAAGACUUUUGGUGAUAUUCUAGACAGUCCAAUAUUGACAACGAUAAAACGAGAUAUAUUCAACAGCUUGUCGUUAUCCAGAAGUCUUUCAGCACCCAUUUCUAAAAUUUACUCACAGGAUGGCUCUGCAAGCGGGCGACUAAUCGAACUAGCAUUAAUCAAAGCGGUAUGUCAGAGACAAGGUGUCCACCGACUAGGCCACCUCGAUAGAUCUUGCGUGUCCUUGGCAUGCCGCAUAAUGAAGGAACGAGCUCCUAUUAUUGACUUGGAUGUUGCAACACGAUUCCAGAUCCUCAAUGCUCUGACUGAAUCCUCAAAUCCAUCUUUCUCUGACCCCAAGAACGCGGUCUUUGAAGCAAGUAGGCUCUACGACACUGUGCGAGAUGGAAUAGGAAUUCAGCUGAAUUCUAAUGCAACCCCGCUCUUGAAAAGAAAGGACCCUCGCCCAGAACCACUCGAAAUAUACUCCAGGUACUCGAGAGCUGACCAACUUCUUGAAAUGAUCAUCCACAACCGUCAAGAGCAAGGGCUUCAAAAUCGACAGGGAUCAAUAACCUGCAUCGUUCAUCAUGAUUGGCUGUCCAGGAUCUGGGUUGAGGGGGAUGUUGUUUUAUUCAAAGUAGAGGGAGCACUGUAUUCAACCUCGUGGGAUGAUUUUCUGUGCAUCAGAUCAAUGGCUAUUUACCGGAGGAACUCCUUCUUGUUGAUAGCAAUUGACCACUCAAUACCUGACACAAUCACUUCCAGCCUAUUGAAACUCCUCCAAUGGCAGGAGCACUGCAUUUUCUCUUACGGAAAUCCAGGAUACGAACUGGCCAAGGCCGUUGAGUCAGUUUUUAAAGCAAGACUUAUGCAGCUGGGAGAUGGACAGUGUACAGGGGACGCUUUUGAGCUCAUGAUCAUCAAACAAAGGGAAAAAGAGGCUAAAUUGACUAGAGACAAAACAUCGCCCCUCGUUGAAAGAUUGCAAGACUUAGCAUUAUCUGUCAGAUUGCCAAGGGAAGCUGCUGAAUUGUUUGGCUGUCUCAAAUUCUCAGGACAUCCUCACAUAGACCCAGAGAUUGCUUCACAAUCUGCCCGAUCUCAUGGGACAGCAAAGGGGAAUCCUGGGUUUGGUGAGACAAUGCGAAUGAGAGCUGAAUUCUGUGAUAUGCUCCUCAAAGGCUAUAUCAAAAAGCAUGCUCGUUGGCCCCCAUUGAUUCACUCACCAGGCCGAAAGACGAAACUUCAAAAACUUCACGAGAAGAGGGUACUAGUGUUCGGGCCAAACGAUUACGAUUACCAAGAUUGGUACUACUCAGCCUGGCCUAAACUCCUUGACUUCGACUACAACAUUGAUUACCUCGACAUGAUGGAUGAUAAGUCAACUGGUCUUGAUCCUCAAGAUGCGUGGAAGGCUUGGGAUAGUUGCAAGAGAGAUGCUCCCUCGUUGGCACAUAUCCCAAACGAGAGGUCAAAGAAGCUGAUAAUUAGGAUCCUUAGCAUGAAAGAGUUUGACCCAAGGGCUAUUUGUGAACAGAUAAGAGUAUUGAACCUAUCGUUAGCAGAUAUCUCUAUGUCCCUCUAUCCGAAAGAGAGAGAGUUCAAGCUUGAGGCUCGAUUGUUUGUUAUGCUAGAAUUCUCUGUGAGGGUUUUUUUGACACUAGCUGAGAAGAACUUCAAGCGCCUUCUGAAAGAUUAUCUACCUGACCAAUCAAUGACGAAAGGUCGAAAGGGGACGAUGCAACAUCUGGAGGGAAUGGCCGCACGACAAUCAAAUCCCGAUAUUGACACUGUUUUCAUAGAAGUUGAUCUUUCUAGAUGGAAUCUUCUAUGGCGUGGUGUUGUAGUGGAUCCAGUUAGCAAUAUUGCCGAUUCUAUCUUUGGGCUUCCAGGAGCGUUCUCGAAAGGUCACGAGAUAUUUGAGAACUCGACAGUUGUUGUGCGAGUAUCUACUGAAACACCGGAUGGAGUGGUUCCAGGGAGUUUUCCUCGAGAAUGGCCGGAAAGCAAGUACGUUUGGAGAAAUCAUCUAGGGGGAUUCGAAGGAAUCAUGCAAGCACAAUGGACUGCUUGCACUCAAGCUGAGAUCAAGGCAGUCAUGAGGGAUUUGGACAUAGUAUCUUACAAACUGCUCGGGCAGGGAGAUAAUCAAAUCCUAGAAGUAUCUUACAAUAGAGACCAUAACAAAAAUCAGAUGGUUCAAGCGCUUGAAGUGUCAGCUAGGUGCACAGAGGAGUUGAGCAGACGAUUUUCUCGGCUUAAUCAAAUCAUUAAACCAGACGAGUGUCUAGCAUCUCGAUCCACUGUCACUUAUAGCAAGAUUUGUUGGCAGGAUGGAGUGUUGAUACCUACUACUCUGAAACAUGCAGCGACAGUGGCUCCGGUUGGCACUUCUAACAUCCCGGGCUUGGUCGUUGGUCUCUCAGCGAUAAGUUCAGGGUGCAGAGCAUCCGCGGAUGCUUUUAUAGAUCCAUCGCUUGGGUAUCUAUACUUCUUAAUACUGUUUAGAGAAUAUCUCCCUAGAGCAUCUCGAACNNNNNNNNNNNNNNNNNNNNNNNNNNNNNNNNNNNNGUCAAGAUCAGCUGGACAGUGCAAGUACCAUCCCUGGGGAUCUAGGAGGGCUCCCAAUACAGAUACCAACAGACUUUUGCUUCGGAGGCACUUCAGACAGGUUAAGCUCCUCAGUUGCCGCGCUUGUCUGCUUGUCUCAUGUGAACAAGAACUCGCAACAAUACCUUGGUUACUUGGAAACAUCGCUACCCUGGAAACCUGAUCCUGACCCUGCAACUCUUCUCGAAGAUCCUUUCUCCGUCCCUAUACUCCCUAGUGUUGGGGCUGAUGUCCAAGUAGAUCAAGCUAUCAAAACUGUAGUUCCAUCUAUUACCCAGAACAUAGAUCUCAAACAGAUCAUGUCGACCUCAGUGGAUGAAUUCGGAAAAUCUCUGUCAUCCUUCCUAACCCGACUCAGACCAUUUUAUCCGCUGCUCAUGGCAGAUCUUGUUGAGCUAUCGGUGAUUGGAGUCAAGAAGAAAGUCUUCANNUCUUCAAGAAAUUCACUGGGACUAGGACCAUACAGCAGUUGGUCCGGAACAAUGCUCCAAUCAAUUAUGGACAUGCUGUCAUUUAUGCAGACUACAAGAGGGUAACGAGGUUAAGGACGUUCAUGUCGCAAUCCUCCCAAGCGGGUCUGACUGGAUCCUUCCCUUCUCGCAGUAUUUUUUCGAGAGUAGUAAAAUAUCGAGAGAGAUGGUUCCCAGACGGCUCUAACAAGCUAGAAGGAGUGACAGUGCUCCAUCCUCUAGAAGCUGUUGCGGAUUCGGGACUCAAACUGCAGAGUCCAGAUUACUUAGAGUUCACUAGUCACGUCUGUUGGAACGACAUGAUGAGUACGAAGGGACCUCAUCCGGGACGAUGGGGGGAUAAGACCUGGGAGCAUCGCAGGGUGACUGGAGUUGAGGUUAUAGGAACACAGAAAGCUGCACUUGCUGCCAAGAGAUUGCUCAUGAUGGAAUCACAAUUAACGGCUAGUGGGGAACUCAAGAAAGCAAUACGAGGUGUUCUUCGUCAACGGACCACAGUUGAUGAGCAUGAUCUAGAGAUAUUCAUGCCGACUGUUGUUGGGGGAGUGGCUGCUCACCGGUGGGAUAGUACAGUUGAGGAAAAGGCUUUCGCUUGGCUCGGCCCUAUCUCCUUGACACAGCACGCCACUGUUCAAACGGAUUCGAUGAGCAGCUUGAGCGGAGGAAUCAAGGAUUACUCAUUCUGUUUUCAAGAGCAUUCGUUCUUUGGCCUUCAGUGUAUGCGAGCAAGCCCAGAGGCAUUUGAUGGAAAUCCUACUCUUCGUCUUCAUUAUGACAUCUCUCCUGAGAUGCUAAUCACAAGUGUUCCAGUAACUGCAGACGGGAAAGCUCCUGAACUUUCUUUGGCUCGAGAUCUGCAGAAAAAUCCACUCGUCUGUGCCAGCAACAUCUUAUAUCAGGUGCUUAGUGACGAGAUGCCAGCAACUAUAGCUCCAAUGUGGCAAACCUCCCCGUCAUGCUCUACAGAAGUUGGAAUCCGGUUGAUGAUUCAUCAUUUCUUAGAUCAACUUGAAAACCCCGUGCUGUCAGAAGCAGCAGUAGAUGCAAAAGAUGCUCCAUCAGGACUGUCACUCGAUGUAGGGAGCUUGAUCGGCGUGGGACUUCAGCCUAUGAUGGUAGCUGCUGGAAGGGCUGUGUUCUUAAGAUCCGUAGAGAUGUCACUAUCAGAAACAGGUUUGAUGGACAGGGUUAUACUGUCAGCUUACAUCGAUGCUCUUUCAGGAGUUGCUGCUCUCCCGUUAGCUCGAUUCGCGAAUAACCCUCAGGUCCGCCAGCAACCAUGGGUUCAAGCAUCAGGAGUGCUUAUAGCUCCCGGAAAGCAUGGCAGUUCUGUGUUGAAAUCACGAUUAUCCUCCUAUAUCAGAGACGAAGCUCACAGGUGUUACAGAGACAUUCGCAACCUUAGUAACUCUCGGAUGAUCUUGUCUUCGUAUGCGGAGCGAGCUACUCCGUCCAGGGUAUUGGGCUGCUAUGUAGCAUGCUCAAUAGUGCUGUUGGGUCUUCGAGGCUCUGUUGACGAUUGUAGGAAGAUUUAUCGGAGACACUUAAAUGGCCUAAGGAACCUCGUCAGAGAGACAGAUAGAAUUACGCACCACGUUACGAUCUUGAACCUCUUAUCUAACCCAGAGUUCGAAGAAUCGUCAGUUUUAGCAACGGAAAUCUUAGAAGGACGAGUCUUGACGCGGACUGCAAUGAGCUUUGAGGAUAGUAAACGAUACUUGCGAACAGGGCCAAGGUCGAUUGCAUCCCAAAGACUCAUUACCCCUCCUUCACCACCCUCUAUUGUCGCUUGGCAGACAGGAGUAGCUGGAGAGUUUCCAUUAUCUAGCCCAGUAUCGAAACCCCUCUUAACUCUCGACAGGCGGACUGAUAGCUUGCGCUUAGUGAGCAGGAAUCUUGGAUGGAGCAUAGGUGGGUCUUCCAUCUCUCGACUUAGCUACCGGAUUCUUUCGGUAGUCAGUAGUGCUCUGAGACAAGGUCCCGUCUUAUGUGUUGGAGUCGGCAAUGGGGCAAUGGCGAGAGAUGCAUUUGCUCUUGGAGCCACCUGUAUCGUUGGAGUGGAUCUUCUUUCAGAUCUGCCGUCAGUUAGUUCAUUAGGAACAGGAUAUCUUCCUCCCGAGAUUCCGGUGGUAGAUCCAACUCUCAACUGGAGAUGGGCGAAAGGAGUGUUCAACCAUGGAGGUGACUGGUUUGACGAGAAGGUCCACUCUGAUGUGCUAUCUGAGCAACCCAGUGUCGUCUGUAUAGACAUCCAACCGGGUAGGAGGAUGAUAUGGGAGGAUCUAUUACCCAUCCUAAAGAGUAUGAUAAGGACUGUCAUAGUGACGAGACGUGAAUUCACAUCGUCGGAAGCCGCGUUGUUUUAUCAAGAAUGUAGGGGCACAUUUUCAUCAUUUUGCAUAUUCCAAUCCACUAUAAACAAGACUGAGUACUGGUUGGUAGCUCGGACGUCAGGAUGUCAAUCUGUGAGCCGACCUACUAGUGCAACCGAACCAGUUUUUCUUCAAGUAGAUCCUCUCAGUUCCCCUCCGAUGGAUUCGCCAUUUUAUCCGAAUCAGUUCGAACUGAAUAGAACGACUCUAGUGAAGGGAAGAGUGCCUCCUGGAGUGAGUCUCGGAGUAGCUAAGGAUUACCUGUUGACAUUCAUAGAUUGGAGUAUGUCGAAAAGGAGUCAUAGGCGUGGAAGAGAAGUGACGGAAGAUAUCGUACUAGCUCUAUAUCUGCUGAGCAGGCUCAAUGCUAUGUUCGAACACGGUCCGCUAACCAAGGAAGAGCUCACUGAACAGCUAGCUGACCUAGCUCAAAGCUCAGCUCCACCUGAUAUUCAGGGACACCAAGUUCCAGCAGUUCGGCAAGCGACGAUAUUCAAUAUGGCAUCUAGAGUUCUGCCACGACUUAUGAUAGAAAAUAGAAAUCGGUGGUCUCUACAAUUGUGAACAUUUAAGAAAAACCAUACAAGAUCAGACCAUGUCACCAUCAUACAAGGAGAAACUGUCCUUAGCCGAAACAAAGCUCCGAAUGCUUAUUGAAGGGGGUGUUCUAGAGAGCUUGGAAAAAGCAAAAAUAGCAUUUGUACAUGCAACAGUCCAAGCCAAGACACAGUCUUUAUUGAAUAGAAUCCGAGAUGCUGCAGAGAUCCACUCUCUUCAGGUUCCCUCGGAGACCCCACAGCUACCAUUCGAGGCUGAGUGGAUGACUGUAUCCUCUCAAGCAAUCAAAGCAAUAGAGACAGCUGAACUGCUAGUAGCUCAGUUGAGUGAUAAGCUGGCUUCAGAAAAAGCUAAGUCACGGCCCCUACGCAGCGGUGGAUCACUUGAUACGAUCCUGAAACAAAUCCAAAUGAUAGAAGACUCAGCAAACUCCCCUCCAUCAAUUGAUUGCCUCGAUGAUUCACAUUUAUCUGCUAUGGGAGAUACUUUCAAGACGCUGAAUCAGGAGACUCAGAAAUAAGGAUUCUGGUUCGUGAUUUAAGAAAACCUAGGCUCGAGGUGUCUGCCUAUUUGGAGUUUUGUGAUCAAACUAGCAACCAGGAC  >Ss-AA_clean.1_(paired)_contig_4241  CUAUCUACGACAAUUCCUCUCAUCAGAAGGUUCCACCUCGUCAGAUCAACUUCAAUGUAGACUCUGAAUCUAUCACUAUUGGGACCAACCUCUUUCGUCAUUUGUACAAACCUUCUUGUUAUUUUUUCCUUAGAUGACGUCAUAGUUUGUUGUGGAAGGUACUUCAUUACUUGCUUGAGAUUGUGCUCUGCUAGAGAAAAAUACCAUCUGACUUCGAAUACUAACAUACAAAACAUUCUAGGAGCGAUUUUAAACUCCUUUUCUUUCGGGUAGAGACAUAUUAUAAGCCAUUCGAAGGGGAUUCUCUCAUAGAUCAUUUGGUGAACUAUUUUUUUAGGAUCCAUAUCAGGUCGUGCGAGCAUGGACAGUAAGACUUUGUGGGAGUAGAUUCCAGUUUUAUGAUUCGAACCAGUGUAAGACUCUGACAUUCGCGUUCUGUAUUUUGACAUAGAUUUAUCAUCCAUCAACUCGAGAUAGUUCGGAUAAUAAUCGAACUCUAAGAAUUUUUCAAACUCGCAUUCUAUCCAGUCAUCUAGAGGAUAAGAAUUCUUUGGAAGAAUGGGAACUCUUCUUUUGUAGAGCUUAUAUAGUUUAGUUGCUUUUCCUUCCUCAGAAAAUUUAAGAGGAGGCCAAUCUGAAUGCUCUUCCACAUAACCCCUCAAGAUCAUUUCAGCUACUCUCUGUUUGGUUUUGAACGCUUCUGGUAGGUCGAUGUUUCCUUCGGAAGUCCCGUGUUGCUGAGCUGACUUACCUCCCACAGCUGGGUCUACUAUCGGGAAAGAAGCAGUUUUGAUUAGACCUCCCAAUUCACUUAUAAACCUUAAGGAUGUAGUGGCCUCCAGAUUUCUGAUAAUUCUACGGAUGAAUUUUCCUGUUCCGCCUAAAUCCCGUUCCUUUUCGUAAUACUUGCGUUUGACAUUAUCAUAAGAUGUAGUGAUAAUAUCAUUGCACCGAACAGAUAGUAAGCAUUUAGCUAAAGCUUCUGGACUCUUAACUAAGUCAUAACCAAGAUCACCAAAUCUAGUCACAAUAUCUUCUUGAAUAUCCAACUGAAUCAAGAUGUGUUUCGAUAAUCCCGGAUAUUGAGCAUCAGCUCCAUACAAGGCCAAUGCUCUAGUACGAUACAUAUCUUGGAAGCCUAAUAAGUCAUCAUAAGAUGUUAUCCAUGCUCCUUCAGGAUAUCUGAUAAGUACUAUUUGAUCAUCUAUCAGUAUUUUGUAACUUUUUGUUUCUAUUAAAACCAAGUCACGCUUUCCUUGUUUCUUAAGAUUUCUUAAUCUUUGAACCCCGUUUUCAAAAAAUUCGUAAGCAGCAAGAUUUUGAAGCACGUGGUUCGACAACUUGACGUUCUGAGGAAUCGCUUGAGAUAAUCCAAAGUGUUUGCUCAUAUUGUGAUUCAAAACAUUGAUCAUUCUAUUAGAGUCUCUUAUAUCCUCGGCCAAUUUAUCUAAGUAAGACGGUUUGAUCUCUGAUUCUAUCUCUUGAAGUAAACUGGAUAGAAAUACAAAUCGAUCAGUCGUUUCUAAUACUGUCACAGACACCUCGUCUAUCUUAGGAAACCUUCUUCUAAAACGCUCAAUGGUUCUGAUAGUAUUCUUAACCAGCUUGCUAGGACUGAACUUCACUUUCAUUCUCAAGGUAGACACGAAUCUAUCACUCGAGUGCCGCAUUAACGGAGAGUCCAAUAUAUAAGAUGGAAAAAACCGUUGCAUUGCUCGAAAUUCAUCAUCGUCGAAUAAUAGAUUUUCCAUGAUGUAAGAUGUUUUACUUAAUGUCUGGUCCUAAGUUUUCUUAAAUGAUAGUAUAAGUGAGUCAACGAGAACGAUCCAGGUGGAGCGACAAUUUAUCCGAAAGAAAGAAUACAGCGAAAGUUGACGUUUGGAAGUACGAUAAAGAGCGACAAACUAUAAGAGGUUAAGGAUGACAAGUGAAAAUCGAAUCUUGACUGCGAAGAAUCACUCAACUGAUUUGACAGGAUUAGUAGUGACAGUAACUUCGGGAAGUUGAUUGAUGGAUUUGAGGAAUUGGAUGAAUUCCUGGACAGCUCCUCCAUGUUGGGCGGACAAUCCUUGAGCAUAGUUGGCUAGUGUUGGCUGCAAGGUCACAUGAUACUGUUUGGCACAAGCAACGAUUAAUCCAAGUUCAUUAGAUGUUGGGAGUUUAUCUGCGCGAUGCUGAAUUAUUCGGAUGUAGCCGAUUUUCUCUGGAUCUAUCUGCGAGUAUACUUCCAGUGCUCGGGCCAAUGUUGGAAUGUAAGGUUGGAAGGGCAGAUAAUCAAUUACCUGUGGCACAGCAGCAAUCAUAGUCGCGAUAAGGUCAAUGUAUUUGAGACCAGCACCGUCCAAGAGAUUAAACAUGGUAAUGAUUGGCUGCUUAUUAGGAUCAUUAUGUCCUGAAGUUCUCCAAGAGAAAAAUACAUUGGCAACUGCCACGCGAAAACUAACAAACCAGUUCAUGCUUCCAUAGAUGUACUGAAUGGGCUCAACUGUAUCAAAUUUGCUUUUCAUUACUCCCAGUUCAAUGUCUGUCAUAGCGAACAUGCUUGCAAAAUUUUGCAACCUAUUGUGAUAAGCUUGAGGGUAGUUGGCUUGACUAGGUUGCUUUGCUAGAACGUAGACUAGAAGUGCACACAUACAUCGGAUAUGGGUGCUAGACAAAUUUUGAGGUAUGCCUUGGCCAGAAAACACUAUAUUACUCACUGAGUACUCUUUGUAAACCGGGAGUUUUUUGGAGUAGUUUUCUUCGGAUUCUUCCGCCUCUUUGGCUGGCUUGCGAGCAACGAAGGCUUGUUCAAGUUUUAAUGGGUCAACAGCAGAUGGCAACAAUUUCAUACCAUAAGUGGAGGUAACUGUUUGUUUUGCAAAUCCAGGGUGAAGUGCAUCAAAUAUCGCACCGUAGUAUUUGAUCACUCCUUUGUGACCCAUGAGGUGUGCAUCUUGAAGUAGAACUAGGAGCAUGUUCAAGGCAGCCUUGCAUCGAAUUUUUUCUGGGAUUUCCUUACCAAAAGUUACUACGACAACUUCAUUGAGAUCUCCGGGGUUGGUAGAUUGAUUUACAAUAUCUGGCCUAGUCGACUUGAUGUGAACUUGACUGGCAGUUUUAGCCAUCGCUAAAGCUCGAGAAGAAGGUUGAUGAGACAUGGUGUUAAGCAAGAAUUUAUGAGUUUUGCAAUAGUGAAUGGUUAAUUUAAUAUGUAUCUGGUCCUAAGUUUUAUUAAAUGACUUGAUAGGUUAAUCAGGAAGGAAUGAAUGCAAUGUAAGAAGAGGUCAAUUUAGACUUUAAGAGAAUGUGGUAAACAAUAAAUAGAAUAGCAUUGAGUAAAGAAAUAGACAAAGAAGAAGCAAGAAAAGAUUCAAUCUUCAAAGACAACUCUGCGACGGAACACAGUAGGAUCAGGAUUAUUAUACGCUUCAACAGCUGCUCCGACAUCAAGGUCGGAAGCAAUUGACAUAGCAACUGAAGGUGUUGGAGCUGGAGGAGGCGGAGAAGCCGGGAAUCUCUGCAACUGUGAAAAUUCCUUAAUGAUUUGCUUUUGACUCUGCAUCAUUUGAGCCAAAUCUCGCUCUAGUUUUGCCAUUCGGUUAUUCUGCAGGUCUCGAUAUUCUAAUAUCUCGUCAAUUUUUGCAUUCAAUCGGAUAAUUUGAGAACUACGCAUUUCAAUAGUUUUGUGUUGAGUUCGAACUAUAUCAAUUAGUUGUUCUUUAGAAAAAUCUGUACUAAAUGCGUCUUCAUCAGAUUCUAUUUCUUCCCCCGUUGUUUGUUCAAUCCCGGGCGCAAGAUCAGGGUUGGUAGAAUCUACCGGAUCCAUAGUUUCUUCUGCCCAGGAUUUCCUAACAGGAGAAUCAGGGAUAUCAGAAACGGGUUGAGAGCUUCUAGCCGCGGAUUGUGAUGUAGUUGCUUUAUCUUUCUUCCUUUGAAGUCUCUUUUCUGCUUUUUUCUCAGCUUUGGUCAGCUUGAUCAUUGGCUCAGAUUUCCUGGUCAGAGUCGGAGUGAUAUUUUCUUCGUCCGAGUCAACUGCGAUAUCUGUUAAGUCGGGAUUGUUUCCGAAGUUGGUUAGUGGAACUUCAAUUUCAAUGGGUUCCGGACGAUAAUGUUGCUCUGAUGGUUUGGAUUCCAUCAACUCUAGUUCUUUGACUCUACCCUGAAUAAGACUCAGUGCAGACCUUGCAUUGGCUUGCGAUUGAAGUAUCUCAUCUUUUUUGAGAUCAUCGACGUCUUCACUUGCGAUAUCUAUACUUUUUCCAGAAUAACUCAUCGUCGAUUAAUCCGUAUUGGCUAUUUUGUUAUAAGAAUAUACCAGUGCCAGAUGAACGAUACAGGAUGAACGUUAUUACUUUUUGUUUAAAGGUAACAAUUUCUGGUCCUAAUUAAUUGAUGAUUUCCUAGAUAGAGUAGCGUGUAGAUCGUCACCUGUUUGGGUG  >Ss-AA_clean.1_(paired)_contig_89  AUUAACUGUAGCUACUGUCUAUAGUACUCUCGUCCUUGCGUCUAUCCGACAACUGCAUAGCUCUUGUCCUCAUACUGAUAUAUUUUAUAUUCGAUAAUUUUUACCAUGUCUGAUUCUCCAGAAACCGCACUGCCGUUGAUUGACUUACUUAAUGUCGACAAUGAGUCCCGCGCGACUUCAGCCCCAUCCAAGGUACCUGCGACUGAUGAGCGAGGGGAGCACGAUGAGCCGGAAACUACCUCGUCGCCACCGAGUAAACAAAAAGCUCCAGAACUUGAUCCCAAAGAUAUUCAAAGUUCUCGAUUGCCGUCGUCUGUUCCGAAAUUGAAGCCAAGUUCAGAGAUCUAUACUCCGUCCAUGACUGAUGAUGAAAAGUAUAAACUUCAAAUAAGUGUGAAUAAAGCAGAAACAACAUUCAUUGAAGACCCUCCUGAAAUUGACUACGAAGACCGCAUAAAGAAGAUUACUUCUGGAGGAAUUUGGAGCGAGACAGAAGGAUUAGACUCUCGUGACUGGACUGGAGAAGUAGGUAAUCCCUCAGCUAAUACGUUGGCGGGAAAAACUUAUCAGCCACGGACUCCGUCAGAAAUGAAUUAUGACAUACUGUCUAGUGCUGGUUUGAAUACGGAAGUACAACAUGCUCUUAUGUCAAAAAUAGAGUUGAGCAUUUGCAGAAAAGCAGGAACGAAAUUCUCUACUUUGAACGAGCUUCUCAACUUCCGACUUCAGCAAUUAAACGAGGAAAUGAAUGAUAAACUUGCGACACUCACAUCAGAAGUUAAUCGUCGCAUUUUGGCUCACGACGAUGCGAUGGGUAAACGAGUCGCGAAACUUGAGGAAAUUGUUGCAACUAUCUCCAUUCAGGUGGAAGGAAUUAAAGAGUUGAUACCGAAGAACGGACCUGAAGAAGUUGCUGUUCUUCAAGAAGCAUAUAACAUUGUCAAACAGCAGCAGCGAGCAGCAGACUCAGAGGGAACACCUAGUGUUGCAGAACGACUGGAUGAACAAAUGGAACACCUCAACACUACAGCUGCCAAAUUACAGCAAAUGAGCAAAUUAACGGUGACUCCGUCAGUAUCUGGACCUUCUGCUCCUACGUCUGCUUCUGCUGCCUUCGUAGCAAGUCCGGCUUUUGCCAAGAAACGCGGACGCAGAGGUCUUUAAUUUAUGAGAUAUCUAUUAUUGGAUAUUAUUUGUUUGGCGGUGUGUAUUUCUUUCCGAAGUCGAAUUCAGUCUGUCUUUAACCCAUCUAGUGAUCAUUCUAUCUUGUCUUUCAUUUAAAGAAACUAGGAGAAAUAAAACGAUGAUUCCAGAUACAGCAAUUAAAGAGUGGUCCACUCAAUCUAUCAACCCUCGUACAAACGAGAAAGUUGAGCAUCCGGGCCUAAGACACGAACAGUCGGCGAUAAUGUCUUUAAAAGUUCAGAUUGAAUACAAUCCUUUGGUUCGAUUUUAUUCAAAAGAAAUAGCCGAUAUUAGUGAUGCACCUGCAGAUCAAGUCACGAUCGAAACAGAGGUUAUUCAAGAGUGGAAUAAGUUAGAACGAUUGUUGGACACCUUUGUAACUGAUAUUAACGAUAUCAAGAUUACACGAGCGUGCAGGCGCGAAGCUACAACCCUUGAUGCUCUCACAAAAACUCGAGUCUGUUUUUCUCAAAAUGACUUGGUCGGUUAUAAAUCACUAUUAGACGGAUUGAACUCAGCUUCUAAUGAUUUGAAAGAUUCUACAUCUGACAUAAGUAGUCUAAAUAUGGGAAUCUGAAGUGUACCGAACGGUUUUCAUAUCGUAUUUAAAUUUAAAGAAACUAGGAGAACUAUAAUGACUUCGAACAAUGUCGAGCUUGAUGAUCUAUCCGUACAGCGUGUAUCAAGCAGUAUAUCAGAGCUUCGUGGCGACCACCUAUCUAGUAAGCUAUCUGCUGGCCAUACAAUUCGUGACUCAGGUGAGUUACACCCUAAUACCAUACUUACUCUCGCAAAGAACUCGACUACAAUAGUCGUUCCUUACGCAACUGUUCCAAGAUAUCCUCUUCGACGUGCAUCGGCGUUAUCAGAUCUGACAAUGAAGAAACCAUCUUUCUUCAAGCGCUUGUCAUUGAAAUUCAAGAUCUGGAAGAAAUUGAAGCUUUUCCAUUCUCGAAGAAAAAUAUGACUGUUCAUACACUACACUAAAGAUCAGUGCGAACUGAUAUUCUACGGUGUUCUAUUUUACUUAUUUGCUUGUCUGGUGGUUAUUUAAUUUAAAGAAACUAGGAGAUACUCUUACUACUUUACUCAUCUAACAAUCAUUAUUCGUACUUUAAAAACUUAAAUCAGUUGUCCCAGACAGAAAUUUGCAAUCGUCAUGUCUUAUGCAAGAGCACAAGCUGGUCCUUCUAAAAAGUUCUACGAUGAUGAUGUUGUCGAUAUUAGUGGUUCAGGAUUCAAAAAAUCAAUUCCAGAUUUGCCGAAAGUCGUCGGUGAUUUCCAAGUUGUCAAUGGCGGUGAUUUAUCAACUUUGGCUUACUUAAUUCUGCAGAUCGUGAAGAGCUCCGAUAAAGUAGUGAUUAUUCGCGCUAUCGCGUCUGCUGUUGGCUGGAUUUUAAAUGAUUCUAUCAAUUAUACUGGCAGCGAUACGGCGCCAGAAGCUAUCACCGGACUUGAACAUGUGUUUGCAGACGACAAAAAGAAUUCUCCUGAGGUGCUUGUCAAAACAAAUUUCGCCGAUGAAGAACCUGUUUCUUAUGGCGAAAUUUGGACCUUACUAAAUGCCGAUGAUGAUGAAAUGGGUUCUUUCAUCGGAUUAUUAUUCUUAGCUGGAGUUAAACAAAUAACAGCUCAAAACAGAACCGCGUUCAACGAGAAGCGUGCUAAAAGUGUCGCAUCAACUCUUACAAGUGCUCCUCUCAUCUUCGUCCCUAACUCCAUUUACCUCAGUGAUCGAACUUUAAAAGCAGUCUACGCUACGUUCACAUCUUGCGGAGCAAUUCGAGCCAACAUGCUCUCUUUGUUAAUCGCUAAAAUGAGCCCAGCACAUAUGGGAAAGGCUCGGUCAAUGUACUCGAUGAUUCUACUACUCGUCGACUCUGGAAUGAGUGGUCUCAAAAUAAUCAAAGAGGCGACUAUCAAGUAUAGAUGGAUUGUUACCUCUUUCCCCGAACUUGCCCCUGAAUUAGAAGCUGCUAAUAAGGCAUUUCAAGUAAUUGGCGCGGCACCUGCUAGUACUAGACCGUUUCUUAAAGCUAUUCAUGGAUCUGAAUUUGUUCCUGUAGCUUACGCUGAAAUCAAUAAUUUAGUCGGGGUAUGCAAGGAAGUAUUGACACGCACGACUCCUUCUUAUAUGAAUUAUCACGGAGGCGAAGCAAAUGCUCAUCAAAAACAAAUCAUUGACUCCUAUCUUCAGGUUGCGAAAGACGAGCCAGCGUCUGUACAAGUUGAGUGAUCCGACAGUUAGAUACAUAGAUAUUGAGCUCAUUUCCAUUUCGUGUUCGUUCCUCCACUAUCAUUAAACUGACUCUUGUUGUCCUUGUCGUAUUUCUUUUAGUCGCUCUUUAAUUAGGAUUUUCGAUUAUUUUCAGUUUGGAUCGCUCGCACGCUUAUAUUUCAUAUCUGACAAUUUAAAGAAACUAAUGAUUAGGAGCAAUAAUGUCUAUUCCAUCUGCACGAGAGAGAAACAAUUAUAAAGCAAAAUAUUUAGCUGCGACUCAAACGCCUUCCACUACAGAGCUAAAAGAGAAAUUGUCGACUGUGAGCGAUCAAAUAACUAAUACUCAACAGGAAACUGUUAGGAAGUUACAUAUUCUUCAGCAGCAGACGAUAGAAACUGCUAAUCGGGUCAAAACGUUAGUCAACGUACUUGACACGUACCCGAUUAUUGCUGGGUGCGAAUUUGAACGCGUACAUCACCCAAUAGAUGAUAAGCUACAACAAAACGAUUCGAUCAACGUGCUAAAGAGAACUAAAGAUGUAGUACAACUAGCAUCUACACGAAUAGGAGUUCUUAGCGAAGCUCUUAUGGCAAUUUCCGAUUCUUAUACUAUGCUUCGAUCUGAAUUAGACAAGCUCUCGGGAAACGUGGCACCGUUAAGCGUCGCAGCGGCUCACUCGCUUUUAAUCGGUCCAGAUCCUACCAGAACAGAGCUAGAAAAUUACAAUCCUGGGUUGAUUCAGACCCCGAUAAAUAGAUUUGGUAUAGAAUACGAAGAAUUGGACGAAGACGCAUCAAUCUUUGUCAACGACAAAGCGACGAAUAUUAAACCUUCUACUACUUCUACCAGUAACCGGAAACCAAAAAUAUGAACCUAAUCACGUCGUCGAGGUUCGGUACGCAAUAGUUAUUAGUUUCUUUAAAUUAGUAUAGUAGUAAAUUUAAAUUUAAUAAAACUAGGAGUAAUAACAUGAAUUCAAAUACCGUCGCUAGAAAAUAUGAAGCGCUCGCAUCACUACGUACCAAACUUAAUUAUGCCUAUGGAUAUAUGGAUUUGGUUGAUCCUCGUUGUCGCCUUAGCGUUCGAGUUGAUGCUAAAGCGCGUAAUAUCGCUGAAUGUUAUAUUCAUGAGAUAUUGUCUAAAUCUACUUGUACACCCGUUGCGGAGUGUAUUCGCAUUUCUGAUAUGCACAAUCGAGAUAAUCGUCGUUUGUUUGACUAUAUGCAUGGACUGUCUUACGUGACAACGUUUUAUUUAGAGAACUUAGAUGCGACAGAAGGAGUUGAUUCAGUCAAAUUGAUUAAUUGGGUUGAAGAAUUUAUCGCGAUCGUAUGCGAAAUCAAAAGCAAGUGAAAAAGAGAACAAGAAAAAGAAACUAUUCGUUACAAGAUUAACAUACAUCUUUAAGUCAUGUAUAUAGCGUUGUUGAUUUAAAGAAACUAUCAUCUAGUCAAGUGCGGAGAUCAACGGUAAAUUGUAAUCAUAUUUAGAUGCGUGCUAAAUAUGUCUCAGUACUUGUGCGGGUGGCUAAAGGAUGUAAAAGUUAACCGACGGCUAUUCAUGUAUGAUAAAUAACAAUAUAAAUUUUCCGUAGUUUUCAAUACGUAGACAAGUGGAGGGGCGUUCGCAUUAUGCUUAUGAUAGGUAGCUUAAAUUCGCGGAGCUAAGG  >Ss-AA_clean.1_(paired)_contig_579  AUUAACUGUAGCUACUGUCUAUUCCCCUAACCCAUAUUGUCUACCCGCGACGUAAGUCUUGUUUACGAUGUAUGAAACAAUUGUUGAAGACGACGAUUUCGUCAUUGACAACUUGGACGACAAUACAGAGGAGUAUCGAAAUAUCACUCCUCUUACACCAGAAAAACAUCUGUCAUCGCCGAUUACUACAACUUUACUUGAGAGAUUGCAUAAAAUUCUAGAUACAAUUGCGAUUGACGAAAAACACAUCAAAUUGAGUGGACCAUUUCCUCCUCGAGUAAACGCGUUUACUAAUUACUUUCGUCGAUAUCCGCAUGAAGCCUACUCUUCUCUUAAGAAAUAUGCAUAUCAGUUCUGUAGUGCCCUCGAUUUUUGUGCCGUUCGACCACAAACUUUAACUGCUCAUGAUUAUCCGUUCUUGUUUUCAUUGACGGCUGAAACUUCAUAUCGAUUACAGACAGAUUUCAAACUCGCUAAGGAUAUUUUCGCUCAAGAGUUAGAUGCAUAUUUAUUUCACAUGUCUGGAUUGUUGAACCACAAACAGAUCAAUGCUCUUCGUGAAAAAUUAACUCCUCCAACUACACUCGGACUACGAGUGGUAACUUCUGCUCGUCAGGUACAAUUAUGGUCAGACAUAGUCGAGAAAUAUCGUUACAACUAUGCAAAGAACAAUCAGAUGAGAAAAUUAACGAUCGGUCCUUUGCGAGUUAUUCUUUGUGACGGUUUUCUACUAAUCAAACACGAUAUAUUUACUGAAUGGAAACUCCUCACCUUCGAACAAUUACAAAUGAUUCAAGAUUGCUGUUUAGCGCGACAUAACGUAGAGCUCGCGCUUCAAUUCAACUUCCACAAUGGAACUAAGAUGCUAACGCAGCAUGUAGACUCCAUUCUGCGAUGGCAAGAAGAUGUCUUAGUCAAAUUAGGAAACGACGGGUAUGAACUAGUCAAAGCUCCUGAAUCUGUCUUCAAAGCAUGGAUUAAUAGUCUGACUAAUGGUGAUCUGCUUACUUACUCGUCUUACGAUAGAACGCUCGAUAAGAUGAGGGAUAAAGAACGCGAUCUCCAGUCCACUUACGAUCUAACUGGCGCAAUGCAUAGGAUUGUAUUGACUGUUACGGAUCUUCAUGAUGCAGCUGAAUUAUUUGGGCUGGCAAAGCUUUCUGGCCAUCCGACAGUAGAUCCGUACAAGUCUGCACGAUCAGUGCGUAAAGAAGCUCAGGGUCGAGGACGUGUACUGCCUUUCGCUAUGAAGCAAACCGUUCGAAUGUUCAAGCAUCUAACGAUUUCCGGUUAUUUGAAUAAAAACGGCGAAUGGCCUCCUUUUUUCUGUGAACCUGCGCUUGAUACAACCUUAAGACGCUUGUAUUUGAACAGAGUUACCUCUCUUCCAAUUGGUUGUUAUCCUCUAAACGAGAUAGAUUCCAUUAUCUUCGGAAAGUUCUGUGAAGUAGAUUACUCAGAAGAUUACCUUAAAUUUAUGGAUGAUAAAGCAAUUUGCCCUGGCGCAAGUGAGAUGUCCAAAUUUUGGUUCAAUGGUCAAAAGGAUGAAACUCGACGACUACUUCAGAAAACGAUAGAACUGCAUCAUAUUGACAUGAUUGCCUUAAUUGAACGCUUUAGGAAAGGUCAAUUUAAUGAAGAAGAACUCGUGAUCGAGCUGACACAAAAAGAACGGGAACUAAAAAUUGCAGCUCGUUGUUUCUGUAAACUUACAUAUAAUGUUCGACUAUUUUUCACAUAUACUGAAUGGUUGCUUAAGAAACAAUUCAUGGGUCAUUACAUGCCGCAGCAGACAAUGACGAUGUCAAACACGGAGACCAAGCAACGACUCUACAAUCUGACUAGAGGUGCCAAGGCCAAAAAUAGAACAUUUCUAGAAGUUGAUUACUCUAGAUGGAAUCUACGUAUGAGGCACACCACCGUUGAUCCUAUAGCAGAAAUAUUGAAUGAUAUUUUUGGGCUUCCAGGUCUUUUUACUCAAGCACAUCGAUUCUUUGAACGCGCUACUGUUGUGAUGACUGAUAAACAUUCUUUGCCUGCUGGUGCUGACCCAGAUGUACCUGUCACUAGAUGGCCGGUUAGUGACUUGGUAUGGAGAGGAACACAUGAAGGUGGUUUCGAAGGUAUUCAGCAAGCUUUAUGGACAACUUUUACGAUCGCCAUGAUGUACUGGGUACUGUAUGAUCAAAACCUCGCUUUCAUUAUGGCCGGUCAAGGAGACAACCAGGUAUUUGUCCUGACCUUCGACGAGUCAGUGGAAGACUCUGCUCAUCAACUUCGUAAGCUGCUAGCUAUCAUGGAAGUCCGAUGUAGUUUCUUGAAUCACACAGUCAAGCCGGAGGAAUGUAUUGACUCUCAAACUGUACUGACUUAUAGUAAAGACAUAUACGUGGACGGCAAUCACGUGUUGUAUAAUCUAAAAUUCGCAGCUCGAUCAUUUCGCAGAGAGGAGGUAGACAUCCCAUCCUUAUCCACUGAGAUAUCAUCAAUCAGUGCGAGCGCUGUCGCAUGUGCGGACAAUGUAUAUGAAACACCUCGCGCGAUUUUUUGGAAAACUUACCAGACACUUCGAUUCCUUUCUAAUCGCCGGUCAUCAAAGAAUUACGAGCCAGAACAUGUUUCAUUAUCGAAACUUCUUUUAAAUAAGAAAUUGUUAAAGUUUGCAAUCCUACUUCCGGGCUCUUUAGGUGGAUUGCCCACGAUGUCCUGGACACGAUUUUUUAUAAAAGGAGAGGUAGACGAUUUGUCUUGGGAUGUACCUGCAAUUUUAACGUUGGCCAAAGAAGACCCAAUUUUCGGAUGGGACAUGGCUCUCUUAUUAGAAGGAAACUACACUAGUUCAAAGCCAAAUCUUCAACAGCUGAUACUGGAUCCGCAUUCUAUUCCUGUAGAAAGACCGAAAGAUCUUAAGCGAUUGGUCAAAGACGCAAUUGCCCAACGAUUACCUGCUCUGACGAAAAAUGAAUGGCUAUAUCAAUUGAUAUCAGACAAAGACGACGCAGCUGGACGAUCGUUGAUGGAAACAUUGGCUACCGCUCGCCCGUUUUACCCUGAAAUCAUGCAUGAUAUAUACGCGUUGUCUCCUAGCGGUAUUCGCGAUGCUAUGCUCGCUCGAUUCACACUGACACGUACGAUUAGUAACAUAACUGGUAAUCCAAAUUUUUCUCGAGAGAUCCUAGGCGCAAAUGCCCGUUUACUAGACUUUGUUAUUAAACGAUAUGACGCCGCAGCAUUGAAGAGAGGAGUGCCUGUAUUACCCAAGACAGCUUAUGACACCUGUAUUCGUUUGCGAAAGUUAUGGGGUCCAGACAUUGAACAUAAGAACAUAGGCGUGUAUAACCCGUUCGAUUUUAAAUUGAAAUUCGCUGACUCUUCUGUUCCAAUGAUUUCUGCUUCCUCUAGAUGUGCAGGAAAGUCAUUGACGACUGAAAUCGGCCCCUACCCGCCCAAUUUCGGUACUCACACACGGCAAAAACUGGGAAAUCACGGCAUUACUGUUAUCUCCUCUUCAUCUACGGUCGCAGAUCUCAAGCGCUUAGUUGUGUUGUUCAGUGAACUAGGAUCGACACCUGAGCUAGACGAAAUCUUAUCUCGCAUUAGUAUUGCUAGAUGUCCGCGCACAAUUCGACAACUGGCAACCAUCCUACCCACUGCUUAUGGAGGCACAGCUGCACAUCGCCAUGCAGCGUUGAAUAGGUCUGCUUUUUCCAUCCUUGGUUCUAGGACUGUUCCGACACAUCUCAAUUUCUGUACCGAUUUAGCUGGAAAACUCAGCGGUGGUGAAUUUGAUUAUCCAGUGGCAUUUCAAGAGUUCACUCUUGUUUUAACAAAUAUCUAUCAAGUCCUGACAACUUAUGAUUUAUUAGAUGUUAAUGCCUCAAUAGGCUUCGCCCUAACAGACGACUACGAGCCUCUACCUACUGAGCCCGUAGUUUGCGAUCCUCCUAAAAUUGAGUGCAAUUGGGAAGUCACCACGAACAAUAAGCUCGUCUAUAUUAGCAACUUCCAGAUUGCAGAACAGCCUGAUGUGCCCGCAGUCAAUCAAGUUCCUCACGUCCAAUCCAAAAAUAUUCCGCCUGCCGUCUUAGUGUACAAUAAACUGCUUGCUAAAUAUUGUUCUAUAAGAAAACUCUUCUCCAGUACAUCUGUGGUGAAUCUGCCGGUAGAUUUAAUAGAUAUGAAGGAAUUCACACAUUGCCCGUUUCUAGACCUUAUAGCAGGCACAUGCUAUUUCAUUCAAACAAUGUCUGUUUAUUUGACUAUUACUGAGUAUACGAAAGAUGCAAACGUGUUUUUAUCUGCCACCAUUCGGAAAAUGUGUAAUUCUUGCUCUGGACUUCUGACCCGCAUGAUGCUUCACCCUCUCUUCCAAGGAACGCAACAAGCAAAAGAAGCUGGAGUACUCUGCACUCCGGGACAAUCAGGAGCGAGAGCGGCCGCAGAUAACUUCGCAGGCGAAUUAUACACUUAUACUUAUCUGAAUAUUAGCGCUCGAUCAAUGCUCACGAAAAAUAUUCCUCUCAUCCUAUUUGCCGAUUAUAUGUCUUAUGGUGCGACAAUUAGUGAGAUGCAUGCUUACUCGCUAAUAGCGAUGCAGUCUUACGAUCCGAAAAAGAUAUUAGUUACGGGUUAUCAACGCUCUAUGUUAGUUGCAGCGCGCCAAGGUUUGUAUUUCUCAUCUCGUGCCUUAACUGUAGCACUUAACUUUAGGGCGACGGUCCAUGAGUUAGCAAGAGGAAGACGUCUCAGAAAUGAACAAGGUGCAAACAGUUUUGAUACUAAGUUUGUUCUGCAAUAUUGCAACGAGACUCCAGAGGAAGCUAUUCGCGCGUUAAGAGAAUUACCAUUGGAUGAACGUAGGCAGAUUACGAUCCUUGAUCCUCCUCCACUCUCAUUUGGACCUACAAAAGGGAAAUGUAUCAUUGAGGACACAUCUUUUGACGGGUCACUACGUCCGACUCACACUUGCUCAGAUACAGAUGAGCGUAACCGUCGAGCAGAUAGGUUUCUGUCAUUAUUGGUGAGACCAUUAGGAGUAUAUAGUAGUGCAGUAAGUGUAUGGUUGUCGAUAUUCAAGAUGUAUAAGAAAUCAUUCAUCAAGAAAAGAGUUGUCUCCAUAGGAGUAGGGCAUGGUGCAGUUGCAGCAUCUGCUCUAAUGUUAUCAGCAUAUCAGGUAUACGGUAUCGACUUACGAUCCUCAUUUCCAAUUAUAACACAACGAGAAGCUACUUAUAUACCCCCUGAGGUAGUAGAAACAGGAAUGCUGGCUAAAUUUAAAUGGUCGAACUUUGUGUCUACUUGCGGCGGAGAUGUGAUACAAAAUGCGGACAAAUUGACAGAUACCGAAGCGGCAGACACAUGGGUUAUAGACAUUGAGCAAGAUUUUAAUGAAUUGGAACCAGUAUUAGAUCACGUACCAAUCGGCGUAACAUUGAUAGUUCGUUUCAUAUGCUGUGCUGACUGGGCUAUGUUCGCUUACGACGCAAUGAACGCUACCGGUUGUUAUAACACUUCGGCUGUUCGUACCACGCACAAACAGUCUUACAUUAUGAUCUGUGAGAACUUUCGAGGAUAUAAUAAGAACGCAAAUUAUCAACGAUAUGCCAUUACAUCUUGUUCUCCAUGGAAGUCUUCCGUAAUUAAAGAUCUAAAAUAUUCUGUAAGAAGAGUCAAUCAUUUUCUACGACCGGUCGGCGAGUCAGUUAAGGUAAUUUCGUCUCAUGAACUACUACGAAUAUCUGAGAAACUACGCAAUGACGCCCUGAAUAGUCCAUACGCUGAUAUUCGAGAAAUUUUAAAUGCCAAAUCUAUCGCUCUCUCCAACGUUGCUAAUUUUUUCUUGGAUCUGCCAACGAUGGUCAAUGUGAAGGAACGUAUUCAUUUGUUAUCUUCCGACGAGCAACGCUUGUUCGCUGGAUGGUAUUCUAACACGCAAUUACCUUUGGAUGAGAUCUCAUAUCCGCUGUUGUCCUGAGUUUGAAUGGAUCAUUUAAAGAAACUAGGAGAUACAAUAUCUACAUAUGUUCGAUAAUACUGUUAUUUCGUUAAUGAUGGCAAGAUCAAUCAACUCAAACGCAUCCGAUGAUUCGUCACUUUCAGGUCAUUCUCAUUAUAUCGAGGAUAAUGGAUCCUCUAUGAUGGCAGUAUUCCAAACAUUAGAAGACAUUCAUGAGCAGCUAGUAUCUGUCUCACUCUCUCAAUCAAUUCGAUGGGAGUUUAAAGAGGCUAUGCGAAGACAUAAGAAUAACUUUAUCGCCGCCGCUUCUAUGAAUAUUCCGGUUCACAUCAAUAUUUAUUAUAAUUUGUGCGGGUUUAAUGAGUCCGAAACACUCGAAGAAGUUCACAAUUUUUACUUGUCUAUGGUUUCAUCCUUUAAUUAUAUGGUUAUUCAUCAGGUAGUUCCCUUAAGCUCGUCUUUAAGAAGUAUGGAUCAAGCAUGCCUCGACUUCUCGAUUGCACUUUACGCAGAGUUAGACUCUAUCAUCUAUAACGGACCAACUUCCGCAAUAGUAGUAACAACGGGAAUAUAGUUACCGUUUUCGACUAAGACAAACGGGGAUGCGCGCGCUGUAGGUUAUUUAAAGAAACUAGGAGAUAACGUUGCAAAUUUAUUCCACAGUUAAACUACUAUGCCUCGACGAGCCCUAAACAACAUAUGUUAUGUCACAAUUCCUCCCUUGUCCGGGCAAGAACUAUUUGAUAGUCAUAGGUUUAGCAUCUACAAUGCAGAAACAAUUGUACAACGCAAAGCGACACUUGCGCUUGACUUAUUAGAGAAAGACGCAUUACAGACUCAAGUAUGGGUGUCAUACAAUGAACUAUGGACUUCGAUGAUAAACCAGACCUUACUAUUGACUACUGAUGAAGACACUCCUGUUAUUAUCAUUGUUCCAUCUAAAUGCGUCGGGAUUGCGGGCGGUUGGCAUGAUUUCGGAGUAGCAGCCAUCGCUCGAUCUGAGUGGGGUGCAAAUGUUGUUCGUGAAAAACUCCUAACUGAUGAUAUAAAUAGGAAUAUGUUCAUACUUAACUGCAUUGACAGUGCGUUCGGCCUUGGAGCUGCCGUGUUUUCACACCCAAAUGGAAUAUUAGAUUGGUUGUGCGUGCGUCAGCAACUUUGGGCUGACUUCGUGAAACGACAAGUAGAGGAAGAAGACGGCCUCAGAGGCAGACAAAGAAUUAAAGACACGGAAAUUCGGAUAUAUCCAGAGCCAGGGCGACGCGUCUCCGCUGAGUAUGUGUCAACUCUAGUCACAUCCGCCGAACAGUAUCUCACUGCUCCUUCAGACGUAGUAACGAACUUAACUCGUCAGGCAGACCUGCUAUCACUCACUGAGCAUGGAGAGACAGAUGAGCAUUUAUCCACCGAUCAAGGUCCCGAAUAAGUGUGUGAUACAUGAUCAUCCAACUUUUCCUUUGUUUUUCCUUGCUUUGAGCUAAUCGUUAAUGUUUCAAAUUUUACUCGUGUAUUUUGCCAUGUCUCUCCGCGUUCCAUCAAAUGAUAAUGCAGCCGCUUUUGUUCUUCACCGUCUAUUUUUGAUAUUUACAUCUUGUUCGUCUUUCCUACUCCGAUUGUCUUUUCUUUCUUCUCUUUAGAAUUAUUUAAGAAAACUAUAUUUAUAUGUUAUACAUUAAAGACCUUAAAGUCGUGUUUUAUUUAUUUAAAUAAGUCUAUCCGGGAUUAUGUAGUAUGGACACCUCAGUUCGCAUUUUAAAUUUAAACUAUUCUAAUCGUCAACUUAAAUGUAUUUCAGUACAUUAGCAACCCGUGGGUCGUUCGCAAUUAUGCUUAGAUAGGAUGCUUAAAUUCGCGGAGCUAAGG  >Ss-AA_clean.1_(paired)_contig_1114  UAAGCGGAGAAUACACAGCUCUCGCAUCUAGCUUUUUAGCAGCAGGAGAGCAUUUCCCGAAUCCGUUACUUGCGUUCUCAGUUUCAUUAUGGUUUGCUCCCCGGUGGCUAAGAAGAAGAAUGAUAUUGGACUCUAUACCGUUGCCGACUCAAGAUGUAAGACGACUAGUAAGCAUAUUACCGUCUGCGGUCGGAGGGUUGGGGUUCAACUCUAUUGCUGAUUAUAUGUGUAGAGGUGCAAGUGAUCCGCUAUCCAAUGCGUUUGCGGGACUCAAAAUGCUCUCAGGGAUACCAUUGGUCAAACAGUACGCUGGUUAUCUAGAAACUAGGUUGCCUUACUCAGAACAGCCGAAUCUUAUGAGCUUGAUUCAAGACCCAUUCUCUCUUCCUUUUCGGAAACCAACAUCUGCAGUAAAUGUUUCUGCUUCUGCCAAGCGAGACAUUGUCAAAGAGCUCUCGAGGUCUAAUAAGCACGUUCACUCCUUGUUUACUAUAGCUGACGCUAAAUAUACUAAAAGCUUUGAAGAAACCUUAAUUAGUUCAAAUCCUUUUUAUGUCUUACCCUUACAUGAUAUACUCAAGUCCUCCGCUAUAGGAAUAAGGAAAGAAUUUGAAACUAUGUUUACUAGCACUCGGACUAUGCAAGACUUAGCCUCUAAAGAUAGGAGAGUUGACUUUGGAGGUAACAUCAUUCAACAAGAUGAAAAGACAGUCAACUACGCUAUCAGUCUGGUAGAAGAUGCGAAACAUAGAAAACUACGGACCAAAUUUAACUCGAUCUUCGAGACAGUGAGUAACCUCAGAAAGUAUUGGUUCAAAGAUGGUCGAACUGUGGAAGGAAUCACUACUCUACACCCGUUCGACAGCAAAAUCGAGUACACUAAUAAGCUACCCGGUAUAACUGCCUUAUUUCUGUCUGAUCCAAAAGACAGUGUGACGACUAGAGGUAAAAUUGAUCCUUACAUAGGAGCGAGAACAAAAGAAAAACGAGGGGAUUACGGAAUUAAGACAGUUACCAAGGAUCCAGCCAGCAAAGCUGCUAAGAAAAUGGCUCUAUUAUGGUCUCAAUUGCCUGGAGAUGAUAACUGGAGGUCCCUUAUCAAAAGUGUAGUAGAAACUAGAGUAGGGAUGAAAUUCGAAGAAGUCGAGAAAAUAAUGCCACAUGUGUUCGGUGGAAUUUCUGCACAUAGGUAUGAUGCAAUGGAAAGCAGGAAGAAAUU  >Ss-AA_clean.1_(paired)_contig_6890  UUGAAUCUUGCUGCGAUCUCUUUGAGCACCAAACUCUUUUAGAAUACGUAACCACAGUUGUUGAUACAAUGUUUUCUUCCGGCUUUACUAUAUGGUUGAUUCUGGCGAAAGUGUCCUCUAAUCGCUUCUCGACUUUCGAUAUCAAAGAUUUUUGGAGGUCGAUGGUAGGAUAAUCGGAUUUCUUAAAGUAGAGACUCAACACUUGAUUGUCUCCUUGACCAGUUAAUUGGAAUUCAACAUCUAAGCCAUUUAGAGAAUUUUCCAUUGCUGCGAUAGUAACAAUUGUCCAUUGCUUUUGAAUAAUACCUUCAAAACCACCAAUAUGUGGUUUGUCUGAACCCCACGCAAGAUCGCUAUCAGGUAGAGUGUGAACUCCUGAAGUCAUCCCUUUAGGAGGACAAUCAGCCACACGAACUAGGAUUUGGGAACUAGAAAAAAAUCUGUGAGCAACUGUGUACGCAUCCAAGCCAAAUGUCUGAUUGAGGAUCACUCCAACUCUAUCUACGACAAUUCCUCUCA  >Ss-AA_clean.1_(paired)_contig_2147  TATACAATTCCTTAACAGACAAATGTGGCTGACAACCCAGCCACTCCTTAAAAGAATGAAAAAACTTAATACCACCCCGTATATCGTCGAATACAGCATATTCCACGTCAGGTGCTTTTAAACACTCGGTACCACTCACTAGACCAACACAGTATATATGTTTTCCAAGGCTTCTGGCCCAAGAAGTCTTTCCTGTTTGTGAAGGTCCGTACAGGACCAAGCTCTTTACTCTTCCTCGTCTCTCCTCACTAGAGCCGATTCCAGACTGTTCCAACCACCGATTTCTCCCGTCAAGGTCACCTCCAATAAAATGAGCTGCTGCTGGTGACTCATACTCGGGAGCATGGTACTGATACTTCCAGTCGCAGTATCTCTGAAGTTGGGAAAAAGAAGTTGCTGCAGCCTTTGGATCCAACTCGTGCACCAATTCCCAAAACTCATCTCTATTCGTCGCTGACGTAATGATAGCCCACTTAGAATCAGACGGCCGAGTTCCCACTCTGCCAACGCTTGGCCTTCCAAGGCCCCCACAAATGACATCTCCATCTTTGATCGCGTAGTCATACCCCTTCTCCGGAGTCCCTCGAGACTTTGTAATGTTTGGGTGGTGACCATCGACATCGAATACATCAGCCTTTCTACTGCGAAACTTCCGTCCGAACTCTGCAAAGCAGTGTAGGTGAATGCCTCCATCCTCGTGATGCTCTCTTCCAATGATGCACTCAGCTCCCAGAAGTGAAAGTTTATCCATAACTCGGAAAGCATCGAGCTCTCCACATTGTGCGTATGTGAGCAAGACATATTTAGCGTGGAAGTCGAAAGTCATGTGATAAATTGTTGTCCCTTTGAGTCTCTGGGCTAAACTAATATTATAGCCCAGGGACGGGGACACTTCCAACTATAAATACCCCGTCACTTCCCACGCAACGGACAATCAAACATGTCCGCATCATCACAAGATGGCATACGCACGTTATCGCTCTTCCAGGTTTCGGGCAAGAAGGCCAATCAAGACTTCCAGAAGAGGCGGTGTGTCAAAGCCAAGACGCCGATCTTACAGCGCGAAGAAGCGAACCTATCGCAAAAAAGCCTCAATGTCAAAGCGGAGCATCCTCAACACGACATCCCGAAAAAAACGTAATGGAATGCTTACGTTCTCCAACACTACCAATAGTGGGGCTAGTACAGCTCCAGCCGCTGCTCCCGTTTACGTAAATGCAGTTCTTGGCGGCACATTTATTTGGTGCCCTACTGCAATGAACCTTGTCGCGCAGTCTCTTATCTCCAATATGTCTTCTCGCACGGCTACGACGTGTTATATGAGAGGGTTGTCTGAACACATTCGAATTCAGACTAGTTCTGGTATACCCTGGTTTCATCGCAGGGTATGTTTTACTTGGAAAGGTTTAGGGCCTTTCAACGTTATGGCAACAGAGTCTCCAACTTCTGATTTCCCTTGGTTACCTTATCAGGACTCCAGTAATGGAGTTCAACGTCTCTTTTTTAATGAGAACGTCAACAACATGCCTCAGACAATCAACCGCCAACGTAACATTTTGTTTAAGGGGGCAGAGAATGTTGATTGGACCGATCCTATTATTGCCCCTCTTGATACCGGTCGTATTACAGTCAAGTTTGATAAGACTTGGACTATGCAATCTGGCAATACTAATGGGATTGTTCGGGAACGGAAATTGTGGCATCCAATGAACCACAATCTCGTGTACGATGACGACGAATACGGAGATATTGAATCAGGCAGCTGGTTCAGTACTGATAGCAAGGCAGGTATGGGAGATTATTATGTAATGGATATTATACAAGGCGGTCAGGGAGGTACGGCGTCCGACCTTCTTCGTATGACCGCTAACTCTACTATGTATTGGCATGAAAAGTAGGAGCAAGGCAGATTTGTACACAAGGGCGTGTCTCTGCACCATGTGGGGAGGGGCCCCCTGGGGGGGACCCACGTGGGGCTAGACCGACCAACCCTGCCCGGCGCAGCCGATATGGATAATTAATTGAATTCTACTACTGGCTCTGTAATCTCAATGAAAGTGCAATTCATCTCCATCCAATCAATGTCCACTTGTAACAACTCGTTTCGAGGATCAGCGTTTGAACACCAAATGCTCGGTTTACCCCACTCAATAACCTTAGGTTCTC |
| --- |
