# Supplementary material for: Virome Characterization of a Collection of S. sclerotiorum from Australia
Source: Front Microbiol. 2018 Jan 11;8:2540. doi: 10.3389/fmicb.2017.02540 (PMC5768646; doi:10.3389/fmicb.2017.02540)
Supplement: Supplementary file 3 [file Table3.DOC]

**Table S3**.Primer pairs used to confirm viral sequences in strains of *Sclerotinia sclerotiorum*

| **Name** | **Contig number** | **Primer name** | **Primer sequence** | **Primer length** | **Amplicon size (bp)** |
| --- | --- | --- | --- | --- | --- |
| SsPV2 | contig_661 | F | GCCGAGAGTGTCAGACGAGAAT | 22 | 494 |
|  |  | R | AGAAGAAGCGTCCTGGCAAGAT | 22 |  |
| SsPV3 | contig_904 | F | GTCGGACACTCGTAGCAGGAT | 21 | 669 |
|  |  | R | GCACGGCTTGGACTTCTCAG | 20 |  |
| SsVV1 | contig_9550 | F | GGTTGAGTGGTGCGAGTTGAAG | 22 | 544 |
|  |  | R | GTGTGACCCTGACCTTGTTGC | 21 |  |
| SsBRV3 | contig_823 | F | TTGATGGCGAGATTGCTGTTCC | 22 | 467 |
|  |  | R | AGACGAGGAGACATTGCGAGAA | 22 |  |
| SsAftRV1 | contig_6913 | F | CAAGCGTTCTGCCATTGAGGAT | 22 | 453 |
|  |  | R | ATGCCAGCCGTGCGGTATT | 19 |  |
| SsEV7 | contig_14516 | F | TTGCCTCTAGCCTGTCTTCCAT | 22 | 462 |
|  |  | R | ACTTGTCTGGTGGCGCTATGA | 21 |  |
| SsEV1 | contig_7442 | F | ATCACCATCGCACCTGTCACTA | 21 | 469 |
|  |  | R | CACGAACTGCCAGACCTCCTT | 21 |  |
| SsEV2 | contig_3829 | F | CGCACCACAACCAACCACAG | 20 | 395 |
|  |  | R | GCACCGAGCAACTTAGCACATT | 22 |  |
| SsEV3 | contig_1364 | F | TGAACACCTGGAGGCACAACA | 21 | 435 |
|  |  | R | GTCCGTCGGTCTGTCTGTCTT | 21 |  |
| SsEV4 | contig_282 | F | GGCATTAGTTGTGTCGGCGTTA | 22 | 478 |
|  |  | R | TGGTCATGTCAGCGGTGGATT | 21 |  |
| SsEV5 | contig_6278 | F | ATTGCGGTGGTGTGGTGGTA | 20 | 473 |
|  |  | R | GTCATGTCAGTTGAGAGAGCCTTG | 24 |  |
| SsEV5 | contig_15215 | F | GTTGTCTTGGTGTGGGCTTGG | 21 | 273 |
|  |  | R | GCTCGGAGATGTGTCCTTGTTG | 21 |  |
| SsHV3 | contig_1373 | F | GACACGCACCTGCACCTTATTC | 22 | 401 |
|  |  | R | CCGCTGCTGTAGACGCCATA | 20 |  |
| SsHV1 | contig_259 | F | TGTTCCGTTGCCCTCATTCGTA | 22 | 353 |
|  |  | R | GCGTAGACACAAGTTGCCTCAG | 22 |  |
| SsHV4 | contig_1333 | F | ACACGGACTGGGTAACGCTTT | 21 | 732 |
|  |  | R | ACGGCTAAGTGGTGGAGGAATT | 22 |  |
| SsOLV-1 | contig_58 | F | CACGGATGTCTTGGACTGCTC | 21 | 149 |
|  |  | R | GCTGGGCGATGAAGGATATGG | 21 |  |
| SsOLV-3 | contig_30 | F | GTCGCTGATGTGTCGGTACAAG | 22 | 539 |
|  |  | R | GCCTGGTGGTGTGAGTGGAA | 20 |  |
| SsMV1 | contig_192 | F | GCAACTGAGATGTCCACACCAA | 22 | 348 |
|  |  | R | TGCCACTGACCGTTTCCCAATA | 22 |  |
| SsMV1/HC025 | contig_67 | F | CAGTATGGAACGCTCGGTGATC | 22 | 575 |
|  |  | R | GAGGAGGCAATCAGGTCTACCA | 22 |  |
| SsMV2 | contig_283 | F | AGCATCACCCGTCCCTGTTC | 20 | 547 |
|  |  | R | GGTCCGTAGCCGAGGTTAGATC | 22 |  |
| SsMV4 | contig_238 | F | TGCTTCAGACACCATTCCACCT | 22 | 604 |
|  |  | R | TTCCTGAGCCGCCTTTACGAT | 21 |  |
| SsMV5 | contig_188 | F | ATTCAAGGATCACCGAGCGTTC | 22 | 601 |
|  |  | R | TGAGATGTCCACACCGAGTCTT | 22 |  |
| SsMV6 | contig_308 | F | AACAAGAAAGCCGACGACAACA | 22 | 438 |
|  |  | R | GTGGAGGAGATAGGCGAGCAAT | 22 |  |
| SsMV32 | contig_1865 | F | GGCGTAATCTCCTCTCCATCGA | 22 | 592 |
|  |  | R | TGCGAGTGGTAGGTGTAGTTGA | 22 |  |
| SsMV8 | contig_42 | F | CTGGTGTATGATGGCTCTCCGA | 22 | 129 |
|  |  | R | GCAAACAGCCCGTCCGAAAG | 20 |  |
| SsMV7 | contig_69 | F | GCGGCTTCCTGGACAATCAC | 20 | 633 |
|  |  | R | GAAGAAGGCGGCGTGAGGAT | 20 |  |
| SsMV9 | contig_202 | F | ACTGCGAGGTGTTGTCCATCA | 21 | 497 |
|  |  | R | TCCGTGAACAGGTAGCAAGCT | 21 |  |
| SsMV10 | contig_226 | F | TTATGGCGGTCGAGAAGGAAGG | 22 | 477 |
|  |  | R | TCAGAGCAAGGATAGGGAGGGA | 22 |  |
| SsMV11 | contig_140 | F | CCTGACGGGAGAACATCGGAAA | 22 | 290 |
|  |  | R | CGGTTCGTAAGAGCCAGTTGGT | 22 |  |
| SsMV12 | contig_30 |  | GCATCCGCAGACGAAGAACAC | 21 | 705 |
|  |  |  | TGTGCTGAAGCCTGAGACCTC | 21 |  |
| SsMV14 | contig_126 | F | CAAGAGTCTCAGGCGGTCCAA | 21 | 434 |
|  |  | R | TGTTGGCGGTGAAGTCCTCTC | 21 |  |
| SsMV17 | contig_73 | F | CGACGGAGGAGGTTACCAGTT | 21 | 451 |
|  |  | R | TGCCATTACAGGTCAGACTCGG | 22 |  |
| SsMV33 | contig_90 | F | GGTCGTTCAGGTCGCATTAAGT | 22 | 500 |
|  |  | R | GGTAGTGCTCCGTCTGGTATCC | 22 |  |
| SsMV19 | contig_569 | F | AGCCGAGATGGAGCAAGAGAC | 21 | 387 |
|  |  | R | CTCAGAGCAAGGACAGGTAGGG | 22 |  |
| SsMV26 | contig_634 | F | CTCTTGGTGCTCTGCTTCATGG | 22 | 484 |
|  |  | R | TTACTTCAGCCACCGACCGATT | 22 |  |
| SsMV27 | contig_958 | F | ACCGCTAGGAACCTGCTCTG | 20 | 491 |
|  |  | R | CCTTACACGAATGCCGACTGAC | 22 |  |
| SsMV28 | contig_867 | F | TCGGCTGCAACTGCGTGAT | 19 | 391 |
|  |  | R | TGCGTCGGTCGGTTAGTAAGG | 21 |  |
| SsMV29 | contig_536 | F | CCACTGCTAACCGTCCGAAGA | 22 | 516 |
|  |  | R | ATGATCTGTCCGCTGCTACTGA | 22 |  |
| SsMV30 | contig_59 | F | GCCATCACCGACTGCGTTAC | 20 | 503 |
|  |  | R | GGTTACCTCCGTCTGCTTATGC | 22 |  |
| SsTLRV2 | contig_3545 | F | ATACGCCTCCCTCAACCTTTCC | 22 | 537 |
|  |  | R | CCGACCGATTCCGAAGACAGT | 21 |  |
| SsTLRV3 | contig_10849 | F | TCGTTGTTGGCAAGCATCTGTT | 22 | 378 |
|  |  | R | CAGGTTGGAAGAGGGAGCAAAC | 22 |  |
| SsTLRV4 | contig_11935 | F | GTCGTTGGTCGTGCTTCTGATG | 22 | 538 |
|  |  | R | GCCGAAGGATGACGCTCTGA | 20 |  |
| SsTLRV5 | contig_14854 | F | GGTGTCAGAGCAGCGAAGAATG | 22 | 558 |
|  |  | R | ATCAACGCCTACGACTCCAAGA | 22 |  |
| SsTLRV6 | contig_9241 | F | ATAGCCACTTCGTCGCAACCA | 21 | 591 |
|  |  | R | CAATGCCATTCTTCGCCACTCT | 22 |  |
| SsMV24 | contig_24 | F | TTCCTCTGTATCTCCTGGTGGC | 22 | 176 |
|  |  | R | CGGCTGACCTGCTAGAAATCTG | 22 |  |
| SsMV25 | contig_15 | F | ACAGTGTTGGACAGCCGATGG | 21 | 648 |
|  |  | R | CCGATTCCTCGACAGCCTCTAC | 22 |  |
| SsMV31 | contig_31 | F | ACCCACAACCTTCACCTTCCC | 21 | 419 |
|  |  | R | CGCCTCCGTACAGTAAGTCCTT | 22 |  |
| SsMV8 | contig_43 | F | CGACCGATCCGTTGGTTGTTC | 21 | 300 |
|  |  | R | GCCATCACAGACTGCGTTACC | 21 |  |
| SsULV2 | contig_224 | F | CCTGACGGACCTCCTTAGCAAT | 22 | 369 |
|  |  | R | GACGACGAGACGGACTTGGT | 20 |  |
| SsULV3 | contig_534 | F | GATGAGGAGAGGCAGGTCGTAG | 22 | 341 |
|  |  | R | TGGCTGGTGTTGTCGTGCTA | 22 |  |
| SsNSRV6 | contig_89 | F | TGGGAAAGGCTCGGTCAATGT | 21 | 300 |
|  |  | R | AAGAAGGAGTCGTGCGTGTC | 21 |  |
| SsNSRV7 | contig_579 | F | TTCCGCCTGCCGTCTTAGTG | 20 | 366 |
|  |  | R | CCGCTCTCGCTCCTGATTGT | 20 |  |
| SsNSRV8 | contig_1114 | F | CTTCTGCCAAGCGAGACATTGT | 22 | 416 |
|  |  | R | TCCTTCCACAGTTCGACCATCT | 22 |  |
| SsNSRV1 | contig_398 | F | ATCTCCTTGTCCCGCCATTTGA | 22 | 514 |
|  |  | R | GGTTCGCCTGTTCTTCGTGTT | 21 |  |
| SsNSRV2 | contig_100 | F | AGCATAGCGACACGAGCATCT | 21 | 487 |
|  |  | R | CACCGTCTCTTCTCCTCAACCA | 22 |  |
| SsNSRV3 | contig_557 | F | CCGAGTCCAAGGCATCATAGGT | 22 | 418 |
|  |  | R | GGTAGAACGGAGATGTGAGCGA | 22 |  |
| SsNSRV4 | contig_512 | F | TCTTGCGTGTCTCTGGCTTGT | 21 | 318 |
|  |  | R | TCCTTGCTCTTGACGGTTGTGA | 22 |  |
| SsNSRV5 | contig_4241 | F | GGTCAACAGCAGATGGCAACAA | 22 | 675 |
|  |  | R | CCTCCAGCTCCAACACCTTCA | 21 |  |
| SsHADV1 | contig_2147 | F | GCAGTTCTTGGCGGCACATT | 20 | 332 |
|  |  | R | CGTTGGCGGTTGATTGTCTGAG | 22 |  |
