# Supplementary material for: Virome Characterization of a Collection of S. sclerotiorum from Australia
Source: Front Microbiol. 2018 Jan 11;8:2540. doi: 10.3389/fmicb.2017.02540 (PMC5768646; doi:10.3389/fmicb.2017.02540)
Supplement: Supplementary file 4 [file Table4.DOC]

**Table S4.** Viruses selected for phylogenetic analysis in this study

| **Family** | **Virus name** | **RdRP Accession number** | **Reference** |
| --- | --- | --- | --- |
| *Partitiviridae* | Verticillium dahliae partitivirus 1 | YP_009164038.1 | (Cañizares et al., 2015) |
|  | Botryotinia fuckeliana partitivirus 1 | YP_001686789.1 | (De Guido et al., 2005) |
|  | Penicillium stoloniferum virus S | AAN86834.2 | (Kim et al., 2003) |
|  | Pseudogymnoascus destructans partitivirus-pa | YP_009259752.1 | (Ren et al., 2016) |
|  | Mycovirus FusoV | BAA09520.1 | (Nomura et al., 2003) |
|  | Verticillium albo-atrum partitivirus-1 | AIE47664.1 | (Cañizares et al., 2014) |
|  | Helicobasidium mompa dsRNA mycovirus | BAC23065.1 | (Osaki et al., 2002) |
|  | Sclerotinia sclerotiorum partitivirus S | YP_003082248.1 | (Liu et al., 2012) |
|  | Cherry chlorotic rusty spot associated partitivirus | CAH03668.1 | (Covelli et al., 2004) |
|  | Beet cryptic virus 1 | YP_002308574.1 | (Szego et al., 2010) |
|  | White clover cryptic virus 1 | YP_086754.1 | (Boccardo and Candresse, 2005) |
|  | Heterobasidion partitivirus 2 | ADL66905.1 | (Vainio et al., 2011) |
|  | Atkinsonella hypoxylon partitivirus | NP_604475.1 | (Oh and Hillman, 1995) |
|  | Sclerotinia sclerotiorum partitivirus 1 | AFR78160.1 | (Xiao et al., 2014) |
|  | Fusarium graminearum dsRNA mycovirus-1 | YP_223920.2 | (Kwon et al., 2009) |
|  | Botryosphaeria dothidea virus 1 | AKE49495.1 | (Zhai et al., 2016) |
|  | Sclerotinia nivalis victorivirus 1 | YP_009259368.1 | (Wu et al., 2016) |
| *Totiviridae* | Sclerotinia nivalis victorivirus 1 | YP_009259368.1 | (Wu et al., 2016) |
|  | Ustilaginoidea virens RNA virus 1 | AGI61065.1 | (Zhong et al., 2014) |
|  | Sphaeropsis sapinea RNA virus 1 | NP_047558.1 | (Preisig et al., 1998) |
|  | Helicobasidium mompa totivirus 1-17 | NP_898833.1 | (Nomura et al., 2003) |
|  | Rosellinia necatrix victorivirus 1 | YP_008130308.1 | (Chiba et al., 2013) |
|  | Helminthosporium victoriae virus 190S | NP_619670.2 | (Huang and Ghabrial, 1996) |
|  | Coniothyrium minitans RNA virus | ALM62231.1 | (Marzano and Domier, 2016) |
|  | Gremmeniella abietina RNA virus L1 | NP_624332.2 | (Tuomivirta and Hantula, 2003) |
|  | Leishmania RNA virus 2 | AHK06414.1 | Direct Submission |
|  | Trichomonas vaginalis virus 1 | AAA62868.1 | (Tai and Ip, 1995) |
|  | Saccharomyces cerevisiae virus L-BC (La) | NP_042581.1 | (Park et al., 1996) |
|  | Saccharomyces cerevisiae virus L-A | NP_620495.1 | (Icho and Wickner, 1989) |
|  | Sclerotinia sclerotiorum endornavirus 1 | AJF94392.1 | (Lee Marzano et al., 2016) |
| Unclassified | Cladosporium cladosporioides virus 1 | YP_009052470.1 | Unpublished |
|  | Alternaria tenuissima virus | AJP08049.1 | Unpublished |
|  | Aspergillus fumigatus tetramycovirus-1 | CDP74618.1 | (Kanhayuwa et al., 2015) |
|  | Beauveria bassiana polymycovirus 1 | CUS18595.1 | (Kotta-Loizou and Coutts, 2017) |
|  | Pepper cryptic virus 1 | AEJ07890.1 | (Sabanadzovic and Valverde, 2011) |
|  | Penicillium chrysogenum virus | YP_392482.1 | (Castón et al., 2003) |
|  | Sclerotinia nivalis victorivirus 1 | YP_009259368.1 | (Wu et al., 2016) |
|  | Rosellinia necatrix victorivirus 1 | BAM36400.1 | (Yaegashi et al., 2013) |
|  | Sclerotinia sclerotiorum botybirnavirus 2 | AMT92140.1 | (Ran et al., 2016) |
|  | Sclerotinia sclerotiorum botybirnavirus 1 | YP_009141012.1 | (Liu et al., 2015) |
|  | Botry porri botybirnavirus | YP_006390637.1 | (Wu et al., 2012) |
|  | Sclerotinia sclerotiorum magabirnavirus 1 | AKJ87317.1 | (Wang et al., 2015) |
|  | Bipolaris maydis chrysovirus | ARM36038.1 | Unpublished |
| *Endornaviridae* and *Hypoviridae* | Vicia faba endornavirus | YP_438201.1 | (Pfeiffer, 1998) |
|  | Rosellinia necatrix endornavirus 1 | BAT32944.1 | (Yaegashi and Kanematsu, 2015) |
|  | Sclerotinia sclerotiorum endornavirus 2 | YP_009022070.1 | (Khalifa and Pearson, 2014a) |
|  | Sclerotinia sclerotiorum endornavirus 1 | AJF94392.1 | (Lee Marzano et al., 2016) |
|  | Cryphonectria hypovirus 1 | ADO15048.1 | (Shapira et al., 1991) |
|  | Cryphonectria hypovirus2 | NP_613266.1 | (Hillman et al., 1994) |
|  | Cryphonectria hypovirus 3 | AAF13604.1 | (Smart et al., 1999) |
|  | Cryphonectria hypovirus 4 | YP_138519.1 | (Linder-Basso et al., 2005) |
|  | Phomopsis longicolla hypovirus\| YP_009051683.1 | YP_009051683.1 | (Koloniuk et al., 2014) |
|  | Fusarium graminearum hypovirus 1 | YP_009011065.1 | (Wang et al., 2013) |
|  | Sclerotinia sclerotiorum hypovirus 1 | AEL99352.1 | (Xie et al., 2011) |
|  | Sclerotinia sclerotiorum hypovirus 2 | AIA61616.1 | (Hu et al., 2014) |
|  | Valsa ceratosperma hypovirus 1 | BAM08994.1 | (Yaegashi et al., 2012) |
| Mitovirus | Slerotinia sclerotiorum mitovirus 1 | AEX91878.1 | (Xie and Ghabrial, 2012) |
|  | Sclerotinia sclerotiorum mitovirus 1 HC025 | AHX72146.1 | (Xu et al., 2015b) |
|  | Sclerotinia sclerotiorum mitovirus 5 | AHX84130.1 | (Khalifa and Pearson, 2014b) |
|  | Sclerotinia sclerotiorum mitovirus 4 | AMT92141.1 | (Ran et al., 2016) |
|  | Ophiostoma mitovirus 6 | NP_660181.1 | (Hong et al., 1999) |
|  | Sclerotinia sclerotiorum mitovirus 8 | AHF48624.1 | (Lee Marzano et al., 2016) |
|  | Sclerotinia sclerotiorum mitovirus 17 | ALD89134.1 | (Lee Marzano et al., 2016) |
|  | Sclerotinia sclerotiorum mitovirus 2 | AHX84129.1 | (Khalifa and Pearson, 2014b) |
|  | Rhizoctonia solani mitovirus 10 | ALD89102.1 | (Lee Marzano et al., 2016) |
|  | Sclerotinia sclerotiorum mitovirus 6 | AHX84134.1 | (Khalifa and Pearson, 2014b) |
|  | Sclerotinia sclerotiorum mitovirus 15 | AHF48631.1 | (Lee Marzano et al., 2016) |
|  | Sclerotinia sclerotiorum mitovirus 9 | AHF48625.1 | (Lee Marzano et al., 2016) |
|  | Sclerotinia sclerotiorum mitovirus 16 | AHF48632.1 | (Lee Marzano et al., 2016) |
|  | Sclerotinia sclerotiorum mitovirus 10 | AHF48626.1 | (Lee Marzano et al., 2016) |
|  | Sclerotinia sclerotiorum mitovirus 19 | ALD89136.1 | (Lee Marzano et al., 2016) |
|  | Botrytis cinerea mitovirus 3 | YP_009182161.1 | Unpublished |
|  | Ophiostoma mitovirus 1a | CAJ32466.1 | (Doherty et al., 2006) |
|  | Sclerotinia sclerotiorum mitovirus 7 | AHX84135.1 | (Khalifa and Pearson, 2014b) |
|  | Sclerotinia sclerotiorum mitovirus 12 | AHF48628.1 | (Lee Marzano et al., 2016) |
|  | Sclerotinia sclerotiorum mitovirus 11 | AHF48627.1 | (Lee Marzano et al., 2016) |
|  | Ophiostoma mitovirus 3a | NP_660176.1 | (Hong et al., 1998) |
|  | Macrophomina phaseolina mitovirus 1 | ALD89100.1 | (Lee Marzano et al., 2016) |
|  | Sclerotinia sclerotiorum mitovirus 14 | AHF48630.1 | (Lee Marzano et al., 2016) |
|  | Botrytis cinerea mitovirus 1 | YP_002284334.2 | (Wu et al., 2010) |
|  | Clitocybe odora virus | YP_005352912.1 | (Heinze, 2012) |
|  | Fusarium coeruleum mitovirus 1 | YP_009126873.1 | (Osaki et al., 2015) |
|  | Gremmeniella abietina mitochondrial RNA virus S2 | YP_077184.1 | (Tuomivirta and Hantula, 2005) |
|  | Ophiostoma mitovirus 4 | NP_660179.1 | (Hong et al., 1999) |
|  | Ophiostoma mitovirus 5 | NP_660180.1 | (Hong et al., 1999) |
|  | Thielaviopsis basicola mitovirus | YP_002822229.1 | (Park et al., 2006) |
|  | Tuber aestivum mitovirus | YP_004564622.1 | Direct Submission |
|  | Saccharomyces 20S RNA narnavirus | NP_660178.1 | (Rodriguezcousiño et al., 1991) |
|  | Saccharomyces 23S RNA narnavirus | NP_660177.1 | (Esteban et al., 1992) |
| *Ourmiavirus* and *Tombusviridae* | Tobacco bushy top virus | CUC08880.1 | Unpublished |
|  | Groundnut rosette virus | YP_009162058.1 | (Taliansky et al., 1996) |
|  | Carrot mottle virus | ACJ03580.2 | (Menzel et al., 2008) |
|  | Carrot mottle mimic virus | NP_054007.4 | (Gibbs et al., 1996) |
|  | Pea enation mosaic virus-2 | AEM45994.1 | (Vemulapati et al., 2011) |
|  | Carnation mottle virus | ADA69469.1 | Unpublished |
|  | Panicum mosaic virus | NP_068342.1 | (Turina et al., 1998) |
|  | Maize chlorotic mottle virus | NP_619718.1 | (Scheets, 2016) |
|  | Sclerotinia sclerotiorum umbra-like virus 1 | YP_009253998.1 | (Lee Marzano et al., 2016) |
|  | Diaporthe ambigua RNA virus 1 | NP_037581.1 | (Preisig et al., 2000) |
|  | Magnaporthe oryzae RNA virus | YP_009115495.1 | (Ai et al., 2016) |
|  | Rhizoctonia solani ourmia-like virus 1 RNA 1 | ALD89131.1 | (Lee Marzano et al., 2016) |
|  | Sclerotinia sclerotiorum ourmia-like virus 1 RNA 1 | ALD89138.1 | (Lee Marzano et al., 2016) |
|  | Soybean leaf-associated ourmiavirus 1 | ALM62238.1 | (Marzano and Domier, 2016) |
|  | Soybean leaf-associated ourmiavirus 2 | ALM62250.1 | (Marzano and Domier, 2016) |
|  | Sclerotinia sclerotiorum ourmia-like virus 2 RNA 1 | ALD89139.1 | (Lee Marzano et al., 2016) |
|  | Botrytis ourmia-like virus | YP_009182165.1 | (Donaire et al., 2016) |
| -ssRNA virus | Sclerotinia sclerotiorum negative-stranded RNA virus 1 | YP_009094317.1 | (Liu, 2014) |
|  | Sclerotinia sclerotiorum negative-stranded RNA virus 3 | AJT39503.1 | (Lee Marzano et al., 2016) |
|  | Soybean-associated negative-stranded RNA virus 2 | ALM62227.1 | (Marzano and Domier, 2016) |
|  | Soybean-associated negative-stranded RNA virus 1 | ALM62220.1 | (Marzano and Domier, 2016) |
|  | Soybean-associated negative-stranded RNA virus 3 | ALM62228.1 | (Marzano and Domier, 2016) |
|  | Sclerotinia sclerotiorum negative-stranded RNA virus 2 | ALD89145.1 | (Lee Marzano et al., 2016) |
|  | Sclerotinia sclerotiorum negative-stranded RNA virus 4 | ALD89140.1 | (Lee Marzano et al., 2016) |
|  | Soybean-associated negative-stranded RNA virus 4 | ALM62229.1 | (Marzano and Domier, 2016) |
|  | Lettuce necrotic yellows virus | YP053236 | (Torok and Vetten, 2003) |
|  | Rabies virus | NP056797 | Unpublished |
|  | Zaire ebolavirus | NP_066251.1 | (Volchkov et al., 1999) |
|  | Marburg marburgviru | YP_001531159.1 | (Enterlein et al., 2006) |
|  | Nipah virus | NP_112028.1 | (Xu et al., 2015a) |
|  | Mumps virus | NP_054714.1 | (Takeuchi et al., 1988) |
|  | Newcastle disease virus | AFH08721.1 | (Takimoto et al., 2000) |
|  | Nyamanini nyavirus | YP002905337 | (Mihindukulasuriya et al., 2009) |
|  | Borna disease virus | P52639 | (Rudolph et al., 2003) |
|  | Rhizoctonia solani negative-stranded virus 2 | ALD89130 | (Lee Marzano et al., 2016) |
|  | Rhizoctonia solani negative-stranded virus 1 | ALD89129 | (Lee Marzano et al., 2016) |
|  | Citrus psorosis virus | YP_089661 | (Mahua and Bibha, 2006) |
|  | Lettuce ring necrosis virus | YP_053236 | (Torok and Vetten, 2003) |

**References which are not addressed in the main text**

Ai YP, Zhong J, Chen CY, Zhu HJ, Gao BD. (2016). A novel single-stranded RNA virus isolated from the rice-pathogenic fungus *Magnaporthe oryzae* with similarity to members of the family Tombusviridae. *Archives of Virology* 161**:** 725-729.

Boccardo G, Candresse T. (2005). Complete sequence of the RNA2 of an isolate of White clover cryptic virus 1 , type species of the genus *Alphacryptovirus*. *Archives of Virology* 150**:** 399-402.

Cañizares MC, PérezArtés E, GarcíaPedrajas NE, GarcíaPedrajas MD. (2015). Characterization of a new partitivirus strain in *Verticillium dahliae* provides further evidence of the spread of the highly virulent defoliating pathotype through new introductions. *Phytopathologia Mediterranea* 54**:** 516-523.

Jiang D, Ghabrial SA. (2004). Molecular characterization of Penicillium chrysogenum virus: reconsideration of the taxonomy of the genus Chrysovirus. Journal of General Virology 85: 2111-2121.

Chiba S, Lin YH, Kondo H, Kanematsu S, Suzuki N. (2013). A novel victorivirus from a phytopathogenic fungus, Rosellinia necatrix, is infectious as particles and targeted by RNA silencing. Journal of virology 87: 6727.

Covelli L, Coutts RH, Di SF, Citir A, Açikgöz S, Hernández C et al. (2004). Cherry chlorotic rusty spot and Amasya cherry diseases are associated with a complex pattern of mycoviral-like double-stranded RNAs. I. Characterization of a new species in the genus Chrysovirus. Journal of General Virology 85: 3389-3397.

De Guido M, Minafra A, Santomauro A, Pollastro S, De Miccolis Angelini R, Faretra F. (2005). Molecular characterization of mycoviruses from Botryotinia fuckeliana. Journal of Plant Pathology 87: 293.

Doherty M, Coutts RH, Brasier CM, Buck KW. (2006). Sequence of RNA-dependent RNA polymerase genes provides evidence for three more distinct mitoviruses in Ophiostoma novo-ulmi isolate Ld. Virus Genes 33: 41-44.

Enterlein S, Volchkov V, Weik M, Kolesnikova L, Volchkova V, Klenk HD et al. (2006). Rescue of recombinant Marburg Virus from cDNA is dependent on nucleocapsid protein VP30. Journal of Virology 80: 1038-1043.

Esteban LM, Rodriguezcousiño N, Esteban R. (1992). T double-stranded RNA (dsRNA) sequence reveals that T and W dsRNAs form a new RNA family in Saccharomyces cerevisiae. Identification of 23 S RNA as the single-stranded form of T dsRNA. Journal of Biological Chemistry 267: 10874 -10881.

Gibbs MJ, Cooper JI, Waterhouse PM. (1996). The genome organization and affinities of an Australian isolate of Carrot mottle umbravirus. Virology 224: 310-313.

Hillman BI, Halpern BT, Brown MP. (1994). A viral dsRNA element of the chestnut blight fungus with a distinct genetic organization. Virology 201: 241-250.

Hong Y, Cole TE, Brasier CM, Buck KW. (1998). Evolutionary relationships among putative RNA-dependent RNA polymerases encoded by a mitochondrial virus-like RNA in the Dutch elm disease fungus, Ophiostoma novo-ulmi, by other viruses and virus-like RNAs and by the Arabidopsis mitochondrial genome. Virology 246: 158-169.

Hong Y, Dover SL, Cole TE, Brasier CM, Buck KW. (1999). Multiple mitochondrial viruses in an isolate of the Dutch Elm disease fungus Ophiostoma novo-ulmi. Virology 258: 118-127.

Huang S, Ghabrial SA. (1996). Organization and expression of the double-stranded RNA genome of Helminthosporium victoriae 190S virus, a totivirus infecting a plant pathogenic filamentous fungus. Proc Natl Acad Sci U S A 93: 12541-12546.

Icho T, Wickner RB. (1989). The double-stranded RNA genome of yeast virus L-A encodes its own putative RNA polymerase by fusing two open reading frames. Jouranl of Biological Chemistry 264: 6716-6723.

Kim JW, Shi YK, Kim KM (2003). Genome organization and expression of the Penicillium stoloniferum Virus S. Virus Genes 27: 249-256

Koloniuk I, Elhabbak MH, Petrzik K, Ghabrial SA. (2014). Complete genome sequence of a novel hypovirus infecting Phomopsis longicolla. Archives of Virology 159: 1861.

Li P, Lin Y, Zhang H, Wang S, Qiu D, Guo L. (2015). Molecular characterization of a novel mycovirus of the family Tymoviridae isolated from the plant pathogenic fungus Fusarium graminearum. Virology 489: 86-94.

Linder-Basso D, Dynek JN, Hillman BI. (2005). Genome analysis of Cryphonectria hypovirus 4, the most common hypovirus species in North America. Virology 337: 192-203.

Mahua S, Bibha K. (2006). Genetic variation of populations of Citrus psorosis virus. Journal of General Virology 87: 3097-310.

Menzel W, Maiss E, Vetten HJ. (2008). Complete nucleotide sequence of a carrot isolate of Carrot mottle virus from Germany. Archives of Virology 153: 2163.

Mihindukulasuriya KA, Nguyen NL, Wu GH, Da RA, Popov VL, Tesh RB et al. (2009). Nyamanini and midway viruses define a novel taxon of RNA viruses in the order Mononegavirales. Journal of Virology 83: 5109-5116.

Nomura K, Osaki H, Iwanami T, Matsumoto N, Ohtsu Y. (2003). Cloning and characterization of a totivirus double-stranded RNA from the plant pathogenic fungus, Helicobasidium mompa Tanaka. Virus Genes 26: 219-226.

Oh CS, Hillman BI. (1995). Genome organization of a partitivirus from the filamentous ascomycete Atkinsonella hypoxylon. Journal of General Virology 76 ( Pt 6): 1461-1470.

Osaki H, Nomura K, Iwanami T, Kanematsu S, Okabe I, Matsumoto N et al. (2002). Detection of a double-stranded RNA virus from a strain of the violet root rot fungus Helicobasidium mompa Tanaka 25: 139-145.

Osaki H, Nakamura H, Sasaki A, Matsumoto N, Yoshida K. (2006). An endornavirus from a hypovirulent strain of the violet root rot fungus, Helicobasidium mompa. Virus Research 118: 143-149.

Park CM, Lopinski JD, Masuda J, Tzeng TH, Bruenn JA. (1996). A second double-stranded RNA virus from yeast. Virology 216: 451-454.

Preisig O, Wingfield BD, Wingfield MJ. (1998). Coinfection of a fungal pathogen by two distinct double-stranded RNA Viruses. Virology 252: 399-406.

Preisig O, Moleleki N, Smit WA, Wingfield BD, Wingfield MJ. (2000). A novel RNA mycovirus in a hypovirulent isolate of the plant pathogen Diaporthe ambigua. Journal of General Virology 81: 3107-3114.

Ran H, Liu L, Li B, Cheng J, Fu Y, Jiang D et al. (2016). Co-infection of a hypovirulent isolate of Sclerotinia sclerotiorum with a new botybirnavirus and a strain of a mitovirus. Virol J 13: 92.

Ren P, Rajkumar SS, Sui H, Masters PS, Martinkova N, Kubatova A et al. (2016). Novel Partitivirus infection of bat white-nose syndrome (WNS) fungal pathogen Pseudogymnoascus destructans links eurasian and North American isolates. Biorixv doi: https://doi.org/10.1101/059709.

Rodriguezcousiño N, Esteban LM, Esteban R. (1991). Molecular cloning and characterization of W double-stranded RNA, a linear molecule present in Saccharomyces cerevisiae. Identification of its single-stranded RNA form as 20 S RNA. Journal of Biological Chemistry 266: 12772-12778.

Rudolph MG, Kraus I, Dickmanns A, Eickmann M, Garten W, Ficner R. (2003). Crystal structure of the borna disease virus nucleoprotein. Structure 11: 1219-1226.

Sabanadzovic S, Valverde RA. (2011). Properties and detection of two cryptoviruses from pepper ( Capsicum annuum ). Virus Genes 43: 307-312.

Scheets K. (2016). Analysis of gene functions in Maize chlorotic mottle virus. Virus Research 222: 71-79.

Shapira R, Choi GH, Nuss DL. (1991). Virus-like genetic organization and expression strategy for a double-stranded RNA genetic element associated with biological control of chestnut blight. EMBO Journal 10: 731-739.

Szego A, Enünlü N, Deshmukh SD, Veliceasa D, Hunyadigulyás E, Kühne T et al. (2010). The genome of Beet cryptic virus 1 shows high homology to certain cryptoviruses present in phylogenetically distant hosts. Virus Genes 40: 267-276.

Tai JH, Ip CF. (1995). The cDNA sequence of Trichomonas vaginalis virus-T1 double-stranded RNA. Virology 206: 773-776.

Takeuchi K, Hishiyama M, Yamada A, Sugiura A. (1988). Molecular cloning and sequence analysis of the mumps virus gene encoding the P protein: mumps virus P gene is monocistronic. Journal of General Virology 69: 2043-2049.

Takimoto T, Taylor GL, Crennell SJ, Scroggs RA, Portner A. (2000). Crystallization of Newcastle disease virus hemagglutinin-neuraminidase glycoprotein. Virology 270: 208-214.

Taliansky ME, Robinson DJ, Murant AF. (1996). Complete nucleotide sequence and organization of the RNA genome of groundnut rosette umbravirus 77: 2335-2345.

Torok V, Vetten H. (2003). Identification and molecular characterization of a new ophiovirus associated with lettuce ring necrosis disease. Proceedings of Arbeitskreis Viruskrankheiten der Pflanzen, Heidelberg, Germany.

Tuomivirta TT, Hantula J. (2003). Two unrelated double-stranded RNA molecule patterns in Gremmeniella abietina type A code for putative viruses of the families Totiviridae and Partitiviridae. Archives of Virology 148: 2293-2305.

Turina M, Maruoka M, Monis J, Jackson AO, Scholthof KBG. (1998). Nucleotide sequence and infectivity of a full-length cDNA clone of Panicum Mosaic Virus . 241: 141-155.

Vainio EJ, Keriö S, Hantula J. (2011). Description of a new putative virus infecting the conifer pathogenic fungus Heterobasidion parviporum with resemblance to Heterobasidion annosum P-type partitivirus. Archives of Virology 156: 79-86.

Vemulapati B, Druffel KL, Eigenbrode SD, Karasev A, Pappu HR. (2011). Genomic characterization of pea enation mosaic virus-2 from the Pacific Northwestern USA. Archives of Virology 156: 1897-1900.

Volchkov VE, Volchkova VA, Chepurnov AA, Blinov VM, Dolnik O, Netesov SV et al. (1999). Characterization of the L gene and 5' trailer region of Ebola virus. Journal of General Virology 80: 355-362.

Wang S, Kondo H, Liu L, Guo L, Qiu D. (2013). A novel virus in the family Hypoviridae from the plant pathogenic fungus Fusarium graminearum. Virus Research 174(1-2) : 69-77.

Xu K, Chan YP, Bradel-Tretheway B, Akyol-Ataman Z, Zhu Y, Dutta S et al. (2015a). Crystal structure of the pre-fusion Nipah virus fusion glycoprotein reveals a novel hexamer-of-trimers assembly. Plos Pathogens 11: e1005322.

Yaegashi H, Kanematsu S, Ito T. (2012). Molecular characterization of a new hypovirus infecting a phytopathogenic fungus, Valsa ceratosperma. Virus Research 165: 143-150.

Yaegashi H, Nakamura H, Sawahata T, Sasaki A, Iwanami Y, Ito T et al. (2013). Appearance of mycovirus-like double-stranded RNAs in the white root rot fungus, Rosellinia necatrix, in an apple orchard. Fems Microbiology Ecology 83: 49-62.

Yaegashi H, Kanematsu S. (2015). Natural infection of the soil-borne fungus Rosellinia necatrix with novel mycoviruses under greenhouse conditions. Virus Research 219: 83-91.

Zhong J, Lei XH, Zhu JZ, Song G, Zhang YD, Chen Y et al. (2014). Detection and sequence analysis of two novel co-infecting double-strand RNA mycoviruses in Ustilaginoidea virens. Archives of Virology 159: 3063-3070.

Ai, Y.P., Zhong, J., Chen, C.Y., Zhu, H.J., and Gao, B.D. (2016). A novel single-stranded RNA virus isolated from the rice-pathogenic fungus Magnaporthe oryzae with similarity to members of the family Tombusviridae. *Archives of Virology* 161(3)**,** 725-729.

Boccardo, G., and Candresse, T. (2005). Complete sequence of the RNA2 of an isolate of White clover cryptic virus 1 , type species of the genus Alphacryptovirus. *Archives of Virology* 150(2)**,** 399-402.

Cañizares, M.C., Pérezartés, E., and Garcíapedrajas, M.D. (2014). The complete nucleotide sequence of a novel partitivirus isolated from the plant pathogenic fungus *Verticillium albo-atrum*. *Archives of Virology* 159(11)**,** 3141-3144.

Cañizares, M.C., PérezArtés, E., GarcíaPedrajas, N.E., and GarcíaPedrajas, M.D. (2015). Characterization of a new partitivirus strain in Verticillium dahliae provides further evidence of the spread of the highly virulent defoliating pathotype through new introductions. *Phytopathologia Mediterranea* 54(3)**,** 516-523.

Castón, J.R., Ghabrial, S.A., Jiang, D., Rivas, G., Alfonso, C., Roca, R., et al. (2003). Three-dimensional structure of penicillium chrysogenum virus: a double-stranded RNA virus with a genuine T=1 capsid. *Journal of Molecular Biology* 331(2)**,** 417-431.

Chiba, S., Lin, Y.H., Kondo, H., Kanematsu, S., and Suzuki, N. (2013). A novel victorivirus from a phytopathogenic fungus, Rosellinia necatrix, is infectious as particles and targeted by RNA silencing. *Journal of Virology* 87(12)**,** 6727.

Covelli, L., Coutts, R.H., Di, S.F., Citir, A., Açikgöz, S., Hernández, C., et al. (2004). Cherry chlorotic rusty spot and Amasya cherry diseases are associated with a complex pattern of mycoviral-like double-stranded RNAs. I. Characterization of a new species in the genus Chrysovirus. *Journal of General Virology* 85(Pt 11)**,** 3389-3397.

De Guido, M., Minafra, A., Santomauro, A., Pollastro, S., De Miccolis Angelini, R., and Faretra, F. (2005). Molecular characterization of mycoviruses from Botryotinia fuckeliana. *Journal of Plant Pathology* 87(4)**,** 293.

Doherty, M., Coutts, R.H., Brasier, C.M., and Buck, K.W. (2006). Sequence of RNA-dependent RNA polymerase genes provides evidence for three more distinct mitoviruses in Ophiostoma novo-ulmi isolate Ld. *Virus Genes* 33(1)**,** 41-44.

Donaire, L., Rozas, J., and Ayllon, M.A. (2016). Molecular characterization of Botrytis ourmia-like virus, a mycovirus close to the plant pathogenic genus *Ourmiavirus*. *Virology* 489**,** 158-164. doi: 10.1016/j.virol.2015.11.027.

Enterlein, S., Volchkov, V., Weik, M., Kolesnikova, L., Volchkova, V., Klenk, H.D., et al. (2006). Rescue of Recombinant Marburg Virus from cDNA Is Dependent on Nucleocapsid Protein VP30. *Journal of Virology* 80(2)**,** 1038-1043.

Esteban, L.M., Rodriguezcousiño, N., and Esteban, R. (1992). T double-stranded RNA (dsRNA) sequence reveals that T and W dsRNAs form a new RNA family in Saccharomyces cerevisiae. Identification of 23 S RNA as the single-stranded form of T dsRNA. *Journal of Biological Chemistry* 267(15)**,** 10874.

Gibbs, M.J., Cooper, J.I., and Waterhouse, P.M. (1996). The Genome Organization and Affinities of an Australian Isolate of Carrot Mottle Umbravirus. *Virology* 224(1)**,** 310-313.

Heinze, C. (2012). A novel mycovirus from Clitocybe odora. *Archives of Virology* 157(9)**,** 1831-1834.

Hillman, B.I., Halpern, B.T., and Brown, M.P. (1994). A viral dsRNA element of the chestnut blight fungus with a distinct genetic organization. *Virology* 201(2)**,** 241-250.

Hong, Y., Cole, T.E., Brasier, C.M., and Buck, K.W. (1998). Evolutionary relationships among putative RNA-dependent RNA polymerases encoded by a mitochondrial virus-like RNA in the Dutch elm disease fungus, Ophiostoma novo-ulmi, by other viruses and virus-like RNAs and by the Arabidopsis mitochondrial genome. *Virology* 246(1)**,** 158-169.

Hong, Y., Dover, S.L., Cole, T.E., Brasier, C.M., and Buck, K.W. (1999). Multiple mitochondrial viruses in an isolate of the Dutch Elm disease fungus Ophiostoma novo-ulmi. *Virology* 258(1)**,** 118-127.

Hu, Z., Wu, S., Cheng, J., Fu, Y., Jiang, D., and Xie, J. (2014). Molecular characterization of two positive-strand RNA viruses co-infecting a hypovirulent strain of *Sclerotinia sclerotiorum*. *Virology* 464-465**,** 450-459. doi: 10.1016/j.virol.2014.07.007.

Huang, S., and Ghabrial, S.A. (1996). Organization and expression of the double-stranded RNA genome of Helminthosporium victoriae 190S virus, a totivirus infecting a plant pathogenic filamentous fungus. *Proceedings of the National Academy of Sciences* 93(22)**,** 12541-12546.

Icho, T., and Wickner, R.B. (1989). The double-stranded RNA genome of yeast virus L-A encodes its own putative RNA polymerase by fusing two open reading frames. 264(12)**,** 6716-6723.

Kanhayuwa, L., Kotta-Loizou, I., S, Ö., Gunning, A.P., and Coutts, R.H. (2015). A novel mycovirus from *Aspergillus fumigatus* contains four unique dsRNAs as its genome and is infectious as dsRNA. *Proceedings of the National Academy of Sciences of the United States of America* 112(29)**,** 9100-9105.

Khalifa, M.E., and Pearson, M.N. (2014a). Molecular characterisation of an endornavirus infecting the phytopathogen Sclerotinia sclerotiorum. *Virus Research* 189**,** 303–309.

Khalifa, M.E., and Pearson, M.N. (2014b). Molecular characterisation of novel mitoviruses associated with Sclerotinia sclerotiorum. *Archives of Virology* 159(11)**,** 3157.

Kim, J.W., Shi, Y.K., and Kim, K.M. (2003). Genome Organization and Expression of the Penicillium stoloniferum Virus S. *Virus Genes* 27(3)**,** 249-256.

Koloniuk, I., Elhabbak, M.H., Petrzik, K., and Ghabrial, S.A. (2014). Complete genome sequence of a novel hypovirus infecting Phomopsis longicolla. *Archives of Virology* 159(7)**,** 1861.

Kotta-Loizou, I., and Coutts, R.H. (2017). Studies on the Virome of the Entomopathogenic Fungus Beauveria bassiana Reveal Novel dsRNA Elements and Mild Hypervirulence. *Plos Pathogens* 13(1)**,** e1006183.

Kwon, S.J., Lim, W.S., Park, S.H., Park, M.R., and Kim, K.H. (2009). Molecular characterization of a dsRNA mycovirus, Fusarium graminearum virus-DK21, which is phylogenetically related to hypoviruses but has a genome organization and gene expression strategy resembling those of plant potex-like viruses. *Molecules and Cells* 28(1)**,** 304-315.

Lee Marzano, S.Y., Nelson, B.D., Ajayi-Oyetunde, O., Bradley, C.A., Hughes, T.J., Hartman, G.L., et al. (2016). Identification of diverse mycoviruses through metatranscriptomics characterization of the viromes of five major fungal plant pathogens. *Journal of Virology* 90(15)**,** JVI.00357-00316.

Linder-Basso, D., Dynek, J.N., and Hillman, B.I. (2005). Genome analysis of Cryphonectria hypovirus 4, the most common hypovirus species in North America. *Virology* 337(1)**,** 192-203.

Liu, H., Fu, Y., Xie, J., Cheng, J., Ghabrial, S.A., Li, G., et al. (2012). Evolutionary genomics of mycovirus-related dsRNA viruses reveals cross-family horizontal gene transfer and evolution of diverse viral lineages. *BMC Evolutionary Biology* 12(1)**,** 91.

Liu, L. (2014). Fungal negative-stranded RNA virus that is related to bornaviruses and nyaviruses. *Proceedings of the National Academy of Sciences* 111(33)**,** 12205-12210.

Liu, L., Wang, Q., Cheng, J., Fu, Y., Jiang, D., and Xie, J. (2015). Molecular characterization of a bipartite double-stranded RNA virus and its satellite-like RNA co-infecting the phytopathogenic fungus Sclerotinia sclerotiorum. *Frontiers in Microbiology* 6**,** 406.

Mahua, S., and Bibha, K. (2006). Genetic variation of populations of Citrus psorosis virus. *Journal of General Virology* 87(87)**,** 3097-3102.

Marzano, S.Y., and Domier, L.L. (2016). Reprint of "Novel mycoviruses discovered from metatranscriptomics survey of soybean phyllosphere phytobiomes". *Virus Research* 213**,** 332-342.

Menzel, W., Maiss, E., and Vetten, H.J. (2008). Complete nucleotide sequence of a carrot isolate of Carrot mottle virus from Germany. *Archives of Virology* 153(11)**,** 2163.

Mihindukulasuriya, K.A., Nguyen, N.L., Wu, G.H., Da, R.A., Popov, V.L., Tesh, R.B., et al. (2009). Nyamanini and midway viruses define a novel taxon of RNA viruses in the order Mononegavirales. *Journal of Virology* 83(83)**,** 5109-5116.

Nomura, K., Osaki, H., Iwanami, T., Matsumoto, N., and Ohtsu, Y. (2003). Cloning and characterization of a totivirus double-stranded RNA from the plant pathogenic fungus, Helicobasidium mompa Tanaka. *Virus Genes* 26(3)**,** 219-226.

Oh, C.S., and Hillman, B.I. (1995). Genome organization of a partitivirus from the filamentous ascomycete Atkinsonella hypoxylon. *Journal of General Virology* 76 ( Pt 6)(6)**,** 1461-1470.

Osaki, H., Nomura, K., Iwanami, T., Kanematsu, S., Okabe, I., Matsumoto, N., et al. (2002). Detection of a double-stranded RNA virus from a strain of the violet root rot fungus Helicobasidium mompa Tanaka. 25(2)**,** 139-145.

Osaki, H., Sasaki, A., Nomiyama, K., Sekiguchi, H., Tomioka, K., and Takehara, T. (2015). Isolation and characterization of two mitoviruses and a putative alphapartitivirus from Fusarium spp. *Virus Genes* 50(3)**,** 466.

Park, C.M., Lopinski, J.D., Masuda, J., Tzeng, T.H., and Bruenn, J.A. (1996). A Second Double-Stranded RNA Virus from Yeast. *Virology* 216(2)**,** 451-454.

Park, Y., Chen, X., and Punja, Z.K. (2006). Molecular and Biological Characterization of a Mitovirus in Chalara elegans (Thielaviopsis basicola). *Phytopathology* 96(5)**,** 468.

Pfeiffer, P. (1998). Nucleotide sequence, genetic organization and expression strategy of the double-stranded RNA associated with the '447' cytoplasmic male sterility trait in Vicia faba. *Journal of General Virology* 79 ( Pt 10)(10)**,** 2349-2358.

Preisig, O., Moleleki, N., Smit, W.A., Wingfield, B.D., and Wingfield, M.J. (2000). A novel RNA mycovirus in a hypovirulent isolate of the plant pathogen Diaporthe ambigua. *Journal of General Virology* 81(Pt 12)**,** 3107-3114.

Preisig, O., Wingfield, B.D., and Wingfield, M.J. (1998). Coinfection of a Fungal Pathogen by Two Distinct Double-Stranded RNA Viruses ☆. *Virology* 252(252)**,** 399-406.

Ran, H., Liu, L., Li, B., Cheng, J., Fu, Y., Jiang, D., et al. (2016). Co-infection of a hypovirulent isolate of *Sclerotinia sclerotiorum* with a new botybirnavirus and a strain of a mitovirus. *Virol J* 13**,** 92. doi: 10.1186/s12985-016-0550-2.

Ren, P., Rajkumar, S.S., Sui, H., Masters, P.S., Martinkova, N., Kubatova, A., et al. (2016). Novel Partitivirus Infection of Bat White-nose Syndrome (WNS) Fungal Pathogen Pseudogymnoascus destructans Links Eurasian and North American Isolates.

Rodriguezcousiño, N., Esteban, L.M., and Esteban, R. (1991). Molecular cloning and characterization of W double-stranded RNA, a linear molecule present in Saccharomyces cerevisiae. Identification of its single-stranded RNA form as 20 S RNA. *Journal of Biological Chemistry* 266(19)**,** 12772-12778.

Rudolph, M.G., Kraus, I., Dickmanns, A., Eickmann, M., Garten, W., and Ficner, R. (2003). Crystal structure of the borna disease virus nucleoprotein. *Structure* 11(10)**,** 1219-1226.

Sabanadzovic, S., and Valverde, R.A. (2011). Properties and detection of two cryptoviruses from pepper ( Capsicum annuum ). *Virus Genes* 43(2)**,** 307-312.

Scheets, K. (2016). Analysis of gene functions in Maize chlorotic mottle virus. *Virus Research* 222**,** 71.

Shapira, R., Choi, G.H., and Nuss, D.L. (1991). Virus-like genetic organization and expression strategy for a double-stranded RNA genetic element associated with biological control of chestnut blight. *Embo Journal* 10(4)**,** 731-739.

Smart, C.D., Yuan, W., Foglia, R., Nuss, D.L., Fulbright, D.W., and Hillman, B.I. (1999). Cryphonectria hypovirus 3, a virus species in the family hypoviridae with a single open reading frame. *Virology* 265(1)**,** 66-73.

Szego, A., Enünlü, N., Deshmukh, S.D., Veliceasa, D., Hunyadigulyás, E., Kühne, T., et al. (2010). The genome of Beet cryptic virus 1 shows high homology to certain cryptoviruses present in phylogenetically distant hosts. *Virus Genes* 40(2)**,** 267-276.

Tai, J.H., and Ip, C.F. (1995). The cDNA sequence of Trichomonas vaginalis virus-T1 double-stranded RNA. *Virology* 206(1)**,** 773-776.

Takeuchi, K., Hishiyama, M., Yamada, A., and Sugiura, A. (1988). Molecular cloning and sequence analysis of the mumps virus gene encoding the P protein: mumps virus P gene is monocistronic. *Journal of General Virology* 69 ( Pt 8)(8)**,** 2043-2049.

Takimoto, T., Taylor, G.L., Crennell, S.J., Scroggs, R.A., and Portner, A. (2000). Crystallization of Newcastle disease virus hemagglutinin-neuraminidase glycoprotein. *Virology* 270(1)**,** 208-214.

Taliansky, M.E., Robinson, D.J., and Murant, A.F. (1996). Complete nucleotide sequence and organization of the RNA genome of groundnut rosette umbravirus. 77 ( Pt 9)(9)**,** 2335-2345.

Torok, V., and Vetten, H. (2003). Identification and molecular characterization of a new ophiovirus associated with lettuce ring necrosis disease. *Proceedings of Arbeitskreis Viruskrankheiten der Pflanzen, Heidelberg, Germany*.

Tuomivirta, T.T., and Hantula, J. (2003). Two unrelated double-stranded RNA molecule patterns in Gremmeniella abietina type A code for putative viruses of the families Totiviridae and Partitiviridae. *Archives of Virology* 148(12)**,** 2293-2305.

Tuomivirta, T.T., and Hantula, J. (2005). Three unrelated viruses occur in a single isolate of Gremmeniella abietina var. abietina type A. *Virus Research* 110(1–2)**,** 31-39.

Turina, M., Maruoka, M., Monis, J., Jackson, A.O., and Scholthof, K.B.G. (1998). Nucleotide Sequence and Infectivity of a Full-Length cDNA Clone of Panicum Mosaic Virus ☆ ☆☆. 241(1)**,** 141-155.

Vainio, E.J., Keriö, S., and Hantula, J. (2011). Description of a new putative virus infecting the conifer pathogenic fungus Heterobasidion parviporum with resemblance to Heterobasidion annosum P-type partitivirus. *Archives of Virology* 156(1)**,** 79-86.

Vemulapati, B., Druffel, K.L., Eigenbrode, S.D., Karasev, A., and Pappu, H.R. (2011). Genomic characterization of pea enation mosaic virus-2 from the Pacific Northwestern USA. *Archives of Virology* 156(10)**,** 1897-1900.

Volchkov, V.E., Volchkova, V.A., Chepurnov, A.A., Blinov, V.M., Dolnik, O., Netesov, S.V., et al. (1999). Characterization of the L gene and 5' trailer region of Ebola virus. *Journal of General Virology* 80 ( Pt 2)(2)**,** 355.

Wang, M., Wang, Y., Sun, X., Cheng, J., Fu, Y., Liu, H., et al. (2015). Characterization of a Novel Megabirnavirus from Sclerotinia sclerotiorum Reveals Horizontal Gene Transfer from Single-Stranded RNA Virus to Double-Stranded RNA Virus. *J Virol* 89(16)**,** 8567-8579. doi: 10.1128/JVI.00243-15.

Wang, S., Kondo, H., Liu, L., Guo, L., and Qiu, D. (2013). A novel virus in the family Hypoviridae from the plant pathogenic fungus Fusarium graminearum. *Virus Research* 174(1-2)**,** 69.

Wu, M., Deng, Y., Zhou, Z., He, G., Chen, W., and Li, G. (2016). Characterization of three mycoviruses co-infecting the plant pathogenic fungus Sclerotinia nivalis. *Virus Research* 223**,** 28–38.

Wu, M., Jin, F., Zhang, J., Yang, L., Jiang, D., and Li, G. (2012). Characterization of a Novel Bipartite Double-Stranded RNA Mycovirus Conferring Hypovirulence in the Phytopathogenic Fungus Botrytis porri. *Journal of Virology* 86(12)**,** 6605.

Wu, M., Zhang, L., Li, G., Jiang, D., and Ghabrial, S.A. (2010). Genome characterization of a debilitation-associated mitovirus infecting the phytopathogenic fungus Botrytis cinerea. *Virology* 406(1)**,** 117-126.

Xiao, X., Cheng, J., Tang, J., Fu, Y., Jiang, D., Baker, T.S., et al. (2014). A novel partitivirus that confers hypovirulence on plant pathogenic fungi. *Journal of Virology* 88(17)**,** 10120-10133.

Xie, J., and Ghabrial, S.A. (2012). Molecular characterization of two mitoviruses co-infecting a hypovirulent isolate of the plant pathogenic fungus Sclerotinia sclerotiorum [corrected]. *Virology* 428(2)**,** 77-85. doi: 10.1016/j.virol.2012.03.015.

Xie, J., Xiao, X., Fu, Y., Liu, H., Cheng, J., Ghabrial, S.A., et al. (2011). A novel mycovirus closely related to hypoviruses that infects the plant pathogenic fungus Sclerotinia sclerotiorum. *Virology* 418(1)**,** 49-56.

Xu, K., Chan, Y.P., Bradel-Tretheway, B., Akyol-Ataman, Z., Zhu, Y., Dutta, S., et al. (2015a). Crystal Structure of the Pre-fusion Nipah Virus Fusion Glycoprotein Reveals a Novel Hexamer-of-Trimers Assembly. *Plos Pathogens* 11(12)**,** e1005322.

Xu, Z., Wu, S., Liu, L., Cheng, J., Fu, Y., Jiang, D., et al. (2015b). A mitovirus related to plant mitochondrial gene confers hypovirulence on the phytopathogenic fungus Sclerotinia sclerotiorum. *Virus Res* 197**,** 127-136. doi: 10.1016/j.virusres.2014.12.023.

Yaegashi, H., and Kanematsu, S. (2015). Natural infection of the soil-borne fungus Rosellinia necatrix with novel mycoviruses under greenhouse conditions. *Virus Research* 219**,** 83-91.

Yaegashi, H., Kanematsu, S., and Ito, T. (2012). Molecular characterization of a new hypovirus infecting a phytopathogenic fungus, Valsa ceratosperma. *Virus Research* 165(2)**,** 143-150.

Yaegashi, H., Nakamura, H., Sawahata, T., Sasaki, A., Iwanami, Y., Ito, T., et al. (2013). Appearance of mycovirus-like double-stranded RNAs in the white root rot fungus, Rosellinia necatrix , in an apple orchard. *Fems Microbiology Ecology* 83(1)**,** 49–62.

Zhai, L., Xiang, J., Zhang, M., Fu, M., Yang, Z., Hong, N., et al. (2016). Characterization of a novel double-stranded RNA mycovirus conferring hypovirulence from the phytopathogenic fungus Botryosphaeria dothidea. *Virology* 493**,** 75-85.

Zhong, J., Lei, X.H., Zhu, J.Z., Song, G., Zhang, Y.D., Chen, Y., et al. (2014). Detection and sequence analysis of two novel co-infecting double-strand RNA mycoviruses in Ustilaginoidea virens. *Archives of Virology* 159(11)**,** 3063-3070.
