# Supplementary material for: Virome Characterization of a Collection of S. sclerotiorum from Australia
Source: Front Microbiol. 2018 Jan 11;8:2540. doi: 10.3389/fmicb.2017.02540 (PMC5768646; doi:10.3389/fmicb.2017.02540)
Supplement: Supplementary file 5 [file Table5.DOC]

**Table S5.** *Sclerotinia sclerotiorum* isolates from Australia used in this study.

| CODE | HOST | Description/collected from | DATE | AUSTRALIAN STATE | NOTES |
| --- | --- | --- | --- | --- | --- |
| S8 |  | unknown |  |  |  |
| S41 | *Lactuca sativa* | Vic DPI lettuce site 3 | 2009 | Vic |  |
| S44 | *L. sativa* | Weribee conference display site | 2009 | SA |  |
| S48 | *Phaseolus vulgaris* | Supermarket bean | 2009 | SA |  |
| S49 | *L. sativa* | Hydrofresh russo lettuce | 2009 | SA |  |
| S50 | *P. vulgaris* | TIAR ADD109 | 2009 | Tas |  |
| S51 | *P. vulgaris* | TIAR BDB409 | 2009 | Tas |  |
| S52 | *P. vulgaris* | TIAR BRA109 | 2009 | Tas |  |
| S59(1) | *P. vulgaris* | TIAR VIZ109 | 2009 | Tas | Mixed culture. Isolates not compatible |
| S59(2) | *P. vulgaris* | TIAR VIZ109 | 2009 | Tas | Mixed culture. Isolates not compatible |
| S61 | *Apium graveolens* | #2132, Harslett | 2008 | QLD |  |
| S62 | *L. sativa* | #2155, Schultz | 2008 | QLD |  |
| S63 | *Daucus carota* | #2159, Windley | 2008 | QLD |  |
| S64 | *P. vulgaris* | #2248, Buchanan Trial Site | 2009 | QLD |  |
| S66 | *P. vulgaris* | #2263, Millard | 2009 | QLD |  |
| S67 | *P. vulgaris* | #2264, Fentrill | 2009 | QLD |  |
| S69 | *Brassica oleracea* var*. capitata* | VL09, DPI Vic | 2009 | Vic |  |
| S70 | *Cichorium intybus ‘Witlof’* | VT08, DPI-Vic | 2009 | Vic |  |
| S71 | *L.sativa* | VL08, DPI-Vic | 2009 | Vic |  |
| S75 | *B. oleracea* var*. capitata* | Gingin | 2010 | WA |  |
| S77 | *Solanum tuberosum* | Myalup | 2010 | WA |  |
| S78 | *B. oleracea* var*. botrytis* | Hills Fresh, Gumeracha | 2010 | SA |  |
| UWA1 | *L. angustifolius* | Medina #11 | 2011 | WA |  |
| UWA2 | *B. napus* | Kendenup | 2009 | WA | Also called UWA10S2 |
| UWA3 | *B. napus* | East Chapman | 2009 | WA | Also called UWA7S3 |
| UWA4 | *L. angustifolius* | Medina #3 | 2011 | WA |  |
| WW3-2 | *B. napus* | Walkaway, Geraldton | 2004 | WA |  |
| AMB | *B. napus* | Attenuated MBRS-1 | 2004 | WA | Growth on PDA slow. No sclerotia produced. |
| UWABOC1 | *B. oleracea* var*. capitata* | Perth | 2008 | WA |  |
| UWADC1 | *D. carota* | Perth | 2008 | WA |  |
| MBRS1 | *B. napus* | Mount Barker | 2004 | WA |  |
| MBRS5 | *B. napus* | Mount Barker | 2004 | WA |  |
| UWA10S1 | *B. napus* | Kendenup, | 2009 | WA |  |
| UWA10S2 | *B. napus* | Kendenup, | 2009 | WA |  |
| UWA10S4 | *B. napus* | Kendenup, | 2009 | WA |  |
| UWA11S1 | *B. napus* | Mt. Baker | 2009 | WA |  |
| UWA11S2 | *B. napus* | Mt. Baker | 2009 | WA |  |
| UWA11S4 | *B. napus* | Mt. Baker | 2009 | WA |  |
| UWA11S5 | *B. napus* | Mt. Baker | 2009 | WA |  |
| UWA12S3 | *L.angustifolius* | Moonyoonooka | 2009 | WA |  |
| UWA12S4 | *L.angustifolius* | Moonyoonooka | 2009 | WA |  |
| UWA13S1 | *B. napus* | Naragulu, | 2009 | WA |  |
| UWA13S3 | *B. napus* | Naragulu, | 2009 | WA |  |
| UWA13S7 | *B. napus* | Naragulu, | 2009 | WA |  |
| UWA13S8 | *B. napus* | Naragulu, | 2009 | WA |  |
| UWA1S1 | *B. napus* | Narra Tarra | 2009 | WA |  |
| UWA1S2 | *B. napus* | Narra Tarra | 2009 | WA |  |
| UWA1S3 | *B. napus* | Narra Tarra | 2009 | WA |  |
| UWA1S4 | *B. napus* | Narra Tarra | 2009 | WA |  |
| UWA1S5 | *B. napus* | Narra Tarra | 2009 | WA |  |
| UWA2S1 | *B. napus* | Walkaway, | 2009 | WA |  |
| UWA2S2 | *B. napus* | Walkaway, | 2009 | WA |  |
| UWA2S3 | *B. napus* | Walkaway, | 2009 | WA |  |
| UWA3S1 | *B. napus* | Walkaway, | 2009 | WA |  |
| UWA3S2 | *B. napus* | Walkaway, | 2009 | WA |  |
| UWA3S4 | *B. napus* | Walkaway, | 2009 | WA |  |
| UWA3S5 | *B. napus* | Walkaway, | 2009 | WA |  |
| UWA4S1 | *B. napus* | East Chapman | 2009 | WA |  |
| UWA5S1 | *L. angustifolius* | Moonyoonooka | 2009 | WA |  |
| UWA5S2 | *L. angustifolius* | Moonyoonooka | 2009 | WA |  |
| UWA5S3 | *L. angustifolius* | Moonyoonooka | 2009 | WA |  |
| UWA6S1 | *L. angustifolius* | Walkaway | 2009 | WA |  |
| UWA6S2 | *L. angustifolius* | Walkaway | 2009 | WA |  |
| UWA6S3 | *L. angustifolius* | Walkaway | 2009 | WA |  |
| UWA6S4 | *B. napus* | Walkaway | 2009 | WA |  |
| UWA7S1 | *B. napus* | East Chapman | 2009 | WA |  |
| UWA7S2 | *B. napus* | East Chapman | 2009 | WA |  |
| UWA7S3 | *B. napus* | East Chapman | 2009 | WA |  |
| UWA7S4 | *B. napus* | East Chapman | 2009 | WA |  |
| UWA7S5 | *B. napus* | East Chapman | 2009 | WA |  |
| UWA8S1 | *B. napus* | East Chapman | 2009 | WA |  |
| UWA8S3 | *B. napus* | East Chapman | 2009 | WA |  |
| UWA8S4 | *B. napus* | East Chapman | 2009 | WA |  |
| UWA8S5 | *B. napus* | East Chapman | 2009 | WA |  |
| UWA9S1 | *B. napus* | Walkaway, | 2009 | WA |  |
| UWA9S2 | *B. napus* | Walkaway, | 2009 | WA |  |
| UWA9S3 | *B. napus* | Walkaway, | 2009 | WA |  |
| UWA9S4 | *B. napus* | Walkaway, | 2009 | WA |  |
| UWA9S5 | *B. napus* | Walkaway, | 2009 | WA |  |
| WW1 | *B. napus* | Walkaway | 2004 | WA |  |
| WW3 | *B. napus* | Walkaway | 2004 | WA |  |
| WW4 | *B. napus* | Walkaway | 2004 | WA |  |
| WW5-1 | *B. napus* | Walkaway | 2004 | WA |  |
| WW5-2 | *B. napus* | Walkaway | 2004 | WA |  |

**Note:** WA = Western Australia, Vic = Victoria, SA = South Australia, QLD = Queensland and Tas = Tasmania. See Ge *et al*. 2012 for additional location details of WA isolates.
