# Supplementary material for: Virome Characterization of a Collection of S. sclerotiorum from Australia
Source: Front Microbiol. 2018 Jan 11;8:2540. doi: 10.3389/fmicb.2017.02540 (PMC5768646; doi:10.3389/fmicb.2017.02540)
Supplement: Supplementary file 6 [file Table6.DOC]

**Table S6 The sequences of four virus after assembled**

| Sclerotinia sclerotiorum endornavirus 5 (SsEV5) | | |
| --- | --- | --- |
| The name of the contig | length(bp) | Primer |
| Ss-AA_clean.1_(paired)_contig_6278 | 617 | Sense primer:CGTTAGCACTATGTGGGTGGATG |
| Ss-AA_clean.1_(paired)_contig_15215 | 302 | Anti-sense primer:AATCGCTCAGGCTGTTTTGGT |
| CCTCGGGTGGCAGCAGAGATGCAGTATTTCCGGTTTACTTCAATACCAGCTTCACCACTCACTCTATGTTGGACAACCAACACGTTATCAGCCTCTTGTGACTGGAATGTGTGTGGAGTGTACCGATTAAGTTTGCGTATTGCGGTGGTGTGGTGGTACTGTTTGAGGGCATTCTTGAATTTGTTAGCTGTGGCATTGTGGAACGTCAGCAGGACGTGGACGTTGTGCTCAGCGGCCATTTTTATGATGCCATTGACGTCAAGCTCTTGTATTGCTGTGGTTGTCACGTCCGTAGTCCTGTCAGACAATGTTTTGAGACCAGGTATGACCTTGGCCAGCTCAAGTGCAAGTGGGTTGCCGTATCTCCTGGTCTCGGTCTTGGACTCTACGTCTTTAATATGTTTGAACAAATTGCTGCGCATCCTAGTGCCAGCTAGGGTATCCGTGTCTATAACTCCTATTTGATCTTCATCACCCATGATGAATATGTTGTCTGGTTCATGCTTCATTAATTGCATCAACGTGACGGGCTCGATCATGGTGGCTTCATCCACCCACATAGTGCTAACGTGCAAGTTGTCAGTTTGCAAGGCTCTCTCAACTGACATGACGCGATCTGTTGGTTTGTTGATCTTCTTTAGCTTGTCTTTGATGGATTGCACTCCGTTAGTGGTCATAGCAAGGGCCAAATCCGGGGTGGCCATGCGTTCAGCTAGCATGGTGGATTTGCCCCAACCACCAGTCGCCAACAGTCCTTTAGCTCTATTAAGAACGGACACCATGCGAACTGTATGATTCTGCGTGGTCAGCAAAGATATAATAGACCTCAAAGCACTTCCAGCAGACACCTTACTGTAGCCGATGGTCAATTTTGATGCTCCGGGTTTCTTGCTGATACCCAGAGACACCATACCGTTTTCAACTGTGATCTGCACTGGAGTCGGTTTATCGTAGTTAATGGTCTTCGGTAGGACGACCACATCTCCAGTTTTGAGCTTGGTAGAGCTAACATCAATGCACACTCTATTGCCATCTTTCGTCAGTATCGGGACGTCGATCCAGACCAATCTATTATCATTATCTAGTAGATGGTTGAAGCAGCACGCTTCTTTGTACTGATACACTTTAGCTCGGACTAGTTCTGGCATTTGGGCCCAAACTGCAGTTGGGTCATCTGTTTGGTCAAGTGGTTGTTTAAGCCAACTGTTGTTAATGGTTTGCGGGTCGTTGTTAACCAGCAATTTTACACTTTGTTCGTACCTATCAGGTACAGCAACATTGAATTTAGGTACTGGGTGTGGAACAAAATTAACATGAGCGTTGATTTCTGAGTCCACACAATTATACATGAGTCGCATTTGTCCACTTATCTTTGAAAATGATATCTCCCACAAGCCGGAGTTGGCCCACGAGGTATTCATAATGCTAGATTTGGCAATTAACAACTGCATCCTGGCATGTTGGACCTTAGTCAGGGTGAATGGATCAGGATCTGTCGCGGAACCAGCTGTTGCCCTTTTCACAGCAGTTCGAAGAACTGCTAAAGTGCAATTGTCTTGGAATGCTATCGGTCCCATGTGTATTTGCTGTTGCAATGCTTTCACTGGATACCAATGGTCTATACCTGTCGACTTTACTCTTGAAGAGTGAGCTAGAACAGGATACCAATCACTAAGTGTCATCTTTGTGATTCTAGTGCTCTCATTGGTGATTGTGATCAAGTTGCTTGATGTGTTGCGTGCTATGTTGTGCAGATCATTTTCGCTGAACCAGCCGTCTTTCTTAGCTATTCTAATCATAGTGTCTAAAGGCGGACCATTAGGGTCCAGGTAATGCCAACTAGTGTATCCGCATATGGCTCCAGGATTGGAATCTTCATACACAGTGATTATTTCCATCATATTGCTGGTGCCTGTCAACTGACATATTGGCATGAATGAGACGCGAGATCTGAACGGTTCGTCACCTGGCGTTATTTTGTCGACGAAGTTAACCGGTTCTTGATTTGATGAGGTAGCCATGACCAACATCGCAAACTGCAAATTTGTGCGCTCAATCAGTTCATTCAACAGGTCGTCTTCTTGCTCCGATTTCTTACTGTCTGATTTAGCAACCTCTTGTTTTGGCAAGACCTCTTTGGGTGTTGGTTGAACTTGCTCGGGTGCACCTGTTAATTCTCTCCGCTGTTCTGGTTGAGCCTGATCGATCGATGGAGCTGGCTTGACAAGTTTATTAGCGGTGTTGCCCTCACAAGCTGGGCAGTCACCCTTGAACAAGGGGTGCATTGTTGACTTAAACTTGTGTTGGTGCTGGTAGATCGCCCCACAGTGGCACAGGTGTTGGTGTAATCCGTCACCCAAATCATCACATGGTTGCAAAGCGGTTCTAACTTGCACAACTCTGCCATCAGGCGTGGTGTAATCGTGATAAGTTGTGGTAGGTTGGTCCTTAATTTGTTGTCTTGGTGTGGGCTTGGGCAATCGCTCAGGCTGTTTTGGTCTACTCGTTTTGATTGGCTCACCGGAATCAGTTGGCCAAACCCAGGTCTTGCCGTAGTCGATTGTCTCGTCGGGGACTGGCTCATCGCAAGACAAGCATGTAGCGTTTTCAGAATCCATCTTGACACCACAGCAGTTGCATACTTCACCTTCTTGATTTCCGTGACCAACACATGAGTGTTCACAAGCGTGCTGGCAGGGTGTTTCGCAACAAGGACACATCTCCGAGCTAGCTGGTTTACCACAACACTCACATTC | | |
|  |  |  |
| Sclerotinia sclerotiorum mitovirus 8-A(SsMV8) | | |
| The name of the contig | length(bp) | Primer |
| First_Contig42 | 765 | Sense primer:CAATAACTACATCATCACCCAGGAC |
| First_Contig43 | 529 | Anti-sense primer:GCGGTAACGCAGTCTGTGATG |
| CTCCGAAGACCAACCGTCACCTAGGAGCAGATGCTCCAAGGGATTGAAGGTCGAGGGAGTTCTGGTGTATGATGGCTCTCCGAACAAGAGAGTTCTCAAAACCAGACGGATATGACGAGAAACCCGACAGGGTAATAGAGTTCAGATATAGAAATCTGGAATTCTATTGCCTTTCGGACGGGCTGTTTGCCAAGTTAAAACATCAAAACGATGTTTAACCCGACGAACAGCATCAATAATCTGATTTGCTCGGACTGGAGTCAGCGAGCTAGACATCGTTAAAAACGATGCTAGCCCTACTCGAGTCGGAACAAAACCGAAAGGTCCTTTGATAGTCCACAGAATCGATTCCAGATGGCTCTGACGAACCGTCGGGACCCGACTCAACAGACTATCGACCGAGTCCTCGGTGAAAGTAACACCCTTCCCGCTCAAGTCCCGTAACAGAGATGGAACTCCGTTAAGAGACTGCAGAGCGAGAAGTGCATTACTAGCACCCAACGGAGTAAGTTCAACATCCCTAGACACCAATCTCTTTGCAAACTCGAAAGAATGCTCAGAGACTAGAGTCTTGGAAAGGTTAATCTTAACTCCGAGGATATCCACCATGATACTATGGTAGGACCTTGCGACCAGATCATTAGCAATAACTACATCATCACCCAGGACCGCGTAGTCGCTAAAATCGTGTAAAGAAACACGACGAGCAGCTAAACGGACTATGTAATGGTGTGTTAACGCTAACATACCCCATGAACTAAGGGCTCCCATGGGCTGACCAACGGCATAACGGAACTGCTCTTCCTCAGCTGGAAGACCAGGTACCGTCACCACCTTGGCAGACCAATCCCTGTCGGTCATCACTGACCGCCAGAGAGTACCGAACTGTTCACCGAAGTAGATGGACAGGATTTGCACCTGTAAATCTACGGGTAAACGGTCCGTGGCAGCACTTAGATCATAGGAGTAAAACTTCTGACCTTCTAATTTACCATCCCTCTTCAATTCTCGAAGACGGTCCAGGGGGGCACTTTGATTAAAAGTACCATCCTGGGGAACCCGTCGAAGACAATCAAAGATCGCGGCGGATAAGGGAGCCATCACAGACTGCGTTACCGCGTCAGTGATGGCGAAGATCCTTACCTTCCCCGCAGCCTCTTCCTTAAAGGACAACCGACCAAGGTGAAGAACCTTGGAAGGGAGGTCTCTAAGGGCAGAGACTTCGGAGAGAAGCCCTAATCGGAAGATCCTCGCCGTCGGGGAAAAGCTTGATAAAATCAAGCAATGACCCGAAGTTAGGACGAGATGCCCAGGCCTTGATATCTAATCAAATACCAAGACCGGCCACCTTACAATTAGGTCCCGCCGAACGGGAGAACAACCAACGGATCGGTCGCAGCTCCCCCAAAGGAGGGAGAGACGACTTAACCCGCTAGAAGTTCATAGATCGGAAGGGTAGAAGATAACCCAGTGAAGGGACCCACTATA | | |
|  |  |  |
| Sclerotinia sclerotiorum mitovirus 24 (SsMV24) | | |
| The name of the contig | length(bp) | Primer |
| Ss-AA_clean.1_(paired)_contig_24 | 293 | Sense primer:TTCCAGATTTCTAGCAGGTCAGC |
| NODE_1283_length_116_cov_53.844826 | 134 | Anti-sense primer:CGAGGTGATGACGAAACTATGCTA |
| CTCATCAAGATTTTATTATAAAACTAAACGGCCTTTAGGAGTTAAGGCGTGGATTTCCATAAAAGTAATTCCTCTGTATCTCCTGGTGGCCTCTTGATTGTTCAGTACATCTATTCATAGATCACATGTTAAGCTGTTTGCGACTAGGGTTGTTACCCTAGTTGAGAGATCTGGTTCTAACTTCACATTCCTGTATCTTAAGGAATGTTTTAGACTAGTTTCCAGATTTCTAGCAGGTCACCCGGATAAATGTCGTAAGATCTTTGTTCGCGCTGACAAAGATGGGTTACCGAAGATCATTCCTATCTCAATGCGTTTACTGTTACGTAGACGCCGTGAGAATCAGAAAGACATTCGGTTCATCCTGACGTTATTATCTGTCTTCCGTGTCCTTCCTACAACACCTGTTGTGTCATATGGTTCTATTGAGTCCGGTTTTACTGGGCTTTTTAAAACATTTGATACACGTCGTGCTGTACGAAGAATATGGAAGCATAAGATCAATATCGCCAAACTCCCTATATTTATTATAGGGGGTGAGTCAGCTGGGCCGAACTCCAAGAAGGCTGGTTGGGGGTCGTTGTTAGACGCTCTTGCGTTGTTACATCGACCTCTCCCCATCCTTCGGTATTTAGTAAAGTCAAAATCTTTATTCTGACTTATTTGATTACTAAATATTTGATTGTTGTTCGGGCCTCTTTACTTGATCATTAGTTTCACAACACGGTCTCGTTTGGAAGAAGGGAGACTATCTGTTGTGCGTGATCAAGCAGGAAAAGCCAGGGTAGTTGCTATAACTAACTGGTGAATACAACTGGTTCTTAGACCGCTCCATCGGAAGTTATTTTCAATTCTTAAGAAAATTCCTACTGATGGAACCTTTGATCAGATGGCACCTATTCAGCGTTTAGCTGATAAGGCGTCTTCTGATTTAGAGGCGGGTCTAGATCCGGTTGTATTATCCTGTTTTGATCTAACCGCAGCAACGGATCGACTACCGGTTGATCTTCAATCTGATATTTTATCAGTTTTAGGTTTTCCGGGGCCGATCTGACGTAGTCTTCTCGACATCAGTTGGTTAACGCCAGAACACTTGTACCATATCAAGTATTCTGTGGGTCAACCAATGGGTGCCTATTCCTCATGAGCCATGTTGGCTATGACACATCATGTGATTGTCCAAGAAGCTGCAGCCCGTTCAGGGTTCGTGGCTTTCTTTGATGATTACGCTATCCTTGGAGACGACATTGTCATTCGTAATGACTCTGTTGCTCACCAGTATAGCTTGTTGATGGAAGGTCTTGGTTTGGAAATTAACCCTTACAAGTCCATATCATCTTCGGATTTTATGGAGTTTGCTAAGAAGTTAATTGGTCCCGACCTAGACTATACGCCGTTTGGTCCTGGACTTATTGTCCAAGCCATTCGGAATAGAGGAATTGTCTCTTCTGTTATAAGAGAGGCTATATCCTGAAAATCATCACTTGA | | |
|  |  |  |
| Sclerotinia sclerotiorum mitovirus 26 (SsMV26) | | |
| The name of the contig | length(bp) | Primer |
| First_Contig87 | 1350 | Sense primer:AGGGAGTGCCGAGGTAAAGTG |
| Ss-AA_clean.1_(paired)_contig_634 | 1262 | Anti-sense primer:TCAGACCATCCATAAATGCTTCC |
| GACCCTGGGCCCCTATTGGGGACCAAGGGAGTGCCGAGGTAAAGTGGCACCCCCGCAGGTCTCAATAGGGGTCTTAGGAGTAATCCTAAGGGAGTCGACATGACTATAACTGCCCGGAGCTGGCTGTAGGGGCTAATATTCCCTGCAGTGATCTCCGGCCCGTTAACTCGGGATCACAACCTCCTCAACTTATTACAAAATACACTAGAACAACAAAATACAAATGATTTGTCGCTCTAGTGAGTAATACATTGAGTTGGATCATTTGGGCGTCAAGCCCAAATGGACTGGCTCTACGGGATTTTCAACCCTTCCTTGAGCACATGGAAGCAATTTTAATAGCTCGCGGACCTAGAGGTCTGATCGAGATGTTAAAATTGTCTCGTATGTGTGTAGTTAACTACTTGTCAGGTAGTGACCTACGTCCCAAGGGAGTTAGGTTGGTCACGAAGGCTAGGCTACCCTACTCTTTAGGTCCTCTTATTAATAAGGTCATAGATGGCGACCCACTAAACGTGCGTCTCATCATGACGGTACTTTTCAGTACTAGGGCCTTAAAGCTAGAGTCCAAACCGGATCTAAGTCCCATTACGGCTCCCCTGAAAGATGGGTGCAACCTTAATGTTGGACTATTCGGTGGGGACTTCTGGCGCGAGTTGGGTTTCCAGCATTCGGGAACGGTCCCACGACCATTGAGATGACGAAAGTTTCACTTCACCACCAAATCAGGTCCGACGTCATGTCACGCTTTGTGATCATGACTGTCAGACTTTGATTTGTTAACACCTGATATGATAAAAAATATCGGGGTGTTAGGTGGAGAGAAAATTTCCCATCTAATGGACGTGTTCCGGGCTAATTTTGATTTCCTGCGGGAGATCCACCGAGAGGATTTCCGAGAAATTCCCTTTACAAGGAAAATCTCGTCATTTCCAGATCGGGAAGGTAAGACTAGGGTAATCGCCATTGGTGATTACTTTAGTCAAACCGTCTTAAGGGCTCTGCATAATTACTTATTCAGAATCCTTAAGAGGATCCCTCAGGATTTCACTTTTAACCAGGAAGCATTTATGGATGGTCTGAAGGGAGCAACGGAATATCATTCCATTGATCTTACTTCAGCCACCGACCGATTCCCTATAAAGGTAATCGAGTTGGTGCTTTCCAGCCATCTTCCGGCCTCCTATGTTAAGGCCTGGAGAGATCTTATGGTGGGAGAGCCCTTTATGGTGTCTACCGGCCCCGGTAGGGGAAAGGCTTACAAGCCTATCCGCTATGAGGTTGGTAACCCAATGGGGTTCTACTCATCATGAGCATCTTTCGCAATCGCTCATCACTATATAGTCTACTATTGTTGTAGGCTAGAAGGTGTTGAGTGACGGACCTTACCTTATTGTCTCTTAGGAGACGATATTGTAATAGGAGATGGAAGAGTTGCCAGTCGATATCTGTCGGTTATGAATGACCTTGGGGTGGATGTATCCCCTTTAAAAACTCATCATAGTAAAACTATTCTTGAGTTTGCAAAGAGATGAATCCACAAAGGTCATGAGATAACGCCCTTCCCTATTTCAGCCATGAAGCAGAGCACCAAGAGATCCTATCTCTTGGCCGCCCTGTTAACGGAAGAAATGGTTAAGAAGGGTTGGCAGTTTAAGCCTGTCGACGTTATGGTTGAAAGGTGGTATGGACAGTTTAGGAGAATGCCTTCGCGTTTAACGAAGGTGTTCTCAAAAACTGCCTACCTGACTAACAACATGATTAAAATCATGTGAGGAGTCTCTCCGGCTGGTTCGTCTCTTACTGAGATGGCCAGGCGGGTAGGGATCATCCTAAAGCCCATTCCTGATGAAGTAGGGACTAACATCCTTGCTAACATCATGGTGGAGCTATTTAGTCAGTCCAACGCGAGATTTGATTCCAAGGCTACTACCGGAAAACCGTTAGGTTTAATGGCAGAAACCTTGGTGATGCAAATCACTGGTCGGGACC | | |
